# A Desilylative Approach to Alkyl Substituted C(1)‐Ammonium Enolates: Application in Enantioselective [2+2] Cycloadditions

**DOI:** 10.1002/anie.202208800

**Published:** 2022-08-08

**Authors:** Yihong Wang, Claire M. Young, Honglei Liu, Will C. Hartley, Max Wienhold, David. B. Cordes, Alexandra M. Z. Slawin, Andrew D. Smith

**Affiliations:** ^1^ EaStCHEM School of Chemistry University of St Andrews St Andrews Fife KY16 9ST UK

**Keywords:** β-Lactone, Cycloaddition, Desilylation, Enolate, Isothiourea

## Abstract

The catalytic generation of C(1)‐ammonium enolates from the corresponding α‐silyl‐α‐alkyl substituted carboxylic acids using the isothiourea HyperBTM is reported. This desilylative approach grants access to α‐unsubstituted and α‐alkyl substituted C(1)‐ammonium enolates, which are typically difficult to access through traditional methods reliant upon deprotonation. The scope and limitations of this process is established in enantioselective [2+2]‐cycloaddition processes with perfluoroalkylketones (31 examples, up to 96 % yield and >99 : 1 er), as well as selective [2+2]‐cycloaddition with trifluoromethyl enones (4 examples, up to 75 % yield and >99 : 1 er). Preliminary mechanistic studies indicate this process proceeds through an initial kinetic resolution of an in situ prepared (±)‐α‐silyl‐α‐alkyl substituted anhydride, while the reaction process exhibits overall pseudo zero‐order kinetics.

## Graphical abstract

The catalytic generation of unsubstituted and alkyl substituted C(1)‐ammonium enolates from α‐silyl‐α‐alkyl substituted carboxylic acids using the isothiourea HyperBTM has been developed and applied in enantioselective [2+2]‐cycloaddition reactions.

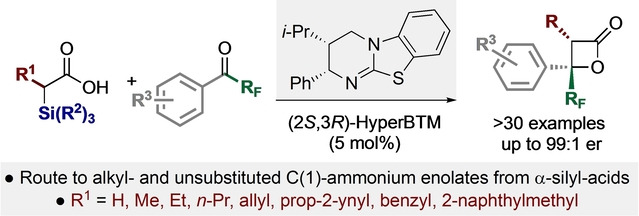

## Introduction

**Figure 1:**
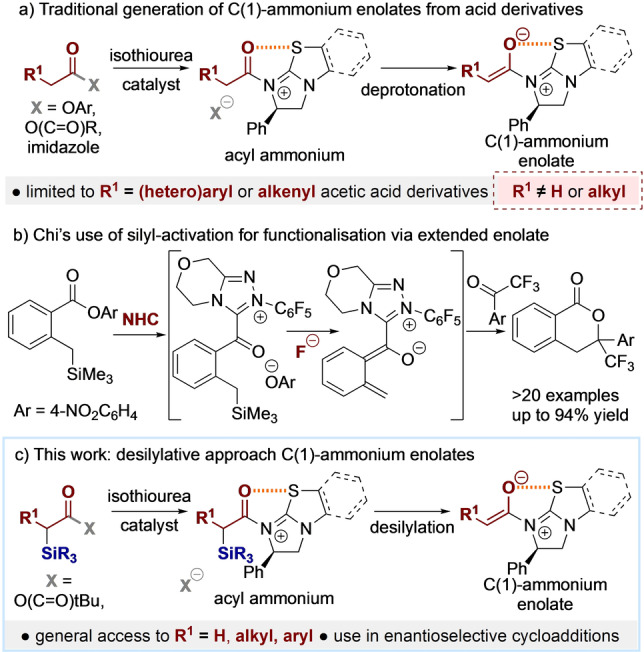
a) Traditional method of C(1)‐ammonium enolate generation from carboxylic acids. b) Chi's desilylative functionalisation of benzylic substituents. c) Proposed desilylative generation of C(1)‐ammonium enolates from carboxylic acids using isothioureas.

C(1)‐Ammonium enolates are recognised as important and useful synthetic intermediates that react with electrophiles (such as a reactive ketone, enone or palladium‐π‐allyl species) to generate stereodefined products with high enantioselectivity.^[1]^ These C(1)‐ammonium enolate species are traditionally derived from the reaction of Lewis basic tertiary amines with either acid chlorides or ketenes (either pre‐formed or prepared in situ*)* as starting materials.^[2]^ In recent years, focus in this area has shifted to enable their use from carboxylic acids (via an in situ formed mixed anhydride^[3]^ or ester derivative^[4]^), acyl imidazoles,^[5]^ or electron deficient aryl esters,^[6]^ with isothioureas proving particularly effective Lewis base catalysts.^[7]^ Despite significant advances, one common limitation within this area is the restricted structural variation allowed within the α‐substituted carboxylic acid derivative in *intermolecular* reactions. A necessary constraint is that processes require an α‐aryl‐, α‐heteroaryl, or α‐alkenyl‐substituted derivative, with only extremely limited exceptions within the literature (Figure 1A).^[8]^ As C(1)‐ammonium enolate generation using this strategy requires initial formation of an acyl ammonium ion pair, followed by deprotonation, this structural bias may be due to these substituent patterns leading to increased acidity and facilitating deprotonation. To address this limitation, an alternative strategy applicable to the generation of a range of unsubstituted and α‐alkyl C(1)‐ammonium enolates in a one‐pot protocol from carboxylic acids is reported. Circumventing deprotonation of an intermediate acyl ammonium species, alternative pathways for the generation of the desired C(1)‐ammonium enolate were considered. In particular, the work of Chi and co‐workers was invoked who used silicon‐based precursors to functionalise the benzylic position within 2‐[trimethylsilyl]methyl benzoates using the combination of fluoride and NHC catalysts (Figure 1B).^[9]^ Building upon this work, herein the development of α‐silyl‐α‐alkyl substituted carboxylic acids as precursors to a range of unsubstituted and α‐alkyl substituted C(1)‐ammonium enolates, alongside an evaluation of their scope and limitations in enantioselective [2+2]‐cycloaddition processes with perfluoroalkylketones and trifluoromethylenones is demonstrated (Figure 1C).

## Results and Discussion

### Investigation of Optimal Reaction Conditions

The effective generation of an unsubstituted C(1)‐ammonium enolate from α‐trimethylsilyl acid **1**, and its formal [2+2]‐cycloaddition with trifluoromethyl ketone **2** to give β‐lactone **3**, was chosen as the initial target for optimisation. Limited catalytic methods for the generation of such chiral acetyl enolate equivalents have been demonstrated. NHC‐catalysed approaches using α‐functionalised acetaldehydes or acetate esters are known,^[10]^ while current routes using tertiary amine catalysts rely on in situ ketene generation from acetyl chloride.^[11]^ Initial formation of the corresponding mixed anhydride from acid **1** (2 equiv) and pivaloyl chloride (3 equiv) in MTBE, prior to addition of (2*S*,3*R)‐*HyperBTM **4** (5 mol%) and ketone **2** (1 equiv) at room temperature, gave the β‐lactone **3** in excellent yield and enantioselectivity (96 % yield, 94 : 6 er) (Table 1, entry 1). Further experimentation indicated that reduced stoichiometry of pivaloyl chloride, base or HyperBTM still led to high product enantiocontrol, but with reduced yield even with extended reaction times (entries 2–4). The use of alternative isothioureas **5** and **6** led to significantly reduced reactivity (less than 10 % conversion to product) (entries 5 and 6), while alternative solvents also gave lower yields and/or enantioselectivity (entries 7–9).^[12]^

**Table 1:** Variation of reaction conditions.^[a]^

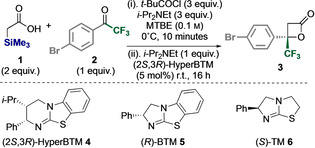			
Entry	Variation	Yield^[b]^ [%]	er^[c]^
1	–	96	94 : 6
2	Acid **1** (1 equiv)	36	90 : 10
3	*t*‐BuCOCl (2 equiv), *i*‐Pr_2_NEt (2 equiv)	82	94 : 6
4	As entry 3 but 24 hours	88	94 : 6
5	(*R)‐*BTM **5** (5 mol%)	<10^[d]^	–
6	(*S*)‐tetramisole **6** (5 mol%)	<10^[d]^	–
7	MeCN (0.1 M)	55	71 : 29
8	CH_2_Cl_2_ (0.1 M)	82	79 : 21
9	toluene (0.1 M)	98	84 : 16

[a] *t*‐BuCOCl (1.2 mmol), *i*‐Pr_2_NEt (1.2 mmol) and acid **1** (0.8 mmol) in MTBE (4 mL, 0.1 m) was stirred at 0° C for 10 minutes before addition of *i*‐Pr_2_NEt (0.4 mmol), ketone **2** (0.4 mmol) and (2*S*,3*R)‐*HyperBTM **4** (5 mol%) at r.t. for 16 h. [b] Isolated yield. [c] Determined by HPLC analysis on a chiral stationary phase. [d] Determined by ^1^H NMR analysis of the crude reaction product. MTBE=methyl *tert*‐butyl ether. r.t.=room temperature. HyperBTM=3‐isopropyl‐2‐phenyl‐3,4‐dihydro‐2*H*‐benzo[4,5]thiazolo[3,2‐*a*]pyrimidine. BTM=benzotetramisole. TM=tetramisole.

### Scope and Limitations

With the optimal conditions (Table 1, Entry 1) established, the generality of this protocol was investigated through variation of both the α‐silyl‐ and α‐alkyl‐substituents within the carboxylic acid, alongside the aryl‐ and perfluoroalkyl‐substituents within the ketone (Table 2). Initially, the effect of variation of the α‐alkyl‐substituents within the carboxylic acid reaction component under the developed conditions was investigated (Table 2A). Preparation of a range of α‐SiMe_3_‐α‐alkyl carboxylic acids, and their application under the developed reaction conditions provided access to a range of C(3)‐alkyl substituted β‐lactone products, with C(3)‐methyl, ethyl, *n*‐propyl, allyl, prop‐2‐ynyl, (substituted) benzyl and 2‐naphthylmethyl substituents all successfully incorporated, giving the corresponding β‐lactones **7**–**15** in good yields (up to 87 %) and excellent enantioselectivity (95 : 5 to >99 : 1 er). As a control experiment, α‐phenyl‐α‐trimethylsilyl carboxylic acid was used in this protocol, giving the C(3)‐Ph‐β‐lactone **16** in high dr and er. The relative and absolute configuration within both **7** and **16** were proven by comparison with the literature, with that within **16** identical to that prepared from the corresponding phenylacetic acid.^[13]^

**Table 2:**
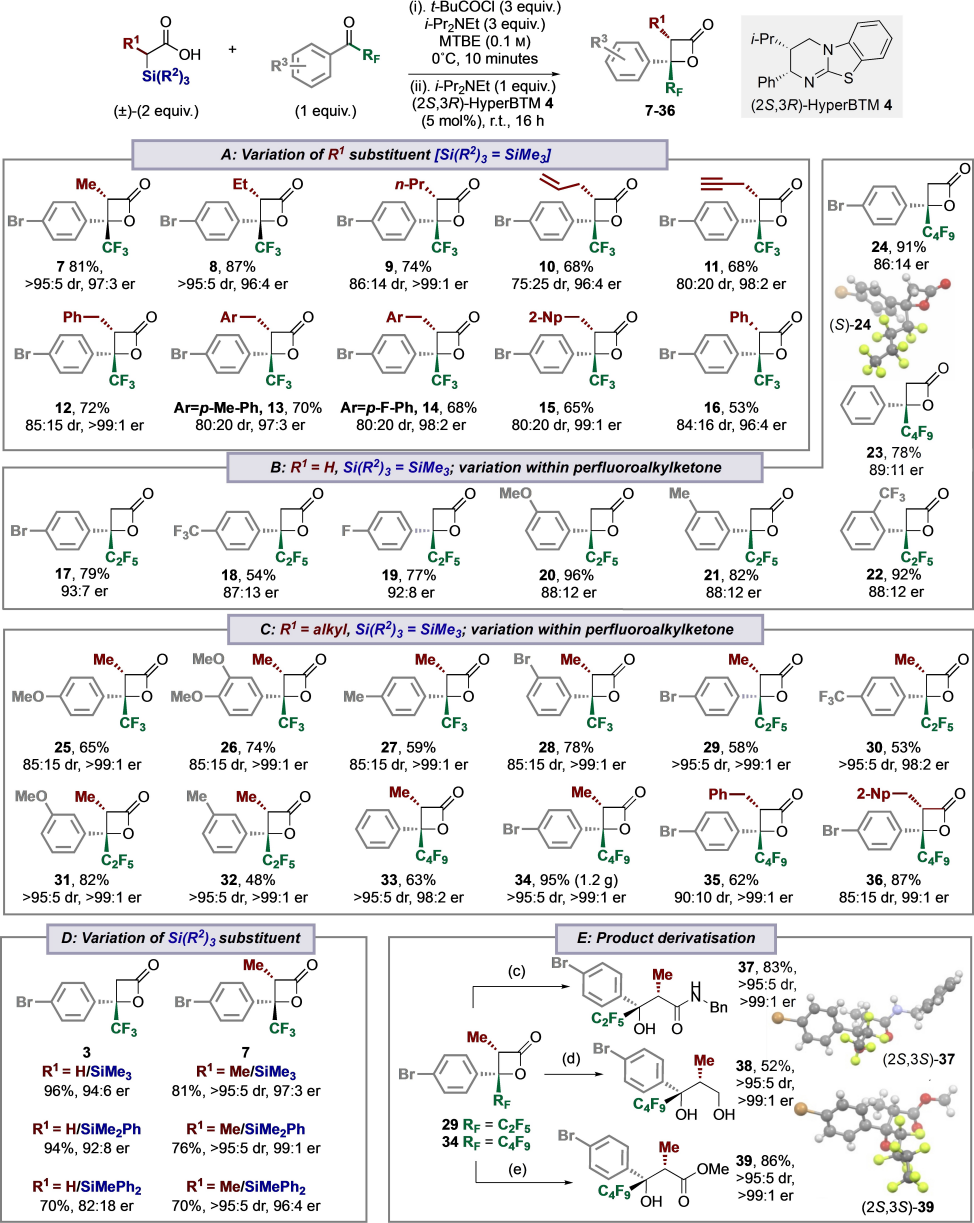
Scope and limitations of the enantioselective [2+2]‐cycloaddition using (±)‐α‐silyl‐α‐alkyl‐carboxylic acids as C(1)‐ammonium enolate precursors.^[a,b]^

[a] Isolated yield; [b] dr determined by ^1^H NMR analysis of the crude reaction product; er determined by HPLC analysis on a chiral stationary phase; [c] **29** (1.0 equiv), NH_2_Bn (2.0 equiv) CH_2_Cl_2_ (0.5 M), r.t., 16 h; [d] **34** (1.0 equiv), DIBAL (2.0 equiv), CH_2_Cl_2_ (0.1 M), −78 °C, 90 min; [e] **34** (1.0 equiv), NaOMe (5.0 equiv), MeOH, −4 °C, 16 h.

Further demonstration of the scope and limitations of this process focused upon the reactivity of the unsubstituted C(1)‐ammonium enolate generated from α‐trimethylsilyl acid **1** through reaction with a range of perfluoroalkylketones (Table 2B). Using the developed protocol, variation to include perfluoroethyl and perfluorobutyl ketones was tested, with the electronic nature of the aromatic substituent of the ketone also varied. Electron‐withdrawing substituents (with positive Hammett σ‐constants such as *para*‐CF_3,_ *ortho*‐CF_3,_ *meta*‐OMe), as well electron‐neutral or weakly electron‐donating groups (with negative Hammett σ‐constants)^[14]^ were well tolerated, giving β‐lactone products **17**–**24** in good to excellent yield and high enantioselectivity (85 : 15 to 94 : 6 er). The absolute configuration of (*S*)‐**24** was determined by single crystal X‐ray crystallography, with all other products assigned by analogy.^[15]^ Further structural variation focused upon demonstrating the reactivity of alkyl substituted C(1)‐ammonium enolates generated in this method (Table 2C). Using α‐trimethylsilyl‐α‐methyl acetic acid as a standard, its reactivity with a range of perfluoroalkylketones was demonstrated, with the introduction of both strongly electron‐donating (such as *para*‐OMe) and electron‐withdrawing substituents (such as *para*‐CF_3_) tolerated, leading to the formation of β‐lactones **25**–**33** in good to excellent yield (53 % to 95 %) and with excellent diastereo‐ and enantioselectivity (from 85 : 15 to >95 : 5 dr, up to >99 : 1 er). The preparation of **34** was performed on gram scale, giving β‐lactone **34** (1.2 g) in 95 % yield, >95 : 5 dr and >99 : 1 er. Further extension of this protocol to the generation of C(3)‐benzyl‐ and C(3)‐2‐naphthylmethyl substituted perfluorobutyl substituted β‐lactones was successful, generating **35** and **36** in good to excellent yield and enantiocontrol (85 : 15 to 90 : 10 dr, >99 : 1 er).

Further studies probed variation of the silyl‐substituent (Table 2D). The incorporation of α‐SiMe_3_, α‐SiMe_2_Ph and α‐SiMePh_2_ groups within the carboxylic acid were all shown to be effective C(1)‐ammonium enolate precursors. Using the corresponding unsubstituted α‐silyl acids gave β‐lactone **3** in good yields in each case, although reduced er was observed using the α‐SiMePh_2_ substituent. Extending this protocol to the corresponding α‐methyl‐α‐silyl‐carboxylic acids showed that α‐SiMe_3_, α‐SiMe_2_Ph and α‐SiMePh_2_ groups were tolerated, each giving C(3)‐Me‐β‐lactone **7** in >95 : 5 dr and excellent enantiocontrol (up to >99 : 1 er). Facile derivatisation of the β‐lactone products **29** and **34** to the corresponding amide **37** (from **29**), as well as alcohol **38** or ester **39** (from **34**) was also demonstrated (Table 2E) with exclusive ring‐opening at C(2) observed as confirmed by single crystal X‐ray crystallography.^[15]^

The scope and limitations of this methodology was then investigated through its application to reaction with trifluoromethyl enones (Table 3). These enones have been previously used in a range of enantioselective [4+2]‐cycloaddition methodologies with enolate equivalents, for example using NHCs, secondary amines via enamine catalysis, and isothioureas via C(1)‐aryl substituted ammonium enolates.^[16]^ Interestingly, the application of α‐trimethylsilyl carboxylic acid **1** in the reaction with CF_3_ enone **40** resulted in exclusive [2+2]‐addition, giving the β‐lactone **42** in 92 : 8 er. The use of an α‐methyl‐α‐trimethylsilyl‐carboxylic acid with enone **40** gave preferential [2+2]‐cycloaddition over [4+2]‐cycloaddition (90 : 10 ratio), with C(3)‐Me‐β‐lactone **43** isolated in 75 % yield, with excellent stereocontrol (>95 : 5 dr and 99 : 1 er). The incorporation of an α‐Me‐substituent within the trifluoromethylenone **41** led to exclusive [2+2]‐cycloaddition in reaction with α‐methyl‐α‐trimethylsilyl‐carboxylic acid, giving C(3)‐Me‐β‐lactone **44** in 65 % yield, >95 : 5 dr and >99 : 1 er. Application of this method using α‐phenyl‐α‐trimethylsilyl‐carboxylic acid led exclusively to [4+2]‐cycloaddition, giving **45** in 70 % yield, 92 : 8 er and 96 : 4 er. The relative and absolute configuration within **45** was confirmed by comparison with the literature and is identical to that prepared from the corresponding phenylacetic acid.^[16e]^ These results indicate that the C(1)‐substituent of the ammonium enolate generated using isothioureas plays a significant role in dictating [2+2] or [4+2] cycloaddition in reactions with trifluoromethylenones. C(1)‐alkyl/unsubstituted enolates favour [2+2]‐cycloaddition, while aryl‐substituents give [4+2]‐cycloaddition.

**Table 3:**
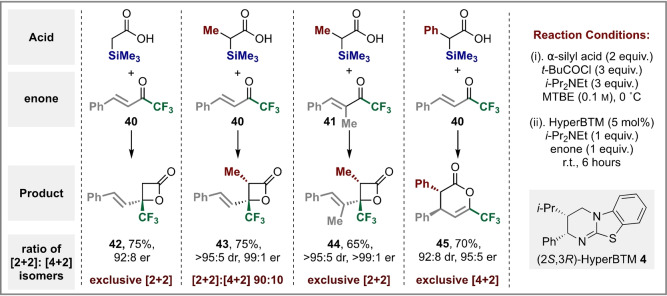
[2+2]‐ versus [4+2]‐formal cycloaddition using α‐silyl‐carboxylic acids as C(1)‐ammonium enolate precursors.^[a,b]^

[a] Isolated yield; [b] dr determined by ^1^H NMR analysis of the crude reaction product; er determined by HPLC analysis on a chiral stationary phase.

### Preliminary Mechanistic Investigations

Further investigations focused on developing mechanistic insights. Control studies demonstrated the significant beneficial effect of the incorporation of an α‐silyl substituent within the carboxylic acid (Table 4A). Treatment of either acetic acid or acetic anhydride under the developed conditions gave the β‐lactone **3** in significantly decreased product yields (32 % and 36 % respectively) but similar enantioselectivity to the corresponding α‐trimethylsilyl carboxylic acid. Similarly, the use of propionic acid or propionic anhydride as starting material also led to significantly reduced yields (10 % and 16 %) of β‐lactone **7**.

**Table 4:**
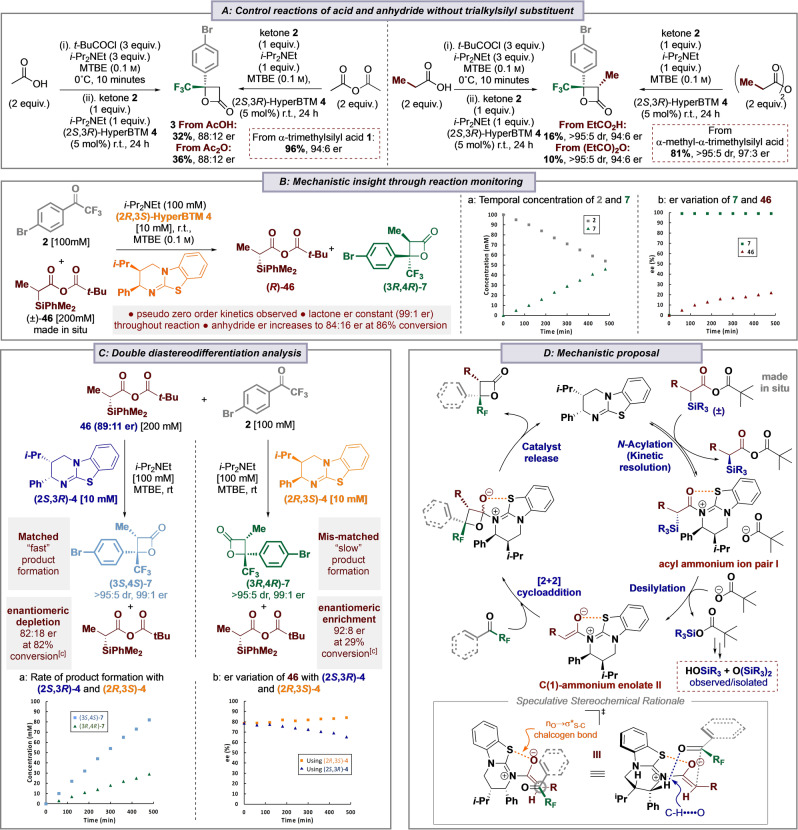
Mechanistic control studies and proposed outline mechanism for desilylative generation of α‐alkyl C(1)‐ammonium enolates.^[a,b]^

[a] Isolated yield; [b] dr determined by ^1^H NMR analysis of the crude reaction product; er determined by HPLC analysis on a chiral stationary phase; [c] determined by ^19^F NMR analysis of the crude reaction product with respect to conversion of ketone **2** to product **7**.

The role of using *racemic* α‐alkyl‐α‐silyl‐carboxylic acids as starting material in this process and any potential enantiodiscrimination was studied. Using the generation of β‐lactone **7** from (±)‐α‐methyl‐α‐dimethylphenylsilyl‐carboxylic acid **46** as a model, direct in situ temporal reaction monitoring by ^19^F{^1^H} NMR spectroscopy proved difficult as the generation of *i*‐Pr_2_NEt⋅HCl during the formation of the intermediate mixed anhydride led to a heterogeneous reaction mixture. Instead, samples were taken throughout the reaction, and analysis of these by HPLC, ^1^H and ^19^F{^1^H} NMR, after filtration and work up, allowed reproduceable datasets to be collected. These data allowed the concentration of ketone **2** and product β‐lactone **7** to be monitored quantitatively throughout the reaction (Table 4B). Notably, the formation of β‐lactone **7** correlated directly with the consumption of ketone **2** throughout the reaction. Monitoring consumption of the mixed anhydride **46** showed that its concentration deviated significantly from that expected based on the concentration of β‐lactone **7** and ketone **2**, with the silanol (HOSiPhMe_2_) and siloxane (O(SiPhMe_2_)_2_) the only observable by‐products.[^12^, ^17^] HPLC analysis on a chiral stationary phase allowed the er of both the lactone **7** and the mixed anhydride **46** to be measured as the reaction proceeded. Using (2*R*,3*S)‐*HyperBTM, these studies indicated that the er of β‐lactone **7** (99 : 1 er) was independent of product conversion, while the mixed anhydride **46** became progressively enriched in the (*R*)‐enantiomer, reaching 61 : 39 er after 480 minutes (at 46 % conversion of ketone to β‐lactone **7**), and 84 : 16 er at 86 % conversion to product. Notably a linear relationship between product concentration and time was observed, indicating that the reaction exhibits overall pseudo zero‐order reaction kinetics.[^18^, ^19^] Intrigued by these observations, enantiomerically enriched (*R*)‐anhydride **46** (89 : 11 er) was prepared,^[20]^ with the effect of using both (2*R*,3*S*)‐ and (2*S*,3*R*)‐enantiomers of HyperBTM catalysts under the developed conditions investigated (Table 4C). In each case, the corresponding enantiomeric β‐lactones were prepared in >95 : 5 dr and >99 : 1 er (independent of conversion) but at markedly different rates, consistent with a stereochemically matched and mismatched reactant pairing. Using (2*R*,3*S)‐*HyperBTM a concurrent enrichment in the er of remaining anhydride (*R*)‐**46** (to 92 : 8 er at 29 % conversion to β‐lactone **7** after 480 mins) was observed, with a corresponding depletion in the er of the (*R*)‐anhydride **46** when using (2*S*,3*R)‐*HyperBTM (to 82 : 18 er at 82 % conversion to β‐lactone **7** after 480 mins). Control studies showed that treatment of enantiomerically enriched anhydride **46** (89 : 11 er) with *i*‐Pr_2_NEt with either enantiomer of HyperBTM returned anhydride **46** with unchanged enantiomeric ratio.

Building upon these observations a tentative mechanism for the developed process is suggested (Table 4D). Catalysis is initiated by acylation of the isothiourea catalyst (2*S*,3*R)‐*HyperBTM **4** by the (±)‐α‐alkyl‐α‐silyl mixed anhydride, with preferential acylation of the (*R*)‐enantiomer leading to kinetic resolution, furnishing enantioenriched (*S*)‐anhydride and acyl ammonium ion pair **I**. Desilylation to generate the corresponding (*Z*)‐ammonium enolate **II** is hypothesised to be promoted either through direct substitution (either by the pivalate counterion or an alternative nucleophile such as chloride, water), or through a Brook‐type C‐ to *O*‐silyl rearrangement followed by pivalate or nucleophile promoted *O*‐desilylation.^[21]^ Although the silyl ester of pivalic acid could not be observed, the corresponding silanol (HOSiPhMe_2_) and siloxane (O(SiPhMe_2_)_2_) were identified and isolated in the reactions of **46** outlined in Table 4C. Key to the observed stereochemical outcome is a stabilizing 1,5‐O⋅⋅⋅S chalcogen bonding interaction (n_O_ to σ*_S−C_)[^22^, ^23^, ^24^, ^25^] that provides a conformational bias and ensures coplanarity between the 1,5‐O‐ and S‐ atoms within the (*Z*)‐enolate, with preferential addition *anti*‐ to the stereodirecting phenyl substituent within the catalyst. The observed relative and absolute configuration within the β‐lactone products is consistent with that observed in the previously reported [2+2]‐cycloaddition of aryl‐substituted C(1)‐ammonium enolates and trifluoromethylketones.^[13]^ By analogy, a similar concerted asynchronous [2+2]‐cycloaddition pathway via transition state assembly **III**, that allows for a stabilizing non‐classical CH⋅⋅⋅O interaction between the acidic C−H α‐to the positively‐charged ammonium ion and the carbonyl O is proposed.^[26]^ Subsequent catalyst release generates the observed β‐lactone.

## Conclusion

To conclude, an approach for the catalytic generation of alkyl substituted C(1)‐ammonium enolates from (±)‐α‐silyl‐α‐alkyl substituted carboxylic acids using the isothiourea HyperBTM has been developed. The scope and limitations of this process has been evaluated in enantioselective [2+2]‐cycloaddition processes with perfluoroalkylketones (31 examples, up to 96 % yield and >99 : 1 er), as well as selective [2+2]‐cycloaddition with trifluoromethyl enones (4 examples, up to 75 % yield and >99 : 1 er). Mechanistic studies indicate this process proceeds through an initial kinetic resolution of an in situ generated (±)‐α‐silyl‐α‐alkyl substituted anhydride. Further applications of this methodology and its demonstration in further C(1)‐ammonium enolate transformations is currently under investigation.^[27]^

## Conflict of interest

The authors declare no conflict of interest.

1

## Supporting information

As a service to our authors and readers, this journal provides supporting information supplied by the authors. Such materials are peer reviewed and may be re‐organized for online delivery, but are not copy‐edited or typeset. Technical support issues arising from supporting information (other than missing files) should be addressed to the authors.

Supporting Information

Supporting Information

Supporting Information

## Data Availability

The data that support the findings of this study are openly available in PURE at https://doi.org/10.17630/941e041b‐bfd5‐4ccf‐ba56‐0c72af3eceee, reference number 1.
