# Supplementary material for: A Desilylative Approach to Alkyl Substituted C(1)‐Ammonium Enolates: Application in Enantioselective [2+2] Cycloadditions
Source: Angew Chem Int Ed Engl. 2022 Aug 8;61(38):e202208800. doi: 10.1002/anie.202208800 (PMC9543305; doi:10.1002/anie.202208800)

checkCIF/PLATON report

Structure factors have been supplied for datablock(s) 24, 37, 39

THIS REPORT IS FOR GUIDANCE ONLY. IF USED AS PART OF A REVIEW PROCEDURE FOR PUBLICATION, IT SHOULD NOT REPLACE THE EXPERTISE OF AN EXPERIENCED CRYSTALLOGRAPHIC REFEREE.

No syntax errors found.   CIF dictionary   Interpreting this report

Datablock: 24

```
Bond precision:   C-C = 0.0171 A          Wavelength=1.54184

Cell:               a=6.0454(3)      b=9.8442(5)      c=25.7165(11)
                    alpha=90         beta=90          gamma=90
Temperature:        125 K

                 Calculated                    Reported
Volume           1530.44(13)                   1530.44(13)
Space group      P 21 21 21                    P 21 21 21
Hall group       P 2ac 2ab                     P 2ac 2ab
Moiety formula   C13 H6 Br F9 O2               C13 H6 Br F9 O2
Sum formula      C13 H6 Br F9 O2               C13 H6 Br F9 O2
Mr               445.08                        445.08
Dx,g cm-3        1.932                         1.932
Z                4                             4
Mu (mm-1)        4.721                         4.725
F000             864.0                         864.0
F000'            865.18
h,k,lmax         7,12,32                       7,12,32
Nref             3238[ 1900]                   3092
Tmin,Tmax        0.893,0.954                   0.355,0.954
Tmin'            0.541

Correction method= # Reported T Limits: Tmin=0.355 Tmax=0.954
AbsCorr = MULTI-SCAN

Data completeness= 1.63/0.95       Theta(max)= 76.910

R(reflections)= 0.0628( 1993)                        wR2(reflections)=
                                                     0.1825( 3092)
S = 0.985                   Npar= 327
```

The following ALERTS were generated. Each ALERT has the format
test-name_ALERT_alert-type_alert-level.
Click on the hyperlinks for more details of the test.

Alert level B

```
PLAT090_ALERT_3_B Poor Data / Parameter Ratio (Zmax > 18) ........       5.65 Note
PLAT341_ALERT_3_B Low Bond Precision on  C-C Bonds ...............     0.0171 Ang.
```

Alert level C

```
PLAT031_ALERT_4_C Refined Extinction Parameter Within Range of ...      3.286 Sigma
PLAT213_ALERT_2_C Atom O2              has ADP max/min Ratio .....        3.7 prolat
PLAT213_ALERT_2_C Atom F7A             has ADP max/min Ratio .....        3.5 oblate
PLAT234_ALERT_4_C Large Hirshfeld Difference F5       --C13      .       0.21 Ang.
PLAT234_ALERT_4_C Large Hirshfeld Difference F4A      --C12      .       0.20 Ang.
PLAT234_ALERT_4_C Large Hirshfeld Difference F6A      --C13      .       0.25 Ang.
PLAT241_ALERT_2_C High   'MainMol' Ueq as Compared to Neighbors of         O1 Check
PLAT241_ALERT_2_C High   'MainMol' Ueq as Compared to Neighbors of         C3 Check
PLAT242_ALERT_2_C Low    'MainMol' Ueq as Compared to Neighbors of         C2 Check
PLAT431_ALERT_2_C Short Inter HL..A Contact  Br1      ..O2       .       3.15 Ang.
                                        -1+x,-1+y,z  =      1_445 Check
PLAT911_ALERT_3_C Missing FCF Refl Between Thmin & STh/L=    0.600          7 Report
```

Alert level G

```
PLAT002_ALERT_2_G Number of Distance or Angle Restraints on AtSite         25 Note
PLAT172_ALERT_4_G The CIF-Embedded .res File Contains DFIX Records          3 Report
PLAT301_ALERT_3_G Main Residue  Disorder .............(Resd  1  )        44% Note
PLAT398_ALERT_2_G Deviating  C-O-C   Angle From 120 for O1       .       93.3 Degree
PLAT811_ALERT_5_G No ADDSYM Analysis: Too Many Excluded Atoms ....          ! Info
PLAT860_ALERT_3_G Number of Least-Squares Restraints ............         25 Note
PLAT912_ALERT_4_G Missing # of FCF Reflections Above STh/L=  0.600         44 Note
PLAT941_ALERT_3_G Average HKL Measurement Multiplicity ..........         4.8 Low
PLAT978_ALERT_2_G Number C-C Bonds with Positive Residual Density.          1 Info
```

0 **ALERT level A** = Most likely a serious problem - resolve or explain
2 **ALERT level B** = A potentially serious problem, consider carefully
11 **ALERT level C** = Check. Ensure it is not caused by an omission or oversight
9 **ALERT level G** = General information/check it is not something unexpected

0 ALERT type 1 CIF construction/syntax error, inconsistent or missing data
9 ALERT type 2 Indicator that the structure model may be wrong or deficient
6 ALERT type 3 Indicator that the structure quality may be low
6 ALERT type 4 Improvement, methodology, query or suggestion
1 ALERT type 5 Informative message, check

Datablock: 37

Bond precision: C-C = 0.0054 A   Wavelength=1.54184

Cell:	a=6.20838(13)	b=7.51926(15)	c=40.6928(10)
	alpha=90	beta=90	gamma=90
Temperature:	173 K		

	Calculated	Reported
Volume	1899.64(7)	1899.64(7)
Space group	P 21 21 21	P 21 21 21
Hall group	P 2ac 2ab	P 2ac 2ab
Moiety formula	C19 H17 Br F5 N O2	C19 H17 Br F5 N O2
Sum formula	C19 H17 Br F5 N O2	C19 H17 Br F5 N O2
Mr	466.24	466.24
Dx,g cm-3	1.630	1.630
Z	4	4
Mu (mm-1)	3.535	3.538
F000	936.0	936.0
F000'	936.54	
h,k,lmax	7,9,49	7,8,48
Nref	3492[2075]	3472
Tmin,Tmax	0.880,0.932	0.616,0.932
Tmin'	0.678	

Correction method= # Reported T Limits: Tmin=0.616 Tmax=0.932
AbsCorr = MULTI-SCAN

Data completeness= 1.67/0.99 Theta(max)= 68.358

R(reflections)= 0.0293(3296) wR2(reflections)= 0.0801(3472)

S = 1.048 Npar= 262

The following ALERTS were generated. Each ALERT has the format
test-name_ALERT_alert-type_alert-level.
Click on the hyperlinks for more details of the test.

Alert level C

PLAT090_ALERT_3_C Poor Data / Parameter Ratio (Zmax > 18) .... 7.89 Note
PLAT911_ALERT_3_C Missing FCF Refl Between Thmin & STh/L= 0.600 2 Report

---

Alert level G

PLAT002_ALERT_2_G Number of Distance or Angle Restraints on AtSite 4 Note
PLAT172_ALERT_4_G The CIF-Embedded .res File Contains DFIX Records 1 Report
PLAT242_ALERT_2_G Low 'MainMol' Ueq as Compared to Neighbors of C5 Check
PLAT434_ALERT_2_G Short Inter HL..HL Contact F2 ..F5 . 2.77 Ang.
-1+x,y,z = 1_455 Check
PLAT791_ALERT_4_G Model has Chirality at C2 (Sohnke SpGr) S Verify

```
PLAT791_ALERT_4_G Model has Chirality at C3          (Sohnke SpGr)          S Verify
PLAT860_ALERT_3_G Number of Least-Squares Restraints .............          2 Note
PLAT912_ALERT_4_G Missing # of FCF Reflections Above STh/L=  0.600          2 Note
PLAT978_ALERT_2_G Number C-C Bonds with Positive Residual Density.          3 Info
```

---

0 **ALERT level A** = Most likely a serious problem - resolve or explain
0 **ALERT level B** = A potentially serious problem, consider carefully
2 **ALERT level C** = Check. Ensure it is not caused by an omission or oversight
9 **ALERT level G** = General information/check it is not something unexpected

0 ALERT type 1 CIF construction/syntax error, inconsistent or missing data
4 ALERT type 2 Indicator that the structure model may be wrong or deficient
3 ALERT type 3 Indicator that the structure quality may be low
4 ALERT type 4 Improvement, methodology, query or suggestion
0 ALERT type 5 Informative message, check

Datablock: 39

Bond precision: C-C = 0.0178 A   Wavelength=1.54184

Cell:	a=18.2247(11)	b=5.8495(4)	c=17.2067(12)
	alpha=90	beta=98.661(7)	gamma=90
Temperature:	173 K		

	Calculated	Reported
Volume	1813.4(2)	1813.4(2)
Space group	C 2	C 1 2 1
Hall group	C 2y	C 2y
Moiety formula	C15 H12 Br F9 O3	C15 H12 Br F9 O3
Sum formula	C15 H12 Br F9 O3	C15 H12 Br F9 O3
Mr	491.15	491.15
Dx,g cm-3	1.799	1.799
Z	4	4
Mu (mm-1)	4.092	4.096
F000	968.0	968.0
F000'	969.51	
h,k,lmax	21,7,20	21,7,20
Nref	3318[1833]	2946
Tmin,Tmax	0.502,0.921	0.413,0.921
Tmin'	0.403	

Correction method= # Reported T Limits: Tmin=0.413 Tmax=0.921
AbsCorr = MULTI-SCAN

Data completeness= 1.61/0.89   Theta(max)= 68.288

R(reflections)= 0.0963(2616)   wR2(reflections)= 0.2692(2946)

S = 1.209   Npar= 258

The following ALERTS were generated. Each ALERT has the format
test-name_ALERT_alert-type_alert-level.
Click on the hyperlinks for more details of the test.

Alert level B

```
PLAT341_ALERT_3_B Low Bond Precision on  C-C Bonds ...............    0.01779 Ang.
```

Alert level C

```
PLAT084_ALERT_3_C High wR2 Value (i.e. > 0.25) ...................       0.27 Report
PLAT090_ALERT_3_C Poor Data / Parameter Ratio (Zmax > 18) ........       7.03 Note
PLAT911_ALERT_3_C Missing FCF Refl Between Thmin & STh/L=    0.600          7 Report
PLAT915_ALERT_3_C No Flack x Check Done: Low Friedel Pair Coverage         76 %
PLAT934_ALERT_3_C Number of (Iobs-Icalc)/Sigma(W) > 10 Outliers ..          1 Check
PLAT971_ALERT_2_C Check Calcd Resid. Dens.  0.95Ang From Br1             1.75 eA-3
PLAT975_ALERT_2_C Check Calcd Resid. Dens.  0.96Ang From O3      .       0.62 eA-3
PLAT975_ALERT_2_C Check Calcd Resid. Dens.  0.99Ang From O3      .       0.43 eA-3
```

Alert level G

```
PLAT002_ALERT_2_G Number of Distance or Angle Restraints on AtSite          2 Note
PLAT066_ALERT_1_G Predicted and Reported Tmin&Tmax Range Identical          ? Check
PLAT072_ALERT_2_G SHELXL First  Parameter in WGHT  Unusually Large       0.20 Report
PLAT172_ALERT_4_G The CIF-Embedded .res File Contains DFIX Records          1 Report
PLAT242_ALERT_2_G Low    'MainMol' Ueq as Compared to Neighbors of         C7 Check
PLAT434_ALERT_2_G Short Inter HL..HL Contact F1        ..F4        .     2.79 Ang.
                                                   x,1+y,z  =      1_565 Check
PLAT434_ALERT_2_G Short Inter HL..HL Contact F6        ..F6        .     2.80 Ang.
                                                 1-x,y,1-z  =      2_656 Check
PLAT791_ALERT_4_G Model has Chirality at C2           (Sohnke SpGr)         S Verify
PLAT791_ALERT_4_G Model has Chirality at C3           (Sohnke SpGr)         S Verify
PLAT860_ALERT_3_G Number of Least-Squares Restraints .............          2 Note
PLAT912_ALERT_4_G Missing # of FCF Reflections Above STh/L=  0.600         13 Note
PLAT941_ALERT_3_G Average HKL Measurement Multiplicity ...........        4.9 Low
PLAT978_ALERT_2_G Number C-C Bonds with Positive Residual Density.          0 Info
```

---

0 **ALERT level A** = Most likely a serious problem - resolve or explain
1 **ALERT level B** = A potentially serious problem, consider carefully
8 **ALERT level C** = Check. Ensure it is not caused by an omission or oversight
13 **ALERT level G** = General information/check it is not something unexpected

1 ALERT type 1 CIF construction/syntax error, inconsistent or missing data
9 ALERT type 2 Indicator that the structure model may be wrong or deficient
8 ALERT type 3 Indicator that the structure quality may be low
4 ALERT type 4 Improvement, methodology, query or suggestion
0 ALERT type 5 Informative message, check

It is advisable to attempt to resolve as many as possible of the alerts in all categories. Often the minor alerts point to easily fixed oversights, errors and omissions in your CIF or refinement strategy, so attention to these fine details can be worthwhile. In order to resolve some of the more serious problems it may be necessary to carry out additional measurements or structure refinements. However, the purpose of your study may justify the reported deviations and the more serious of these should normally be commented upon in the discussion or experimental section of a paper or in the "special_details" fields of the CIF. checkCIF was carefully designed to identify outliers and unusual parameters, but every test has its limitations and alerts that are not important in a particular case may appear. Conversely, the absence of alerts does not guarantee there are no aspects of the results needing attention. It is up to the individual to critically assess their own results and, if necessary, seek expert advice.

Publication of your CIF in IUCr journals

A basic structural check has been run on your CIF. These basic checks will be run on all CIFs submitted for publication in IUCr journals (*Acta Crystallographica*, *Journal of Applied Crystallography*, *Journal of Synchrotron Radiation*); however, if you intend to submit to *Acta Crystallographica Section C* or *E* or *IUCrData*, you should make sure that full publication checks are run on the final version of your CIF prior to submission.

Publication of your CIF in other journals

Please refer to the *Notes for Authors* of the relevant journal for any special instructions relating to CIF submission.

PLATON version of 18/05/2022; check.def file version of 17/05/2022

Datablock 24 - ellipsoid plot

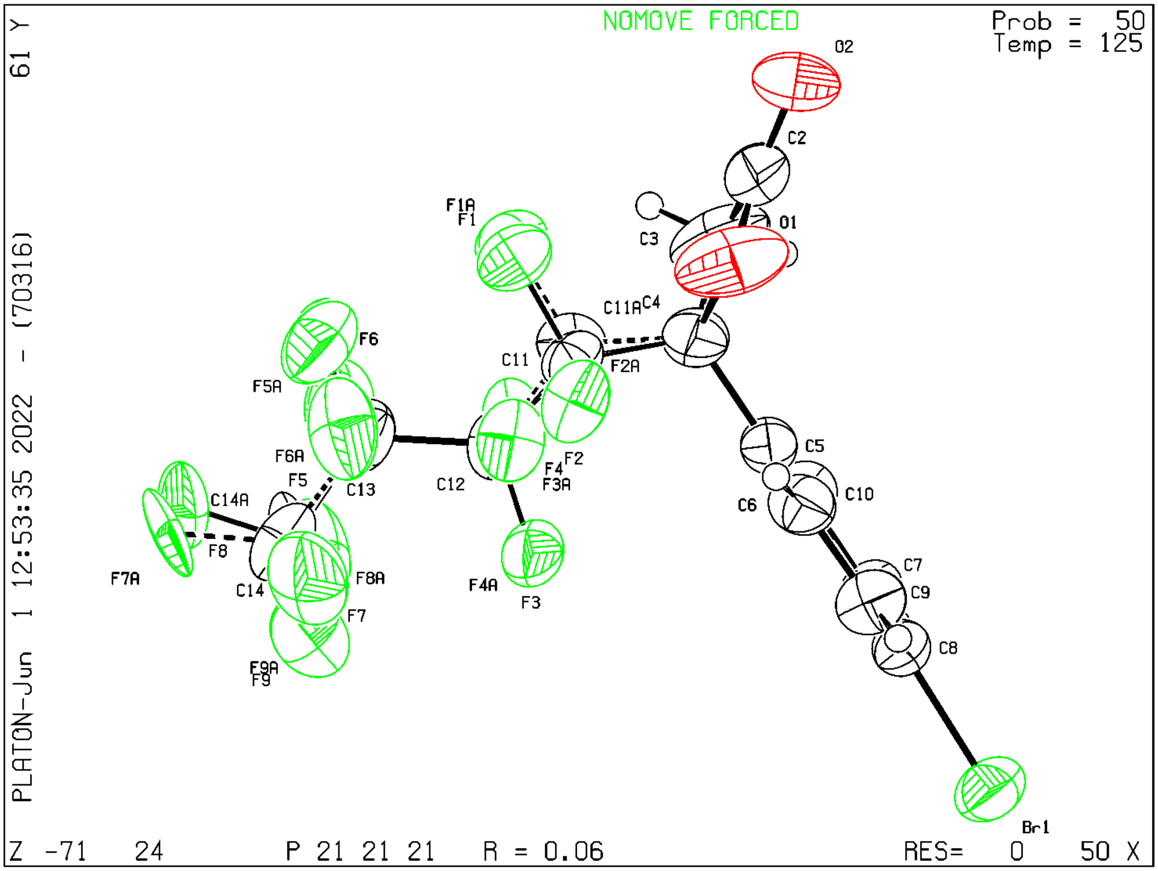

Datablock 37 - ellipsoid plot

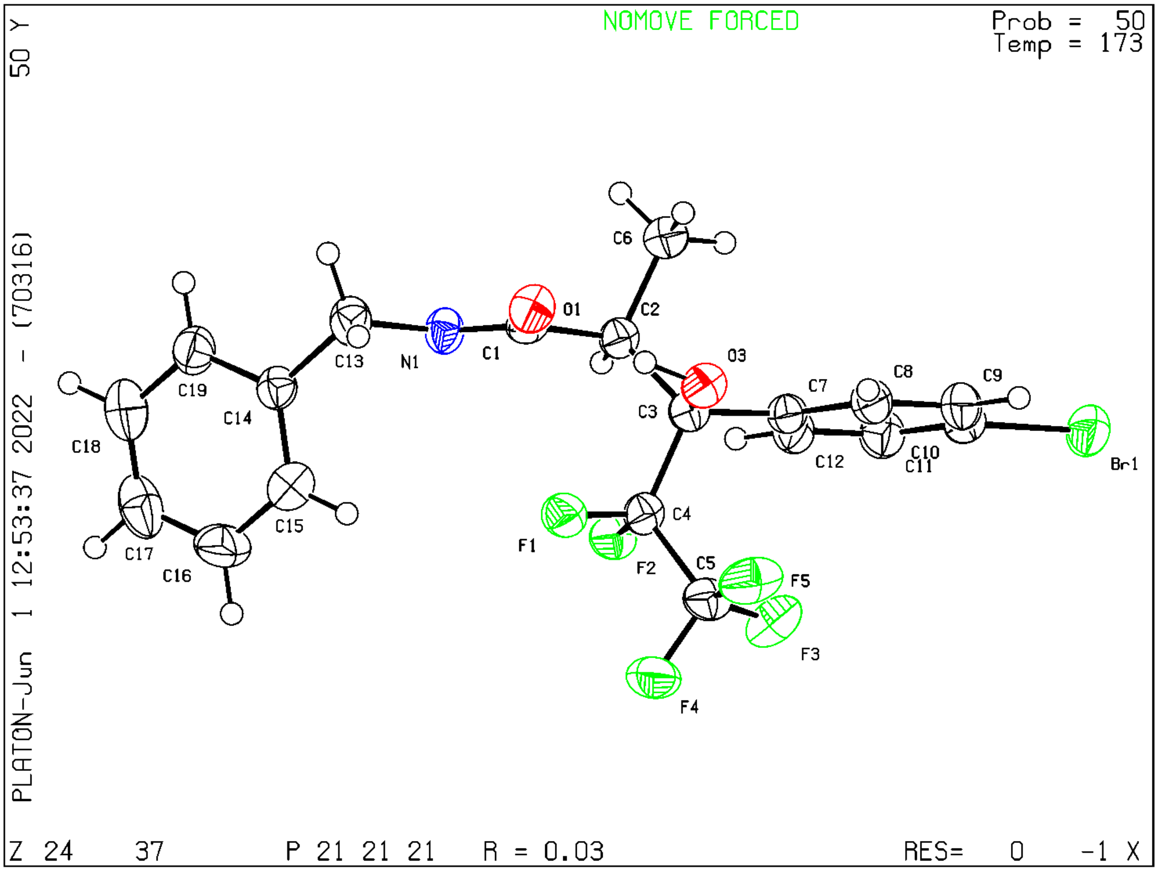

Datablock 39 - ellipsoid plot

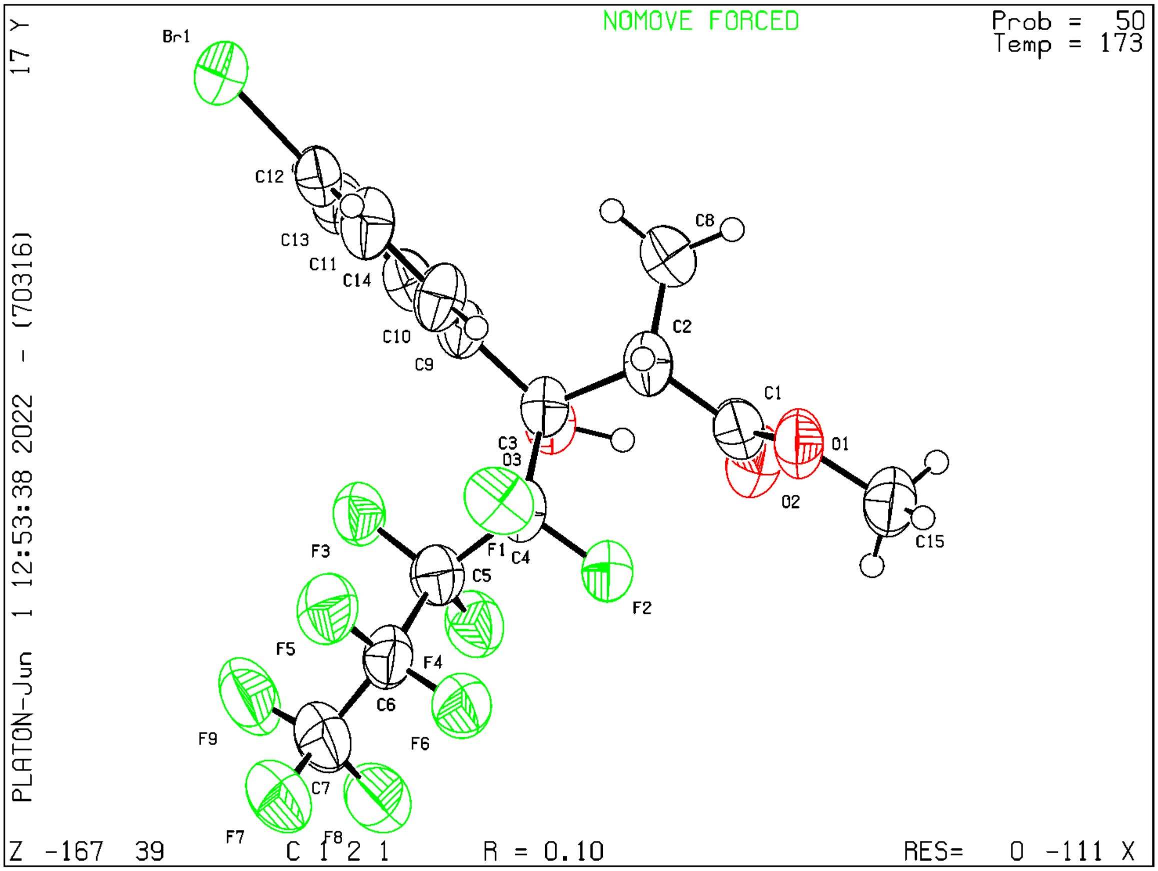

Supplement: Supplementary file 2 — Supporting Information [file ANIE-61-0-s001.pdf]
