# Supplementary material for: A Desilylative Approach to Alkyl Substituted C(1)‐Ammonium Enolates: Application in Enantioselective [2+2] Cycloadditions
Source: Angew Chem Int Ed Engl. 2022 Aug 8;61(38):e202208800. doi: 10.1002/anie.202208800 (PMC9543305; doi:10.1002/anie.202208800)
Supplement: Supplementary file 3 — Supporting Information [file ANIE-61-0-s002.pdf]

Angewandte Chemie
Eine Zeitschrift der Gesellschaft Deutscher Chemiker

Supporting Information

# A Desilylative Approach to Alkyl Substituted C(1)-Ammonium Enolates: Application in Enantioselective [2+2] Cycloadditions

*Y. Wang, C. M. Young, H. Liu, W. C. Hartley, M. Wienhold, D. B. Cordes, A. M. Z. Slawin, A. D. Smith**

# Supporting Information


| | | |
|---|---|---|
| 1. | General information | 2 |
| 2. | General Procedures | 4 |
| 3. | Synthesis of Starting Materials | 7 |
| 4. | Catalysis Products | 25 |
| 5. | Product Derivatizations | 56 |
| 6. | Mechanistic Investigations | 60 |
| 7. | Determination of Product Configuration by X-ray Crystallography | 88 |
| 8. | References | 92 |
| Appendix I. 1H, 19F and 13C{1H} NMR Spectra | | 95 |
| Appendix II. HPLC Traces | | 245 |

## 1. General information

Reactions involving moisture sensitive reagents were carried out in flame-dried glassware under a nitrogen atmosphere using standard vacuum line techniques and using anhydrous solvents. HyperBTM **4** and benzotetramisole (BTM) **5** were synthesized in house,[1] Tetramisole•HCl **6** was obtained from Sigma-Aldrich. Anhydrous solvents ($CH_2Cl_2$, PhMe) was obtained after passing through an alumina column (Mbraun SPS-800). Anhydrous MTBE and MeCN was obtained by treatment with activated 4Å molecular sieves. Petrol is defined as petroleum ether 40–60 °C. All other solvents and commercial reagents were used as supplied without further purification unless otherwise stated. EtOAc, $Et_2O$, $CH_2Cl_2$ and Petrol for purification purposes were used as obtained from suppliers without further purification. Room temperature (r.t.) refers to 20–25 °C. Temperatures of 0 °C and −78 °C were obtained using ice/water and $CO_2$(s)/acetone baths respectively. Reactions involving heating were performed using a DrySyn block and a contact thermocouple.

*In vacuo* refers to the use either a Büchi Rotavapor R-200 with a Büchi V-491 heating bath and Büchi V-800 vacuum controller; a Büchi Rotavapor R-210 with a Büchi V-491 heating bath and Büchi V-850 vacuum controller; a Heidolph Laborota 4001 with vacuum controller; an IKA RV10 rotary evaporator with an IKA HB10 heating bath and ILMVAC vacuum controller; or an IKA RV10 rotary evaporator with an IKA HB10 heating bath and Vacuubrand CVC3000 vacuum controller. Rotary evaporator condensers are fitted to Julabo FL601 Recirculating Coolers filled with ethylene glycol set to −6 °C.

Analytical thin layer chromatography was performed on pre-coated aluminium plates (Kieselgel 60 F254 silica). TLC visualisation was carried out with ultraviolet light (254 nm), followed by staining with a 1% aqueous KMnO4 solution. Automated chromatography was performed on a Biotage Isolera Four running Biotage OS578 with a UV/Vis detector using the method stated and cartridges filled with Kieselgel 60 silica.

Melting points were recorded on an Electrothermal 9100 melting point apparatus and are uncorrected.

Optical rotations were measured on a Perkin Elmer Precisly/Model-341 polarimeter operating at the sodium D line with a 100 mm path cell at 20 °C.

HPLC analyses were obtained using either a Shimadzu HPLC consisting of a DGU-20A5 degassing unit, LC-20AT liquid chromatography pump, SIL-20AHT autosampler, CMB-20A communications bus module, SPD-M20A diode array detector and a CTO-20A column oven; or a Shimadzu HPLC consisting of a DGU-20A5R degassing unit, LC-20AD liquid chromatography pump, SIL-20AHT autosampler, SPD-20A UV/Vis detector and a CTO-20A column oven. Separation was achieved using DAICEL CHIRALPAK AS-H and IB columns using the method stated. HPLC traces of enantiomerically enriched compounds were compared with authentic racemic spectra.

$^{1}$H, $^{13}$C{1H} and $^{19}$F{$^{1}$H} nuclear magnetic resonance (NMR) spectra were acquired on either a Bruker Avance II 400 ($^{1}$H 400 MHz; $^{13}$C{$^{1}$H} 101 MHz, $^{19}$F{$^{1}$H} 376 MHz) or a Bruker Avance II 500 ($^{1}$H 500 MHz; $^{13}$C{$^{1}$H} 126 MHz, $^{19}$F{$^{1}$H} 471 MHz) spectrometer at ambient temperature in the deuterated solvent stated. All chemical shifts are quoted in parts per million (ppm) and referenced to the residual solvent peak. All coupling constants, *J*, are quoted in Hz. Multiplicities are indicated by s (singlet), d (doublet), t (triplet), q (quartet), dd (doublet of doublets), dt (doublet of triplets), dq (doublet of quartets), tt (triplet of triplets), ddd (doublet of doublet of doublets) and m (multiplet). The abbreviation Ar is used to denote aromatic, Ph to denote phenyl, Bn to denote benzyl, br to denote broad and app to denote apparent. NMR peak assignments were confirmed using 2D $^{1}$H correlated spectroscopy (COSY), $^{1}$H−$^{13}$C heteronuclear single quantum coherence (HSQC) and 2D $^{1}$H−$^{13}$C heteronuclear multiple-bond correlation spectroscopy (HMBC) where necessary.

Infrared spectra were recorded on a Shimadzu IRAffinity-1 Fourier transform IR spectrophotometer fitted with a Specac Quest ATR accessory (diamond puck). Spectra were recorded of either thin films or solids, with characteristic absorption wavenumbers ($ν_{max}$) reported in $cm^{-1}$.

Mass spectrometry (**HRMS**) data were acquired by either electrospray ionisation (ESI), electron impact (EI), atmospheric pressure chemical ionization (APCI) or nanospray ionization (NSI) either at either the University of St Andrews Mass Spectrometry Facility or at the EPSRC UK National Mass Spectrometry Facility at Swansea University.

## 2. General Procedures

### *2.1 General Procedure A: Synthesis of α-Silyl Acids*

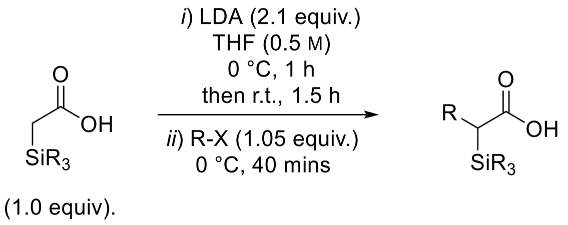


According to a procedure reported by Rogers *et al.*[2], diisopropylamine (9.6 mmol, 2.1 equiv.) was dissolved in THF (10 mL) under an $N_2$-atmosphere. The solution was cooled to −78 °C and *n*-BuLi (9.6 mmol, 2.1 equiv.) was added. The mixture was warmed to r.t. for 15 min before being cooled to −78 °C again. 2-(Trimethylsilyl) acetic acid (4.5 mmol, 1.0 equiv.) was added and the mixture was stirred at 0 °C for 1 h, followed by 1.5 h at r.t.. Subsequently the specified halide (4.7 mmol, 1.05 equiv.) was added at 0 °C and the mixture was stirred additional 30 min at 0 °C. Then the reaction was quenched by the addition of HCl (1 M) and the pH adjusted to 2. The aqueous layer was extracted with $Et_2O$ (3 × 15 mL). The combined organic layers were dried over $MgSO_4$, filtered and the solvent was removed under reduced pressure. The crude residue was triturated from pentane to give the desired product.

### *2.2 General Procedure B: Synthesis of Perfluoroalkylketones*

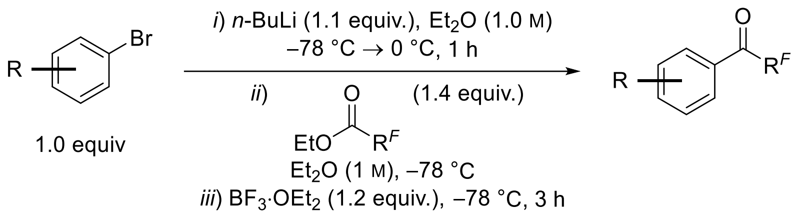


According to a procedure reported by Smith *et al*,[3] *n*-Butyl lithium (2.5 M in hexane, 1.1 equiv.) was added dropwise to a solution of the requisite bromoarene (1 equiv.) in anhydrous $Et_2O$ (1 M) at −78 °C under an $N_2$ atmosphere. The reaction mixture was then allowed to warm to 0 °C and allowed to stir for 1 h. This solution was then transferred by cannula into a flask containing the required polyfluoroester (1.4 equiv.) in $Et_2O$ (1 M) at −78 °C under an inert atmosphere. $BF_3{\cdot}OEt_2$ (1.2 equiv.) was then added and the mixture was allowed to stir at −78 °C for 3 h. Saturated aqueous

$NH_4Cl$ was added and the biphasic mixture was allowed to warm to r.t.. The layers were separated, the aqueous layer was extracted with $Et_2O$ (3 × 20mL), and the combined organic layers were washed with brine, dried over $MgSO_4$, filtered and concentrated under reduced pressure. The residue was purified as described to afford the desired ketone.

*2.3 General Procedure C: Synthesis of Alternative α-Silyl Acids*

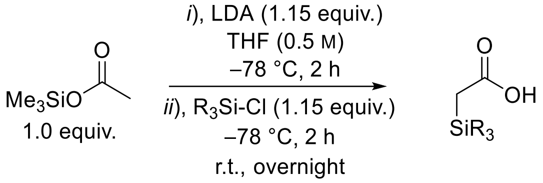


According to a procedure reported by Becker *et al*,[4] to an oven dried round-bottomed flask (250 mL) equipped with a magnetic stirring bar was added diisopropylamine (24.0 mmol, 1.15 equiv.) and anhydrous THF (40 mL). The mixture was cooled to −78 °C, then *n*-BuLi 1.6 M (24.0 mmol, 1.15 equiv.) was added dropwise. The mixture was warmed to r.t. for 15 minutes and cooled again to −78 °C. Trimethylsilyl acetate ($CH_3CO_2SiMe_3$) (21.0 mmol, 1.0 equiv.) was added dropwise to the cooled solution of LDA over 15 minutes and the reaction mixture was stirred for 2 hours at −78 °C. Then chlorosilane (24.0 mmol, 1.15 equiv.) in anhydrous THF (5 mL) was added dropwise to the solution over 10 minutes. The reaction mixture was then stirred at −78 °C for 2 additional hours and allowed to reach room temperature overnight. A solution of saturated aqueous NaCl solution (30 ml) was added, and the pH was adjusted to 3 using 1 M aqueous HCl. The aqueous layer was extracted with $Et_2O$ (3 × 30 mL) and the combined organic extracts were washed with water, dried over $MgSO_4$, filtered and concentrated under reduced pressure. The residual crude product was dissolved in THF (30 mL) and saturated aqueous $NH_4Cl$ solution (20 mL) was added. The reaction mixture was then stirred at room temperature for one hour. Afterwards, the aqueous layer was extracted with $Et_2O$ (3 × 30 mL) and the combined organic extracts were washed with water (30 mL), and dried over $MgSO_4$, filtered and concentrated under reduced pressure. The crude residue was crystallized from hexane to give the desired product.

*2.4 General Procedure D: Isothiourea-Catalysed Enantioselective [2+2] Cycloaddition to β-Lactones*

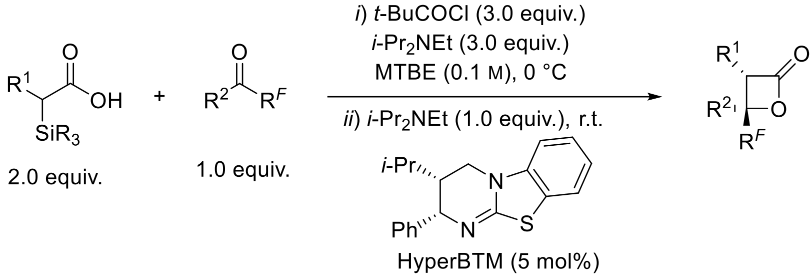


In a flame dried Schlenk tube under an $N_2$ atmosphere, *N,N*-diisopropylethylamine (3.0 equiv.) and pivaloyl chloride (3.0 equiv.) were added sequentially to a solution of appropriate acid (2.0 equiv.) in anhydrous MTBE (0.1 M) at 0 °C. The mixture was allowed to stir for 15 min at 0 °C, followed by the sequential addition of the specified ketone (1.0 equiv.), (2*S*,3*R*)-HyperBTM (5 mol%) and *N,N*-diisopropylethylamine (1.0 equiv.). The mixture was allowed to stir for the specified time at r.t.. The solvent was then removed under reduced pressure, and the crude residue purified by Biotage automated column chromatography in the stated solvent system to give the desired product.

## 3. Synthesis of Starting Materials

*3.1 α-Silyl Acids*

**2-(trimethylsilyl)acetic acid (1)**

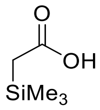

**1**

Following a procedure reported by Bertounesque *et al.*[5], diisopropylamine (36.0 mmol, 2.1 equiv.) was dissolved in THF (35 mL) under an $N_2$-atmosphere. The solution was cooled to −78 °C and *n*-BuLi (36.0 mmol, 2.1 equiv.) was added. The mixture was warmed to r.t. for 25 min before it cooled to −78 °C again. At −78 °C, acetic acid (17 mmol, 1.0 equiv.) was added slowly, and the mixture was then heated to 68 °C for 24 h. The reaction was cooled to −78 °C again and trimethylsilyl chloride (41.0 mmol, 2.4 equiv.) was added. Then the mixture was warmed to r.t. overnight. A saturated aqueous NaCl solution (20 mL) was added, and the mixture was acidified to pH=2 by the addition of HCl (1 M). The aqueous layer was extracted with $Et_2O$ (3 × 20 mL) and the organic layer was washed with $H_2O$ (2 × 10 mL). The solvent was removed from the organic layer under reduced pressure and the residue was dissolved in THF (10 mL) before a saturated aqueous $NH_4Cl$-solution (10 mL) was added. This mixture was stirred for 1 h at r.t.. Subsequently the aqueous layer was extracted with $Et_2O$ (3 × 20 mL), the organic layer was dried over $MgSO_4$, filtered and the solvent was removed under reduced pressure. The crude residue was triturated with pentane at −21 °C for 24 hours to give the desired product as white solid (1.5 g, 63%) with spectroscopic data in accordance with the literature.[5]

**$^1$H NMR** (400 MHz, $CDCl_3$) $\delta_H$: 0.18 (9H, s, Si(C*H*$_3$)$_3$), 1.96 (2H, s, COC*H*$_2$).

**$^{13}$C{$^1$H} NMR** (126 MHz, $CDCl_3$) $\delta_C$ 1.5 (Si(*C*H$_3$)$_3$), 27.2 (CO*C*H$_2$), 179.9 (*C*O).

**2-(Methyldiphenylsilyl)acetic acid (S2)**

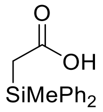

**S2**

Following General Procedure C, diisopropylamine (3.4 mL, 24.0 mmol) in THF (30 mL), *n*-BuLi (9.6 mL, 24.0 mmol), trimethylsilyl acetate (3.2 mL, 21.0 mmol), diphenylmethylchlorosilane (5.0 mL, 24.0 mmol) gave, after trituration, 2-(methyldiphenylsilyl)acetic acid as a withe solid (4.3 g, 80%) with spectroscopic data in accordance with the literature.[4]

**mp** 103 – 105 °C.

**$^1$H NMR** (400 MHz, $CDCl_3$) $\delta_H$: 0.74 (3H, s, $SiC\mathit{H}_3Ph_2$), 2.46 (2H, s, $COC\mathit{H}_2$), 7.37 – 7.45 (6H, m, Ph*H*), 7.56 – 7.59 (4H, m, Ph*H*).

**2-(Dimethyl(phenyl)silyl)acetic acid (S3)**

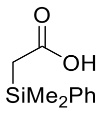

**S3**

Following General Procedure C, diisopropylamine (3.4 mL, 24.0 mmol) in THF (30 mL), *n*-BuLi (9.6 mL, 24.0 mmol), trimethylsilyl acetate (3.2 mL, 21.0 mmol), phenyldimethylchlorosilane (4.0 mL, 24.0 mmol) gave, after trituration, 2-(methyldiphenylsilyl)acetic acid as a white solid (3.5 g, 85%) with spectroscopic data in accordance with the literature.[4]

**mp** 88 – 90 °C.

**$^1$H NMR** (400 MHz, $CDCl_3$) $\delta_H$: 0.47 (6H, s, $Si(C\mathit{H}_3)_2Ph$), 2.17 (2H, s, $COC\mathit{H}_2$), 7.38 – 7.43 (3H, m, Ph*H*), 7.56 – 7.59 (2H, m, Ph*H*).

**2-(Trimethylsilyl)propanoic acid (S4)**

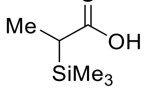

**S4**

Following General Procedure A, diisopropylamine (1.35 mL, 9.6 mmol, 2.1 equiv.), *n*-BuLi (3.8 mL, 9.6 mmol, 2.1 equiv.), 2-(trimethylsilyl) acetic acid **1** (600 mg, 4.5 mmol, 1.0 equiv.), iodomethane (292 µL, 4.7 mmol, 1.05 equiv.) in THF (10 mL), gave, after trituration, 2-(trimethylsilyl)propanoic acid as a white solid (540 mg, 82%) with spectroscopic data in accordance with the literature.[2]

**$^1$H NMR** (400 MHz, $CDCl_3$) $\delta_H$: 0.14 (9H, s, Si(C*H*$_3$)$_3$), 1.21 (3H, d, *J* 7.0, CHC*H$_3$*), 2.12 (1H, q, *J* 7.0, COC*H*).

**$^{13}$C{$^1$H} NMR** (126 MHz, $CDCl_3$) $\delta_C$ −3.0 (Si(*C*H$_3$)$_3$), 10.7 (CH*C*H$_3$), 30.5 (CO*C*H), 183.2 (*C*O).

***tert*-Butyl 2-methyl-3-oxobutanoate (S5)**

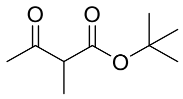

**S5**

Following a procedure reported by Iwasa *et al*,[6] to a stirred suspension of NaH (840 mg, 21.0 mmol, 1.05 equiv.) in THF (20 mL) at 0 ºC was added *tert*-butyl acetoacetate (3.32 mL, 20.0 mmol, 1.0 equiv.) and the mixture was stirred for 5 min. MeI (1.3 mL, 21.0 mmol, 1.05 equiv.) was added slowly at 0 ºC and the solution was stirred for 1 h warming from 0 ºC to r.t.. The reaction mixture was quenched with saturated aqueous $NH_4Cl$-solution (10 mL). and the organic layer was separated and the aqueous layer was extracted with $Et_2O$. The organic layer was dried over $MgSO_4$, filtered and the solvent was evaporated. Purification of the crude material by column chromatography gave *tert*-butyl 2-methyl-3-oxobutanoate as yellow oil (1.35 g, 36 %) with spectroscopic data in accordance with the literature.[6]

**IR** $\nu_{max}$ (film) 1711 (C=O).

**$^1$H NMR** (400 MHz, $CDCl_3$) δ 1.28 (3H, d, *J* 7.2, CHC*H*$_3$), 1.46 (9H, s, C(C*H*$_3$)$_3$), 2.22 (3H, s, COC*H*$_3$), 3.40 (1H, q, *J* 7.1, C*H*CH$_3$).

***tert*-Butyl 2-diazopropanoate (S6)**

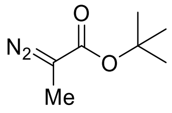


**S6**

Following a procedure reported by Iwasa *et al*,[6] to a stirred suspension of *tert*-butyl 2-methyl-3-oxobutanoate **S5** (4.2 g, 21.8 mmol, 1 equiv.) in $CH_3CN$ (20 mL) was added *p*-ABSA (7.7 g, 32.9 mmol, 1.5 equiv.) under a nitrogen atmosphere. The mixture was cooled to 0 ºC, and DBU (0.71 mL, 4.7 mmol, 1.5 equiv.) was added. After stirring for 30 min at 0 ºC, then r.t. for 16 h, the reaction

mixture was quenched with $H_2O$ (10 mL) and extracted with $Et_2O$ (3 × 10 mL). The organic phase was dried over $Mg_2SO_4$, filtered and evaporated gave crude product. Purification of crude material by column chromatography with Hexane/EtOAc gave *tert*-butyl 2-diazopropanoate as yellow oil (1.3 g, 38%) with spectroscopic data in accordance with the literature.[6]

**IR** $v_{max}$ (film) 2075 (C=N), 1682 (C=O).

**$^1$H NMR** (400 MHz, $CDCl_3$) δ 1.49 (1H, s, C$H_3$), 1.93 (3H, s, C(C$H_3$)$_3$).

### ***tert*-Butyl (*R*)-2-(dimethyl(phenyl)silyl)propanoate (S7)**

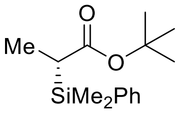

**S7**

Following a procedure reported by Iwasa *et al*,[6] *tert*-butyl 2-diazopropanoate **S6** (156.0 mg, 1.0 mmol) was added to the solution of $CH_2Cl_2$ (5 mL) and dimethylphenylsilane (0.3 mL, 2.0 mmol) and Ru(II)-(*S*)-Pheox (6.3 mg, 0.01mmol) at 0 ºC, after 10 min, the resulting mixture was purified by column chromatography with Hexane/EtOAc as an eluent gave *tert*-butyl (*R*)-2-(dimethyl(phenyl)silyl) propanoate as colourless oil (207 mg, 78% yield) with spectroscopic data in accordance with the literature.[6]

$[\alpha]_D^{20}$ +34.8 (*c* 0.2, $CHCl_3$).

**Chiral HPLC analysis**, Chiralpak IC (99.8:0.2 hexane:IPA, flow rate 1.0 mL min$^{-1}$, 211nm, 30 °C), $t_R$(major): 6.3 min, $t_R$(minor): 7.0 min, 90:10 er.

**IR** $v_{max}$ (film) 1709 (C=O).

**$^1$H NMR** (400 MHz, $CDCl_3$) $\delta_H$: 0.40 (6H, d, *J* 7.1, Si(C$H_3$)$_2$Ph), 1.14 (3H, d, *J* 7.2, CHC$H_3$), 1.36 (9H, s, C(C$H_3$)$_3$), 2.21 (1H, q, *J* 7.1, COC*H*), 7.35 – 7.42 (3H, m, Ph*H*), 7.53 – 7.57 (2H, m, Ph*H*).

### **(*R*)-2-(Dimethyl(phenyl)silyl)propanoic acid (S8)**

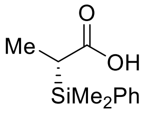

**(*R*)-S8**

*tert*-Butyl (*R*)-2-(dimethyl(phenyl)silyl) propanoate **S7** (264 mg, 1 mmol) was dissolved in $CH_2Cl_2$ (4 mL) at r.t. and treated with $CF_3COOH$ (2 mL). After 1 h, the solvent was removed under reduced pressure and the residue was dissolved in $CH_2Cl_2$ (5 mL) and saturated aqueous $NaHCO_3$ (5 mL), the mixture was hydrolysed with 1 M HCl to pH=3. The aqueous layer was extracted with $CH_2Cl_2$ (3 × 10 mL) and the combined organic layer, dried over anhydrous $MgSO_4$, filtered and concentrated to give (*R*)-2-(dimethyl(phenyl)silyl)propanoic acid as colourless oil (166 mg, 80%).

$[\alpha]_D^{20}$ +31.3 (*c* 0.2, $CHCl_3$).

**Chiral HPLC analysis**, Chiralcel OJ-H (97:3 hexane:IPA, flow rate 0.6 mL $min^{-1}$, 211nm, 30 °C), $t_R$(minor): 18.6 min, $t_R$(major): 22.7 min, 89:11 er.

**IR** $\nu_{max}$ (film) 1678 (C=O).

**$^1$H NMR** (400 MHz, $CDCl_3$) $\delta_H$: 0.45 (6H, d, *J* 2.6, Si(C*H*$_3$)$_2$Ph), 1.17 (3H, d, *J* 7.0, CHC*H*$_3$), 2.34 (1H, q, *J* 7.0, COC*H*), 7.37 – 7.43 (3H, m, Ph*H*), 7.54 – 7.56 (2H, m, Ph*H*).

**$^{13}$C{$^1$H} NMR** (101 MHz, $CDCl_3$) $\delta_C$ −5.2 (Si(*C*H$_3$CH$_3$), −3.9 (Si(CH$_3$*C*H$_3$), 11.1 (CH*C*H$_3$), 30.2 (CO*C*H), 127.9 (Ph*C*(2,6)H), 129.7 (Ph*C*(4)H), 133.9 (Ph*C*(3,5)H), 135.7 (Ph*C*(1)), 182.5 (*C*O).

**HRMS** (ESI$^+$) $C_{11}H_{16}O_2NaSi$ [M+Na]$^+$ found 231.0805, requires 231.0812 (−2.7 ppm).

**2-(Dimethyl(phenyl)silyl)propanoic acid (S8)**

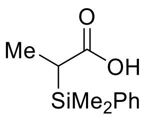


**(±)-S8**

Following General Procedure A, diisopropylamine (1.8 mL, 12.8 mmol, 2.1 equiv.), *n*-BuLi (5.1 mL, 12.8 mmol, 2.1 equiv.), 2-(methyldiphenylsilyl)acetic acid **S3** (1.07 g, 6.1 mmol, 1.0 equiv.), iodomethane (370 µL, 6.3 mmol, 1.05 equiv.) in THF (15 mL) gave. After trituration, 2-(dimethyl(phenyl)silyl)propanoic acid as a white solid (570 mg, 45%).

**mp** 38 – 40 °C.

**IR** $\nu_{max}$ (film) 1678 (C=O), 1115 (C−O).

**$^1$H NMR** (400 MHz, $CDCl_3$) $\delta_H$: 0.45 (6H, d, *J* 2.6, Si(C*H*$_3$)$_2$Ph), 1.17 (3H, d, *J* 7.0, CHC*H*$_3$), 2.34 (1H, q, *J* 7.0, COC*H*), 7.37 – 7.43 (3H, m, Ph*H*), 7.54 – 7.56 (2H, m, Ph*H*).

**$^{13}$C{$^1$H} NMR** (101 MHz, $CDCl_3$) $\delta_C$ −5.2 (Si(*C*H$_3$CH$_3$), −3.9 (Si(CH$_3$*C*H$_3$), 11.1 (CH*C*H$_3$), 30.2

(CO*C*H), 127.9 (Ph*C*(2,6)H), 129.7 (Ph*C*(4)H), 133.9 (Ph*C*(3,5)H), 135.7 (Ph*C*(1)), 182.5 (*C*O).

**HRMS** (ESI$^+$) $C_{11}H_{16}O_2NaSi$ [M+Na]$^+$ found 231.0805, requires 231.0812 (−2.7 ppm).

**(*R*)-(*R*)-2-(Dimethyl(phenyl)silyl)propanoic pivalic anhydride (46)**

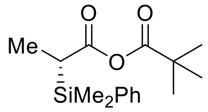

**46**

2-(Dimethyl(phenyl)silyl)propanoic acid **S8** (42 mg, 0.2 mmol, 1 equiv.) was added in MTBE (2 mL), pivaloyl chloride (50 μL, 0.4 mmol, 2 equiv.) and *N,N*-diisopropylethylamine (70 μL, 0.4 mmol, 2 equiv) were added. After 10 min at 0 ºC, filtered and diethyl ether washed, remove the solvent to give the product as bright yellow oil (47 mg, 80%).

$[\alpha]_D^{20}$ +0.2 (*c* 0.4, CHCl₃).

**Chiral HPLC analysis**, Chiralcel OD-H (99.9:0.1 hexane:IPA, flow rate 1.0 mL min$^{-1}$, 211 nm, 30 °C), $t_R$(major): 8.4 min, $t_R$(minor): 11.4 min, 89:11 er.

**IR** $v_{max}$ (film) 1796 (C=O), 1051 (C−O).

**$^1$H NMR** (500 MHz, $CDCl_3$) $\delta_H$: 0.46 (6H, d, *J* 8.3, Si(C*H*$_3$)$_2$Ph), 1.13 (9H, s, C(C*H*$_3$)$_3$), 1.23 (3H, d, *J* 6.8, CHC*H*$_3$), 2.44 (1H, q, *J* 7.0, COC*H*), 7.38 – 7.43 (3H, m, Ph*H*), 7.53 – 7.55 (2H, m, Ph*H*).

**$^{13}$C{$^1$H} NMR** (126 MHz, $CDCl_3$) $\delta_C$ −4.7 (Si(*C*H$_3$CH$_3$), −4.3 (Si(CH$_3$*C*H$_3$), 11.2 (CH*C*H$_3$), 26.4 (C(*C*H$_3$)$_3$), 32.4 (CO*C*H), 39.6 (*C*(CH$_3$)$_3$), 128.0 (Ph*C*(2,6)), 129.8 (Ph*C*(4)), 133.9 (Ph*C*(3,5)), 135.3 (Ph*C*(1)), 172.3 (*C*OCH), 174.2 (*C*OC).

**HRMS** (ESI$^+$) $C_{16}H_{24}O_3NaSi$ [M+Na]$^+$ found 315.1381, requires 315.1387 (−1.9 ppm).

**2-(Methyldiphenylsilyl)propanoic acid (S10)**

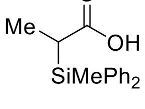

**S10**

Following General Procedure A, diisopropylamine (1.8 mL, 12.8 mmol, 2.1 equiv.), *n*-BuLi (5.1 mL, 12.8 mmol, 2.1 equiv.), 2-(methyldiphenylsilyl)acetic acid **S2** (1.56 g, 6.1 mmol, 1.0 equiv.),

iodomethane (370 µL, 6.3 mmol, 1.05 equiv.) in THF (15 mL) gave. After trituration, 2-(dimethyl(phenyl)silyl)propanoic acid as a white solid (1.24 g, 75%) with spectroscopic data in accordance with the literature.[7]

**$^1$H NMR** (400 MHz, $CDCl_3$) $\delta_H$: 0.70 (3H, s, Si*C**H**$_3$*), 1.25 (3H, d, *J* 7.1, CHC*H$_3$*), 2.70 (1H, q, *J* 7.1, COC*H*), 7.35 – 7.46 (6H, m, Ph*H*), 7.57 – 7.60 (4H, m, Ph*H*).

**$^{13}$C{$^1$H} NMR** (101 MHz, $CDCl_3$) $\delta_C$ −5.3 (Si*C*H$_3$), 11.9 (CH*CH$_3$*), 29.0 (CO*C*H), 127.9 (Ph$_A$*C*(2,6)), 128.0 (Ph$_B$*C*(2,6)), 129.8 (Ph*C*(4)), 133.8 (Ph$_A$*C*(4)), 134.2 (Ph$_B$*C*(4)), 134.8 (Ph$_A$*C*(3,5)), 134.9 (Ph$_B$*C*(3,5)), 181.7 (*C*O).

**2-(Methyldiphenylsilyl)propanoic pivalic anhydride (S11)**

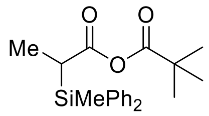


**S11**

2-(Methyl(diphenyl)silyl)propanoic acid **S10** (52 mg, 0.2 mmol, 1 equiv.) was added in MTBE (2 mL), pivaloyl chloride (50 µL, 0.4 mmol, 2 equiv.) and *N,N*-diisopropylethylamine (70 µL, 0.4 mmol, 2 equiv.) added, after 10 min at 0 ºC, filtered and diethyl ether washed, remove the solvent to give the product as bright yellow oil (47 mg, 80%).

**IR** $\nu_{max}$ (film) 1796 (C=O), 1049 (C−O).

**$^1$H NMR** (500 MHz, $CDCl_3$) $\delta_H$: 0.74 (3H, s, Si(C*H$_3$*Ph$_2$), 0.99 (9H, s, C(C*H$_3$*)$_3$), 1.30 (3H, d, *J* 7.0, CHC*H$_3$*), 2.82 (1H, q, *J* 7.0, COC*H*), 7.38 – 7.44 (6H, m, Ph*H*), 7.57 – 7.62 (2H, m, Ph*H*).

**$^{13}$C{$^1$H} NMR** (126 MHz, $CDCl_3$) $\delta_C$ −5.8 (Si(*C*H$_3$)), 11.8 (CH*CH$_3$*), 26.2 (C(*C*H$_3$)$_3$), 31.2 (CO*C*H), 39.5 (*C*(CH$_3$)$_3$), 128.1 (Ph*C*(2,6)), 130.0 (Ph*C*(4)), 133.5 (Ph$_A$*C*(1)), 133.6 (Ph$_B$*C*(1)), 134.8 (Ph$_A$*C*(3,5)), 134.8 (Ph$_B$*C*(3,5)), 172.2 (*C*OCH), 174.0 (*C*OC).

**HRMS** (ESI$^+$) $C_{21}H_{26}O_3NaSi$ [M+Na]$^+$ found 377.1539, requires 377.1543 (−1.1 ppm).

**3-Phenyl-2-(trimethylsilyl)propanoic acid (S12)**

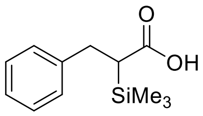

**S12**

Following General Procedure A, *N*,*N*-diisopropylamine (1.35 mL, 9.6 mmol, 2.1 equiv.), *n*-BuLi (3.8 mL, 9.6 mmol, 2.1 equiv.), 2-(trimethylsilyl) acetic acid **1** (600 mg, 4.5 mmol, 1.0 equiv.) and benzyl bromide (558 µL, 4.7 mmol, 1.05 equiv.) in THF (10 mL) gave, after trituration, 2-(trimethylsilyl)propanoic acid as a white solid (789 mg, 79%) with spectroscopic data in accordance with the literature.[8]

**$^1$H NMR** (400 MHz, $CDCl_3$) $\delta_H$: 0.18 (9H, s, Si(C*H*$_3$)$_3$), 2.41 (1H, dd, *J* 11.6, 3.1, COC*H*), 2.76 (1H, dd, *J* 14.5, 3.1 PhC*H*$_A$H$_B$), 3.12 (1H, dd, *J* 14.5, 11.8 PhCH$_A$*H*$_B$) 7.20 – 7.22 (3H, m, Ph*H*), 7.26 – 7.30 (2H, m, Ph*H*).

**$^{13}$C{$^1$H} NMR** (126 MHz, $CDCl_3$) $\delta_C$ −2.7 (Si(*C*H$_3$)$_3$), 32.5 (Ph*C*H$_2$), 39.8 (CO*C*H), 126.1 (Ph*C*(4)H), 128.2 (Ph*C*(2,6)H), 128.4 (Ph*C*(3,5)H), 141.7 (Ph*C*(1)), 181.1 (*C*O).

**3-(*p*-Tolyl)-2-(trimethylsilyl)propanoic acid (S13)**

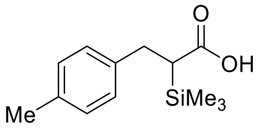

**S13**

Following General Procedure A, *N*,*N*-diisopropylamine (1.35 mL, 9.6 mmol, 2.1 equiv.), *n*-BuLi (3.8 mL, 9.6 mmol, 2.1 equiv.), 2-(trimethylsilyl) acetic acid **1** (600 mg, 4.5 mmol, 1.0 equiv.) and 1-(bromomethyl)-4-methylbenzene (770 µL, 5.5 mmol, 1.2 equiv.) in THF (10 mL) gave, after trituration, 3-(*p*-Tolyl)-2-(trimethylsilyl)propanoic acid as a white solid (670 mg, 63%).

**mp** 78 – 81 °C.

**IR** $\nu_{max}$ (film) 2955 (O−H), 1678 (C=O).

**$^1$H NMR** (400 MHz, $CDCl_3$) $\delta_H$ 0.19 (9H, s, Si(C*H*$_3$)$_3$), 2.34 (3H, s, ArC*H*$_3$), 2.39 (1H, dd, *J* 11.6, 3.1, C*H*CH$_2$), 2.73 (1H, dd, *J* 14.5, 3.0, CHC*H*$_2$), 3.09 (1H, dd, *J* 14.6, 11.8, CHC*H*$_2$), 7.09 – 7.13 (4H, m, Ar*H*).

**$^{13}$C{$^1$H} NMR** (101 MHz, $CDCl_3$) $\delta_C$ −2.7 (Si(*C*H$_3$)$_3$), 21.0 (Ar*C*H$_3$), 31.8 (CH*C*H$_2$), 39.9 (*C*HCH$_2$),

128.0 (Ar*C*(2,6)H), 119.1 (Ar*C*(3,5)H), 135.5 (Ar*C*(4)), 138.7 (Ar*C*(1)), 181.3 (*C*O);

**HRMS** (ESI$^+$) $C_{13}H_{20}O_2NaSi$ [M+Na]$^+$ found 259.1118, requires 259.1125 (−2.6 ppm).

**3-(4-Fluorophenyl)-2-(trimethylsilyl)propanoic acid (S14)**

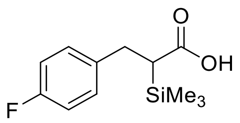

**S14**

Following General Procedure A, *N*,*N*-diisopropylamine (1.35 mL, 9.6 mmol, 2.1 equiv.), *n*-BuLi (3.8 mL, 9.6 mmol, 2.1 equiv.), 2-(trimethylsilyl) acetic acid **1** (600 mg, 4.5 mmol, 1.0 equiv.) and 1-(bromomethyl)-4-fluorobenzene (680 µL, 5.5 mmol, 1.2 equiv.) in THF (10 mL) gave, after trituration, 3-(4-fluorophenyl)-2-(trimethylsilyl)propanoic acid as a white solid (649 mg, 60%).

**mp** 64 – 67 °C.

**IR** $v_{max}$ (film) 2957 (O−H), 1680 (C=O).

**$^1$H NMR** (400 MHz, $CDCl_3$) $\delta_H$ 0.18 (9H, s, Si(C*H*$_3$)$_3$), 2.36 (1H, dd, *J* 11.9, 3.2, C*H*CH$_2$), 2.73 (1H, dd, *J* 14.6, 3.2, CHC*H*$_2$), 3.07 (1H, dd, *J* 14.6, 11.9, CHC*H*$_2$), 6.93 – 6.99 (2H, m, ArC(3,5)*H*), 7.14 – 7.18 (2H, m, ArC(2,6)*H*).

**$^{19}$F NMR** (377 MHz, $CDCl_3$) $\delta_F$ −117.3 (s).

**$^{13}$C{$^1$H} NMR** (101 MHz, $CDCl_3$) $\delta_C$ −2.8 (Si(*C*H$_3$)$_3$), 31.5 (CH*C*H$_2$), 40.1 (*C*HCH$_2$), 115.1 (d, *J* 21.1, Ar*C*(3,5)), 129.6 (d, *J* 7.8, Ar*C*(2,6)), 137.3 (Ar*C*(1)), 161.5 (d, *J* 244.3, Ar*C*(4)), 181.1 (*C*O);

**HRMS** (ESI$^+$) $C_{12}H_{17}O_2FNaSi$ [M+Na]$^+$ found 263.0869, requires 263.0874 (−1.9 ppm).

**2-(Trimethylsilyl)butanoic acid (S15)**

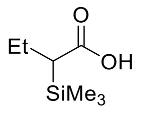

**S15**

Following General Procedure A, *N*,*N*-diisopropylamine (1.35 mL, 9.6 mmol, 2.1 equiv.), *n*-BuLi (3.8 mL, 9.6 mmol, 2.1 equiv.), 2-(trimethylsilyl) acetic acid **1** (600 mg, 4.5 mmol, 1.0 equiv.) and iodoethane (370 µL, 4.7 mmol, 1.05 equiv.) in THF (10 mL) gave, after purification by Biotage®

Isolera™ 4 [SNAP KP-Sil 25 g, 36 mL min$^{-1}$, petrol: $Et_2O$ (96:4 4 CV, 80:20 24 CV)], 2-(trimethylsilyl)butanoic acid as a white solid (374 mg, 52%) with spectroscopic data in accordance with the literature.[8]

**$^1$H NMR** (500 MHz, $CDCl_3$) $\delta_H$: 0.13 (9H, s, Si(C$H_3$)$_3$), 1.01 (3H, t, *J* 7.2, $CH_2CH_3$), 1.46 – 1.54 (1H, m, C$H_A$$H_B$), 1.77 – 1.86 (1H, m, C$H_A$$H_B$), 1.95 (1H, dd, *J* 11.5, 3.0, C*H*).

**$^{13}$C{$^1$H} NMR** (126 MHz, $CDCl_3$) $\delta_C$ −2.7 (Si($CH_3$)$_3$), 15.1 ($CH_3$), 20.0 ($CH_2$), 40.0 (*C*H), 182.0 (*C*O).

**2-(Trimethylsilyl)pentanoic acid (S16)**

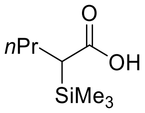


**S16**

Following General Procedure A, , *N*,*N*-diisopropylamine (1.35 mL, 9.6 mmol, 2.1 equiv.), *n*-BuLi (3.8 mL, 9.6 mmol, 2.1 equiv.), 2-(trimethylsilyl) acetic acid **1** (600 mg, 4.5 mmol, 1.0 equiv.) and 1-iodopropane (536 µL, 5.5 mmol, 1.2 equiv.) in THF (10 mL) gave, after trituration, 2-(trimethylsilyl)pentanoic acid as a white solid (628 mg, 80%) with spectroscopic data in accordance with the literature.[9]

**$^1$H NMR** (400 MHz, $CDCl_3$) $\delta_H$ 0.13 (9H, s, Si(C$H_3$)$_3$), 0.93 (3H, t, *J* 7.3, $CH_2CH_3$), 1.27 – 1.41 (2H, m, C$H_2$$CH_3$), 1.44 – 1.57 (1H, m, CHC$H_2$), 1.76 – 1.86 (1H, m, CHC$H_2$), 2.04 (1H, dd, *J* 11.6, 2.5, C*H*).

**$^{13}$C{$^1$H} NMR** (101 MHz, $CDCl_3$) $\delta_C$ −2.8 (Si($CH_3$)$_3$), 13.9 ($CH_3$), 23.6 ($CH_2CH_3$), 28.7 (CH$CH_2$), 37.8 (*C*(3)H), 182.1 (*C*O).

**3-(Naphthalen-2-yl)-2-(trimethylsilyl)propanoic acid (S17)**

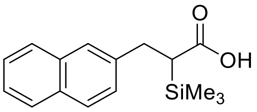


**S17**

Following General Procedure A, diisopropylamine (1.35 mL, 9.6 mmol, 2.1 equiv.), *n*-BuLi (3.8 mL, 9.6 mmol, 2.1 equiv.), 2-(trimethylsilyl) acetic acid **1** (600 mg, 4.5 mmol, 1.0 equiv.) and 2-

(bromomethyl)naphthalene (1.04 g, 4.7 mmol, 1.05 equiv.) in THF (10 mL) gave, after trituration, 2-(trimethylsilyl)propanoic acid as a white solid (771 mg, 63%) with spectroscopic data in accordance with the literature.[10]

**$^1$H NMR** (400 MHz, $CDCl_3$) $\delta_H$: 0.19 (9H, s, Si(C*H*$_3$)$_3$), 2.51 (1H, dd, *J* 11.6, 3.1, COC*H*), 2.92 (1H, dd, *J* 14.6, 3.1 ArC*H*$_A$H$_B$), 3.27 (1H, dd, *J* 14.7, 11.8 ArCH$_A$*H*$_B$), 7.34 (1H, dd, *J* 8.4, 1.7, Ar*H*), 7.40 – 7.49 (2H, m, Ar*H*), 7.66 (1H, s, Ar*H*), 7.74 – 7.82 (3H, m, Ar*H*).

**$^{13}$C{$^1$H} NMR** (126 MHz, $CDCl_3$) $\delta_C$ −2.7 (Si(*C*H$_3$)$_3$), 32.4 (Ph*C*H$_2$), 39.6 (CO*C*H), 125.2 (Ar*C*(6)), 125.9 (Ar*C*(7)), 126.3 (Ar*C*(4)), 127.0 (Ar*C*(3)), 127.6 (Ar*C*(5)), 127.6 (Ar*C*(8)), 128.0 (Ar*C*(1)), 132.1 (Ar*C*(10)), 133.6 (Ar*C*(9)), 139.2 (Ar*C*(2)), 180.5 (*C*O).

**2-(Trimethylsilyl)pent-4-enoic acid (S18)**

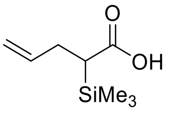

**S18**

Following General Procedure A, diisopropylamine (1.35 mL, 9.6 mmol, 2.1 equiv.), *n*-BuLi (3.8 mL, 9.6 mmol, 2.1 equiv.), 2-(trimethylsilyl) acetic acid **1** (600 mg, 4.5 mmol, 1.0 equiv.) and 3-bromoprop-1-ene (407 µL, 4.7 mmol, 1.05 equiv.) in THF (10 mL) gave, after trituration, 2-(trimethylsilyl)propanoic acid as a colourless oil (620 mg, 80%).

**IR** $\nu_{max}$ (film) 3001 (C-H), 912 (C-H), 1682 (C=O).

**$^1$H NMR** (400 MHz, $CDCl_3$) $\delta_H$: 0.15 (9H, s, Si(C*H*$_3$)$_3$), 2.13 − 2.21 (2H, m, COC*H*, C*H*$_A$H$_B$), 2.25 – 2.58 (1H, m, CH$_A$*H*$_B$), 4.99 – 5.01 (1H, m, CH=C*H*$_A$H$_B$), 5.06 – 5.11 (1H, m, CH=CH$_A$*H*$_B$), 5.82 – 5.90 (1H, m, C*H*=CH$_A$H$_B$).

**$^{13}$C{$^1$H} NMR** (126 MHz, $CDCl_3$) $\delta_C$ −2.7 (Si(*C*H$_3$)$_3$), 30.4 (*C*H$_2$CH=CH$_2$), 37.3 (CO*C*H), 115.1 (CH=*C*H$_2$), 137.6 (*C*H=CH$_2$), 181.4 (*C*O).

**HRMS** (ESI$^+$) $C_8H_{16}O_2NaSi$ [M+Na]$^+$ found 195.0812, requires 195.0812 (+0.1 ppm).

**2-(Trimethylsilyl)pent-4-ynoic acid (S19)**

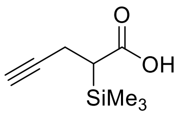

**S19**

Following General Procedure A, diisopropylamine (1.35 mL, 9.6 mmol, 2.1 equiv.), *n*-BuLi (3.8 mL, 9.6 mmol, 2.1 equiv.), 2-(trimethylsilyl) acetic acid **1** (600 mg, 4.5 mmol, 1.0 equiv.) and 3-bromoprop-1-yne (356 µL, 4.7 mmol, 1.05 equiv.) in THF (10 mL) gave, after triturated, 2-(trimethylsilyl)propanoic acid as a yellow oil (574 mg, 75%) with spectroscopic data in accordance with the literature.[11]

**IR** $\nu_{max}$ (film) 3308 (C-H), 1684 (C=O).

**$^1$H NMR** (400 MHz, $CDCl_3$) $\delta_H$: 0.18 (9H, s, Si(C*H*$_3$)$_3$), 2.00 – 2.01 (1H, m, COC*H*), 2.31 – 2.42 (2H, m, C*H*$_2$), 2.60 – 2.70 (1H, m, CC*H*).

**3-Methyl-2-(trimethylsilyl)butanoic acid (S20)**

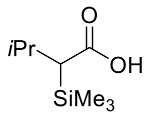

**S20**

Following General Procedure A, diisopropylamine (1.35 mL, 9.6 mmol, 2.1 equiv.), *n*-BuLi (3.8 mL, 9.6 mmol, 2.1 equiv.), 2-(trimethylsilyl) acetic acid **1** (600 mg, 4.5 mmol, 1.0 equiv.) and 2-bromopropane (2.4 mL, 22.5 mmol, 5.0 equiv.) in THF (10 mL) were reacted for 16 hours to give, after trituration, 2-(trimethylsilyl)propanoic acid as a colourless oil (470 mg, 60%).

**IR** $\nu_{max}$ (film) 1680 (C=O).

**$^1$H NMR** (300 MHz, $CDCl_3$) $\delta_H$: 0.17 (9H, s, Si(C*H*$_3$)$_3$), 1.05 (6H, dd, *J* 9.2, 6.4, CH(C*H*$_3$)$_2$), 1.81 (1H, d, *J* 10.0, C*H*CO), 2.11 – 2.23 (1H, m, C*H*(CH$_3$)$_2$).

**$^{13}$C{$^1$H} NMR** (126 MHz, $CDCl_3$) $\delta_C$ −1.5 (Si(*C*H$_3$)$_3$), 22.7 (CH(*C*$_A$H$_3$)$_2$), 23.5 (CH(*C*$_B$H$_3$)$_2$), 28.1 (*C*H(CH$_3$)$_2$), 46.4 (CO*C*H), 181.8 (*C*O).

**HRMS** (ESI$^+$) $C_8H_{17}O_2Si$ [M−H]$^-$ found 173.0994, requires 173.1003 (−5.0 ppm).

**2-Phenyl-2-(trimethylsilyl)acetic acid (S21)**

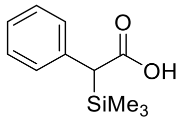


**S21**

Following a procedure reported by Cheng *et al*,[12] *n*-BuLi (15 mL, 12.0 mmol, 1.2 equiv.) was added to a solution of benzyltrimethylsilane (1.64 g, 10.0 mmol, 1.0 equiv.) in anhydrous THF (25 mL) dropwise over 40 min at 0 °C and the mixture was stirred at r.t. overnight. Carbon dioxide gas was bubbled into the solution which turned orange-brown until the color disappeared. The reaction was quenched by the addition of HCl (1 M) and the pH adjusted to pH=2, The organic layer was separated and the aqueous solution extracted with $Et_2O$ (3 × 30 mL), the combined organic layers was dried over $MgSO_4$, filtered and the solvent was removed under reduced pressure to give 2-phenyl-2-(trimethylsilyl)acetic acid as a white solid (832 mg, 40%) with spectroscopic data in accordance with the literature.[12]

**$^1$H NMR** (500 MHz, $CDCl_3$) $\delta_H$: 0.12 (9H, s, Si(C*H*$_3$)$_3$), 3.50 (1H, s, C*H*Ph), 7.21 – 7.24 (1H, m, Ph*H*), 7.31 – 7.36 (1H, m, Ph*H*).

**$^{13}$C{$^1$H} NMR** (126 MHz, $CDCl_3$) $\delta_C$ −2.4 (Si(*C*H$_3$)$_3$), 46.4 (*C*H(CH$_3$)$_2$), 125.8 (Ph*C*(4)H), 128.2 (Ph*C*(3,5)H), 128.5 (Ph*C*(2,6)H), 179.7 (*C*O).

*3.2 Enones and Perfluoroalkylketones*

**(*E*)-1,1,1-Trifluoro-4-phenylbut-3-en-2-one (40)**

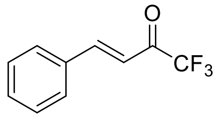


**40**

Following a procedure reported by Smith *et al.*,[13] methyl cinnamate (2.0g, 12.3 mmol, 1.0 equiv.) was dissolved in pentane (60 mL) and the solution was cooled to 0 °C. Trimethyl(trifluoromethyl)silane (2.3 mL, 15.5 mmol, 1.25 equiv.) and TBAF (68 µL, 0.25 mmol, 2 mol%) were added before the mixture was warmed to r.t. and stirred for 18 h. The solvent was removed under reduced pressure and the residue was dissolved in THF (10 mL) before aqueous

HCl was added (10 mL, 4 M). Subsequently the mixture was stirred for 18 h at r.t. before $Et_2O$ (50 mL) was added. The organic layer was washed with brine (2 × 40 mL), dried over $MgSO_4$, filtered and the solvent was removed under reduced pressure. The crude residue was purified by Biotage® Isolera™ 4 [SNAP KP-Sil 25 g, 36 mL min$^{-1}$, petrol : $Et_2O$ (98:2 4 CV, 98:2 to 96:4 30 CV)] to give the (*E*)-1,1,1-trifluoro-4-phenylbut-3-en-2-one as a pale yellow oil (1.2 g, 49%) with spectroscopic data in accordance with the literature.[13]

**$^1$H NMR** (400 MHz, $CDCl_3$) $\delta_H$: 7.05 (1H, dd, *J* 16.0, 0.9, C=C*H*CO), 7.46 – 7.55 (3H, m, Ar*H*), 7.66 – 7.69 (2H, m, Ar*H*), 8.00 (1H, d, *J* 16.0, ArC*H*=C).

**$^{19}$F NMR** (376 MHz, $CDCl_3$) $\delta_F$: −77.6 (C*F*$_3$).

**(*E*)-1,1,1-Trifluoro-3-methyl-4-phenylbut-3-en-2-one (41)**

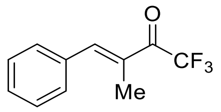


**41**

Following a procedure reported by Smith *et al*,[14] the benzaldehyde (0.50 mL, 5 mmol 1.0 equiv.), piperidine (0.49 mL, 5 mmol 1.0 equiv.) and acetic acid (0.43 mL, 7.5 mmol 1.5 equiv.) were dissolved in toluene (5 mL) at 0 °C. A solution of 1,1,1-trifluorobutan-2-one (1.36 mL, 10 mmol, 2 equiv.) in toluene (5 mL) was added and the reaction was stirred for 2 hours at 0 °C, followed by heating at 50 °C for 16 hours. The reaction was cooled to r.t. and quenched with saturated aqueous $NH_4Cl$ (10 mL). The organic layer was washed with water (2 × 10 mL), dried over $MgSO_4$, filtered and concentrated under reduced pressure to leave the crude product, after purification by Biotage® Isolera™ 4 [SNAP KP-Sil 25 g, 36 mL min$^{-1}$, petrol : $Et_2O$ (98:2 4 CV, 98:2 to 96:4 30 CV)] to leave (3*E*)-1,1,1-trifluoro-3-methyl-4-phenylbut-3-en-2-one (690 mg, 64%) as a colourless oil with spectroscopic data in accordance with the literature.[14]

**$^1$H NMR** (400 MHz, $CDCl_3$) $\delta_H$: 2.20 (3H, s, C*H*$_3$), 7.28 – 7.53 (5H, m, Ph*H*), 7.77 (1H, s, C*H*=C).

**$^{19}$F NMR** (376 MHz, $CDCl_3$) $\delta_F$: −68.9 (C*F*$_3$).

**1-(4-Bromophenyl)-2,2,3,3,3-pentafluoropropan-1-one (S24)**

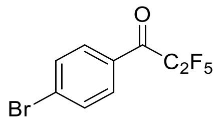

**S24**

Following General Procedure B, 1,4-dibromobenzene (2.36 g, 10 mmol), *n*-BuLi (2.5 M in hexanes, 4.4 mL, 11 mmol), ethyl pentafluoropropionate (4.14 mL, 28 mmol) and $BF_3{\cdot}OEt_2$ (1.5 mL, 12 mmol) gave, after distillation, 1-(4-bromophenyl)-2,2,3,3,3-pentafluoropropan-1-one as a pale yellow oil (1.60 g, 53%) with spectroscopic data in accordance with the literature.[3]

**$^1$H NMR** (400 MHz, $CDCl_3$) $\delta_H$: 7.71 – 7.75 (2H, m, ArC(3,5)*H*), 7.95 – 7.98 (2H, m, ArC(2,6)*H*).

**$^{19}$F NMR** (376 MHz, $CDCl_3$) $\delta_F$: −115.7 (C*F*$_2$), −81.6 (C*F*$_3$).

**1-(4-Bromophenyl)-2,2,3,3,4,4,5,5,5-nonafluoropentan-1-one (S25)**

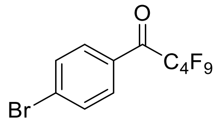

**S25**

Following General Procedure B, 1,4-dibromobenzene (2.5 g, 10.6 mmol), *n*-BuLi (2.5 M in hexanes, 4.7 mL, 11.7 mmol), ethyl perfluoropentanoate (4.6 g, 14.9 mmol) and $BF_3{\cdot}OEt_2$ (1.6 mL, 12.7 mmol) gave, after column chromatography (eluent $Et_2O$:hexane, 0% to 0.2%), 1-(4-bromophenyl)-2,2,3,3,4,4,5,5,5-nonafluoropentan-1-one as a colourless oil (2.75 g, 64%) with spectroscopic data in accordance with the literature.[3]

**$^1$H NMR** (400 MHz, $CDCl_3$) $\delta_H$: 7.71 – 7.74 (2H, m, ArC(3,5)*H*), 7.94 – 7.96 (2H, m, ArC(2,6)*H*).

**$^{19}$F NMR** (376 MHz, $CDCl_3$) $\delta_F$: −125.3 – −125.2 (m, C*F*$_2$), −122.0 – −121.9 (m, C*F*$_2$), −113.1 – −113.0 (m, C*F*$_2$), −80.9 (tt, *J* 10.0, 2.3, C*F*$_3$).

**2,2,3,3,3-Pentafluoro-1-(4-(trifluoromethyl)phenyl)propan-1-one (S26)**

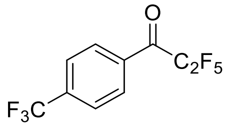

**S26**

Following General Procedure B, 4-bromobenzotrifuoride (2.8 mL, 20 mmol), *n*-BuLi (2.5 M in hexanes, 8.8 mL, 22 mmol), ethyl pentafluoropropionate (4.14 mL, 28 mmol) and $BF_3 \cdot OEt_2$ (3 mL, 24 mmol) gave, after column chromatography ($Et_2O$:hexane, 3:97), 2,2,3,3,3-pentafluoro-1-(4-(trifluoromethyl)phenyl)propan-1-one as a colourless oil (1.19 g, 20%) with spectroscopic data in accordance with the literature.[3]

**$^1$H NMR** (400 MHz, $CDCl_3$) $\delta_H$: 7.82 – 7.88 (2H, m, ArC(3,5)*H*), 8.20 – 8.26 (2H, m, ArC(2,6)*H*).

**$^{19}$F NMR** (376 MHz, $CDCl_3$) $\delta_F$: −115.9 (C*F*$_2$), −81.5 (C*F*$_3$), −63.6 (ArC(4)C*F*$_3$).

**2,2,3,3,3-Pentafluoro-1-(3-methoxyphenyl)propan-1-one (S27)**

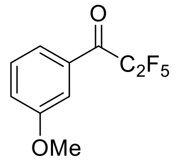


**S27**

Following General Procedure B, 3-iodoanisole (2.38 mL, 20 mmol), *n*-BuLi (2.5 M in hexanes, 8.8 mL, 22 mmol), ethyl pentafluoropropionate (4.14 mL, 28 mmol) and $BF_3 \cdot OEt_2$ (3 mL, 24 mmol) gave, after column chromatography ($Et_2O$:hexanes, 3:97), 2,2,3,3,3-pentafluoro-1-(3-methoxyphenyl)propan-1- one as a colourless oil (3.93 g, 78%) with spectroscopic data in accordance with the literature.[3]

**$^1$H NMR** (400 MHz, $CDCl_3$) $\delta_H$: 3.90 (3H, s, OC*H*$_3$), 7.26 – 7.29 (m, 1H, ArC(4)*H*), 7.48 (1H, t, *J* 8.2 , ArC(5)*H*), 7.59 (1H, dd, *J* 2.3, 1.7, ArC(2)*H*), 7.69 – 7.74 (1H, m, ArC(6)*H*).

**$^{19}$F NMR** (376 MHz, $CDCl_3$) $\delta_F$: −115.3 (C*F*$_2$), −81.6 (C*F*$_3$).

**2,2,3,3,3-Pentafluoro-1-(m-tolyl)propan-1-one (S28)**

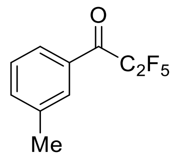


**S28**

Following General Procedure B, 3-bromotoluene (2.43 mL, 20 mmol), *n*-BuLi (2.5 M in hexanes, 8.8 mL, 22 mmol), ethyl pentafluoropropionate (4.14 mL, 28 mmol) and $BF_3 \cdot OEt_2$ (3 mL, 24 mmol)

gave, after distillation, 2,2,3,3,3-pentafluoro-1-(*m*-tolyl)propan-1-one as a light yellow oil (2.48 g, 52%) with spectroscopic data in accordance with the literature.[15]

**$^1$H NMR** (400 MHz, $CDCl_3$) $\delta_H$: 2.47 (3H, s, C*H*$_3$), 7.44 – 7.48 (1H, m, ArC(*5*)*H*), 7.53 – 7.56 (1H, m, Ar*C*(4)*H*), 7.91 – 7.92 (2H, m, ArC(2, 6)*H*).

**$^{19}$F NMR** (376 MHz, $CDCl_3$) $\delta_F$: −115.4 (C*F*$_2$), −81.6 (C*F*$_3$).

**2,2,3,3,4,4,5,5,5-Nonafluoro-1-phenylpentan-1-one (S29)**

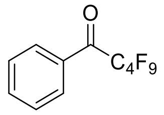


**S29**

Following General Procedure B, bromobenzene (2.12 g, 20 mmol), *n*-BuLi (2.2 M in hexanes, 10 mL, 44 mmol), ethyl perfluoropentanoate (8.16 g, 28 mmol) and $BF_3 \cdot OEt_2$ (2.96 mL, 24 mmol) gave, after distillation, 2,2,3,3,4,4,5,5,5-nonafluoro-1-phenylpentan-1-one as a colourless oil (4.83 g, 76%) with spectroscopic data in accordance with the literature.[3]

**$^1$H NMR** (400 MHz, $CDCl_3$) $\delta_H$: 7.55 – 7.60 (2H, m, PhC(3,5)*H*), 7.72 – 7.76 (1H, m, PhC(4)*H*), 8.09 – 8.12 (2H, m, PhC(2,6)*H*).

**$^{19}$F NMR** (376 MHz, $CDCl_3$) $\delta_F$: −125.3 – −125.2 (m, C*F*$_2$), −121.9 (m, C*F*$_2$), −121.9 (m, C*F*$_2$), −112.9 (m, C*F*$_2$), −81.0 (tt, *J* 10.1, 2.2, C*F*$_3$).

**2,2,3,3,3-Pentafluoro-1-(2-(trifluoromethyl)phenyl)propan-1-one (S30)**

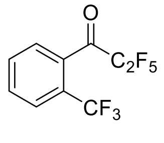


**S30**

Following General Procedure B, 2-bromobenzotrifuoride (2.8 mL, 20 mmol), *n*-BuLi (2.5 M in hexanes, 8.8 mL, 22 mmol), ethyl pentafluoropropionate (4.14 mL, 28 mmol) and $BF_3 \cdot OEt_2$ (3 mL, 24 mmol) gave, after column chromatography ($Et_2O$:hexane, 3:97), 2,2,3,3,3-pentafluoro-1-(2-(trifluoromethyl)phenyl)propan-1-one as a colourless oil (1.36 g, 23%) with spectroscopic data in accordance with the literature.[15]

**$^{1}$H NMR** (400 MHz, $CDCl_3$) $\delta_H$: 7.62 – 7.64 (1H, m, PhC(3,5)*H*), 7.72 – 7.79 (2H, m, PhC(4)*H*), 7.84 – 7.87 (1H, m, PhC(4,6)*H*).

**$^{19}$F NMR** (376 MHz, $CDCl_3$) $\delta_F$: −118.1 (m, C$F_2$), −81.4 (m, C$F_3$), −58.5 (m, ArC(2)C$F_3$).

## 4. Catalysis Products

### **(*S*)-4-(4-Bromophenyl)-4-(trifluoromethyl)oxetan-2-one (3)**

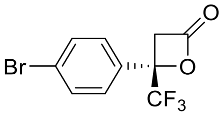

**3**

Following General Procedure D, 2-(trimethylsilyl) acetic acid (53 mg, 0.4 mmol), *N*,*N*-diisopropylethylamine (105 µL, 0.6 mmol), pivaloyl chloride (66 µL, 0.6 mmol) and MTBE (2 mL) for 15 mins, followed by 1-(4-bromophenyl)-2,2,2-trifluoroethan-1-one (50 mg, 0.2 mmol), (2*S*,3*R*)−HyperBTM (3 mg, 10 µmol) and *N*,*N*-diisopropylethylamine (35 µL, 0.2 mmol) for 16 h gave, after purification by Biotage® Isolera™ 4 [SNAP KP-Sil 25 g, 36 mL min$^{-1}$, petrol : $Et_2O$ (98:2 4 CV, 98:2 to 95:5 40 CV)], the title compound (57 mg, 96%) as a colorless oil.

$[\alpha]_D^{20}$ −25.0 (*c* 3.7, $CHCl_3$).

**Chiral HPLC analysis**, Chiralpak IB (99.3:0.7 hexane:IPA, flow rate 1.0 mLmin$^{-1}$, 254 nm, 30 °C), $t_R$ (major): 9.3 min, $t_R$ (minor):10.3 min, 94:6 er.

**IR** $\nu_{max}$ (film) 1848 (C=O), 1175 (C−O).

**$^1$H NMR** (400 MHz, $CDCl_3$) $\delta_H$ 3.72 − 3.76 (1H, dq, *J* 16.5, 2.2, C(3)$H_AH_B$), 4.13 (1H, d, *J* 16.5, C(3)$H_AH_B$), 7.36 – 7.38 (2H, m, ArC(2,6)*H*), 7.63 – 7.66 (2H, m, ArC(3,5)*H*).

**$^{19}$F NMR** (376 MHz, $CDCl_3$) $\delta_F$ −79.8 (C$F_3$).

**$^{13}$C{$^1$H} NMR** (126 MHz, $CDCl_3$) $\delta_C$ 46.9 (*C*(3)$H_2$), 74.6 (q, *J* 33.5, *C*(4)), 123.0 (q, *J* 281.3, *C*$F_3$), 124.8 (Ar*C*(4)Br), 128.4 (Ar*C*(2,6)H), 130.6 (Ar*C*(1)), 132.1 (Ar*C*(3,5)H), 163.0 (*C*(2)).

**HRMS** (ESI$^+$) $C_{10}H_7O_3BrF_3O_3$ [M+OH]$^-$ found 310.9534, requires 310.9536 (−0.6 ppm).

### **(3*S*,4*S*)-4-(4-Bromophenyl)-3-methyl-4-(trifluoromethyl)oxetan-2-one (7)**

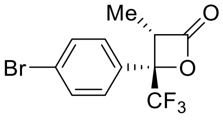

**7**

Following General Procedure D, 2-(trimethylsilyl)propanoic acid (58 mg, 0.4 mmol), *N*,*N*-

diisopropylethylamine (105 μL, 0.6 mmol), pivaloyl chloride (66 μL, 0.6 mmol) and MTBE (2 mL) for 15 mins, followed by 1-(4-bromophenyl)-2,2,2-trifluoroethan-1-one (50 mg, 0.2 mmol), (2*S*,3*R*)−HyperBTM (3 mg, 10 μmol) and *N*,*N*-diisopropylethylamine (35 μL, 0.2 mmol) for 16 h gave, after purification by Biotage® Isolera™ 4 [SNAP KP-Sil 25 g, 36 mL min$^{-1}$, petrol : $Et_2O$ (98:2 4 CV, 98:2 to 95:5 40 CV)], the title compound (50 mg, 81%) as a colourless oil.

$[\alpha]_D^{20}$ −60.6 (*c* 0.8, $CHCl_3$).

**Chiral HPLC analysis**, Chiralpak AS-H (99.5:0.5 hexane:IPA, flow rate 1.0 mLmin$^{-1}$, 211 nm, 30 °C), $t_R$ (minor): 4.6 min, $t_R$ (major): 5.7 min, 97:3 er.

**IR** $\nu_{max}$ (film) 1848 (C=O), 1175 (C−O).

**$^1$H NMR** (400 MHz, $CDCl_3$) $\delta_H$ 1.14 (3H, d, *J* 7.7, C(3)HC*H*$_3$), 4.23 (1H, q, *J* 7.8, C(3)*H*), 7.33 – 7.35 (2H, m, ArC(2,6)*H*), 7.63 – 7.66 (2H, m, ArC(3,5)*H*).

**$^{19}$F NMR** (376 MHz, $CDCl_3$) $\delta_F$ −78.4 (C*F*$_3$).

**$^{13}$C{$^1$H} NMR** (126 MHz, $CDCl_3$) $\delta_C$ 10.8 (C(3)H*C*H$_3$), 51.9 (*C*(3)H), 78.9 (q, *J* 33.2, *C*(4)), 123.5 (q, *J* 281.0, *C*F$_3$), 124.6 (Ar*C*(4)Br), 128.0 (Ar*C*(2,6)H), 128.3 (Ar*C*(1)), 132.2 (Ar*C*(3,5)H), 167.5 (*C*(2)).

**HRMS** (ESI$^+$) $C_{11}H_9O_3F_3$ [M+OH]$^-$ found 324.9689, requires 324.9693 (−1.2 ppm).

**(3*S*,4*S*)-4-(4-Bromophenyl)-3-ethyl-4-(trifluoromethyl)oxetan-2-one (8)**

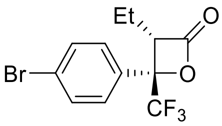


**8**

Following General Procedure D, 2-(trimethylsilyl)butanoic acid (64 mg, 0.4 mmol), *N*,*N*-diisopropylethylamine (105 μL, 0.6 mmol), pivaloyl chloride (66 μL, 0.6 mmol) and MTBE (2 mL) for 15 mins, followed by 1-(4-bromophenyl)-2,2,2-trifluoroethan-1-one (50 mg, 0.2 mmol), (2*S*,3*R*)−HyperBTM (3 mg, 10.0 μmol) and *N*,*N*-diisopropylethylamine (35 μL, 0.2 mmol) for 18 h gave, after purification by Biotage® Isolera™ 4 [SNAP KP-Sil 10 g, 36 mL min$^{-1}$, petrol : $Et_2O$ (98:2 4 CV, 98:2 to 95:5 30 CV)], the title compound (56 mg, 87%) as a colourless oil.

$[\alpha]_D^{20}$ −72.4 (*c*, 0.4, $CHCl_3$).

**Chiral HPLC analysis**, Chiralpak IB (99.9:0.1 hexane:IPA, flow rate 1.0 mLmin$^{-1}$, 211 nm, 30 °C), $t_R$ (minor): 5.4 min, $t_R$ (major): 5.9 min, 96:4 er.

**IR** $\nu_{max}$ (film) 1850 (C=O), 1171 (C−O).

**$^1$H NMR** (500 MHz, $CDCl_3$) $\delta_H$ 1.03 (3H, t, *J* 7.4, C(3)$HCH_2CH_3$), 1.32 – 1.41 (1H, m, C(3)HC$H_AH_B$), 1.52 – 1.60 (1H, m, C(3)HC$H_AH_B$), 4.03 (1H, dd, *J* 10.3, 6.3, C(3)*H*), 7.36 – 7.37 (2H, m, ArC(2,6)*H*), 7.64 – 7.65 (2H, m, ArC(3,5)*H*).

**$^{19}$F NMR** (471 MHz, $CDCl_3$) $\delta_F$ −78.1 (s, C$F_3$).

**$^{13}$C{$^1$H} NMR** (126 MHz, $CDCl_3$) $\delta_C$ 11.0 ($CH_2CH_3$), 19.9 ($CH_2CH_3$), 58.2 (C(3)H), 78.4 (q, *J* 34.0, *C*$CF_3$), 123.5 (q, *J* 282.7, *C*$F_3$), 124.5 (Ar*C*(4)Br), 128.1 (Ar*C*(2,6)H), 128.4 (Ar*C*(1)), 132.2 (Ar*C*(3,5)H), 167.0 (*C*O).

**HRMS** (ESI$^+$) $C_{12}H_{11}BrO_3F_3$ $[M+OH]^-$ found 338.9846, requires 338.9849 (−0.9 ppm).

**(3*S*,4*S*)-4-(4-Bromophenyl)-3-propyl-4-(trifluoromethyl)oxetan-2-one (9)**

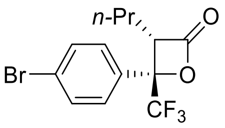


**9**

Following General Procedure D, 2-(trimethylsilyl)pentanoic acid (70 mg, 0.4 mmol), *N*,*N*-diisopropylethylamine (105 µL, 0.6 mmol), pivaloyl chloride (66 µL, 0.6 mmol) and MTBE (2 mL) for 15 mins, followed by 1-(4-bromophenyl)-2,2,2-trifluoroethan-1-one (50 mg, 0.2 mmol), (2*S*,3*R*)−HyperBTM (3 mg, 10.0 µmol) and *N*,*N*-diisopropylethylamine (35 µL, 0.2 mmol) for 18 h gave, after purification by Biotage® Isolera™ 4 [SNAP KP-Sil 10 g, 36 mL $min^{-1}$, petrol : $Et_2O$ (98:2 4 CV, 98:2 to 95:5 10 CV)], the title compound (50 mg, 74%) as a colourless oil.

$[\alpha]_D^{20}$ −74.0 (*c*, 0.3, $CHCl_3$).

**IR** $\nu_{max}$ (film) 1850 (C=O), 1169 (C−O).

**Chiral HPLC analysis**, Chiralcel OJ-H (99.8:0.2 hexane:IPA, flow rate 1.0 $mLmin^{-1}$, 211 nm, 30 °C), **major diastereoisomer**: $t_R$ (major): 5.9 min, $t_R$ (minor): 6.5 min, >99:1 er; **minor diastereoisomer**: Chiralcel OD-H (99.9:0.1 hexane:IPA, flow rate 1.0 $mLmin^{-1}$, 211 nm, 30 °C), $t_R$ (major): 8.5 min, $t_R$ (minor): 9.3 min, 90:10 er.

**$^1$H NMR** (500 MHz, $CDCl_3$) **major diastereoisomer**: $\delta_H$ 0.89 (3H, t, *J* 7.1, C(3)$HCH_2CH_2CH_3$), 1.26 – 1.33 (1H, m, C(3)$HCH_2CH_AH_B$), 1.37 – 1.47 (2H, m, C(3)$HCH_2CH_AH_B$), 1.49 – 1.57 (1H, m, C(3)HC$H_2$), 4.09 (1H, dd, *J* 10.3, 5.9, C(3)*H*), 7.35 – 7.37 (2H, m, ArC(2,6)*H*), 7.64 – 7.66 (2H, m,

ArC(3,5)*H*); **minor diastereoisomer**: $\delta_H$ 1.05 (3H, t, *J* 7.4, C(3)HCH$_2$CH$_2$C*H*$_3$), 1.65 – 1.75 (1H, m, C*H*$_2$), 1.97 – 2.17 (2H, m, C*H*$_2$), 3.90 (1H, dd, *J* 9.5, 6.6, C(3)*H*), 7.33 – 7.35 (2H, m, ArC(2,6)*H*), 7.62 – 7.64 (2H, m, ArC(3,5)*H*).

**$^{19}$F NMR** (471 MHz, CDCl$_3$) **major diastereoisomer**: $\delta_F$ −78.1 (s, C*F*$_3$); **minor diastereoisomer**: $\delta_F$ −74.0 (s, C*F*$_3$).

**$^{13}$C{$^1$H} NMR** (126 MHz, CDCl$_3$) **major diastereoisomer**: $\delta_C$ 13.6 (CH$_2$*C*H$_3$), 19.8 (*C*H$_2$CH$_3$), 28.2 (C(3)H*C*H$_2$), 56.7 (C(3)H), 78.6 (q, *J* 32.7, *C*CF$_3$), 123.5 (q, *J* 282.1, *C*F$_3$), 124.5 (Ar*C*(4)Br), 128.1 (Ar*C*(2,6)H), 128.4 (Ar*C*(1)), 132.2 (Ar*C*(3,5)H), 167.1 (*C*O); **minor diastereoisomer**: $\delta_C$ 13.7 (CH$_2$*C*H$_3$), 21.0 (*C*H$_2$CH$_3$), 26.9 (C(3)H*C*H$_2$), 61.7 (C(3)H), 127.9 (Ar*C*(2,6)H), 132.0 (Ar*C*(3,5)H);

**HRMS** (ESI$^+$) $C_{13}H_{13}BrO_3F_3$ [M+OH]$^-$ found 353.0009, requires 353.0006 (+0.9 ppm).

**(3*S*,4*S*)-3-Allyl-4-(4-bromophenyl)-4-(trifluoromethyl)oxetan-2-one (10)**

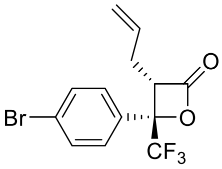


**10**

Following General Procedure D, 2-(trimethylsilyl)pent-4-enoic acid (69 mg, 0.4 mmol), *N*,*N*-diisopropylethylamine (105 µL, 0.6 mmol), pivaloyl chloride (66 µL, 0.6 mmol) and MTBE (2 mL) for 15 mins, followed by 1-(4-bromophenyl)-2,2,2-trifluoroethan-1-one (50 mg, 0.2 mmol), (2*S*,3*R*)−HyperBTM (3 mg, 10 µmol) and *N*,*N*-diisopropylethylamine (35 µL, 0.2 mmol) for 16 h gave, after purification by column chromatography, petrol : Et$_2$O (98:2 to 92:8) to give the compound (46 mg, 68%) as a colourless oil.

$[\alpha]_D^{20}$ +41.1 (*c*, 0.5, CHCl$_3$).

**Chiral HPLC analysis**, Chiralcel OD-H (99.5:0.5 hexane:IPA, flow rate 1.0 mL min$^{-1}$, 211 nm, 30 °C), **major diastereoisomer**: $t_R$ (minor): 9.4 min, $t_R$ (major): 10.3 min, 96:4 er; **minor diastereoisomer**: $t_R$ (major): 13.6 min, $t_R$ (minor): 14.8 min, 72:28 er.

**IR** $\nu_{max}$ (film) 1854 (C=O), 1175 (C−O).

**$^1$H NMR** (400 MHz, CDCl$_3$) **major diastereoisomer**: $\delta_H$ 2.20 – 2.24 (2H, m, C(3)HC*H*$_2$), 4.23 (1H, t, *J* 7.8, C(3)*H*), 5.07 (1H, dq, *J* 17.1, 1.2, CH=C*H*$_A$H$_B$), 5.16 (1H, dq, *J* 10.3, 1.2, CH=CH$_A$*H*$_B$), 5.64

– 5.74 (1H, m, C*H*=CH$_2$), 7.37 – 7.39 (2H, m, ArC(2,6)*H*), 7.63 – 7.66 (2H, m, ArC(3,5)*H*); **minor diastereoisomer**: $\delta_H$ 2.79 – 2.94 (2H, m, C(3)HC*H*$_2$), 3.99 (1H, t, *J* 8.0, C(3)*H*), 5.28 – 5.36 (2H, m, CH=C*H*$_2$), 5.87 – 5.95 (1H, m, C*H*=CH$_2$), 7.33 – 7.35 (2H, m, ArC(2,6)*H*), 7.53 – 7.63 (2H, m, ArC(3,5)*H*).

**$^{19}$F NMR** (376 MHz, CDCl$_3$) **major diastereoisomer**: $\delta_F$ −78.1 (C*F*$_3$); **minor diastereoisomer**: $\delta_F$ −73.8 (C*F*$_3$).

**$^{13}$C{$^1$H} NMR** (126 MHz, CDCl$_3$) **major diastereoisomer**: $\delta_C$ 29.7 (*C*H$_2$), 56.1 (C(3)H), 78.9 (q, *J* 33.3, *C*(4)), 118.8 (CH=*C*H$_2$), 120.7 (q, *J* 273.0, *C*F$_3$), 124.7 (Ar*C*(4)), 124.8 (Ar*C*(1)), 128.0 (*C*H=CH$_2$), 128.3 (Ar*C*(2,6)H), 132.1 (Ar*C*(3,5)H), 166.5 (*C*(2)); **minor diastereoisomer**: $\delta_C$ 29.1 (*C*H$_2$), 61.4 (C(3)H), 118.8 (CH=*C*H$_2$), 120.5 (q, *J* 255.6, *C*F$_3$), 124.5 (Ar*C*(4)Br), 124.6 (Ar*C*(1)), 127.9 (*C*H=CH$_2$), 131.6 (Ar*C*(2,6)H), 132.0 (Ar*C*(3,5)H), 168.3 (*C*(2)).

**HRMS** (ESI$^+$) $C_{13}H_{11}BrF_3O_3$ [M+OH]$^-$ found 350.9849, requires 350.9849 (−0.1 ppm).

**(3*S*,4*S*)-4-(4-Bromophenyl)-3-(prop-2-yn-1-yl)-4-(trifluoromethyl)oxetan-2-one (11)**

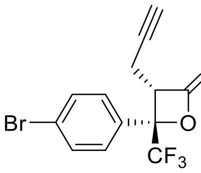


**11**

Following General Procedure D, 2-(trimethylsilyl)pent-4-ynoic acid (68 mg, 0.4 mmol), *N*,*N*-diisopropylethylamine (105 μL, 0.6 mmol), pivaloyl chloride (66 μL, 0.6 mmol) and MTBE (2 mL) for 15 mins, followed by 1-(4-bromophenyl)-2,2,2-trifluoroethan-1-one (50 mg, 0.2 mmol), (2*S*,3*R*)−HyperBTM (3 mg, 10 μmol) and *N*,*N*-diisopropylethylamine (35 μL, 0.2 mmol) for 16 h gave, after purification by Biotage® Isolera™ 4 [SNAP KP-Sil 10 g, 36 mL min$^{-1}$, petrol : Et$_2$O (98:2 4 CV, 98:2 to 94:6 30 CV)], the title compound (45 mg, 68%) as a colourless oil.

$[\alpha]_D^{20}$ +16.9 (*c*, 0.6, CHCl$_3$).

**Chiral HPLC analysis**, Chiralcel OJ-H (99.5:0.5 hexane:IPA, flow rate 1.0 mLmin$^{-1}$, 211 nm, 30 °C), $t_R$ (major): 13.7 min, $t_R$ (minor): 17.4 min, 98:2 er.

**IR** $\nu_{max}$ (film) 3302 (C≡C), 1856 (C=O), 1177 (C−O).

**$^1$H NMR** (300 MHz, CDCl$_3$) **major diastereoisomer**: $\delta_H$ 2.10 (1H, t, *J* 2.7, C(3)*H*), 2.30 (1H, qd, *J*

9.5, 2.7, C(3)HC$H_A$H$_B$), 2.55 (1H, dq, *J* 17.6, 2.7, C(3)HCH$_A$$H_B$), 4.35 (1H, dd, *J* 9.5, 5.0, C≡C*H*), 7.47 – 7.50 (2H, m, ArC(2,6)*H*), 7.64 – 7.69 (2H, m, ArC(3,5)*H*); **minor diastereoisomer**: $\delta_H$ 3.00 – 3.04 (2H, m, C(3)HC$H_2$), 4.19 (1H, dd, *J* 9.1, 7.1, C≡C*H*).

**$^{19}$F NMR** (282 MHz, $CDCl_3$) **major diastereoisomer**: $\delta_F$ −78.4 (s, C$F_3$); **minor diastereoisomer**: −74.0 (s, C$F_3$).

**$^{13}$C{$^1$H} NMR** (126 MHz, $CDCl_3$) **major diastereoisomer**: $\delta_C$ 15.5 (C(3)H*C*$H_2$), 56.1 (C(3)H), 72.1 (*C*≡*C*H), 79.1 (q, *J* 33.0, *C*$CF_3$), 123.1 (q, *J* 283.2, *C*$F_3$), 125.0 (Ar*C*(4)Br), 127.2 (Ar*C*(1)), 128.5 (Ar*C*(2,6)H), 132.2 (Ar*C*(3,5)H), 164.8 (*C*O); **minor diastereoisomer**: $\delta_C$ 60.1 (C(3)H), 71.7 (*C*≡*C*H), 128.4 (Ar*C*(2,6)H), 132.1 (Ar*C*(3,5)H).

**HRMS** (ESI$^+$) $C_{13}H_9BrF_3O_3$ $[M+OH]^-$ found 348.9686, requires 348.9693 (−2.0 ppm).

**(3*S*,4*S*)-3-Benzyl-4-(4-bromophenyl)-4-(trifluoromethyl)oxetan-2-one (12)**

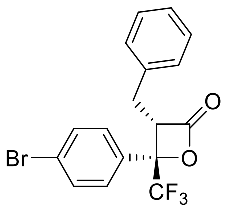


**12**

Following General Procedure D, 3-phenyl-2-(trimethylsilyl)propanoic acid (90 mg, 0.4 mmol), *N*,*N*-diisopropylethylamine (105 µL, 0.6 mmol), pivaloyl chloride (66 µL, 0.6 mmol) and MTBE (2 mL) for 15 mins, followed by 1-(4-bromophenyl)-2,2,2-trifluoroethan-1-one (50 mg, 0.2 mmol), (2*S*,3*R*)−HyperBTM (3 mg, 10 µmol) and *N*,*N*-diisopropylethylamine (35 µL, 0.2 mmol) for 20 h gave, after purification by Biotage® Isolera™ 4 [SNAP KP-Sil 25 g, 36 mL min$^{-1}$, petrol : $Et_2O$ (98:2 4 CV, 98:2 to 95:5 40 CV)], the title compound (70 mg, 72%) as a colourless oil.

$[\alpha]_D^{20}$ +3.0 (*c* 1.3, $CHCl_3$).

**Chiral HPLC analysis**, **major diastereoisomer**: Chiralpak IB (99.9:0.1 hexane:IPA, flow rate 1.0 mLmin$^{-1}$, 211 nm, 30 °C), $t_R$ (minor): 20.9 min, $t_R$(major): 21.5 min, >99:1 er; **minor diastereoisomer**: Chiralpak IB (99.9:0.1 hexane:IPA, flow rate 1.0 mLmin$^{-1}$, 254 nm, 30 °C), $t_R$ (major): 19.0 min, $t_R$ (minor): 35.0 min, 82:18 er.

**IR** $\nu_{max}$ (film) 1854 (C=O), 1177 (C−O).

$^{1}$**H NMR** (400 MHz, $CDCl_3$) **major diastereoisomer**: $\delta_H$ 2.72 (1H, dd, *J* 15.1, 7.9, C(3)HC$H_AH_B$), 2.81, (1H, dd, *J* 15.1, 8.5 C(3)HC$H_A$$H_B$), 4.49 (1H, t, *J* 8.2, C(3)*H*), 7.07 – 7.09 (2H, m, C(4)ArC(2,6)*H*), 7.28 – 7.39 (5H, m, C(3)H$CH_2$Ph*H*), 7.60 – 7.63 (2H, m, C(4)ArC(3,5)*H*); **minor diastereoisomer**: $\delta_H$ 3.32 (1H, dd, *J* 14.9, 7.9, C(3)HC$H_AH_B$), 5.51, (1H, dd, *J* 14.8, 7.7 C(3)C$H_A$$H_B$), 4.22 (1H, t, *J* 7.7, C(3)*H*), 7.33 – 7.36 (5H, m, C(3)H$CH_2$Ph*H*), 7.39 – 7.42 (2H, m, C(4)ArC(2,6)*H*), 7.52 – 7.56 (2H, m, C(4)ArC(3,5)*H*).

$^{19}$**F NMR** (377 MHz, $CDCl_3$) **major diastereoisomer**: $\delta_F$ −77.8 (C$F_3$); **minor diastereoisomer**: $\delta_F$ −73.7 (C$F_3$).

$^{13}$**C{$^{1}$H} NMR** (126 MHz, $CDCl_3$) **major diastereoisomer**: $\delta_C$ 31.6 (C(3)H*C*$H_2$Ph), 57.3 (*C*(3)H), 79.1 (q, *J* 33.6, *C*(4)), 123.5 (q, *J* 283.7, *C*$F_3$), 124.7 (C(4)Ar*C*(4)Br), 127.4 (C(3)H$CH_2$Ph*C*(4)H), 127.9 (C(3)H$CH_2$Ph*C*(1)), 128.4 (C(3)H$CH_2$Ph*C*(2,6)H), 128.4 (C(3)H$CH_2$Ph*C*(3,5)H), 128.8 (C(4)Ar*C*(2,6)H), 132.1 (C(4)Ar*C*(3,5)H), 135.0 (C(4)Ar*C*(1)), 166.5 (C(2)); **minor diastereoisomer**: $\delta_C$ 30.9 (C(3)H*C*$H_2$Ph), 63.2 (*C*(3)H), 123.2 (q, *J* 284.0, *C*$F_3$), 127.5 (C(3)H$CH_2$Ph*C*(4)H), 127.9 (C(3)H$CH_2$Ph*C*(2,6)H), 128.7 (C(3)H$CH_2$Ph*C*(3,5)H), 129.1 (C(4)Ar*C*(2,6)H), 132.0 (C(4)Ar*C*(3,5)H), 166.4 (C(2)).

**HRMS** (ESI$^+$) $C_{17}H_{12}O_2BrF_3Na$ [M+Na]$^+$ found 406.9874, requires 406.9865 (+2.2 ppm).

**(3*S*,4*S*)-4-(4-Bromophenyl)-3-(4-methylbenzyl)-4-(trifluoromethyl)oxetan-2-one (13)**

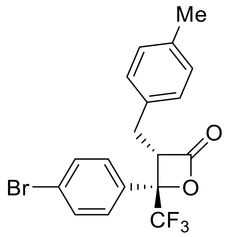


**13**

Following General Procedure D, 3-(*p*-tolyl)-2-(trimethylsilyl)propanoic acid (95 mg, 0.4 mmol), *N*,*N*-diisopropylethylamine (105 µL, 0.6 mmol), pivaloyl chloride (66 µL, 0.6 mmol) and MTBE (2 mL) for 15 mins, followed by 1-(4-bromophenyl)-2,2,2-trifluoroethan-1-one (50 mg, 0.2 mmol), (2*S*,3*R*)−HyperBTM (3 mg, 10 µmol) and *N*,*N*-diisopropylethylamine (35 µL, 0.2 mmol) for 18 h gave, after purification by Biotage® Isolera™ 4 [SNAP KP-Sil 10 g, 36 mL min$^{-1}$, petrol : $Et_2O$ (98:2 4 CV, 98:2 to 96:4 10 CV)], the title compound (56 mg, 70%) as a colourless oil.

$[\alpha]_D^{20}$ +10.3 (*c*, 0.6, $CHCl_3$).

**IR** $\nu_{max}$ (film) 1850 (C=O), 1171 (C−O).

**Chiral HPLC analysis**, Chiralpak IB (99.9:0.1 hexane:IPA, flow rate 1.0 mLmin$^{-1}$, 211 nm, 30 °C), **major diastereoisomer**: $t_R$ (major): 16.5 min, $t_R$ (minor): 21.3 min, 97:3 er; **minor diastereoisomer**: $t_R$ (major): 14.5 min, $t_R$ (minor): 23.7 min, 83:17 er.

**$^1$H NMR** (500 MHz, $CDCl_3$) **major diastereoisomer**: $\delta_H$ 2.35 (3H, s, ArC*H*$_3$), 2.66 – 2.78 (2H, m, C(3)HC*H*$_2$), 4.46 (1H, t, *J* 8.1, C(3)*H*), 6.96 – 6.98 (2H, m, C(3)HCH$_2$ArC(2,6)*H*), 7.12 − 7.14 (2H, m, C(4)ArC(2,6)*H*), 7.28 – 7.30 (2H, m, C(3)HCH$_2$ArC(3,5)*H*), 7.61 – 7.63 (2H, m, C(4)ArC(3,5)*H*); **minor diastereoisomer**: $\delta_H$ 2.39 (3H, s, ArC*H*$_3$), 3.26 – 3.49 (2H, m, C(3)HC*H*$_2$), 4.20 (1H, t, *J* 7.9, C(3)*H*), 6.96 – 6.98 (2H, m, C(3)HCH$_2$ArC(2,6)*H*), 7.10 – 7.12 (2H, m, C(4)ArC(2,6)*H*), 7.20 – 7.23 (2H, m, C(3)HCH$_2$ArC(3,5)*H*), 7.53 – 7.55 (2H, m, C(4)ArC(3,5)*H*).

**$^{19}$F NMR** (471 MHz, $CDCl_3$) **major diastereoisomer**: $\delta_F$ −77.9 (C*F*$_3$); **minor diastereoisomer**: $\delta_F$ −73.7 (C*F*$_3$).

**$^{13}$C{$^1$H} NMR** (126 MHz, $CDCl_3$) **major diastereoisomer**: $\delta_C$ 21.1 (Ar*C*H$_3$), 31.3 (C(3)H*C*H$_2$Ar), 57.4 (*C*(3)H), 79.1 (q, *J* 32.8, *C*(4)), 123.4 (q, *J* 283.3, *C*F$_3$), 124.7 (C(4)Ar*C*(4)Br), 127.9 (C(3)HCH$_2$Ar*C*(1)), 128.3 (C(3)HCH$_2$Ar*C*(2,6)H), 128.4 (C(4)Ar*C*(2,6)H), 129.5 (C(3)HCH$_2$Ar*C*(3,5)H), 131.9 (C(3)HCH$_2$Ar*C*(4)CH$_3$), 132.1 (C(4)Ar*C*(3,5)H), 137.1 (C(4)Ar*C*(1)), 166.6 (C(2)); **minor diastereoisomer**: $\delta_C$ 26.5 (Ar*C*H$_3$), 30.5 (C(3)H*C*H$_2$Ar), 63.3 (*C*(3)H), 123.2 (q, *J* 283.4, *C*F$_3$), 124.4 (C(4)Ar*C*(4)Br), 128.0 (C(3)HCH$_2$Ar*C*(2,6)H), 128.6 (C(4)Ar*C*(2,6)H), 129.7 (C(3)HCH$_2$Ar*C*(3,5)H), 132.0 (C(4)Ar*C*(3,5)H), 133.1 (C(3)HCH$_2$Ar*C*(4)CH$_3$), 137.2 (C(4)Ar*C*(1)).

**HRMS** (ESI$^+$) $C_{18}H_{14}O_2BrF_3Na$ $[M+Na]^+$ found 421.0012, requires 421.0021 (−2.2 ppm).

**(3*S*,4*S*)-4-(4-Bromophenyl)-3-(4-fluorobenzyl)-4-(trifluoromethyl)oxetan-2-one (14)**

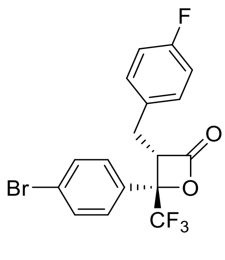


**14**

Following General Procedure D, 3-(4-fluorophenyl)-2-(trimethylsilyl)propanoic acid (96 mg, 0.4 mmol), *N*,*N*-diisopropylethylamine (105 μL, 0.6 mmol), pivaloyl chloride (66 μL, 0.6 mmol) and MTBE (2 mL) for 15 mins, followed by 1-(4-bromophenyl)-2,2,2-trifluoroethan-1-one (50 mg, 0.2 mmol), (2*S*,3*R*)−HyperBTM (3 mg, 10 μmol) and *N*,*N*-diisopropylethylamine (35 μL, 0.2 mmol) for 18 h gave, after purification by Biotage® Isolera™ 4 [SNAP KP-Sil 10 g, 36 mL min$^{-1}$, petrol : $Et_2O$ (98:2 4 CV, 98:2 to 90:10 10 CV)], the title compound (55 mg, 68%) as a colourless oil.

$[\alpha]_D^{20}$ −7.6 (*c*, 0.4, $CHCl_3$).

**IR** $\nu_{max}$ (film) 1850 (C=O), 1171 (C−O).

**Chiral HPLC analysis**, Chiralpak AD-H (99.5:0.5 hexane:IPA, flow rate 1.0 mLmin$^{-1}$, 211 nm, 30 °C), **major diastereoisomer**: $t_R$ (major): 8.6 min, $t_R$ (minor): 9.4 min, 98:2 er; **minor diastereoisomer**: $t_R$ (minor): 13.0 min, $t_R$ (minor): 13.9 min, 85:15 er.

**$^1$H NMR** (500 MHz, $CDCl_3$) **major diastereoisomer**: $\delta_H$ 2.72 (2H, d, *J* 8.2, C(3)HC$H_2$), 4.42 (1H, t, *J* 8.3, C(3)*H*), 6.98 – 7.07 (4H, m, C(3)HCH$_2$ArC(3,5)*H*, C(4)ArC(2,6)*H*), 7.28 – 7.32 (2H, m, C(3)HCH$_2$ArC(2,6)*H*), 7.62 – 7.64 (2H, m, C(4)ArC(3,5)*H*); **minor diastereoisomer**: $\delta_H$ 3.28 – 3.51 (2H, m, C(3)HC$H_2$), 4.17 (1H, t, *J* 7.9, C(3)*H*), 7.06 – 7.09 (2H, m, C(3)HCH$_2$ArC(3,5)*H*), 7.12 – 7.14 (2H, m, C(4)ArC(2,6)*H*), 7.32 – 7.33 (2H, m, C(3)HCH$_2$ArC(2,6)*H*), 7.55 – 7.58 (2H, m, C(4)ArC(3,5)*H*).

**$^{19}$F NMR** (471 MHz, $CDCl_3$) **major diastereoisomer**: $\delta_F$ −77.8 (C$F_3$), −114.9 (m, Ar*F*); **minor diastereoisomer**: $\delta_F$ −73.7 (C$F_3$), −114.7 (m, Ar*F*).

**$^{13}$C{$^1$H} NMR** (126 MHz, $CDCl_3$) **major diastereoisomer**: $\delta_C$ 31.0 (C(3)H*C*H$_2$Ar), 57.4 (*C*(3)H), 79.0 (q, *J* 33.1, *C*(4)), 115.7 (d, *J* 21.5, C(3)HCH$_2$Ar*C*(3,5)H), 123.4 (q, *J* 283.0, *C*F$_3$), 124.8 (C(4)Ar*C*(4)Br), 127.8 (C(4)Ar*C*(1)), 128.3 (C(4)Ar*C*(2,6)H), 130.1 (d, *J* 8.0, C(3)HCH$_2$Ar*C*(2,6)H), 130.6 (d, *J* 3.2, C(3)HCH$_2$Ar*C*(1)), 132.2 (C(4)Ar*C*(3,5)H), 162.0 (d, *J* 246.7, C(3)HCH$_2$Ar*C*(4)F), 166.3 (C(2)); **minor diastereoisomer**: 30.2 (C(3)H*C*H$_2$Ar), 63.1 (*C*(3)H), 116.0 (d, *J* 21.4, C(3)HCH$_2$Ar*C*(3,5)H), 123.2 (q, *J* 283.6, *C*F$_3$), 124.6 (C(4)Ar*C*(4)Br), 127.8 (C(4)Ar*C*(2,6)H), 130.4 (d, *J* 8.0, C(3)HCH$_2$Ar*C*(2,6)H), 131.7 (C(4)Ar*C*(1)), 131.9 (d, *J* 3.2, C(3)HCH$_2$Ar*C*(1)), 132.1 (C(4)Ar*C*(3,5)H), 162.1 (d, *J* 246.6, C(3)CH$_2$Ar*C*(4)F), 166.3 (C(2)).

**HRMS** (ESI$^+$) $C_{17}H_{12}O_3BrF_4$ [M+OH]$^-$ found 418.9909, requires 418.9911 (−0.5 ppm).

**(3*S*,4*S*)-4-(4-Bromophenyl)-3-(naphthalen-2-ylmethyl)-4-(trifluoromethyl)oxetan-2-one (15)**

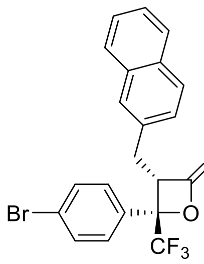


**15**

Following General Procedure D, 3-(naphthalen-2-yl)-2-(trimethylsilyl)propanoic acid (114 mg, 0.4 mmol), *N*,*N*-diisopropylethylamine (105 µL, 0.6 mmol), pivaloyl chloride (66 µL, 0.6 mmol) and MTBE (2 mL) for 15 mins, followed by 1-(4-bromophenyl)-2,2,2-trifluoroethan-1-one (50 mg, 0.2 mmol), (2*S*,3*R*)−HyperBTM (3 mg, 10 µmol) and *N*,*N*-diisopropylethylamine (35 µL, 0.2 mmol) for 16 h gave, after purification by Biotage® Isolera™ 4 [SNAP KP-Sil 10 g, 36 mL min$^{-1}$, petrol : $Et_2O$ (98:2 4 CV, 98:2 to 94:6 30 CV)], the title compound (57 mg, 65%) as a mixture of diastereoisomers in the ratio of 89:11 as a white solid.

**mp** 48 – 50 °C.

$[\alpha]_D^{20}$ +41.1 (*c*, 0.5, $CHCl_3$).

**Chiral HPLC analysis**, Chiralcel OD-H (98:2 hexane:IPA, flow rate 1.0 mLmin$^{-1}$, 211 nm, 30 °C), **major diastereoisomer**: $t_R$ (minor): 26.2 min, $t_R$ (major): 32.0 min, 99:1 er; **minor diastereoisomer**: $t_R$ (major): 23.7 min, $t_R$ (minor): 29.9 min, 89:11 er.

**IR** $\nu_{max}$ (film) 1856 (C=O), 1177 (C−O).

**$^1$H NMR** (400 MHz, $CDCl_3$) **major diastereoisomer**: $\delta_H$ 2.88 (1H, dd, *J* 15.1, 8.1, C(3)HC$H_A$$H_B$), 2.99 (1H, dd, *J* 15.2, 8.1, C(3)HC$H_A$$H_B$), 4.61 (1H, t, *J* 8.1, C(3)*H*), 7.20 – 7.23 (1H, m, Np(*H*)), 7.28 – 7.30 (2H, m, C(4)ArC(2,6)*H*), 7.44 (1H, s, Np(*H*)), 7.48 – 7.55 (2H, m, Np(*H*)), 7.59 – 7.61 (2H, m, C(4)ArC(3,5)*H*), 7.76 – 7.91 (3H, m, Np(*H*)); **minor diastereoisomer**: $\delta_H$ 3.50 (1H, dd, *J* 14.1, 7.8, C(3)HC$H_A$$H_B$), 3.67 (1H, dd, *J* 14.9, 7.6, C(3)HC$H_A$$H_B$), 4.34 (1H, t, *J* 7.8, C(3)*H*), 7.11 – 7.13 (2H, m, C(4)ArC(2,6)*H*).

**$^{19}$F NMR** (376 MHz, $CDCl_3$) **major diastereoisomer**: $\delta_F$ −77.7(s, C*F*$_3$); **minor diastereoisomer**: $\delta_F$ − 73.6.

**$^{13}$C{$^1$H} NMR** (126 MHz, $CDCl_3$) **major diastereoisomer**: $\delta_C$ 31.8 (C(3)H*C*H$_2$), 57.3 (*C*(3)H), 79.2 (q, *J* 33.3, *C*(4)), 123.5 (q, *J* 282.9, *C*F$_3$), 124.8 (C(4)Ar*C*(4)Br), 126.1 (Np*C*H), 126.3 (Np*C*H), 126.5 (Np*C*H), 127.3 (Np*C*H), 127.6 (Np*C*H), 127.7 (Np*C*H), 127.9 (Ar*C*), 128.4 (C(4)Ar*C*(2,6)H), 128.7

(Np*C*H), 132.1 (C(4)Ar*C*(3,5)H), 132.3 (Np*C*), 132.5 (C(4)Ar*C*(1)), 133.3 (Np*C*), 166.5 (*C*O); **minor diastereoisomer**: $\delta_C$ 31.1 (C(3)H*C*$H_2$), 63.0 (*C*(3)H), 123.3 (q, *J* 281.7, *C*$F_3$), 124.5 (C(4)Ar*C*(4)Br), 126.2 (Np*C*H), 126.4 (Np*C*H), 126.6 (Np*C*H), 127.7 (Np*C*H), 127.8 (Np*C*H), 128.9 (Np*C*H), 131.7 (Np*C*), 132.0 (C(4)Ar*C*(3,5)H), 132.6 (C(4)Ar*C*(1)), 133.5 (Np*C*), 166.5 (*C*O).

**HRMS** (ESI$^+$) $C_{21}H_{15}BrF_3O_3$ [M+OH]$^-$ found 451.0167, requires 451.0162 (+1.1 ppm).

**(3*S*,4*S*)-4-(4-Bromophenyl)-3-phenyl-4-(trifluoromethyl)oxetan-2-one (16)**

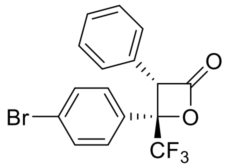


**16**

Following General Procedure D, 2-phenyl-2-(trimethylsilyl)acetic acid (84 mg, 0.4 mmol), *N*,*N*-diisopropylethylamine (105 μL, 0.6 mmol), pivaloyl chloride (66 μL, 0.6 mmol) and MTBE (2 mL) for 15 mins, followed by 1-(4-bromophenyl)-2,2,2-trifluoroethan-1-one (50 mg, 0.2 mmol), (2*S*,3*R*)−HyperBTM (3 mg, 10.0 μmol) and *N*,*N*-diisopropylethylamine (35 μL, 0.2 mmol) for 16 h gave, after purification by Biotage® Isolera™ 4 [SNAP KP-Sil 10 g, 36 mL min$^{-1}$, petrol : $Et_2O$ (98:2 4 CV, 98:2 to 94:6 30 CV)], the title compound (39 mg, 53%) as a colourless oil with spectroscopic data in accordance with the literature.[15]

**Chiral HPLC analysis**, Chiralcel OD-H (97:3 hexane:IPA, flow rate 1.0 mLmin$^{-1}$, 211 nm, 40 °C), **major diastereoisomer**: $t_R$ (minor): 5.0 min, $t_R$ (major): 6.6 min, 96:4 er; **minor diastereoisomer**: $t_R$ (major): 8.8 min, $t_R$ (minor): 9.8 min, 95:5 er.

**$^1$H NMR** (400 MHz, CDCl3) **major diastereoisomer**: $\delta_H$: 5.44 (1H, s, C(3)*H*), 6.91 – 6.93 (2H, m, PhC(2,6)*H*), 6.99 (2H, app d, *J* 8.2, (C(4)ArC(2,6)H), 7.23 – 7.31 (3H, m, PhC(3,4,5)*H*), 7.36 – 7.40 (2H, m, C(4)ArC(3,5)*H*); **minor diastereoisomer**: (selected signals) $\delta_H$: 5.31 (1H, s, C(3)*H*), 7.48 – 7.49 (5H, m, Ph*H*), 7.54 (2H, app d, *J* 8.4, Ar*H*), 7.69 – 7.71 (2H, m, Ar*H*).

**$^{19}$F NMR** (377 MHz, $CDCl_3$) **major diastereoisomer**: $\delta_F$ −78.5 (s, C*F*$_3$); **minor diastereoisomer**: $\delta_F$: −73.3 (C*F*$_3$).

**(*S*)-4-(4-Bromophenyl)-4-(perfluoroethyl)oxetan-2-one (17)**

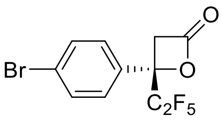


**17**

Following General Procedure D, 2-(trimethylsilyl) acetic acid (105 mg, 0.8 mmol), *N*,*N*-diisopropylethylamine (210 µL, 1.2 mmol), pivaloyl chloride (132 µL, 1.2 mmol) and MTBE (4.0 mL) for 15 mins, followed by 1-(4-bromophenyl)-2,2,3,3,3-pentafluoropropan-1-one (121 mg, 0.4 mmol), (2*S*,3*R*)−HyperBTM (6 mg, 20.0 µmol) and *N*,*N*-diisopropylethylamine (71 µL, 0.4 mmol) for 16 h gave, after purification by Biotage® Isolera™ 4 [SNAP KP-Sil 25 g, 36 mL $min^{-1}$, petrol : $Et_2O$ (98:2 4 CV, 98:2 to 96:4 30 CV)], the title compound (108 mg, 79%) as a colourless waxy solid.

$[\alpha]_D^{20}$ −24.8 (*c* 1.1, $CHCl_3$).

**Chiral HPLC analysis**, Chiralpak IB (99.3:0.7 hexane:IPA, flow rate 1.0 $mLmin^{-1}$, 254 nm, 30 °C), $t_R$ (minor): 8.1 min, $t_R$ (major): 9.1 min, 93:7 er.

**IR** $\nu_{max}$ (film) 1854 (C=O), 1157 (C−O).

**$^1$H NMR** (400 MHz, $CDCl_3$) $\delta_H$ 3.71 – 3.76 (1H, dd, *J* 16.3, 2.3, C(3)$H_AH_B$), 4.26 (1H, d, *J* 16.5, C(3)$H_AH_B$), 7.34 – 7.36 (2H, m, ArC(2,6)*H*), 7.63 – 7.65 (2H, m, ArC(3,5)*H*).

**$^{19}$F NMR** (376 MHz, $CDCl_3$) $\delta_F$ −125.6 (d, *J* 283.2, C$F_2$), −122.4 (d, *J* 275.6, C$F_2$), −78.9 (C$F_3$).

**$^{13}$C{$^1$H} NMR** (126 MHz, $CDCl_3$) $\delta_C$ 48.0 (*C*(3)$H_2$), 75.0 (q, *J* 37.8, *C*(4)), 118.0 (q, *J* 288.1, *C*$F_2$), 118.3 ((q, *J* 288.0, *C*$F_3$), 124.8 (Ar*C*(4)Br), 128.3 (Ar*C*(2,6)H), 130.8 (Ar*C*(1)), 132.1 (Ar*C*(3,5)H), 162.9 (*C*(2)).

**HRMS** ($ESI^+$) $C_{10}H_7O_3BrF_3$ $[M+OH]^-$ found 360.9502, requires 360.9504 (−0.1 ppm)

**(*S*)-4-(Perfluoroethyl)-4-(4-(trifluoromethyl)phenyl)oxetan-2-one (18)**

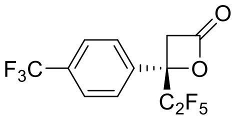


**18**

Following General Procedure D, 2-(trimethylsilyl) acetic acid (105 mg, 0.8 mmol), *N*,*N*-diisopropylethylamine (210 µL, 1.2 mmol), pivaloyl chloride (132 µL, 1.2 mmol) and MTBE (4.0 mL) for 15 mins, followed by 2,2,3,3,3-pentafluoro-1-(4-(trifluoromethyl)phenyl)propan-1-one (117 mg,

0.4 mmol), (2*S*,3*R*)−HyperBTM (6 mg, 20.0 μmol) and *N*,*N*-diisopropylethylamine (71 μL, 0.4 mmol) for 16 h gave, after purification by Biotage® Isolera™ 4 [SNAP KP-Sil 25 g, 36 mLmin$^{-1}$, petrol : $Et_2O$ (98:2 4 CV, 98:2 to 96:4 30 CV)], the title compound (73 mg, 54%) as a colourless oil.

$[\alpha]_D^{20}$ −32.9 (*c*, 2.0, $CHCl_3$).

**Chiral HPLC analysis,** Chiralpak AS-H (99.5:0.5 hexane:IPA, flow rate 1.0 mLmin$^{-1}$, 211 nm, 30 °C), $t_R$ (minor): 5.4 min, $t_R$ (major): 8.0 min, 87:13 er.

**IR** $\nu_{max}$ (film) 1854 (C=O), 1217 (C−O).

**$^1$H NMR** (400 MHz, $CDCl_3$) $\delta_H$ 3.75 – 3.80 (1H, dd, *J* 16.6, 2.6, C(3)$H_AH_B$), 4.32 (1H, d, *J* 16.5, C(3)$H_AH_B$), 7.62 – 7.64 (2H, m, ArC(2,6)*H*), 7.76 – 7.79 (2H, m, ArC(3,5)*H*).

**$^{19}$F NMR** (376 MHz, $CDCl_3$) $\delta_F$ −125.3 (d, *J* 280.0, C$F_2$), −122.2 (d, *J* 281.9, C$F_2$), −78.9 (C$F_3$), 63.1 (ArC$F_3$).

**$^{13}$C{$^1$H} NMR** (126 MHz, $CDCl_3$) $\delta_C$ 48.2 (*C*(3)$H_2$), 74.7 (dd, *J* 28.9, 24.2, *C*(4)), 110.2 – 121.7 (m, *C*$F_2$*C*$F_3$), 124.6 (Ar*C*(4)$CF_3$), 125.8 – 125.9 (m, Ar*C*(3,5)H), 127.3 (m, Ar*C*(2,6)H), 132.4 (q, *J* 32.8, Ar*C*F3), 135.7 (Ar*C*(1)), 162.6 (*C*(2)).

**HRMS** (ESI$^+$) $C_{12}H_7O_3F_8$ [M+OH]$^-$ found 351.0269, requires 351.0273 (−1.1 ppm).

**(*S*)-4-(4-Fluorophenyl)-4-(perfluoroethyl)oxetan-2-one (19)**

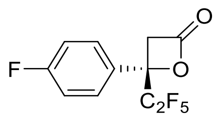


**19**

Following General Procedure D, 2-(trimethylsilyl) acetic acid (105 mg, 0.8 mmol), *N*,*N*-diisopropylethylamine (210 μL, 1.2 mmol), pivaloyl chloride (132 μL, 1.2 mmol) and MTBE (4.0 mL) for 15 mins, followed by 2,2,3,3,3-pentafluoro-1-(4-fluorophenyl)propan-1-one (97 mg, 0.4 mmol), (2*S*,3*R*)−HyperBTM (6 mg, 20.0 μmol) and *N*,*N*-diisopropylethylamine (71 μL, 0.4 mmol) for 16 h gave, after purification by Biotage® Isolera™ 4 [SNAP KP-Sil 25 g, 36 mL min$^{-1}$, petrol : $Et_2O$ (98:2 4 CV, 98:2 to 96:4 40 CV)], the title compound (87 mg, 77%) as a pale yellow oil.

$[\alpha]_D^{20}$ −35.1 (*c*, 2.2, $CHCl_3$).

**Chiral HPLC analysis**, Chiralpak IB (99.3:0.7 hexane:IPA, flow rate 1.0 mLmin$^{-1}$, 254 nm, 30 °C), $t_R$ (minor): 6.6 min, $t_R$ (major): 7.2 min, 92:8 er.

**IR** $\nu_{max}$ (film) 1854 (C=O), 1209 (C−O).

**$^1$H NMR** (400 MHz, $CDCl_3$) $\delta_H$ 3.73 – 3.77 (1H, dd, *J* 16.6, 2.6, C(3)$H_AH_B$), 4.25 (1H, d, *J* 16.5, C(3)$H_AH_B$), 7.16 – 7.22 (2H, m, ArC(3,5)*H*), 7.45 – 7.49 (2H, m, ArC(2,6)*H*).

**$^{19}$F NMR** (376 MHz, $CDCl_3$) $\delta_F$ −126.2 (d, *J* 280.6, C$F_2$), −122.5 (d, *J* 278.0, C$F_2$), −110.0 (Ar*F*), −79.0 (C$F_3$).

**$^{13}$C{$^1$H} NMR** (126 MHz, $CDCl_3$) $\delta_C$ 47.9 (*C*(3)$H_2$), 74.9 (dd, *J* 29.0, 22.7, *C*(4)), 110.9 – 121.7 (m, *C*$F_2$*C*$F_3$), 116.0 (d, *J* 21.8, Ar*C*(3,5)H), 127.7 (Ar*C*(1)), 128.9 (d, *J* 8.6, Ar*C*(2,6)H), 162.8 (d, *J* 69.9, Ar*C*(4)F), 164.6 (*C*(2)).

**HRMS** (ESI$^+$) $C_{11}H_7O_3F_6$ [M+OH]$^-$ found 301.0302, requires 301.0305 (−0.9 ppm).

**(*S*)-4-(3-Methoxyphenyl)-4-(perfluoroethyl)oxetan-2-one (20)**

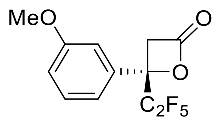


**20**

Following General Procedure D, 2-(trimethylsilyl) acetic acid (105 mg, 0.8 mmol), *N*,*N*-diisopropylethylamine (210 µL, 1.2 mmol), pivaloyl chloride (132 µL, 1.2 mmol) and MTBE (4.0 mL) for 15 mins, followed by 2,2,3,3,3-pentafluoro-1-(3-methoxyphenyl)propan-1-one (102 mg, 0.4 mmol), (2*S*,3*R*)−HyperBTM (6 mg, 20.0 µmol) and *N*,*N*-diisopropylethylamine (71 µL, 0.4 mmol) for 16 h gave, after purification by Biotage® Isolera™ 4 [SNAP KP-Sil 25 g, 36 mL min$^{-1}$, petrol : $Et_2O$ (98:2 4 CV, 98:2 to 96:4 40 CV)], the title compound (114 mg, 96%) as a colourless oil.

$[\alpha]_D^{20}$ −30.6 (*c*, 3.7, $CHCl_3$).

**Chiral HPLC analysis**, Chiralpak IB (99.3:0.7 hexane:IPA, flow rate 1.0 mLmin$^{-1}$, 254 nm, 30 °C), $t_R$ (minor): 6.9 min, $t_R$ (major): 8.3 min, 88:12 er.

**IR** $\nu_{max}$ (film) 1850 (C=O), 1211 (C−O).

**$^1$H NMR** (400 MHz, $CDCl_3$) $\delta_H$ 3.74 – 3.79 (1H, dd, *J* 16.4, 2.4, C(3)$H_AH_B$), 3.86 (3H, s, PhOC$H_3$), 4.23 (1H, d, *J* 16.5, C(3)$H_AH_B$), 7.01 – 7.04 (3H, m, ArC(2,4,6)*H*), 7.38 – 7.42 (1H, m, ArC(5)*H*). **$^{19}$F NMR** (376 MHz, $CDCl_3$) $\delta_F$ −125.4 (d, *J* 276.8, C$F_2$), −122.2 (d, *J* 279.7, C$F_2$), −79.1 (C$F_3$). **$^{13}$C{$^1$H} NMR** (126 MHz, $CDCl_3$) $\delta_C$ 47.9 (*C*(3)$H_2$), 55.4 (O*C*$H_3$), 75.1 (dd, *J* 28.4, 23.1, *C*(4)), 110.1 – 122.1 (m, *C*$F_2$*C*$F_3$), 112.5 (Ar*C*(4)H), 115.5 (Ar*C*(2)H), 118.8 (Ar*C*(6)H), 130.0 (Ar*C*(5)H), 133.2 (Ar*C*(1)),

159.7 (Ar*C*(3)$OCH_3$), 163.6 (*C*(2)).

**HRMS** (ESI$^+$) $C_{12}H_{10}O_4F_5$ [M+OH]$^-$ found 313.0501, requires 313.0505 (−1.2 ppm).

**(*S*)-4-(Perfluoroethyl)-4-(m-tolyl)oxetan-2-one (21)**

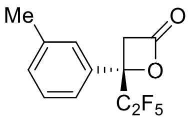

**21**

Following General Procedure D, 2-(trimethylsilyl) acetic acid (105 mg, 0.8 mmol), *N*,*N*-diisopropylethylamine (210 µL, 1.2 mmol), pivaloyl chloride (132 µL, 1.2 mmol) and MTBE (4.0 mL) for 15 mins, followed by 2,2,3,3,3-pentafluoro-1-(m-tolyl)propan-1-one (96 mg, 0.4 mmol), (2*S*,3*R*)−HyperBTM (6 mg, 20.0 µmol) and *N*,*N*-diisopropylethylamine (71 µL, 0.4 mmol) for 16 h gave, after purification by Biotage® Isolera™ 4 [SNAP KP-Sil 25 g, 36 mL min$^{-1}$, petrol : $Et_2O$ (98:2 4 CV, 98:2 to 96:4 40 CV)], the title compound (92 mg, 82%) as a colorless oil.

$[\alpha]_D^{20}$ -35.3 (*c*, 2.3, $CHCl_3$).

**Chiral HPLC analysis**, Chiralpak IB (99.3:0.7 hexane:IPA, flow rate 1.0 mLmin$^{-1}$, 254 nm, 30 °C), $t_R$ (minor): 4.9 min, $t_R$ (major): 5.6 min, 88:12 er**.**

**IR** $\nu_{max}$ (film) 1852 (C=O), 1206 (C−O).

**$^1$H NMR** (500 MHz, $CDCl_3$) $\delta_H$ 2.43 (3H, s, Ph$CH_3$), 3.74 – 3.78 (1H, dd, *J* 12.8, 2.3, C(3)$H_AH_B$), 4.23 (1H, d, *J* 16.5, C(3)$H_AH_B$), 7.25 – 7.30 (3H, m, ArC(2,4,6)*H*), 7.35 – 7.39 (1H, m, ArC(5)*H*). **$^{19}$F NMR** (376 MHz, $CDCl_3$) $\delta_F$ − 125.9 – −125.2 (d, *J* 278.4, C*F*$_2$), −122.8 – −122.0 (d, *J* 280.0, C*F*$_2$), −79.0 (s, C*F*$_3$).

**$^{13}$C{$^1$H} NMR** (126 MHz, $CDCl_3$) $\delta_C$ 21.4 (Ar$CH_3$), 47.8 (*C*(3)$H_2$), 75.3 (dd, *J* 28.5, 23.5, *C*(4)), 110.5 – 119.8 (m, *C*F$_2$*C*F$_3$), 123.8 (Ar*C*(6)H), 127.2 (Ar*C*(4)H), 128.6 (Ar*C*(5)H), 130.8 (Ar*C*(2)H), 131.7 (Ar*C*(3)$CH_3$), 131.7 (Ar*C*(1)), 163.7 (*C*(2)).

**HRMS** (ESI$^+$) $C_{14}H_{10}O_3F_9$ [M+OH]$^-$ found 297.0553, requires 297.0556 (−1.0 ppm).

**(*S*)-4-(Perfluoroethyl)-4-(2-(trifluoromethyl)phenyl)oxetan-2-one (22)**

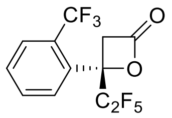

**22**

Following General Procedure D, 2-(trimethylsilyl) acetic acid (105 mg, 0.8 mmol), *N*,*N*-diisopropylethylamine (210 µL, 1.2 mmol), pivaloyl chloride (132 µL, 1.2 mmol) and MTBE (4.0 mL) for 15 mins, followed by 2,2,3,3,3-pentafluoro-1-(2-(trifluoromethyl)phenyl)propan-1-one (117 mg, 0.4 mmol), (2*S*,3*R*)−HyperBTM (6 mg, 20.0 µmol) and *N*,*N*-diisopropylethylamine (71 µL, 0.4 mmol) for 16 h gave, after purification by Biotage® Isolera™ 4 [SNAP KP-Sil 25 g, 36 mL $min^{-1}$, petrol : $Et_2O$ (98:2 4 CV, 98:2 to 96:4 40 CV)], the title compound (122 mg, 92%) as a colourless oil.

$[\alpha]_D^{20}$ −41.5 (*c*, 1.9, $CHCl_3$).

**Chiral HPLC analysis**, Chiralpak IB (99.3:0.7 hexane:IPA, flow rate 1.0 $mLmin^{-1}$, 254 nm, 30 °C), $t_R$ (minor): 5.1 min, $t_R$ (major): 58 min, 88:12 er.

**IR** $\nu_{max}$ (film) 1869 (C=O), 1207 (C−O).

**$^1$H NMR** (500 MHz, $CDCl_3$) $\delta_H$ 3.96 (1H, d, *J* 17.2, C(3)$H_AH_B$), 4.35 (1H, d, *J* 17.4, C(3)$H_AH_B$), 7.66 – 7.73 (2H, m, ArC(4,6)*H*), 7.81 – 7.83 (2H, m, ArC(3,5)*H*).

**$^{19}$F NMR** (376 MHz, $CDCl_3$) $\delta_F$ −119.9 (d, *J* 282.1, C*F*$_2$), − 118.5 (d, *J* 278.0, C*F*$_2$), −79.1 (C*F*$_3$), −56.3 (ArC*F*$_3$).

**$^{13}$C{$^1$H} NMR** (126 MHz, $CDCl_3$) $\delta_C$ 48.7 (*C*(3)$H_2$), 75.5 (dd, *J* 34.2, 24.5, *C*(4)), 112.0 – 119.7 (m, *C*$F_2$*C*$F_3$), 112.5 (Ar*C*$F_3$), 124.7 (Ar*C*(2)$CF_3$), 128.5 (t, *J* 4.9, Ar*C*(6)H), 129.4 (Ar*C*(1)), 129.9 (Ar*C*(3)H), 130.8 (Ar*C*(4)H), 132.1 (Ar*C*(5)H), 163.1 (*C*(2)).

**HRMS** (ESI$^+$) $C_{12}H_7O_3F_8$ $[M+OH]^-$ found 351.0273, requires 351.0273 (0.0 ppm).

**(*S*)-4-(Perfluorobutyl)-4-phenyloxetan-2-one (23)**

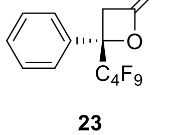

**23**

Following General Procedure D, 2-(trimethylsilyl) acetic acid (105 mg, 0.8 mmol), *N*,*N*-diisopropylethylamine (210 µL, 1.2 mmol), pivaloyl chloride (132 µL, 1.2 mmol) and MTBE (4.0 mL)

for 15 mins, followed by perfluorobutyl-1-phenyl-1-one (130 mg, 0.4 mmol), (2*S*,3*R*)−HyperBTM (6 mg, 20.0 μmol) and *N*,*N*-diisopropylethylamine (71 μL, 0.4 mmol) for 16 h gave, after purification by Biotage® Isolera™ 4 [SNAP KP-Sil 25 g, 36 mL $min^{-1}$, petrol : $Et_2O$ (98:2 4 CV, 98:2 to 96:4 40 CV)], the title compound (114 mg, 78%) as a colourless oil.

$[\alpha]_D^{20}$ −25.2 (*c*, 3.2, $CHCl_3$).

**Chiral HPLC analysis**, Chiralpak IB (99.3:0.7 hexane:IPA, flow rate 1.0 $mLmin^{-1}$, 254 nm, 30 °C), $t_R$ (minor): 5.1 min, $t_R$ (major): 6.0 min, 89:11 er**.**

**IR** $\nu_{max}$ (film) 1859 (C=O), 1202 (C−O).

**$^1$H NMR** (500 MHz, $CDCl_3$) $\delta_H$ 3.79 (1H, dd, *J* 16.6, 2.8, C(3)$H_AH_B$), 4.25 (1H, d, *J* 16.6, C(3)$H_AH_B$), 7.50 (5H, m, Ph*H*).

**$^{19}$F NMR** (376 MHz, $CDCl_3$) $\delta_F$ −127.2 – −125.2 (m, C$F_2$), −122.8 – −121.0 (m, C$F_2$), −119.5 – −118.0 (m, C$F_2$), −80.9 (m, C$F_3$).

**$^{13}$C{$^1$H} NMR** (126 MHz, $CDCl_3$) $\delta_C$ 48.3 (*C*(3)$H_2$), 76.2 (dd, *J* 29.1, 23.7, *C*(4)), 108.0 – 120.6 (m, *C*$F_2$*C*$F_2$*C*$F_2$*C*$F_3$), 126.9 (Ph*C*(2,6)H), 128.7 (Ph*C*(3,5)H), 130.1 (Ph*C*(4)H), 131.8 (Ph*C*(1)), 163.6 (*C*(2)).

**HRMS** (ESI$^+$) $C_{13}H_8O_3F_9$ [M+OH]$^-$ found 383.0337, requires 383.0335 (+0.5 ppm).

**(*S*)-4-(4-Bromophenyl)-4-(perfluorobutyl)oxetan-2-one (24)**

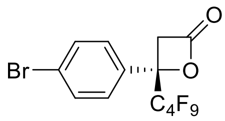


**24**

Following General Procedure D, 2-(trimethylsilyl) acetic acid (105 mg, 0.8 mmol), *N*,*N*-diisopropylethylamine (210 μL, 1.2 mmol), pivaloyl chloride (132 μL, 1.2 mmol) and MTBE (4.0 mL) for 15 mins, followed by 1-(4-bromophenyl)-perfluorobutyl-1-one (161 mg, 0.4 mmol), (2*S*,3*R*)−HyperBTM (6 mg, 20.0 μmol) and *N*,*N*-diisopropylethylamine (71 μL, 0.4 mmol) for 16 h gave, after purification by Biotage® Isolera™ 4 [SNAP KP-Sil 25 g, 36 mL $min^{-1}$, petrol : $Et_2O$ (98:2 4 CV, 98:2 to 96:4 30 CV)], the title compound (162 mg, 91%) as a white solid.

**mp** 64 – 66 °C.

$[\alpha]_D^{20}$ −15.2 (*c* 1.6, $CHCl_3$).

**Chiral HPLC analysis**, Chiralpak IB (99.3:0.7 hexane:IPA, flow rate 1.0 mLmin$^{-1}$, 254 nm, 30 °C), $t_R$ (minor): 7.2 min, $t_R$ (major): 8.5 min, 86:14 er.

**IR** $\nu_{max}$ (film) 1856 (C=O), 1217 (C−O).

**$^1$H NMR** (400 MHz, $CDCl_3$) $\delta_H$ 3.72 – 3.77 (1H, dd, *J* 16.7, 2.5, C(3)$H_AH_B$), 4.26 (1H, d, *J* 16.8, C(3)$H_AH_B$), 7.36 – 7.38 (2H, m, ArC(2,6)*H*), 7.62 – 7.66 (2H, m, ArC(3,5)*H*).

**$^{19}$F NMR** (376 MHz, $CDCl_3$) $\delta_F$ − 127.3 – −125.2 (m, C$F_2$), −122.7 – −120.9 (m, C$F_2$), −119.2 – −117.9 (m, C$F_2$), −80.9 – −80.8 (m, C$F_3$).

**$^{13}$C{$^1$H} NMR** (126 MHz, $CDCl_3$) $\delta_C$ 48.4 (*C*(3)$H_2$), 81.0 (dd, *J* 33.1, 22.4, *C*(4)), 114.4 − 118.6 (m, *C*$F_2$*C*$F_2$*C*$F_2$*C*$F_3$), 124.8 (Ar*C*(4)Br), 128.5 (Ar*C*(2,6)H), 130.8 (Ar*C*(1)), 132.1 (Ar*C*(3,5)H), 163.0 (*C*(2)).

**HRMS** (ESI$^+$) $C_{13}H_7O_3BrF_9$ $[M+OH]^-$ found 460.9436, requires 460.9440 (−1.0 ppm).

**(3*S*,4*S*)-4-(4-Methoxyphenyl)-3-methyl-4-(trifluoromethyl)oxetan-2-one (25)**

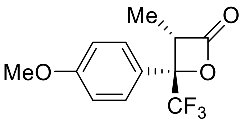


**25**

Following General Procedure D, 2-(trimethylsilyl)propanoic acid (117 mg, 0.8 mmol), *N*,*N*-diisopropylethylamine (210 µL, 1.2 mmol), pivaloyl chloride (132 µL, 1.2 mmol) and MTBE (4.0 mL) for 15 mins, followed by 2,2,2-trifluoro-1-(4-methoxyphenyl)ethan-1-one (82 mg, 0.4 mmol), (2*S*,3*R*)−HyperBTM (6 mg, 20.0 µmol) and *N*,*N*-diisopropylethylamine (71 µL, 0.4 mmol) for 18 h gave, after purification by Biotage® Isolera™ 4 [SNAP KP-Sil 25 g, 36 mL min$^{-1}$, petrol : $Et_2O$ (98:2 4 CV, 98:2 to 90:10 40 CV)], the title compound (68 mg, 65%) as a colourless oil.

$[\alpha]_D^{20}$ −70.3 (*c*, 0.5, $CHCl_3$).

**Chiral HPLC analysis**, Chiralpak AS-H (99.5:0.5 hexane:IPA, flow rate 1.0 mLmin$^{-1}$, 211 nm, 30 °C), $t_R$ (minor): 5.1 min, $t_R$ (major): 6.1 min, >99:1 er.

**IR** $\nu_{max}$ (film) 1844 (C=O), 1173 (C−O).

**$^1$H NMR** (500 MHz, $CDCl_3$) $\delta_H$ 1.13 (3H, d, *J* 7.8, C(3)HC$H_3$), 3.87 (3H, s, OC$H_3$), 4.18 (1H, q, *J* 7.7, C(3)*H*), 7.00 – 7.02 (2H, m, ArC(3,5)*H*), 7.36 – 7.38 (2H, m, ArC(2,6)*H*).

**$^{19}$F NMR** (471 MHz, $CDCl_3$) $\delta_F$ −78.7 (C$F_3$).

**$^{13}C\{^1H\}$ NMR** (126 MHz, $CDCl_3$) $\delta_C$ 10.7 (C(3)H*C*$H_3$), 51.7 (*C*(3)), 55.4 (O*C*$H_3$), 79.2 (q, *J* 32.7, *C*(4)), 114.3 (Ar*C*(3,5)H), 120.7 (Ar*C*(1)), 123.8 (q, *J* 282.8, *C*$F_3$), 128.0 (Ar*C*(2,6)H), 160.6 (Ar*C*(4)O$CH_3$), 168.3 (*C*(2)).

**HRMS** (ESI$^+$) $C_{12}H_{12}O_3F_3$ $[M+H]^+$ found 261.0732, requires 261.0733 (−0.4 ppm).

**(3*S*,4*S*)-4-(3,4-Dimethoxyphenyl)-3-methyl-4-(trifluoromethyl)oxetan-2-one (26)**

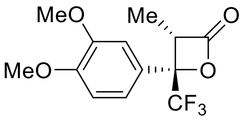


**26**

Following General Procedure D, 2-(trimethylsilyl)propanoic acid (117 mg, 0.8 mmol), *N*,*N*-diisopropylethylamine (210 µL, 1.2 mmol), pivaloyl chloride (132 µL, 1.2 mmol) and MTBE (4.0 mL) for 15 mins, followed by 1-(3,4-dimethoxyphenyl)-2,2,2-trifluoroethan-1-one (94 mg, 0.4 mmol), (2*S*,3*R*)−HyperBTM (6 mg, 20.0 µmol) and *N*,*N*-diisopropylethylamine (71 µL, 0.4 mmol) for 18 h gave, after purification by Biotage® Isolera™ 4 [SNAP KP-Sil 25 g, 36 mL min$^{-1}$, petrol : $Et_2O$ (98:2 4 CV, 98:2 to 80:20 40 CV)], the title compound (86 mg, 74%) as a colourless oil.

$[\alpha]_D^{20}$ −56.7 (*c*, 0.2, $CHCl_3$).

**Chiral HPLC analysis**, Chiralpak AS-H (99.5:0.5 hexane:IPA, flow rate 1.0 mLmin$^{-1}$, 211 nm, 30 °C), $t_R$ (minor): 7.0 min, $t_R$ (major): 8.1 min, >99:1 er.

**IR** $\nu_{max}$ (film) 1844 (C=O), 1169 (C−O).

**$^1H$ NMR** (500 MHz, $CDCl_3$) $\delta_H$ 1.14 (3H, d, *J* 7.7, C(3)HC*H*$_3$), 3.92 (3H, s, ArC(3)OC*H*$_3$), 3.94 (3H, s, ArC(4)OC*H*$_3$), 4.18 (1H, q, *J* 7.7, C(3)*H*), 6.93 – 6.94 (1H, m, ArC(5)*H*), 6.95 – 7.00 (2H, m, ArC(2,6)*H*).

**$^{19}F$ NMR** (471 MHz, $CDCl_3$) $\delta_F$ −78.5 (C*F*$_3$).

**$^{13}C\{^1H\}$ NMR** (126 MHz, $CDCl_3$) $\delta_C$ 10.7 (C*C*$H_3$), 51.8 (*C*(3)H), 56.0 (O*C*$H_3$), 56.1 (O*C*$H_3$), 79.2 (q, *J* 33.2, *C*(4)), 109.5 (Ar*C*(2)H), 111.1 (Ar*C*(5)H), 119.4 (Ar*C*(6)H), 121.0 (Ar*C*(1)), 123.8 (q, *J* 282.1, *C*$F_3$), 149.2 (Ar*C*(4)O$CH_3$), 151.0 (Ar*C*(3)O$CH_3$), 168.2 (*C*(2)).

**HRMS** (ESI$^+$) $C_{13}H_{14}O_4F_3$ $[M+H]^+$ found 291.0835, requires 291.0839 (−1.4 ppm).

**(3*S*,4*S*)-3-Methyl-4-(p-tolyl)-4-(trifluoromethyl)oxetan-2-one (27)**

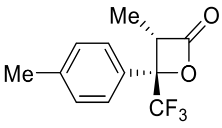

**27**

Following General Procedure D, 2-(trimethylsilyl)propanoic acid (117 mg, 0.8 mmol), *N*,*N*-diisopropylethylamine (210 µL, 1.2 mmol), pivaloyl chloride (132 µL, 1.2 mmol) and MTBE (4.0 mL) for 15 mins, followed by 2,2,2-trifluoro-1-(p-tolyl)ethan-1-one (75 mg, 0.4 mmol), (2*S*,3*R*)−HyperBTM (6 mg, 20.0 µmol) and *N*,*N*-diisopropylethylamine (71 µL, 0.4 mmol) for 18 h gave, after purification by Biotage® Isolera™ 4 [SNAP KP-Sil 25 g, 36 mL min$^{-1}$, petrol : $Et_2O$ (98:2 4 CV, 98:2 to 92:8 40 CV)], the title compound (58 mg, 59%) as a colourless oil.

$[\alpha]_D^{20}$ −91.9 (*c*, 0.3, $CHCl_3$).

**Chiral HPLC analysis**, Chiralcel OJ-H (99.8:0.2 hexane:IPA, flow rate 1.0 mLmin$^{-1}$, 211 nm, 30 °C), $t_R$ (minor): 6.2 min, $t_R$ (major): 7.7 min, >99:1 er.

**IR** $\nu_{max}$ (film) 1846 (C=O), 1175 (C−O).

**$^1$H NMR** (500 MHz, $CDCl_3$) $\delta_H$ 1.13 (3H, d, *J* 7.7, C(3)HC*H*$_3$), 2.42 (3H, s, ArC*H*$_3$), 4.19 (1H, q, *J* 7.7, C(3)*H*), 7.30 – 7.35 (4H, m, Ar*H*).

**$^{19}$F NMR** (471 MHz, $CDCl_3$) $\delta_F$ −78.5 (C*F*$_3$).

**$^{13}$C{$^1$H} NMR** (126 MHz, $CDCl_3$) $\delta_C$ 10.7 (C(3)H*C*H$_3$), 21.2 (Ar*C*H$_3$), 51.7 (*C*(3)H), 79.3 (q, *J* 32.5, *C*(4)), 123.8 (q, *J* 282.0, *C*F$_3$), 125.8 (Ar*C*(4)CH$_3$), 126.5 (Ar*C*(2,6)H), 129.5 (Ar*C*(3,5)H), 140.0 (Ar*C*(1)), 168.3 (*C*(2)).

**HRMS** (ESI$^+$) $C_{12}H_{11}O_2F_3Na$ [M+Na]$^+$ found 267.0601, requires 267.0603 (−0.7 ppm).

**(3*S*,4*S*)-4-(3-Bromophenyl)-3-methyl-4-(trifluoromethyl)oxetan-2-one (28)**

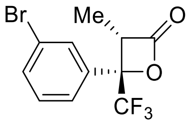

**28**

Following General Procedure D, 2-(trimethylsilyl)propanoic acid (117 mg, 0.8 mmol), *N*,*N*-diisopropylethylamine (210 µL, 1.2 mmol), pivaloyl chloride (132 µL, 1.2 mmol) and MTBE (4.0 mL) for 15 mins, followed by 1-(3-bromophenyl)-2,2,2-trifluoroethan-1-one (101 mg, 0.4 mmol),

(2*S*,3*R*)−HyperBTM (6 mg, 20.0 μmol) and *N*,*N*-diisopropylethylamine (71 μL, 0.4 mmol) for 18 h gave, after purification by Biotage® Isolera™ 4 [SNAP KP-Sil 25 g, 36 mL min$^{-1}$, petrol : $Et_2O$ (98:2 4 CV, 98:2 to 96:4 40 CV)], the title compound (97 mg, 78%) as a colourless oil.

$[\alpha]_D^{20}$ −56.7 (*c*, 0.5, $CHCl_3$).

**Chiral HPLC analysis**, Chiralcel OJ-H (99.8:0.2 hexane:IPA, flow rate 1.0 mLmin$^{-1}$, 211 nm, 30 °C), $t_R$ (minor): 8.8 min, $t_R$ (major): 11.0 min, >99:1 er.

**IR** $\nu_{max}$ (film) 1850 (C=O), 1178 (C−O).

**$^1$H NMR** (500 MHz, $CDCl_3$) $\delta_H$ 1.15 (3H, d, *J* 7.8, C(3)HC*H*$_3$), 4.23 (1H, q, *J* 7.7, C(3)*H*), 7.39 – 7.41 (2H, m, ArC(2,5)*H*), 7.61 – 7.66 (2H, m, ArC(3,6)*H*).

**$^{19}$F NMR** (376 MHz, $CDCl_3$) $\delta_F$ −78.2 (C*F*$_3$).

**$^{13}$C{$^1$H} NMR** (126 MHz, $CDCl_3$) $\delta_C$ 10.8 (C(3)H*C*H$_3$), 52.1 (*C*(3)H), 78.6 (q, *J* 32.8, *C*(4)), 123.2 (Ar*C*(3)Br), 123.5 (q, *J* 282.9, *C*F$_3$), 125.3 (Ar*C*(6)H), 129.8 (Ar*C*(4)H), 130.4 (Ar*C*(5)H), 131.1 (Ar*C*(1)), 133.2 (Ar*C*(2)H), 167.4 (*C*(2)).

**HRMS** (ESI$^+$) $C_{11}H_9BrO_3F_3$ [M+OH]$^-$ found 324.9692, requires 324.9693 (−0.3 ppm).

**(3*S*,4*S*)-4-(4-Bromophenyl)-3-methyl-4-(perfluoroethyl)oxetan-2-one (29)**

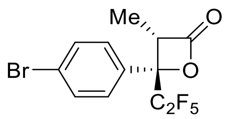


**29**

Following General Procedure D, 2-(trimethylsilyl)propanoic acid (117 mg, 0.8 mmol), *N*,*N*-diisopropylethylamine (210 μL, 1.2 mmol), pivaloyl chloride (132 μL, 1.2 mmol) and MTBE (4.0 mL) for 15 mins, followed by 1-(4-bromophenyl)-2,2,3,3,3-pentafluoropropan-1-one (121 mg, 0.4 mmol), (2*S*,3*R*)−HyperBTM (6 mg, 20.0 μmol) and *N*,*N*-diisopropylethylamine (71 μL, 0.4 mmol) for 18 h gave, after purification by Biotage® Isolera™ 4 [SNAP KP-Sil 25 g, 36 mL min$^{-1}$, petrol : $Et_2O$ (98:2 4 CV, 98:2 to 96:4 30 CV)], the title compound (84 mg, 58%) as a colourless oil.

$[\alpha]_D^{20}$ −57.4 (*c* 1.7, $CHCl_3$).

**Chiral HPLC analysis**, Chiralpak IB (99.9:0.1 hexane:IPA, flow rate 1.0 mLmin$^{-1}$, 254 nm, 30 °C), $t_R$ (major): 5.9 min, >99:1 er.

**IR** $\nu_{max}$ (film) 1861 (C=O), 1204 (C−O).

**$^{1}$H NMR** (400 MHz, $CDCl_3$) $\delta_H$ 1.14 (3H, d, *J* 7.8, C(3)HC*H*$_3$), 4.40 (1H, q, *J* 7.7, C(3)*H*), 7.30 – 7.33 (2H, m, ArC(2,6)*H*), 7.64 – 7.66 (2H, m, ArC(3,5)*H*).

**$^{19}$F NMR** (376 MHz, $CDCl_3$) $\delta_F$ −124.5 (d, *J* 281.6, C*F*$_2$), −121.2 (d, *J* 281.6, C*F*$_2$), −79.1 (C*F*$_3$).

**$^{13}$C{$^{1}$H} NMR** (126 MHz, $CDCl_3$) $\delta_C$ 10.8 (C(3)H*C*H$_3$), 52.4 (d, *J* 4.4, *C*(3)H), 78.4 (dd, *J* 30.6, 23.0, *C*(4)), 118.6 (q, *J* 288.2, *C*F$_2$), 118.7 ((q, *J* 288.1, *C*F$_3$), 124.6 (Ar*C*(4)Br), 128.0 (Ar*C*(2,6)H), 128.3 (Ar*C*(1)), 132.2 (Ar*C*(3,5)H), 167.5 (*C*(2)).

**HRMS** (ESI$^+$) $C_{12}H_9O_3BrF_5$ $[M+OH]^-$ found 374.9660, requires 374.9661 (0.0 ppm).

**(3*S*,4*S*)-3-Methyl-4-(perfluoroethyl)-4-(4-(trifluoromethyl)phenyl)oxetan-2-one (30)**

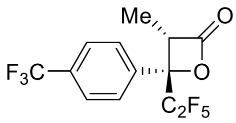

**30**

Following General Procedure D, 2-(trimethylsilyl)propanoic acid (117 mg, 0.8 mmol), *N*,*N*-diisopropylethylamine (210 µL, 1.2 mmol), pivaloyl chloride (132 µL, 1.2 mmol) and MTBE (4.0 mL) for 15 mins, followed by 2,2,3,3,3-pentafluoro-1-(4-(trifluoromethyl)phenyl)propan-1-one (117 mg, 0.4 mmol), (2*S*,3*R*)−HyperBTM (6 mg, 20.0 µmol) and *N*,*N*-diisopropylethylamine (71 µL, 0.4 mmol) for 18 h gave, after purification by Biotage® Isolera™ 4 [SNAP KP-Sil 25 g, 36 mL min$^{-1}$, petrol : $Et_2O$ (98:2 4 CV, 98:2 to 97:3 30 CV)], the title compound (73 mg, 53%) as a colourless oil.

$[\alpha]_D^{20}$ −66.1 (*c*, 2.1, $CHCl_3$).

**Chiral HPLC analysis**, Chiralpak AS-H (99.9:0.1 hexane:IPA, flow rate 1.0 mLmin$^{-1}$, 254 nm, 30 °C), $t_R$ (minor): 4.1 min, $t_R$ (major): 4.4 min, 98:2 er.

**IR** $\nu_{max}$ (film) 1863 (C=O), 1206 (C−O).

**$^{1}$H NMR** (400 MHz, $CDCl_3$) $\delta_H$ 1.12 (3H, d, *J* 8.0, C(3)HC*H*$_3$), 4.46 (1H, q, *J* 8.0, C(3)*H*), 7.58 – 7.60 (2H, m, C(4)ArC(2,6)*H*), 7.77 – 7.79 (2H, m, C(4)ArC(3,5)*H*).

**$^{19}$F NMR** (376 MHz, $CDCl_3$) $\delta_F$ −124.5 (d, *J* 282.3, C*F*$_2$), −121.0 (d, *J* 284.1, C*F*$_2$), −79.1 (C*F*$_3$), 63.0 (ArC*F*$_3$).

**$^{13}$C{$^{1}$H} NMR** (126 MHz, $CDCl_3$) $\delta_C$ 10.9 (C(3)*C*H$_3$), 52.7 (*C*(3)), 78.6 (dd, *J* 30.0, 23.0, *C*(4)), 110.6 – 119.6 (m, *C*F$_2$*C*F$_3$), 124.6 (Ar*C*(4)CF$_3$), 125.9 (m, Ar*C*(3,5)H), 127.2 (m, Ar*C*(2,6)H), 132.2 (q, *J* 32.0, Ar*C*F3), 133.0 (Ar*C*(1)), 167.2 (*C*(2)).

**HRMS** (ESI$^+$) $C_{13}H_9O_3F_8$ [M+OH]$^-$ found 365.0429, requires 365.0429 (0.0 ppm).

**(3*S*,4*S*)-4-(3-Methoxyphenyl)-3-methyl-4-(perfluoroethyl)oxetan-2-one (31)**

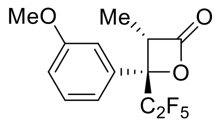

**31**

Following General Procedure D, 2-(trimethylsilyl)propanoic acid (117 mg, 0.8 mmol), *N*,*N*-diisopropylethylamine (210 µL, 1.2 mmol), pivaloyl chloride (132 µL, 1.2 mmol) and MTBE (4.0 mL) for 15 mins, followed by 2,2,3,3,3-pentafluoro-1-(3-methoxyphenyl)propan-1-one (102 mg, 0.4 mmol), (2*S*,3*R*)−HyperBTM (6 mg, 20.0 µmol) and *N*,*N*-diisopropylethylamine (71 µL, 0.4 mmol) for 18 h gave, after purification by Biotage® Isolera™ 4 [SNAP KP-Sil 25 g, 36 mL min$^{-1}$, petrol : $Et_2O$ (98:2 4 CV, 98:2 to 97:3 30 CV)], the title compound (102 mg, 82%) as a colourless oil.

$[\alpha]_D^{20}$ −63.0 (*c*, 3.0, $CHCl_3$).

**Chiral HPLC analysis**, Chiralpak AS-H (99.9:0.1 hexane:IPA, flow rate 1.0 mLmin$^{-1}$, 254 nm, 30 °C), $t_R$ (minor): 4.7 min, $t_R$ (major): 5.3 min, >99:1 er.

**IR** $\nu_{max}$ (film) 1854 (C=O), 1204 (C−O).

**$^1$H NMR** (400 MHz, $CDCl_3$) $\delta_H$ 1.13 (3H, d, *J* 7.6, C(3)HC*H*$_3$), 3.86 (3H, s, ArOC*H*$_3$), 4.37 (1H, q, *J* 8.7, C(3)*H*), 6.98 – 7.02 (3H, m, C(4)ArC(2,4,6)*H*), 7.38 – 7.42 (1H, m, C(4)ArC(5)*H*).

**$^{19}$F NMR** (376 MHz, $CDCl_3$) $\delta_F$ −124.3 (d, *J* 282.3, C*F*$_2$), −121.0 (d, *J* 286.0, C*F*$_2$), −79.2 (C*F*$_3$);

**$^{13}$C{$^1$H} NMR** (126 MHz, $CDCl_3$) $\delta_C$ 10.8 (C(3)H*C*H$_3$), 52.4 (*C*(3)H), 55.4 (O*C*H$_3$), 78.9 (dd, *J* 30.1, 23.5, *C*(4)), 110.8 – 122.0 (m, *C*F$_2$*C*F$_3$), 112.5 (Ar*C*(4)H), 115.1 (Ar*C*(2)H), 118.8 (Ar*C*(6)H), 130.0 (Ar*C*(5)H), 130.3 (Ar*C*(1)), 159.8 (Ar*C*(3)OCH$_3$), 168.1 (*C*(3)).

**HRMS** (ESI$^+$) $C_{13}H_{12}O_4F_5$ [M+OH]$^-$ found 327.0657, requires 327.0661 (−1.2 ppm).

**(3*S*,4*S*)-3-Methyl-4-(perfluoroethyl)-4-(m-tolyl)oxetan-2-one (32)**

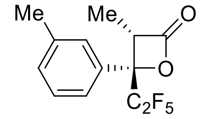

**32**

Following General Procedure D, 2-(trimethylsilyl)propanoic acid (117 mg, 0.8 mmol), *N*,*N*-diisopropylethylamine (210 μL, 1.2 mmol), pivaloyl chloride (132 μL, 1.2 mmol) and MTBE (4.0 mL) for 15 mins, followed by 2,2,3,3,3-pentafluoro-1-(m-tolyl)propan-1-one (96 mg, 0.4 mmol), (2*S*,3*R*)−HyperBTM (6 mg, 20.0 μmol) and *N*,*N*-diisopropylethylamine (71 μL, 0.4 mmol) for 18 h gave, after purification by Biotage® Isolera™ 4 [SNAP KP-Sil 25 g, 36 mL min$^{-1}$, petrol : $Et_2O$ (98:2 4 CV, 98:2 to 96:4 40 CV)], the title compound (56 mg, 48%) as a colourless oil.

$[\alpha]_D^{20}$ −73.7 (*c*, 1.4, $CHCl_3$).

**Chiral HPLC analysis**, Chiralpak AS-H (99.9:0.1 hexane:IPA, flow rate 1.0 mLmin$^{-1}$, 211 nm, 30 °C), $t_R$ (minor): 4.0 min, $t_R$ (major): 4.2 min, >99:1 er**.**

**IR** $\nu_{max}$ (film) 1852 (C=O), 1202 (C−O).

**$^1$H NMR** (400 MHz, $CDCl_3$) $\delta_H$ 1.11 (3H, d, *J* 7.8, C(3)HC*H*$_3$), 2.46 (3H, s, ArC*H*$_3$), 4.37 (1H, q, *J* 7.7, C(3)*H*), 7.19 − 7.30 (3H, m, ArC(2,4,6)*H*), 7.35 − 7.39 (1H, m, ArC(5)*H*).

**$^{19}$F NMR** (376 MHz, $CDCl_3$) $\delta_F$ −124.3 (d, *J* 282.8, C*F*$_2$), −121.2 (d, *J* 282.1, C*F*$_2$), −79.2 (C*F*$_3$);

**$^{13}$C{$^1$H} NMR** (126 MHz, $CDCl_3$) $\delta_C$ 10.8 (C(3)H*C*H$_3$), 21.5 (Ar*C*H$_3$), 52.3 (*C*(3)H), 79.1 (dd, *J* 29.8, 22.6, *C*(4)), 110.9 − 119.8 (m, *C*F$_2$*C*F$_3$), 123.7 (Ar*C*(6)H), 127.0 (Ar*C*(4)H), 128.6 (Ar*C*(5)H), 128.8 (Ar*C*(3)CH$_3$), 130.6 (Ar*C*(2)H), 131.7 (Ar*C*(1)), 168.3 (*C*(2)).

**HRMS** (ESI$^+$) $C_{13}H_{12}O_3F_5$ [M+OH]$^-$ found 311.0712, requires 311.0712 (0.0 ppm).

**(3*S*,4*S*)-3-Methyl-4-(perfluorobutyl)-4-phenyloxetan-2-one (33)**

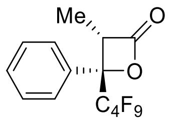


**33**

Following General Procedure D, 2-(trimethylsilyl)propanoic acid (117 mg, 0.8 mmol), *N*,*N*-diisopropylethylamine (210 μL, 1.2 mmol), pivaloyl chloride (132 μL, 1.2 mmol) and MTBE (4.0 mL) for 15 mins, followed by perfluorobutyl-1-phenyl-1-one (130 mg, 0.4 mmol), (2*S*,3*R*)−HyperBTM (6 mg, 20.0 μmol) and *N*,*N*-diisopropylethylamine (71 μL, 0.4 mmol) for 18 h gave, after purification by Biotage® Isolera™ 4 [SNAP KP-Sil 25 g, 36 mL min$^{-1}$, petrol : $Et_2O$ (98:2 4 CV, 98:2 to 96:4 40 CV)], the title compound (96 mg, 63%) as a colourless oil.

$[\alpha]_D^{20}$ −55.7 (*c*, 2.0, $CHCl_3$).

**Chiral HPLC analysis**, Chiralcel OJ-H (99.8:0.2 hexane:IPA, flow rate 1.0 mLmin$^{-1}$, 211 nm, 30 °C), $t_R$ (major): 4.5 min, $t_R$ (minor): 5.0 min, 98:2 er**.**

**IR** $\nu_{max}$ (film) 1850 (C=O), 1202 (C−O).

**$^1$H NMR** (500 MHz, $CDCl_3$) $\delta_H$ 1.10 (3H, d, *J* 7.7, C(3)HC*H*$_3$), 4.40 (1H, q, *J* 7.7, C(3)*H*), 7.46 – 7.51 (5H, m, Ph*H*).

**$^{19}$F NMR** (376 MHz, $CDCl_3$) $\delta_F$ −128.1 – −124.5 (m, C*F*$_2$), −123.1 – −121.4 (m, C*F*$_2$), −118.5 – −115.6 (m, C*F*$_2$), −80.9 (m, C*F*$_3$).

**$^{13}$C{$^1$H} NMR** (126 MHz, $CDCl_3$) $\delta_C$ 10.8 (C(3)H*C*H$_3$), 52.9 (*C*(3)H), 79.9 (dd, *J* 28.2, 20.8, *C*(4)), 108.3 − 118.6 (m, *C*F$_2$*C*F$_2$*C*F$_2$*C*F$_3$), 126.7 (Ph*C*(2,6)H), 128.8 (Ph*C*(3,5)H), 128.8 (Ph*C*(1)), 129.9 (Ph*C*(4)H), 168.2 (*C*(2)).

**HRMS** (ESI$^+$) $C_{14}H_{10}O_3F_9$ [M+OH]$^-$ found 397.0495, requires 397.0492 (+0.7 ppm).

**(3*S*,4*S*)-4-(4-Bromophenyl)-3-methyl-4-(perfluorobutyl)oxetan-2-one (34)**

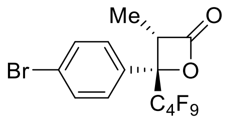


**34**

Following General Procedure D, 2-(trimethylsilyl)propanoic acid (117 mg, 0.8 mmol), *N*,*N*-diisopropylethylamine (210 µL, 1.2 mmol), pivaloyl chloride (132 µL, 1.2 mmol) and MTBE (4.0 mL) for 15 mins, followed by 1-(4-bromophenyl)-perfluorobutyl-1-one (161 mg, 0.4 mmol), (2*S*,3*R*)−HyperBTM (6 mg, 20.0 µmol) and *N*,*N*-diisopropylethylamine (71 µL, 0.4 mmol) for 18 h gave, after purification by Biotage® Isolera™ 4 [SNAP KP-Sil 25 g, 36 mL min$^{-1}$, petrol : $Et_2O$ (98:2 4 CV, 98:2 to 96:4 30 CV)], the title compound (173 mg, 95%) as a colourless oil.

$[\alpha]_D^{20}$ –43.9 (*c*, 2.8, $CHCl_3$).

**Chiral HPLC analysis**, Chiralpak IB (99.9:0.1 hexane:IPA, flow rate 1.0 mLmin$^{-1}$, 254 nm, 30 °C), $t_R$ (major): 5.4 min, >99:1 er.

**IR** $\nu_{max}$ (film) 1855 (C=O), 1206 (C−O).

**$^1$H NMR** (400 MHz, $CDCl_3$) $\delta_H$ 1.11 (1H, d, *J* 7.6, C(3)HC*H*$_3$), 4.41 (1H, q, *J* 8.6, C(3)*H*), 7.32 – 7.34 (2H, m, ArC(2,6)*H*), 7.64 – 7.66 (2H, m, ArC(3,5)*H*).

**$^{19}$F NMR** (376 MHz, $CDCl_3$) $\delta_F$ − 128.1 – −124.5 (m, C*F*$_2$), −122.8 – −121.5 (m, C*F*$_2$), −118.3 –

−115.6 (m, C*F*$_2$), −80.9 – −80.8 (m, C*F*$_3$).

**$^{13}$C{$^1$H} NMR** (126 MHz, CDCl$_3$) $\delta_C$ 10.8 (C(3)H*C*H$_3$), 52.9 (*C*(3)), 79.7 (dd, *J* 33.1, 23.5, *C*(4)), 112.7 – 115.0 (m, *C*F$_2$*C*F$_2$*C*F$_2$*C*F$_3$), 124.6 (Ar*C*(4)Br), 128.0 (Ar*C*(2,6)H), 128.4 (Ar*C*(1)), 132.1 (Ar*C*(3,5)H), 167.6 (*C*(2)).

**HRMS** (ESI$^+$) $C_{14}H_9O_3BrF_9$ [M+OH]$^-$ found 474.9594, requires 474.9597 (−0.6 ppm).

**(3*S*,4*S*)-3-Benzyl-4-(4-bromophenyl)-4-(perfluorobutyl)oxetan-2-one (35)**

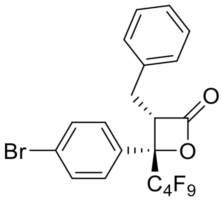


**35**

Following General Procedure D, 3-phenyl-2-(trimethylsilyl)propanoic acid (180 mg, 0.8 mmol), *N*,*N*-diisopropylethylamine (210 µL 1.2 mmol), pivaloyl chloride (132 µL, 1.2 mmol) and MTBE (4.0 mL) for 15 mins, followed by 1-(4-Bromophenyl)-perfluorobutyl-1-one (161 mg, 0.4 mmol), (2*S*,3*R*)−HyperBTM (6 mg, 20.0 µmol) and *N*,*N*-diisopropylethylamine (71 µL, 0.4 mmol) for 20 h gave, after purification by Biotage® Isolera™ 4 [SNAP KP-Sil 25 g, 36 mL min$^{-1}$, petrol : Et$_2$O (98:2 4 CV, 98:2 to 95:5 40 CV)], the title compound (133 mg, 62%) as a colourless oil.

$[\alpha]_D^{20}$ +5.8 (*c* 0.3, CHCl$_3$).

**Chiral HPLC analysis**, Chiralpak IB (99.9:0.1 hexane:IPA, flow rate 1.0 mLmin$^{-1}$, 211 nm, 30 °C), major: $t_R$ (minor): 15.1min, $t_R$ (major): 19.3min, >99:1 er.

**IR** $\nu_{max}$ (film) 1854 (C=O), 1211 (C−O).

**$^1$H NMR** (500 MHz, CDCl$_3$) $\delta_H$ 2.66 – 2.76 (2H, m, C(3)HC*H*$_2$), 4.66 (1H, t, *J* 8.2, C(3)*H*), 7.11 – 7.12 (2H, m, C(4)ArC(2,6)*H*), 7.28 – 7.35 (5H, m, C(3)CH$_2$Ph*H*), 7.60 – 7.62 (2H, m, C(4)ArC(3,5)*H*).

**$^{19}$F NMR** (471 MHz, CDCl$_3$) $\delta_F$ −128.0 – −124.5 (m, C*F*$_2$), −122.7 – −120.9 (m, C*F*$_2$), −117.9 – −114.6 (m, C*F*$_2$), −80.8 (m, C*F*$_3$).

**$^{13}$C{$^1$H} NMR** (126 MHz, CDCl$_3$) $\delta_C$ 31.7 (C(3)H*C*H$_2$Ph), 58.2 (*C*(3)H), 79.8 (dd, *J* 31.5, 23.4, *C*(4)), 108.5 – 120.9 (m, *C*$_4$F$_9$), 124.7 (C(4)Ar*C*(4)Br), 127.4 (C(3)CH$_2$Ph*C*(4)H), 127.8 (C(3)CH$_2$Ph*C*(1)), 128.5 (C(3)CH$_2$Ph*C*(2,6)H), 128.5 (C(4)Ar*C*(2,6)H), 128.8 (C(3)CH$_2$Ph*C*(3,5)H), 132.1 (C(4)Ar*C*(3,5)H), 135.0 (C(4)Ar*C*(1)), 166.5 (C(2)).

**HRMS** (ESI+) $C_{20}H_{13}O_3BrF_9$ [M+OH]$^-$ found 550.9911, requires 550.9910 (+0.2 ppm).

**(3*S*,4*S*)-4-(4-Bromophenyl)-3-(naphthalen-2-ylmethyl)-4-(perfluorobutyl)oxetan-2-one (36)**

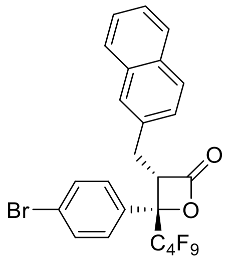

**36**

Following General Procedure D, 3-(naphthalen-2-yl)-2-(trimethylsilyl)propanoic acid (114 mg, 0.4 mmol), *N*,*N*-diisopropylethylamine (105 µL, 0.6 mmol), pivaloyl chloride (66 µL, 0.6 mmol) and MTBE (2 mL) for 15 mins, followed by 1-(4-bromophenyl)-perfluorobutyl-1-one (85 mg, 0.2 mmol), (2*S*,3*R*)−HyperBTM (3 mg, 10.0 µmol) and *N*,*N*-diisopropylethylamine (35 µL, 0.2 mmol) for 16 h gave, after purification by Biotage® Isolera™ 4 [SNAP KP-Sil 10 g, 36 mL min$^{-1}$, petrol : $Et_2O$ (98:2 4 CV, 98:2 to 94:6 30 CV)], the title compound (102mg, 87%) as a mixture of diastereoisomers in the ratio of 91:9, as a colourless oil.

$[\alpha]_D^{20}$ +36.7 (*c*, 0.3, $CHCl_3$).

**Chiral HPLC analysis**, Chiralpak IB (99:1 hexane:IPA, flow rate 1.0 mLmin$^{-1}$, 211 nm, 30 °C), **major diastereoisomer**: $t_R$ (minor): 18.6 min, $t_R$ (major): 27.9 min, 99:1 er; **minor diastereoisomer**: $t_R$ (major): 12.6 min, $t_R$ (minor): 17.4 min, 76:24 er.

**IR** $\nu_{max}$ (film) 1852 (C=O), 1200 (C−O).

**$^1$H NMR** (400 MHz, $CDCl_3$) **major diastereoisomer**: $\delta_H$ 2.84 – 2.94 (2H, m, C(3)HC$H_2$), 4.79 (1H, t, *J* 8.3, C(3)*H*), 7.23 – 7.25 (1H, m, NpC*H*), 7.30 – 7.31 (2H, m, C(4)ArC(2,6)*H*), 7.51 – 7.53 (3H, m, NpC*H*), 7.60 – 7.61 (2H, m, C(4)ArC(3,5)*H*), 7.79 – 7.86 (3H, m, NpC*H*)); **minor diastereoisomer**: $\delta_H$ 3.60 (1H, dd, *J* 14.9, 7.2, C(3)HC$H_AH_B$), 3.72 (1H, dd, *J* 14.9, 8.6, C(3)HC$H_AH_B$), 4.36 (1H, t, *J* 7.5, C(3)*H*), 7.41 – 7.45 (2H, m, Np*H*).

**$^{19}$F NMR** (376 MHz, $CDCl_3$) $\delta_F$ −128.0 – −124.5 (m, C$F_2$), −122.7 – −120.8 (m, C$F_2$), −117.9 – −114.5 (m, C$F_2$), −80.8 (m, C$F_3$).

**$^{13}$C{$^1$H} NMR** (126 MHz, $CDCl_3$) **major diastereoisomer**: $\delta_C$ 31.9 (C(3)$CH_2$), 58.2 (*C*(3)H), 79.9 (dd, *J* 31.5, 23.6 *C*(4)), 108.3 – 120.9 (m, $C_4F_9$), 124.8 (C(4)Ar*C*(4)Br), 126.1 (Np*C*H), 126.4 (Np*C*H),

126.5 (Np*C*H), 127.4 (Np*C*H), 127.6 (Np*C*H), 127.7 (Np*C*H), 127.8 (Np*C*), 128.6 (C(4)Ar*C*(2,6)H), 128.7 (Np*C*H), 132.1 (C(4)Ar*C*(3,5)H), 132.3 (Np*C*), 132.5 (C(4)Ar*C*(1)), 133.3 (Np*C*), 166.5 (*C*O); **minor diastereoisomer**: $\delta_C$ 31.5 (C(3)*C*H$_2$), 66.9 (*C*(3)H), 124.3 (C(4)Ar*C*(4)Br), 126.0 (Np*C*H), 126.5 (Np*C*H), 126.8 (Np*C*H), 127.4 (Np*C*H), 127.6 (Np*C*H), 128.1 (Np*C*H), 128.4 (C(4)Ar*C*(2,6)H), 128.8 (Np*C*H), 129.1 (C(4)Ar*C*(3,5)H), 133.1 (Np*C*), 133.5 (C(4)Ar*C*(1)), 133.6 (Np*C*), 166.4 (*C*O).

**HRMS** (ESI$^+$) $C_{24}H_{15}BrF_9O_3$ [M+OH]$^-$ found 601.0060, requires 601.0066 (−1.0 ppm).

**(*S*,*E*)-4-Styryl-4-(trifluoromethyl)oxetan-2-one (42)**

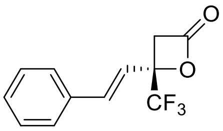


**42**

Following General Procedure D, 2-(trimethylsilyl) acetic acid (66 mg, 0.5 mmol), *N*,*N*-diisopropylethylamine (132 µL, 0.75 mmol), pivaloyl chloride (92 µL, 0.75 mmol) and MTBE (3 mL) for 15 mins, followed by (*E*)-1,1,1-trifluoro-4-phenylbut-3-en-2-one (50 mg, 0.25 mmol), (2*S*,3*R*)−HyperBTM (4 mg, 12.5 µmol) and *N*,*N*-diisopropylethylamine (44 µL, 0.25 mmol) for 16 h gave, after purification by Biotage® Isolera™ 4 [SNAP KP-Sil 25 g, 36 mL min$^{-1}$, petrol : Et$_2$O (98:2 4 CV, 98:2 to 90:10 30 CV)], the title compound (45 mg, 75%) as a bright yellow oil.

$[\alpha]_D^{20}$ −19.3 (*c* 1.4, CHCl$_3$).

**Chiral HPLC analysis**, Chiralpak IB (99.3:0.7 hexane:IPA, flow rate 1.0 mL min$^{-1}$, 254 nm, 30 °C), $t_R$ (major): 16.1 min, $t_R$ (minor): 24.9 min, 92:8 er.

**IR** $\nu_{max}$ (film) 1852 (C=O), 1167 (C−O), 972 (C=C).

**$^1$H NMR** (400 MHz, CDCl$_3$) $\delta_H$ 3.59 – 3.64 (1H, dq, *J* 16.6, 2.2, C(3)*H*$_A$H$_B$), 3.90 (1H, d, *J* 16.4, C(3)H$_A$*H*$_B$), 6.41 (1H, d, *J* 16.0, C*H*=CHPh), 7.02 (1H, d, *J* 16.0, CH=C*H*Ph), 7.31 – 7.57 (5H, m, Ph*H*).

**$^{19}$F NMR** (376 MHz, CDCl$_3$) $\delta_F$ −79.9 (C*F*$_3$).

**$^{13}$C{$^1$H} NMR** (126 MHz, CDCl$_3$) $\delta_C$ 46.0 (*C*(3)H$_2$), 74.2 (q, *J* 34.2, *C*(4)), 117.4 (CH=*C*HPh), 123.3 (q, *J* 281.0, *C*F$_3$), 127.2 (Ph*C*(2,6)H), 128.9 (Ph*C*(3,5)H), 129.5 (Ph*C*(4)H), 134.2 (Ph*C*(1)), 136.8 (*C*H=CHPh), 163.6 (*C*(2)).

**HRMS** (ESI$^+$) $C_{12}H_9O_2F_3Na$ [M+Na]$^+$ found 265.0445, requires 265.0447 (−0.7 ppm).

**(3*S*,4*R*)-3-Methyl-4-((*E*)-styryl)-4-(trifluoromethyl)oxetan-2-one (43)**

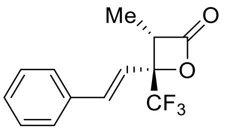


**43**

Following General Procedure D, 2-(trimethylsilyl)propanoic acid (73 mg, 0.5 mmol), *N*,*N*-diisopropylethylamine (132 µL, 0.75 mmol), pivaloyl chloride (92 µL, 0.75 mmol) and MTBE (3 mL) for 15 mins, followed by (*E*)-1,1,1-trifluoro-4-phenylbut-3-en-2-one (50 mg, 0.25 mmol), (2*S*,3*R*)−HyperBTM (4 mg, 12.5 µmol) and *N*,*N*-diisopropylethylamine (44 µL, 0.25 mmol) for 20 h gave, after purification by Biotage® Isolera™ 4 [SNAP KP-Sil 25 g, 36 mL min$^{-1}$, petrol : $Et_2O$ (98:2 4 CV, 98:2 to 95:5 40 CV)], the title compound (48 mg, 75%) as a colourless oil.

$[\alpha]_D^{20}$ −118.4 (*c* 1.1, $CHCl_3$).

**Chiral HPLC analysis**, **major diastereoisomer**: Chiralpak IB (99.5:0.5 hexane:IPA, flow rate 0.7 mLmin$^{-1}$, 254 nm, 30 °C), $t_R$ (minor): 7.3 min, $t_R$ (major): 9.2 min, 99:1 er; **minor diastereoisomer**: Chiralpak IB (99.5:0.5 hexane:IPA, flow rate 0.7 mLmin$^{-1}$, 211 nm, 30 °C), $t_R$ (major): 12.1 min, $t_R$ (minor): 13.9 min, 77:23 er.

**IR** $\nu_{max}$ (film) 1852 (C=O), 1165 (C−O).

**$^1$H NMR** (400 MHz, $CDCl_3$) $\delta_H$ 1.36 (3H, d, *J* 7.7, C(3)HC*H*$_3$), 4.10 (1H, q, *J* 7.7, C(3)*H*), 6.21 (1H, d, *J* 16.0, C*H*=CHPh), 7.05 (1H, d, *J* 16.0, CH=C*H*Ph), 7.38 – 7.44 (3H, m, PhC(3,4,5)*H*), 7.48 – 7.50 (2H, m, PhC(2,6)*H*).

**$^{19}$F NMR** (376 MHz, $CDCl_3$) $\delta_F$ −79.7 (C*F*$_3$).

**$^{13}$C{$^1$H} NMR** (126 MHz, $CDCl_3$) $\delta_C$ 9.7 (C(3)H*C*H$_3$), 52.0 (*C*(3)H), 78.3 (q, *J* 33.2, *C*(4)), 117.4 (CH=*C*HPh), 123.3 (q, *J* 281.0, *C*F$_3$), 127.2 (Ph*C*(2,6)H), 128.9 (Ph*C*(3,5)H), 129.5 (Ph*C*(4)H), 134.2 (Ph*C*(1)), 136.8 (*C*H=CHPh), 163.6 (*C*(2)).

**HRMS** (ESI$^+$) $C_{13}H_{12}O_2F_3$ [M+H]$^+$ found 257.0784, requires 257.0784 (0.0 ppm).

**(3*S*,4*S*)-3-Methyl-4-((*E*)-1-phenylprop-1-en-2-yl)-4-(trifluoromethyl)oxetan-2-one (44)**

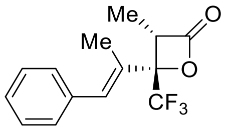

**44**

Following General Procedure D, 2-(trimethylsilyl)propanoic acid (117 mg, 0.8 mmol), *N*,*N*-diisopropylethylamine (210 µL, 1.2 mmol), pivaloyl chloride (132 µL, 1.2 mmol) and MTBE (4.0 mL) for 15 mins, followed by (*E*)-1,1,1-trifluoro-3-methyl-4-phenylbut-3-en-2-one (86 mg, 0.4 mmol), (2*S*,3*R*)−HyperBTM (6 mg, 20.0 µmol) and *N*,*N*-diisopropylethylamine (71 µL, 0.4 mmol) for 16 h gave, after purification by Biotage® Isolera™ 4 [SNAP KP-Sil 25 g, 36 mL min$^{-1}$, petrol : $Et_2O$ (98:2 4 CV, 98:2 to 96:4 40 CV)], the title compound (71 mg, 65%) as a colourless oil.

$[\alpha]_D^{20}$ +32.3 (*c*, 1.1, $CHCl_3$).

**Chiral HPLC analysis**, Chiralcel OJ-H (99.8:0.2 hexane:IPA, flow rate 1.0 mLmin$^{-1}$, 254 nm, 30 °C), $t_R$ (minor): 13.1 min, $t_R$ (major): 17.4 min, >99:1 er.

**IR** $\nu_{max}$ (film) 1846 (C=O), 1171 (C−O).

**$^1$H NMR** (500 MHz, $CDCl_3$) $\delta_H$ 1.41 (3H, d, *J* 7.7, C(3)HC*H*$_3$), 2.00 (3H, s, CH=CC*H*$_3$), 4.07 (1H, q, *J* 7.8, C(3)*H*), 6.94 (1H, s, PhC*H*), 7.32 – 7.36 (3H, m, PHC(2,4,6)*H*), 7.40 – 7.44 (2H, m, PHC(3,5)*H*).

**$^{19}$F NMR** (376 MHz, $CDCl_3$) $\delta_F$ −76.5 (C*F*$_3$).

**$^{13}$C{$^1$H} NMR** (126 MHz, $CDCl_3$) $\delta_C$ 9.7 (CH=C*C*H$_3$), 15.2 (C(3)H*C*H$_3$), 51.5 (*C*(3)H), 80.7 (q, *J* 31.6, *C*(4)), 123.8 (q, *J* 283.6, *C*F$_3$), 125.4 (Ph*C*(1)), 127.8 (Ph*C*(4)H), 128.4 (Ph*C*(2,6)H), 129.2 (Ph*C*(3,5)H), 132.6 (Ph*C*H=C), 135.5 (PhCH=*C*), 168.3 (*C*(2)).

**HRMS** (ESI$^+$) $C_{14}H_{14}O_2F_3$ [M+H]$^+$ found 271.0936, requires 271.0940 (−1.6 ppm).

**(3*R*,4*R*)-3,4-Diphenyl-6-(trifluoromethyl)-3,4-dihydro-2H-pyran-2-one (45)**

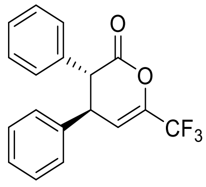

**45**

Following General Procedure D, 2-phenyl-2-(trimethylsilyl)acetic acid (84 mg, 0.4 mmol), *N*,*N*-

diisopropylethylamine (105 μL, 0.6 mmol), pivaloyl chloride (66 μL, 0.6 mmol) and MTBE (2 mL) for 15 mins, followed by (*E*)-1,1,1-trifluoro-4-phenylbut-3-en-2-one (40 mg, 0.2 mmol), (2*S*,3*R*)−HyperBTM (3 mg, 10.0 μmol) and *N*,*N*-diisopropylethylamine (35 μL, 0.2 mmol) for 16 h gave, after purification by Biotage® Isolera™ 4 [SNAP KP-Sil 10 g, 36 mL $min^{-1}$, petrol : $Et_2O$ (98:2 4 CV, 98:2 to 94:6 30 CV)], the title compound (45 mg, 70%) as a white solid in the ratio of 97:3 of diastereoisomer with spectroscopic data in accordance with the literature.[13]

**Chiral HPLC analysis**, Chiralpak AD-H (95:5 hexane:IPA, flow rate 1.0 $mLmin^{-1}$, 211 nm, 30 °C), **major diastereoisomer**: $t_R$ (major): 8.8 min, $t_R$ (minor): 9.6 min, 95:5 er; **minor diastereoisomer**: $t_R$ (minor): 6.5 min, $t_R$ (major): 6.9 min, 93:7 er.

**$^1$H NMR** (500 MHz, $CDCl_3$) **major diastereoisomer**: $\delta_H$ 3.99 (1H, d, *J* 8.8, C(3)*H*), 4.04 – 4.07 (1H, m, C(4)*H*), 6.16 (1H, d, *J* 3.6, C(5)*H*), 7.05 – 7.07 (2H, m, Ph*H*), 7.11 – 7.13 (2H, m, Ph*H*), 7.28 – 7.34 (6H, m, Ph*H*); **minor diastereoisomer**: $\delta_H$ 3.65 – 3.72 (2H, m, C(3)*H*, C(4)*H*), 6.33 (1H, d, *J* 5.9, C(5)*H*), 6.73 – 6.75 (2H, m, Ph*H*), 6.80 – 6.81 (2H, m, Ph*H*), 7.28 – 7.34 (6H, m, Ph*H*).

**$^{19}$F NMR** (471 MHz, $CDCl_3$) **major diastereoisomer**: $\delta_F$ −72.1 (s, C*F*$_3$); **minor diastereoisomer**: $\delta_F$ −72.0 (s, C*F*$_3$).

## 5. Product Derivatizations

**(2*S*,3*S*)-*N*-Benzyl-3-(4-bromophenyl)-4,4,5,5,5-pentafluoro-3-hydroxy-2-methylpentanamide (37)**

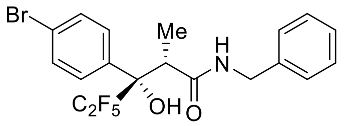


**37**

The β-lactone **29** (35 mg, 0.1 mmol, 1 equiv.) and benzylamine (50 μL, 0.5 mmol, 5 equiv.) were dissolved in dichloromethane (2.0 mL) for 16 h. The organic layer was washed with water (3 × 5 mL), dried over $MgSO_4$, filtered and concentrated under reduced pressure to give title compound as a white solid (36 mg, 83%).

**mp** 108 – 110 °C.

$[\alpha]_D^{20}$ +39.5 (*c*, 1.6, $CHCl_3$).

**Chiral HPLC analysis**, Chiralcel OD-H (95:5 hexane:IPA, flow rate 1.0 mLmin$^{-1}$, 211 nm, 30 °C), $t_R$ (major): 26.5 min, >99:1 er.

**IR** $\nu_{max}$ (film) 3283(O−H), 1628 (C=O).

**$^1$H NMR** (400 MHz, $CDCl_3$) $\delta_H$ 0.95 (3H, d, *J* 7.0, $CH_3$), 2.93 (1H, qd, *J* 7.0, 1.6, $CH_3CH$), 4.51 (2H, d, *J* 5.6, $NHCH_2$), 6.14 (1H, t, *J* 5.6, N*H*), 7.01 – 7.44 (7H, m, Ar*H*), 7.53 – 7.55 (2H, m, C(3)ArC(3,5)*H*).

**$^{19}$F NMR** (376 MHz, $CDCl_3$) $\delta_F$ −119.6 (d, *J* 33.7, $CF_2$), −77.8 ($CF_3$).

**$^{13}$C{$^1$H} NMR** (126 MHz, $CDCl_3$) $\delta_C$ 14.0 ($CH_3$), 41.2 (CO*C*H), 43.9 ($NHCH_2$), 78.1 (t, *J* 21.9, $CC_2F_5$), 113.1 – 122.4 (m, $CF_2CF_3$), 122.9 (Ar*C*(4)Br), 127.9 (Ph*C*(2,4,6)H), 128.0 (Ph*C*(3,5)H), 129.0 (Ar*C*(2,6)H), 131.5 (Ar*C*(3,5)H), 135.1 (Ph*C*(1)), 136.9 (Ar*C*(1)), 175.8 (*C*O).

**HRMS** (ESI$^+$) $C_{19}H_{17}O_2NBrF_5Na$ $[M+Na]^+$ found 488.0252, requires 488.0255 (−0.6 ppm).

**(*S*)-*N*-Benzyl-3-(4-bromophenyl)-4,4,5,5,5-pentafluoro-3-hydroxypentanamide (37b)**

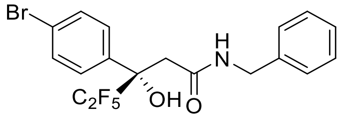


**37b**

The β-lactone **17** (20 mg, 0.06 mmol) and benzylamine (29 μL, 0.29 mmol) were dissolved in dichloromethane (1.5 mL) for 16 h. The organic layer was washed with water (3 × 4 mL), dried over $MgSO_4$, filtered and concentrated under reduced pressure to give title compound as a white solid gave as white solid (23 mg, 88%).

**mp** 102 – 104 °C.

$[\alpha]_D^{20}$ +32.3 (*c*, 1.1, $CHCl_3$).

**Chiral HPLC analysis**, Chiralpak IA (95:5 hexane:IPA, flow rate 1.0 mLmin$^{-1}$, 211 nm, 30 °C), $t_R$ (minor): 12.6 min, $t_R$ (major): 15.1 min, 93:7 er.

**IR** $\nu_{max}$ (film) 3304 (O−H), 1630 (C=O).

**$^1$H NMR** (400 MHz, $CDCl_3$) $\delta_H$ 2.86 (1H, d, *J* 14.7, C(3)$H_AH_B$), 3.13 (1H, d, *J* 14.6, C(3)$H_AH_B$), 4.13 (1H, dd, *J* 14.9, 4.8, NHC$H_AH_B$), 4.51 (1H, dd, *J* 14.9, 6.9, NHC$H_AH_B$), 5.92 (1H, t, *J* 6.5, N*H*), 6.83 − 6.88 (2H, m, ArC(2,6)*H*), 7.28 – 7.30 (3H, m, PhC(2,4,6)*H*), 7.46 – 7.48 (2H, m, PhC(3,5)*H*), 7.52 – 7.55 (2H, m, C(3)ArC(3,5)*H*).

**$^{19}$F NMR** (376 MHz, $CDCl_3$) $\delta_F$ −121.8 (d, *J* 19.0, C$F_2$), −77.8 (C$F_3$).

**$^{13}$C{$^1$H} NMR** (126 MHz, $CDCl_3$) $\delta_C$ 39.4 (CO$CH_2$), 43.4 (NH$CH_2$), 76.3 (t, *J* 24.0, $CC_2F_5$), 111.3 – 122.7 (m, $CF_2CF_3$), 123.2 (Ar*C*(4)Br), 127.3 (Ph*C*(2,6)H), 127.9 (Ph*C*(4)H), 128.4 (Ph*C*(3,5)H), 128.8 (Ar*C*(2,6)H), 131.6 (Ar*C*(3,5)H), 136.6 (Ph*C*(1)), 136.7 (Ar*C*(1)), 170.2 (*C*O).

**HRMS** (ESI$^+$) $C_{18}H_{15}O_2NBrF_5Na$ $[M+Na]^+$ found 474.0088, requires 474.0099 (−2.2 ppm).

**(2*R*,3*S*)-3-(4-Bromophenyl)-perfluorobutyl-2-methylheptane-1,3-diol (38)**

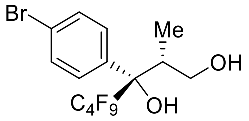


**38**

1 M *i*-$Bu_2AlH$ (DIBAL) solution in hexane (0.8 mL, 0.8 mmol, 2 equiv.) was added dropwise to a solution of a (3*S*,4*S*)-4-(4-Bromophenyl)-3-methyl-4-(perfluorobutyl)oxetan-2-one **34** (184 mg, 0.4 mmol, 1 equiv.) in $CH_2Cl_2$ (4 mL) at −78 °C under an inert atmosphere, and the reaction allowed to stir for 90 min. Saturated aqueous $NH_4Cl$ was added and the mixture was allowed to warm to r.t. The aqueous layer was extracted with $CH_2Cl_2$ (2 × 10 mL) and the combined organic layers dried ($MgSO_4$), filtered and concentrated under reduced pressure to give a crude mixture, after

purification by Biotage® Isolera™ 4 [SNAP KP-Sil 25 g, 36 mL $min^{-1}$, petrol : $Et_2O$ (98:2 4 CV, 98:2 to 85:15 40 CV)], as colourless oil (96 mg, 52%).

$[\alpha]_D^{20}$ −13.2 (*c*, 0.3, $CHCl_3$).

**Chiral HPLC analysis**, Chiralpak AD-H (99.5:0.5 hexane:IPA, flow rate 1.0 $mLmin^{-1}$, 211 nm, 30 °C), $t_R$ (major): 23.8 min, $t_R$ (minor): 26.8 min, >99:1 er.

**IR** $\nu_{max}$ (film) 3418 (OH).

**$^1$H NMR** (400 MHz, $CDCl_3$) $\delta_H$ 0.87 (3H, d, *J* 7.3, C(2)HC$H_3$), 1.96 – 1.98 (1H, m, C(2)*H*), 2.47 – 2.53 (1H, m, C(1)$H_AH_B$), 3.83 (1H, dt, *J* 10.7, 2.6, C(1)$H_AH_B$), 4.48 – 4.53 (1H, m, C(1)O*H*), 5.48 (1H, s, C(3)O*H*), 7.43 – 7.45 (2H, m, ArC(2,6)*H*), 7.51 – 7.54 (2H, m, ArC(3,5)*H*).

**$^{19}$F NMR** (376 MHz, $CDCl_3$) $\delta_F$ −128.0 – −124.0 (m, C$F_2$), −122.4 – −116.1 (m, C$F_2$), −116.7 – −114.5 (m, C$F_3$), −80.8 (m, C$F_3$).

**$^{13}$C{$^1$H} NMR** (126 MHz, $CDCl_3$) $\delta_C$ 12.9 (C$CH_3$), 38.6 (C(2)), 66.4 ($CH_2$), 80.8 (t, *J* 22.8, $CC_4F_9$), 106.5 – 119.9 (m, $CF_2CF_2CF_2CF_3$), 122.4 (Ar*C*(4)Br), 128.1 (Ar*C*(2,6)H), 131.2 (Ar*C*(3,5)H), 136.5 (d, *J* 4.8, Ar*C*(1)).

**HRMS** ($ESI^+$) $C_{14}H_{11}O_2BrF_9$ $[M-H]^-$ found 460.9806, requires 460.9804 (+0.4 ppm).

**Methyl (2*S*,3*S*)-3-(4-bromophenyl)-perfluorobutyl-3-hydroxy-2-methylheptanoate (39)**

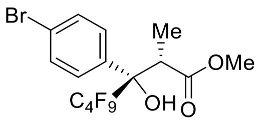


**39**

A freshly prepared solution of 1 M NaOMe in MeOH (2 mL, 2.0 mmol, 4 equiv.) was added to a solution of (3*S*,4*S*)-4-(4-Bromophenyl)-3-methyl-4-(perfluorobutyl)oxetan-2-one **34** (184 mg, 0.4 mmol) in $CH_2Cl_2$ (0.1 M) at −78 °C under an inert atmosphere and allowed to stir for 16 hours at −4 °C gave. Saturated aqueous $NH_4Cl$ was added; the mixture was allowed to warm to r.t., and extracted with $CH_2Cl_2$ (3 × 10 mL). The combined organic layers were dried ($MgSO_4$), filtered and concentrated under reduced pressure to give a residue, after purification by Biotage® Isolera™ 4 [SNAP KP-Sil 25 g, 36 mL $min^{-1}$, petrol : $Et_2O$ (98:2 4 CV, 98:2 to 95:5 40 CV)], as a white solid (169 mg, 86%).

**mp** 68 – 70 °C.

$[\alpha]_D^{20}$ +2.0 (*c*, 0.6, $CHCl_3$).

**Chiral HPLC analysis**, Chiralpak AD-H (99.9:0.1 hexane:IPA, flow rate 1.0 $mLmin^{-1}$, 211 nm, 30 °C), $t_R$ (minor): 4.3 min, $t_R$ (major): 4.8 min, >99:1 er.

**IR** $\nu_{max}$ (film) 3387 (OH), 1715 (CO).

**$^1$H NMR** (500 MHz, $CDCl_3$) $\delta_H$ 0.89 (3H, d, *J* 7.2, C(3)HC$H_3$), 3.27 (1H, dt, *J* 7.2, 2.4, C(3)*H*), 3.85 (3H, s, OC$H_3$), 5.71 (1H, s, O*H*), 7.42 – 7.49 (2H, m, ArC(2,6)*H*), 7.54 – 7.56 (2H, m, ArC(3,5)*H*);

**$^{19}$F NMR** (376 MHz, $CDCl_3$) $\delta_F$ −127.4 – −124.8 (m, C$F_2$), −120.7 – −117.8 (m, C$F_2$), −117.0 – −114.1 (m, C$F_3$), −80.9 (m, C$F_3$).

**$^{13}$C{$^1$H} NMR** (126 MHz, $CDCl_3$) $\delta_C$ 13.0 (CH$CH_3$), 40.8 (*C*H$CH_3$), 52.9 (O$CH_3$), 78.9 (t, *J* 22.2, *C*$C_4F_9$), 106.3 – 119.1 (m, *C*$F_2$*C*$F_2$*C*$F_2$*C*$F_3$), 123.0 (Ar*C*(4)Br), 128.2 (Ar*C*(2,6)H), 131.5 (Ar*C*(3,5)H), 134.1 (d, *J* 4.6, Ar*C*(1)), 177.2 (*C*O).

**HRMS** (ESI$^+$) $C_{15}H_{12}O_3BrF_9Na$ $[M+Na]^+$ found 512.9719, requires 512.9718 (+0.1 ppm).

## 6. Mechanistic Investigations

*General Considerations:* Stock solutions were prepared by adding reagents directly to volumetric glassware. The required stock solution volume was measured using a gas-tight 100 μL microsyringe. 50 μL of Sample were taken throughout the reaction mixture dissolved in 0.3 mL $CDCl_3$ directly and added to the NMR tube for each kinetic experiment. Data were collected at 376 MHz, kinetic experiments (arrays of spectra) were implemented using Bruker Topspin software. The spectra were phase and baseline corrected in MestReNova, each spectrum was processed manually. Aliquots (50 microL) were removed from the reaction mixture for analysis every 60 minutes for first 480 minutes of reaction, the sample for the last measurement was removed at 1440 minutes. Control experiment indicated that the reaction stopped when the reaction mixture dissolved in 0.3 mL $CDCl_3$.

### *6.1 Standard Procedure Kinetic Experiments*

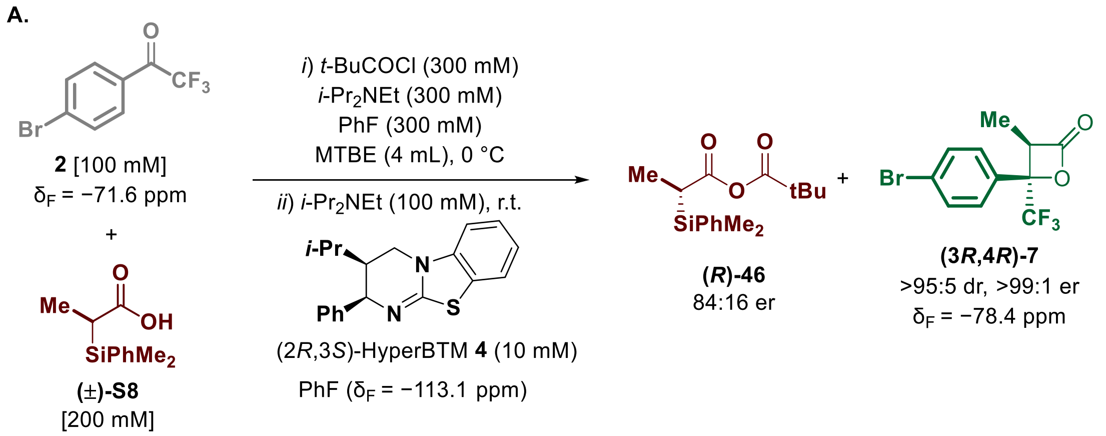


**Scheme S1**. Standard kinetic procedure

The following stock solutions were prepared at the start of the day on which the the experiment was performed:

Stock solution A: 2-(Dimethyl(phenyl)silyl)propanoic acid (±)-**S8** (166.7 mg, 0.8 mmol, 200 mM) and fluorobenzene (113 μL, 1.2 mmol) in 2 mL MTBE.

Stock solution B: 1-(4-Bromophenyl)-2,2,2-trifluoroethan-1-one **2** (101.2 mg, 0.4 mmol, 100 mM), *N*,*N*-diisopropylethylamine (70 μL, 0.4 mmol, 100 mM) in 2 mL MTBE.

Stock solution A (2 mL) was transferred to a flame-dried Schlenk tube under $N_2$, pivaloyl chloride (147 μL, 1.2 mmol, 300 mM) and *N*,*N*-diisopropylethylamine (209 μL, 1.2 mmol, 300 mM) were added at 0 °C and the mixture was stirred for 15 min. Stock solution B was then added to the reaction mixture and an initial $^{19}F\{^1H\}$ spectrum was acquired. (2*R*,3*S*)-HyperBTM **4** (12.3 mg, 0.04 mmol, 10 mM) was added to the reaction mixture and the reaction was stirred for the specified time at r.t..

Parameters for $^{19}F\{^1H\}$ spectra: 260 ppm sweep width (10 to −250 ppm), number scans (ns) = 4, spectral centre (o1p) = −130 ppm, d1 relaxation delay = 25 s.

*Standard Procedure $^{19}F\{^1H\}$ NMR Spectra*

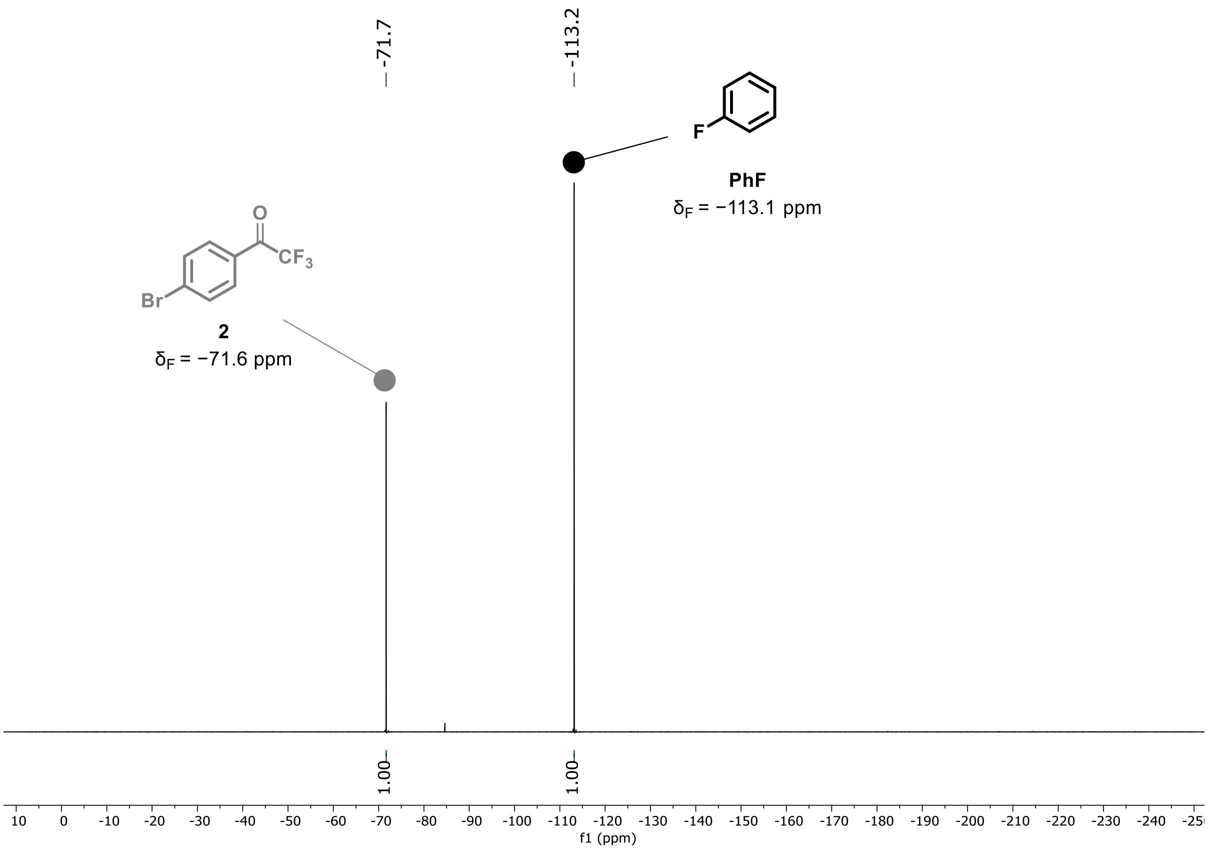


**Figure S1**. Initial $^{19}F\{^1H\}$ spectra (376 MHz) ($CDCl_3$) obtained following the standard procedure for kinetic experiments.

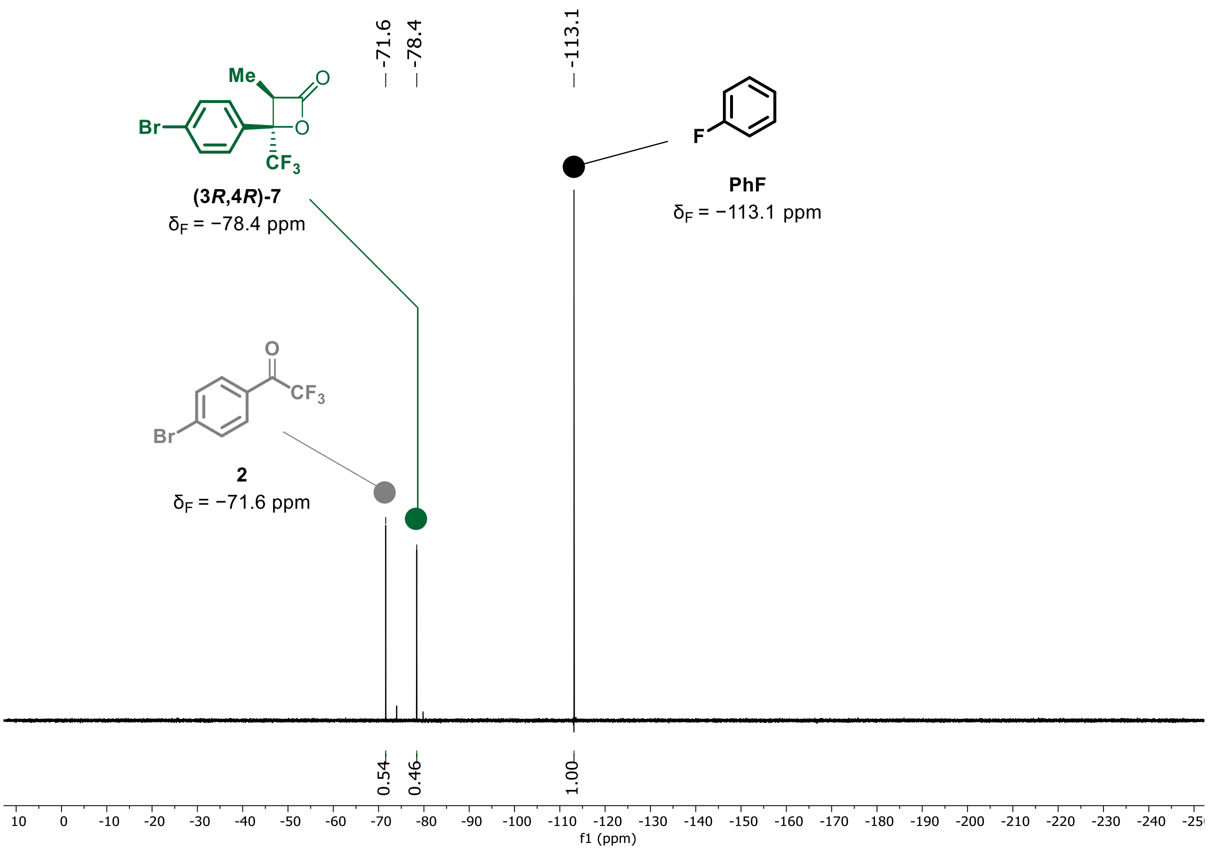


**Figure S2**. $^{19}$F{$^{1}$H} Spectra (376 MHz) ($CDCl_3$) obtained following the standard procedure for kinetic experiments after 480 minutes.

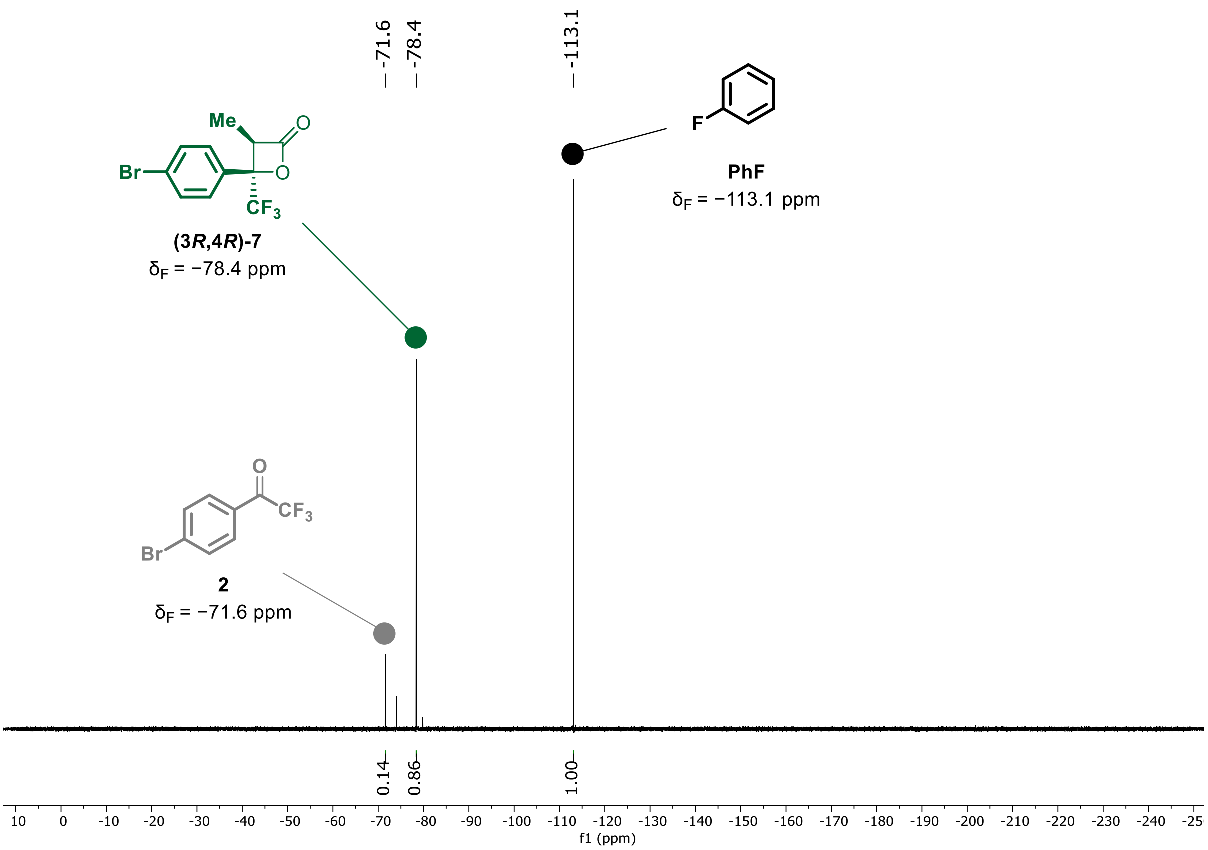


**Figure S3**. $^{19}F\{^1H\}$ Spectra (376 MHz) ($CDCl_3$) obtained following the standard procedure for kinetic experiments after 1440 minutes.

*Concentration data for reaction A (re = repeat):*

| Replicate | **Time** (minutes) | 0 | 60 | 120 | 180 | 240 | 300 | 360 | 420 | 480 | 1440 |
|---|---|---|---|---|---|---|---|---|---|---|---|
| **1** | **2 (mM)** | 100 | 95 | 90 | 84 | 77 | 71 | 65 | 59 | 54 | 14 |
| | **7 (mM)** | 0 | 5 | 10 | 16 | 23 | 29 | 35 | 41 | 46 | 86 |
| **2** | **2 (mM) re1** | 100 | 95 | 91 | 86 | 79 | 72 | 66 | 61 | 56 | 16 |
| | **7 (mM) re1** | 0 | 5 | 9 | 14 | 21 | 27 | 33 | 38 | 43 | 82 |

| | | | | | | | | | | | |
|---|---|---|---|---|---|---|---|---|---|---|---|
| 3 | **2 (mM) re2** | 100 | 95 | 91 | 86 | 78 | 73 | 68 | 62 | 57 | 14 |
| | **7 (mM) re2** | 0 | 5 | 9 | 14 | 22 | 27 | 31 | 36 | 42 | 84 |

*HPLC analysis for reaction A:* The enantioselectivity of β-lactone **7** and anhydride **46** were measure using by HPLC analysis. Aliquots (50 microL) were removed from the reaction mixture for analysis every 60 minutes for first 480 minutes of reaction, the sample for the last measurement was removed at 1440 minutes. The *i*-$Pr_2NEt{\cdot}HCl$ was filtered off and the solvent was removed under reduced pressure. The residue was dissolved *n*-Hexane.

HPLC conditions for **7**: Chiralpak AS-H (99.5:0.5 hexane:IPA, flow rate 1.0 mLmin$^{-1}$, 211 nm, 30 °C), $t_R$ (major): 4.5 min, >99:1 er.

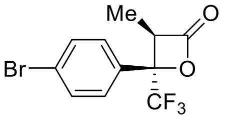


**(3*R*,4*R*)-7**

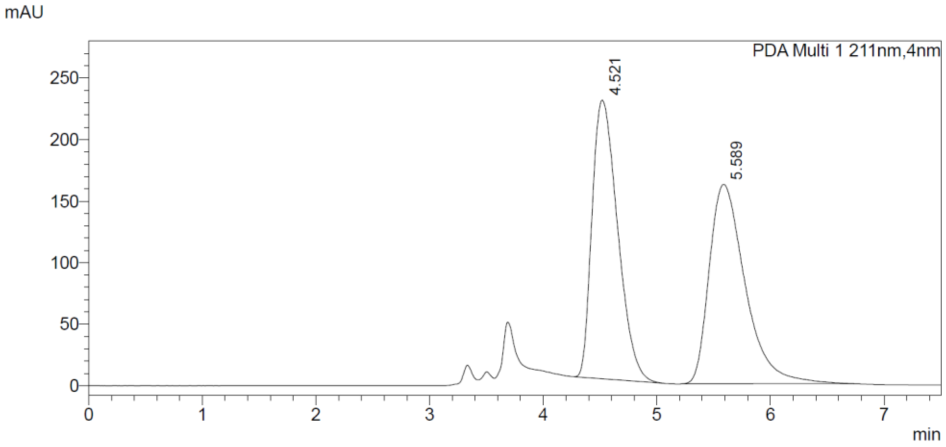


**<Peak Table>**

PDA Ch1 211nm

| Peak# | Ret. Time | Area% |
|---|---|---|
| 1 | 4.521 | 49.787 |
| 2 | 5.589 | 50.213 |
| Total | | 100.000 |

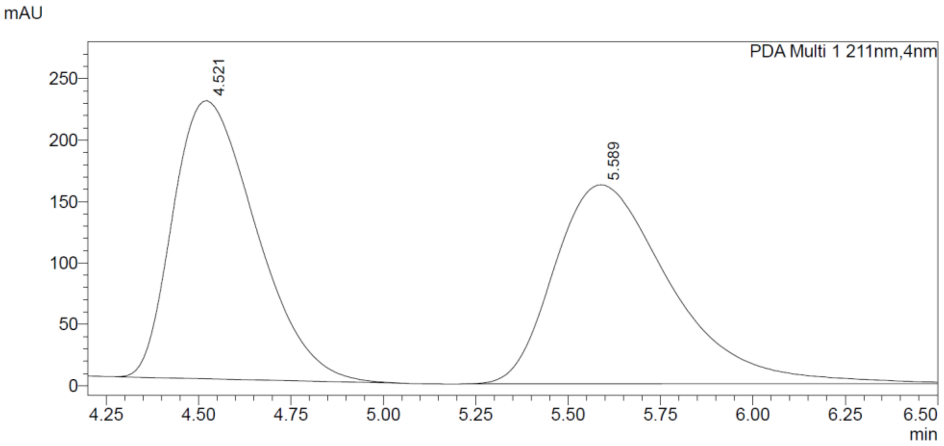


**<Peak Table>**

PDA Ch1 211nm

| Peak# | Ret. Time | Area% |
|---|---|---|
| 1 | 4.521 | 49.787 |
| 2 | 5.589 | 50.213 |
| Total | | 100.000 |

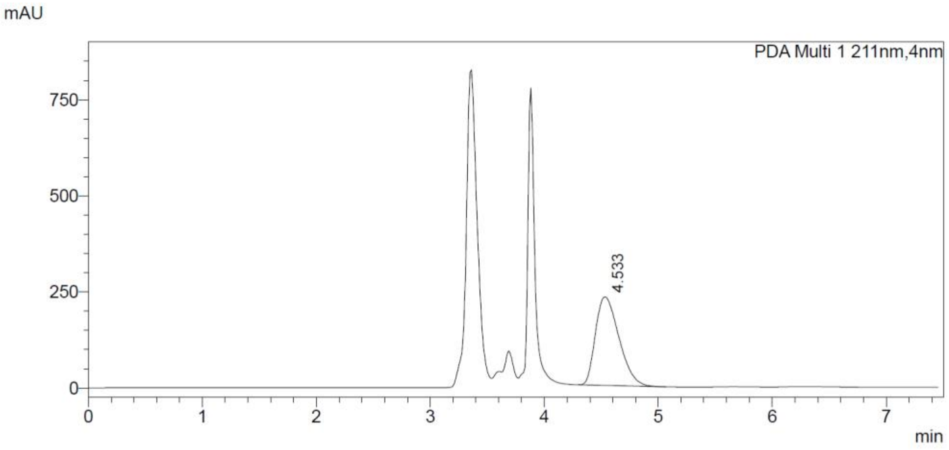


**<Peak Table>**

PDA Ch1 211nm

| Peak# | Ret. Time | Area% |
|---|---|---|
| 1 | 4.533 | 100.000 |
| Total | | 100.000 |

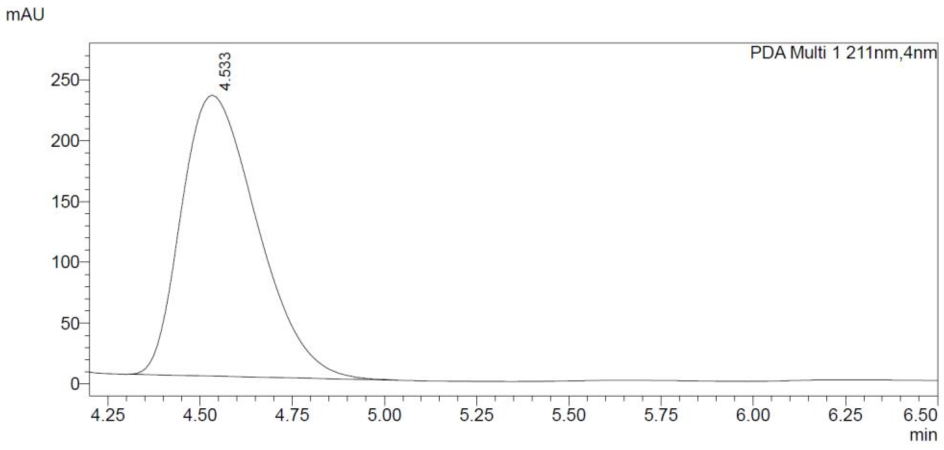


**<Peak Table>**

PDA Ch1 211nm

| Peak# | Ret. Time | Area% |
|---|---|---|
| 1 | 4.533 | 100.000 |
| Total | | 100.000 |

HPLC conditions for **46**: Chiralcel OD-H (99.9:0.1 hexane:IPA, flow rate 1.0 mL min$^{-1}$, 211 nm, 30 °C).

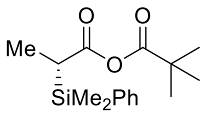


**46**

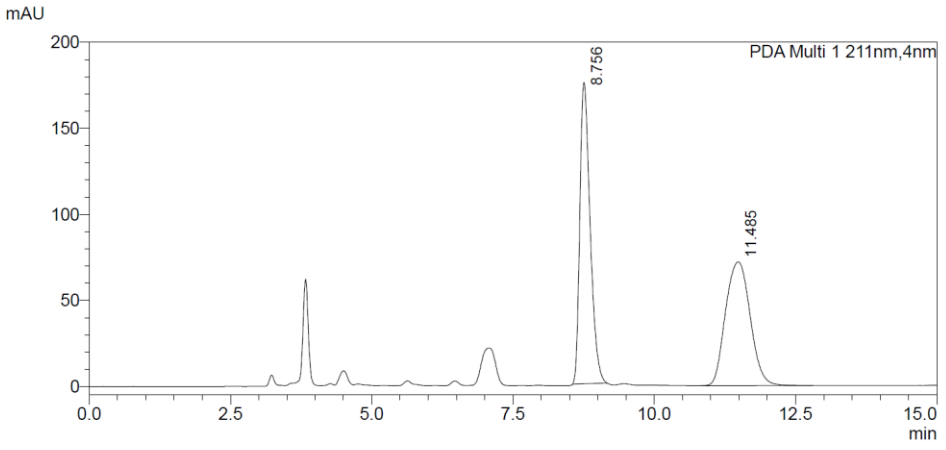


**<Peak Table>**

PDA Ch1 211nm

| Peak# | Ret. Time | Area% |
|---|---|---|
| 1 | 8.756 | 50.307 |
| 2 | 11.485 | 49.693 |
| Total | | 100.000 |

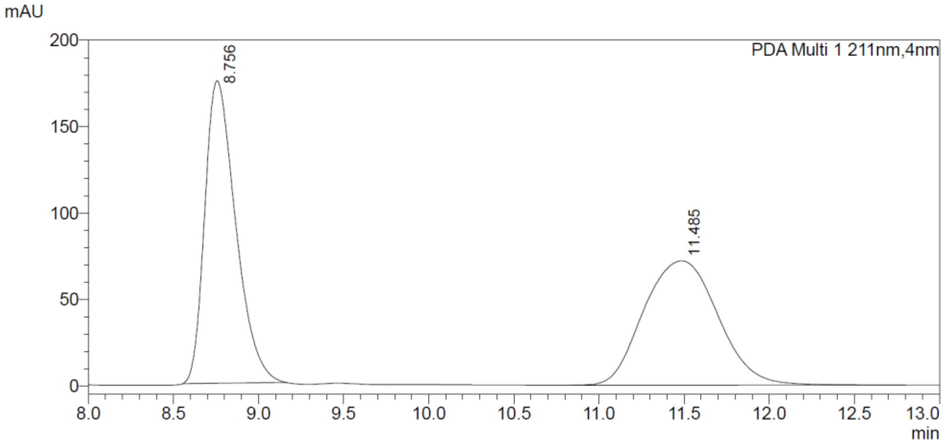


**<Peak Table>**

PDA Ch1 211nm

| Peak# | Ret. Time | Area% |
|---|---|---|
| 1 | 8.756 | 50.307 |
| 2 | 11.485 | 49.693 |
| Total | | 100.000 |

**After 480 minutes**: $t_R$(major): 8.5 min, $t_R$(minor): 11.2 min, 61:39 er.

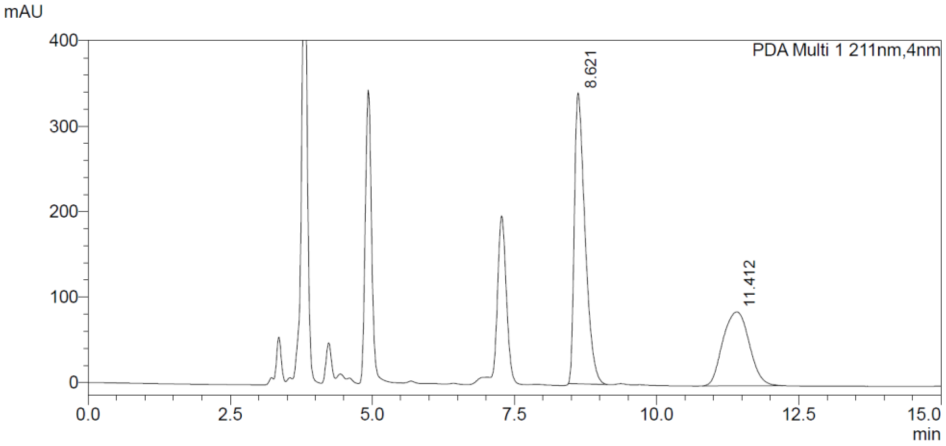


**<Peak Table>**

PDA Ch1 211nm

| Peak# | Ret. Time | Area% |
|---|---|---|
| 1 | 8.621 | 61.475 |
| 2 | 11.412 | 38.525 |
| Total | | 100.000 |

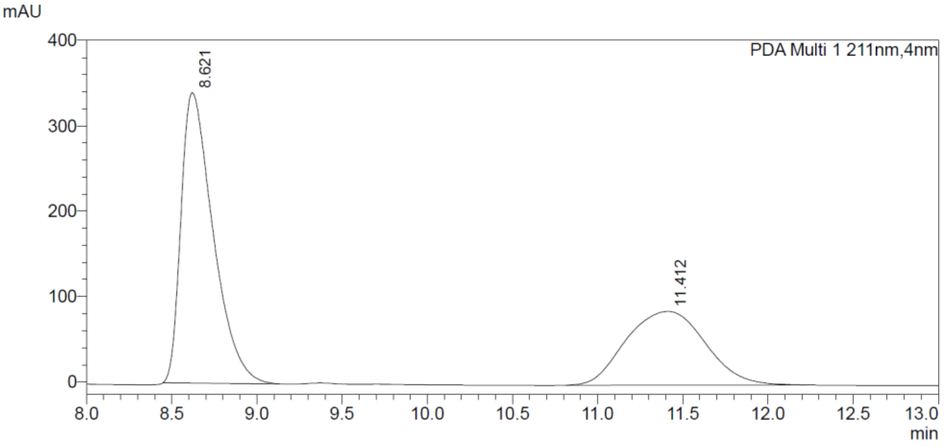


**<Peak Table>**

PDA Ch1 211nm

| Peak# | Ret. Time | Area% |
|---|---|---|
| 1 | 8.621 | 61.475 |
| 2 | 11.412 | 38.525 |
| Total | | 100.000 |

**After 1440 minutes**: $t_R$(major): 8.8 min, $t_R$(minor): 11.7 min, 84:16 er.

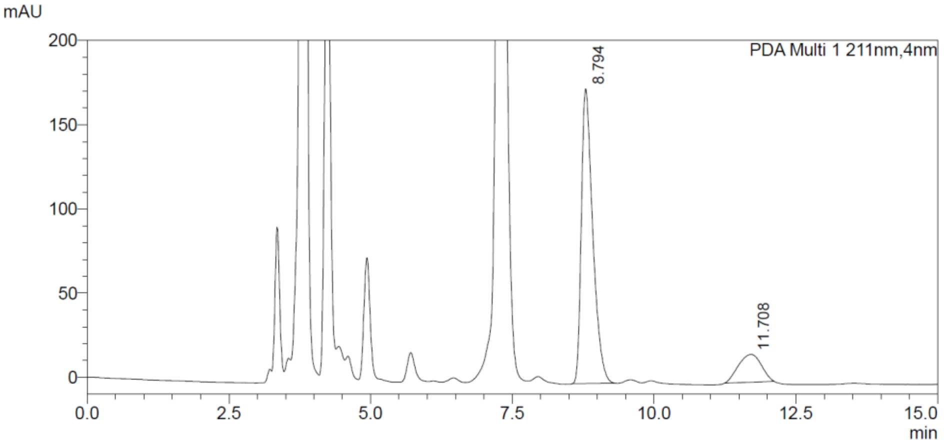


**<Peak Table>**

PDA Ch1 211nm

| Peak# | Ret. Time | Area% |
|---|---|---|
| 1 | 8.794 | 84.105 |
| 2 | 11.708 | 15.895 |
| Total | | 100.000 |

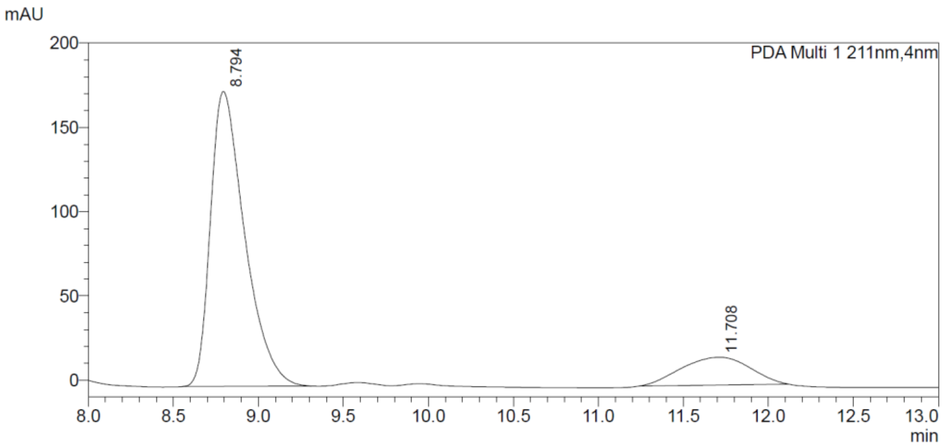


**<Peak Table>**

PDA Ch1 211nm

| Peak# | Ret. Time | Area% |
|---|---|---|
| 1 | 8.794 | 84.105 |
| 2 | 11.708 | 15.895 |
| Total | | 100.000 |

*HPLC data for reaction A (re = repeat):*

| Replicate | **Time** (minutes) | 0 | 60 | 120 | 180 | 240 | 300 | 360 | 420 | 480 | 1440 |
|---|---|---|---|---|---|---|---|---|---|---|---|
| **1** | **46 (ee)** | 0 | 5 | 10 | 13 | 16 | 17 | 18 | 20 | 23 | 68 |
| | **7 (ee)** | / | >99 | >99 | >99 | >99 | >99 | >99 | >99 | >99 | >99 |
| **2** | **46 (ee) re1** | 0 | 5 | 10 | 13 | 16 | 17 | 18 | 20 | 22 | 63 |
| | **7 (ee) re1** | / | >99 | >99 | >99 | >99 | >99 | >99 | >99 | >99 | >99 |
| **3** | **46 (ee) re2** | 0 | 5 | 11 | 14 | 16 | 18 | 19 | 21 | 25 | 66 |
| | **7 (ee) re2** | / | >99 | >99 | >99 | >99 | >99 | >99 | >99 | >99 | >99 |

*6.2 Double Diastereodifferentiation Analysis*

Following the general consideration, aliquots (50 microL) were removed from the reaction mixture for analysis every 60 minutes for first 480 minutes of reaction, the sample for the last measurement

was removed at 1440 minutes.

Mis-match case **B**: slow product formation and increased enantioselectivity of **(*R*)-46**

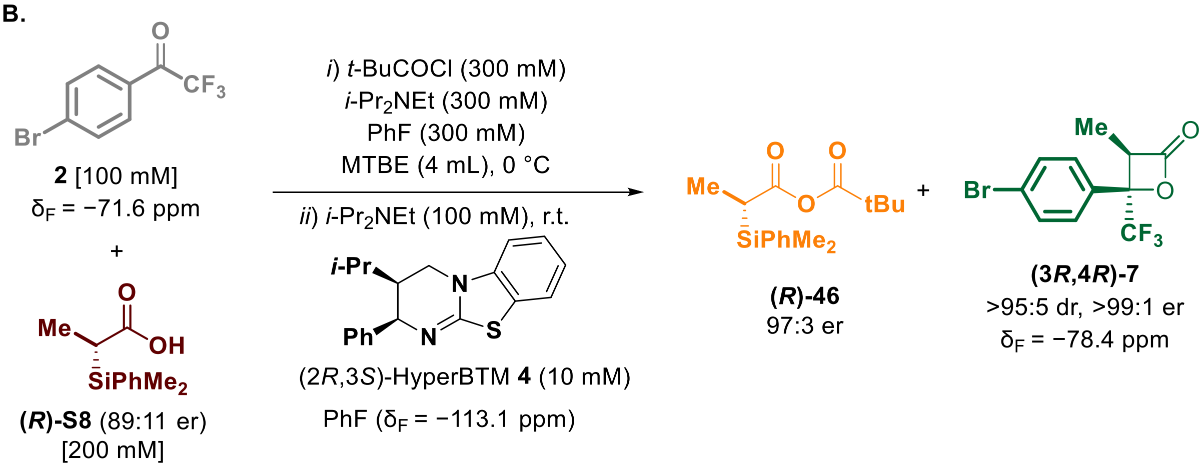


**Scheme S2**. Kinetic procedure with **(*R*)-S8** and (2*R*,3*S*)-HyperBTM **4**, initial concentrations: **(*R*)-S8** (200 mM), **2** (100 mM), *i*Pr2NEt (300 +100 mM), pivaloyl chloride (300 mM), PhF (300 mM), (2*R*,3*S*)-HyperBTM **4** (10 mM).

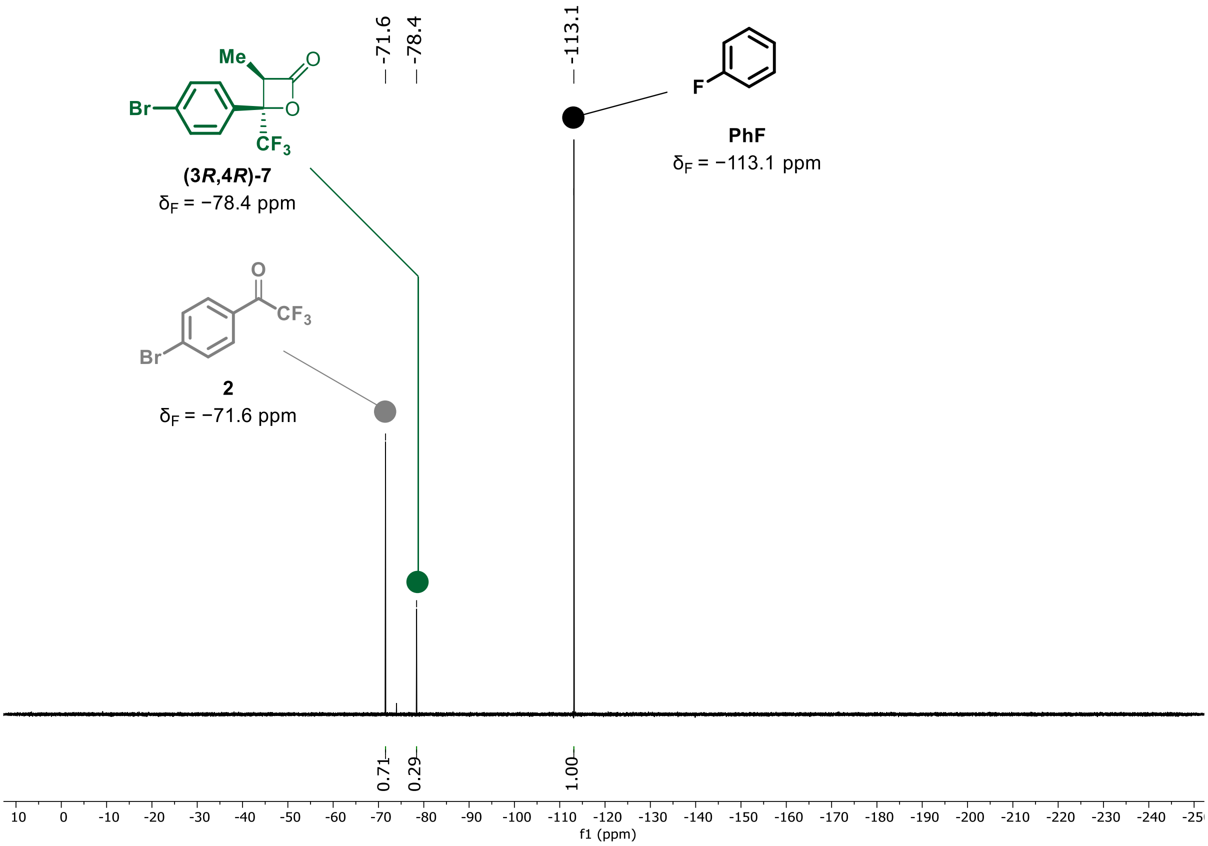


**Figure S4**. $^{19}F\{^1H\}$ Spectra (376 MHz) ($CDCl_3$) obtained following the standard procedure for kinetic experiments

after 480 minutes.

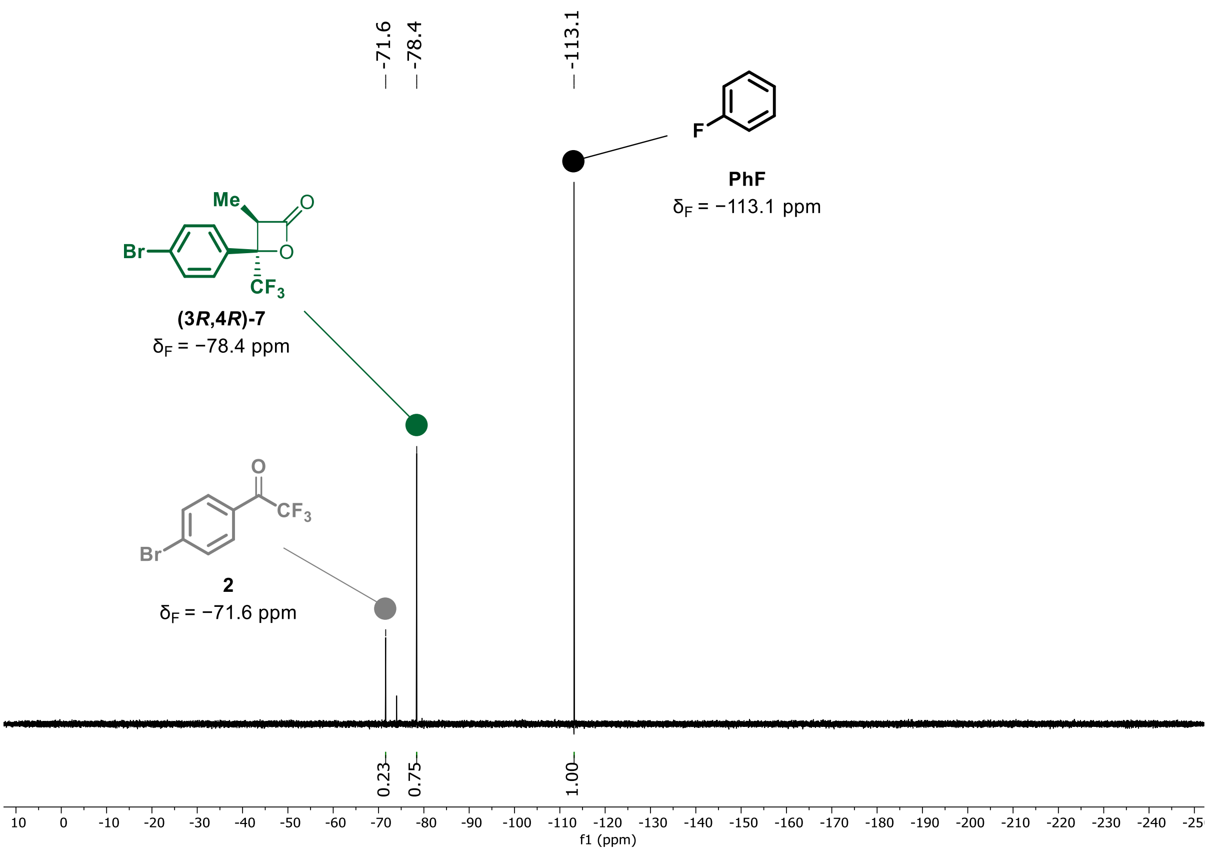


**Figure S5**. $^{19}$F{$^{1}$H} Spectra (376 MHz) ($CDCl_3$) obtained following the standard procedure for kinetic experiments after 1440 minutes.

*Concentration data for reaction B (re = repeat):*

| Replicate | **Time** (minutes) | 0 | 60 | 120 | 180 | 240 | 300 | 360 | 420 | 480 | 1440 |
|---|---|---|---|---|---|---|---|---|---|---|---|
| **1** | **2 (mM)** | 100 | 97 | 92 | 88 | 86 | 81 | 77 | 74 | 71 | 23 |
| | **7 (mM)** | 0 | 3 | 7 | 11 | 14 | 18 | 22 | 25 | 29 | 75 |
| **2** | **2 (mM) re1** | 100 | 97 | 93 | 90 | 86 | 82 | 79 | 76 | 72 | 26 |

| | | | | | | | | | | | |
|---|---|---|---|---|---|---|---|---|---|---|---|
| | **7 (mM) re1** | 0 | 3 | 7 | 10 | 14 | 18 | 21 | 24 | 28 | 72 |
| **3** | **2 (mM) re2** | 100 | 96 | 93 | 89 | 86 | 83 | 80 | 75 | 70 | 26 |
| | **7 (mM) re2** | 0 | 4 | 7 | 11 | 14 | 17 | 20 | 25 | 30 | 72 |

HPLC spectra for **46**: Chiralcel OD-H (99.9:0.1 hexane:IPA, flow rate 1.0 mL min$^{-1}$, 211 nm, 30 °C).

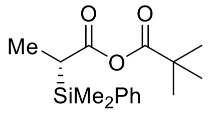


**46**

**After 480 minutes**: $t_R$(major): 8.7 min, $t_R$(minor): 11.6 min, 92:8 er.

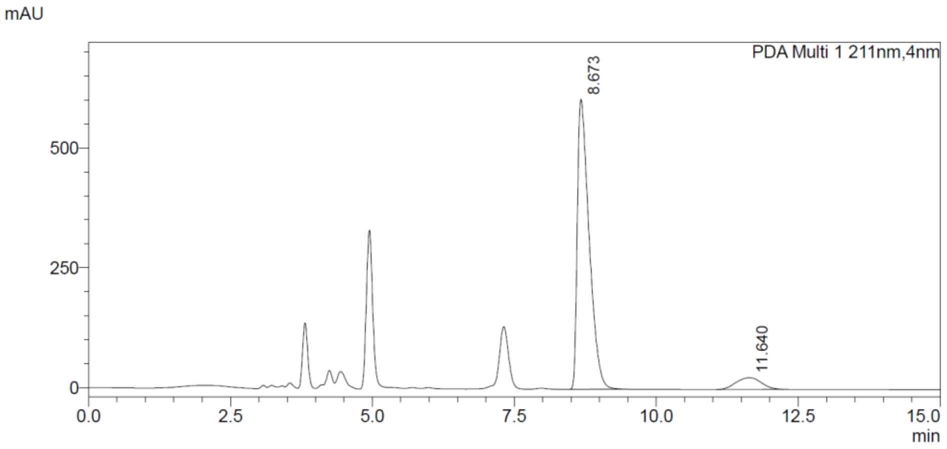


**<Peak Table>**

PDA Ch1 211nm

| Peak# | Ret. Time | Area% |
|---|---|---|
| 1 | 8.673 | 92.084 |
| 2 | 11.640 | 7.916 |
| Total | | 100.000 |

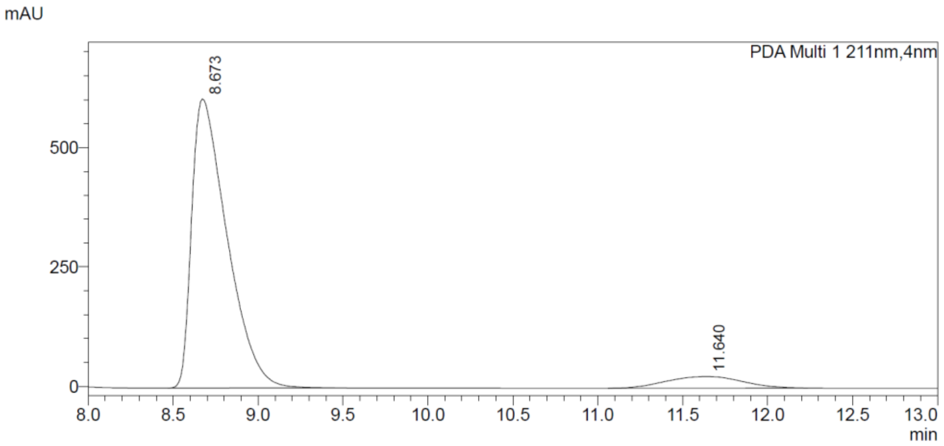


**<Peak Table>**

PDA Ch1 211nm

| Peak# | Ret. Time | Area% |
|---|---|---|
| 1 | 8.673 | 92.084 |
| 2 | 11.640 | 7.916 |
| Total | | 100.000 |

**After 1440 minutes**: $t_R$(major): 8.5 min, $t_R$(minor): 11.4 min, 97:3 er.

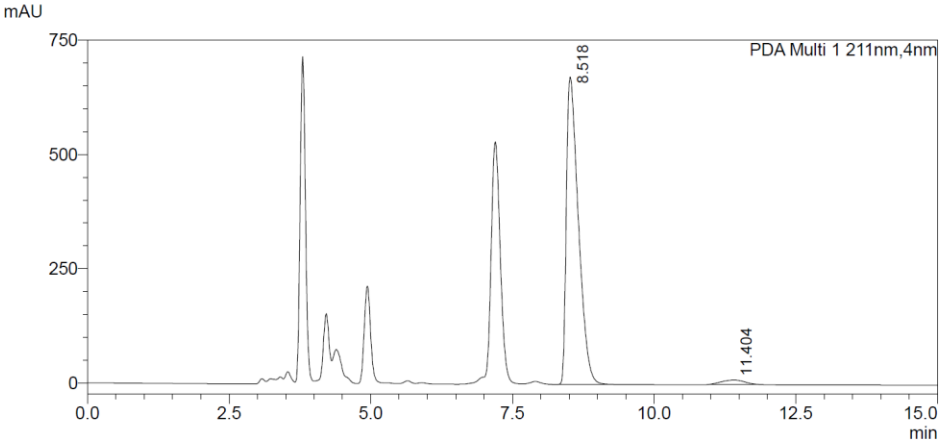


**<Peak Table>**

PDA Ch1 211nm

| Peak# | Ret. Time | Area% |
|---|---|---|
| 1 | 8.518 | 97.268 |
| 2 | 11.404 | 2.732 |
| Total | | 100.000 |

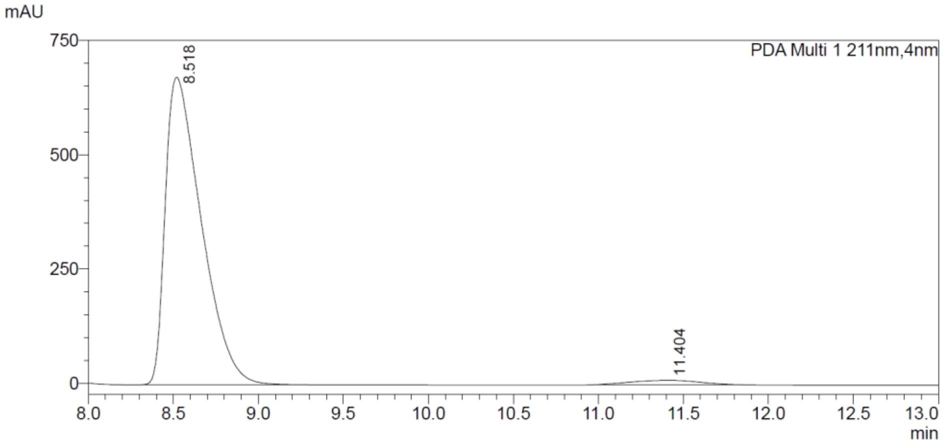


**<Peak Table>**

PDA Ch1 211nm

| Peak# | Ret. Time | Area% |
|---|---|---|
| 1 | 8.518 | 97.268 |
| 2 | 11.404 | 2.732 |
| Total | | 100.000 |

HPLC spectra for **7**: Chiralpak AS-H (99.5:0.5 hexane:IPA, flow rate 1.0 mLmin$^{-1}$, 211 nm, 30 °C), $t_R$ (major): 4.4 min, >99:1 er.

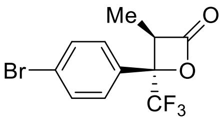


**(3*R*,4*R*)-7**

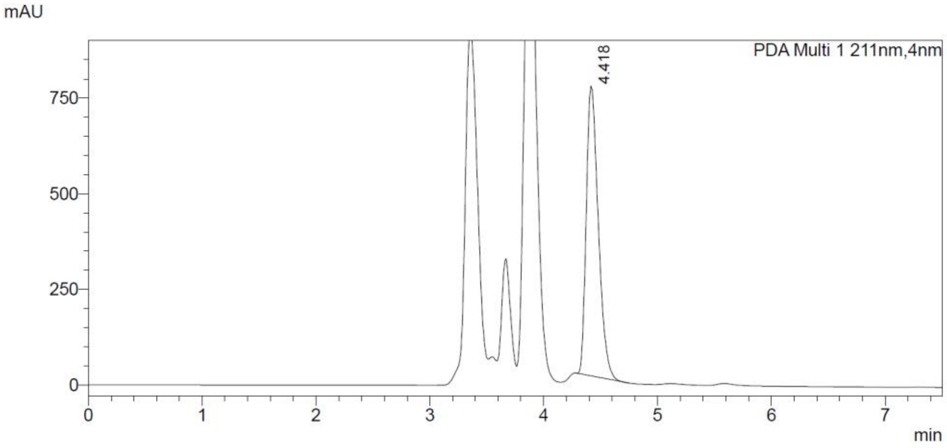


**<Peak Table>**

PDA Ch1 211nm

| Peak# | Ret. Time | Area% |
|---|---|---|
| 1 | 4.418 | 100.000 |
| Total | | 100.000 |

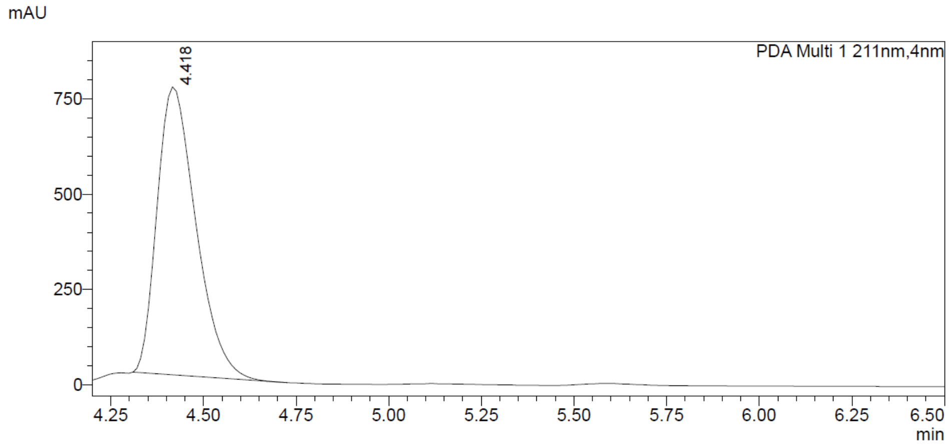


**<Peak Table>**

PDA Ch1 211nm

| Peak# | Ret. Time | Area% |
|---|---|---|
| 1 | 4.418 | 100.000 |
| Total | | 100.000 |

*HPLC data for reaction B (re = repeat):*

| Replicate | **Time** (minutes) | 0 | 60 | 120 | 180 | 240 | 300 | 360 | 420 | 480 | 1440 |
|---|---|---|---|---|---|---|---|---|---|---|---|
| **1** | **46 (ee)** | 79 | 79 | 79 | 82 | 81 | 82 | 83 | 83 | 84 | 94 |
| | **7 (ee)** | / | / | >99 | / | / | / | >99 | / | >99 | >99 |
| **2** | **46 (ee) re1** | 79 | 81 | 82 | 82 | 83 | 83 | 84 | 86 | 86 | 94 |
| | **7 (ee) re1** | / | / | >99 | / | / | / | >99 | / | >99 | >99 |
| **3** | **46 (ee) re2** | 79 | 81 | 82 | 82 | 83 | 83 | 84 | 85 | 86 | 93 |
| | **7 (ee) re2** | / | / | >99 | / | / | / | >99 | / | >99 | >99 |

Match case **C**: fast product formation and decreased enantioselectivity of **(*R*)-46**

C.

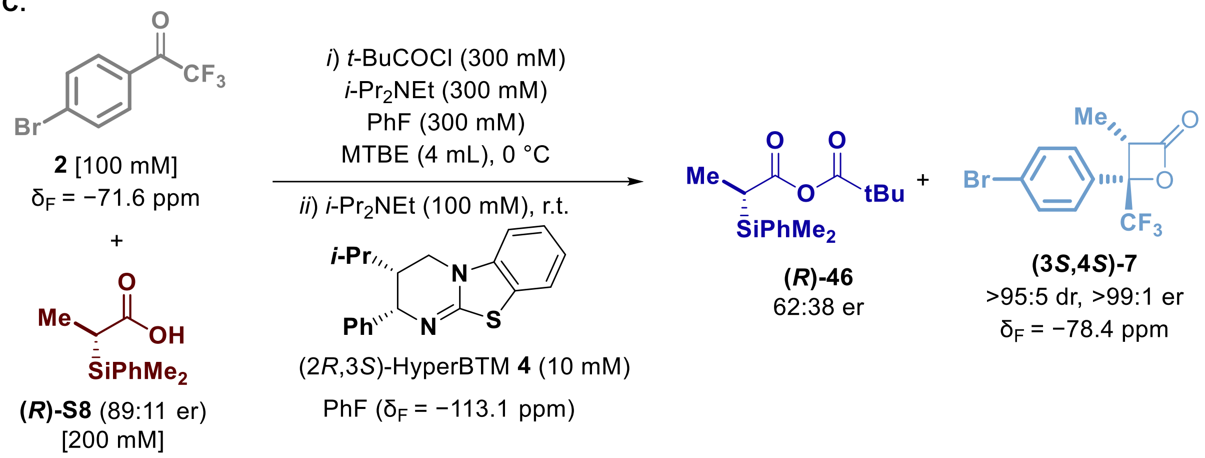


**Scheme S3**. Kinetic procedure with **(*R*)-S8** and (2*S*,3*R*)-HyperBTM **4**, initial concentrations: **(*R*)-S8** (200 mM), **2** (100 mM), *i*$Pr_2$NEt (300 +100 mM), pivaloyl chloride (300 mM), PhF (300 mM), (2*S*,3*R*)-HyperBTM **4** (10 mM).

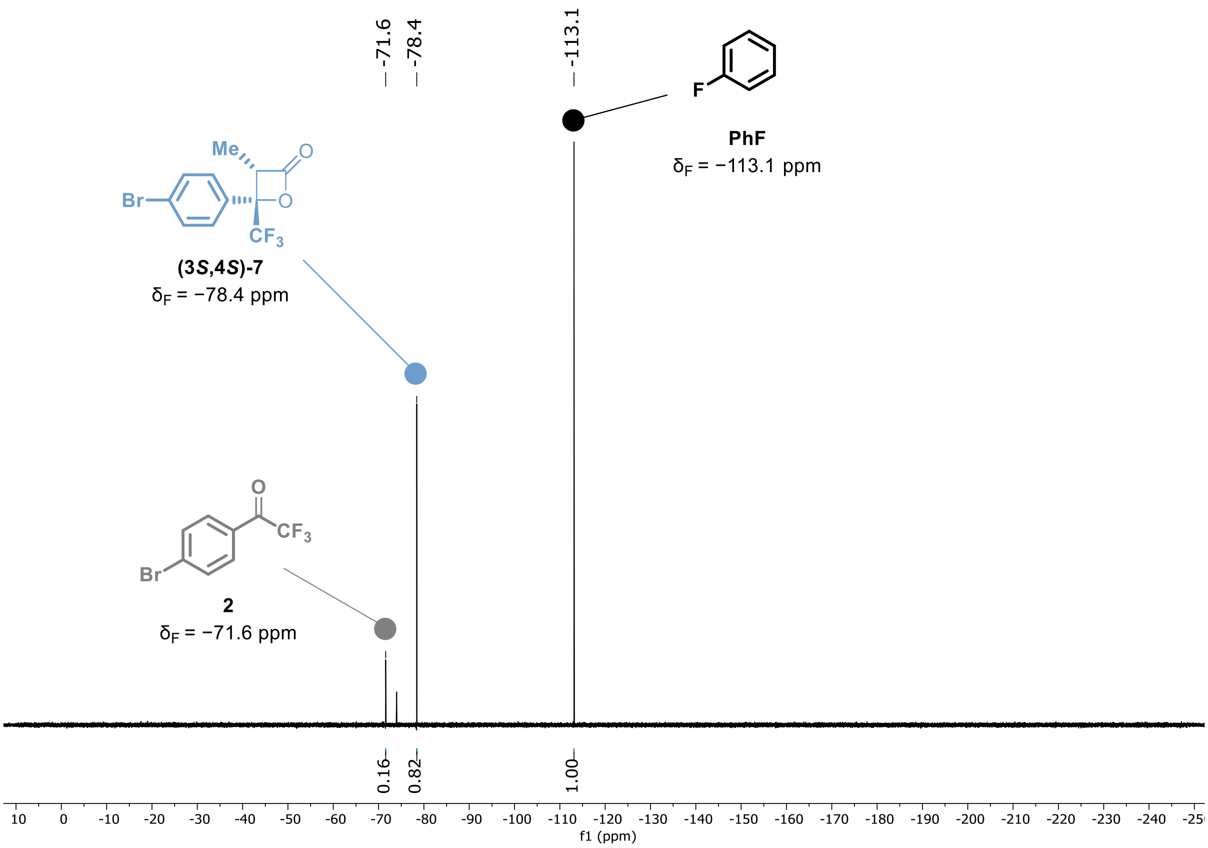


**Figure S6**. $^{19}F\{^1H\}$ Spectra (376 MHz) ($CDCl_3$) obtained following the standard procedure for kinetic experiments after 480 minutes.

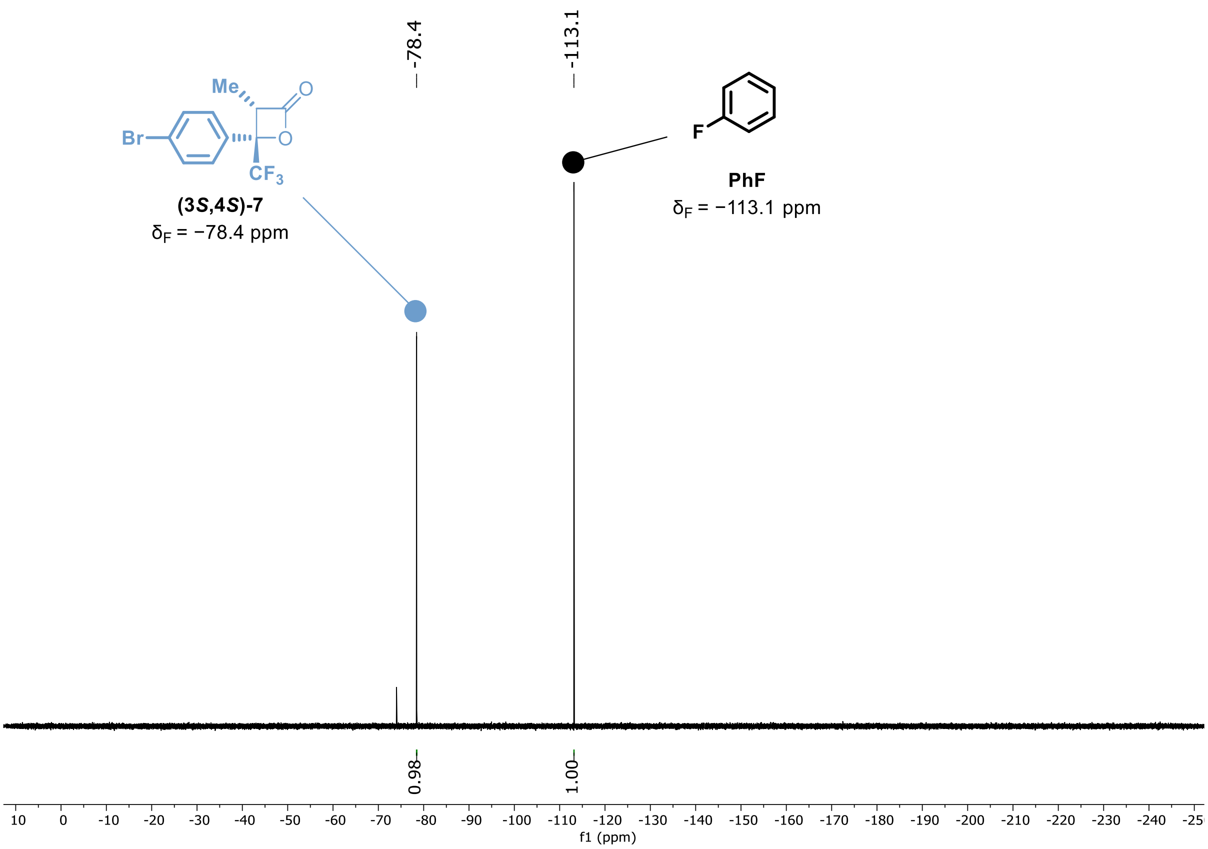


**Figure S7**. $^{19}F\{^{1}H\}$ Spectra (376 MHz) ($CDCl_3$) obtained following the standard procedure for kinetic experiments after 1440 minutes.

*Concentration data for reaction C (re = repeat):*

| Replicate | **Time** (minutes) | 0 | 60 | 120 | 180 | 240 | 300 | 360 | 420 | 480 | 1440 |
|---|---|---|---|---|---|---|---|---|---|---|---|
| **1** | **2 (mM)** | 100 | 90 | 78 | 67 | 56 | 45 | 35 | 26 | 16 | 0 |
| | **7 (mM)** | 0 | 10 | 22 | 32 | 44 | 54 | 65 | 73 | 82 | 98 |
| **2** | **2 (mM) re1** | 100 | 89 | 79 | 68 | 56 | 45 | 34 | 24 | 14 | 0 |
| | **7 (mM) re1** | 0 | 11 | 21 | 32 | 44 | 54 | 65 | 75 | 84 | 96 |

| | | | | | | | | | | | |
|---|---|---|---|---|---|---|---|---|---|---|---|
| **3** | **2 (mM) re2** | 100 | 87 | 78 | 67 | 56 | 45 | 31 | 22 | 11 | 0 |
| | **7 (mM) re2** | 0 | 12 | 21 | 31 | 43 | 53 | 64 | 73 | 84 | 95 |

HPLC spectra for **46**: Chiralcel OD-H (99.9:0.1 hexane:IPA, flow rate 1.0 mL min$^{-1}$, 211 nm, 30 °C).

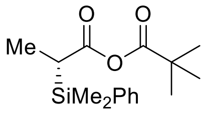


**46**

**After 480 minutes**: $t_R$(major): 8.6 min, $t_R$(minor): 11.5 min, 82:18 er.

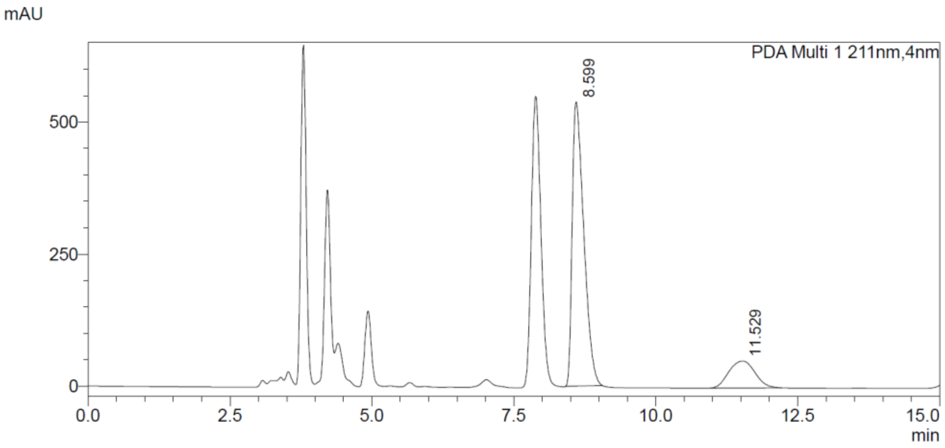


**<Peak Table>**

PDA Ch1 211nm

| Peak# | Ret. Time | Area% |
|---|---|---|
| 1 | 8.599 | 82.489 |
| 2 | 11.529 | 17.511 |
| Total | | 100.000 |

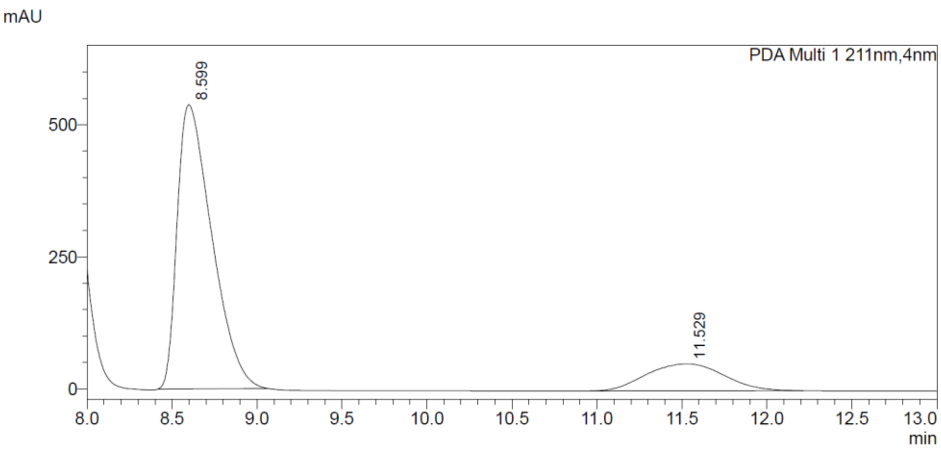


<Peak Table>

PDA Ch1 211nm

| Peak# | Ret. Time | Area% |
|---|---|---|
| 1 | 8.599 | 82.489 |
| 2 | 11.529 | 17.511 |
| Total | | 100.000 |

**After 1440 minutes**: $t_R$(major): 8.7 min, $t_R$(minor): 11.5 min, 62:38 er.

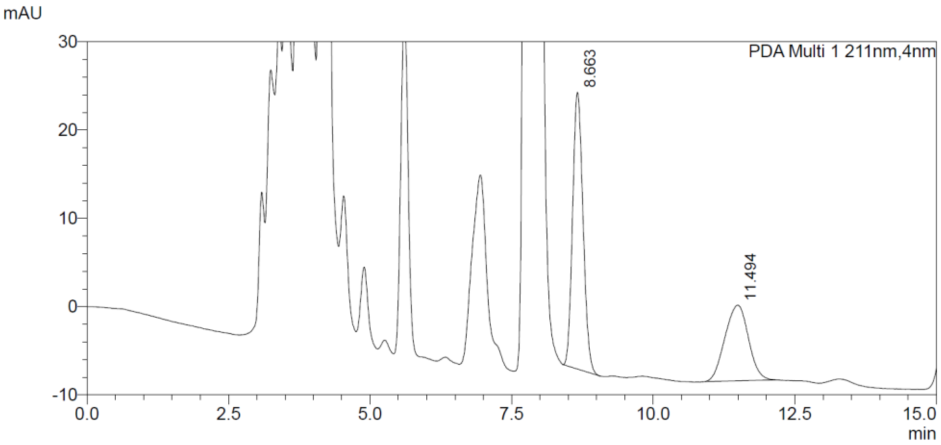


<Peak Table>

PDA Ch1 211nm

| Peak# | Ret. Time | Area% |
|---|---|---|
| 1 | 8.663 | 61.871 |
| 2 | 11.494 | 38.129 |
| Total | | 100.000 |

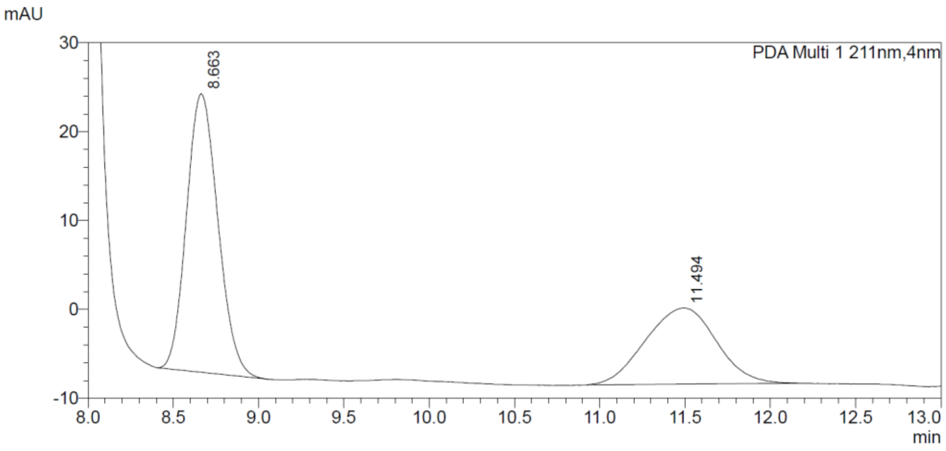


**<Peak Table>**

PDA Ch1 211nm

| Peak# | Ret. Time | Area% |
|---|---|---|
| 1 | 8.663 | 61.871 |
| 2 | 11.494 | 38.129 |
| Total | | 100.000 |

HPLC spectra for **7**: Chiralpak AS-H (99.5:0.5 hexane:IPA, flow rate 1.0 mLmin$^{-1}$, 211 nm, 30 °C), $t_R$ (major): 5.7 min, >99:1 er.

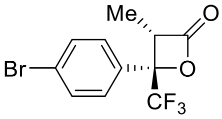


**(3*S*,4*S*)-7**

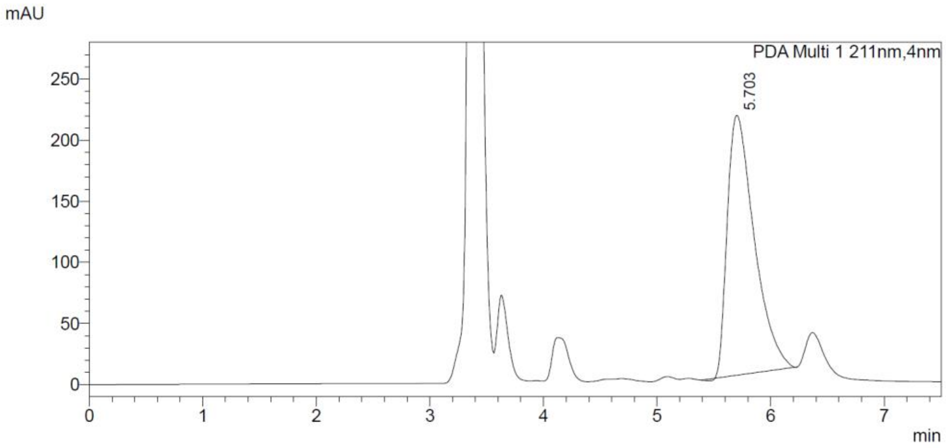


**<Peak Table>**

PDA Ch1 211nm

| Peak# | Ret. Time | Area% |
|---|---|---|
| 1 | 5.703 | 100.000 |
| Total | | 100.000 |

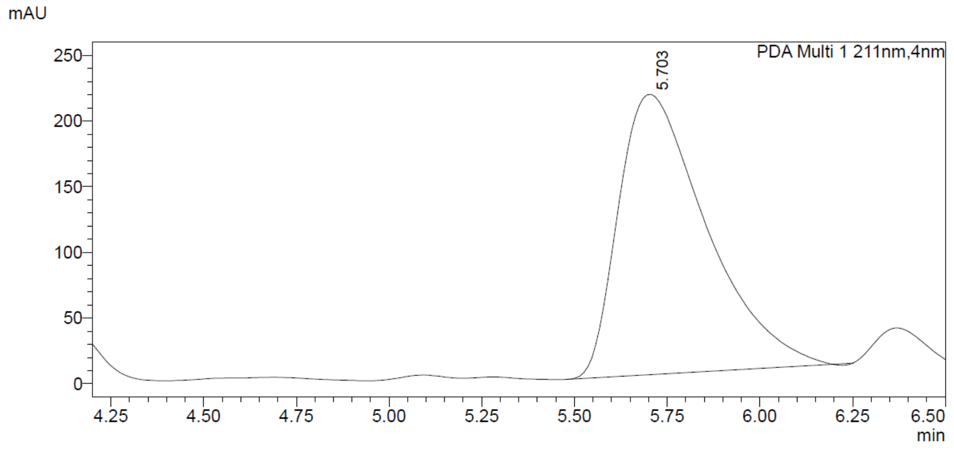


**<Peak Table>**

PDA Ch1 211nm

| Peak# | Ret. Time | Area% |
|---|---|---|
| 1 | 5.703 | 100.000 |
| Total | | 100.000 |

*HPLC data for reaction C (re = repeat):*

| Replicate | **Time** (minutes) | 0 | 60 | 120 | 180 | 240 | 300 | 360 | 420 | 480 | 1440 |
|---|---|---|---|---|---|---|---|---|---|---|---|
| **1** | **46 (ee)** | 79 | 77 | 78 | 76 | 74 | 73 | 71 | 69 | 65 | 24 |
| | **7 (ee)** | / | / | >99 | / | / | / | >99 | / | >99 | >99 |
| **2** | **46 (ee) re1** | 79 | 78 | 77 | 76 | 74 | 73 | 71 | 70 | 67 | 24 |
| | **7 (ee) re1** | / | / | >99 | / | / | / | >99 | / | >99 | >99 |
| **3** | **46 (ee) re2** | 79 | 77 | 77 | 75 | 73 | 72 | 72 | 69 | 67 | 24 |
| | **7 (ee) re2** | / | / | >99 | / | / | / | >99 | / | >99 | >99 |

*6.3 Kinetic Analysis by Using Alternative α-Silyl Acid*

Following the general consideration, aliquots (50 microL) were removed from the reaction mixture

for analysis every 60 minutes for first 480 minutes of reaction, the sample for the last measurement was removed at 1440 minutes.

*Kinetic analysis by using acid **1** and **S10***

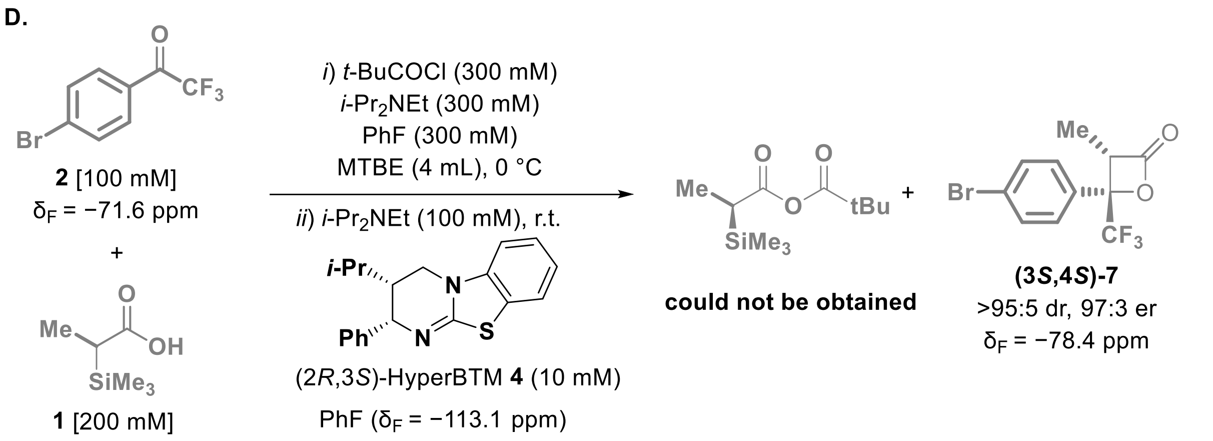


**Scheme S4**. Kinetic procedure with acid **1** and **S10** and (2*S*,3*R*)-HyperBTM **4**, initial concentrations: acid **1** or acid **S10** (200 mM), **2** (100 mM), *i*Pr$_2$NEt (300 +100 mM), pivaloyl chloride (300 mM), PhF (300 mM), (2*S*,3*R*)-HyperBTM **4** (10 mM).

*Concentration data for reaction D (re = repeat):*

| Replicate | **Time** (minutes) | 0 | 60 | 120 | 180 | 240 | 300 | 360 | 420 | 480 | 1440 |
|---|---|---|---|---|---|---|---|---|---|---|---|
| **1** | **1-2 (mM)** | 100 | 95 | 87 | 79 | 71 | 63 | 55 | 47 | 39 | 0 |
| | **1-7 (mM)** | 0 | 5 | 13 | 21 | 29 | 37 | 45 | 53 | 61 | 93 |

| | | | | | | | | | | | |
|---|---|---|---|---|---|---|---|---|---|---|---|
| **2** | **1-2 (mM) re1** | 100 | 94 | 88 | 81 | 73 | 65 | 57 | 49 | 41 | 0 |
| | **1-7 (mM) re1** | 0 | 6 | 12 | 19 | 27 | 35 | 43 | 51 | 59 | 91 |
| **3** | **1-2 (mM) re2** | 100 | 95 | 87 | 80 | 73 | 64 | 56 | 48 | 40 | 0 |
| | **1-7 (mM) re2** | 0 | 5 | 13 | 20 | 27 | 36 | 44 | 52 | 60 | 92 |
| **1** | **S10-2 (mM)** | 100 | 91 | 84 | 78 | 71 | 66 | 61 | 57 | 53 | 32 |
| | **S10-7 (mM)** | 0 | 9 | 16 | 22 | 29 | 34 | 39 | 43 | 47 | 68 |
| **2** | **S10-2 (mM) re1** | 100 | 91 | 82 | 74 | 67 | 62 | 57 | 54 | 50 | 33 |
| | **S10-7 (mM) re1** | 0 | 9 | 18 | 26 | 33 | 38 | 43 | 46 | 50 | 67 |
| **3** | **S10-2 (mM) re2** | 100 | 91 | 83 | 76 | 69 | 64 | 59 | 56 | 52 | 34 |
| | **S10-7 (mM) re2** | 0 | 9 | 17 | 24 | 31 | 36 | 41 | 44 | 48 | 64 |

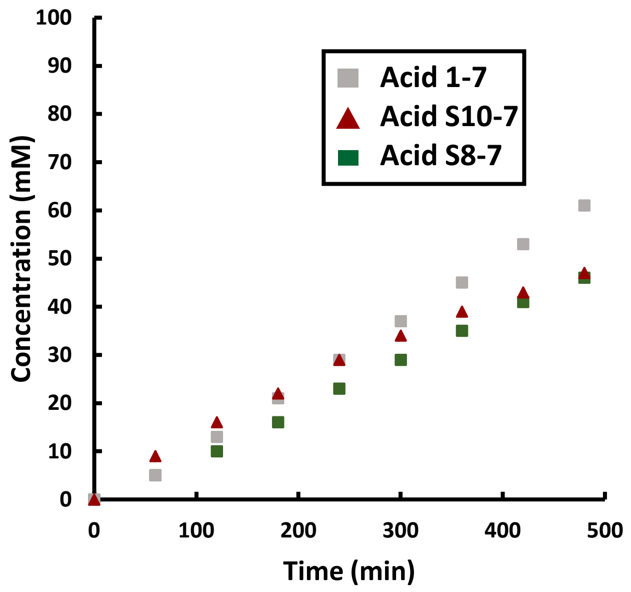

**Figure S8**. Formation of product **7** from alternative α-silyl acid and compare to the standard procedure.

HPLC conditions for **S11**: Chiralcel OD-H (99:1 hexane:IPA, flow rate 0.5 mL min$^{-1}$, 211 nm, 30 °C).

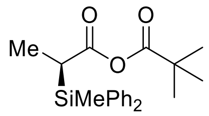


**(*S*)-S11**

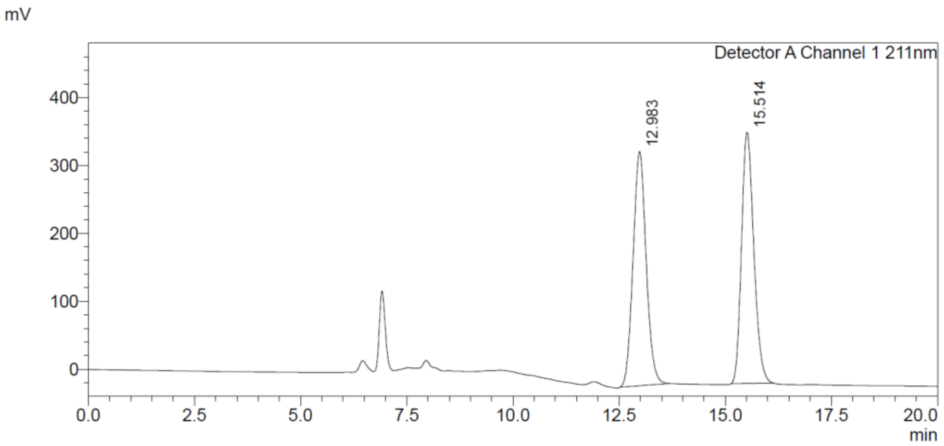


**<Peak Table>**

Detector A Channel 1 211nm

| Peak# | Ret. Time | Area% |
|---|---|---|
| 1 | 12.983 | 49.866 |
| 2 | 15.514 | 50.134 |
| Total | | 100.000 |

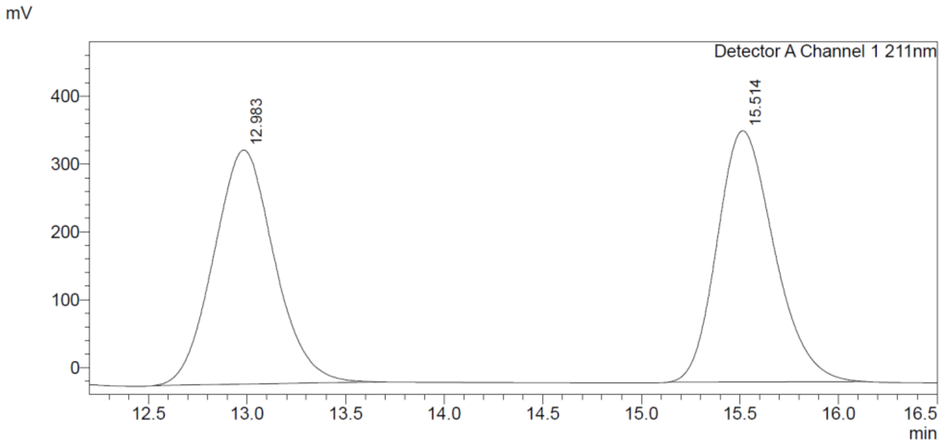


**<Peak Table>**

Detector A Channel 1 211nm

| Peak# | Ret. Time | Area% |
|---|---|---|
| 1 | 12.983 | 49.866 |
| 2 | 15.514 | 50.134 |
| Total | | 100.000 |

**After 480 minutes**: $t_R$(minor): 12.8 min, $t_R$(major): 15.3 min, 69:31 er.

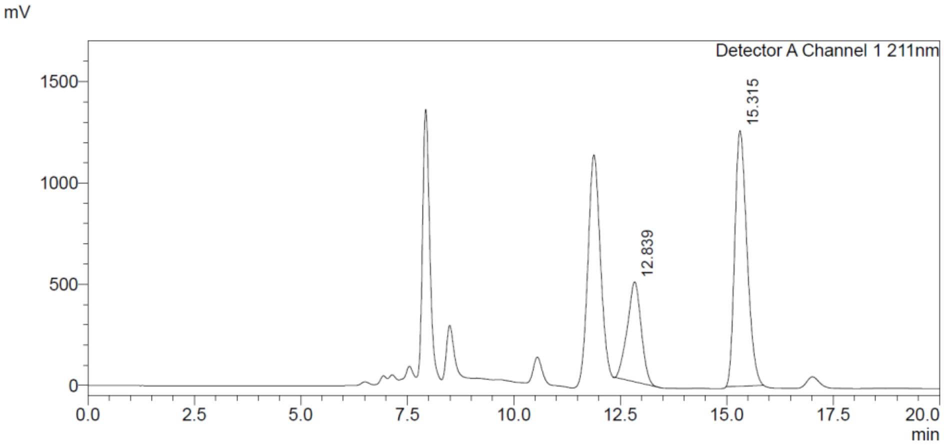


**<Peak Table>**

Detector A Channel 1 211nm

| Peak# | Ret. Time | Area% |
|---|---|---|
| 1 | 12.839 | 30.704 |
| 2 | 15.315 | 69.296 |
| Total | | 100.000 |

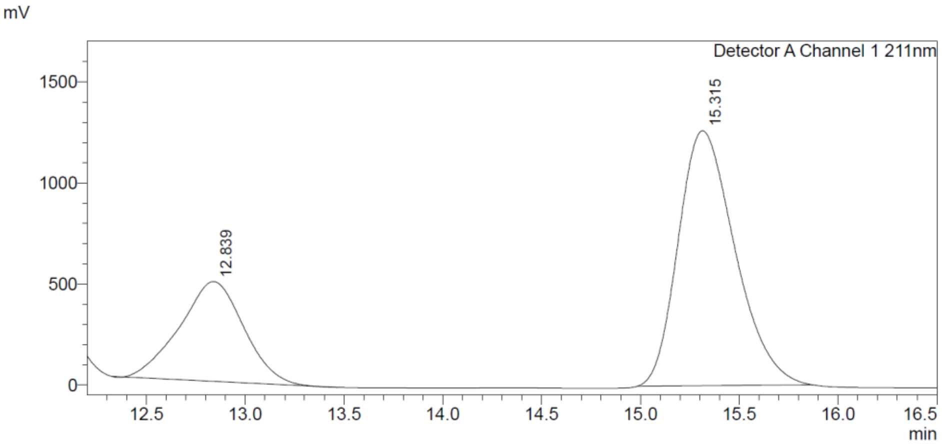


**<Peak Table>**

Detector A Channel 1 211nm

| Peak# | Ret. Time | Area% |
|---|---|---|
| 1 | 12.839 | 30.704 |
| 2 | 15.315 | 69.296 |
| Total | | 100.000 |

**After 1440 minutes**: $t_R$(minor): 12.8 min, $t_R$(minor): 15.3 min, 94:6 er.

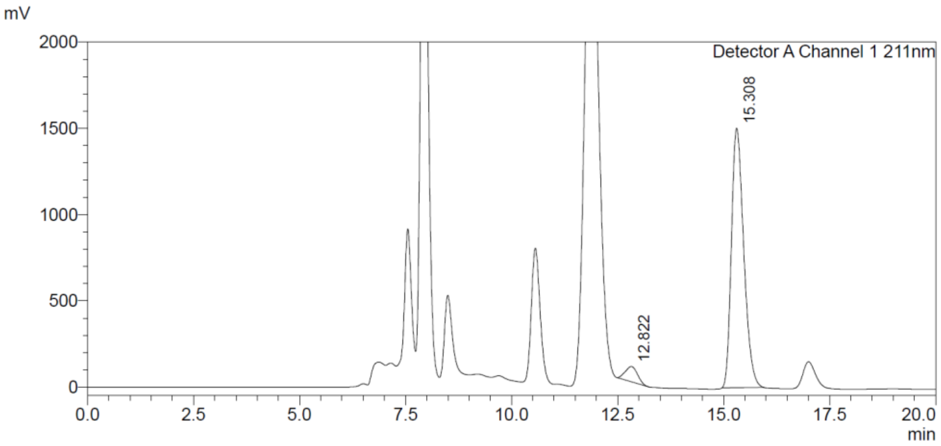


**<Peak Table>**

Detector A Channel 1 211nm

| Peak# | Ret. Time | Area% |
|---|---|---|
| 1 | 12.822 | 5.526 |
| 2 | 15.308 | 94.474 |
| Total | | 100.000 |

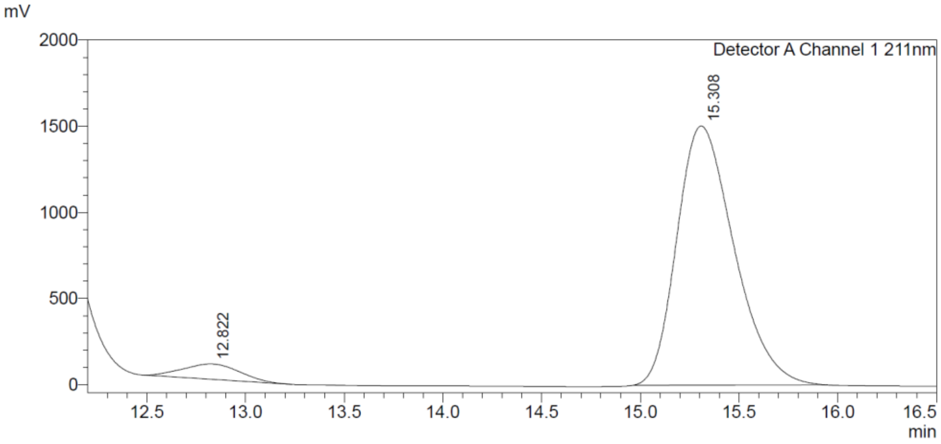


**<Peak Table>**

Detector A Channel 1 211nm

| Peak# | Ret. Time | Area% |
|---|---|---|
| 1 | 12.822 | 5.526 |
| 2 | 15.308 | 94.474 |
| Total | | 100.000 |

*HPLC data for reactionD:*

| **Time** (minutes) | 0 | 60 | 120 | 180 | 240 | 300 | 360 | 420 | 480 | 1440 |
|---|---|---|---|---|---|---|---|---|---|---|
| **S11 (ee)** | 0 | 16 | 20 | 22 | 24 | 26 | 28 | 34 | 38 | 89 |

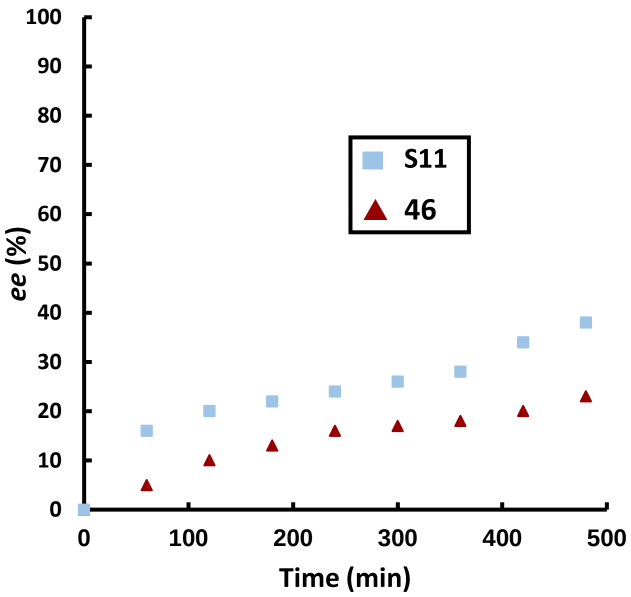


**Figure S9**. Enantioselectivity variation of **S11** and compare to the standard procedure.

## 7. Determination of Product Configuration by X-ray Crystallography

X-ray diffraction data for all three compounds were collected using a Rigaku MM-007HF High Brilliance RA generator/confocal optics [Cu Kα radiation (λ = 1.54187 Å)]. An XtaLAB P200 diffractometer (data collected at 125 K) was used for **24**, while an XtaLAB P100 diffractometer (data collected at 173 K) was used for **37** and **39**. Intensity data were collected using either ω steps or both ω and φ steps accumulating area detector images spanning at least a hemisphere of reciprocal space. Data for all compounds analysed were collected using CrystalClear[16] and processed (including correction for Lorentz, polarization and absorption) using CrysAlisPro.[17] Structures were solved by dual-space (SHELXT)[18] or Patterson (PATTY)[19] methods and refined by full-matrix least-squares against $F^2$ (SHELXL-2018/3).[20] Nonhydrogen atoms were refined anisotropically, and hydrogen atoms were refined using a riding mode, except for those bound to nitrogen, which were located from the difference Fourier map and refined isotropically subject to a distance restraint. All calculations were performed using the CrystalStructure[21] interface. Selected crystallographic data are presented in the following tables. Deposition numbers 2176399–2176401 contains the supplementary crystallographic data for this paper. These data are provided free of charge by the joint Cambridge Crystallographic Data Centre and Fachinformationszentrum Karlsruhe Access Structure service *www.ccdc.cam.ac.uk/structures*.

| | **(*S*)-24** |
|---|---|
| CDCC | 2176399 |
| empirical formula | $C_{13}H_6BrF_9O_2$ |
| crystal size [mm] | 0.13×0.02×0.01 |
| fw | 445.08 |
| crystal description | colourless needle |
| space group | $P2_12_12_1$ (#19) |
| *a* [Å] | 6.0454 (3) |
| *b* [Å] | 9.8442 (5) |
| *c* [Å] | 25.7165 (11) |
| vol [Å]$^3$ | 1530.44 (13) |
| Z | 4 |
| $\rho$ (calc) [g/cm$^3$] | 1.932 |
| $\mu$ [mm$^{-1}$] | 4.725 |
| F(000) | 864.0 |
| reflections collected | 8868 |
| independent reflections ($R_{int}$) | 3092 (0.0819) |
| parameters/restraints | 327/25 |
| GOF on $F^2$ | 0.985 |
| $R_1$ [$I$>2σ($I$)] | 0.0628 |
| ωR$_2$ (all data) | 0.1825 |
| largest diff. peak/hole [e/ Å$^3$] | 0.61, −0.76 |
| Flack parameter | 0.09 (4) |

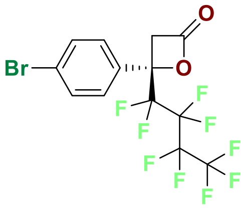

**(*S*)-24**

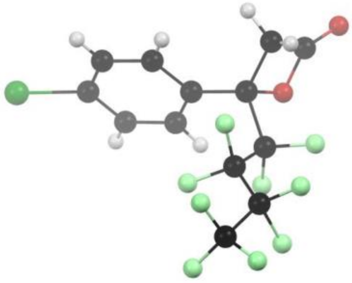

| | **(3*S*,4*S*)-37** |
|---|---|
| CDCC | 2176400 |
| empirical formula | $C_{19}H_{17}BrF_5NO_2$ |
| crystal size [mm] | 0.11×0.03×0.02 |
| fw | 466.24 |
| crystal description | colourless needle |
| space group | $P2_12_12_1$ (#19) |
| *a* [Å] | 6.20838 (13) |
| *b* [Å] | 7.51926 (15) |
| *c* [Å] | 40.6928 (10) |
| vol [Å]$^3$ | 1899.64 (7) |
| Z | 4 |
| *P* (calc) [g/cm$^3$] | 1.630 |
| $\mu$ [mm$^{-1}$] | 3.538 |
| F(000) | 936.0 |
| reflections collected | 19984 |
| independent reflections ($R_{int}$) | 3472 (0.0359) |
| parameters/restraints | 262/2 |
| GOF on $F^2$ | 1.048 |
| $R_1$ [$I$>2σ($I$)] | 0.0293 |
| ω$R_2$ (all data) | 0.0801 |
| largest diff. peak/hole [e/ Å$^3$] | 0.48, −0.62 |
| Flack parameter | −0.045 (8) |

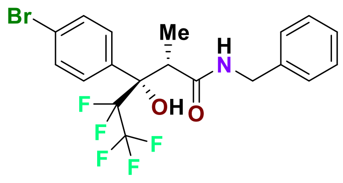


**(2*S*,3*S*)-37**

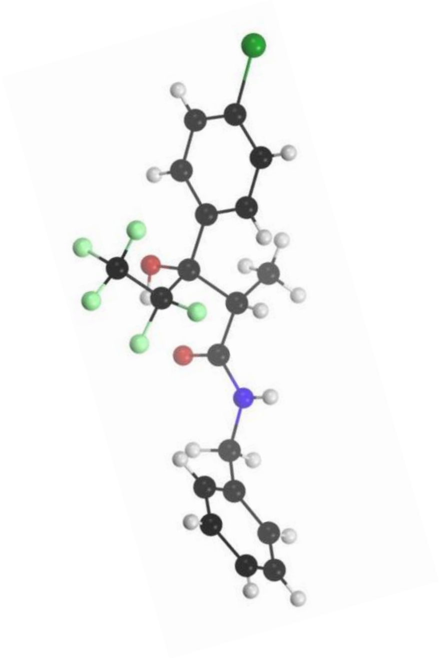

| | (3*S*,4*S*)-39 |
|---|---|
| CDCC | 2176401 |
| empirical formula | $C_{15}H_{12}BrF_9O_3$ |
| crystal size [mm] | 0.21×0.15×0.02 |
| fw | 491.15 |
| crystal description | colourless plate |
| space group | *C*2 (#5) |
| *a* [Å] | 18.2247 (11) |
| *b* [Å] | 5.8495 (4) |
| *c* [Å] | 17.2067 (12) |
| vol [Å]$^3$ | 1813.4 (2) |
| Z | 4 |
| *P* (calc) [g/cm$^3$] | 1.799 |
| $\mu$ [mm$^{-1}$] | 4.096 |
| F(000) | 968.0 |
| reflections collected | 8820 |
| independent reflections ($R_{int}$) | 2946 (0.0611) |
| parameters/restraints | 258/2 |
| GOF on $F^2$ | 1.209 |
| $R_1$ [$I$>2σ($I$)] | 0.0963 |
| $\omega R_2$ (all data) | 0.2692 |
| largest diff. peak/hole [e/ Å$^3$] | 1.72, −1.09 |
| Flack parameter | −0.18 (8) |

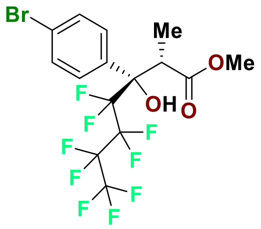


(2*S*,3*S*)-39

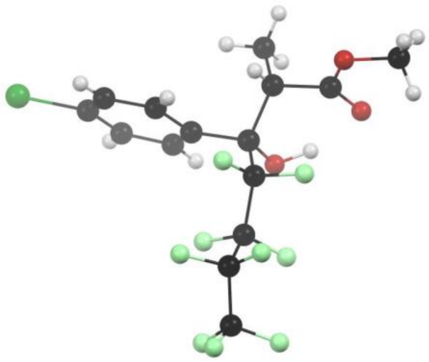

## 8. References

1 a) D. S. B. Daniels, S. R. Smith, T. Lebl, P. Shapland and A. D. Smith, *Synthesis*, **2015**, *47*, 34–41; b) L. C. Morrill, J. Douglas, T. Lebl, A. M. Z. Slawin, D. J. Fox and A. D. Smith, *Chem. Sci.*, **2013**, *4*, 4146–4155.

2 J. Crimmin, P.J. O´Hanlon and N.H. Rogers, *J. Chem. Soc. Perkin Trans*, **1985**, 541–548.

3 A. T. Davies, A. M. Z. Slawin and A. D. Smith, *Chem. Eur. J.*, **2015**, *21*, 18944–18948.

4 A. V.Shtelman and J. Y.Becker, *Tetrahedron Letters*, **2008**, *49*, 3101–3103.

5 M. Bellasoud, J.E. Dubous and E. Bertounesque, *Synthetic Communications: An International Journal for Rapid Communication of Synthetic Organic Chemistry*, **1987**, 1811.

6 Y. Nakagawa, S. Chanthamath, I. Fujisawa, K. Shibatomi and S. Iwasa, *Chem. Commun.,* **2017**, *53*, 3753–3756.

7 A. V.Shtelman and J. Y.Becker, *Tetrahedron*, **2011**, *67*, 1135–1141.

8 P. A. Grieco, C. J. Wang and S. D. Burke, *J. Chem. Soc*., *Chem. Commun*., **1975**, 537–538.

9 P. F. Hudrlik and D. Peterson, *J. Am. Chem. Soc*., **1975**, *97*, 6, 1464–1468.

10 A. J. Blake, C. L. Friend, R. J. Outram, N. S. Simpkins and A. J. Whitehead, *Tetrahedron Letters*, **2001**, *42*, 2877–2881.

11 C. Lambert, K. Utimoto and H. Nozaki, *Tetrahedron Letters*, **1984**, *25*, 5323–5326.

12 W. T. Brady and T. C. Cheng, *Journal of Organometalic Chemistry*, **1977**, *137*, 287–292.

13 P.-P. Yeh, D. S. B. Daniels, D. B. Cordes, A. M. Z. Slawin and A. D. Smith, *Org. Lett.*, **2014**, *16*, 964–967.

14 A. T. Davies, P. M. Pickett, A. M. Z. Slawin and A. D. Smith, *ACS Catal.*, **2014**, *4*, 2696–2700.

15 D. Barrios Antunez, M. D. Greenhalgh, A. C. Brueckner, D. M. Walden, P. Elías-Rodríguez, P. Roberts, B. Young, T. W. West, A. M. Z. Slawin, P. H-Y. Cheong and A. D. Smith, *Chem. Sci.*, **2019**, *10*, 6162–6173.

16 Rigaku Americas, *The Woodlands, Texas, USA*, and Rigaku Corporation, *Tokyo, Japan*, *CrystalClear-SM Exper*t v2.1. **2015**.

*17 Rigaku Oxford Diffraction, Rigaku Corporation, Oxford, U.K., CrysAlisPro v1.171.38.46, v1.171.39.8d.* **2015***.*

18 Sheldrick, G. M. SHELXT – Integrated space-group and crystal structure determination. *Acta Crystallogr., Sect. A: Found. Adv*. **2015**, *71*, 3–8.

19 Beurskens, P. T. Beurskens, G. de Gelder, R. Garcia-Granda, S. Gould, R. O. Israel, R. Smits, J. M. M. Crystallography Laboratory, University of Nijmegen, The Netherlands, *DIRDIF-99*. **1999**.

20 Sheldrick, G. M. Crystal structure refinement with SHELXL Acta Crystallogr., *Sect. C: Struct. Chem*. **2015**, *71*, 3–8.

21 *CrystalStructure v4.3.0.* Rigaku Americas, The Woodlands, Texas, USA, and Rigaku Corporation, Tokyo, Japan, **2018**.

## Appendix I. 1H, 19F and 13C{1H} NMR Spectra

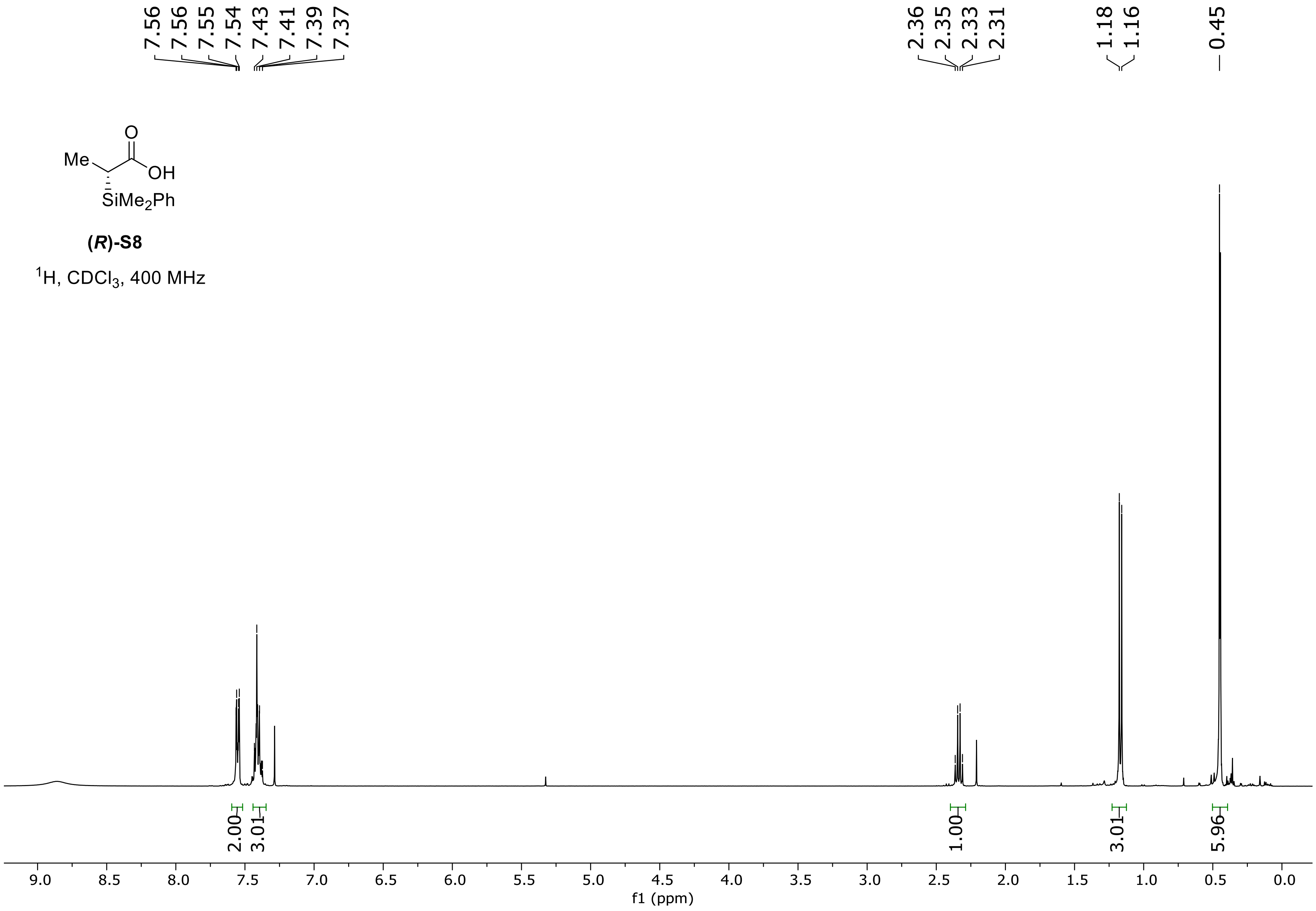

7.56
7.56
7.55
7.54
7.43
7.41
7.39
7.37
2.36
2.35
2.33
2.31
1.18
1.16
0.45
Me
O
OH
SiMe2Ph
(R)-S8
1H, CDCl3, 400 MHz
2.00
3.01
1.00
3.01
5.96
9.0
8.5
8.0
7.5
7.0
6.5
6.0
5.5
5.0
4.5
4.0
3.5
3.0
2.5
2.0
1.5
1.0
0.5
0.0
f1 (ppm)

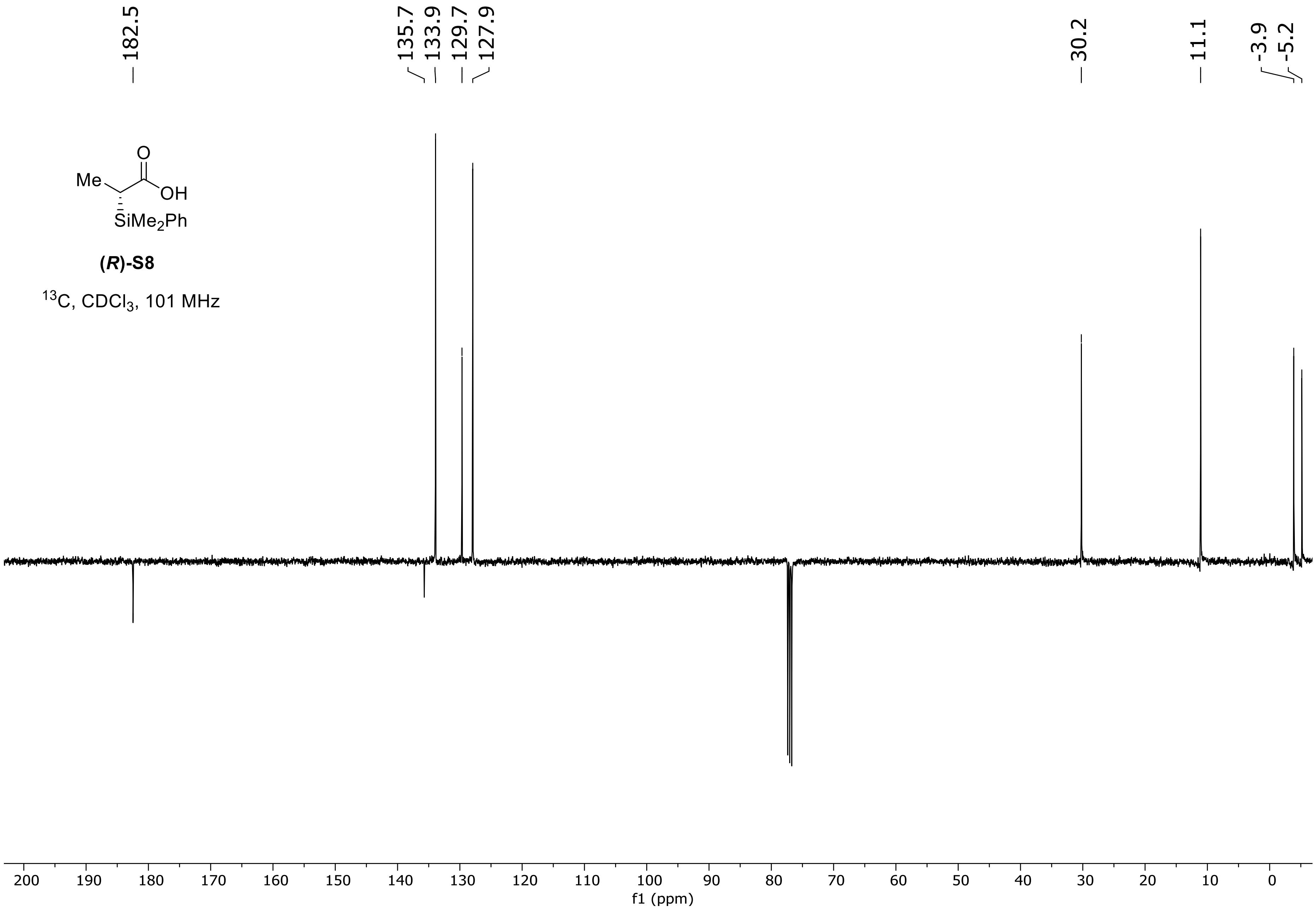


**(*R*)-S8**

$^{13}C$, $CDCl_3$, 101 MHz

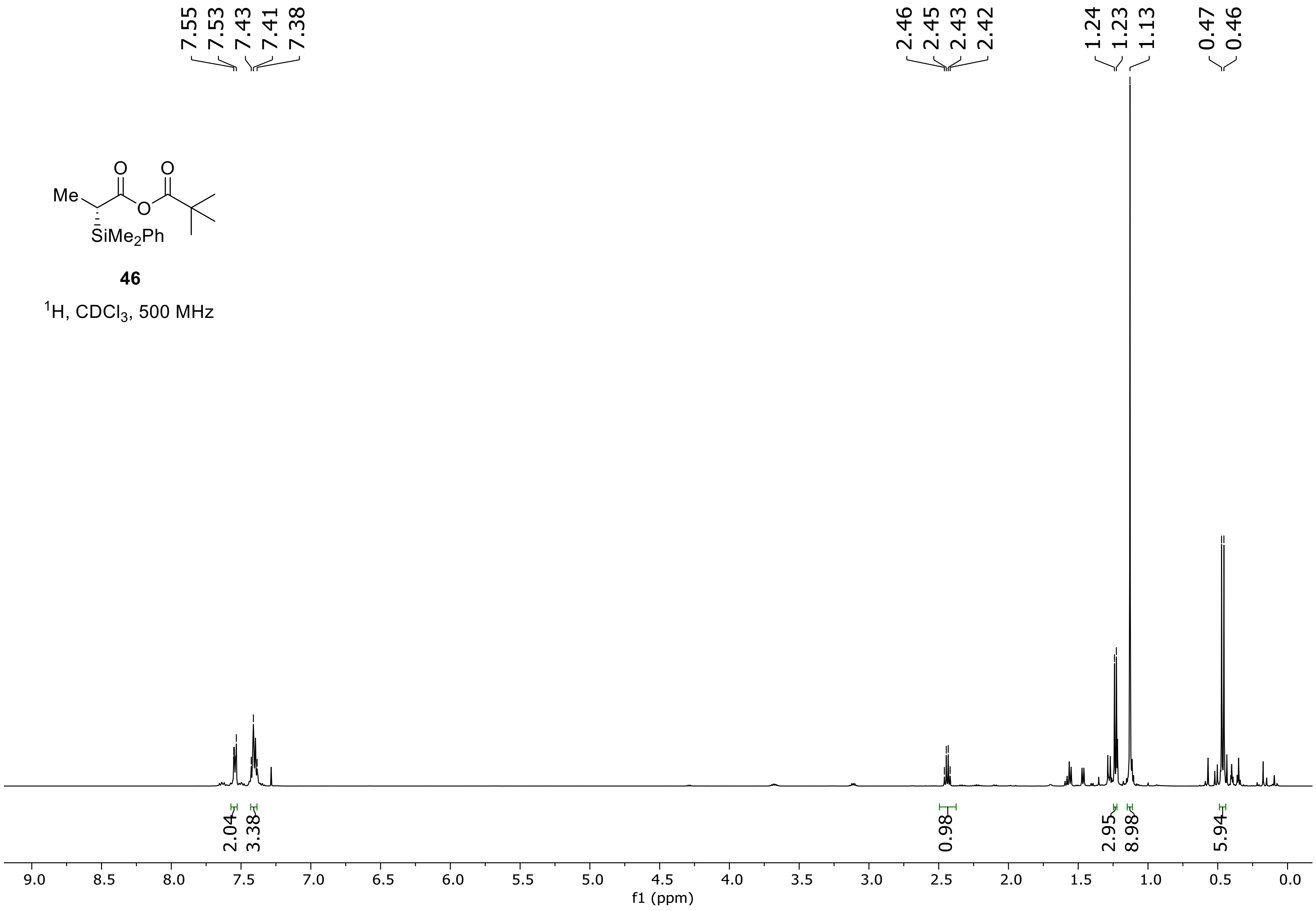
7.55
7.53
7.43
7.41
7.38
2.46
2.45
2.43
2.42
1.24
1.23
1.13
0.47
0.46
Me
O
O
O
SiMe2Ph
46
1H, CDCl3, 500 MHz
2.04
3.38
0.98
2.95
8.98
5.94
9.0 8.5 8.0 7.5 7.0 6.5 6.0 5.5 5.0 4.5 4.0 3.5 3.0 2.5 2.0 1.5 1.0 0.5 0.0
f1 (ppm)

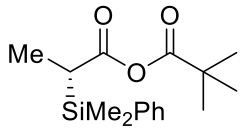

**46**

$^{13}C$, $CDCl_3$, 126 MHz

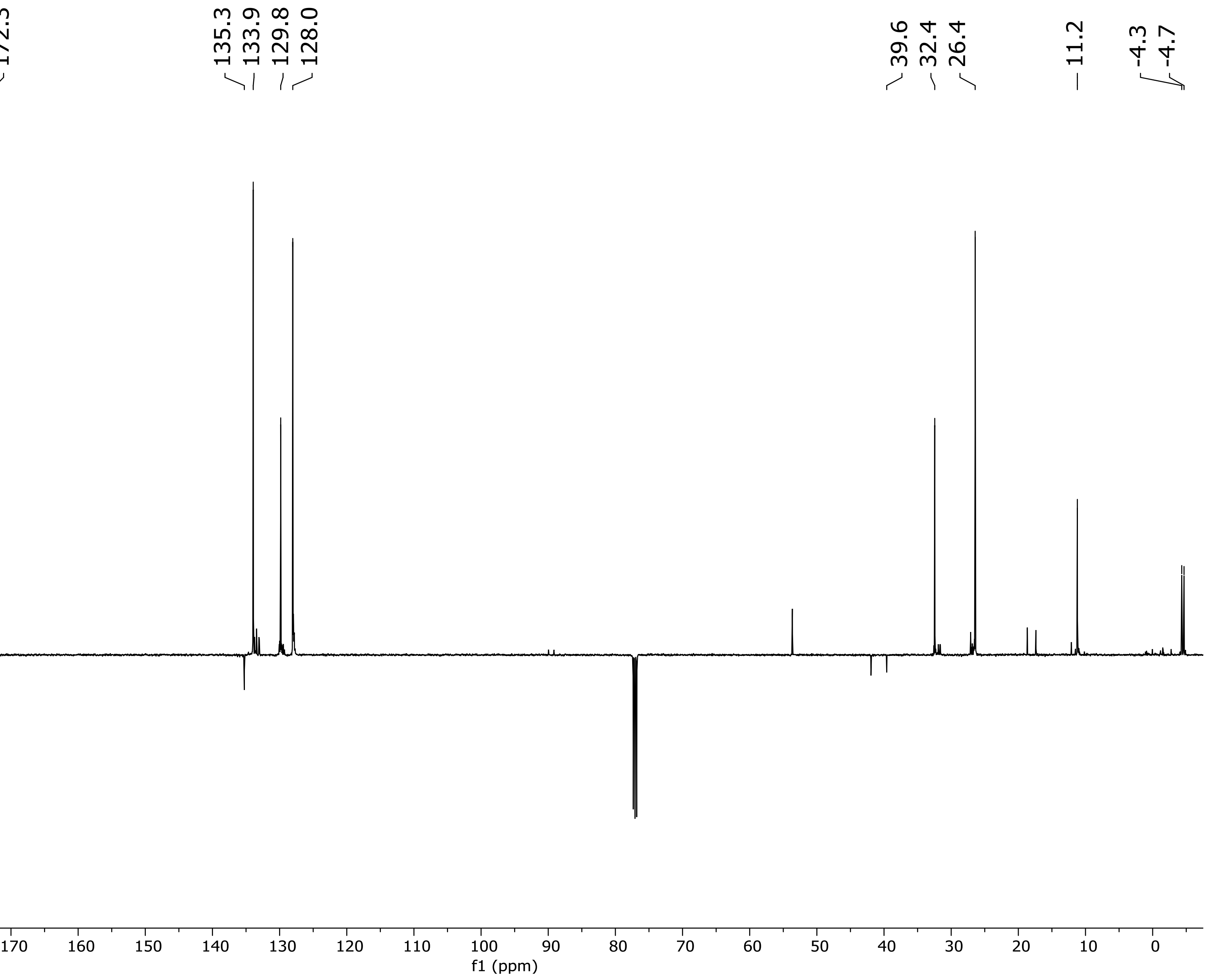

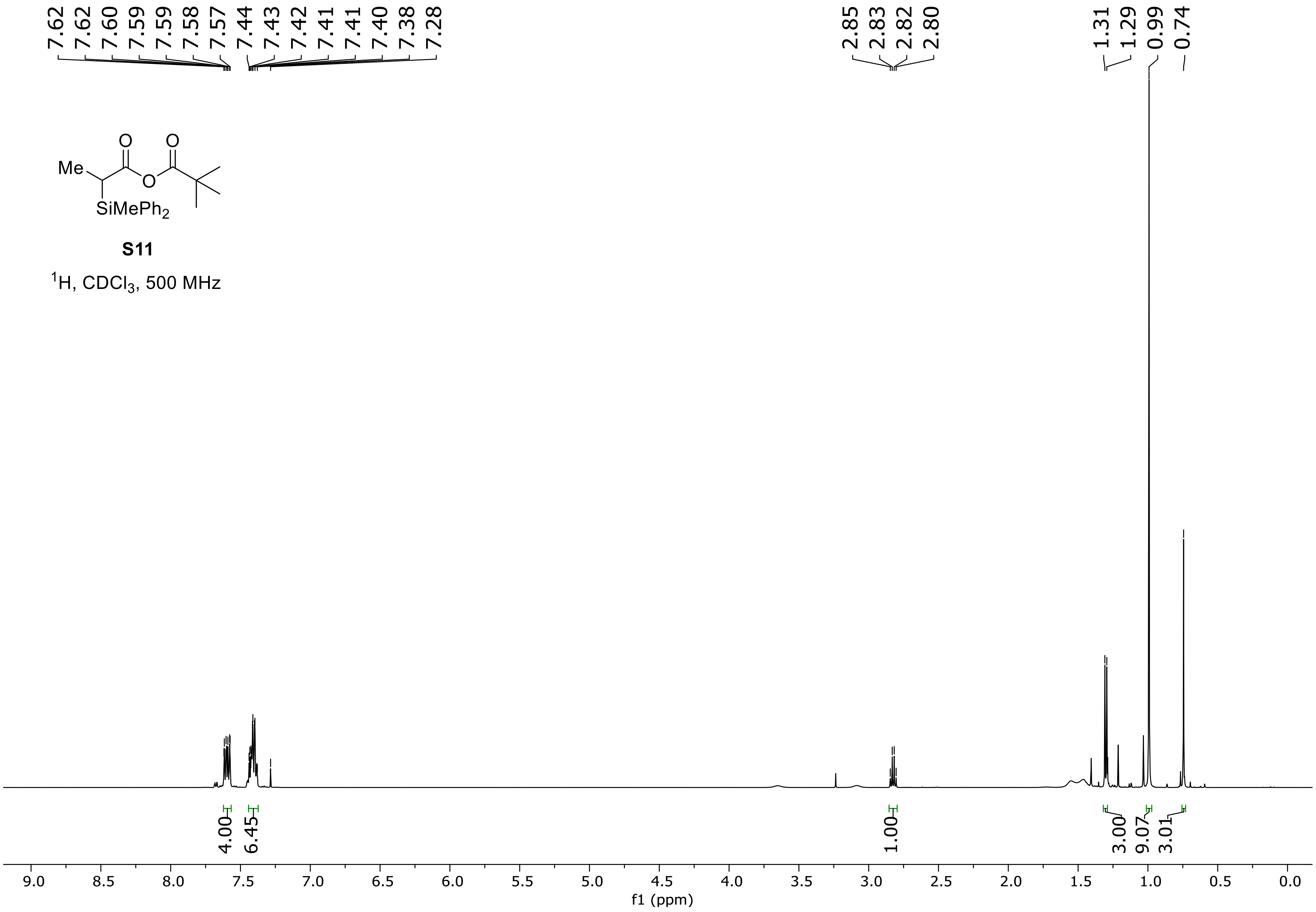
7.62
7.62
7.60
7.59
7.59
7.58
7.57
7.44
7.43
7.42
7.41
7.41
7.40
7.38
7.28
2.85
2.83
2.82
2.80
1.31
1.29
0.99
0.74
Me
O
O
O
SiMePh2
S11
1H, CDCl3, 500 MHz
4.00
6.45
1.00
3.00
9.07
3.01
9.0 8.5 8.0 7.5 7.0 6.5 6.0 5.5 5.0 4.5 4.0 3.5 3.0 2.5 2.0 1.5 1.0 0.5 0.0
f1 (ppm)

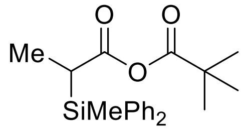


**S11**

$^{13}$C, $CDCl_3$, 126 MHz

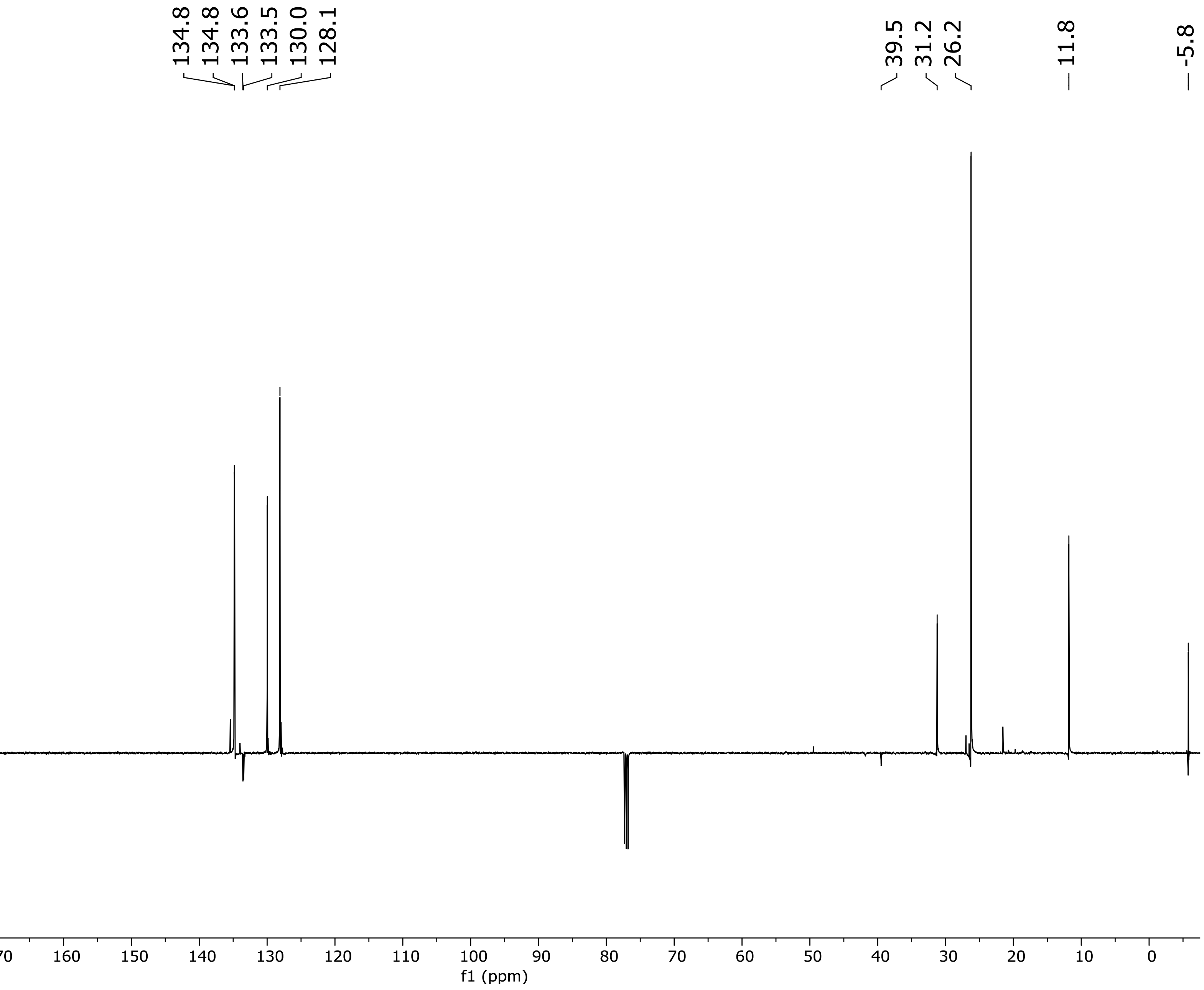

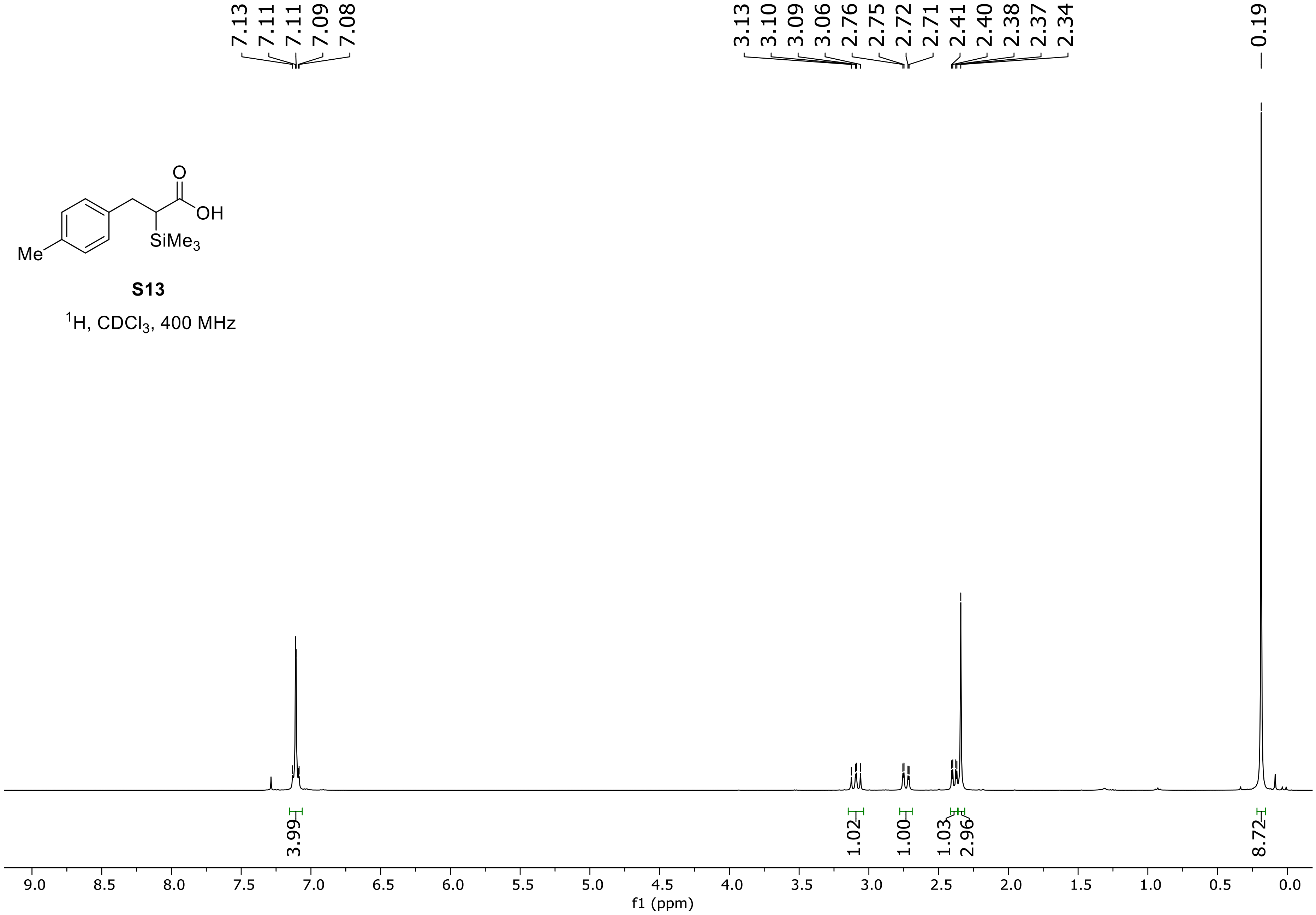


**S13**

$^1H$, $CDCl_3$, 400 MHz

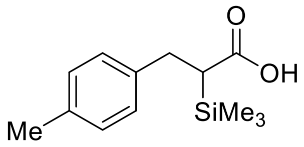

**S13**

$^{13}C$, $CDCl_3$, 101 MHz

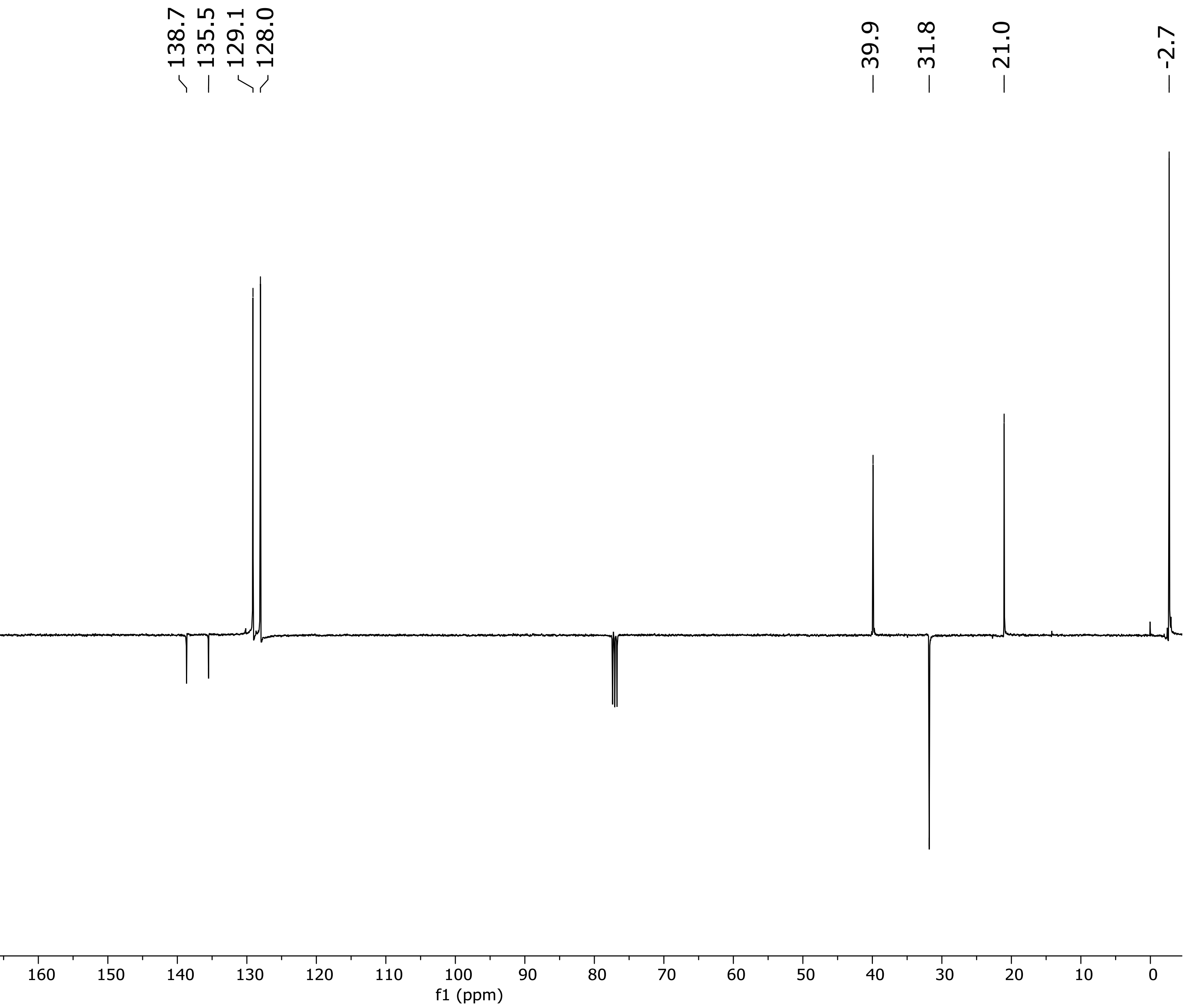

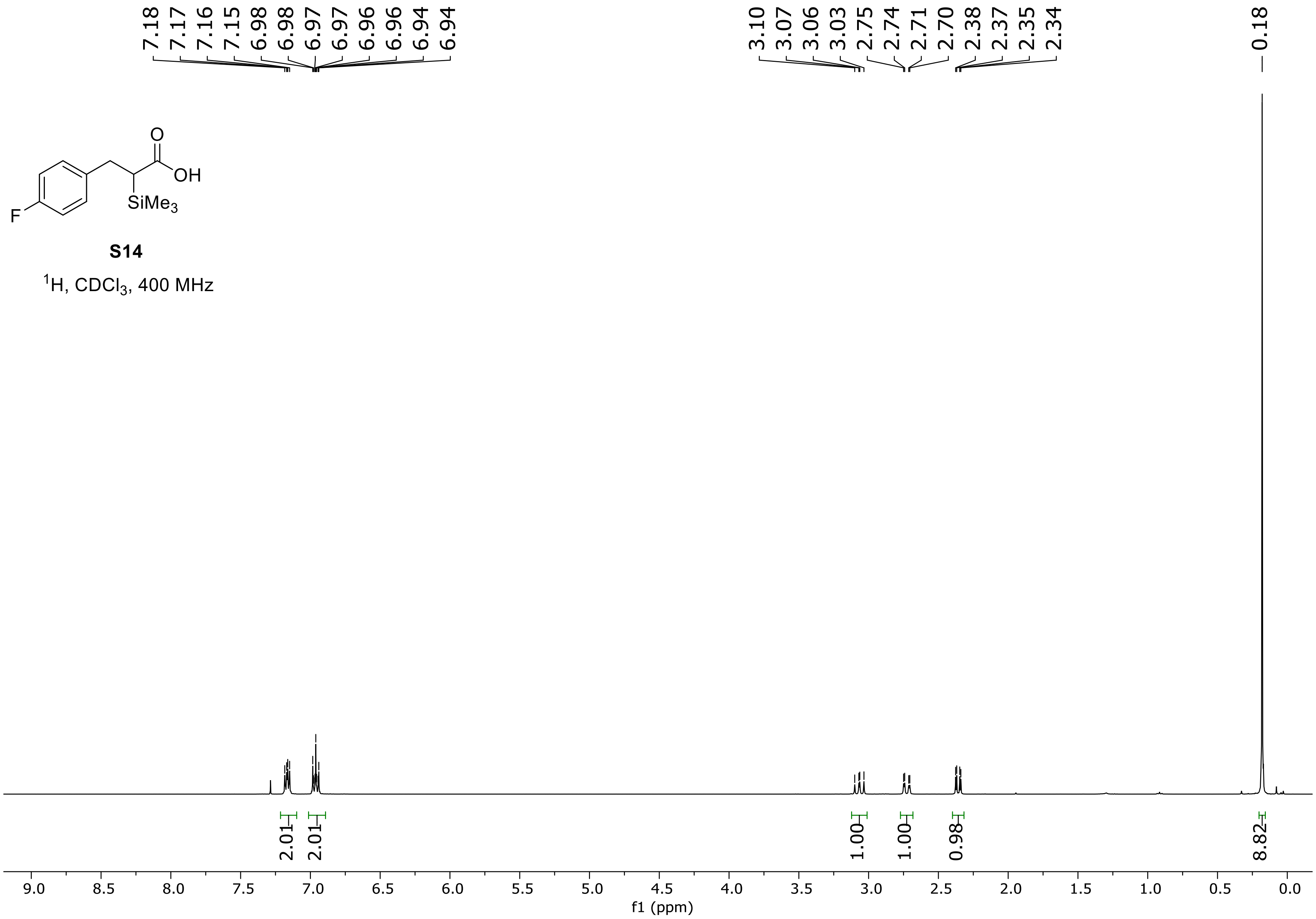


**S14**

$^{1}$H, $CDCl_3$, 400 MHz

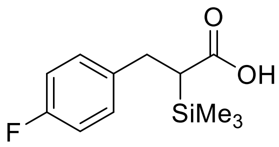

**S14**

$^{19}F$, $CDCl_3$, 377 MHz

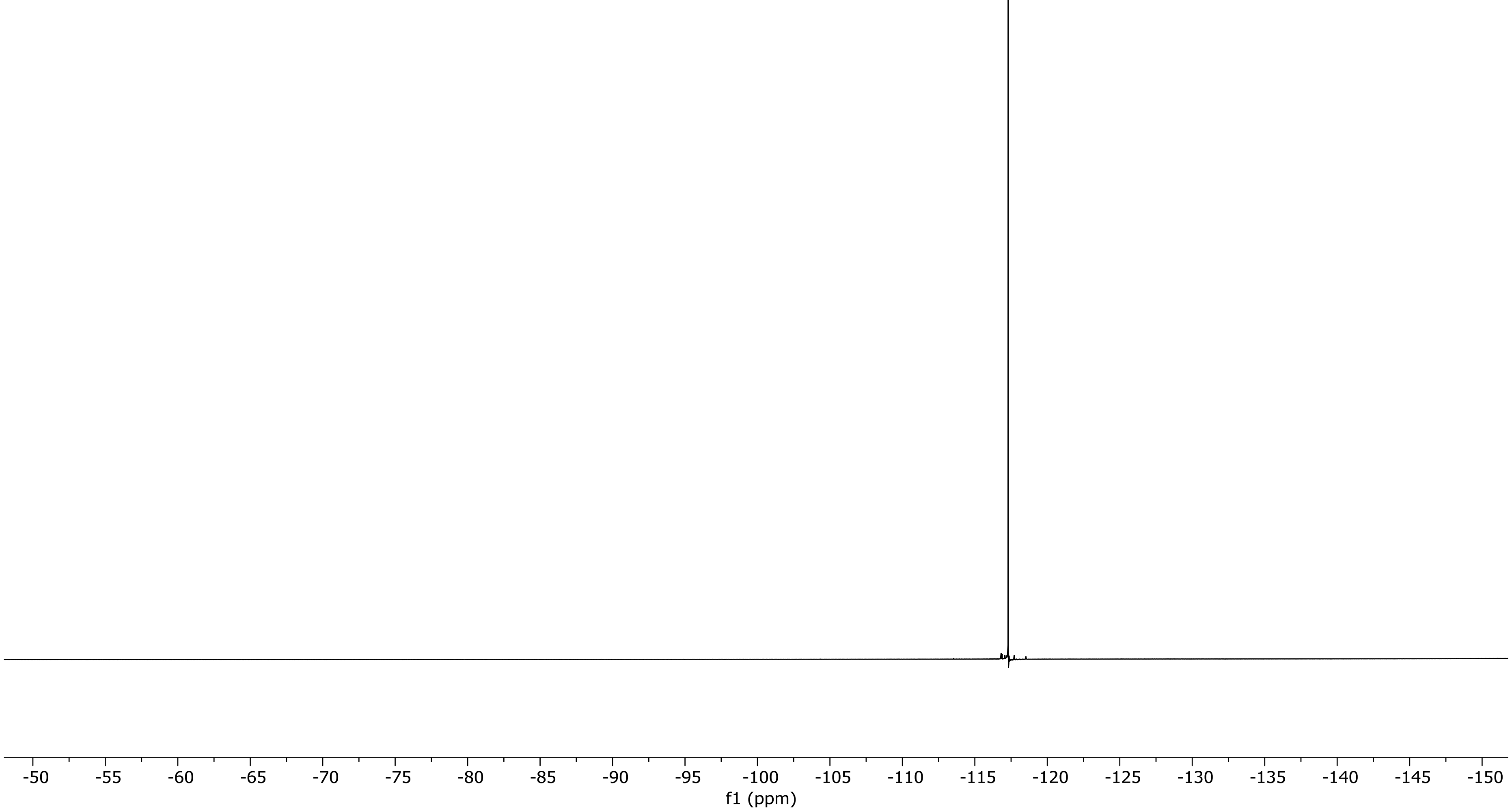

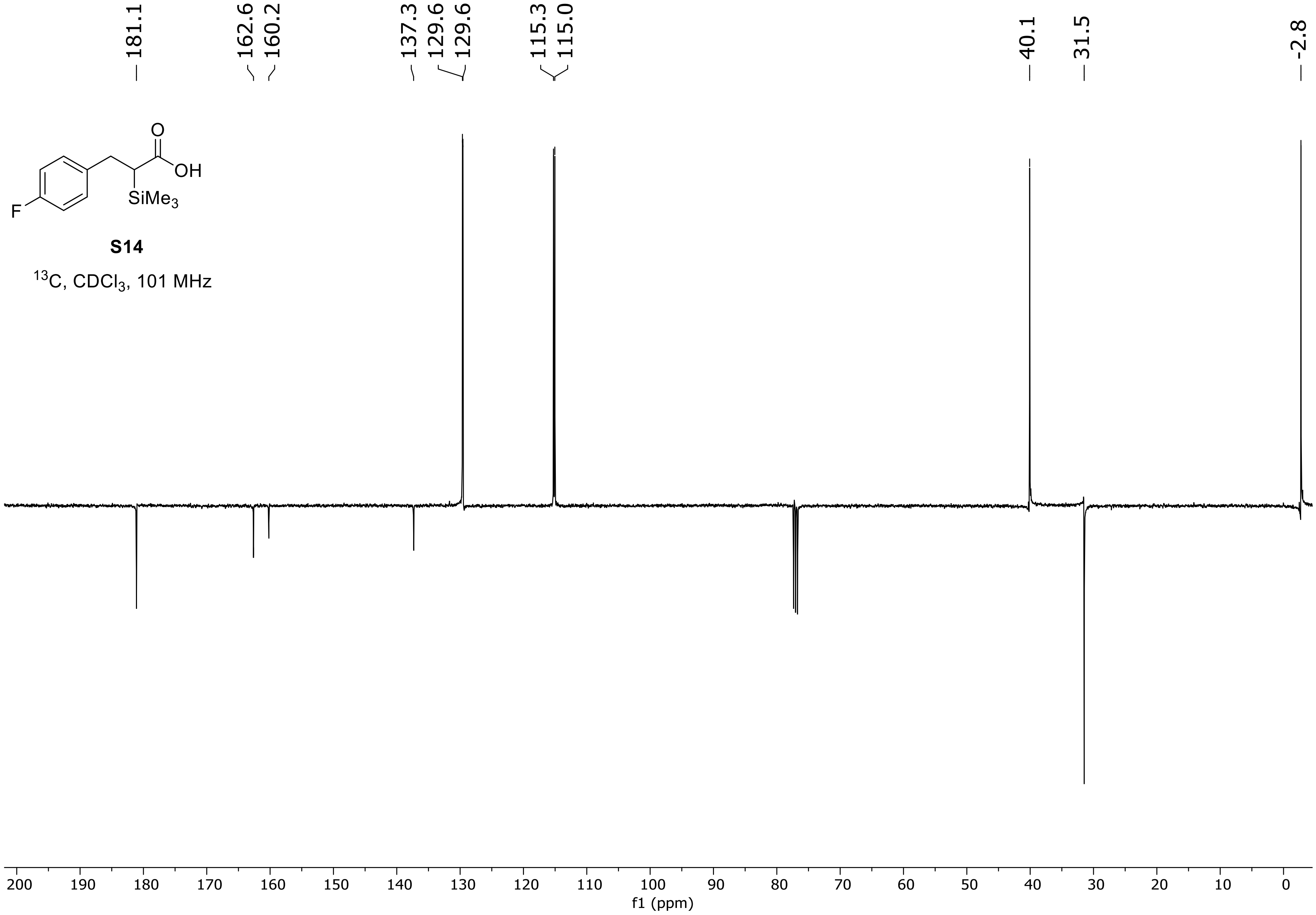
181.1
162.6
160.2
137.3
129.6
129.6
115.3
115.0
40.1
31.5
-2.8
O
OH
SiMe3
F
S14
13C, CDCl3, 101 MHz
200 190 180 170 160 150 140 130 120 110 100 90 80 70 60 50 40 30 20 10 0
f1 (ppm)

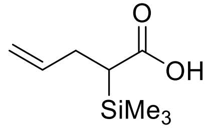

**S18**

$^1H$, $CDCl_3$, 400 MHz

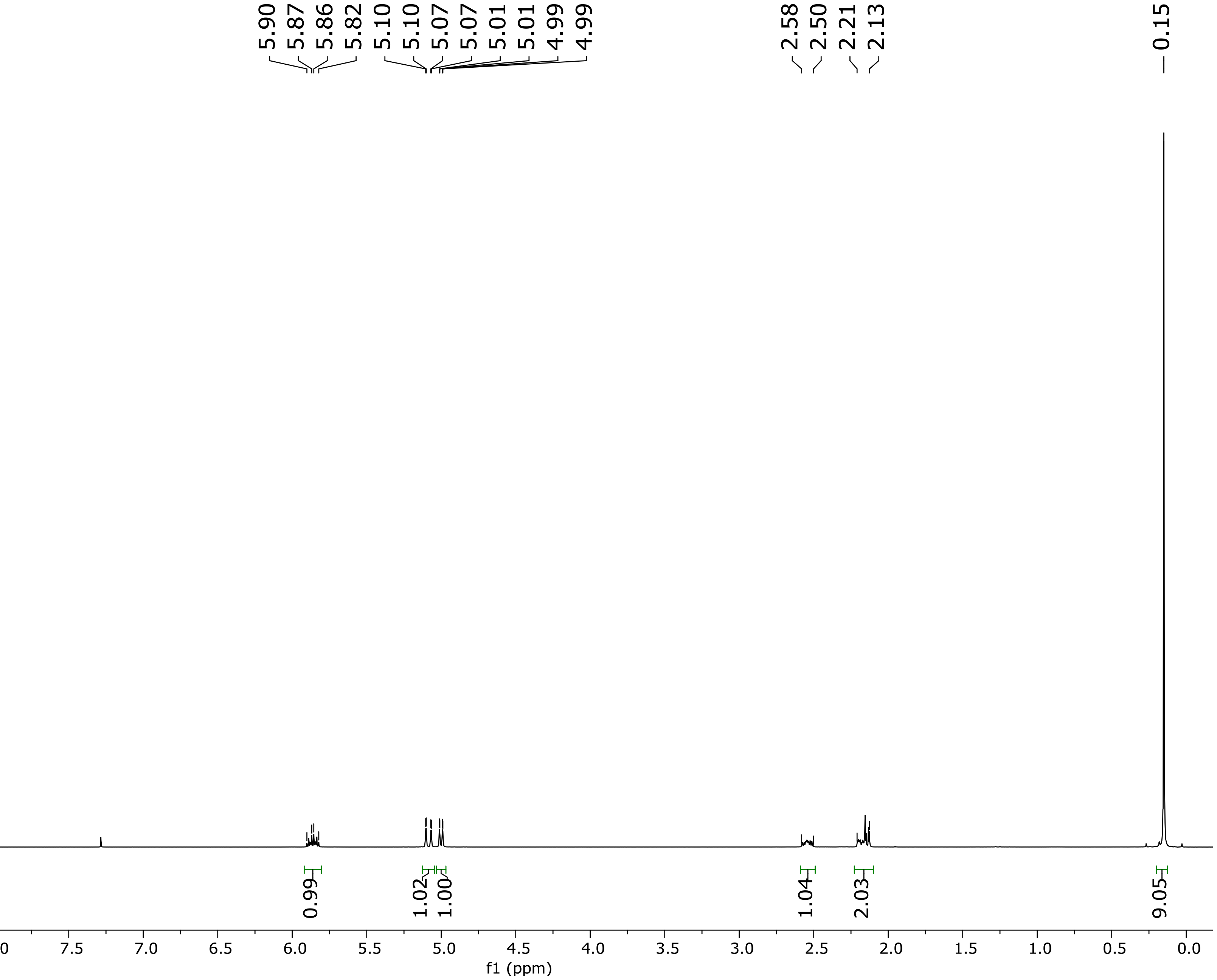

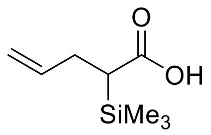


**S18**

$^{13}$C, $CDCl_3$, 126 MHz

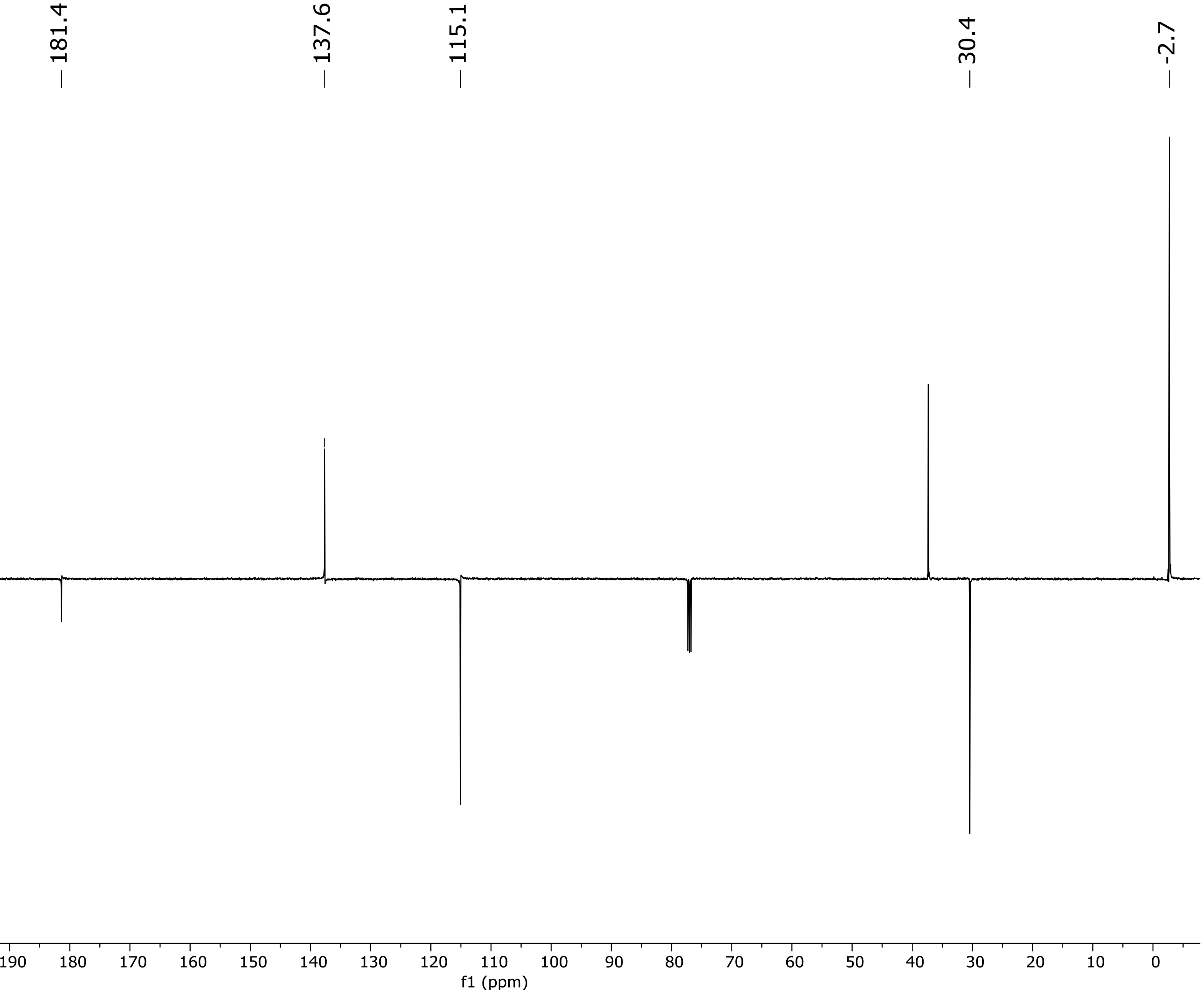

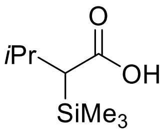


**S20**

$^{1}H$, $CDCl_3$, 300 MHz

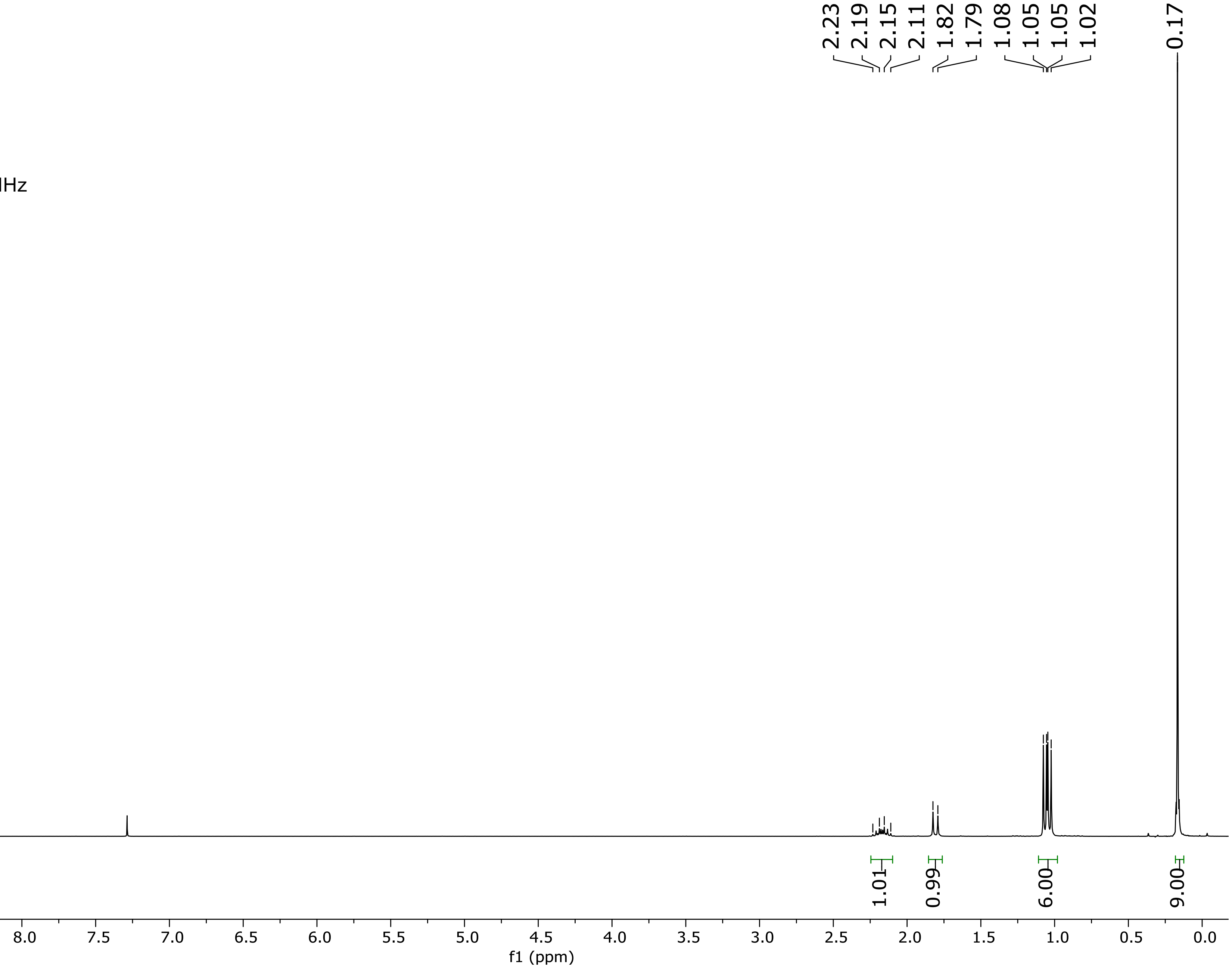

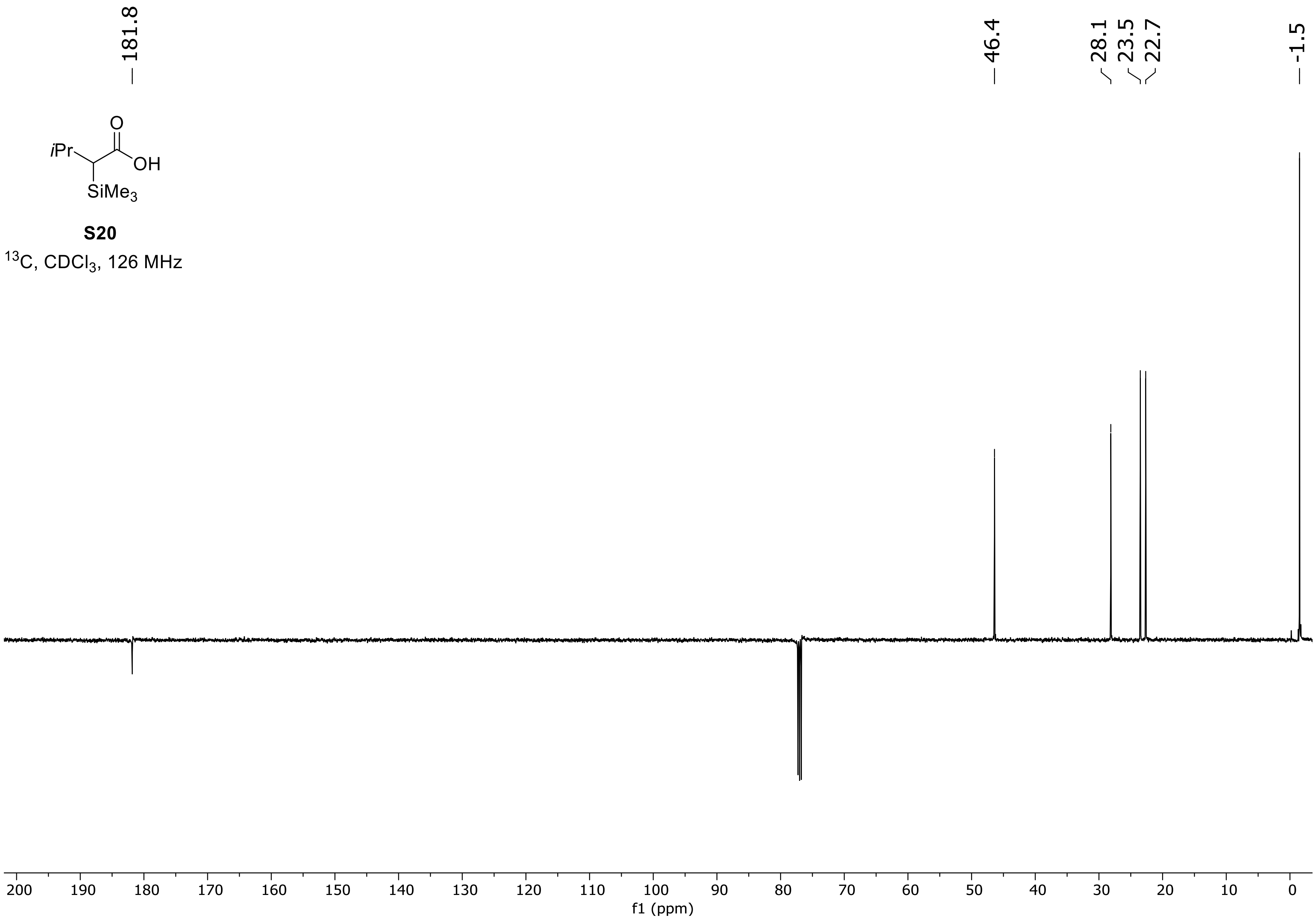


**S20**

$^{13}C$, $CDCl_3$, 126 MHz

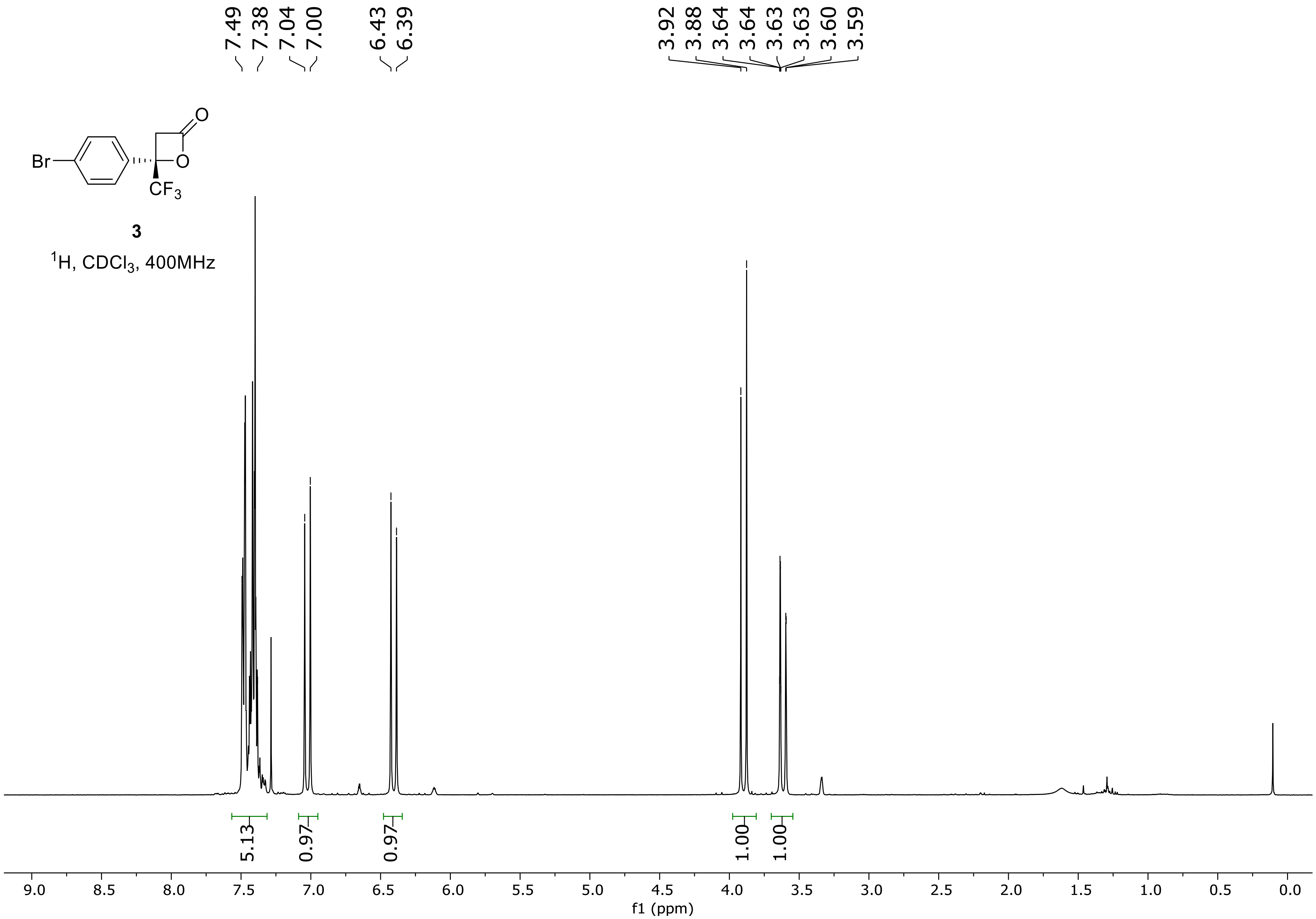
7.49
7.38
7.04
7.00
6.43
6.39
3.92
3.88
3.64
3.64
3.63
3.63
3.60
3.59
Br
O
O
CF3
3
1H, CDCl3, 400MHz
5.13
0.97
0.97
1.00
1.00
9.0
8.5
8.0
7.5
7.0
6.5
6.0
5.5
5.0
4.5
4.0
3.5
3.0
2.5
2.0
1.5
1.0
0.5
0.0
f1 (ppm)

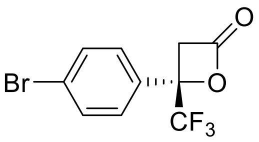

**3**

$^{19}F$, $CDCl_3$, 376 MHz

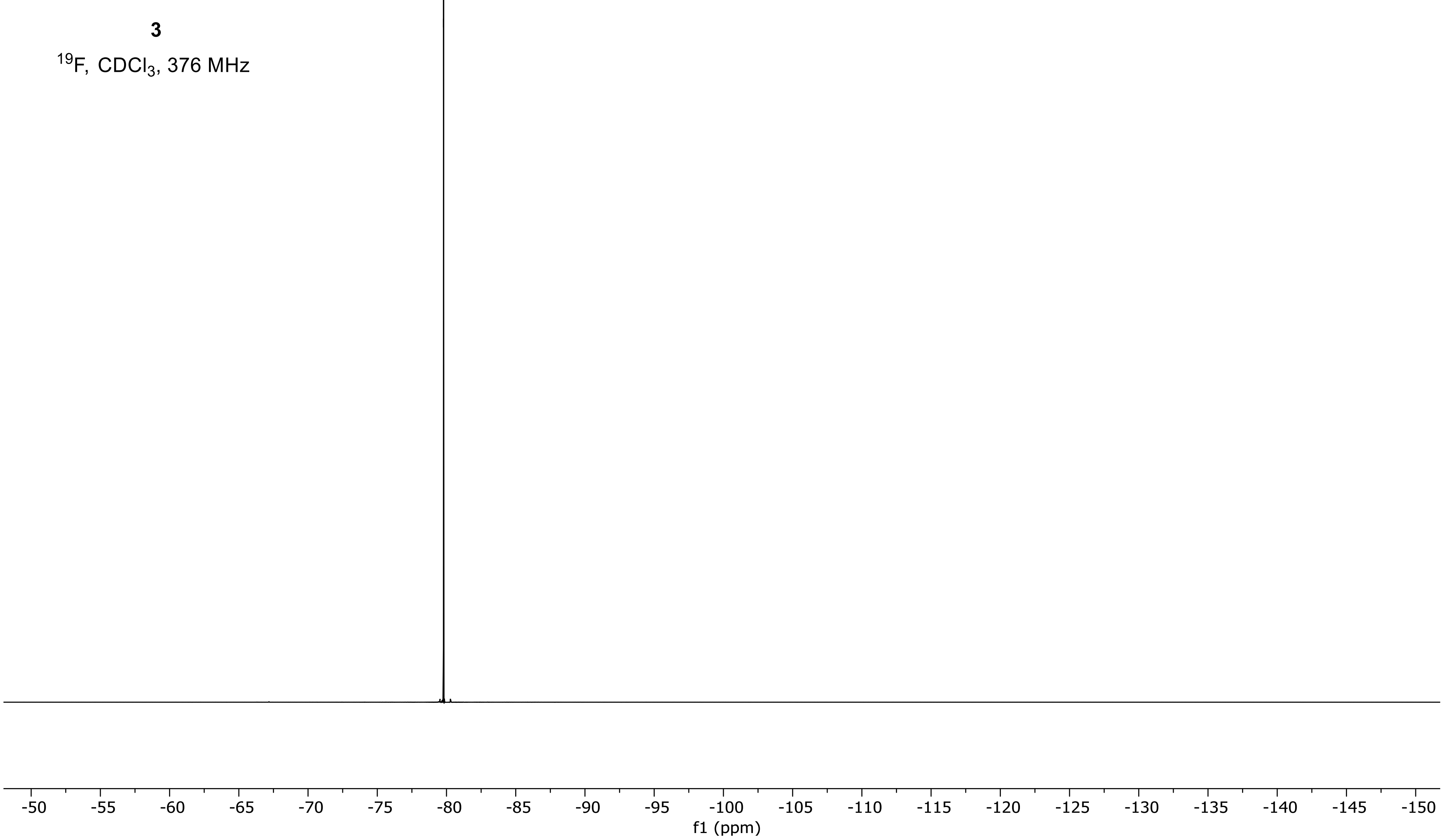

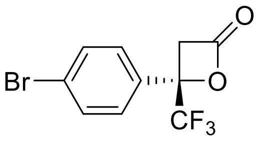

**3**

$^{13}C$, $CDCl_3$, 126 MHz

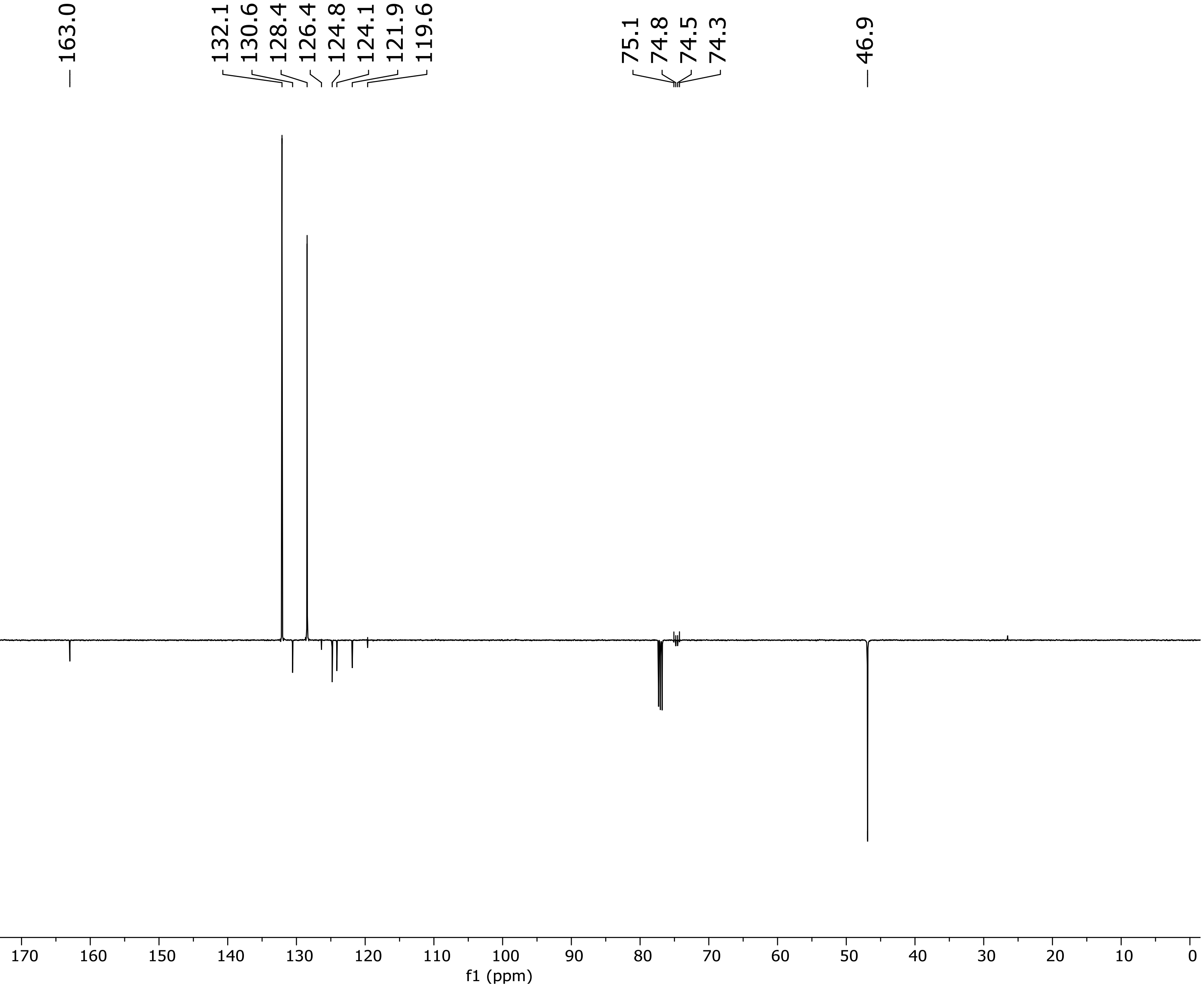

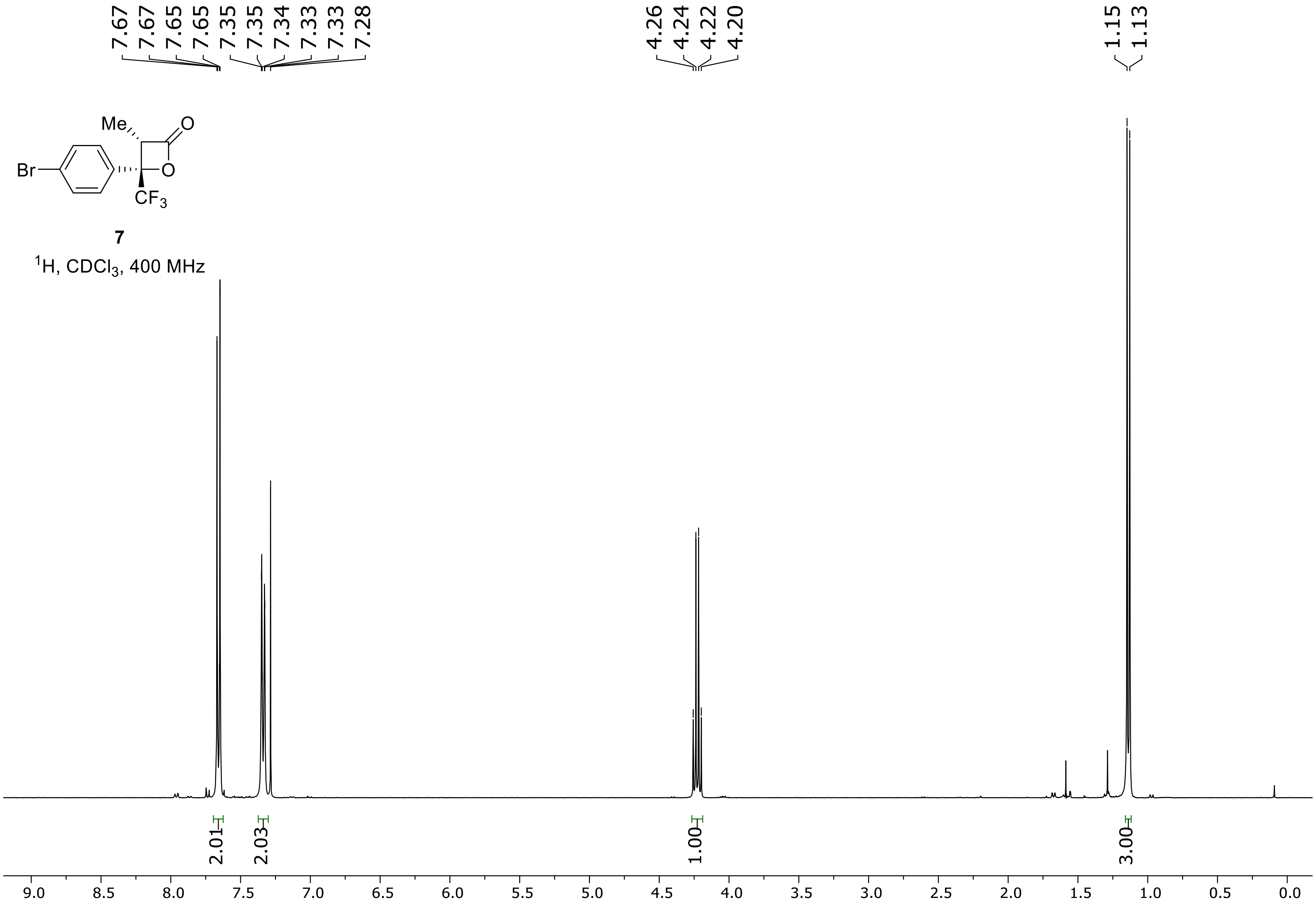


**7**

$^1$H, $CDCl_3$, 400 MHz

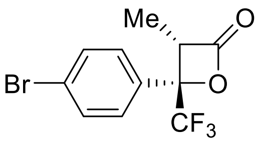

**7**

$^{19}F$, $CDCl_3$, 376 MHz

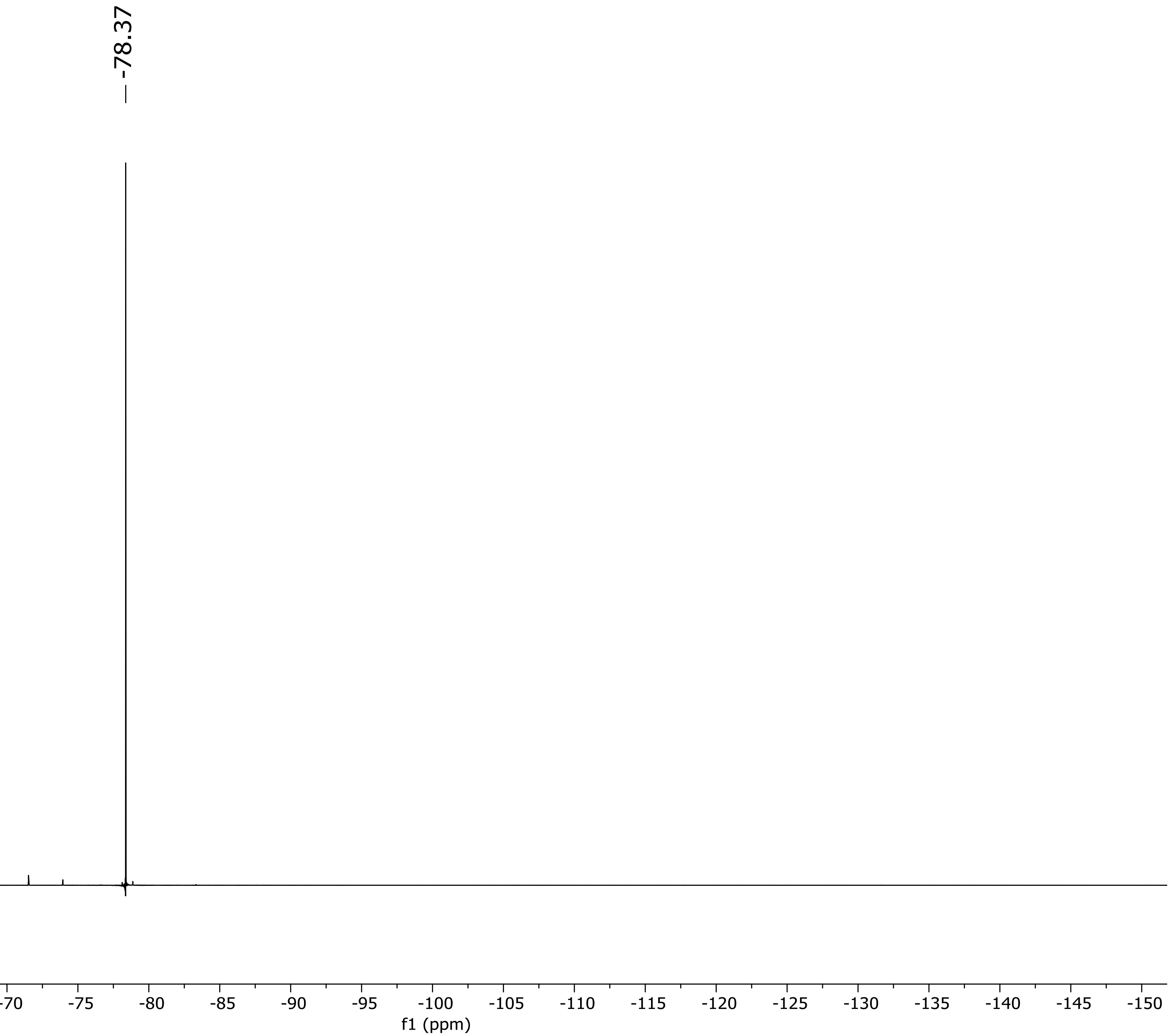

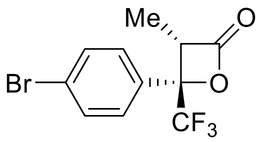

**7**

$^{13}C$, $CDCl_3$, 126 MHz

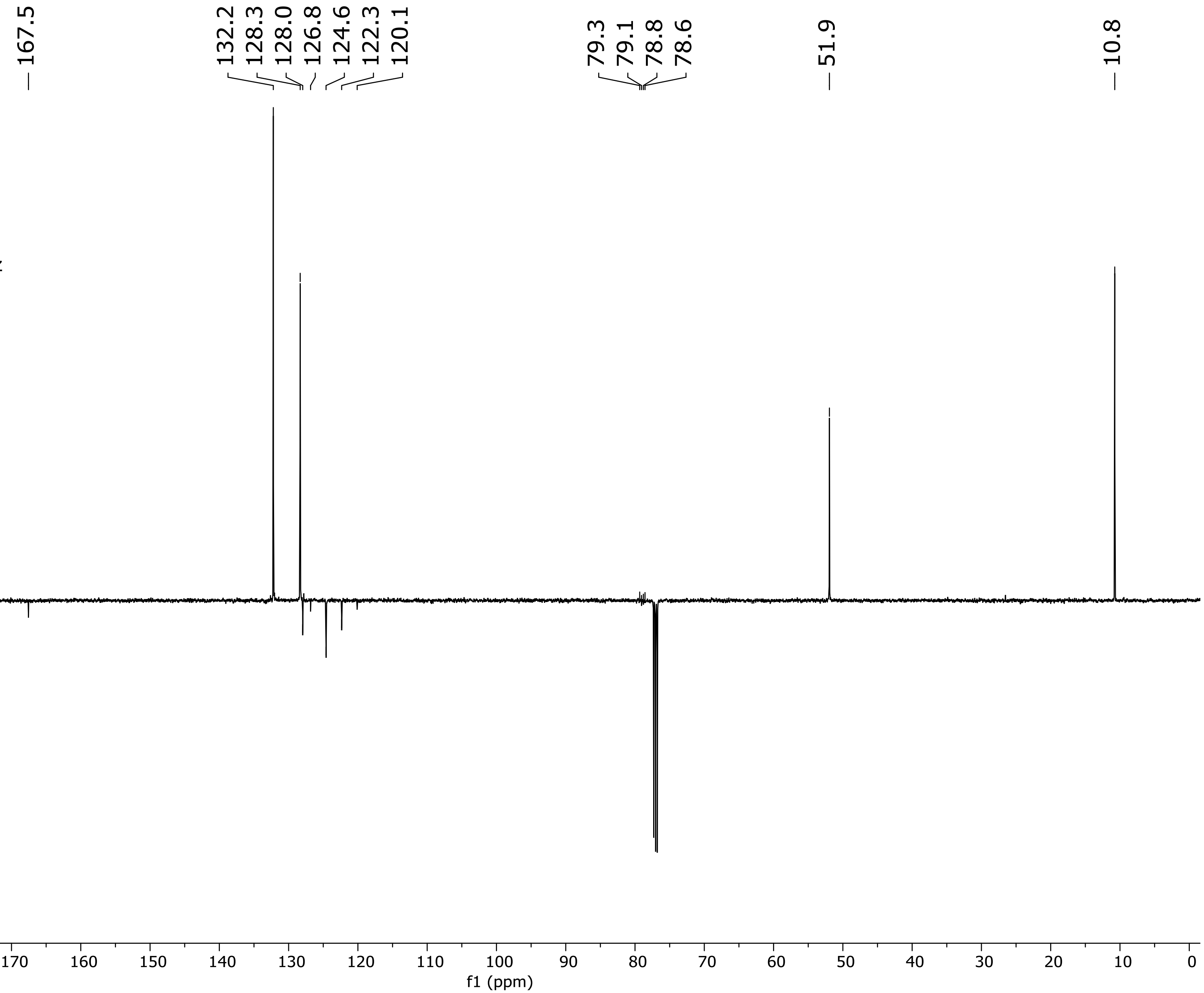

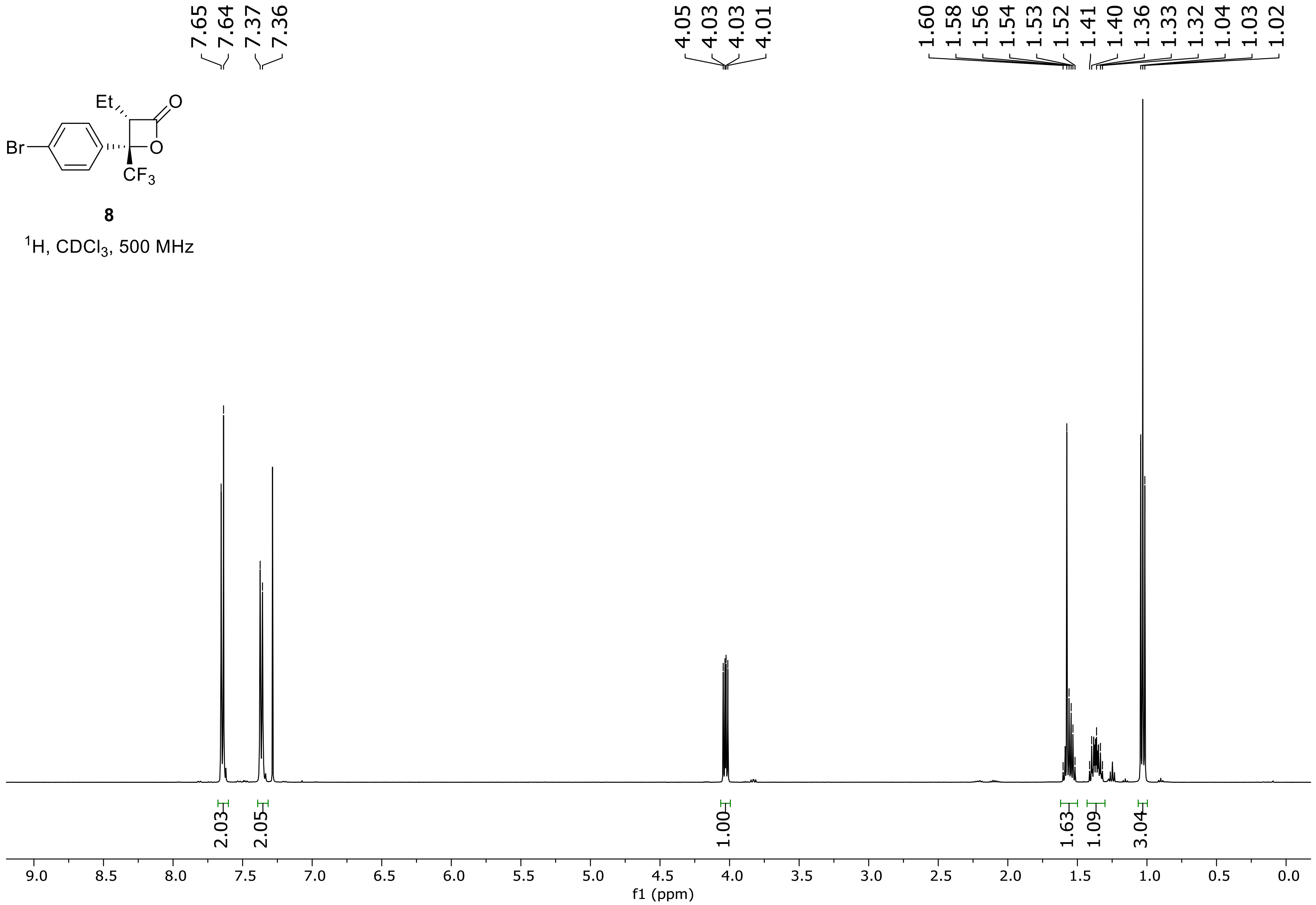

7.65
7.64
7.37
7.36
4.05
4.03
4.03
4.01
1.60
1.58
1.56
1.54
1.53
1.52
1.41
1.40
1.36
1.33
1.32
1.04
1.03
1.02
Et
O
Br
O
CF3
8
1H, CDCl3, 500 MHz
2.03
2.05
1.00
1.63
1.09
3.04
9.0
8.5
8.0
7.5
7.0
6.5
6.0
5.5
5.0
4.5
4.0
3.5
3.0
2.5
2.0
1.5
1.0
0.5
0.0
f1 (ppm)

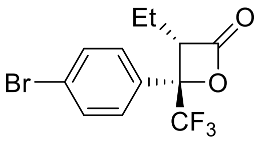

**8**

$^{19}F$, $CDCl_3$, 471 MHz

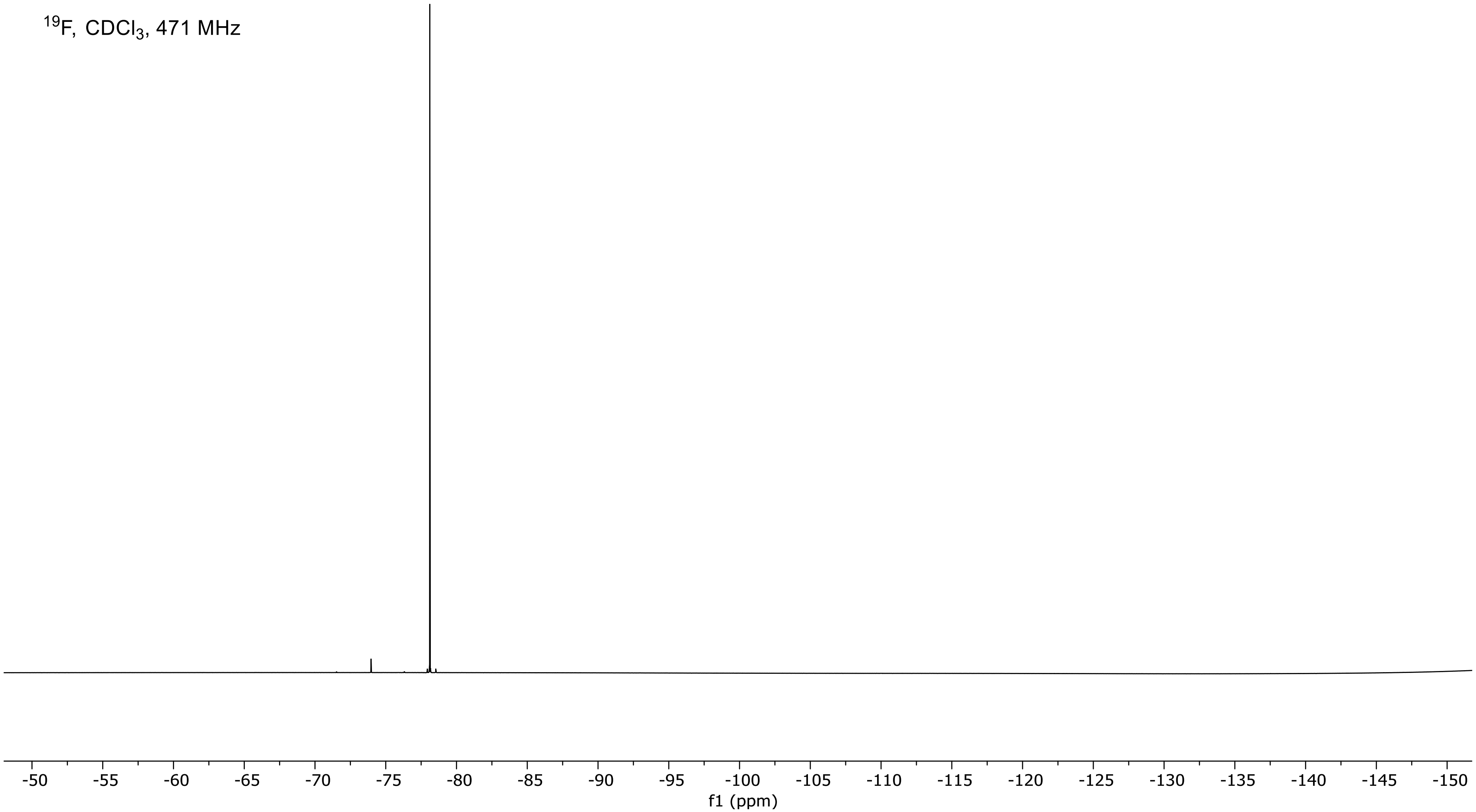

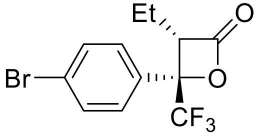

**8**

$^{13}C$, $CDCl_3$, 126 MHz

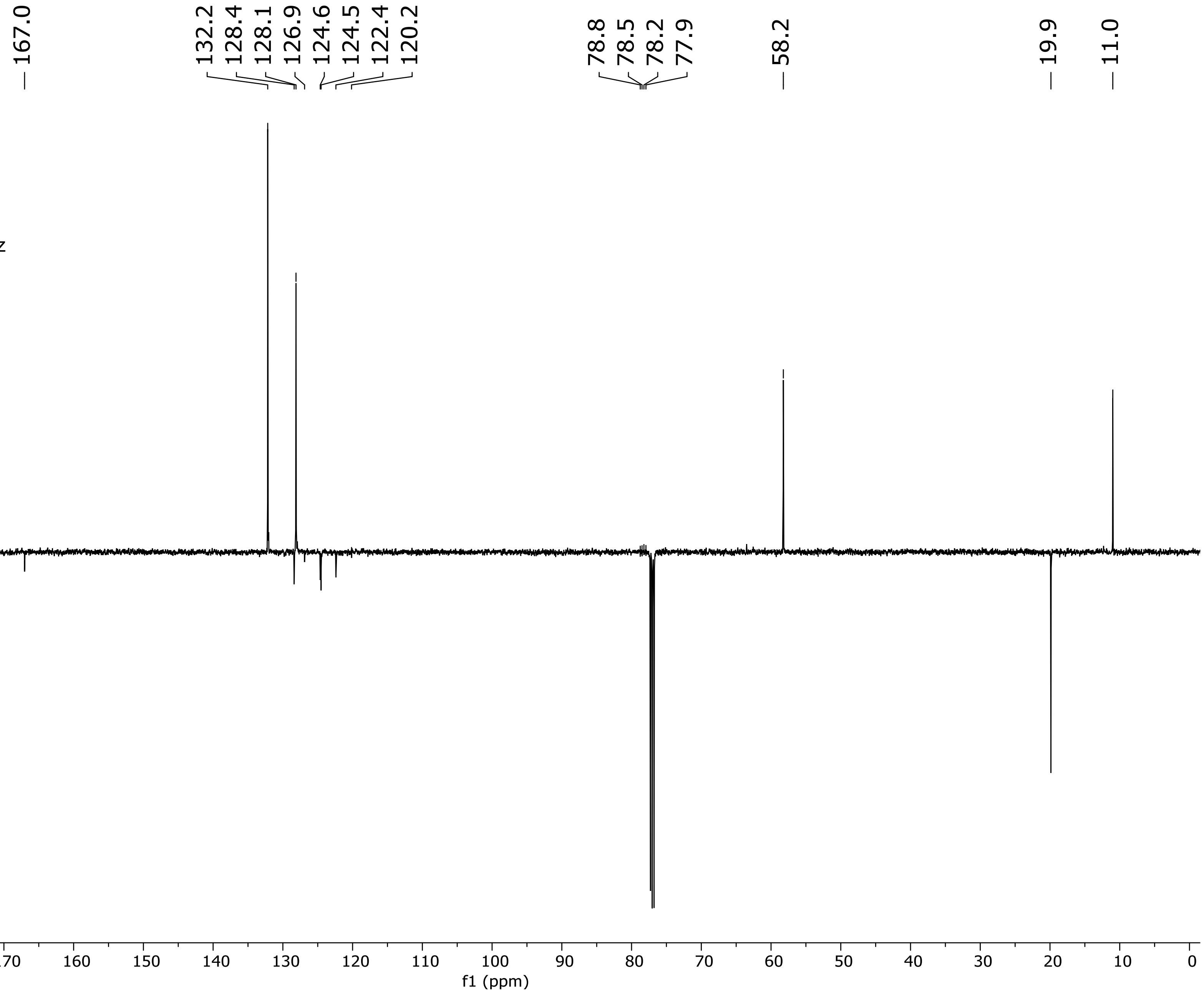

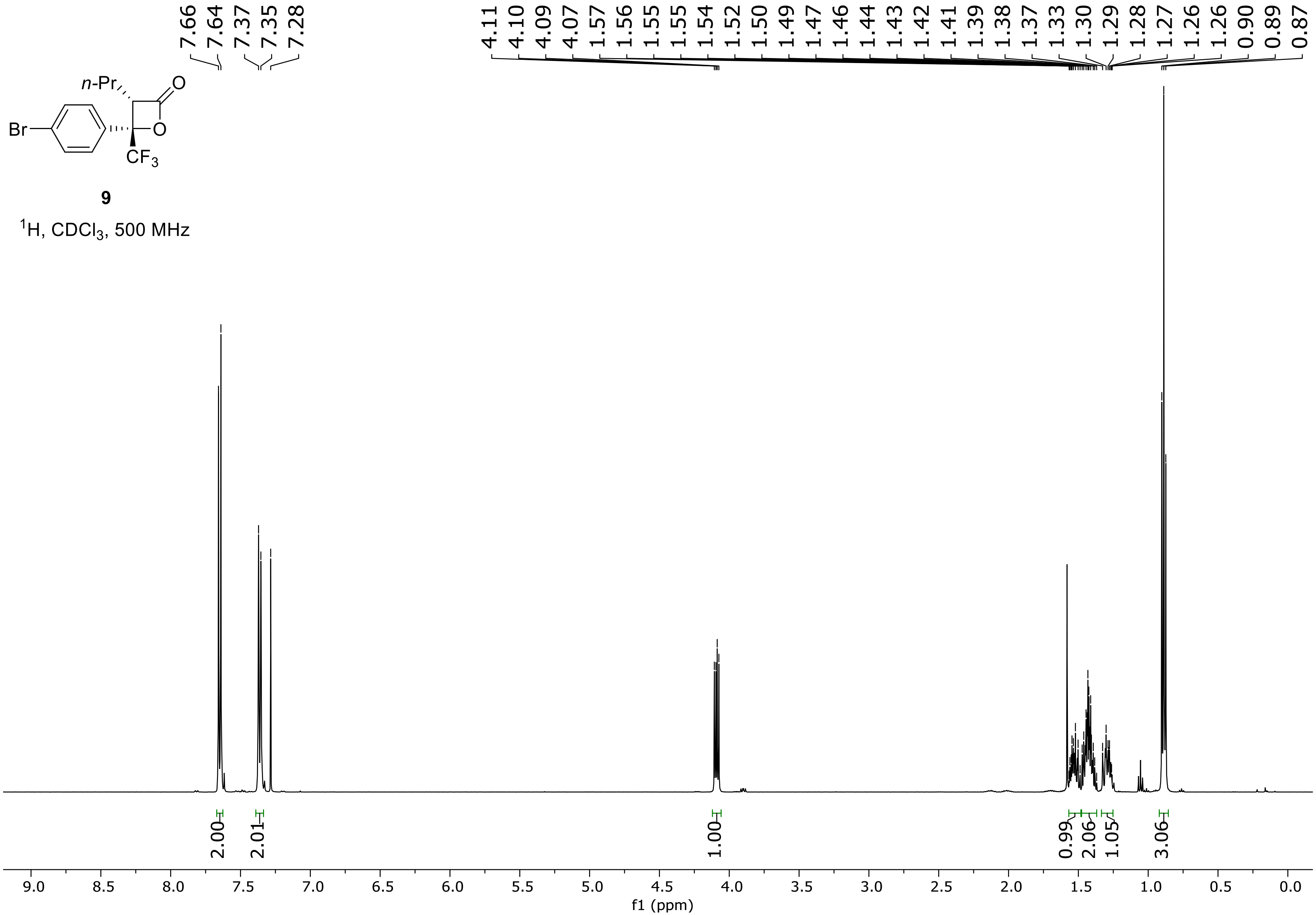


**9**

$^{1}$H, $CDCl_3$, 500 MHz

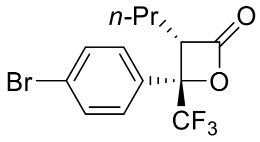

**9**

$^{19}F$, $CDCl_3$, 471 MHz

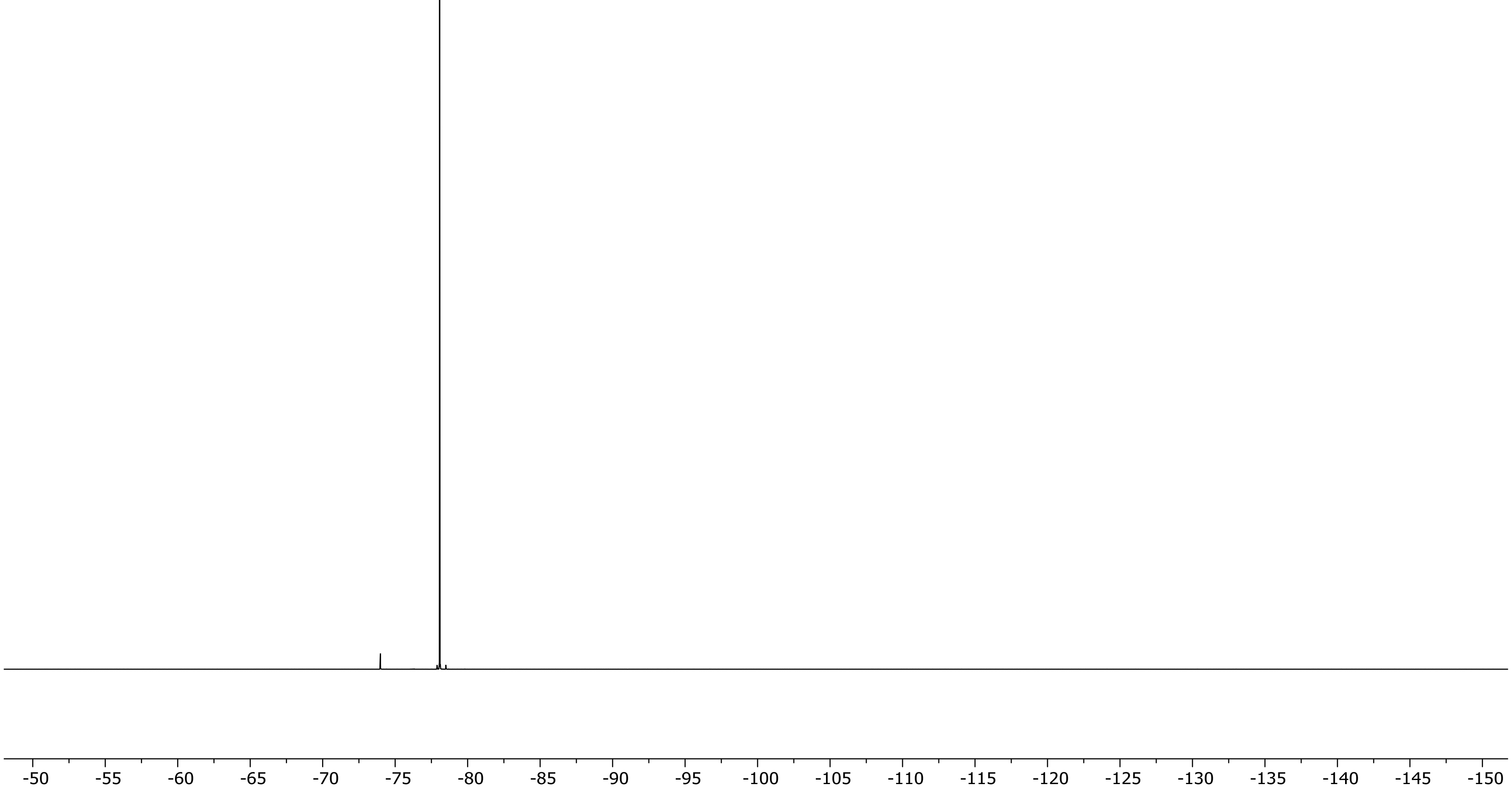

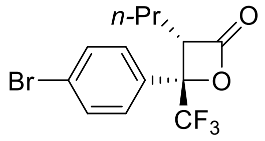


**9**

$^{13}C$, $CDCl_3$, 126 MHz

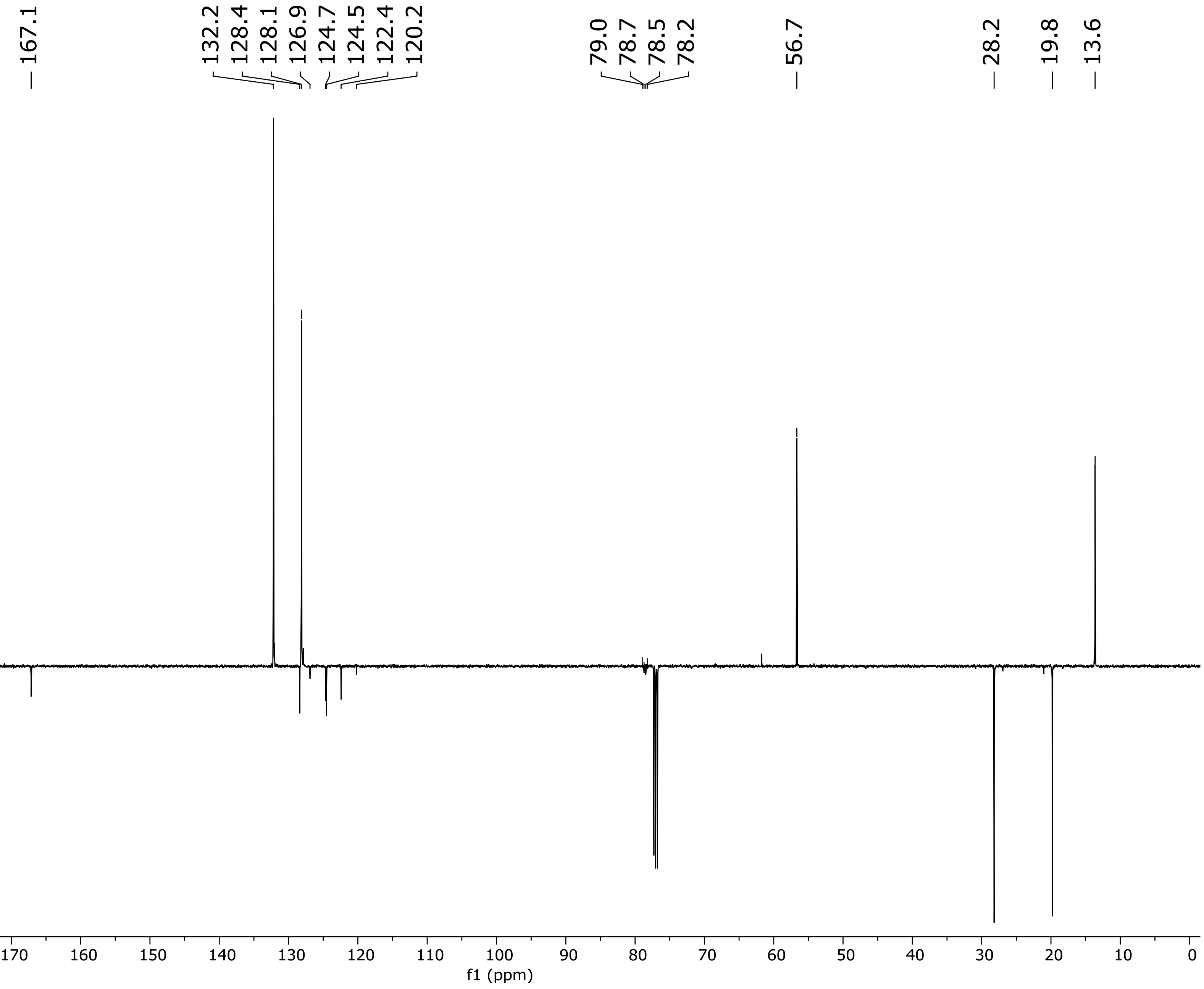

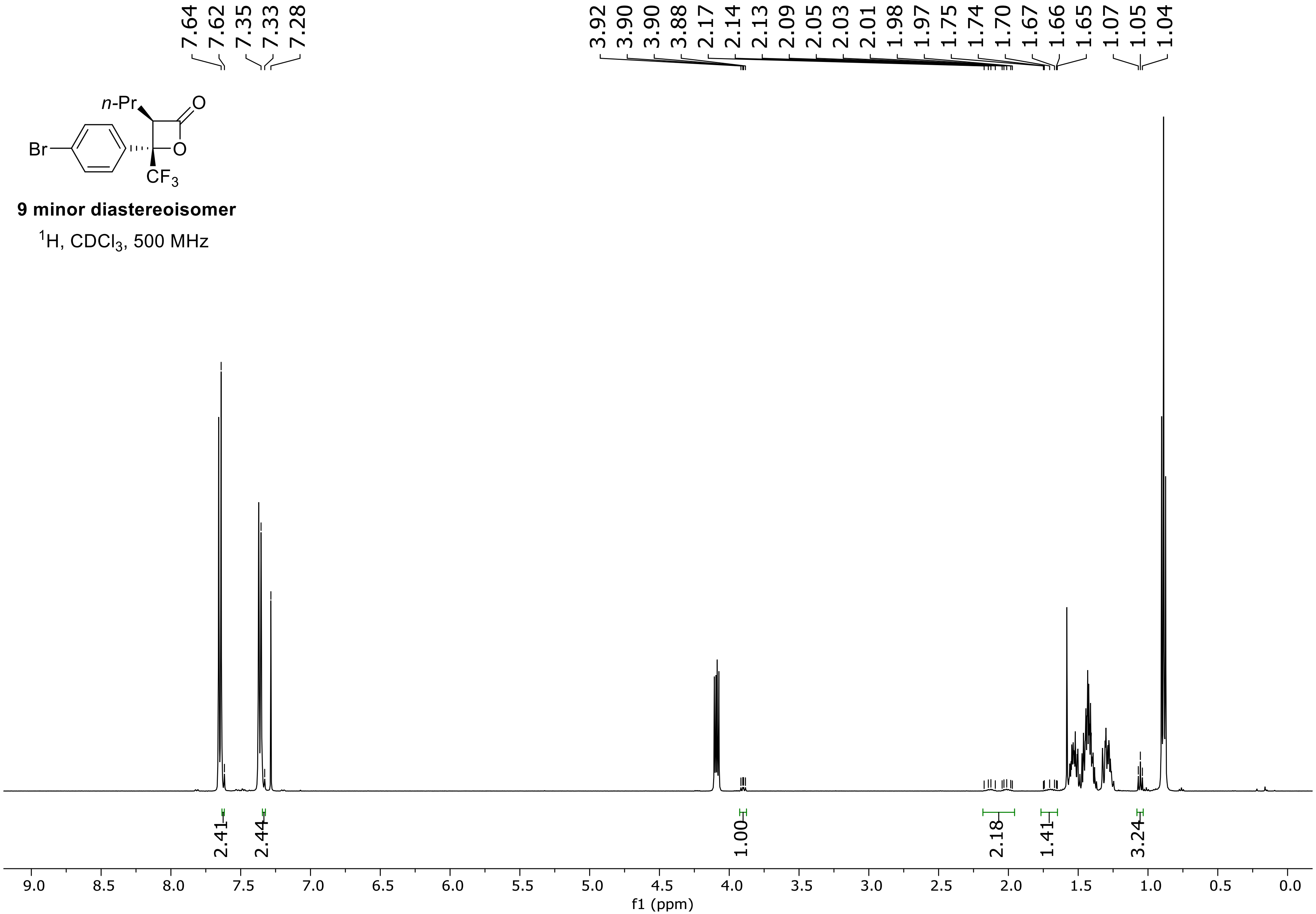
7.64
7.62
7.35
7.33
7.28
3.92
3.90
3.90
3.88
2.17
2.14
2.13
2.09
2.05
2.03
2.01
1.98
1.97
1.75
1.74
1.70
1.67
1.66
1.65
1.07
1.05
1.04
n-Pr
O
Br
O
CF3
**9 minor diastereoisomer**
1H, CDCl3, 500 MHz
2.41
2.44
1.00
2.18
1.41
3.24
9.0
8.5
8.0
7.5
7.0
6.5
6.0
5.5
5.0
4.5
4.0
3.5
3.0
2.5
2.0
1.5
1.0
0.5
0.0
f1 (ppm)

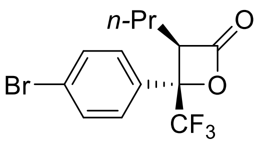

**9 minor diastereoisomer**

$^{19}F$, $CDCl_3$, 471 MHz

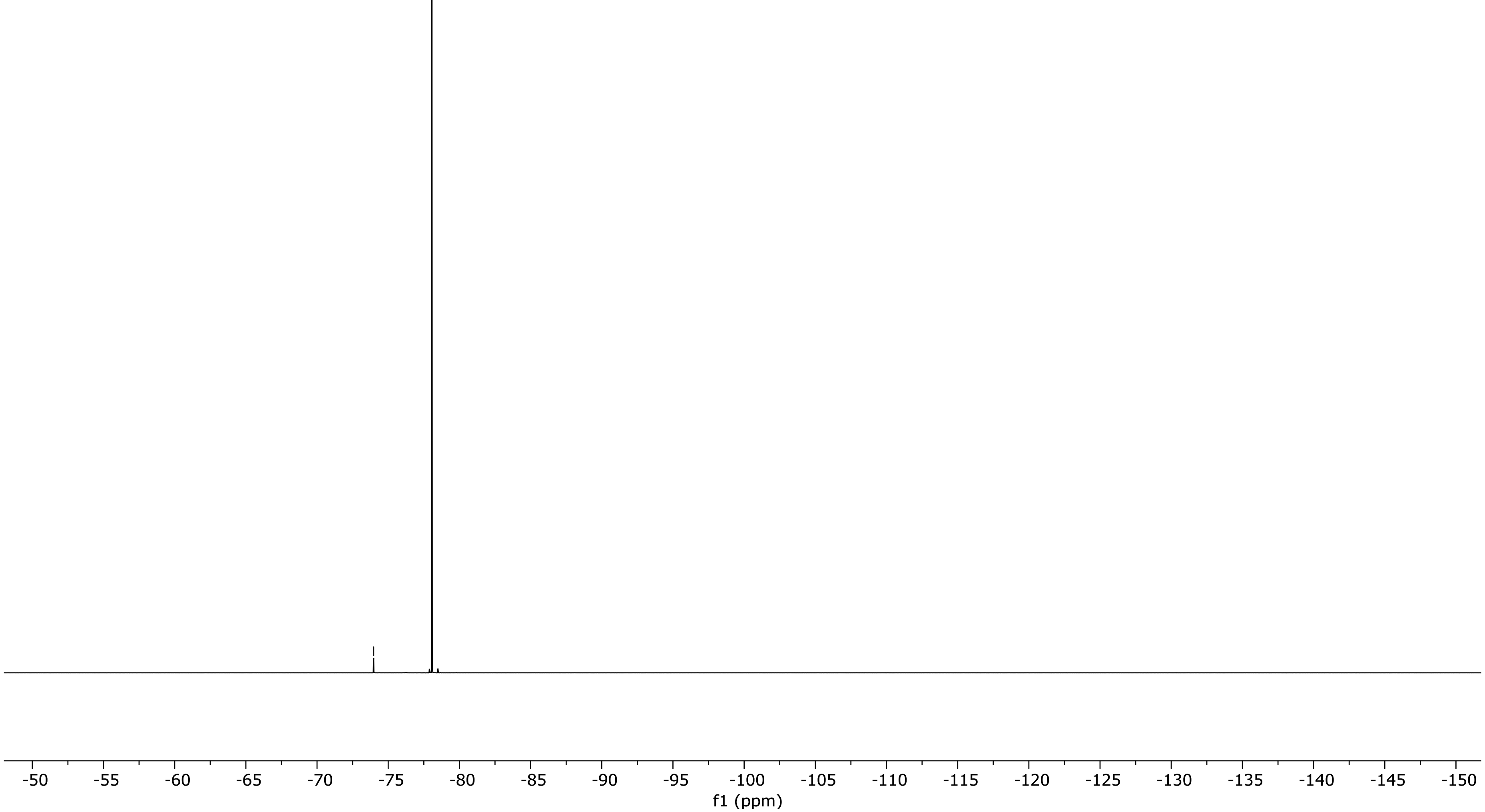

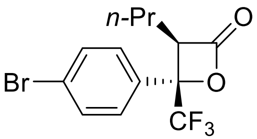


**9 minor diastereoisomer**

$^{13}C$, $CDCl_3$, 126 MHz

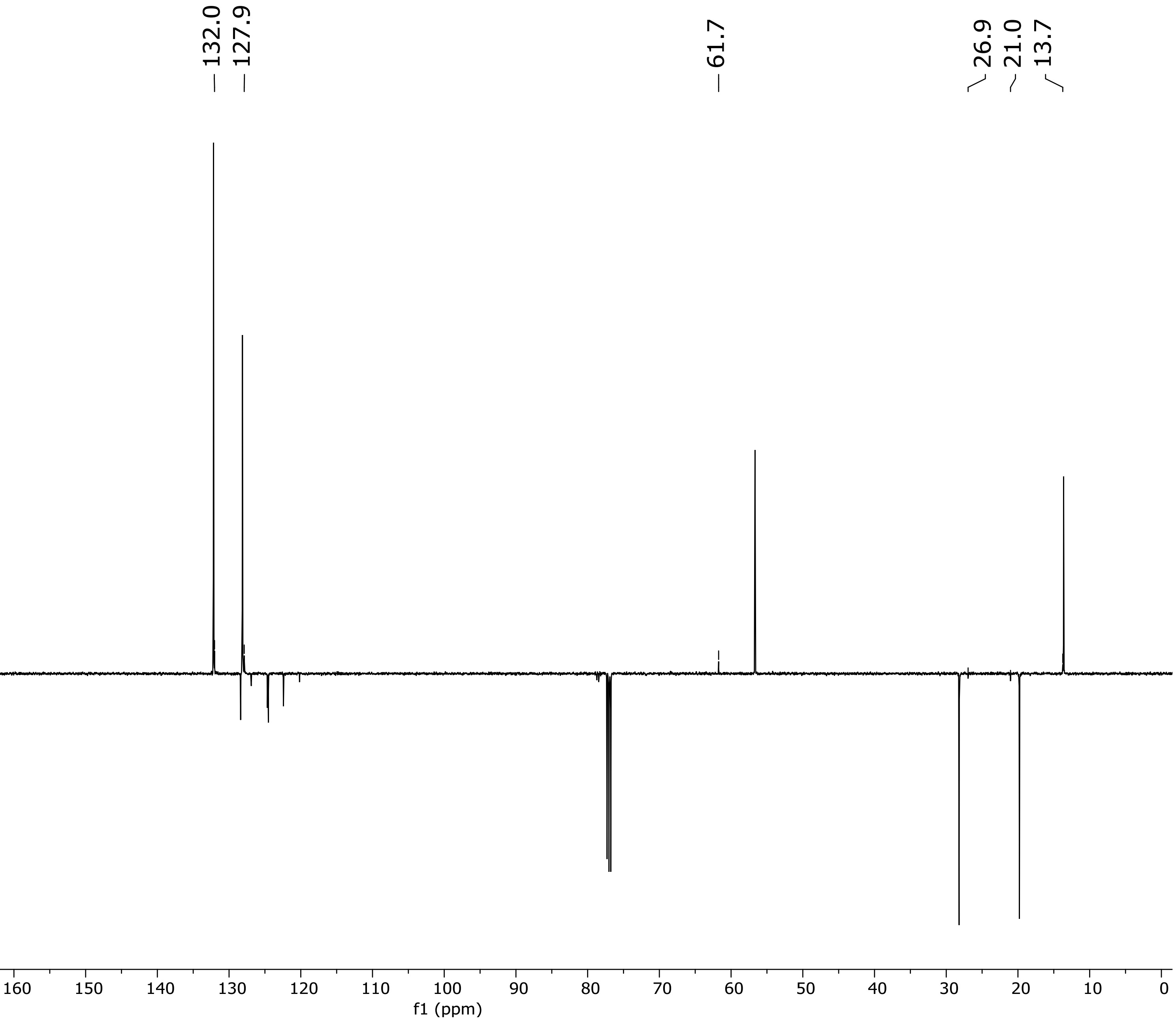

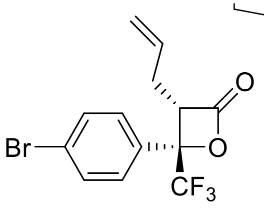


**10**

$^{1}H$, $CDCl_3$, 400 MHz

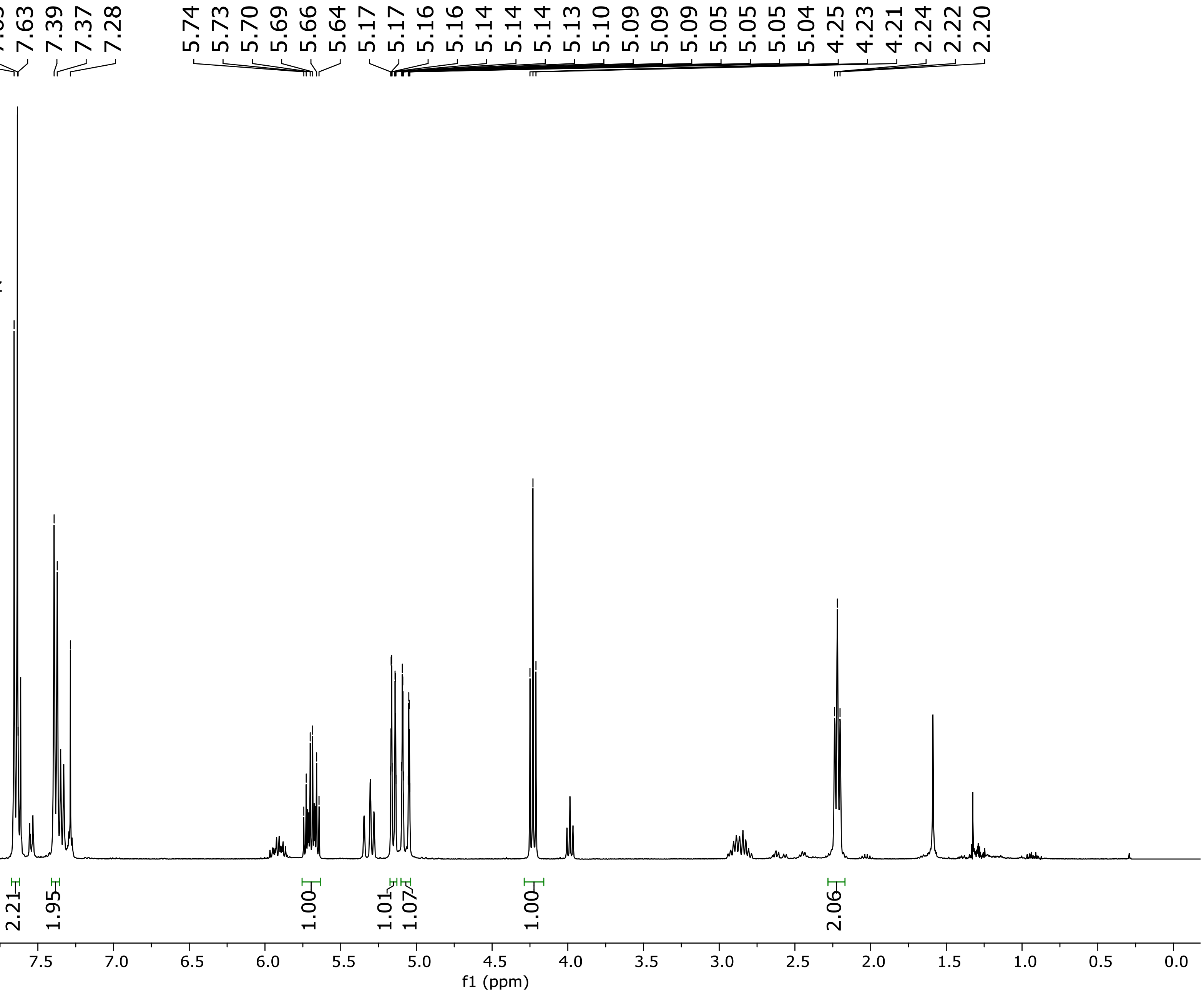

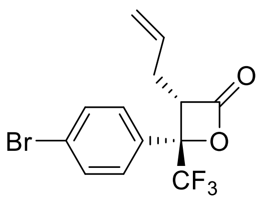

**10**

$^{19}$F, $CDCl_3$, 376 MHz

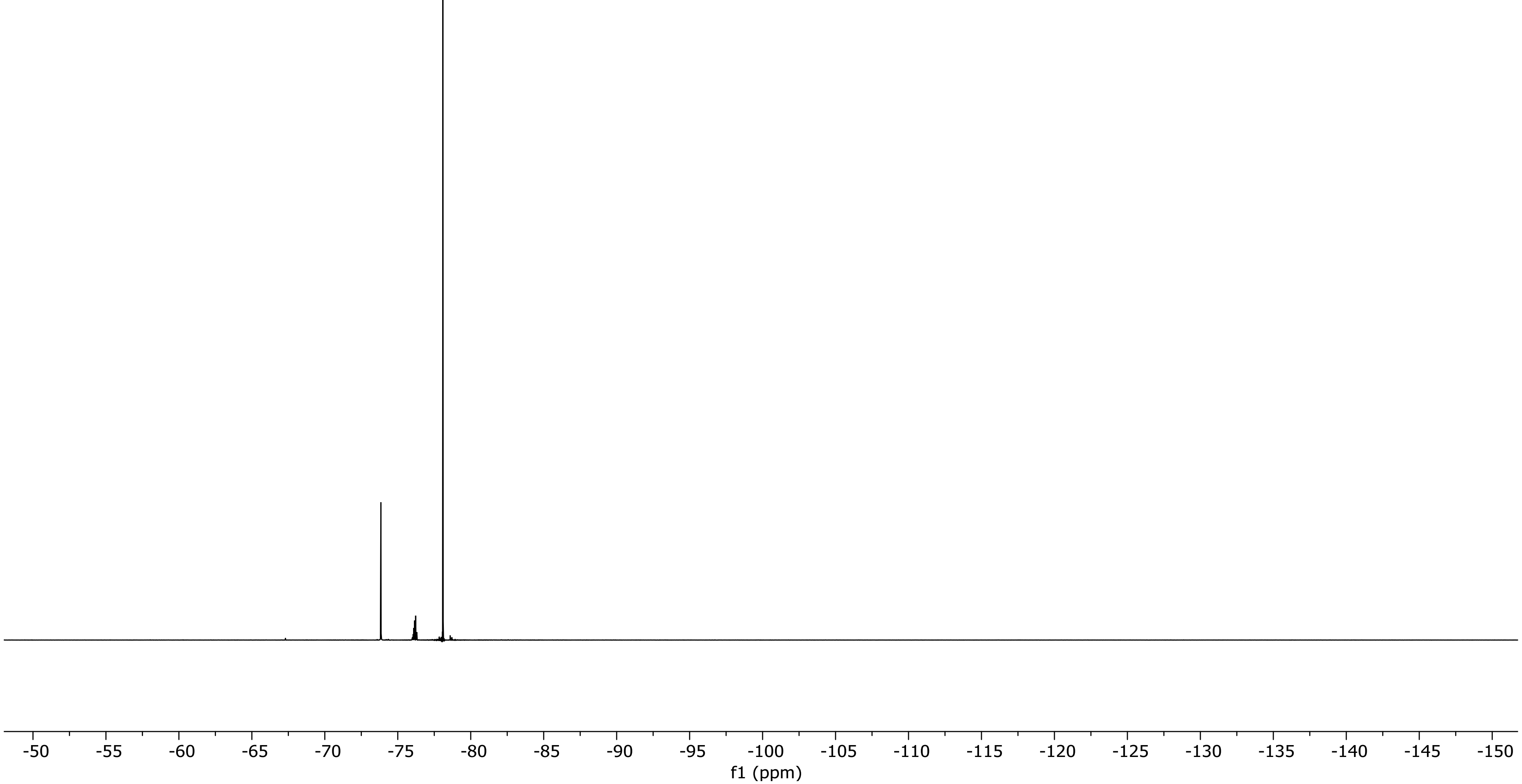

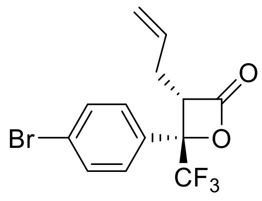

**10**

$^{13}C$, $CDCl_3$, 126 MHz

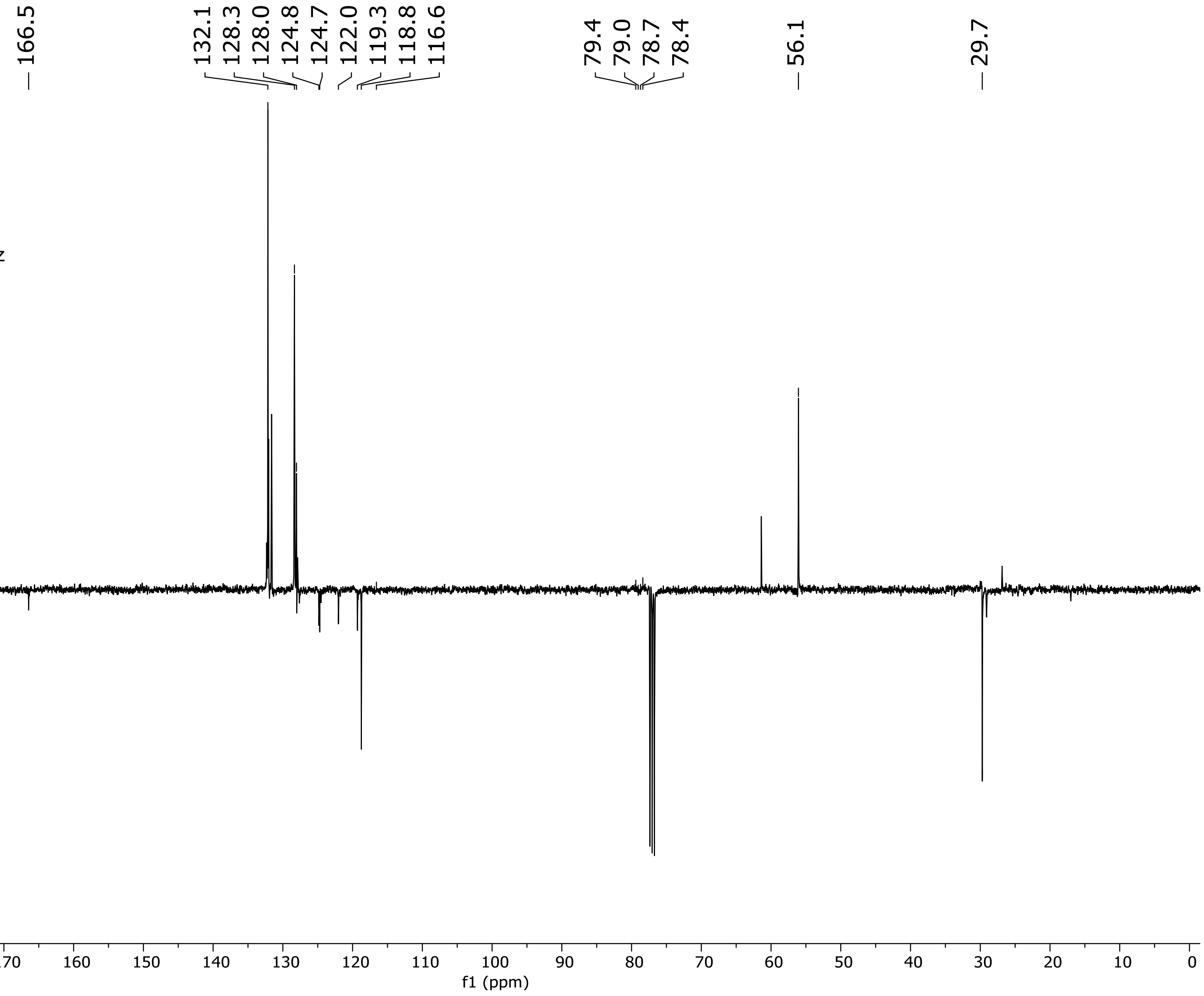

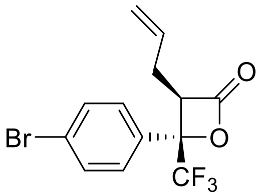

**10 minor diastereoisomer**

$^{1}$H, $CDCl_3$, 400 MHz

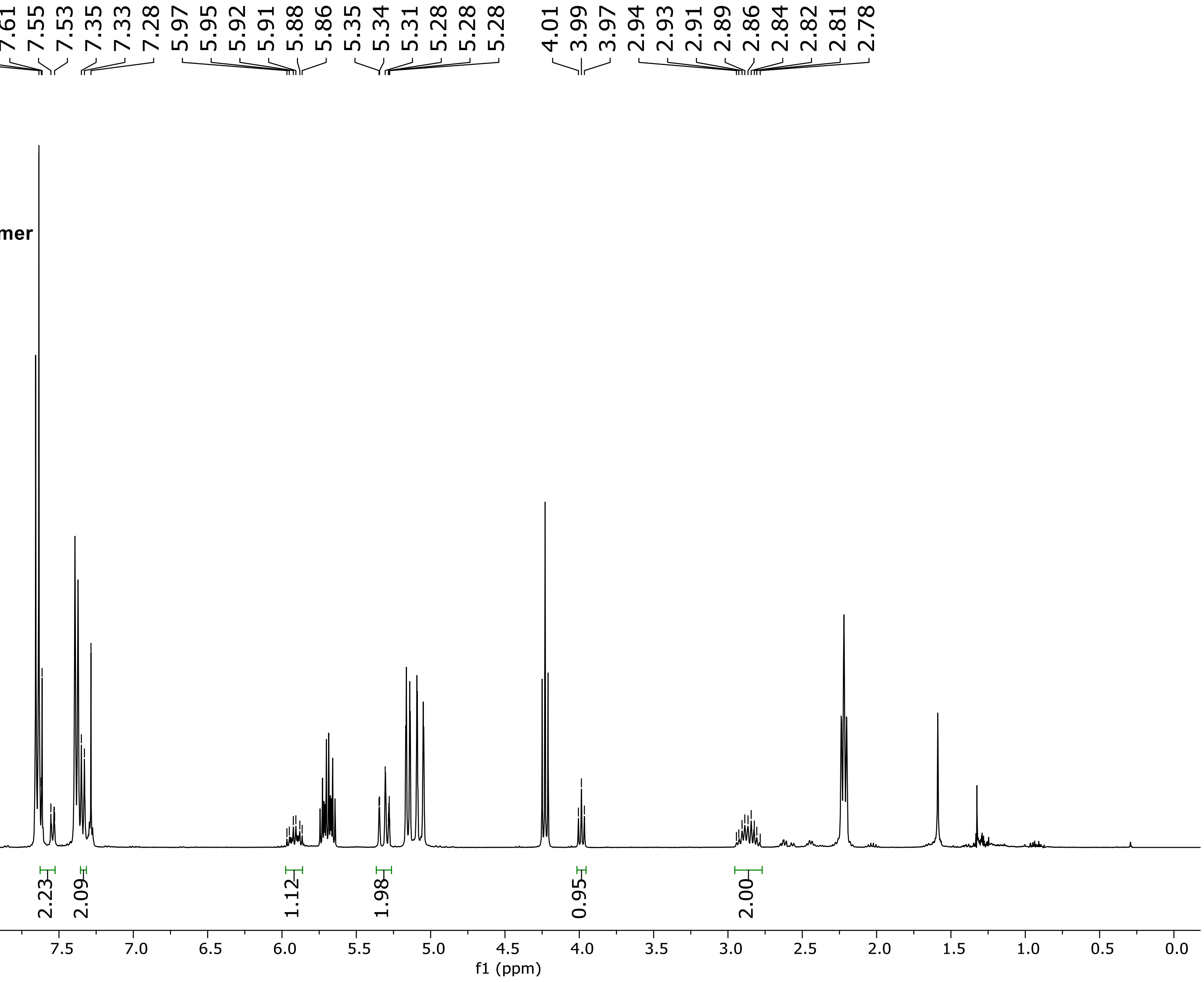

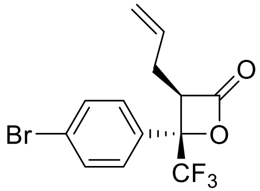

**10 minor diastereoisomer**

$^{19}F$, $CDCl_3$, 376 MHz

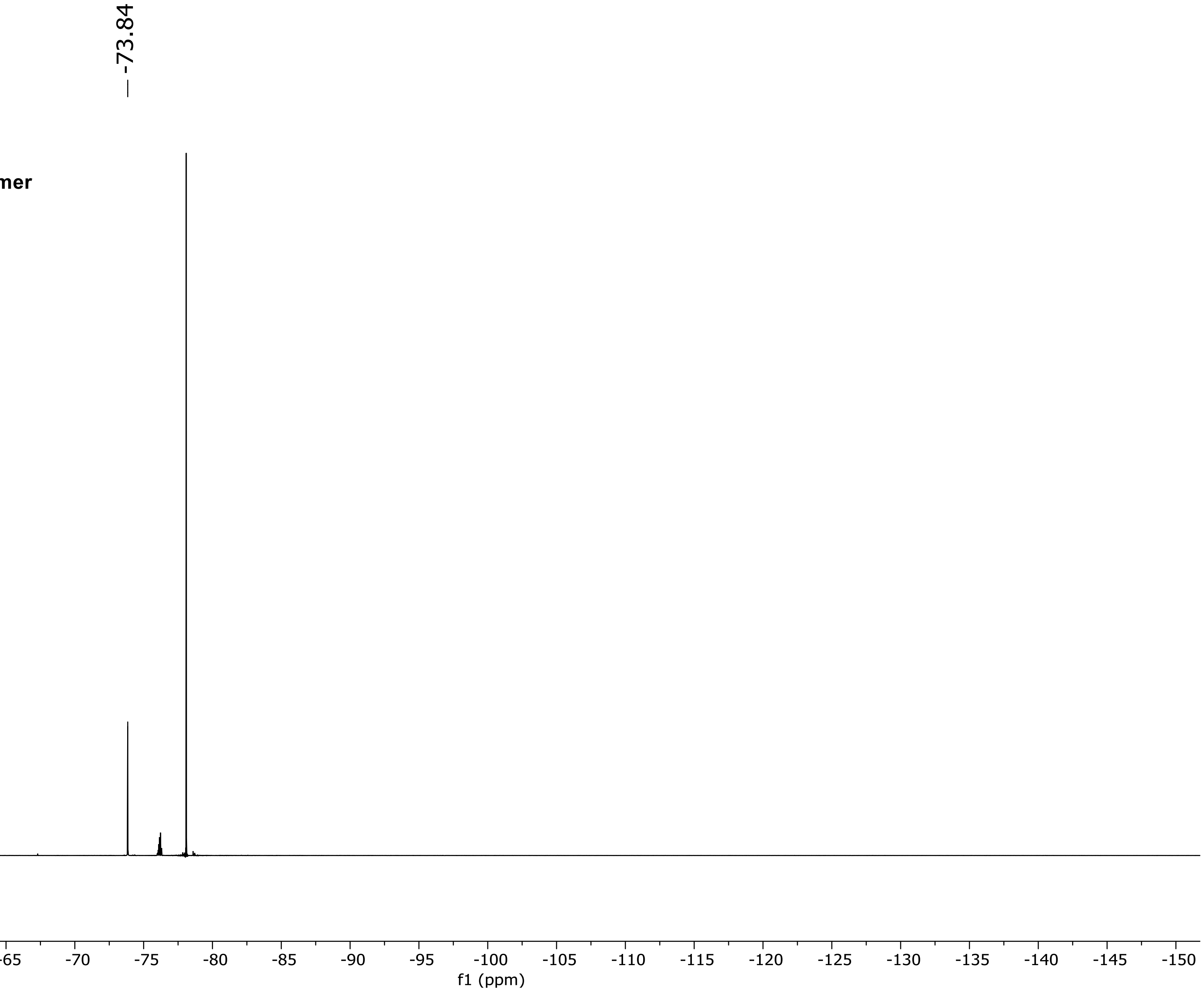

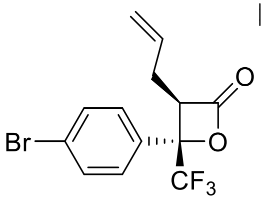

**10 minor diastereoisomer**

$^{13}C$, $CDCl_3$, 126 MHz

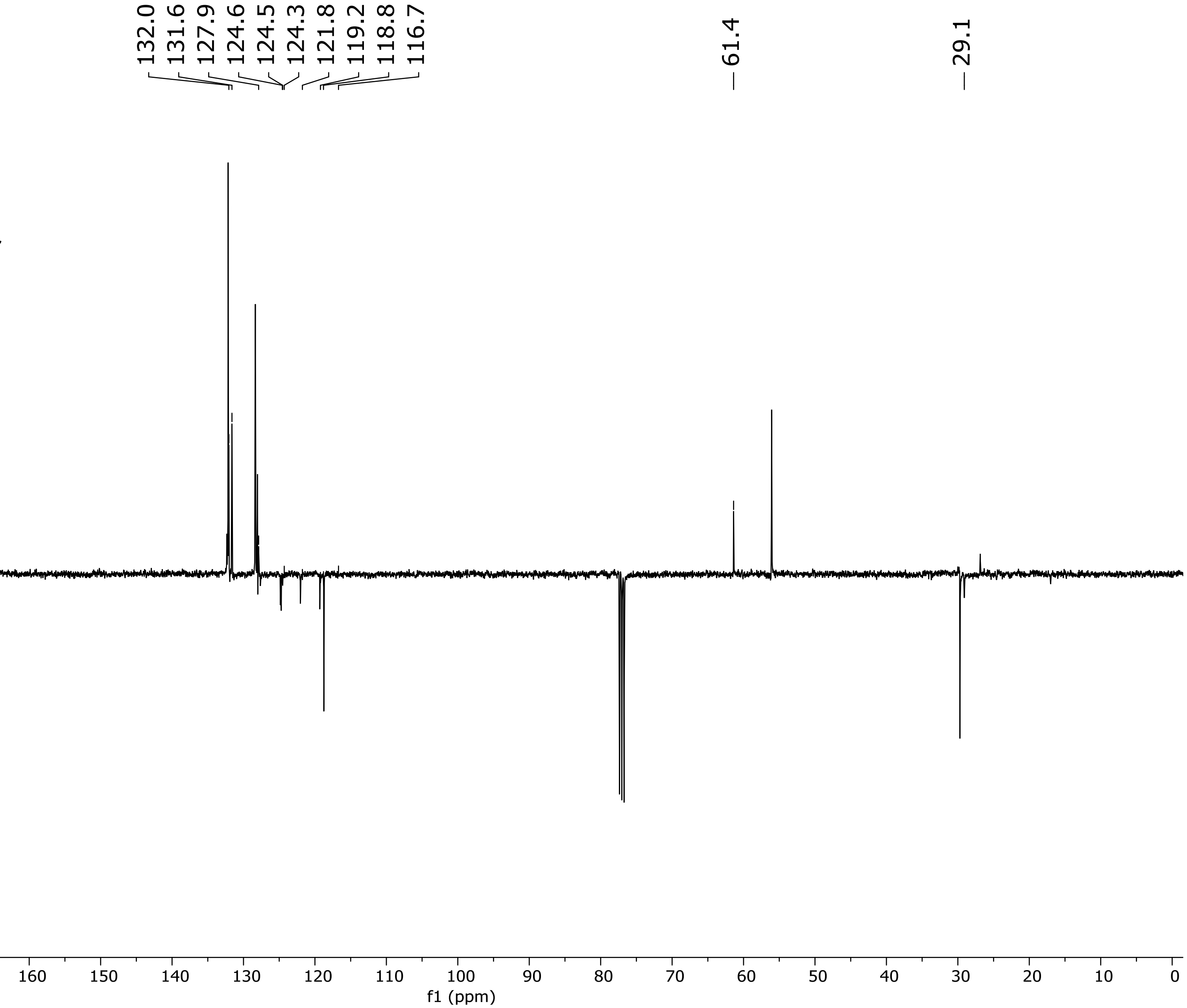

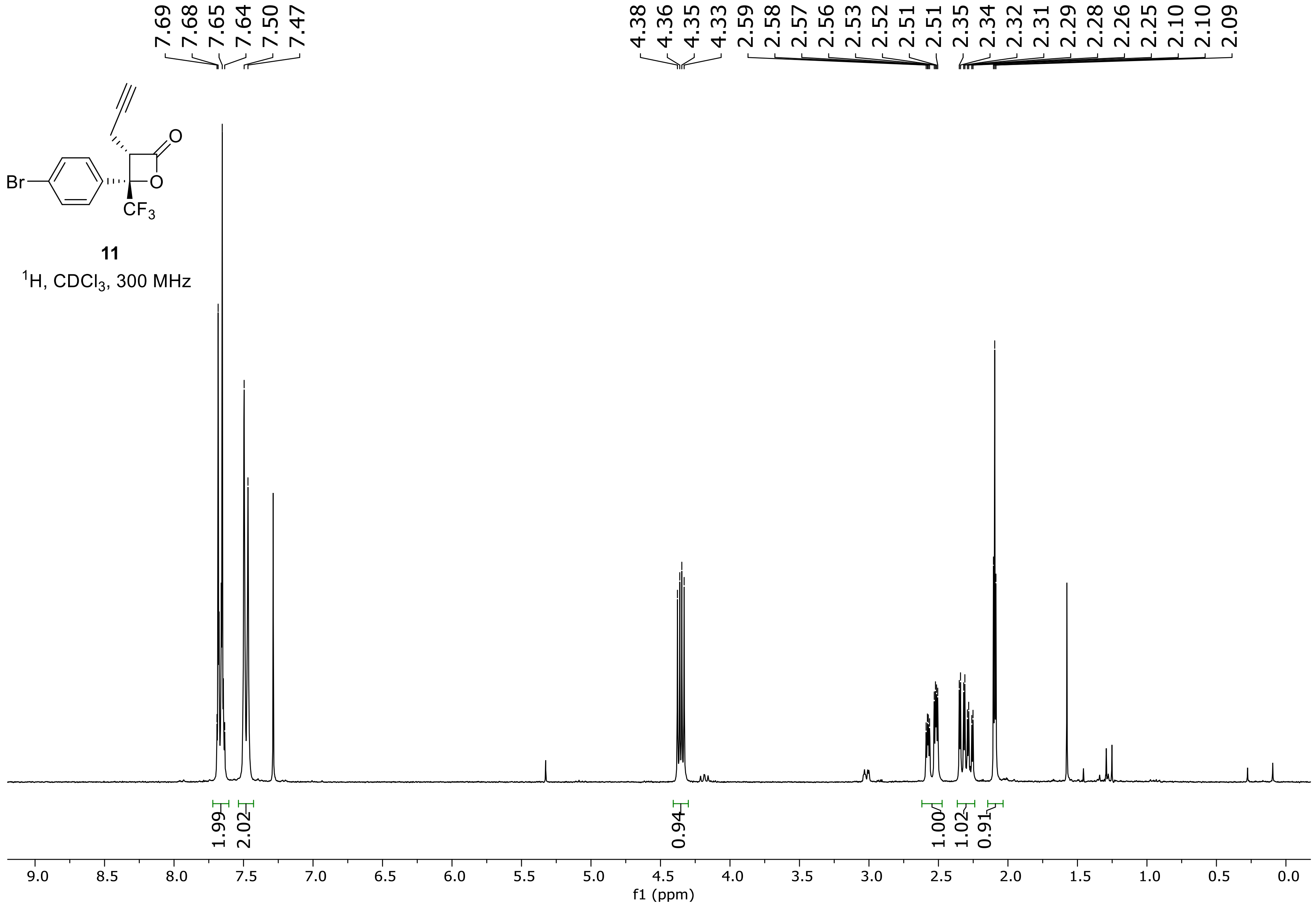
7.69
7.68
7.65
7.64
7.50
7.47
4.38
4.36
4.35
4.33
2.59
2.58
2.57
2.56
2.53
2.52
2.51
2.51
2.35
2.34
2.32
2.31
2.29
2.28
2.26
2.25
2.10
2.10
2.09
Br
O
O
CF3
11
1H, CDCl3, 300 MHz
1.99
2.02
0.94
1.00
1.02
0.91
9.0 8.5 8.0 7.5 7.0 6.5 6.0 5.5 5.0 4.5 4.0 3.5 3.0 2.5 2.0 1.5 1.0 0.5 0.0
f1 (ppm)

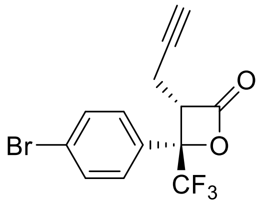

**11**

$^{19}$F, $CDCl_3$, 282 MHz

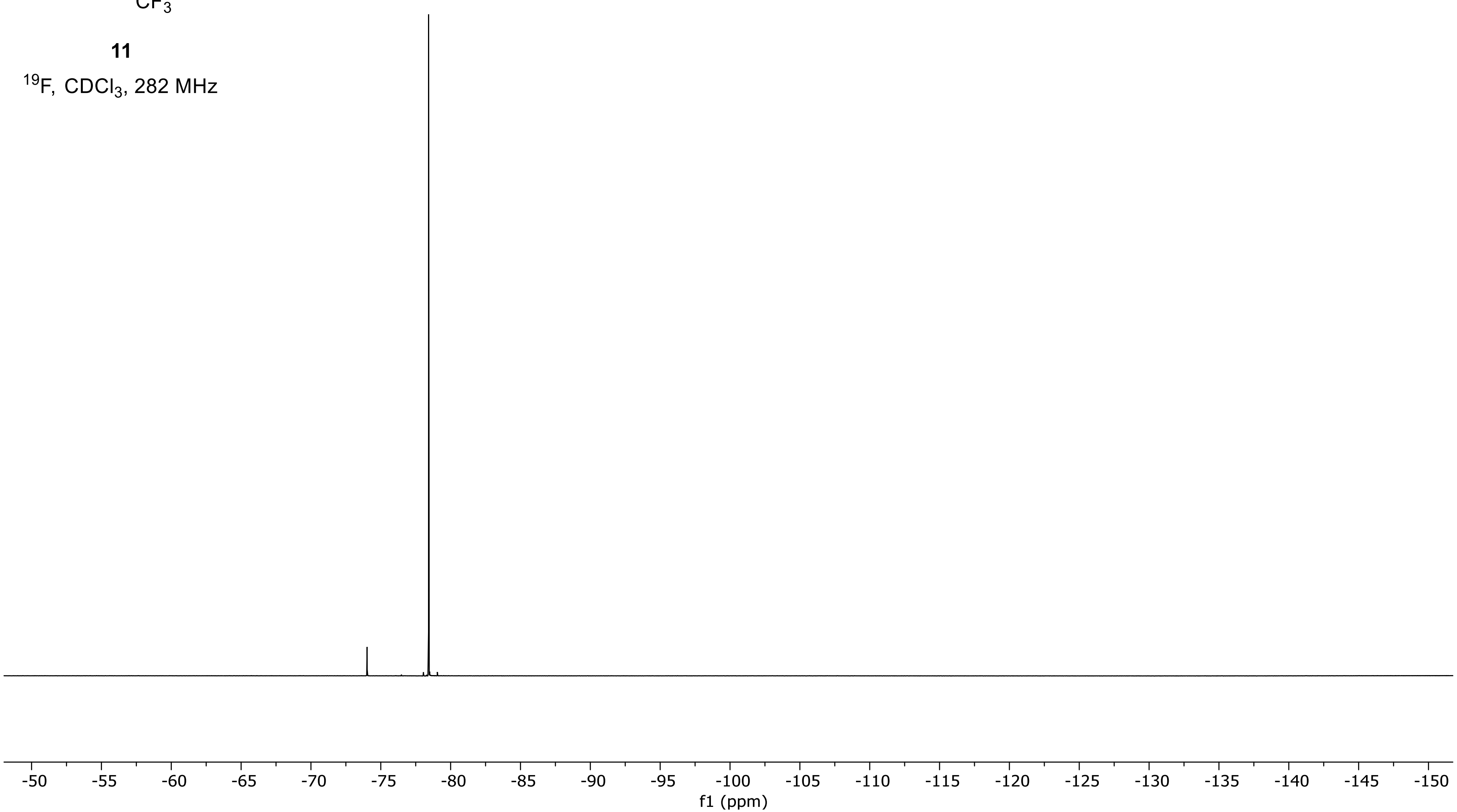

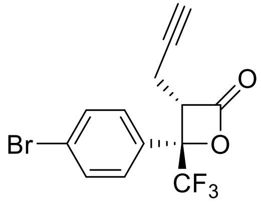

**11**

$^{13}C$, $CDCl_3$, 126 MHz

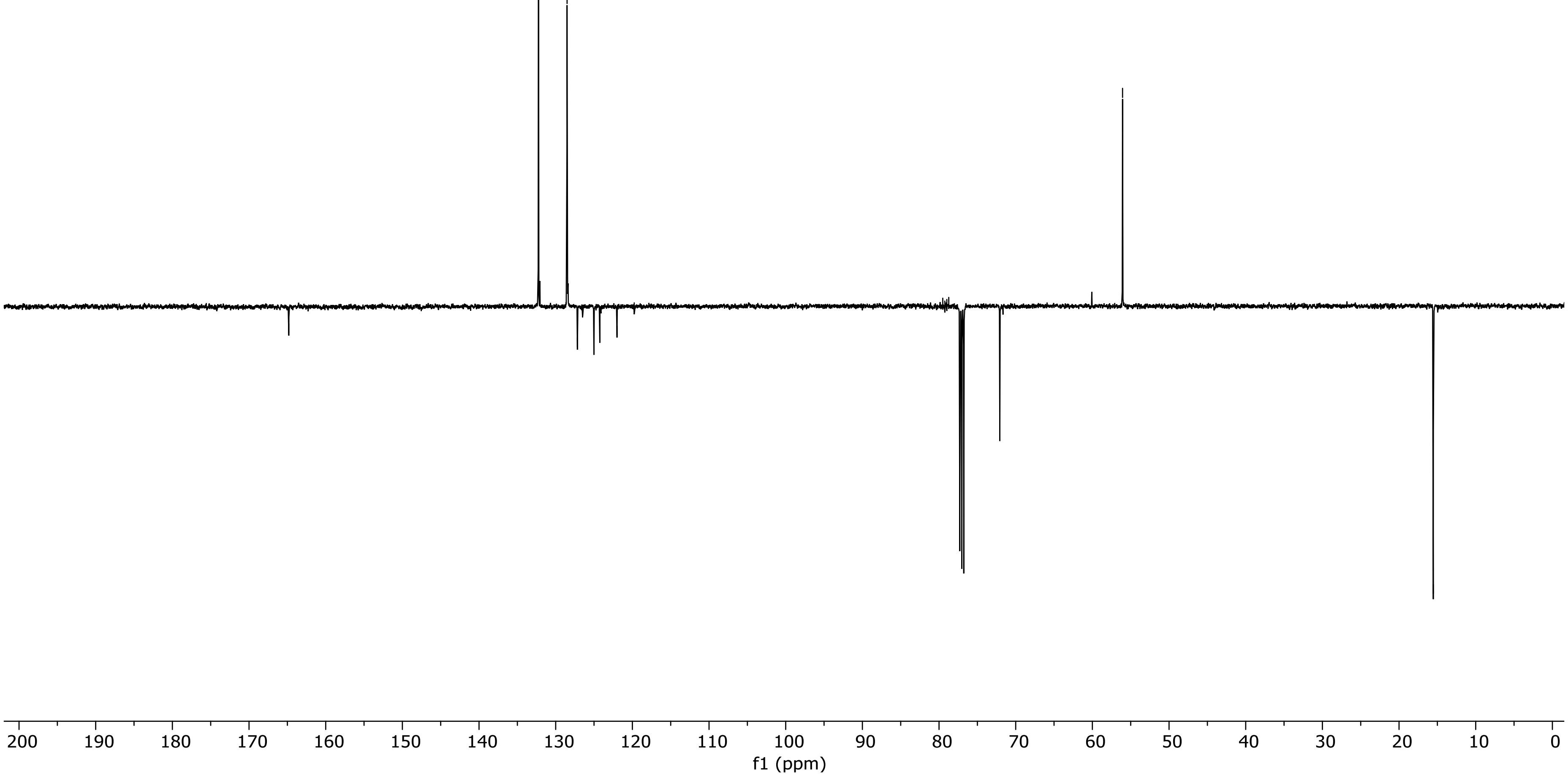

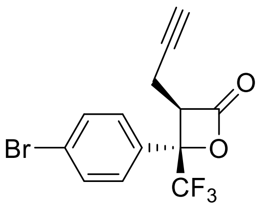

**11 minor diastereoisomer**

$^1H$, $CDCl_3$, 300 MHz

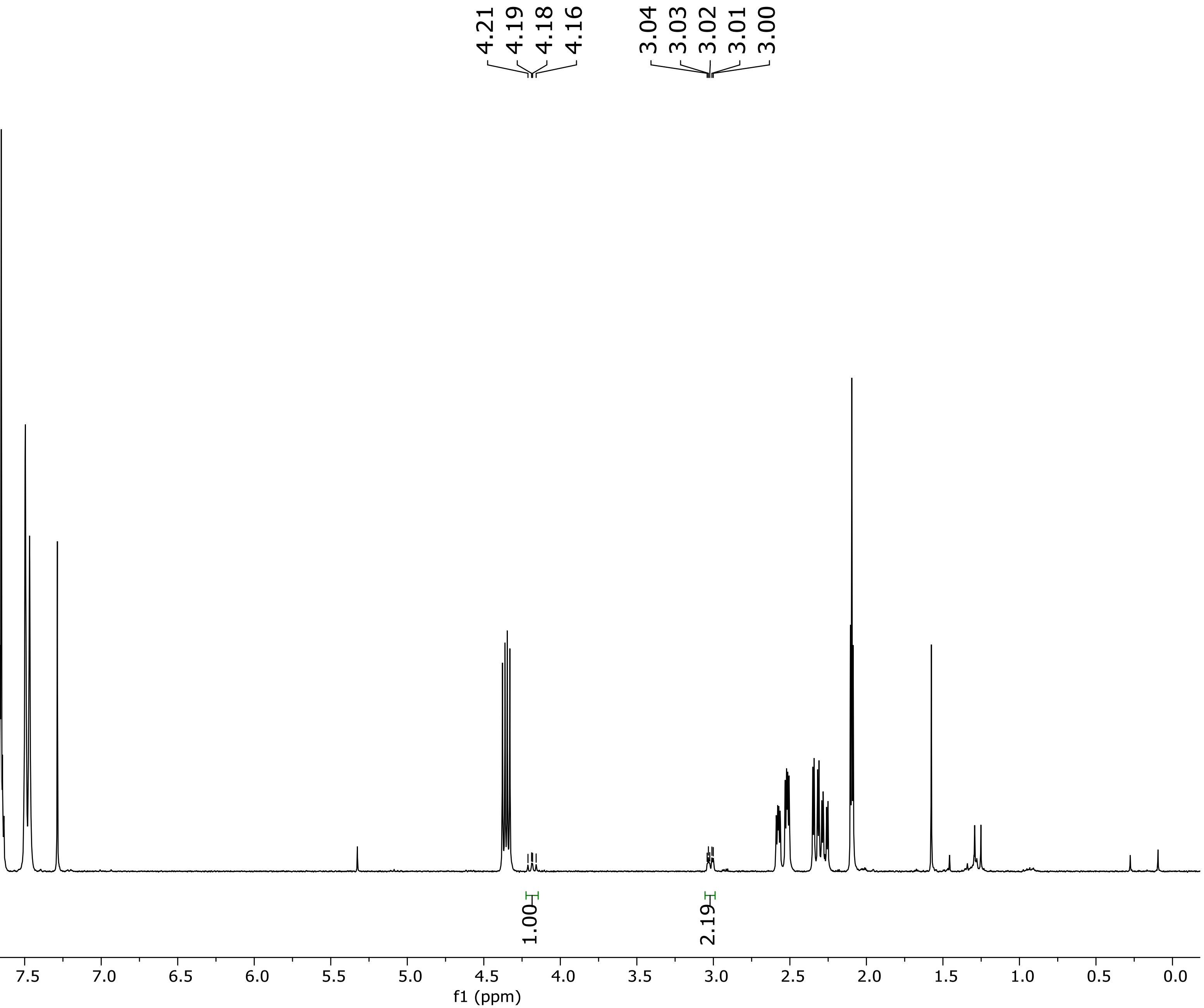

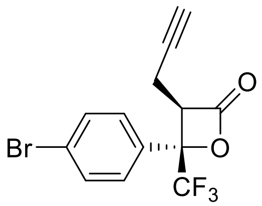

**11 minor diastereoisomer**

$^{19}F$, $CDCl_3$, 282 MHz

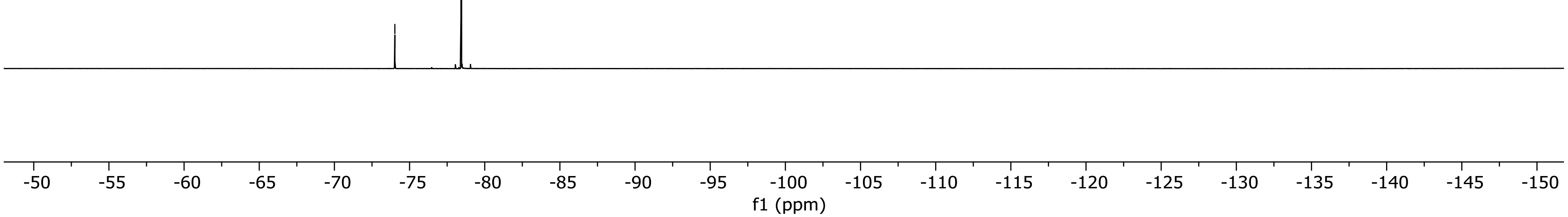

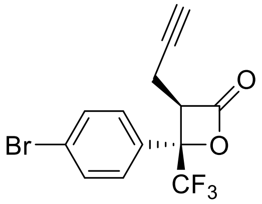


**11 minor diastereoisomer**

$^{13}C$, $CDCl_3$, 126 MHz

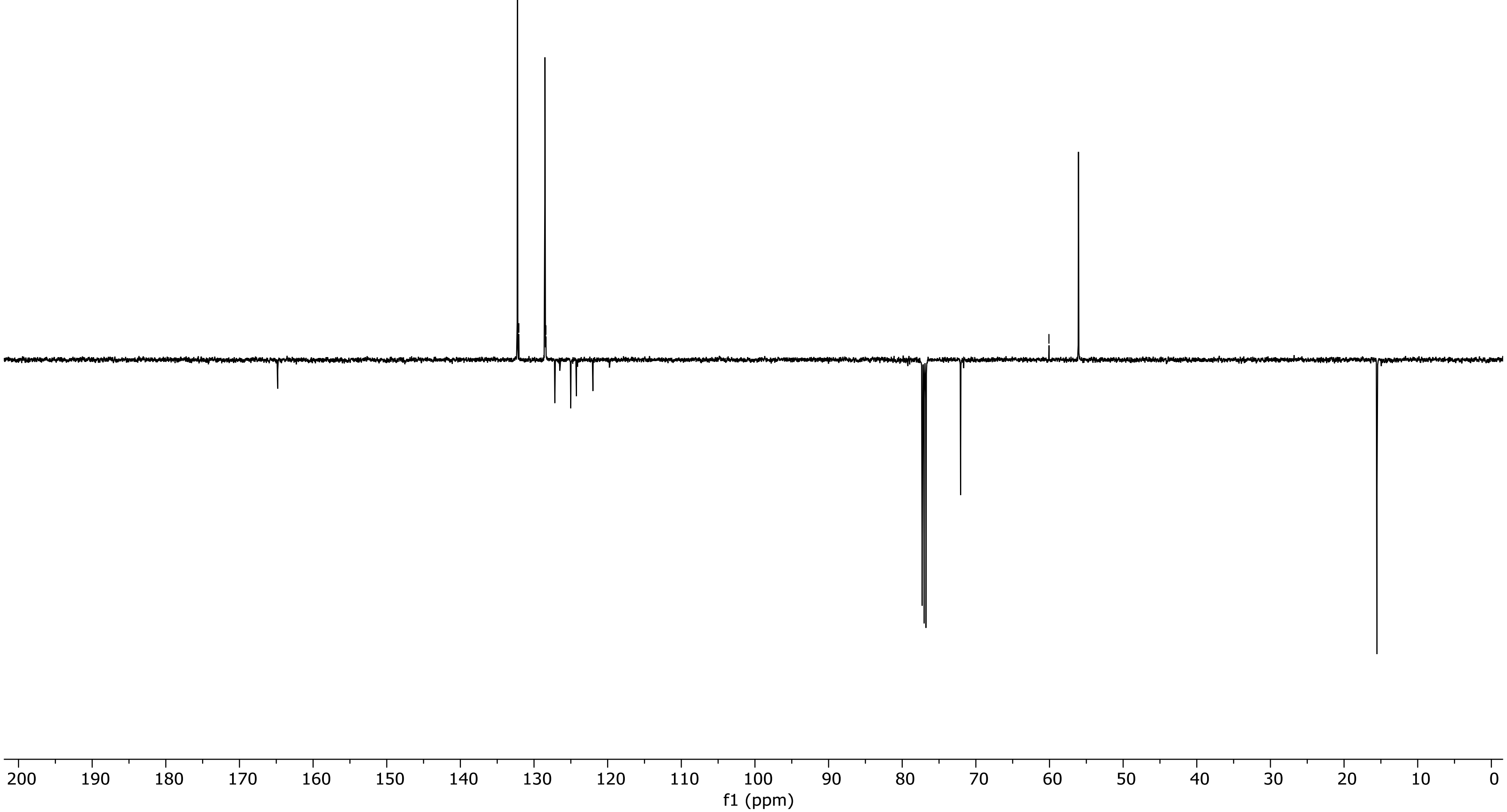

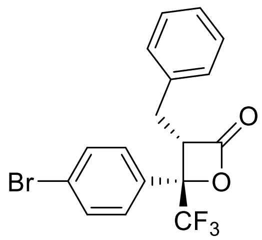

**12**

$^1H$, $CDCl_3$, 400 MHz

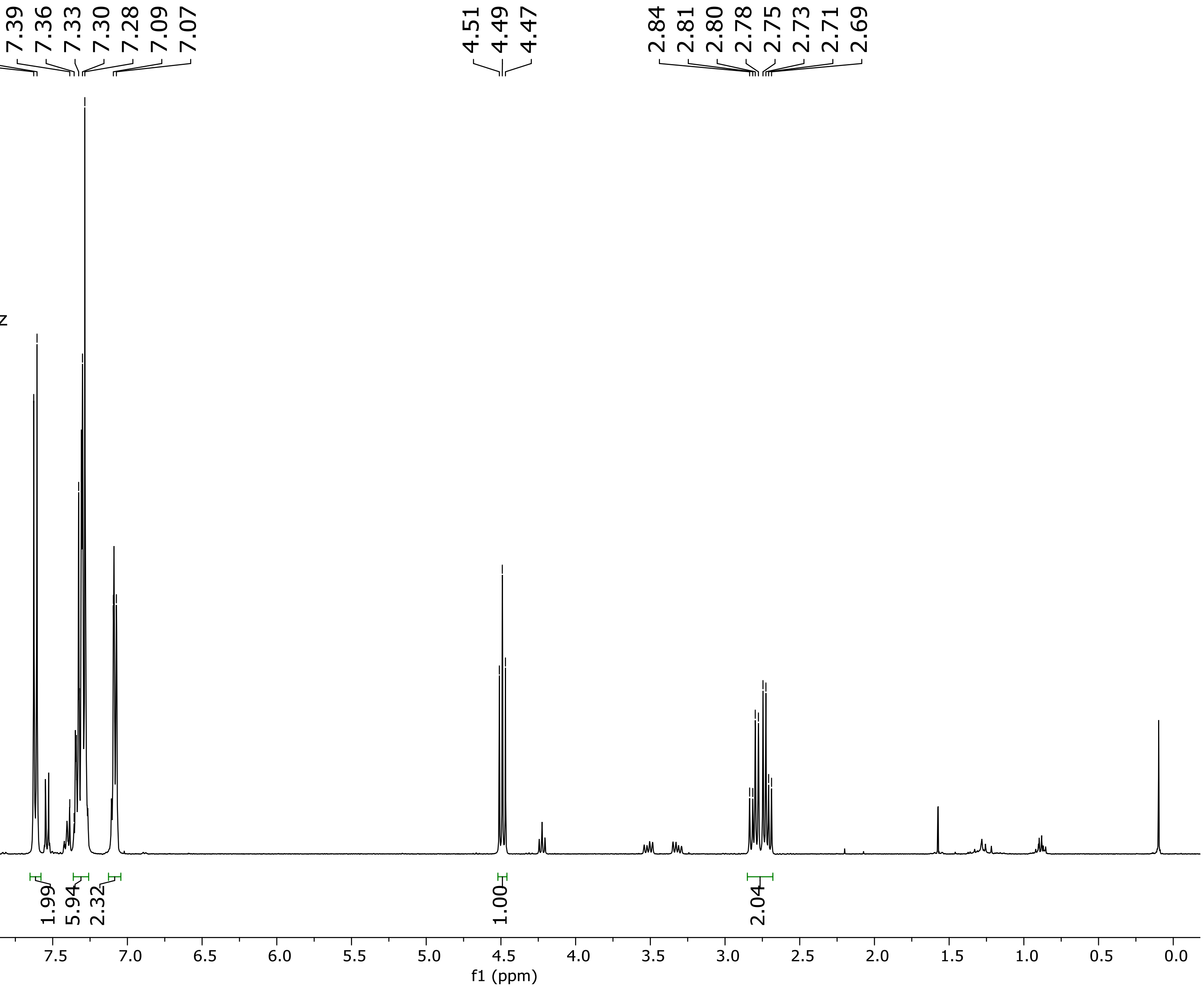

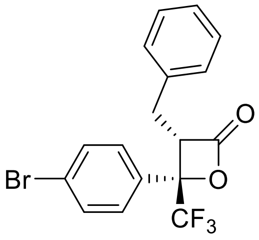

**12**

$^{19}F$, $CDCl_3$, 377 MHz

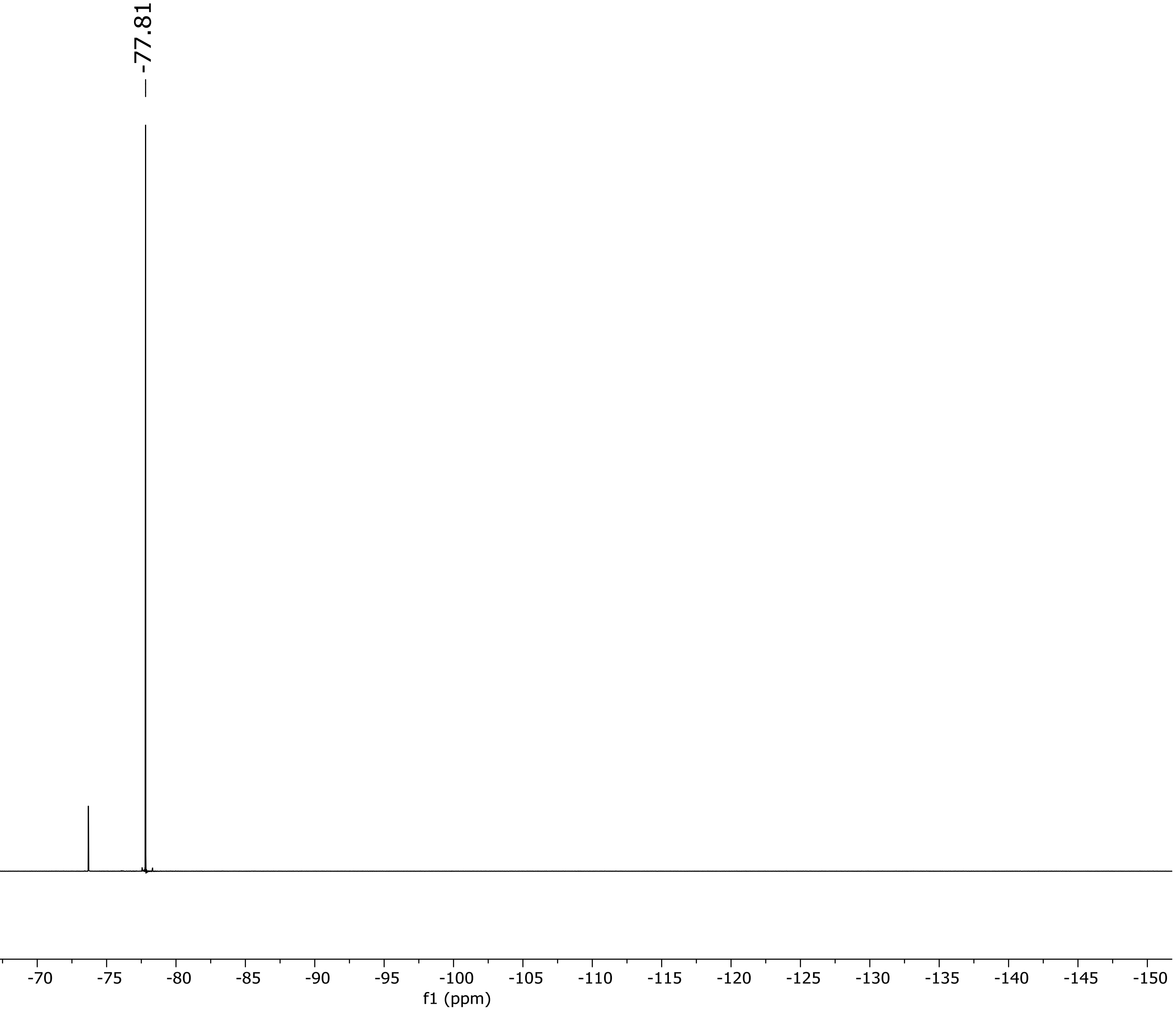

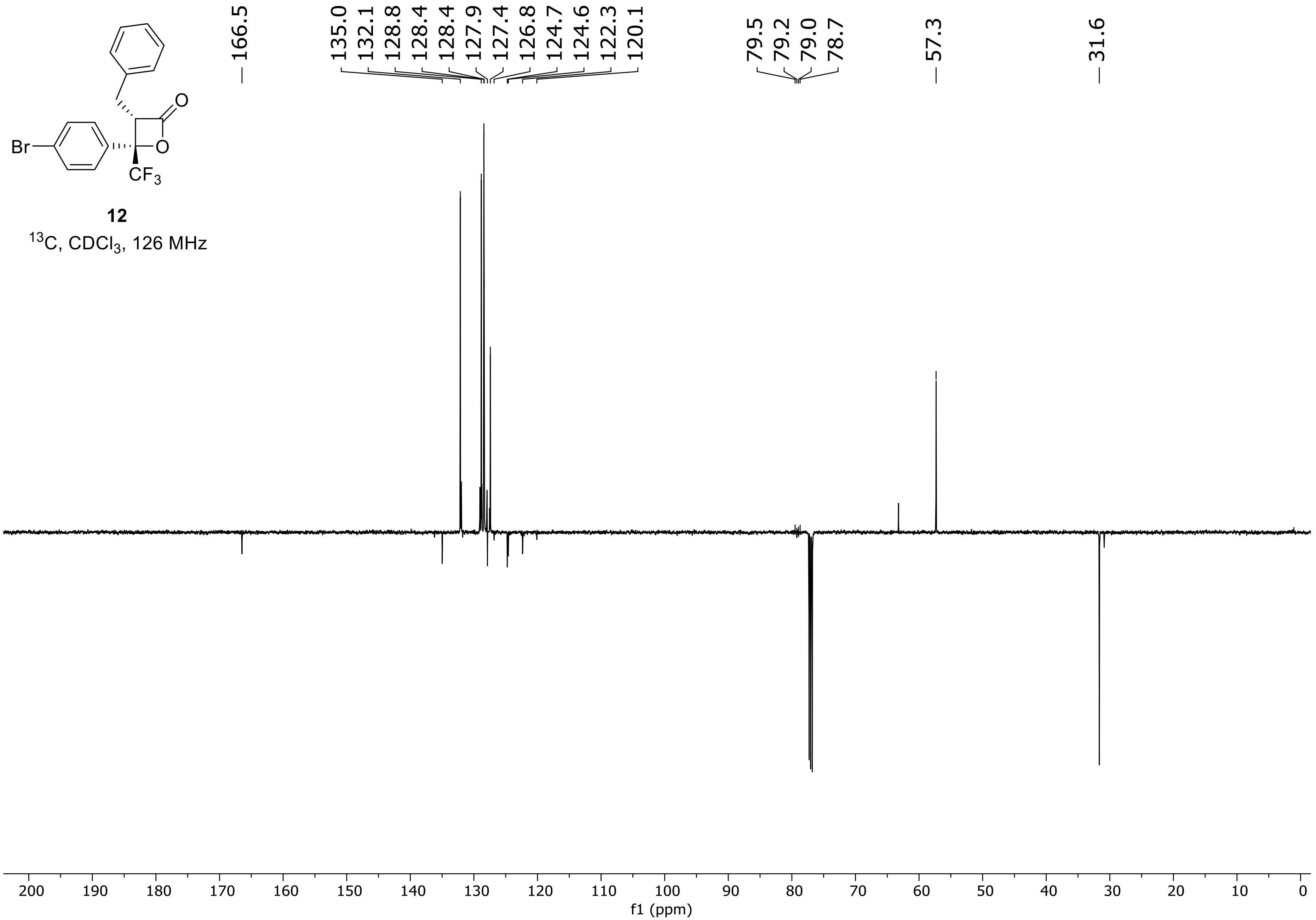
166.5
135.0
132.1
128.8
128.4
128.4
127.9
127.4
126.8
124.7
124.6
122.3
120.1
79.5
79.2
79.0
78.7
57.3
31.6
Br
O
O
$CF_3$
**12**
$^{13}C$, $CDCl_3$, 126 MHz
200
190
180
170
160
150
140
130
120
110
100
90
80
70
60
50
40
30
20
10
0
f1 (ppm)

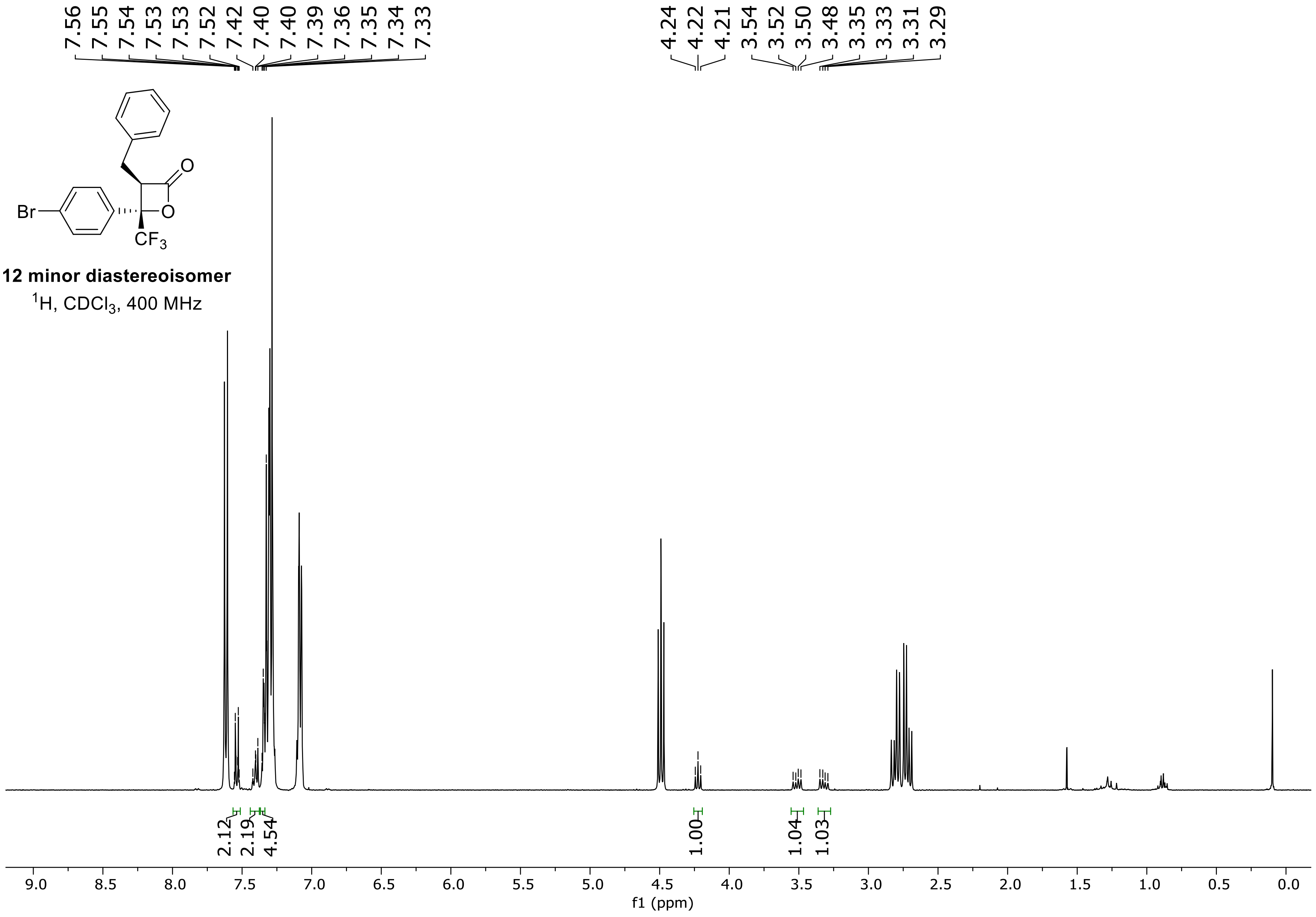
7.56
7.55
7.54
7.53
7.53
7.52
7.42
7.40
7.40
7.39
7.36
7.35
7.34
7.33
4.24
4.22
4.21
3.54
3.52
3.50
3.48
3.35
3.33
3.31
3.29
Br
O
O
CF3
12 minor diastereoisomer
1H, CDCl3, 400 MHz
2.12
2.19
4.54
1.00
1.04
1.03
9.0
8.5
8.0
7.5
7.0
6.5
6.0
5.5
5.0
4.5
4.0
3.5
3.0
2.5
2.0
1.5
1.0
0.5
0.0
f1 (ppm)

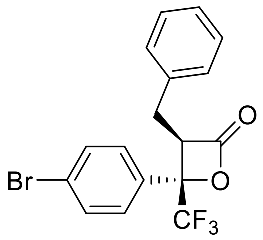


**12 minor diastereoisomer**

$^{19}F$, $CDCl_3$, 377 MHz

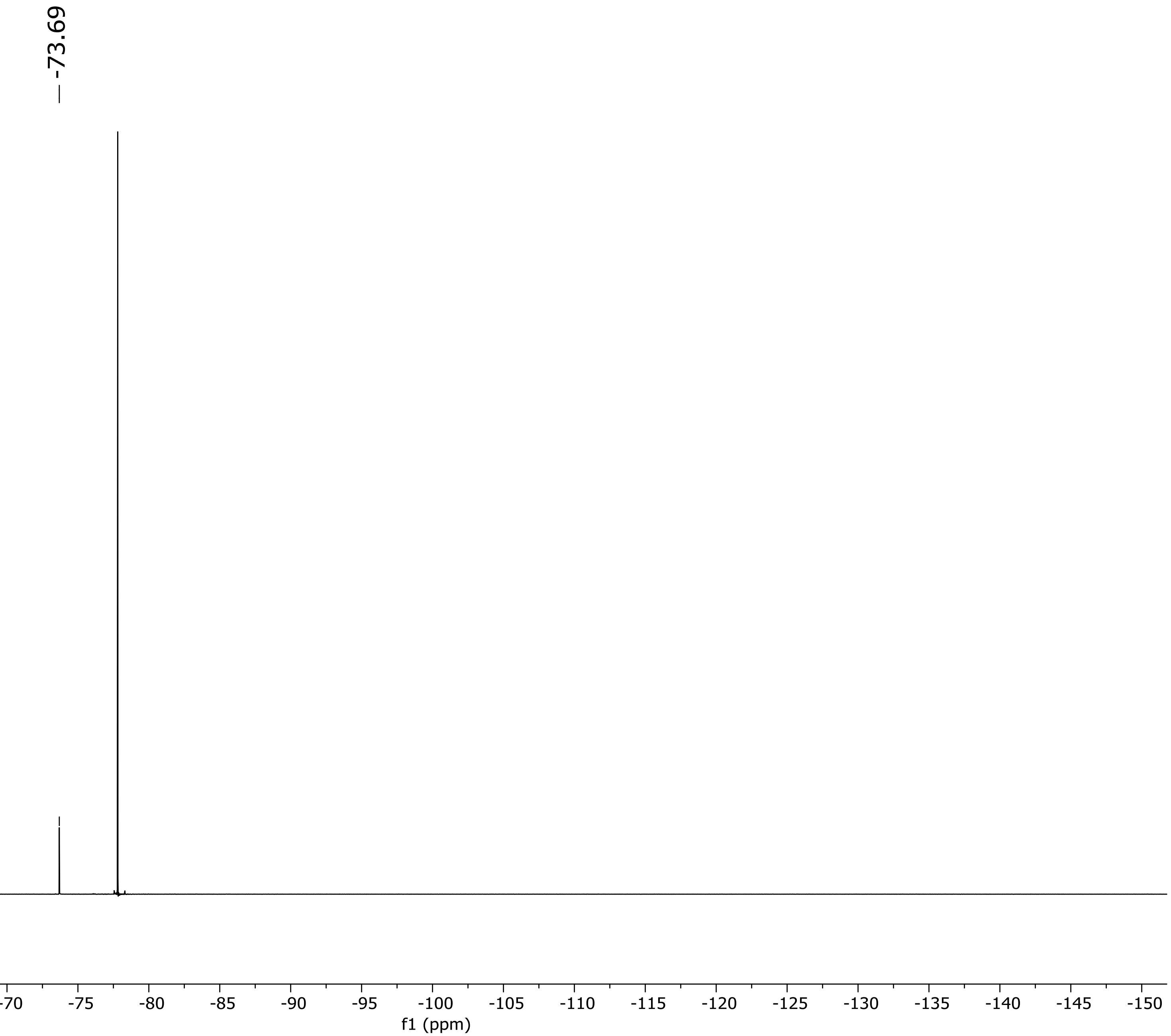

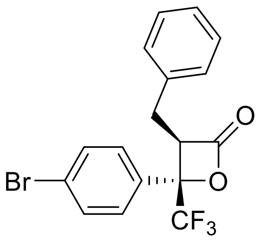


**12 minor diastereoisomer**

$^{13}C$, $CDCl_3$, 126 MHz

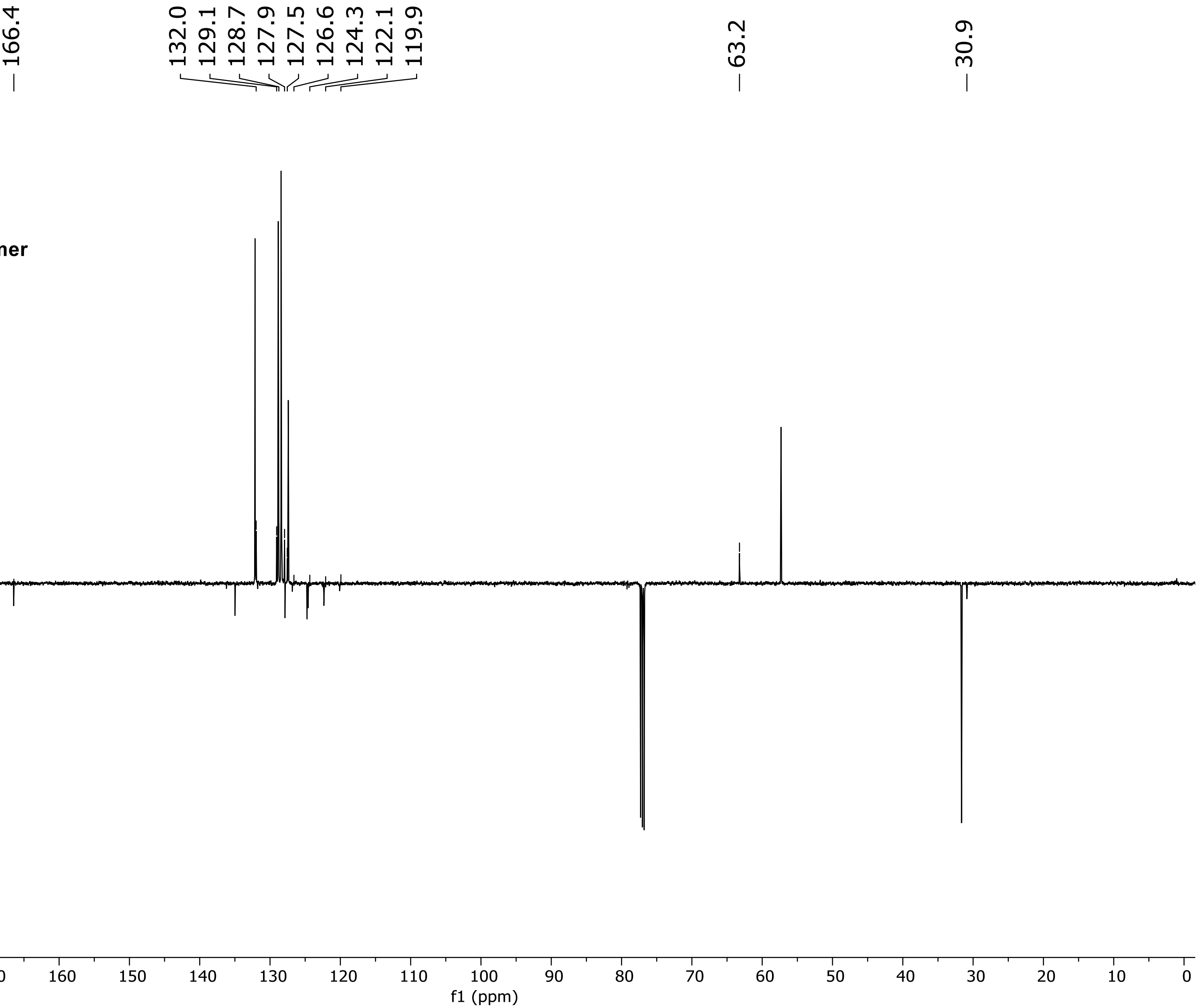

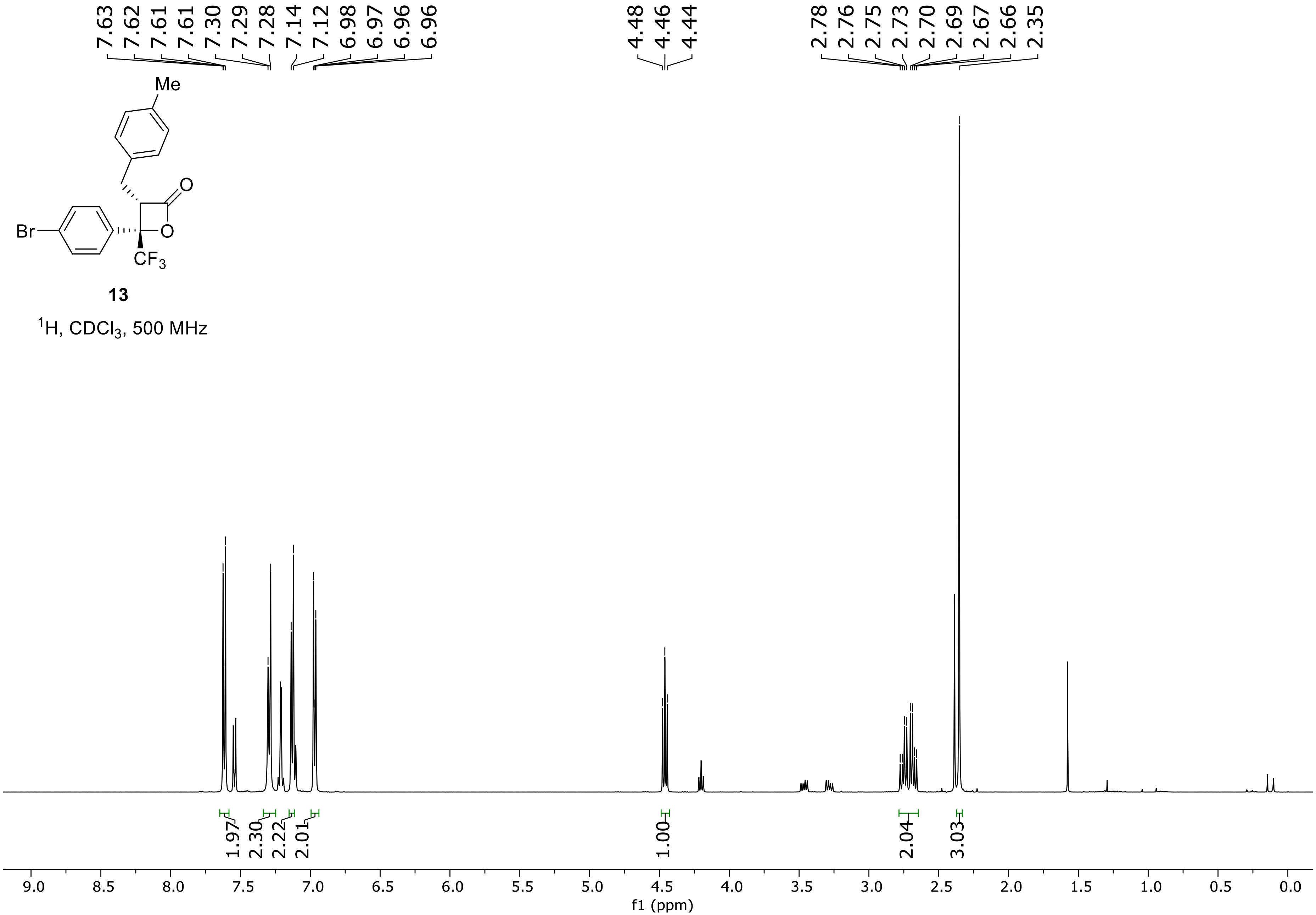
7.63
7.62
7.61
7.61
7.30
7.29
7.28
7.14
7.12
6.98
6.97
6.96
6.96
4.48
4.46
4.44
2.78
2.76
2.75
2.73
2.70
2.69
2.67
2.66
2.35
Me
O
Br
O
CF3
13
1H, CDCl3, 500 MHz
1.97
2.30
2.22
2.01
1.00
2.04
3.03
9.0
8.5
8.0
7.5
7.0
6.5
6.0
5.5
5.0
4.5
4.0
3.5
3.0
2.5
2.0
1.5
1.0
0.5
0.0
f1 (ppm)

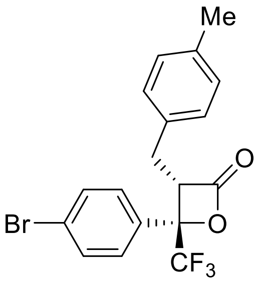


**13**

$^{19}F$, $CDCl_3$, 471 MHz

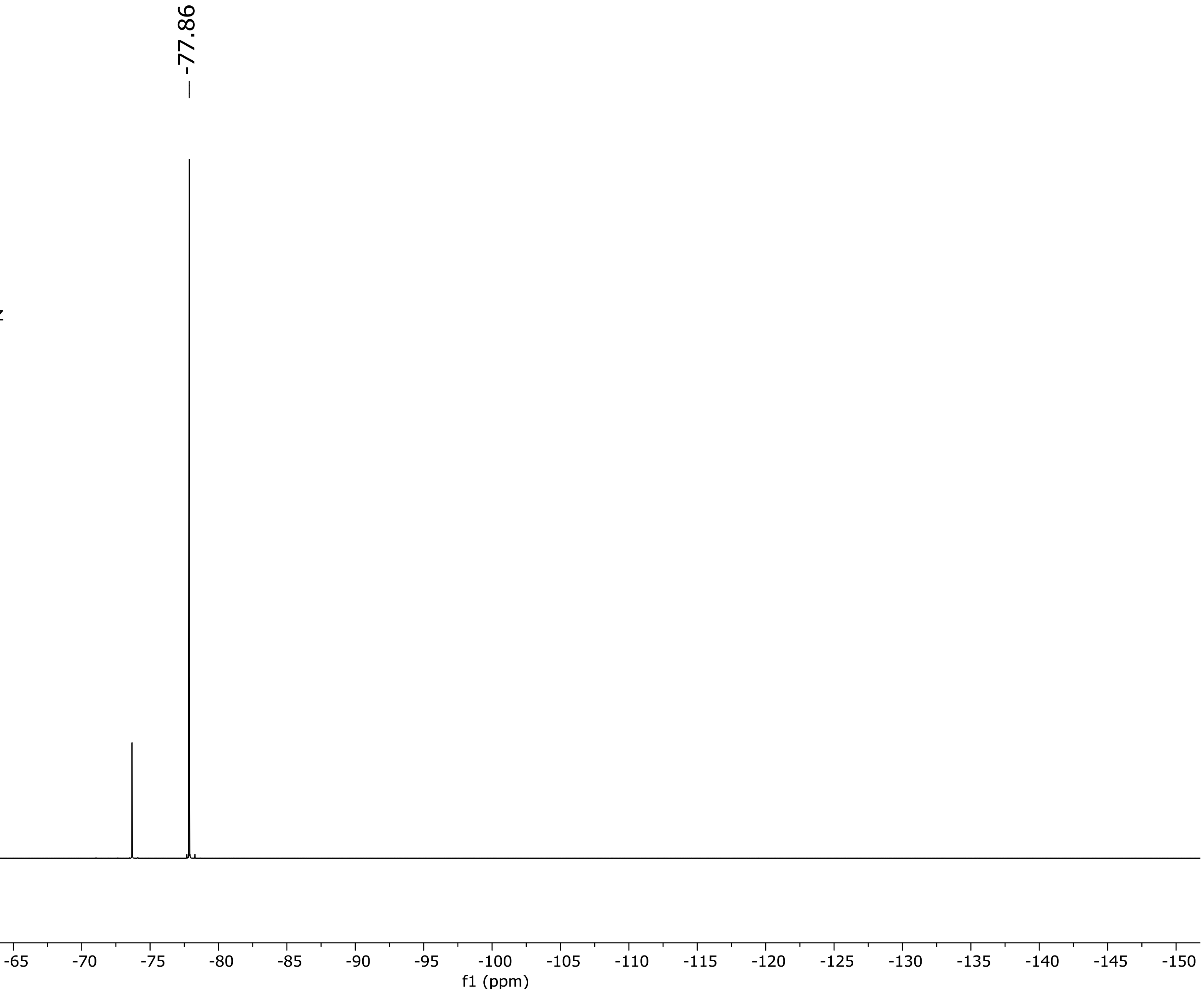

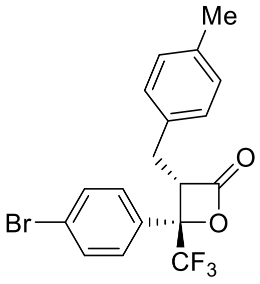


**13**

$^{13}$C, $CDCl_3$, 126 MHz

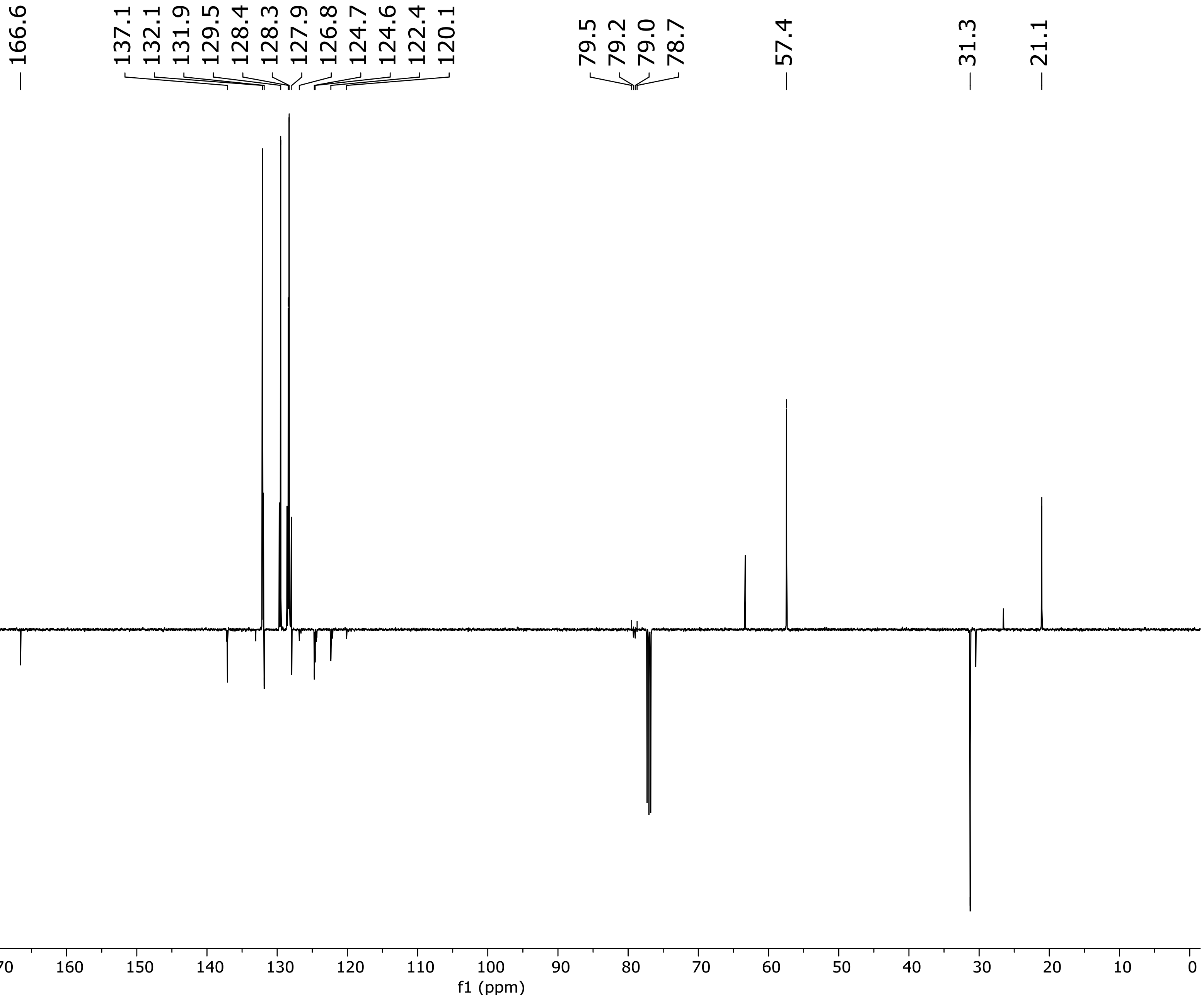

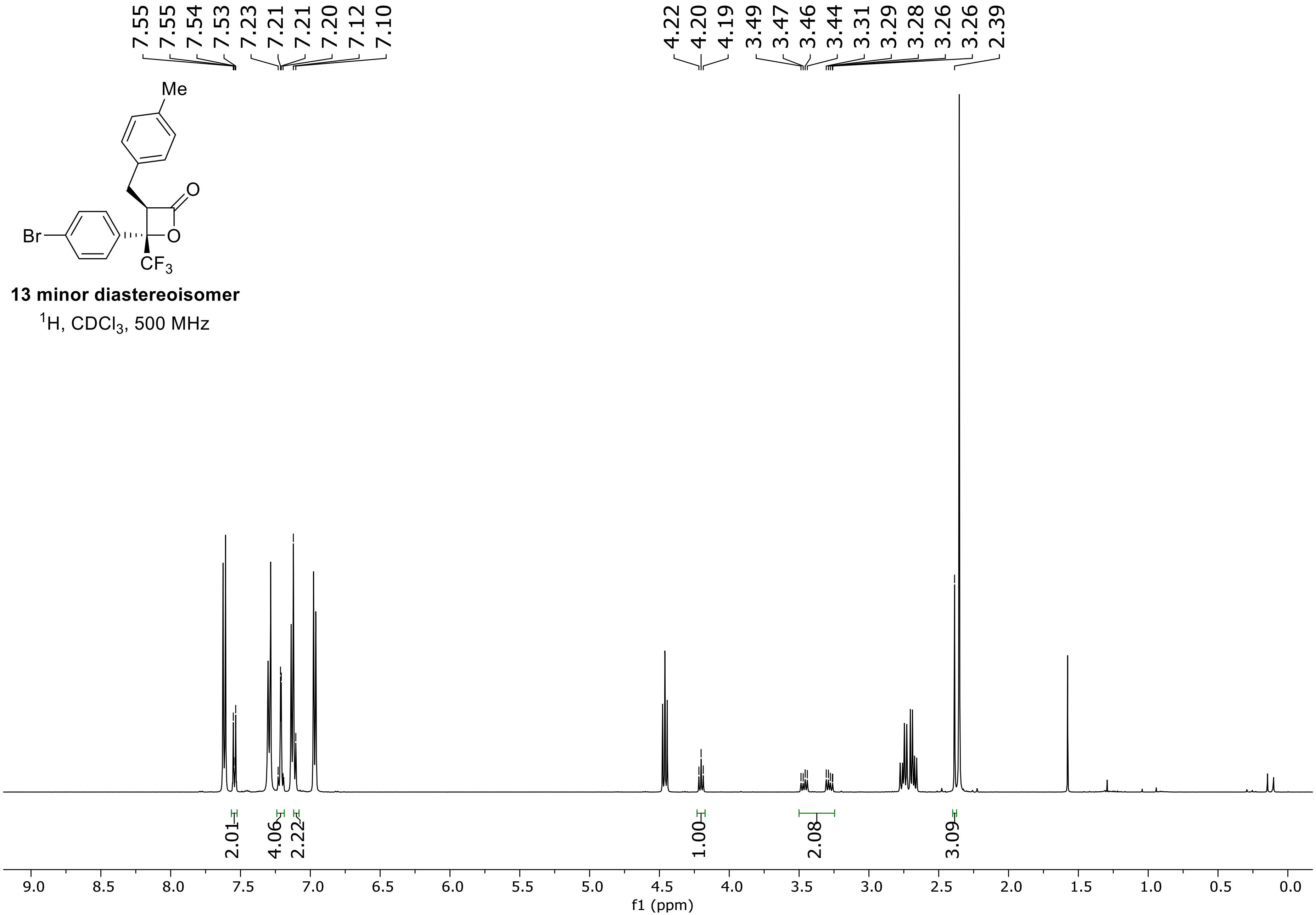

7.55
7.55
7.54
7.53
7.23
7.21
7.21
7.20
7.12
7.10
4.22
4.20
4.19
3.49
3.47
3.46
3.44
3.31
3.29
3.28
3.26
3.26
2.39
Me
O
Br
O
CF3
**13 minor diastereoisomer**
1H, CDCl3, 500 MHz
2.01
4.06
2.22
1.00
2.08
3.09
9.0
8.5
8.0
7.5
7.0
6.5
6.0
5.5
5.0
4.5
4.0
3.5
3.0
2.5
2.0
1.5
1.0
0.5
0.0
f1 (ppm)

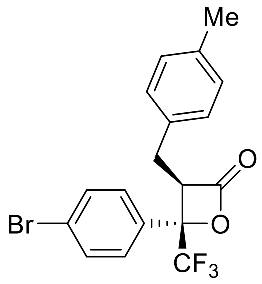


**13 minor diastereoisomer**

$^{19}F$, $CDCl_3$, 471 MHz

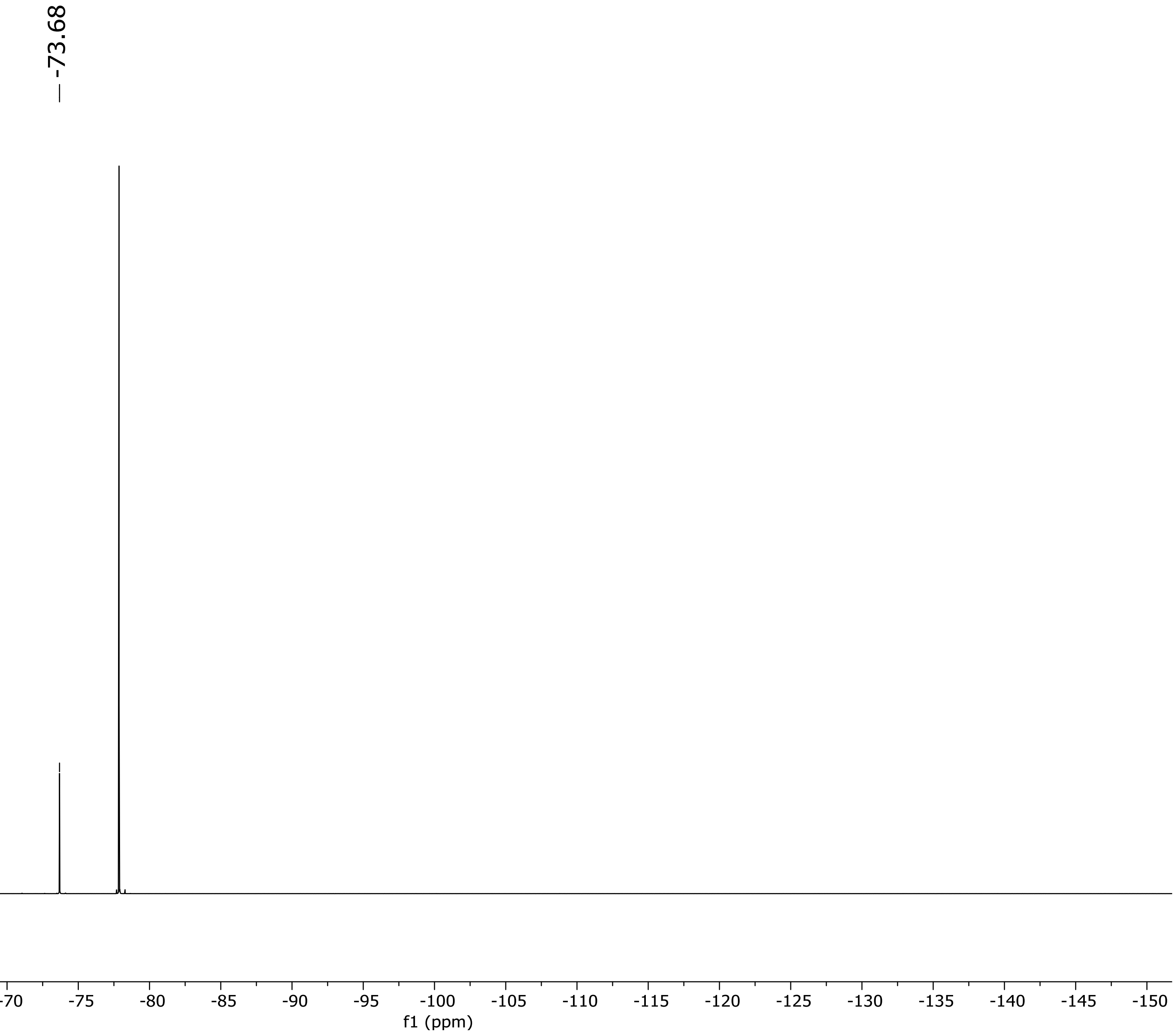

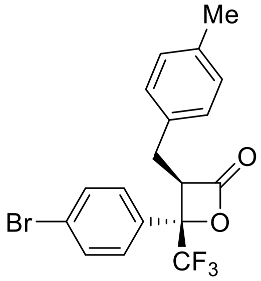


**13 minor diastereoisomer**

$^{13}C$, $CDCl_3$, 126 MHz

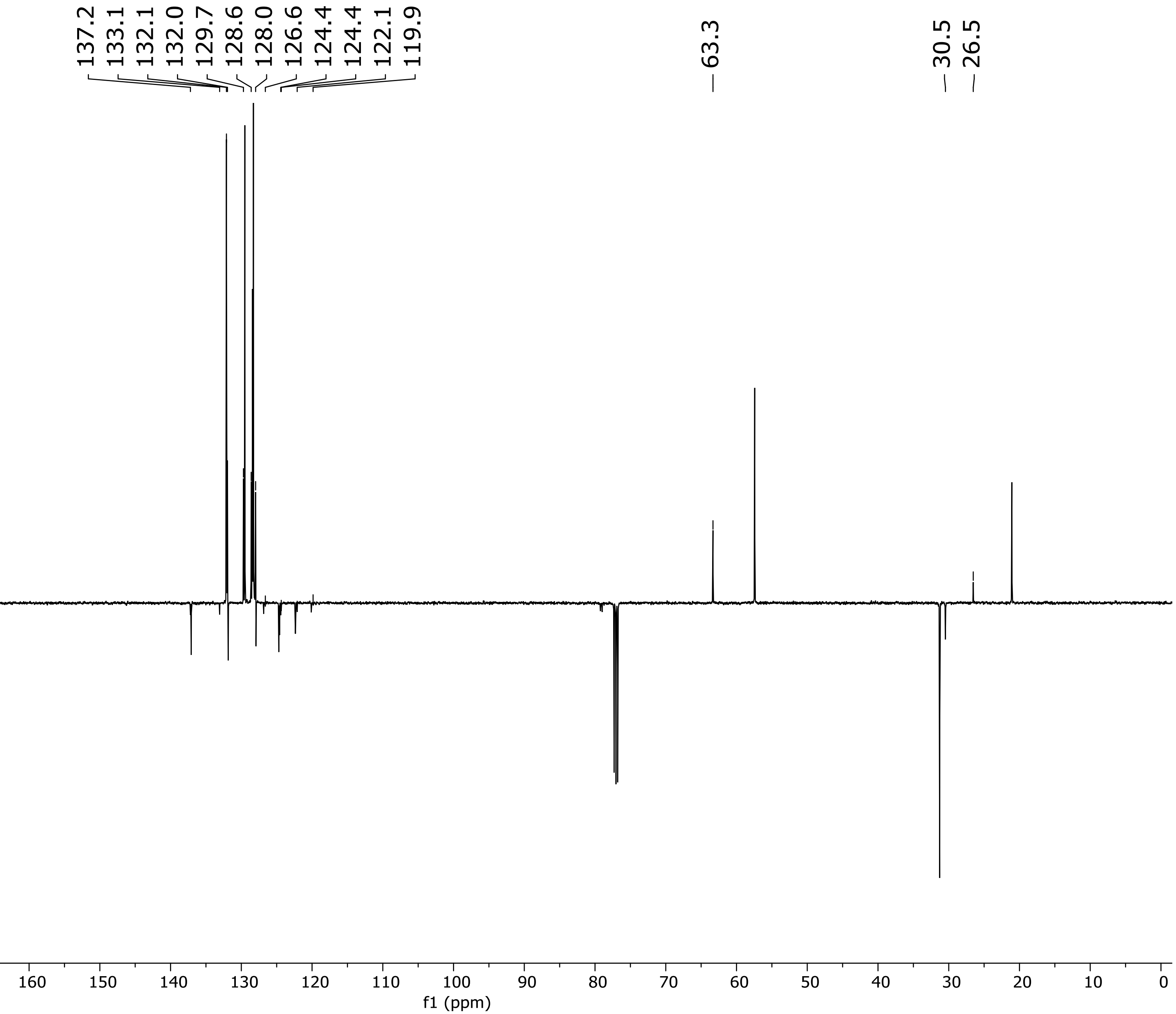

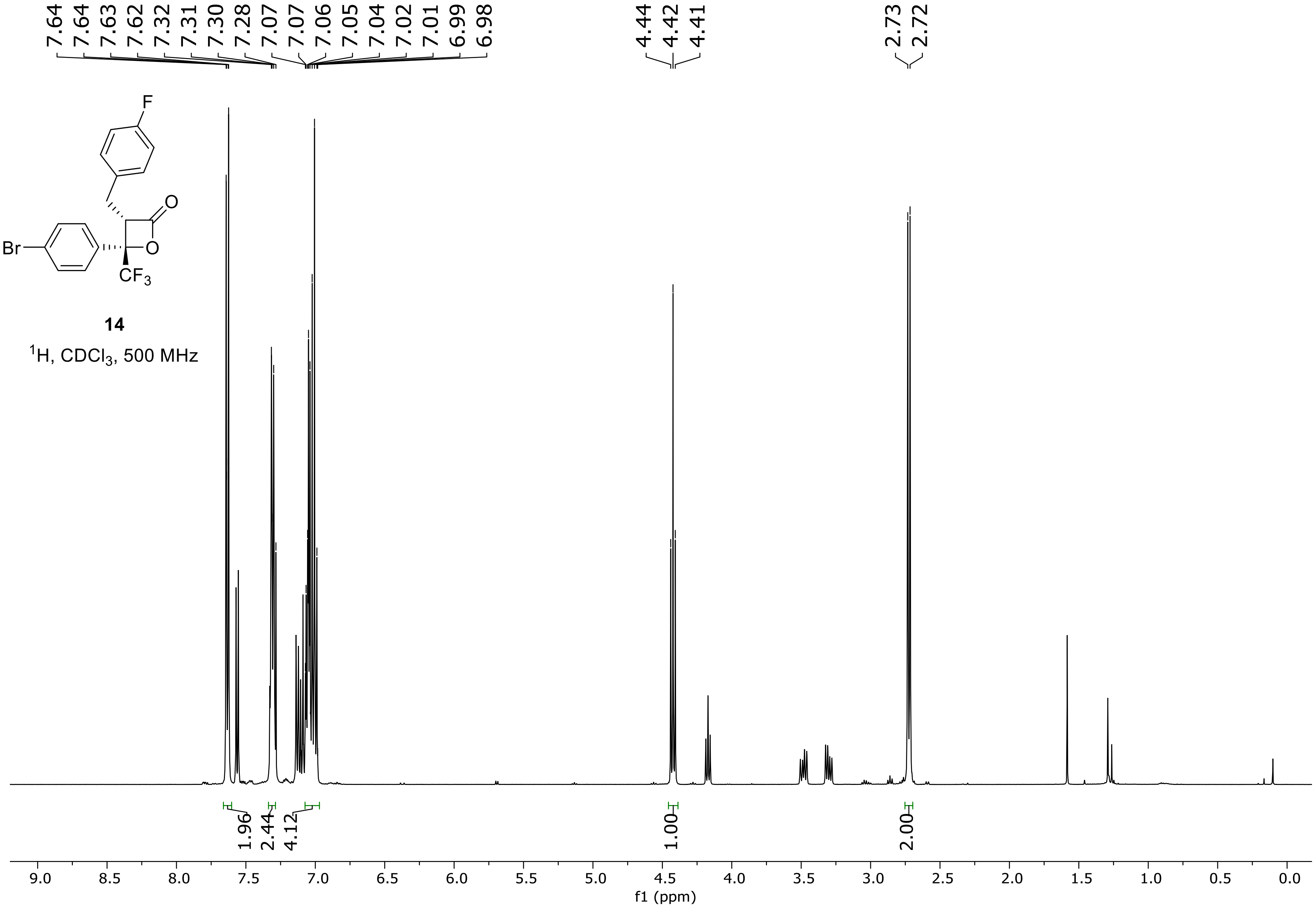
7.64
7.64
7.63
7.62
7.32
7.31
7.30
7.28
7.07
7.07
7.06
7.05
7.04
7.02
7.01
6.99
6.98
4.44
4.42
4.41
2.73
2.72
F
O
Br
O
CF3
14
1H, CDCl3, 500 MHz
1.96
2.44
4.12
1.00
2.00
9.0
8.5
8.0
7.5
7.0
6.5
6.0
5.5
5.0
4.5
4.0
3.5
3.0
2.5
2.0
1.5
1.0
0.5
0.0
f1 (ppm)

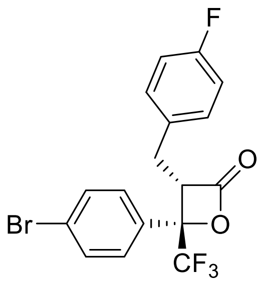


**14**

$^{19}F$, $CDCl_3$, 471 MHz

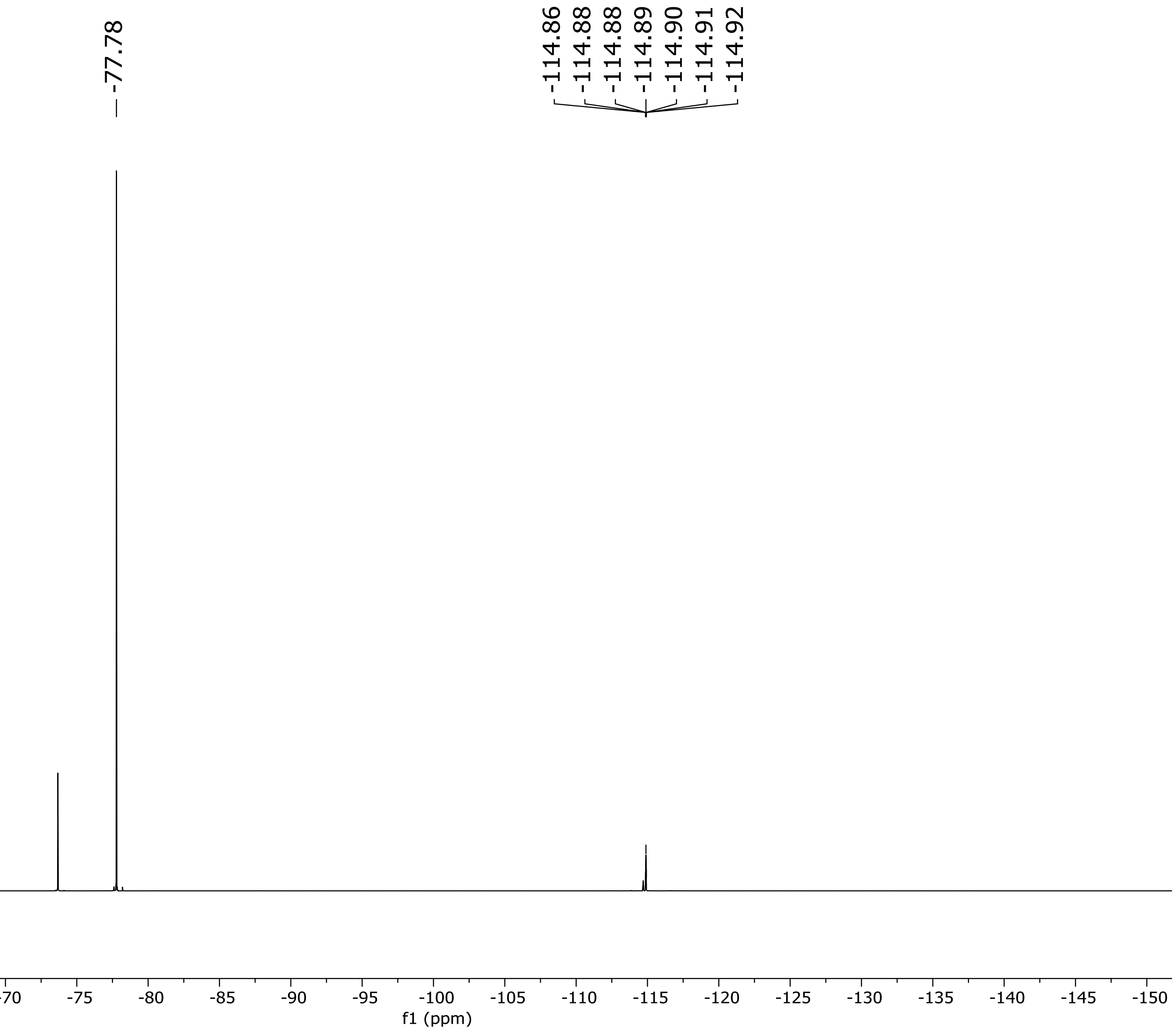

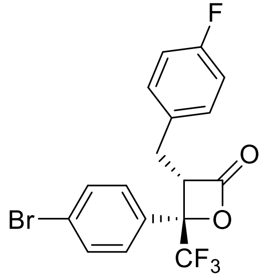

**14**

$^{13}C$, $CDCl_3$, 126 MHz

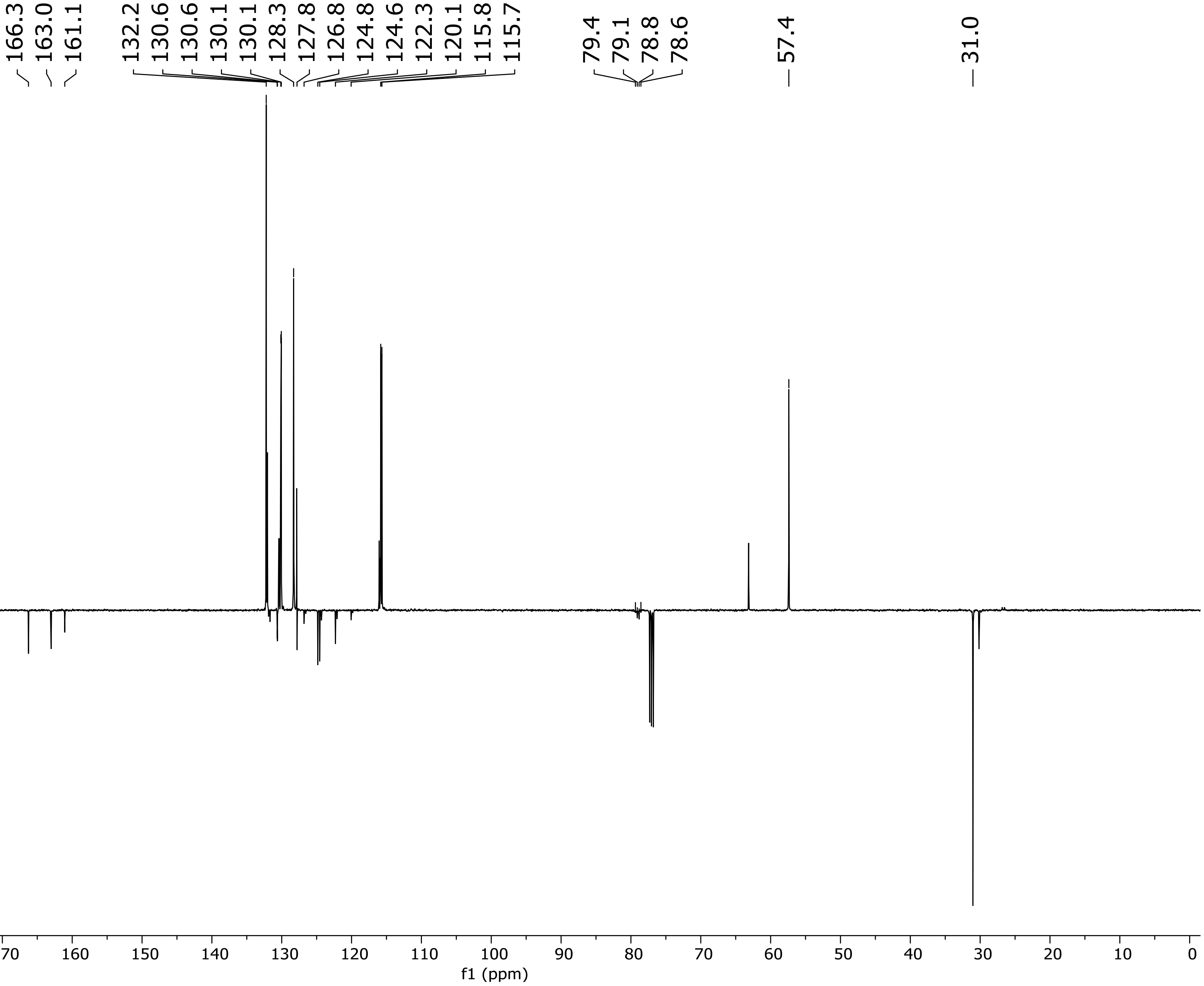

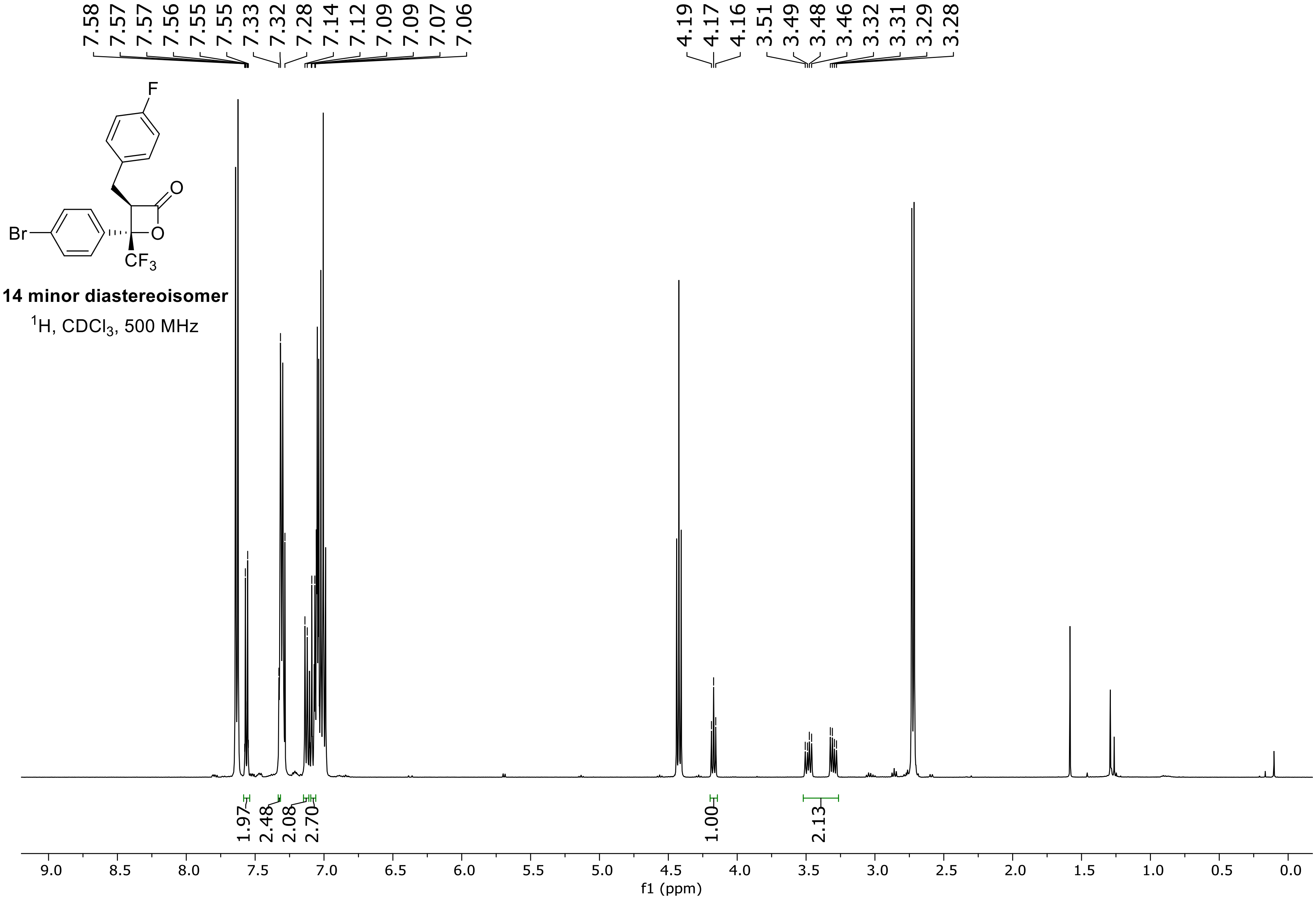
7.58 7.57 7.57 7.56 7.55 7.55 7.33 7.32 7.28 7.14 7.12 7.09 7.09 7.07 7.06
4.19 4.17 4.16 3.51 3.49 3.48 3.46 3.32 3.31 3.29 3.28
F
O
Br
O
CF3
14 minor diastereoisomer
1H, CDCl3, 500 MHz
1.97 2.48 2.08 2.70
1.00
2.13
9.0 8.5 8.0 7.5 7.0 6.5 6.0 5.5 5.0 4.5 4.0 3.5 3.0 2.5 2.0 1.5 1.0 0.5 0.0
f1 (ppm)

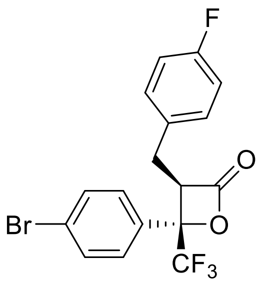


**14 minor diastereoisomer**

$^{19}F$, $CDCl_3$, 471 MHz

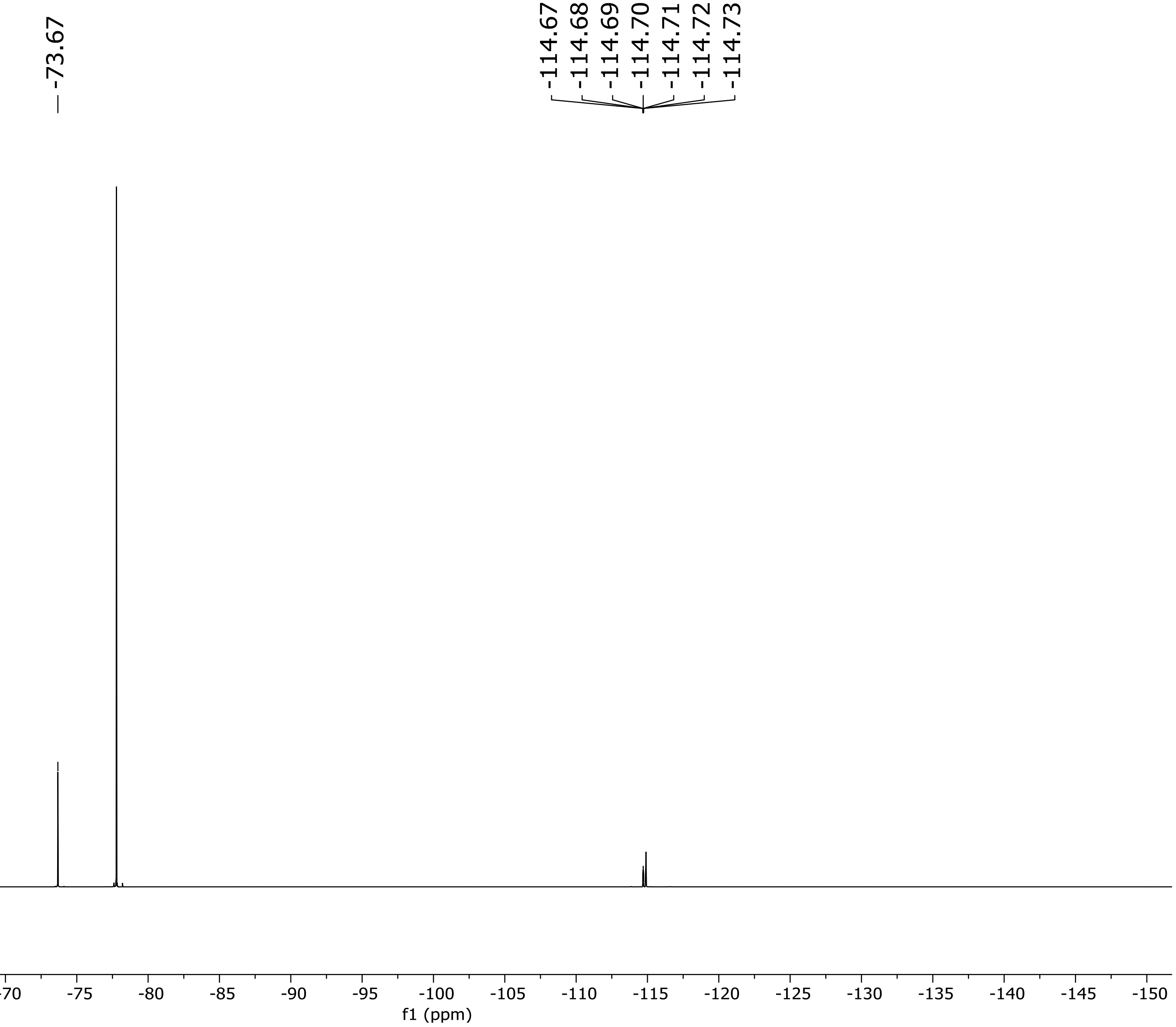

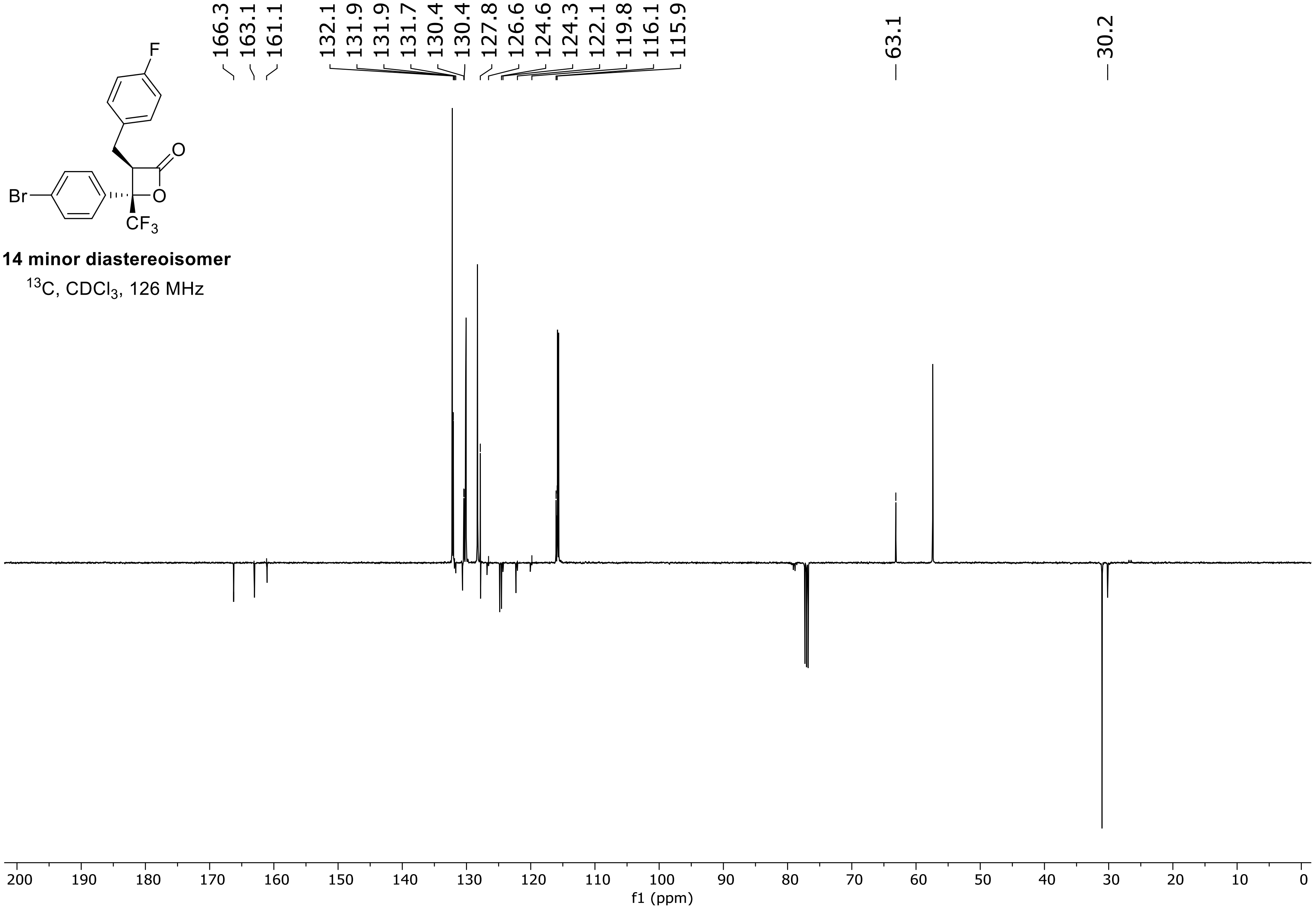


**14 minor diastereoisomer**

$^{13}C$, $CDCl_3$, 126 MHz

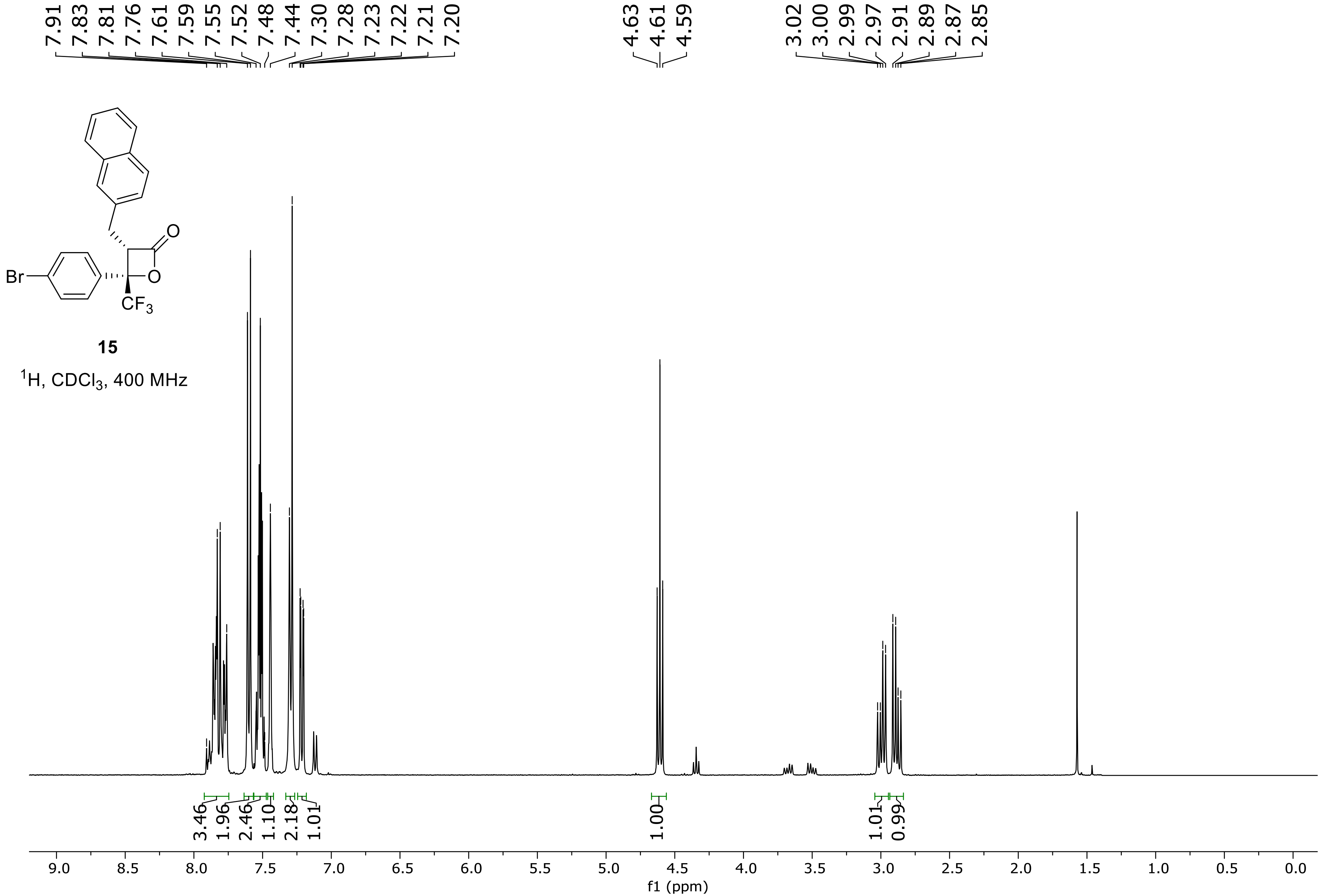
7.91
7.83
7.81
7.76
7.61
7.59
7.55
7.52
7.48
7.44
7.30
7.28
7.23
7.22
7.21
7.20
4.63
4.61
4.59
3.02
3.00
2.99
2.97
2.91
2.89
2.87
2.85
Br
O
O
CF3
15
1H, CDCl3, 400 MHz
3.46
1.96
2.46
1.10
2.18
1.01
1.00
1.01
0.99
9.0
8.5
8.0
7.5
7.0
6.5
6.0
5.5
5.0
4.5
4.0
3.5
3.0
2.5
2.0
1.5
1.0
0.5
0.0
f1 (ppm)

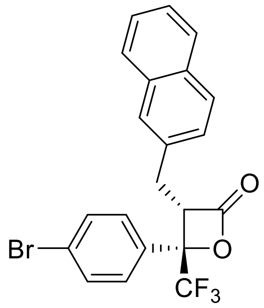

**15**

$^{19}$F, $CDCl_3$, 376 MHz

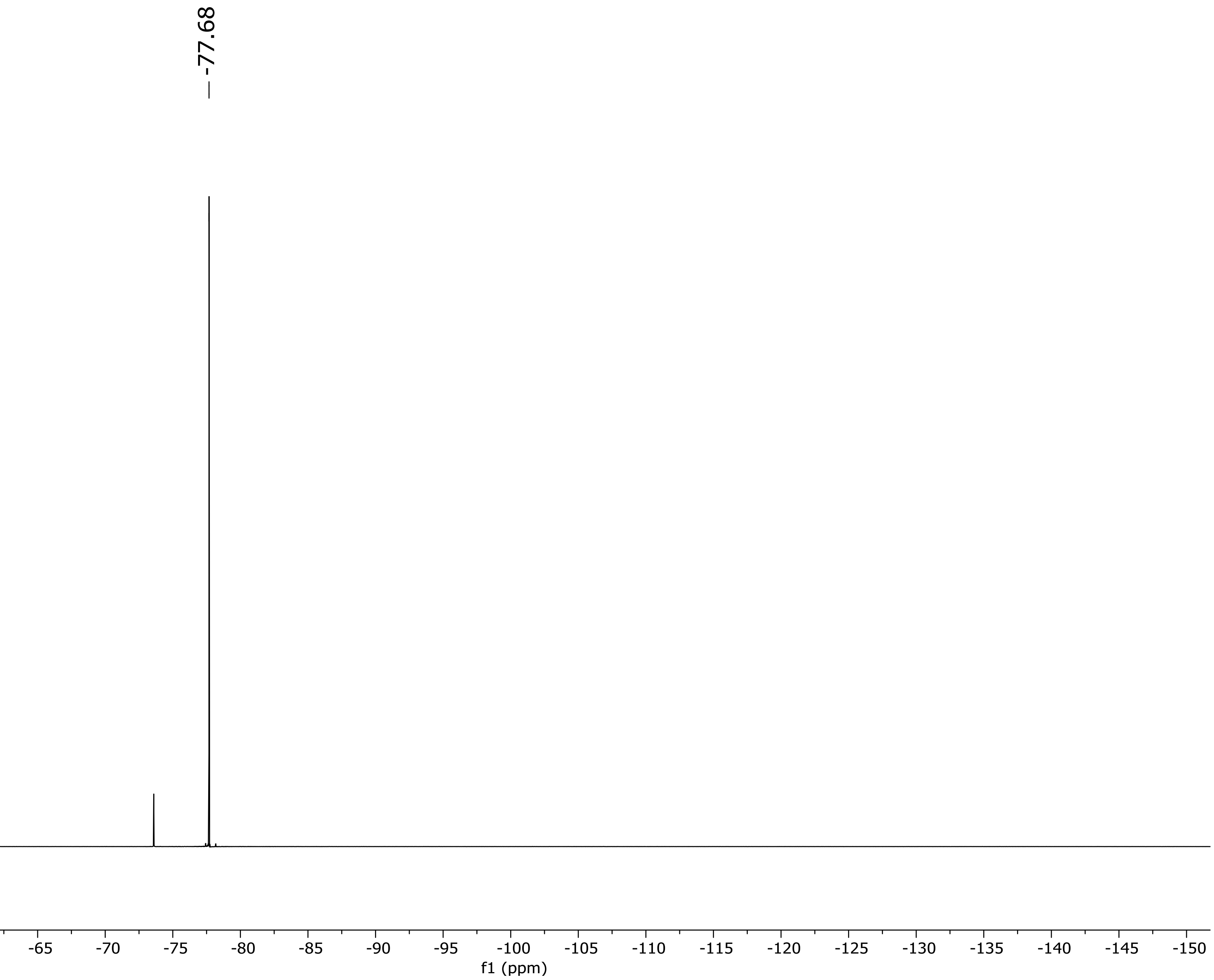

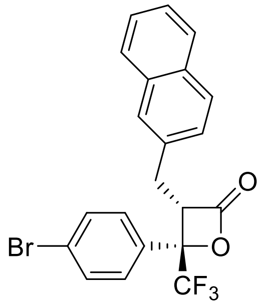


**15**

$^{13}C$, $CDCl_3$, 126 MHz

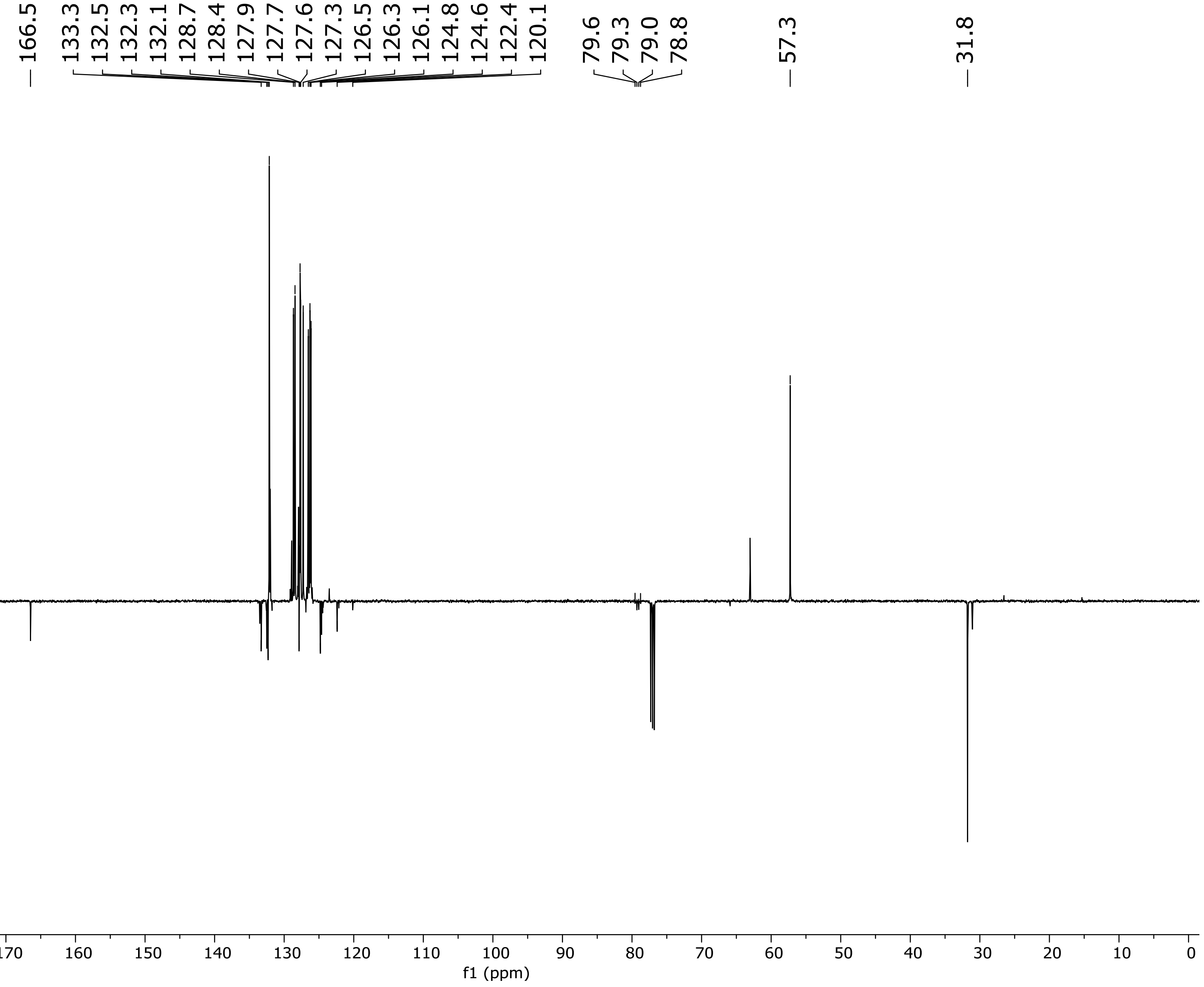

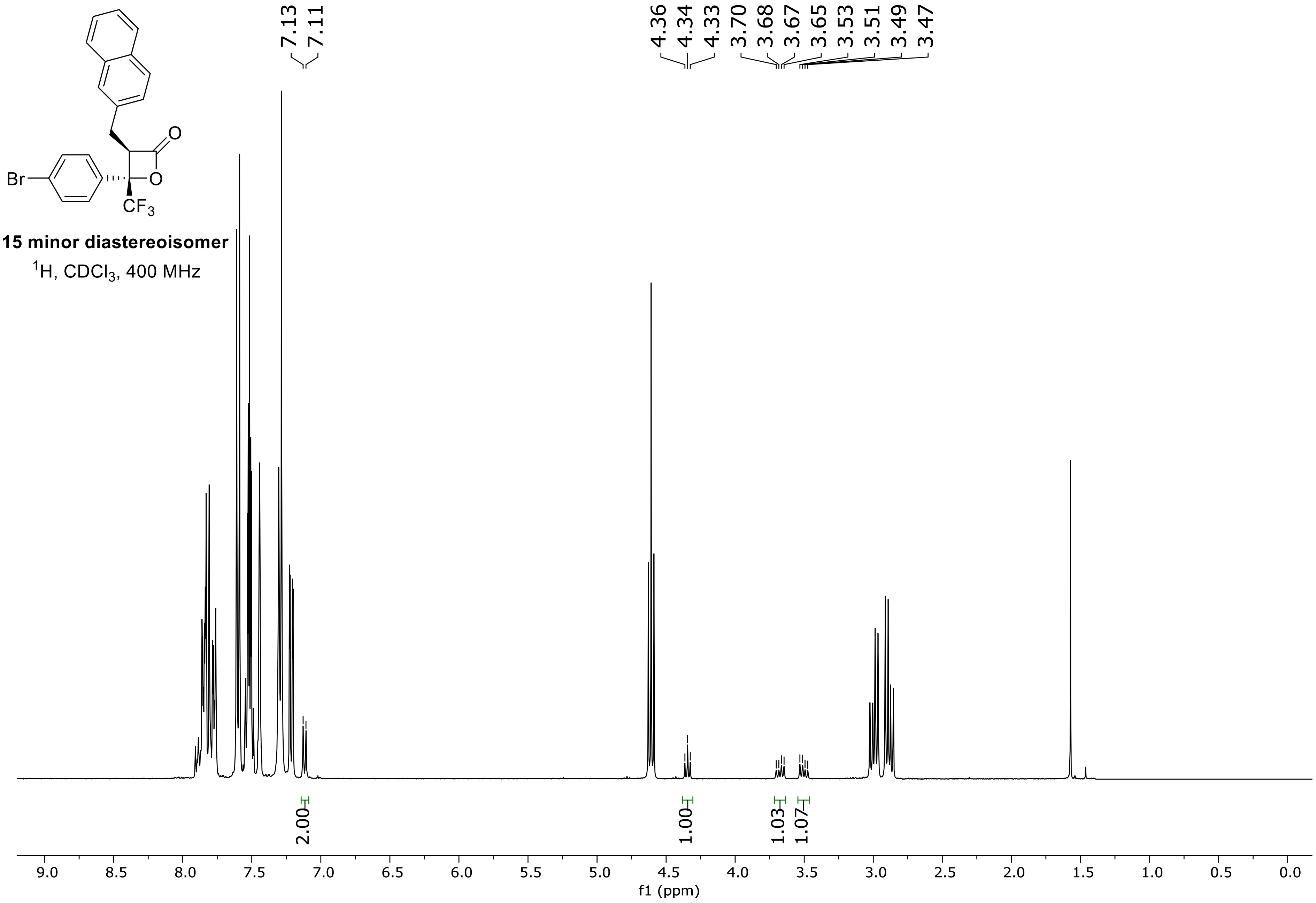
Br
O
O
CF3
15 minor diastereoisomer
1H, CDCl3, 400 MHz
7.13
7.11
4.36
4.34
4.33
3.70
3.68
3.67
3.65
3.53
3.51
3.49
3.47
2.00
1.00
1.03
1.07
9.0 8.5 8.0 7.5 7.0 6.5 6.0 5.5 5.0 4.5 4.0 3.5 3.0 2.5 2.0 1.5 1.0 0.5 0.0
f1 (ppm)

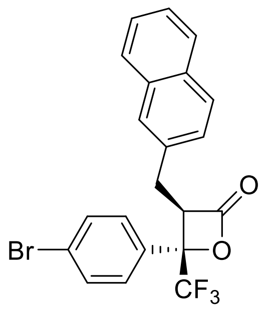

**15 minor diastereoisomer**

$^{19}F$, $CDCl_3$, 376 MHz

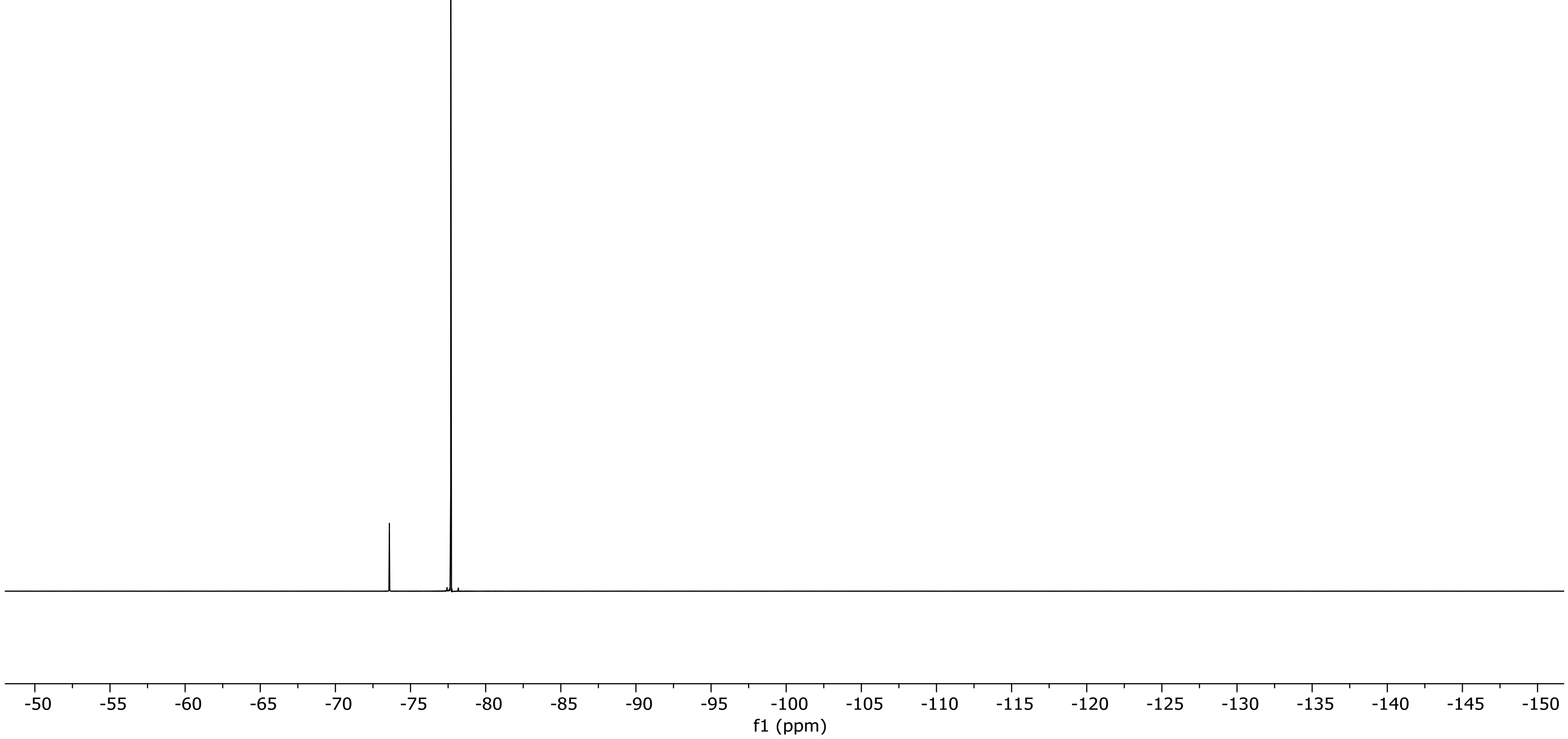

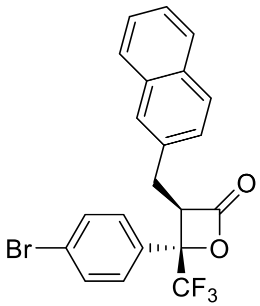

**15 minor diastereoisomer**

$^{1}H$, $CDCl_3$, 400 MHz

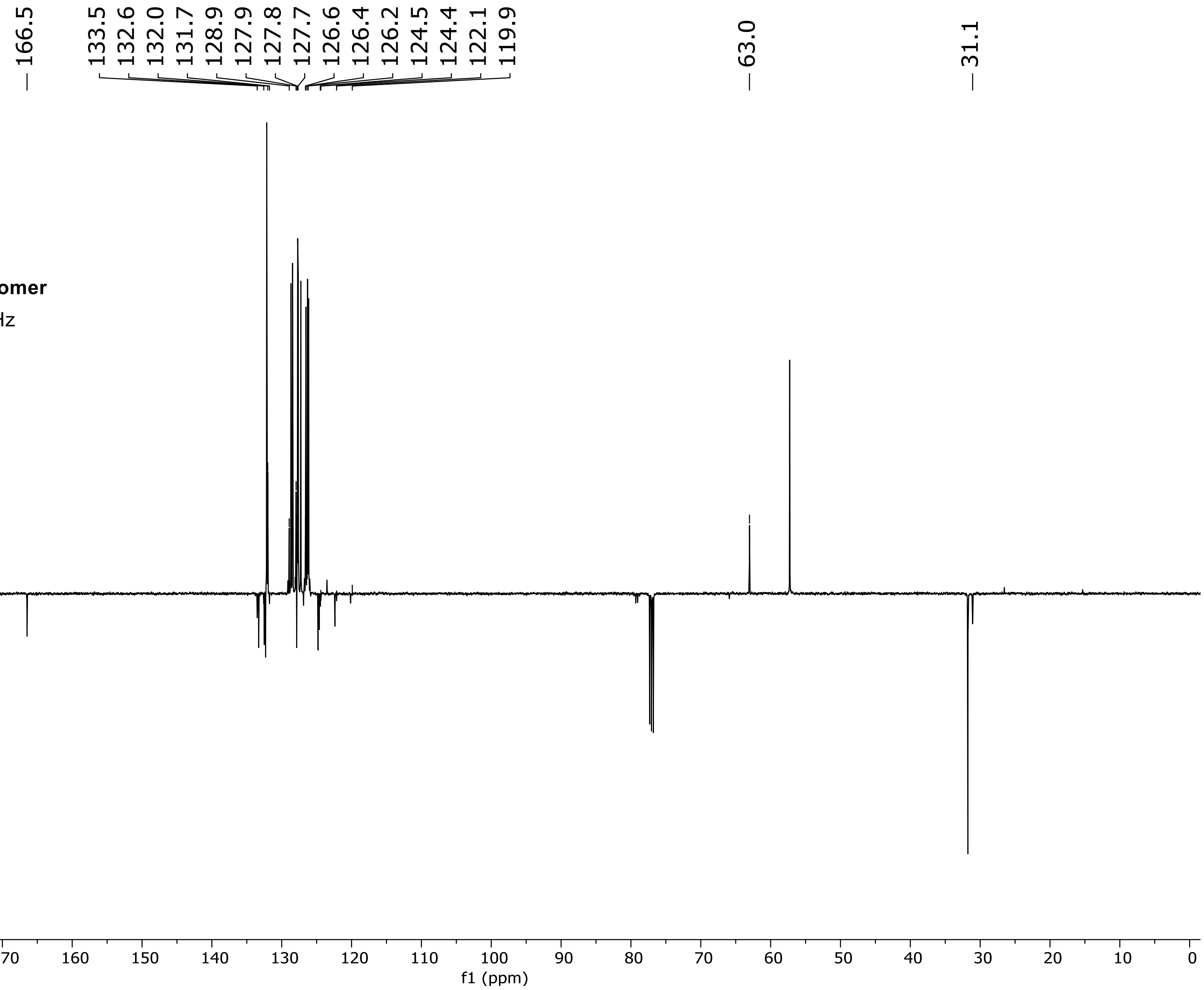

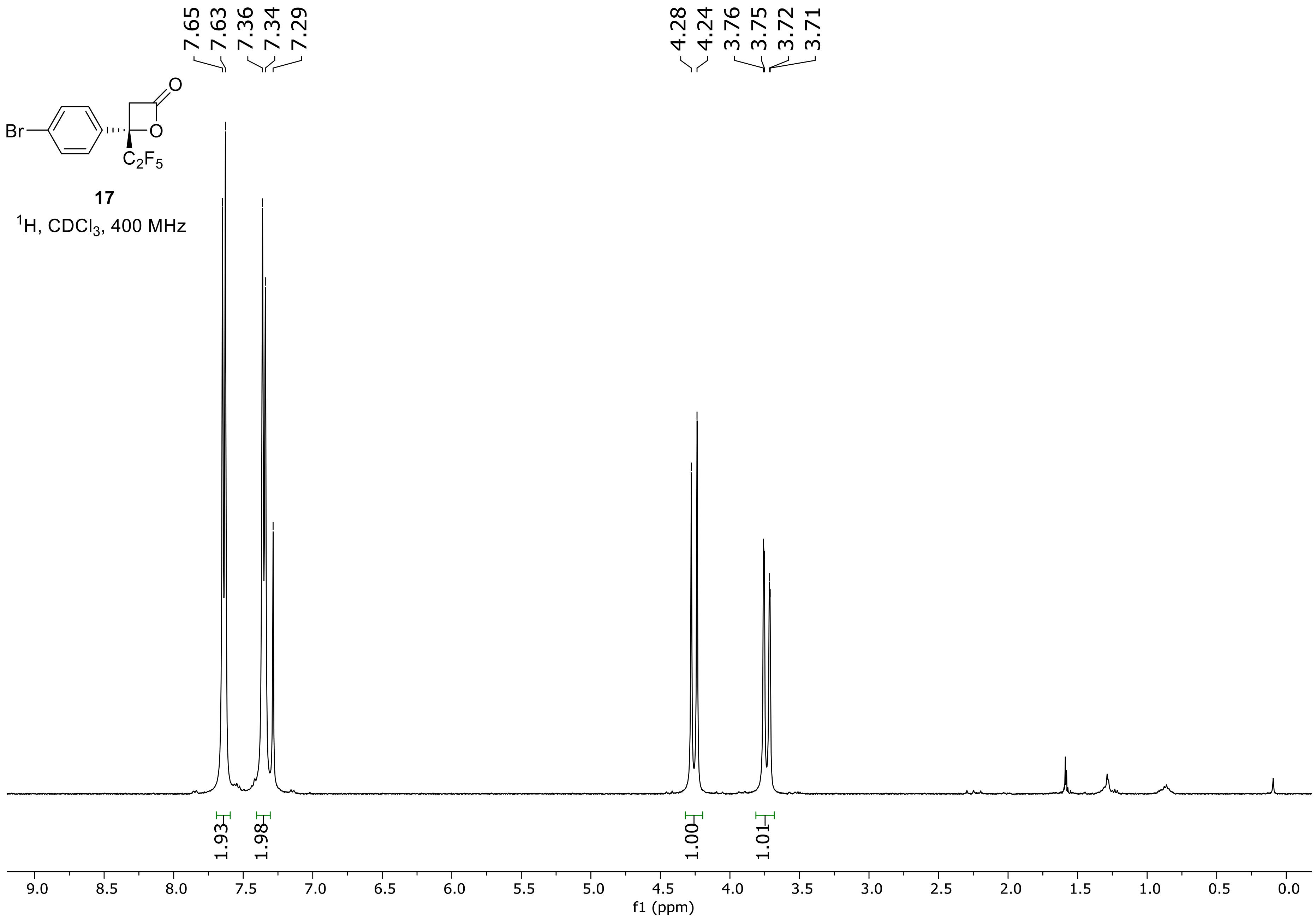

Br
O
O
C2F5
17
1H, CDCl3, 400 MHz
7.65
7.63
7.36
7.34
7.29
4.28
4.24
3.76
3.75
3.72
3.71
1.93
1.98
1.00
1.01
9.0
8.5
8.0
7.5
7.0
6.5
6.0
5.5
5.0
4.5
4.0
3.5
3.0
2.5
2.0
1.5
1.0
0.5
0.0
f1 (ppm)

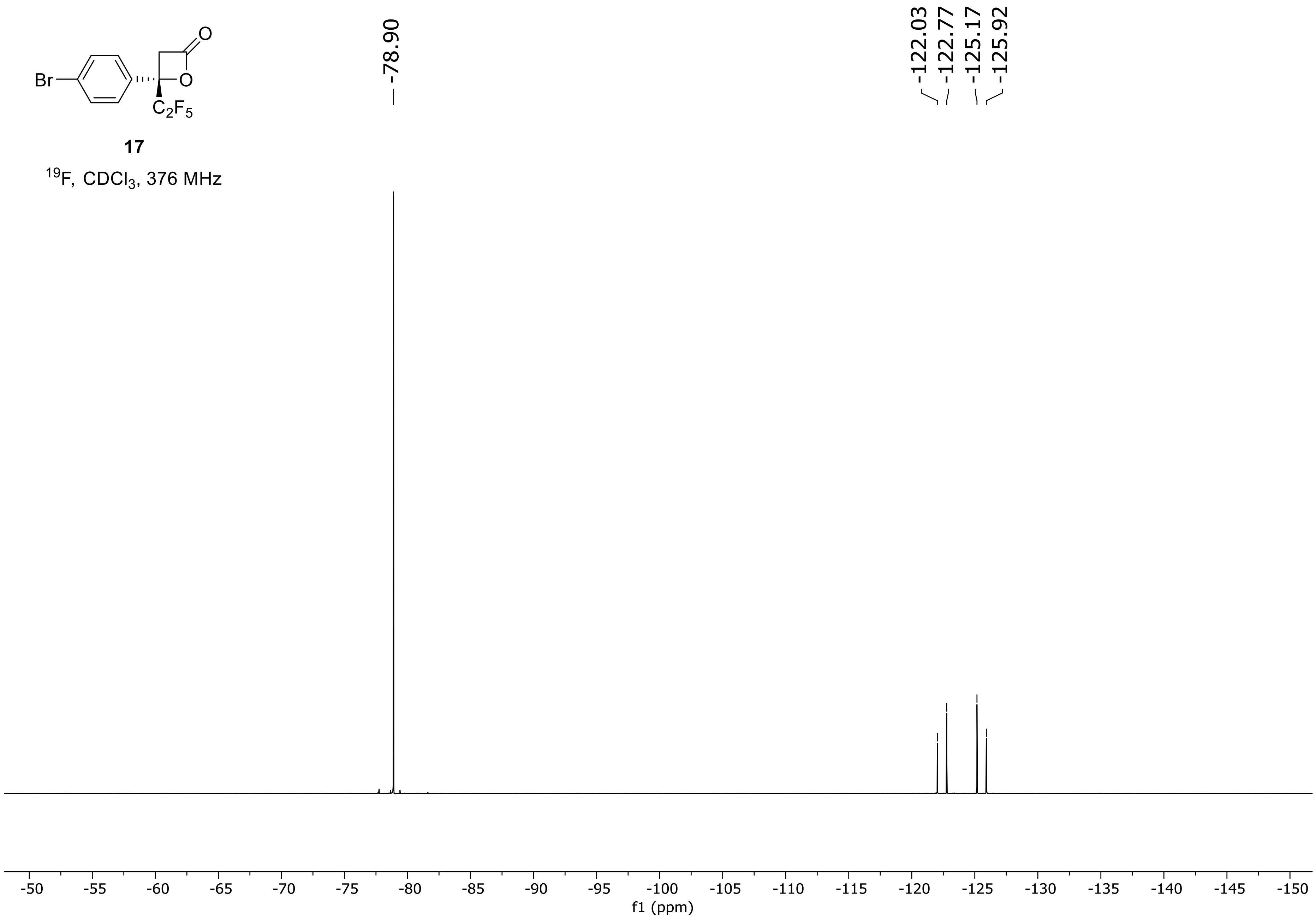
Br
O
O
$C_2F_5$
17
$^{19}F$, $CDCl_3$, 376 MHz
-78.90
-122.03
-122.77
-125.17
-125.92
-50
-55
-60
-65
-70
-75
-80
-85
-90
-95
-100
-105
-110
-115
-120
-125
-130
-135
-140
-145
-150
f1 (ppm)

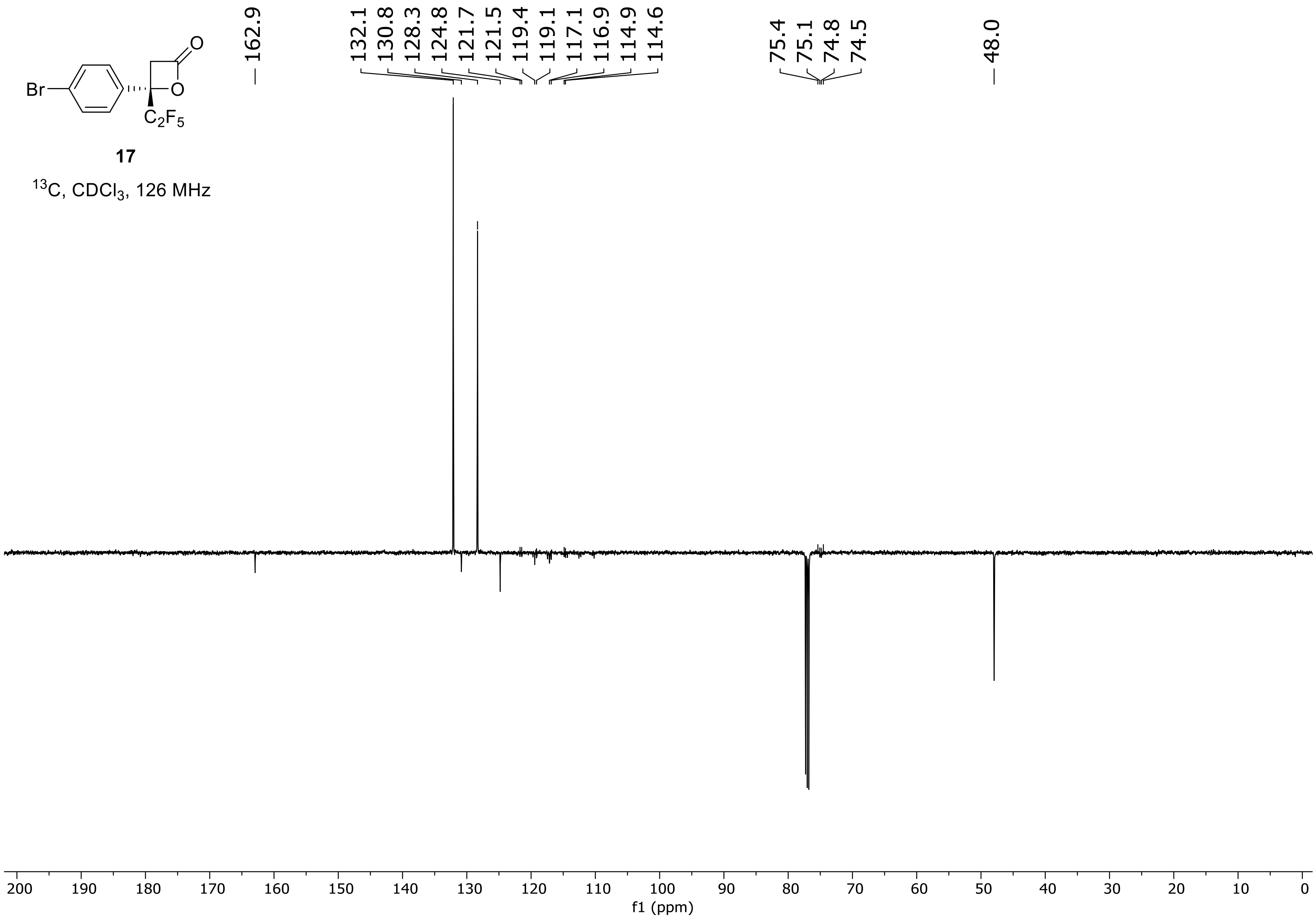
Br
O
O
C2F5
17
13C, CDCl3, 126 MHz
162.9
132.1
130.8
128.3
124.8
121.7
121.5
119.4
119.1
117.1
116.9
114.9
114.6
75.4
75.1
74.8
74.5
48.0
200 190 180 170 160 150 140 130 120 110 100 90 80 70 60 50 40 30 20 10 0
f1 (ppm)

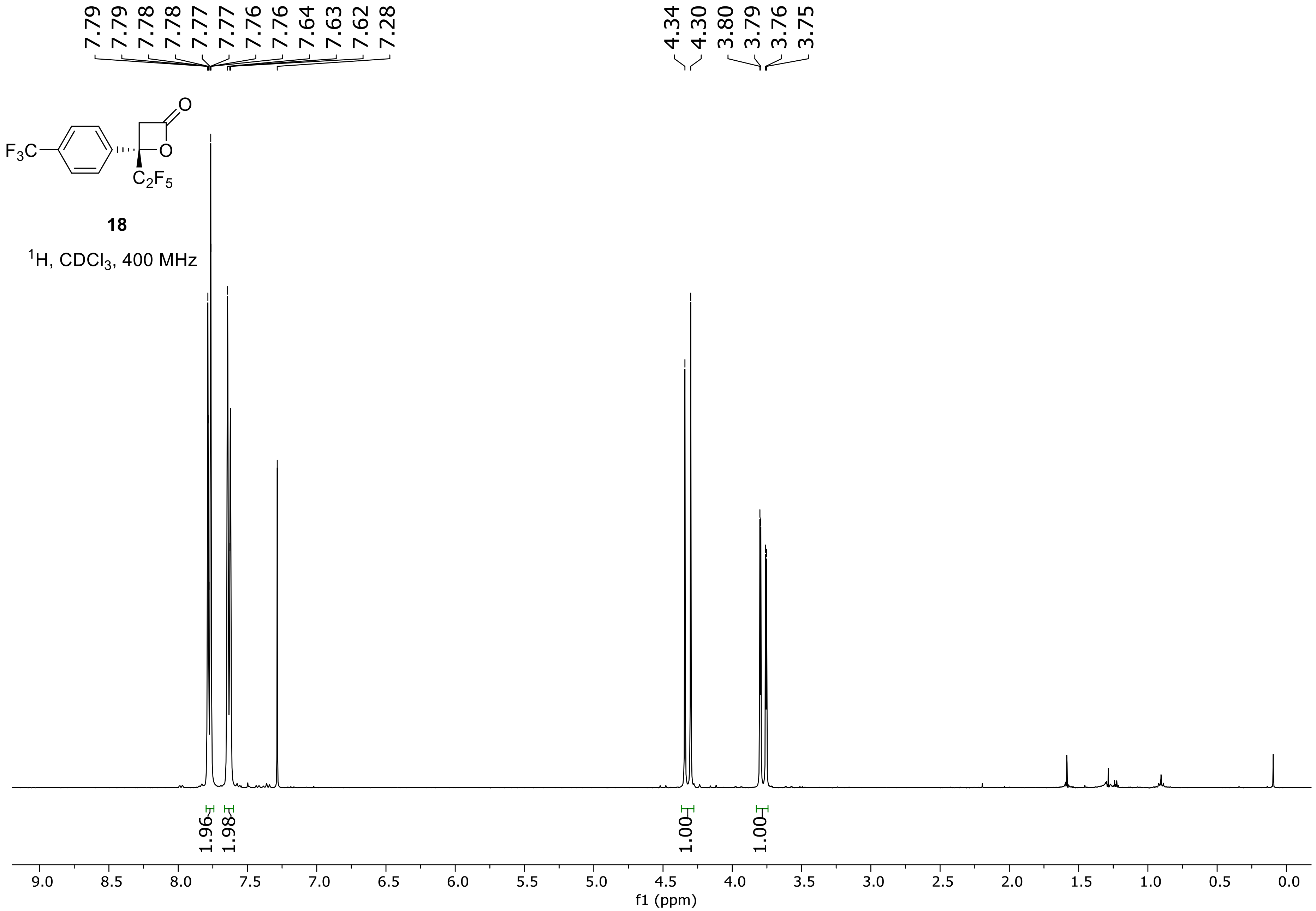
7.79
7.79
7.78
7.78
7.77
7.77
7.76
7.76
7.64
7.63
7.62
7.28
4.34
4.30
3.80
3.79
3.76
3.75
F3C
O
O
C2F5
18
1H, CDCl3, 400 MHz
1.96
1.98
1.00
1.00
9.0
8.5
8.0
7.5
7.0
6.5
6.0
5.5
5.0
4.5
4.0
3.5
3.0
2.5
2.0
1.5
1.0
0.5
0.0
f1 (ppm)

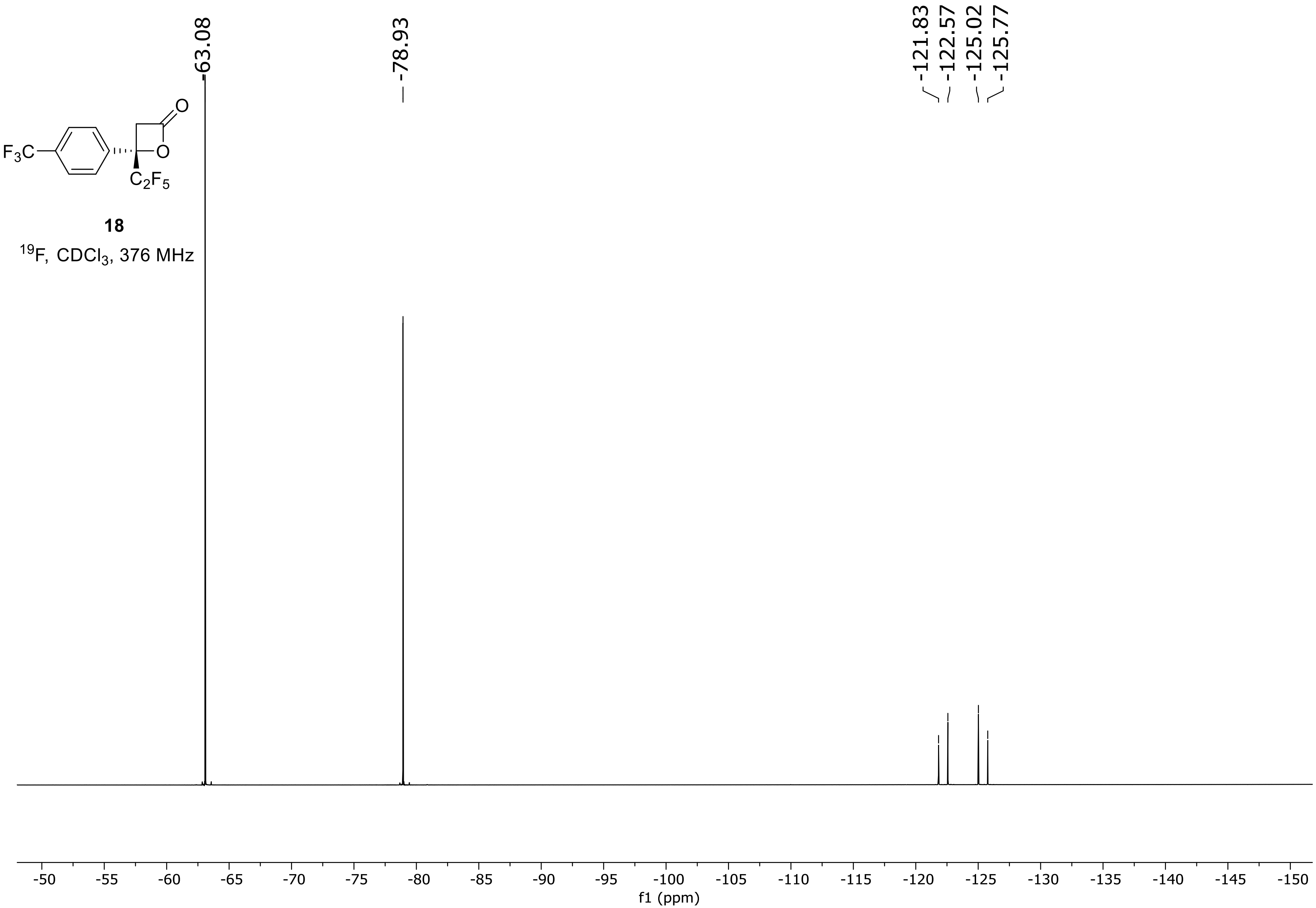


**18**

$^{19}F$, $CDCl_3$, 376 MHz

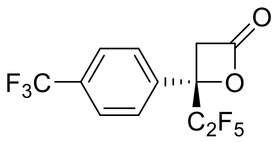


**18**

$^{13}$C, $CDCl_3$, 126 MHz

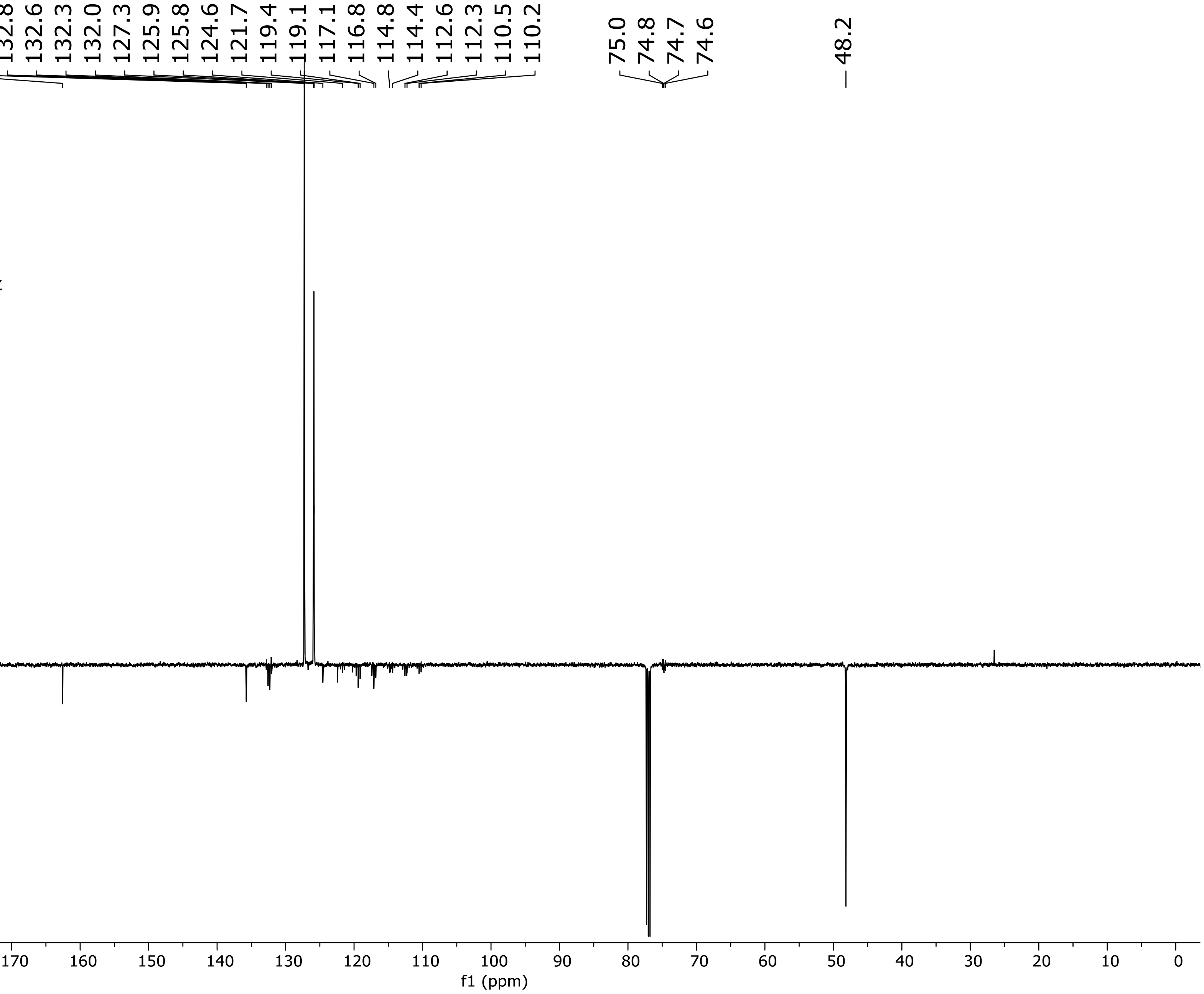

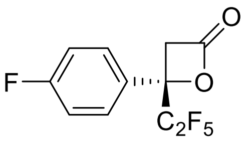


**19**

$^1$H, $CDCl_3$, 400 MHz

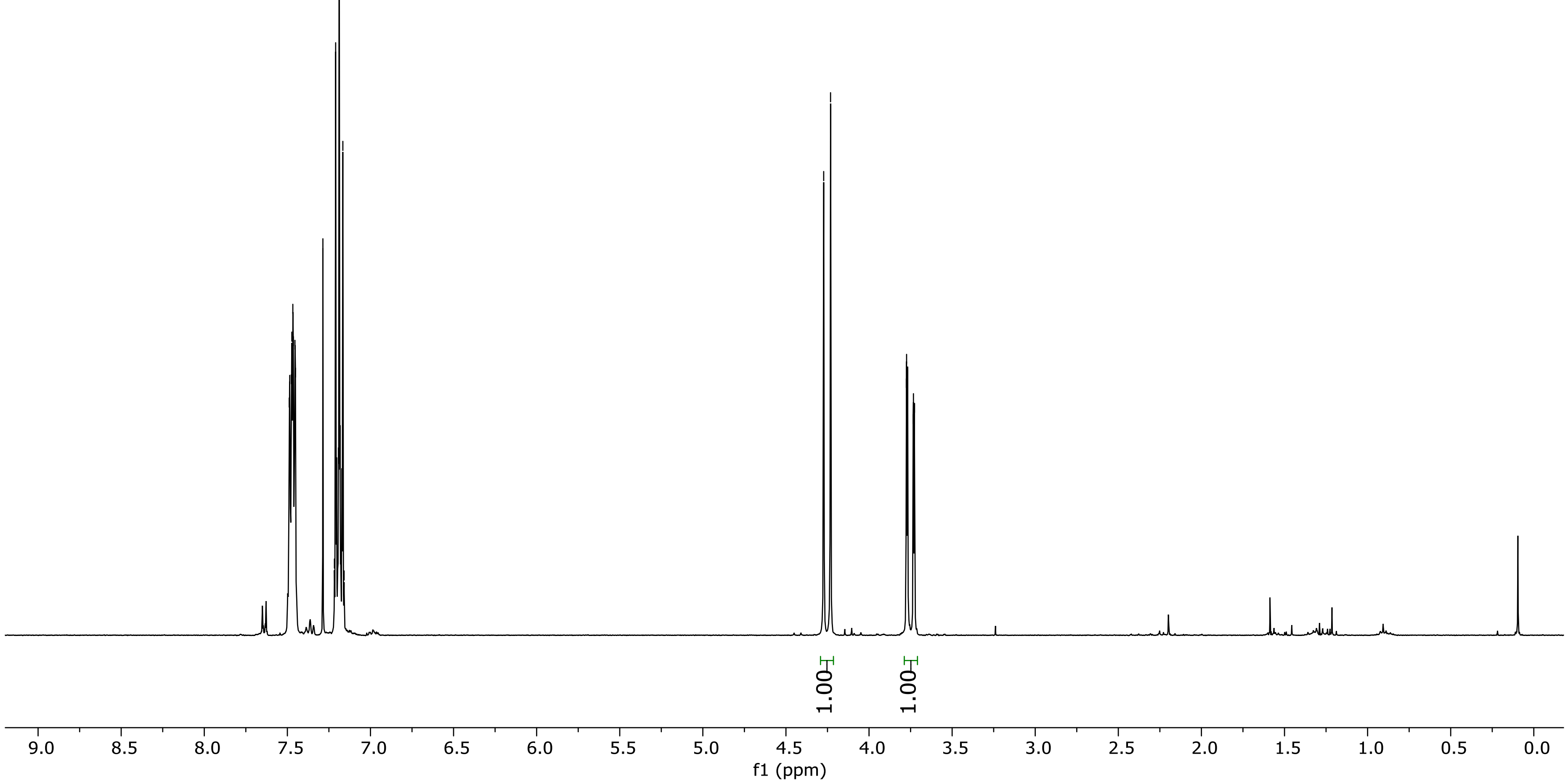

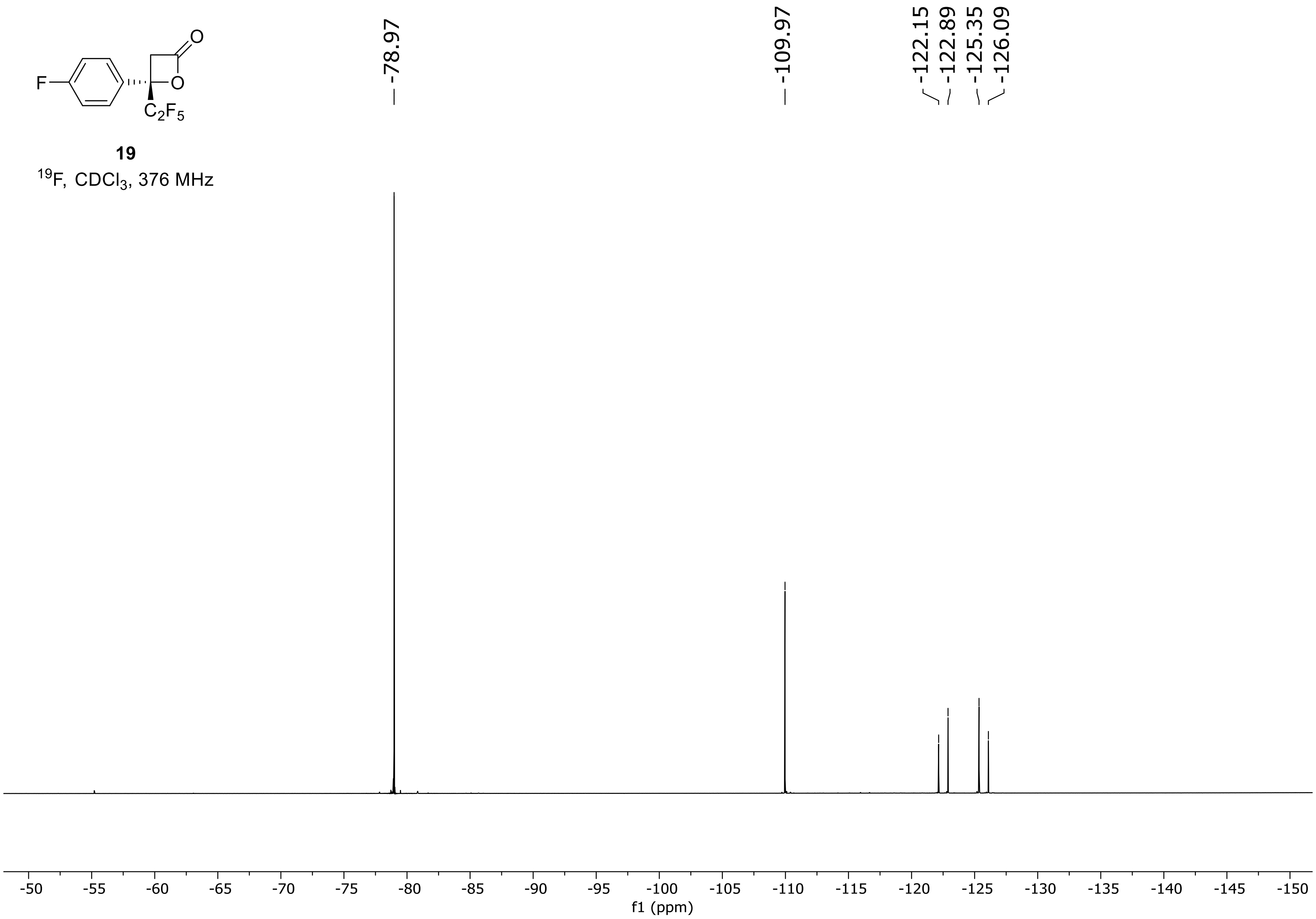


**19**

$^{19}F$, $CDCl_3$, 376 MHz

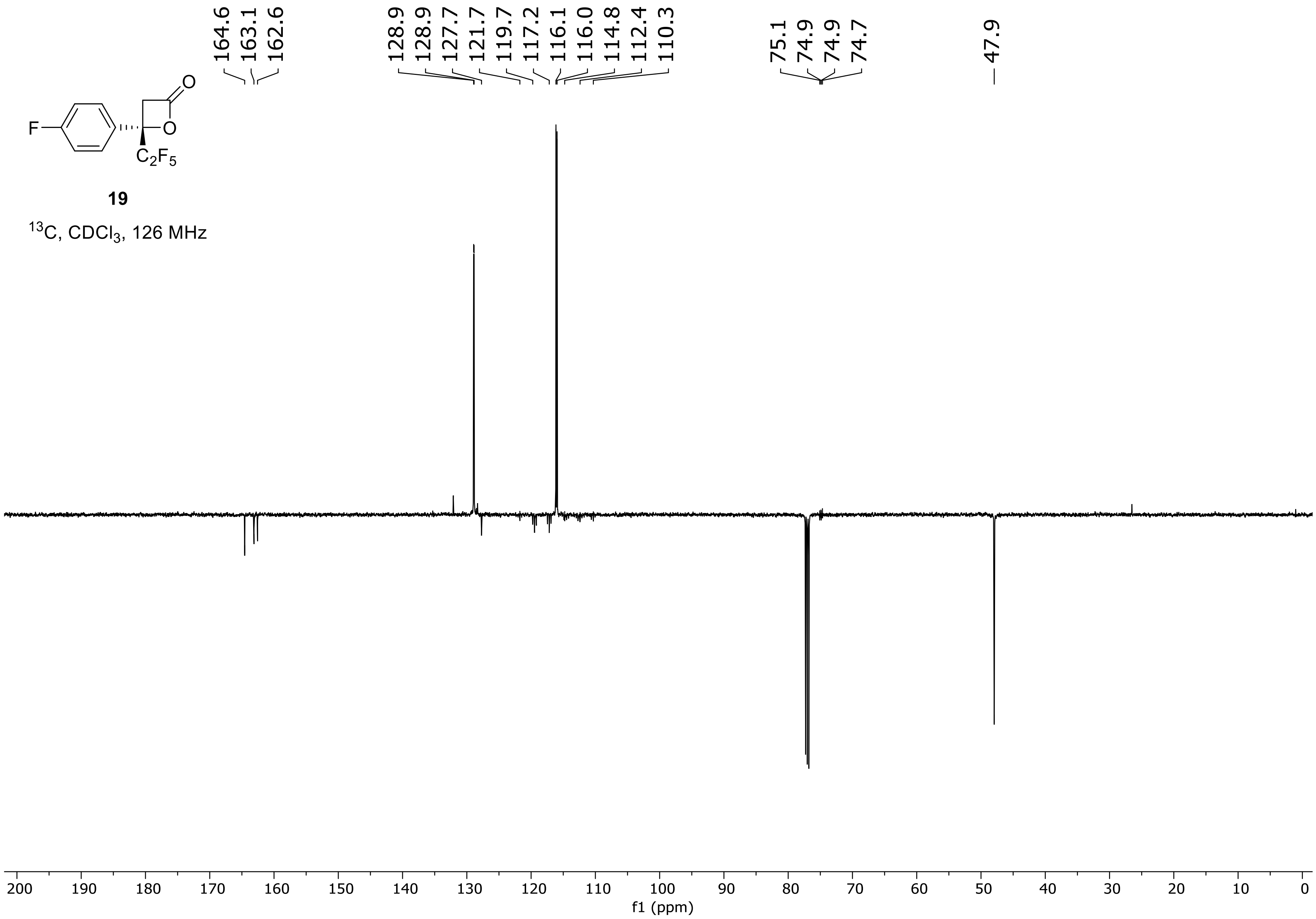

164.6
163.1
162.6
128.9
128.9
127.7
121.7
119.7
117.2
116.1
116.0
114.8
112.4
110.3
75.1
74.9
74.9
74.7
47.9
F
O
O
C2F5
19
13C, CDCl3, 126 MHz
200
190
180
170
160
150
140
130
120
110
100
90
80
70
60
50
40
30
20
10
0
f1 (ppm)

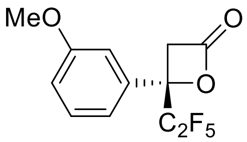


**20**

$^{1}H$, $CDCl_3$, 400 MHz

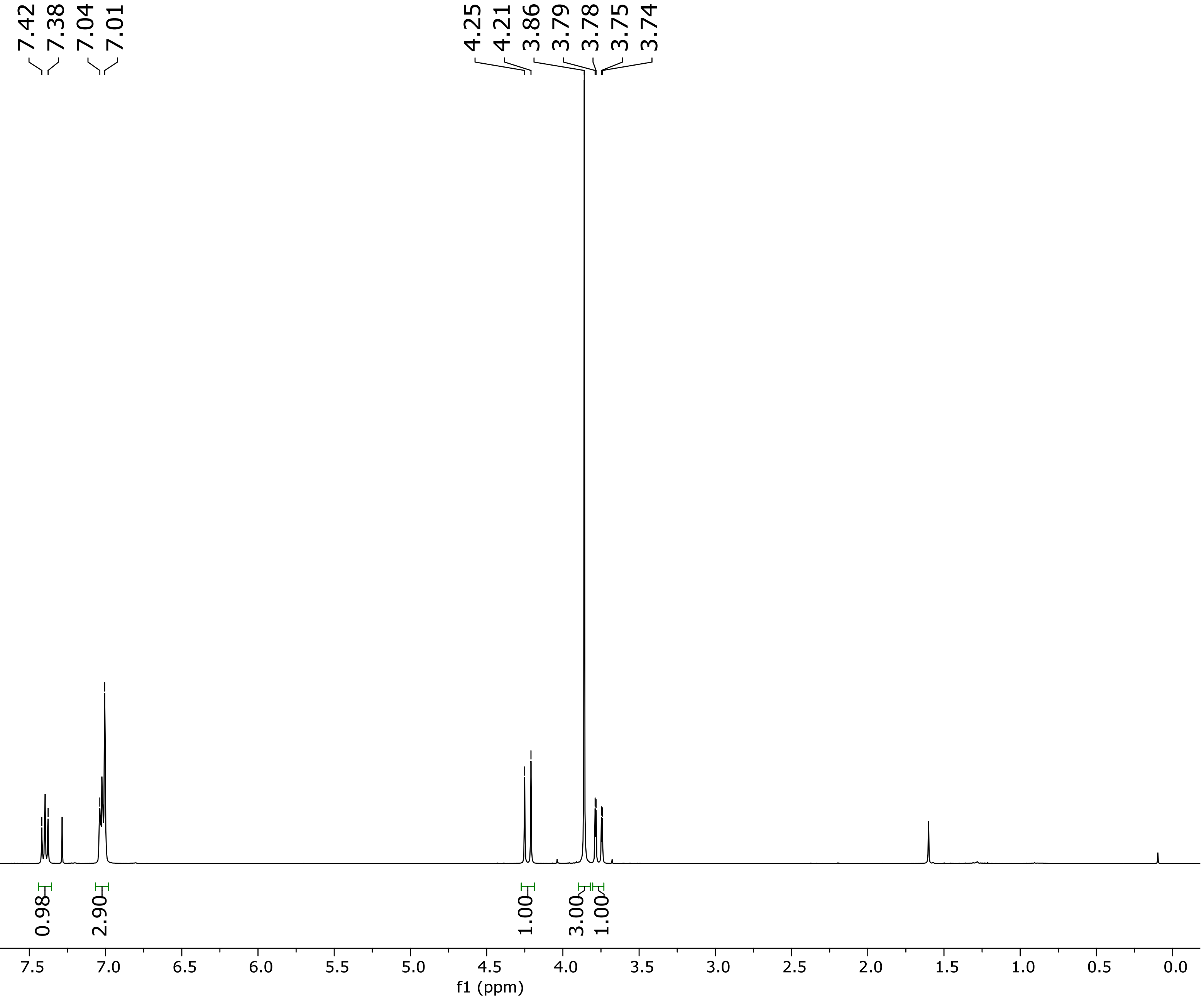

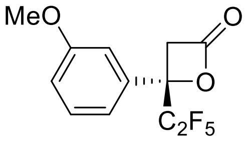


**20**

$^{19}F$, $CDCl_3$, 376 MHz

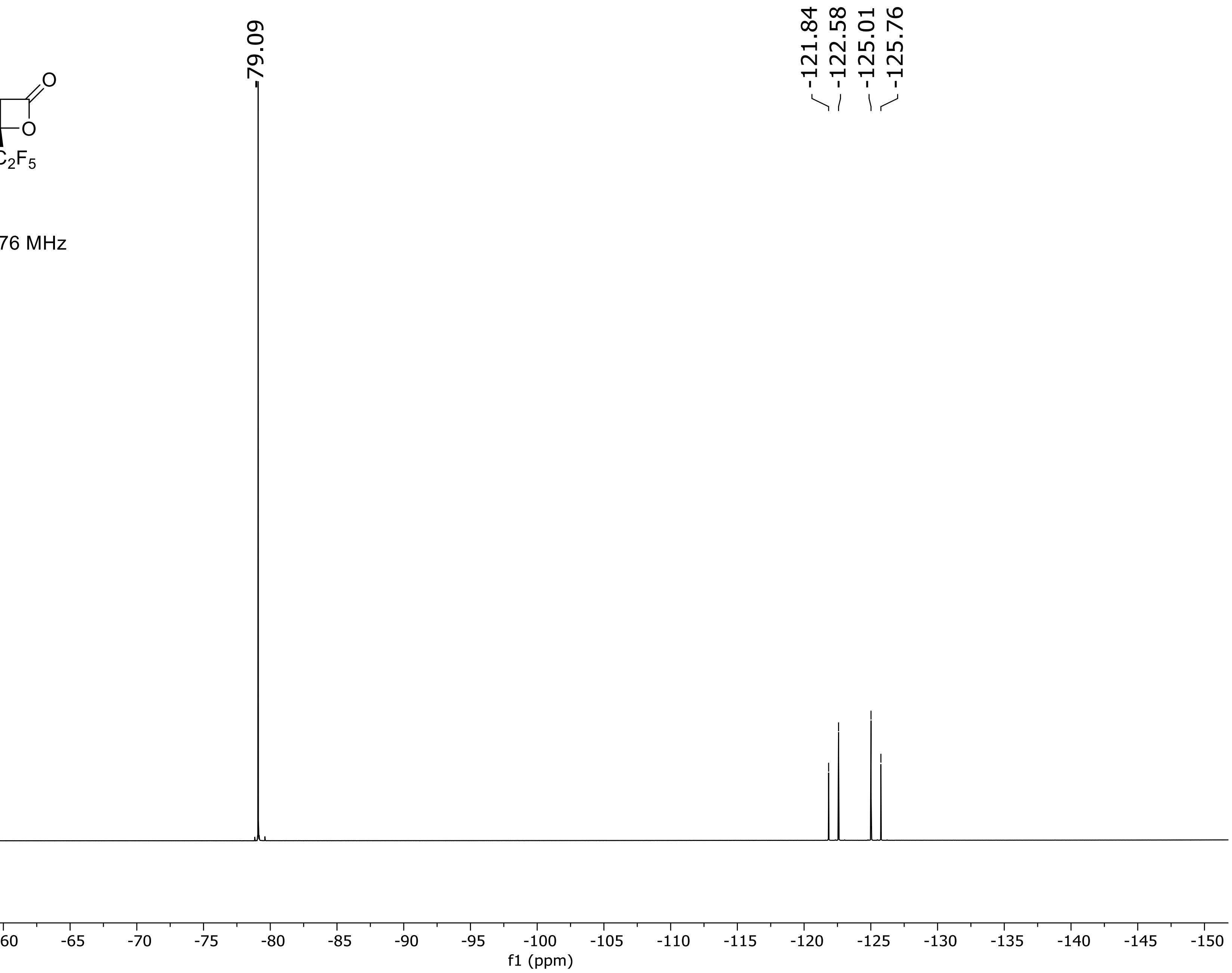

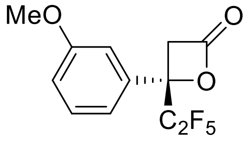

**20**

$^{13}C$, $CDCl_3$, 126 MHz

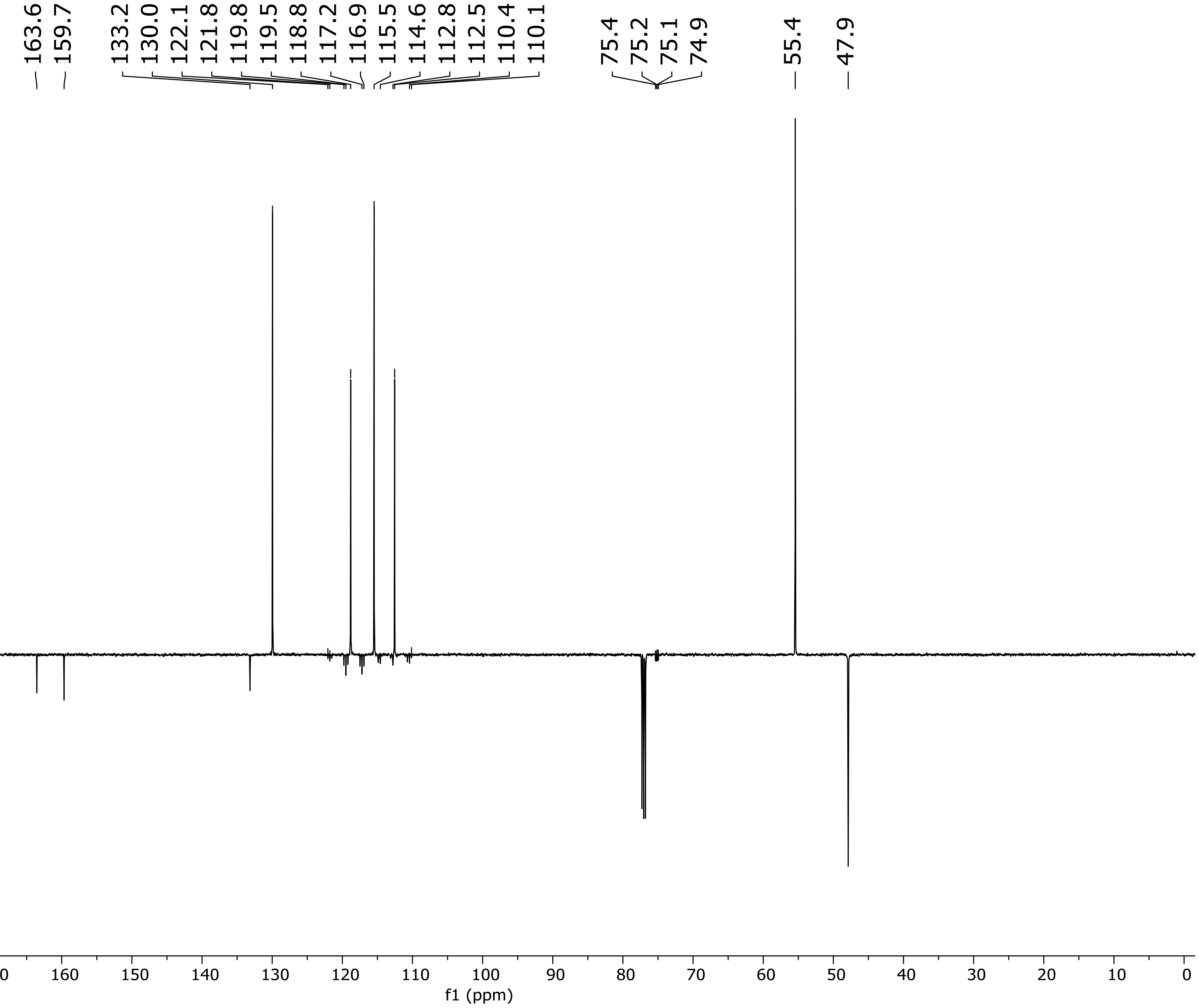

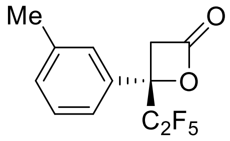


**21**

$^{1}H$, $CDCl_3$, 500 MHz

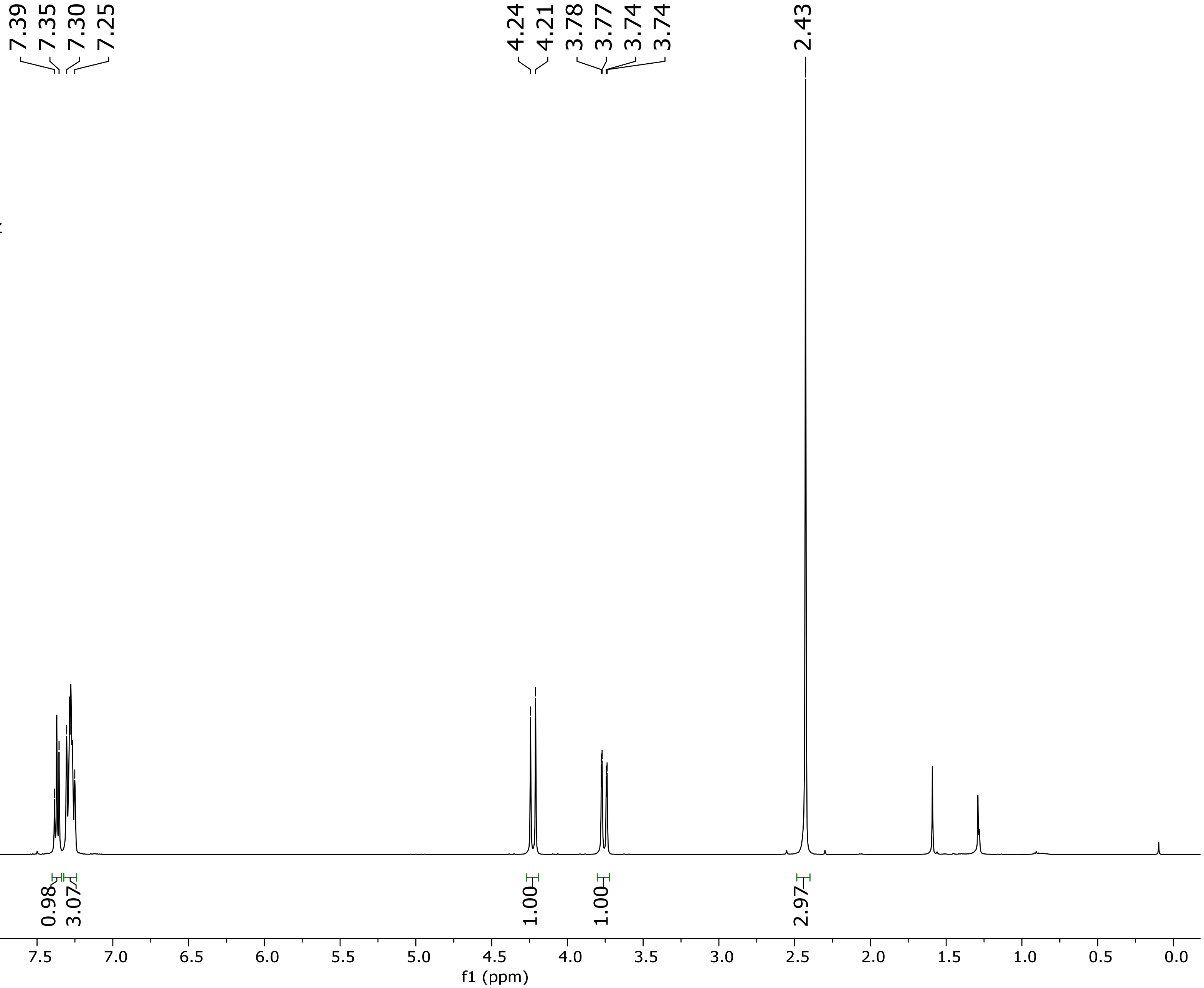

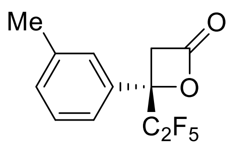

**21**

$^{19}F$, $CDCl_3$, 376 MHz

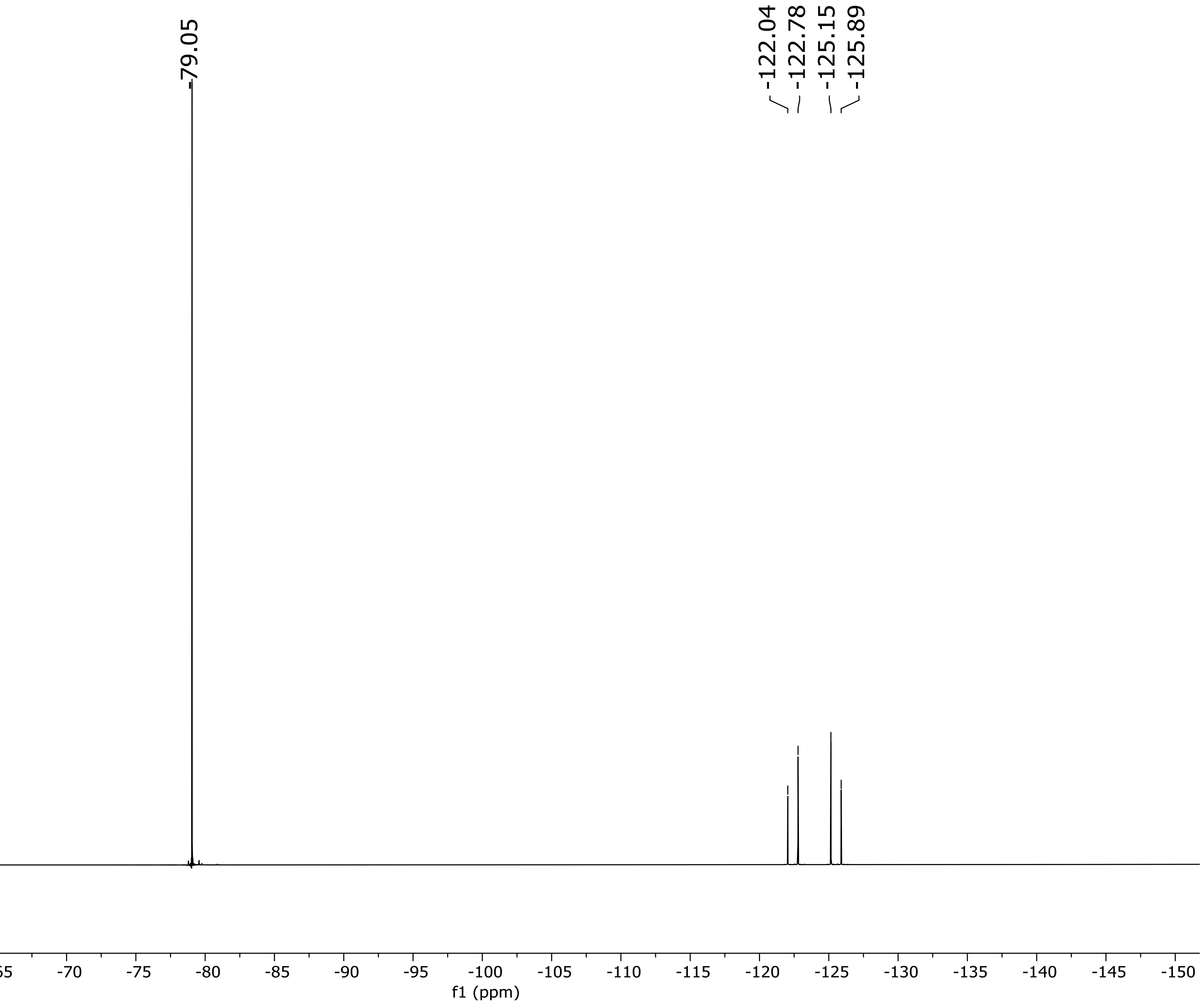

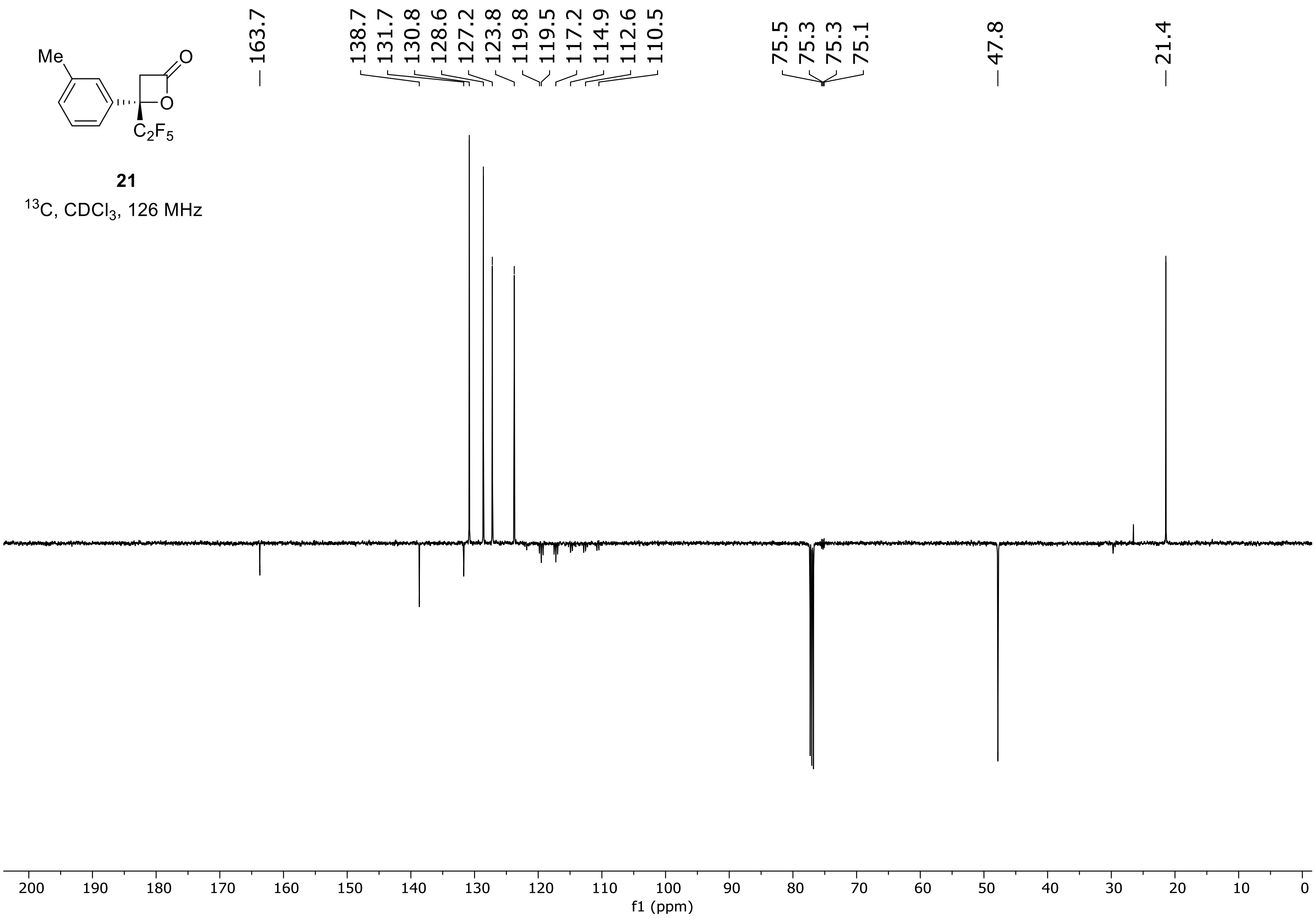

Me
O
O
$C_2F_5$
21
$^{13}$C, $CDCl_3$, 126 MHz
163.7
138.7
131.7
130.8
128.6
127.2
123.8
119.8
119.5
117.2
114.9
112.6
110.5
75.5
75.3
75.3
75.1
47.8
21.4
200 190 180 170 160 150 140 130 120 110 100 90 80 70 60 50 40 30 20 10 0
f1 (ppm)

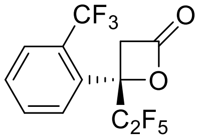


**22**

$^1H$, $CDCl_3$, 500 MHz

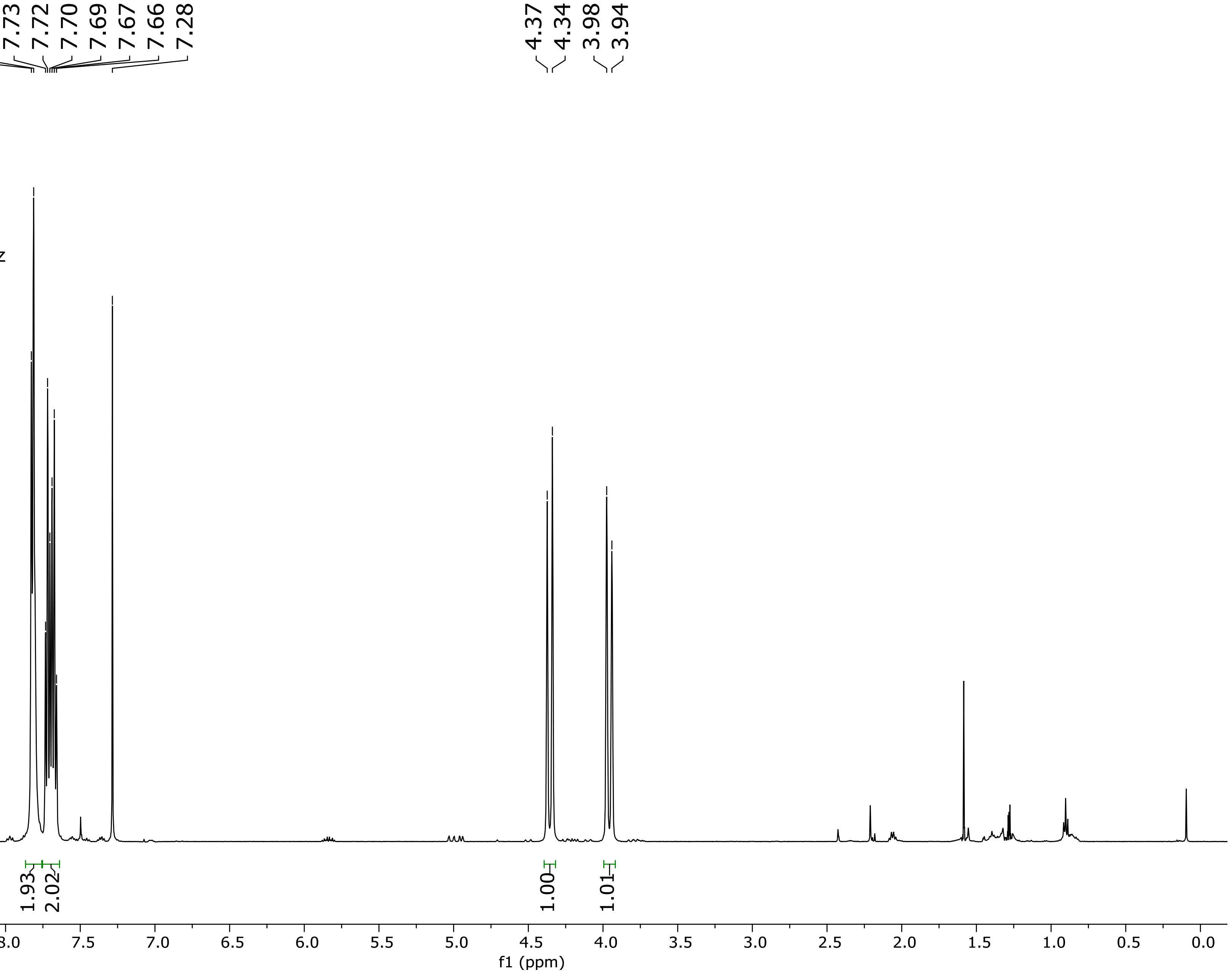

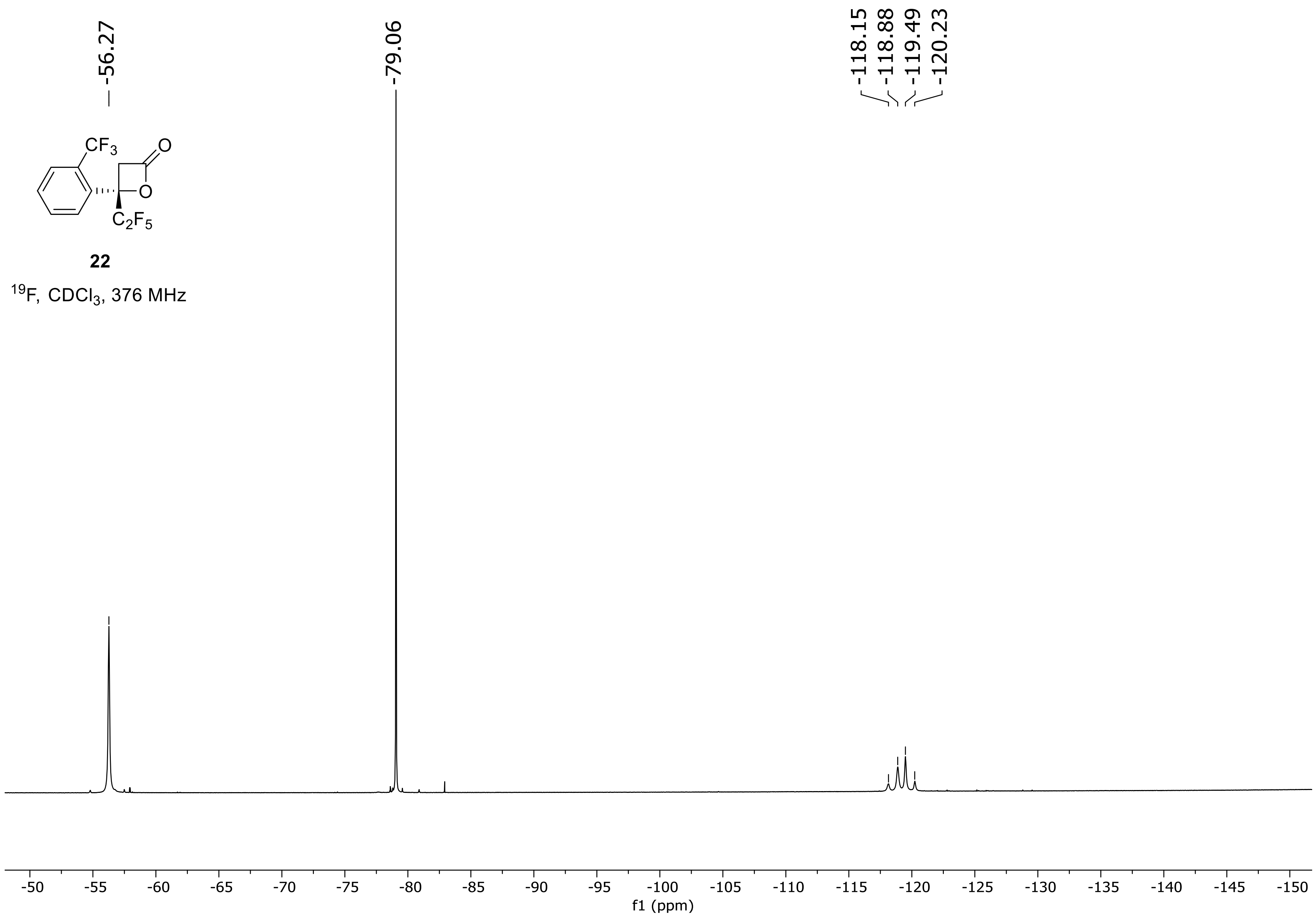
-56.27
-79.06
-118.15
-118.88
-119.49
-120.23
CF3
O
O
C2F5
22
19F, CDCl3, 376 MHz
-50
-55
-60
-65
-70
-75
-80
-85
-90
-95
-100
-105
-110
-115
-120
-125
-130
-135
-140
-145
-150
f1 (ppm)

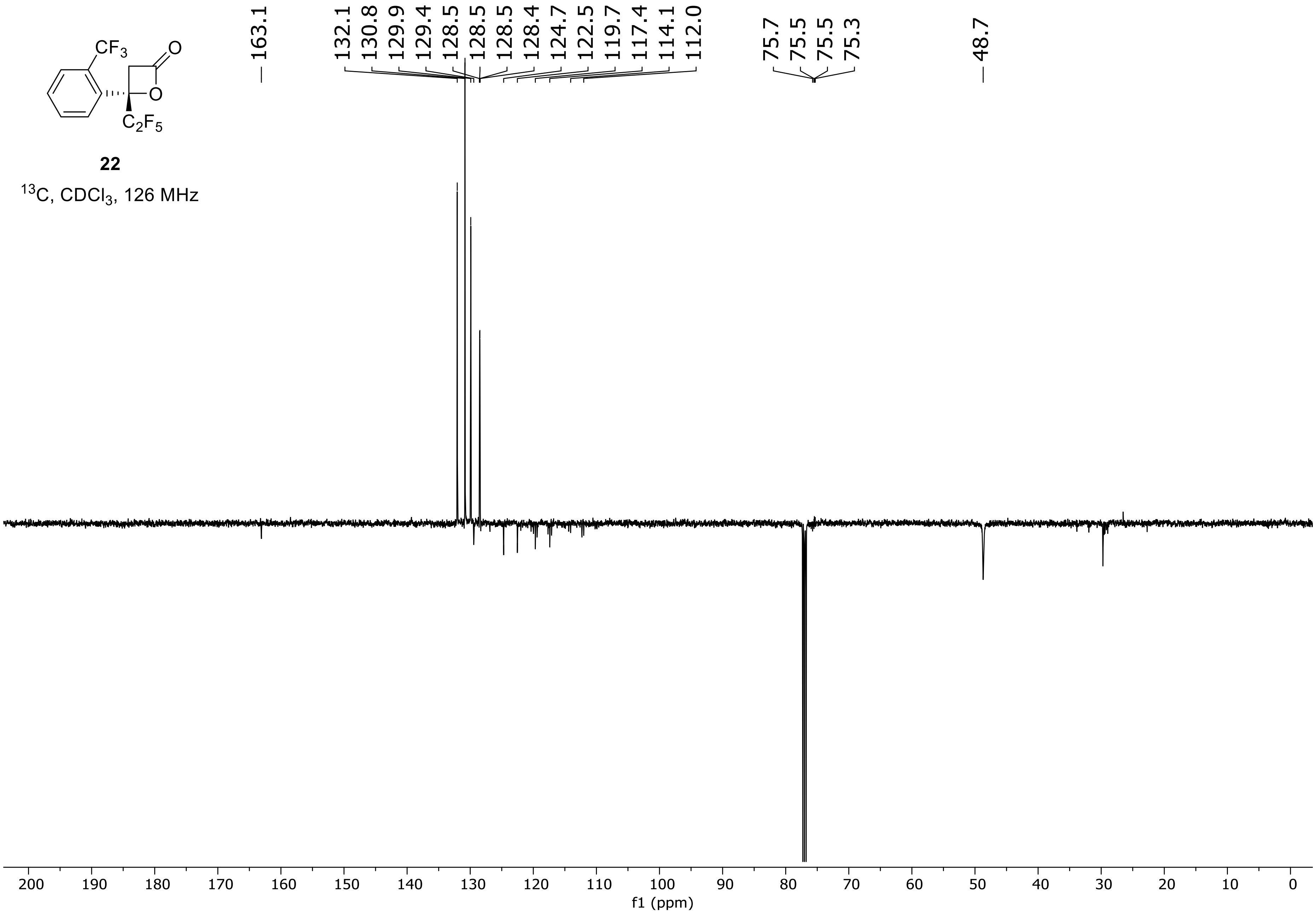

CF3
O
O
C2F5
22
13C, CDCl3, 126 MHz
163.1
132.1
130.8
129.9
129.4
128.5
128.5
128.5
128.4
124.7
122.5
119.7
117.4
114.1
112.0
75.7
75.5
75.5
75.3
48.7
200 190 180 170 160 150 140 130 120 110 100 90 80 70 60 50 40 30 20 10 0
f1 (ppm)

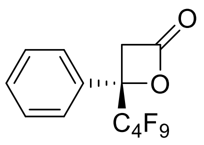


**23**

$^1$H, $CDCl_3$, 500 MHz

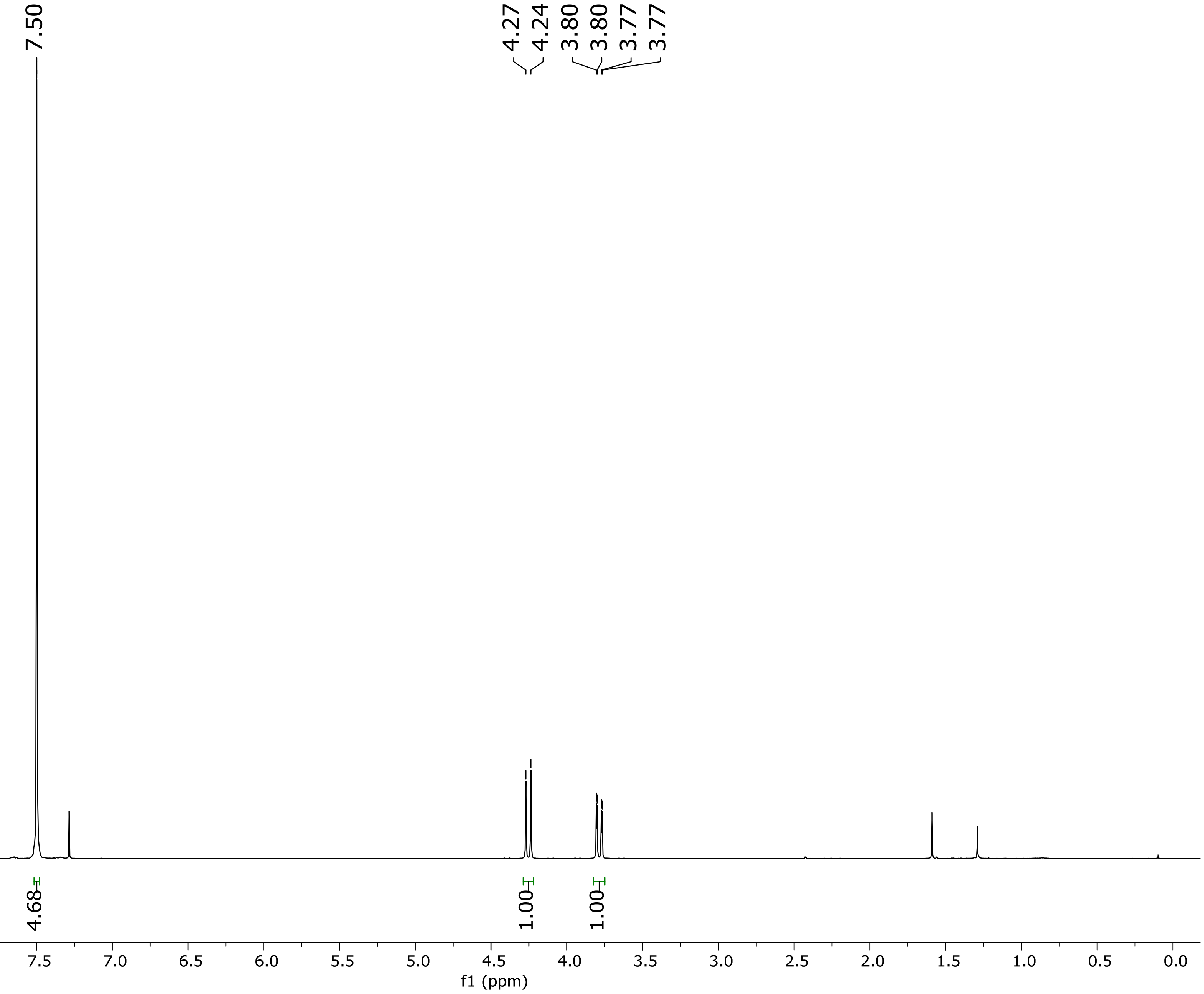

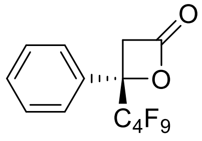


**23**

$^{19}F$, $CDCl_3$, 376 MHz

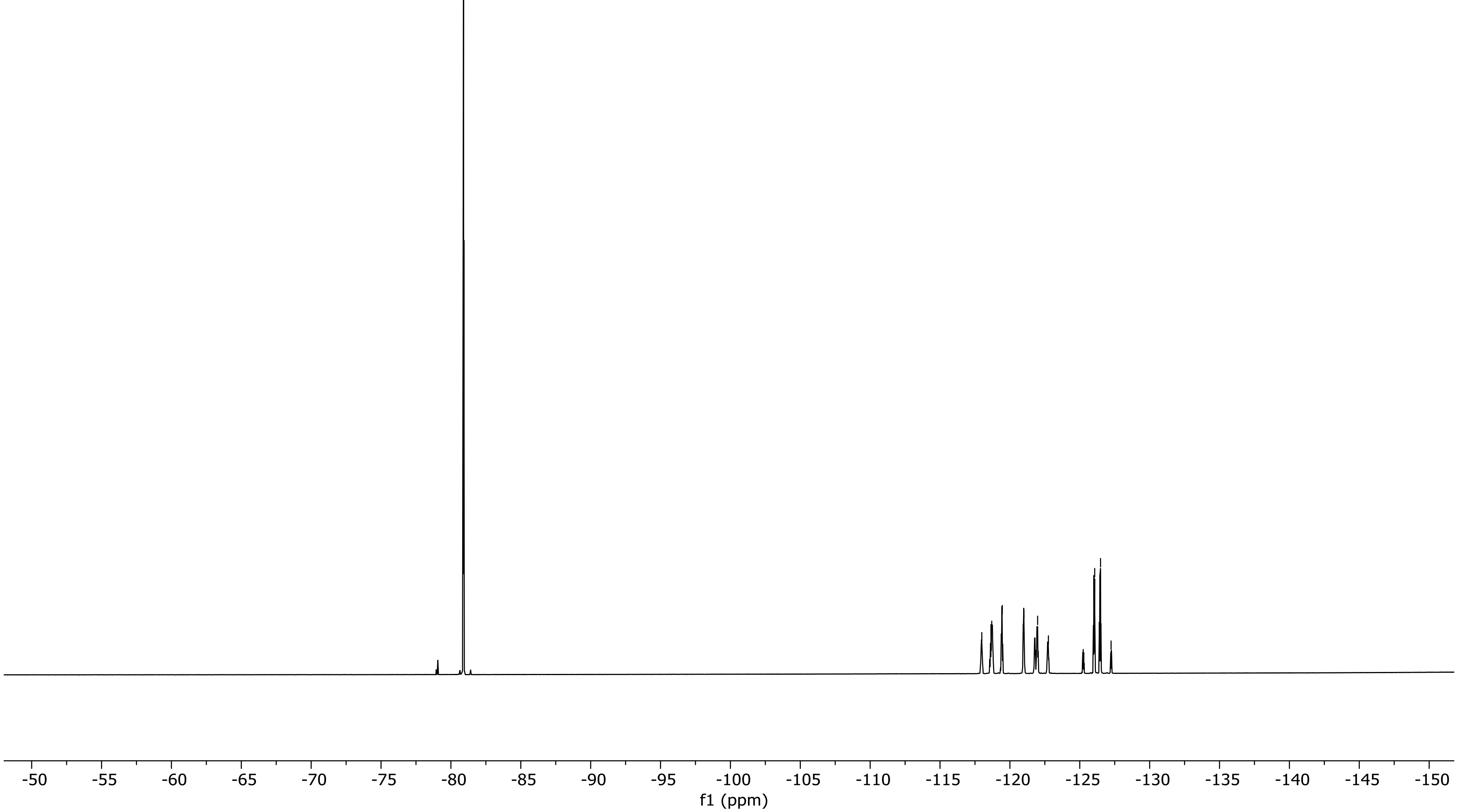

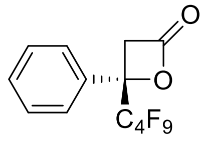

**23**
$^{13}C$, $CDCl_3$, 126 MHz

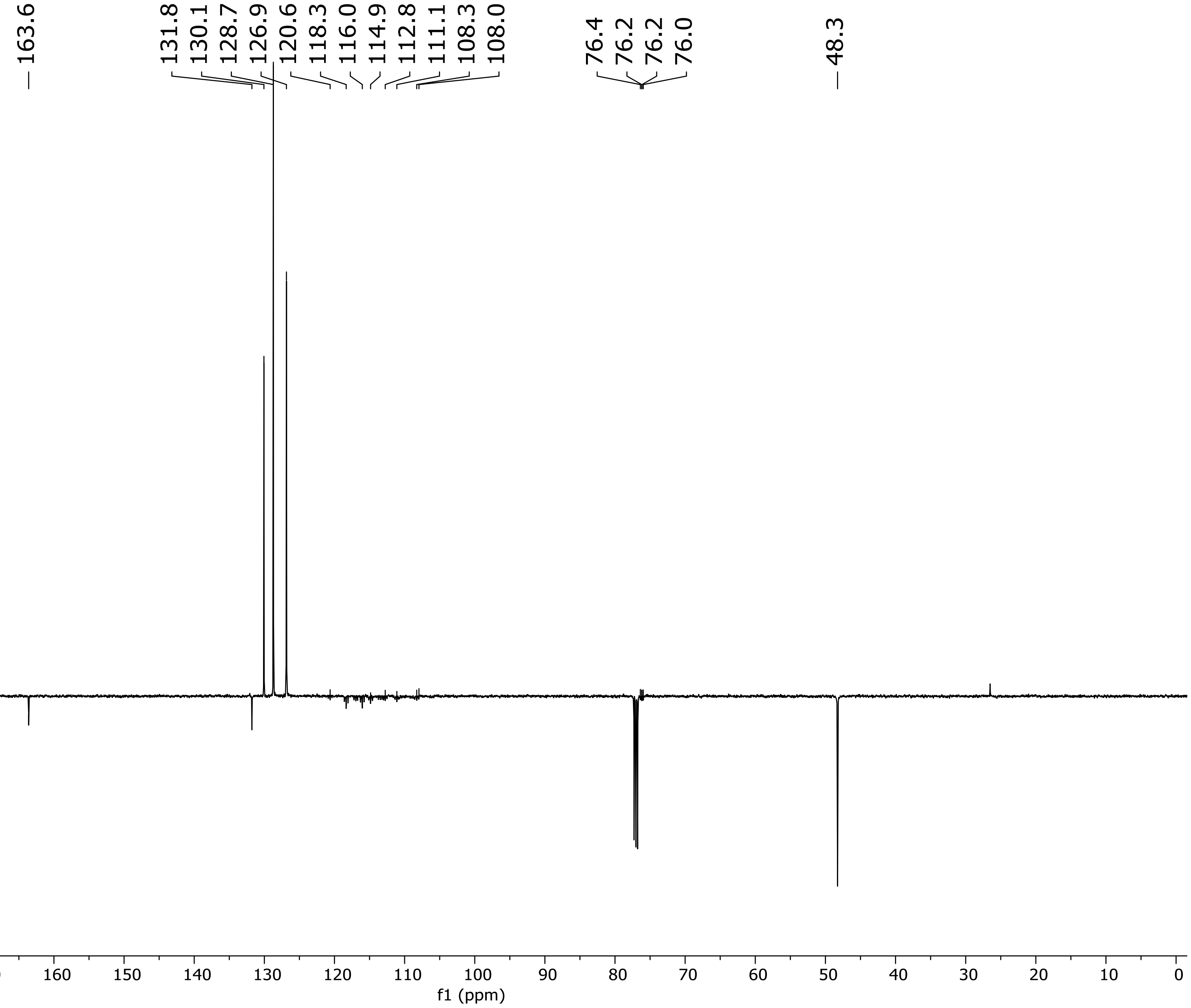

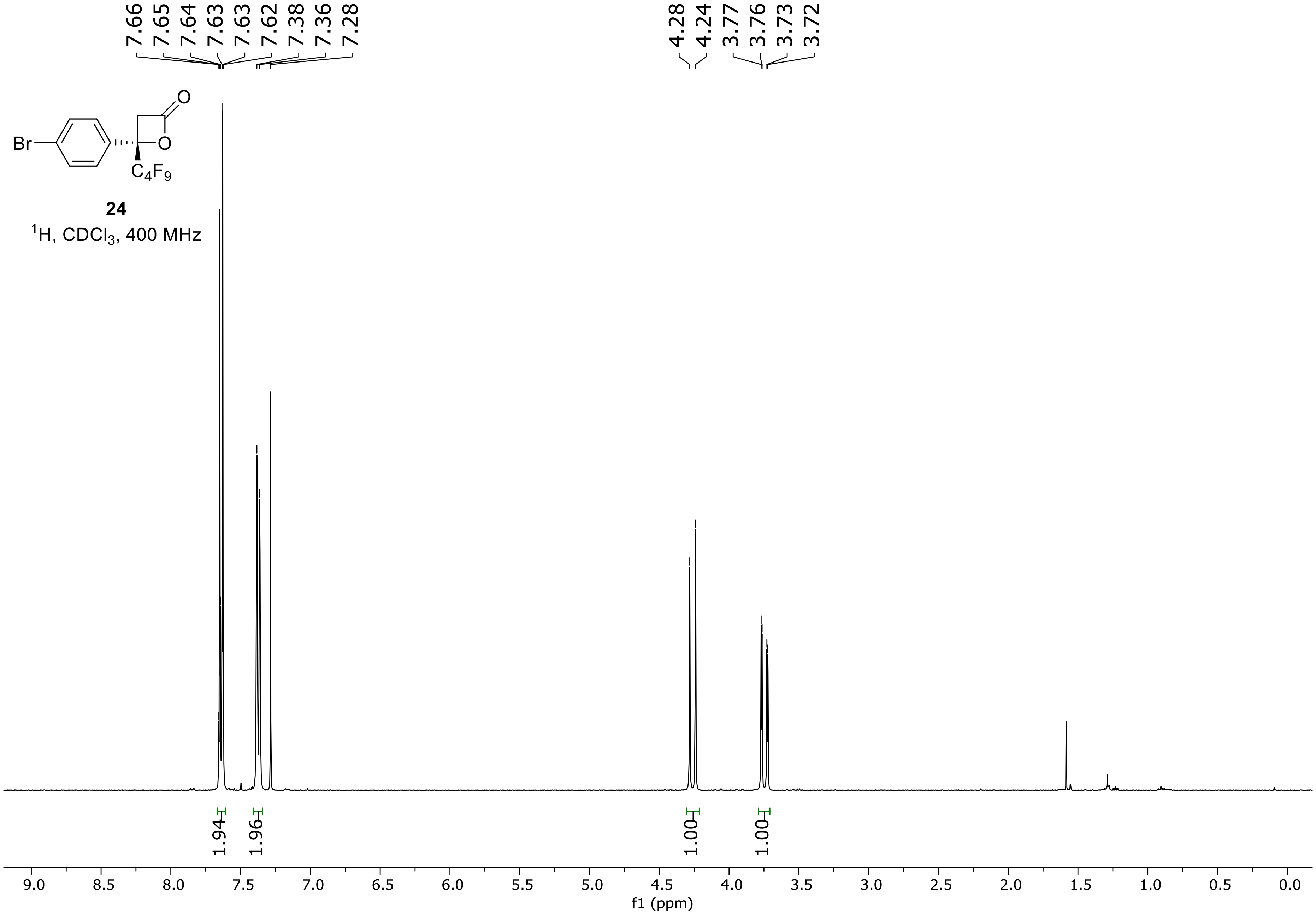


**24**

$^1H$, $CDCl_3$, 400 MHz

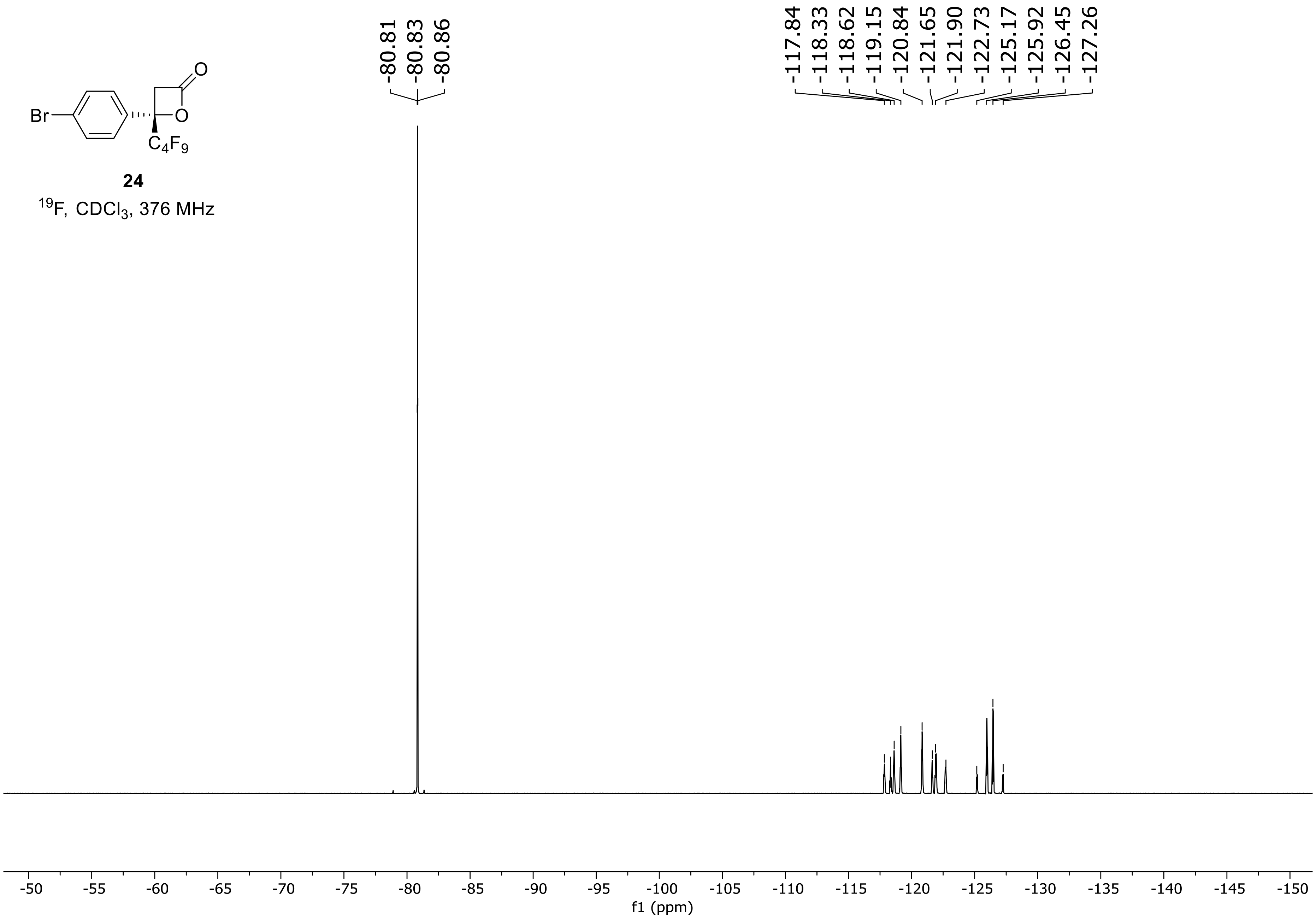
Br
O
O
C4F9
24
19F, CDCl3, 376 MHz
-80.81
-80.83
-80.86
-117.84
-118.33
-118.62
-119.15
-120.84
-121.65
-121.90
-122.73
-125.17
-125.92
-126.45
-127.26
-50 -55 -60 -65 -70 -75 -80 -85 -90 -95 -100 -105 -110 -115 -120 -125 -130 -135 -140 -145 -150
f1 (ppm)

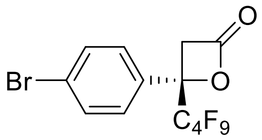


**24**

$^{13}C$, $CDCl_3$, 126 MHz

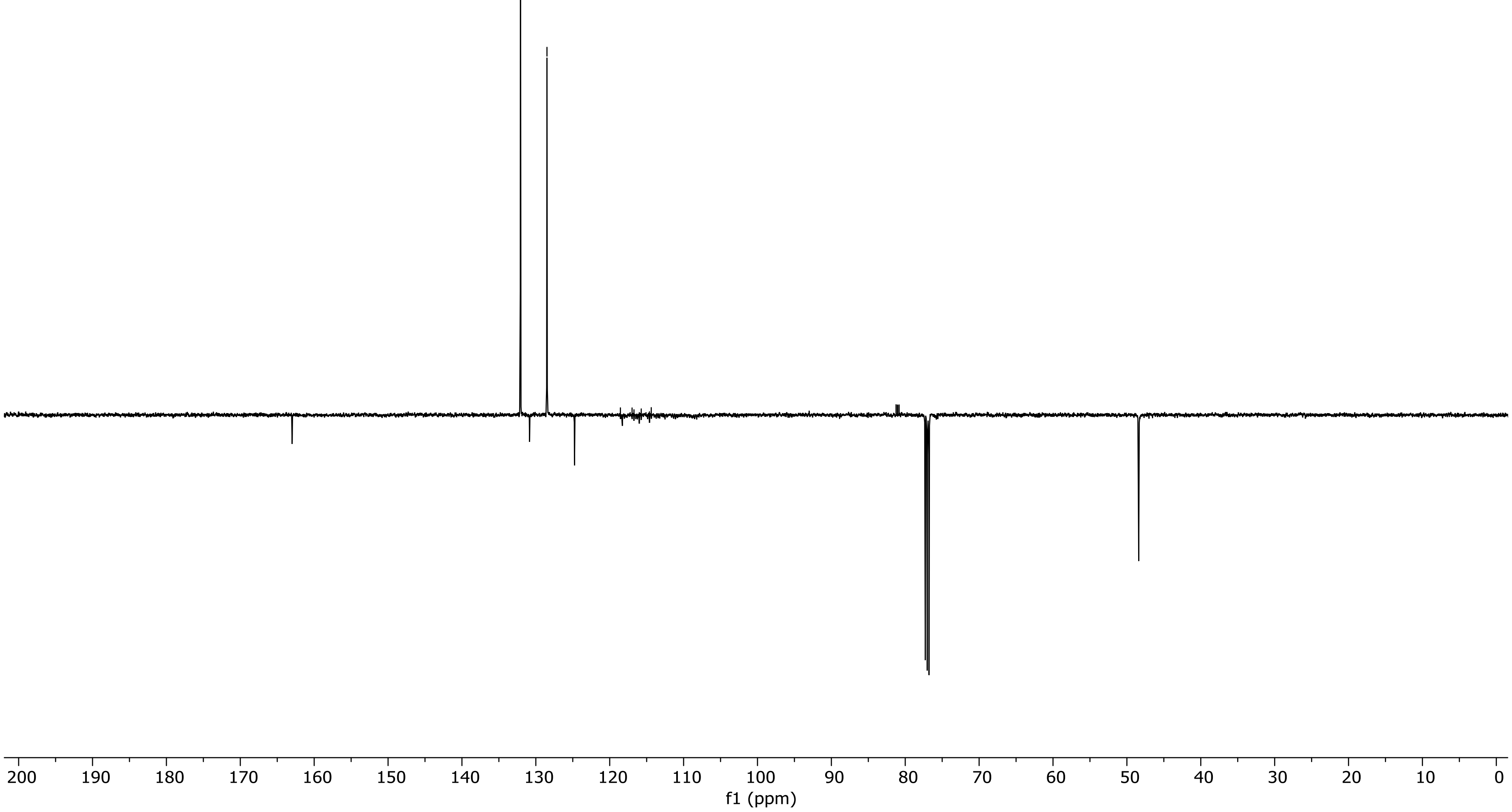

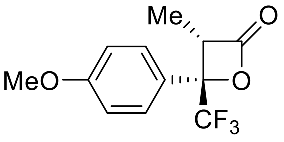


**25**

$^{1}H$, $CDCl_3$, 500 MHz

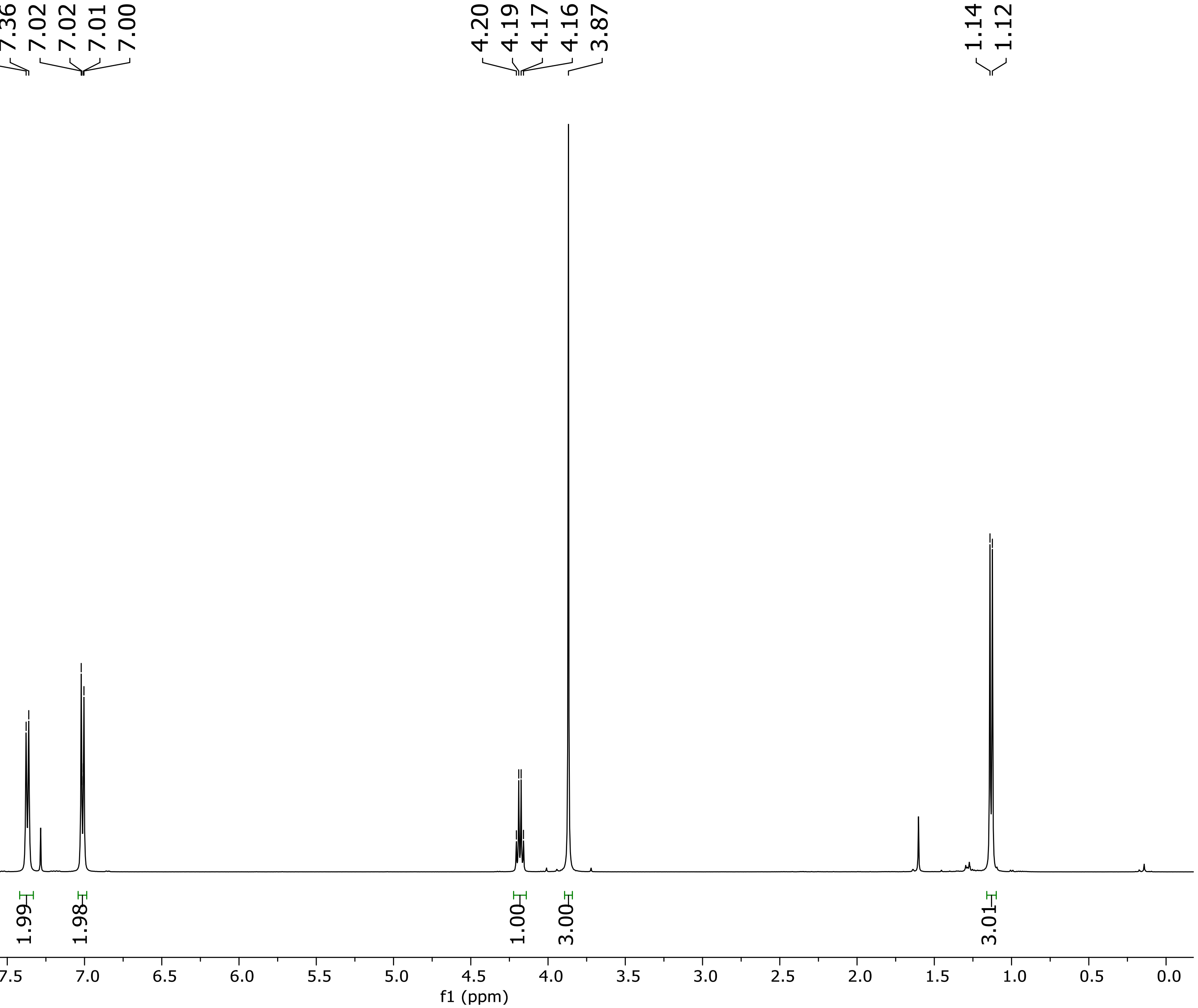

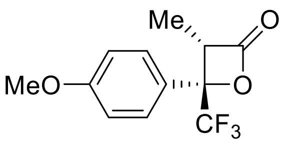


**25**

$^{19}F$, $CDCl_3$, 471 MHz

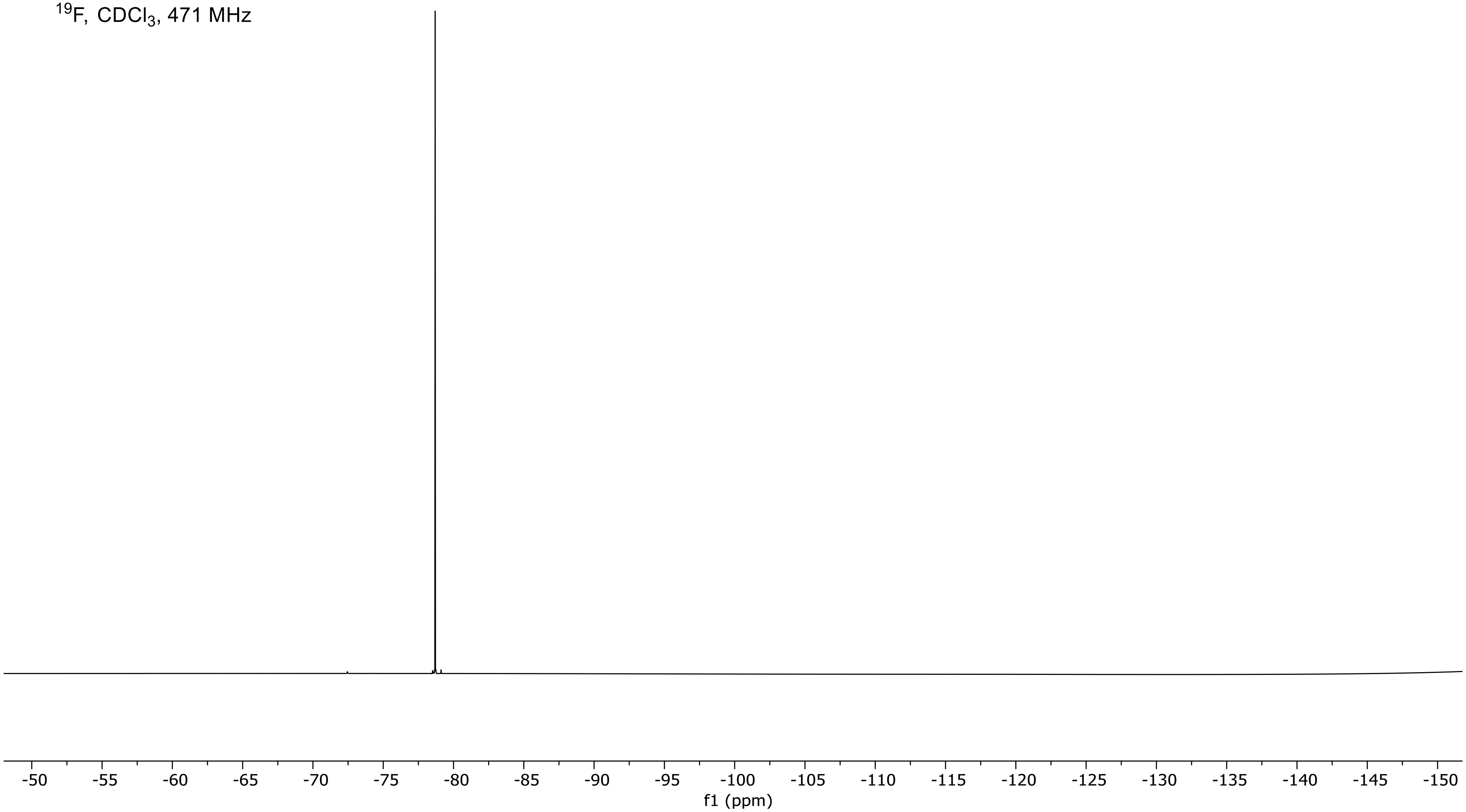

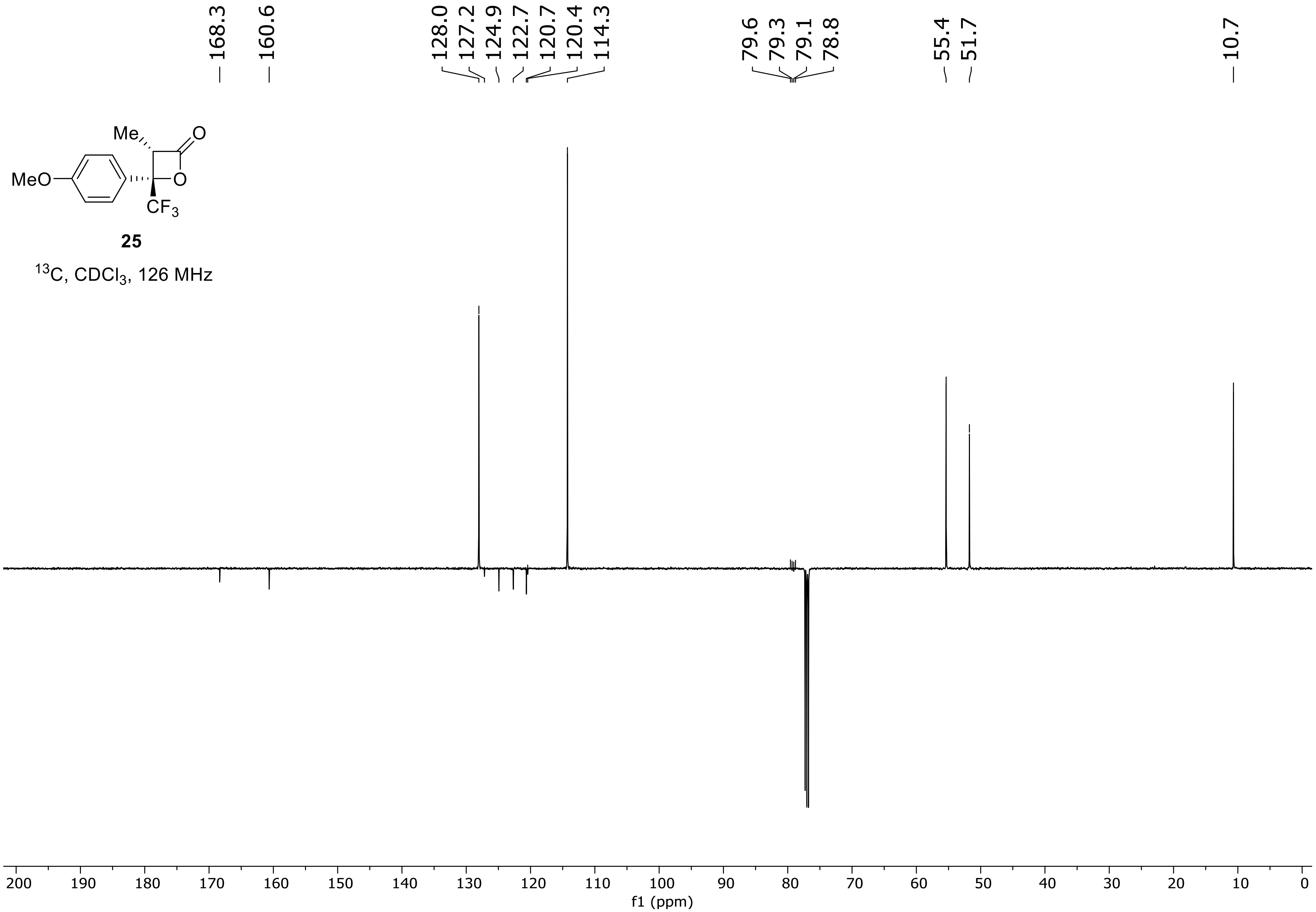

**25**

$^{13}C$, $CDCl_3$, 126 MHz

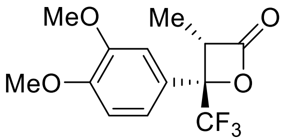


**26**

$^{1}H$, $CDCl_3$, 500 MHz

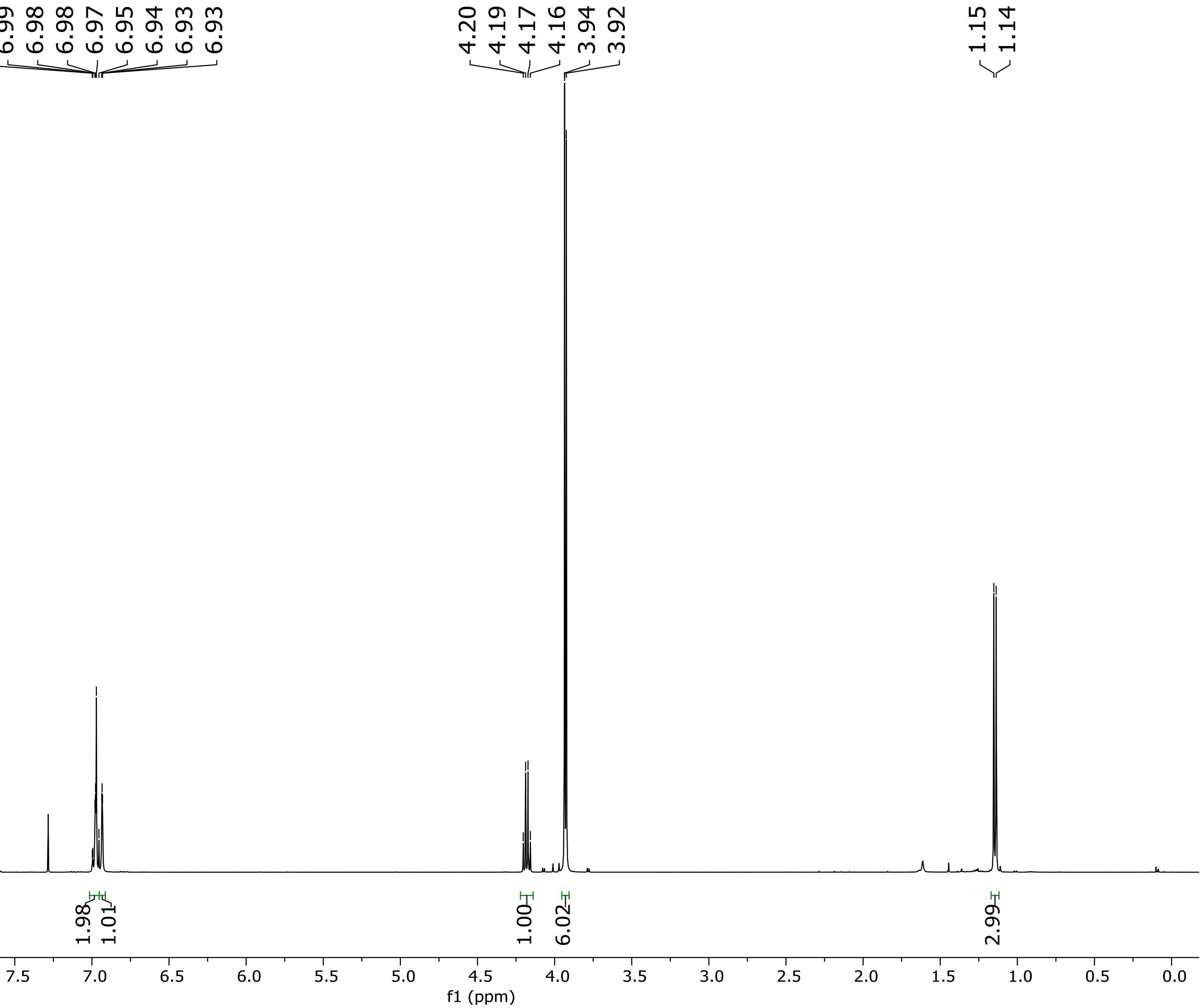

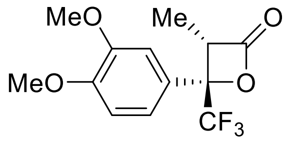

**26**

$^{19}F$, $CDCl_3$, 471 MHz

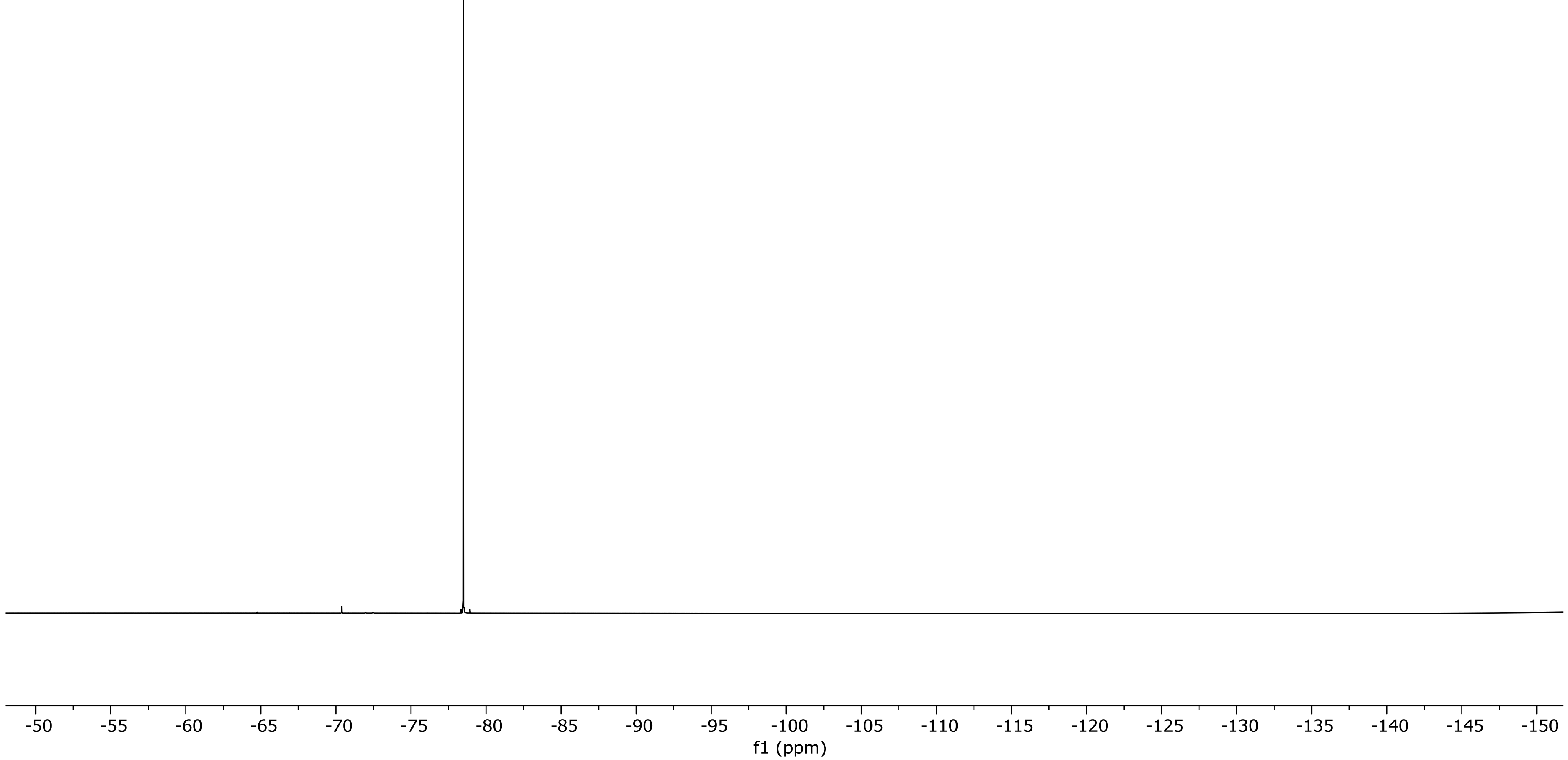

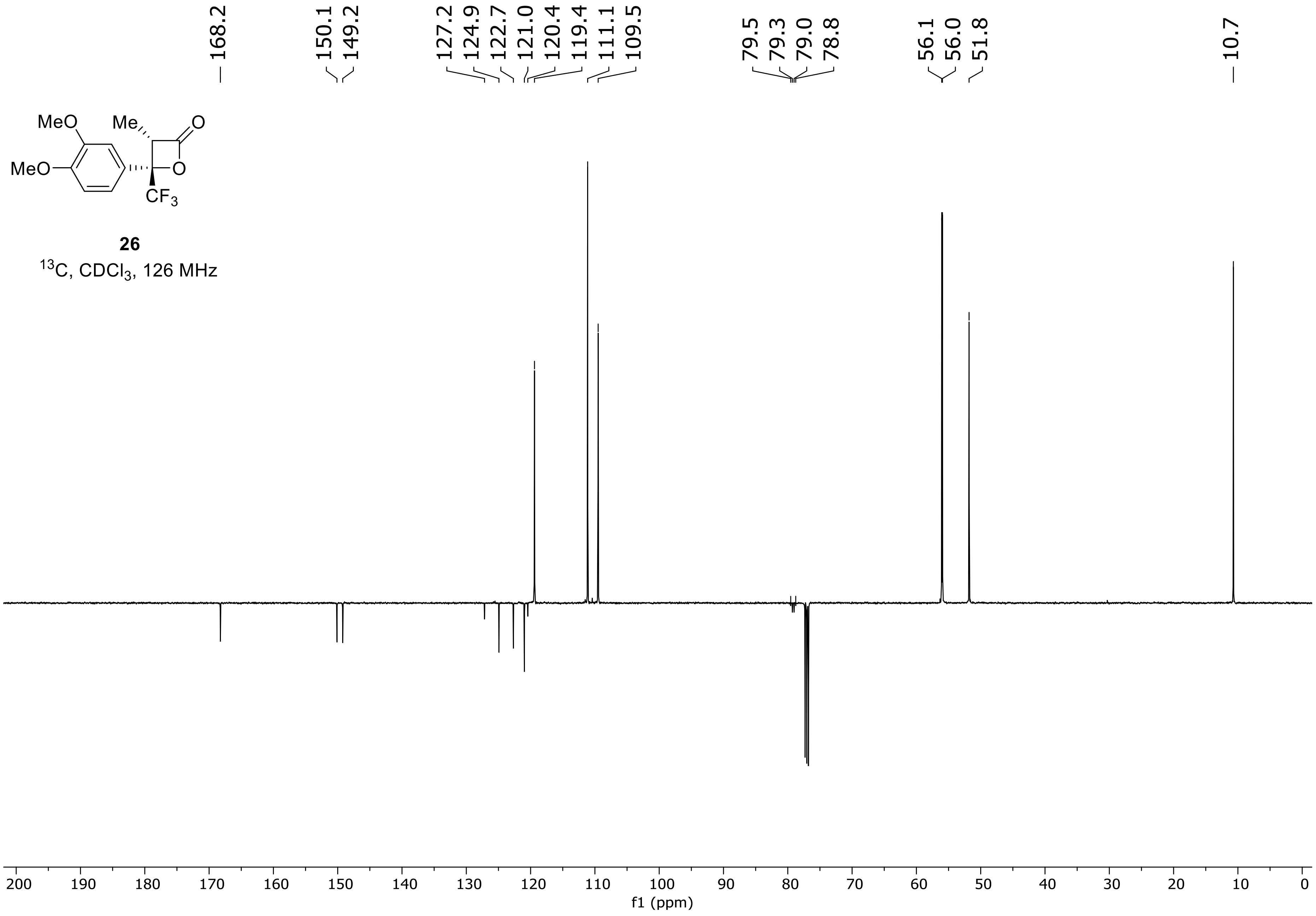


**26**

$^{13}C$, $CDCl_3$, 126 MHz

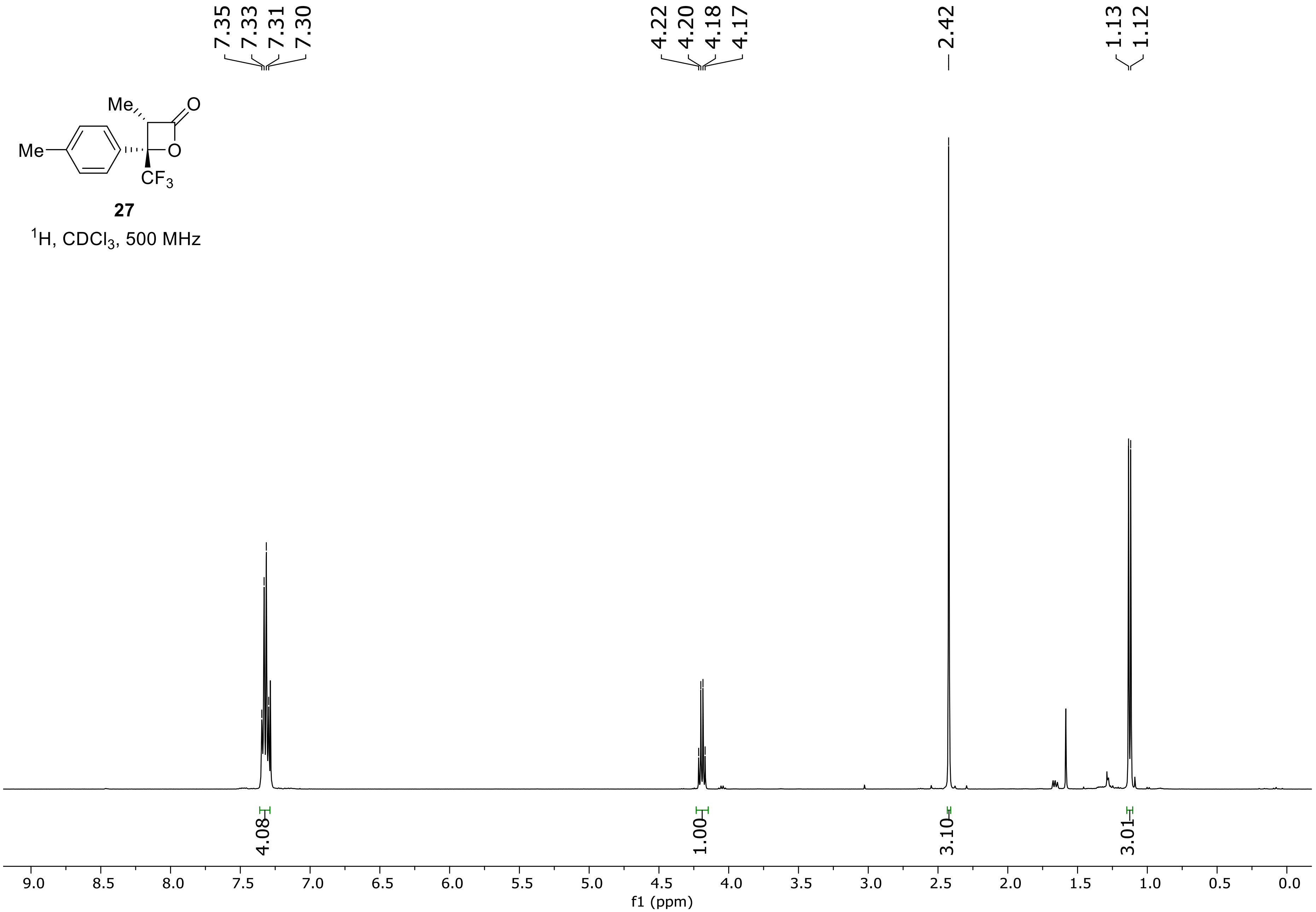


**27**

$^1H$, $CDCl_3$, 500 MHz

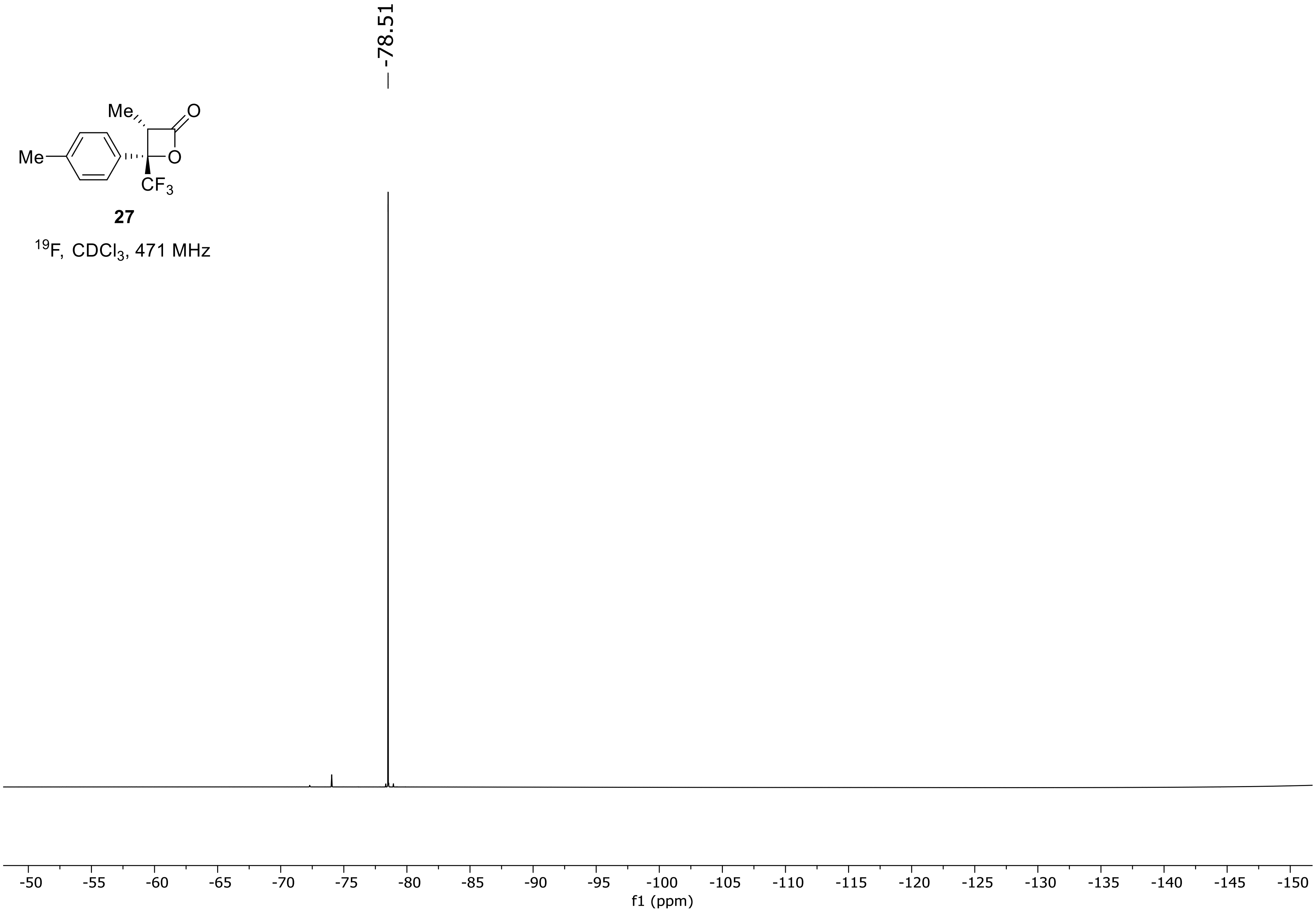


**27**

$^{19}F$, $CDCl_3$, 471 MHz

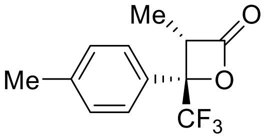

**27**

$^{13}C$, $CDCl_3$, 126 MHz

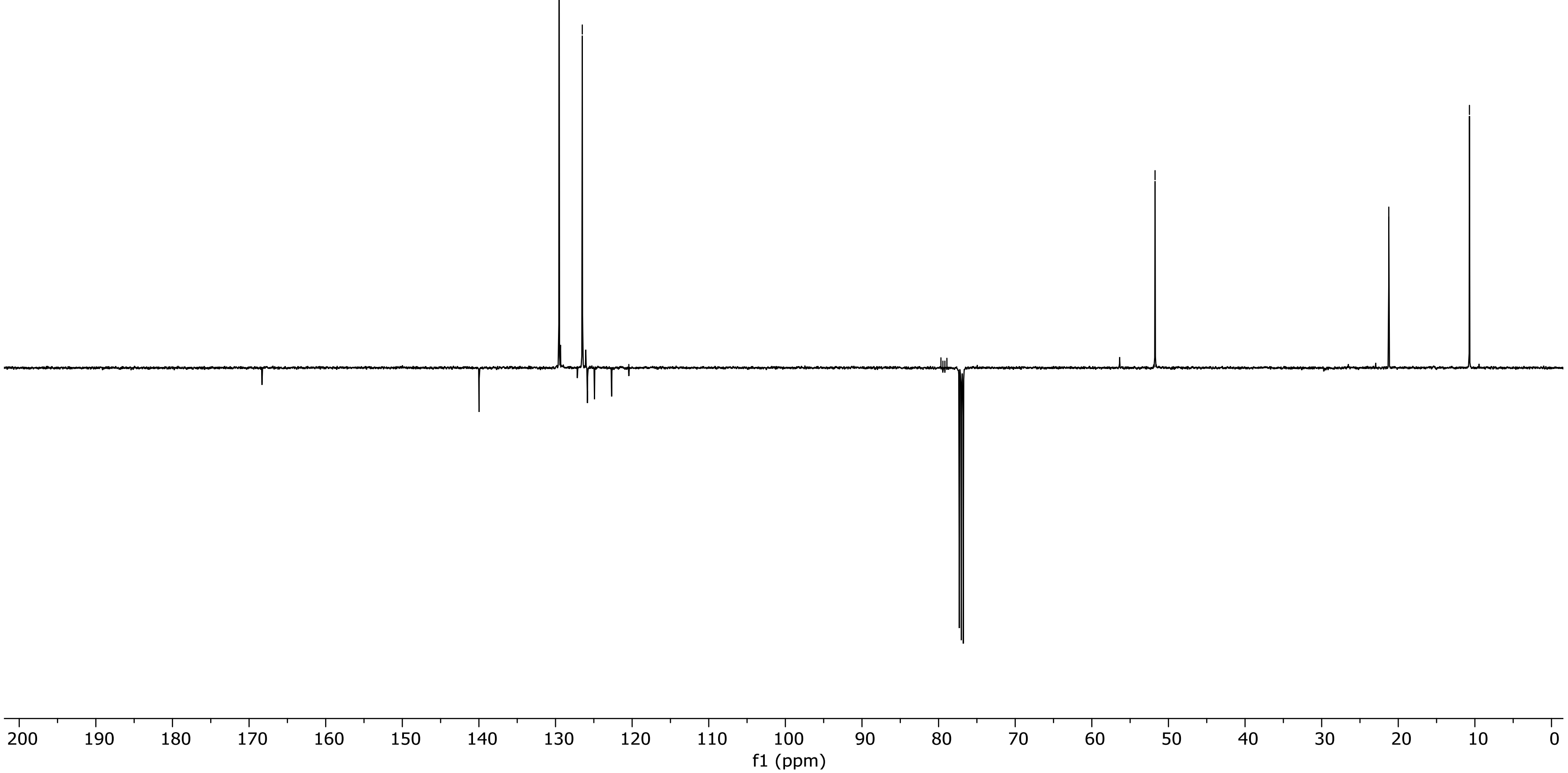

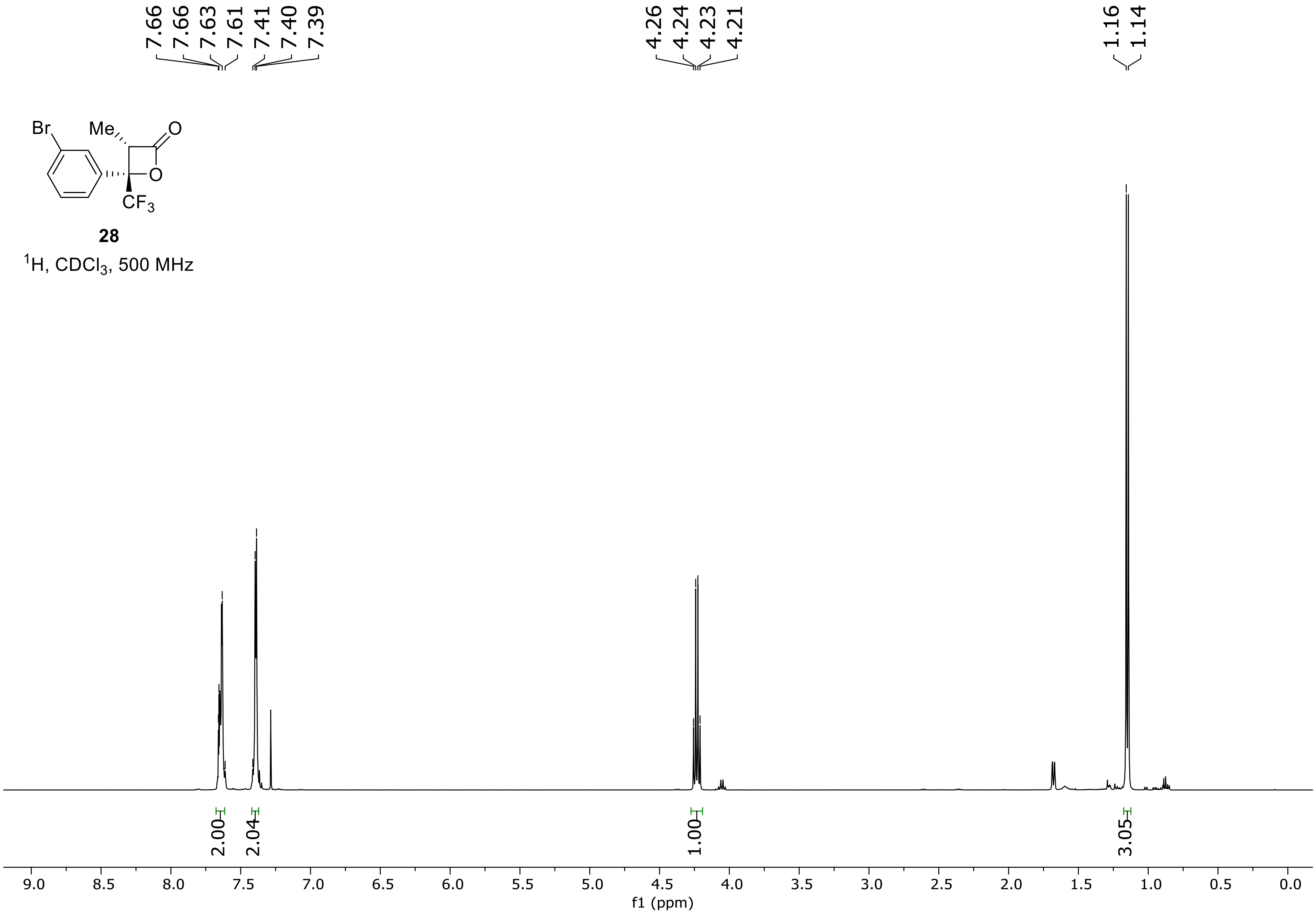
7.66 7.66 7.63 7.61 7.41 7.40 7.39
4.26 4.24 4.23 4.21
1.16 1.14
Br
Me
O
O
CF3
28
1H, CDCl3, 500 MHz
2.00
2.04
1.00
3.05
9.0 8.5 8.0 7.5 7.0 6.5 6.0 5.5 5.0 4.5 4.0 3.5 3.0 2.5 2.0 1.5 1.0 0.5 0.0
f1 (ppm)

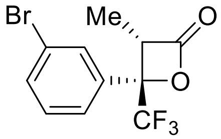


**28**

$^{19}F$, $CDCl_3$, 376 MHz

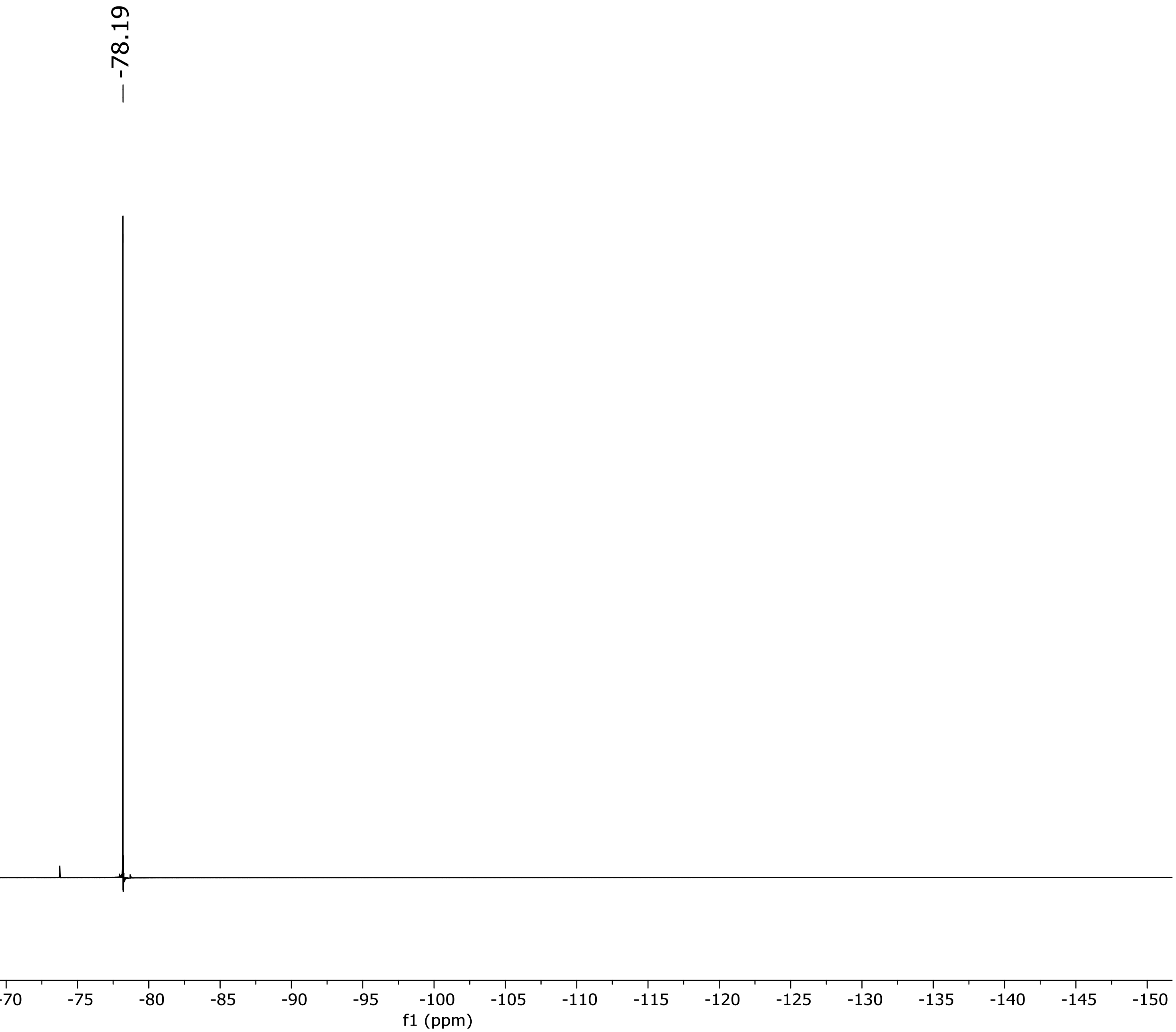

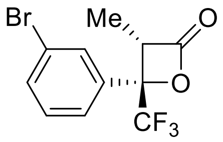

**28**

$^{13}C$, $CDCl_3$, 126 MHz

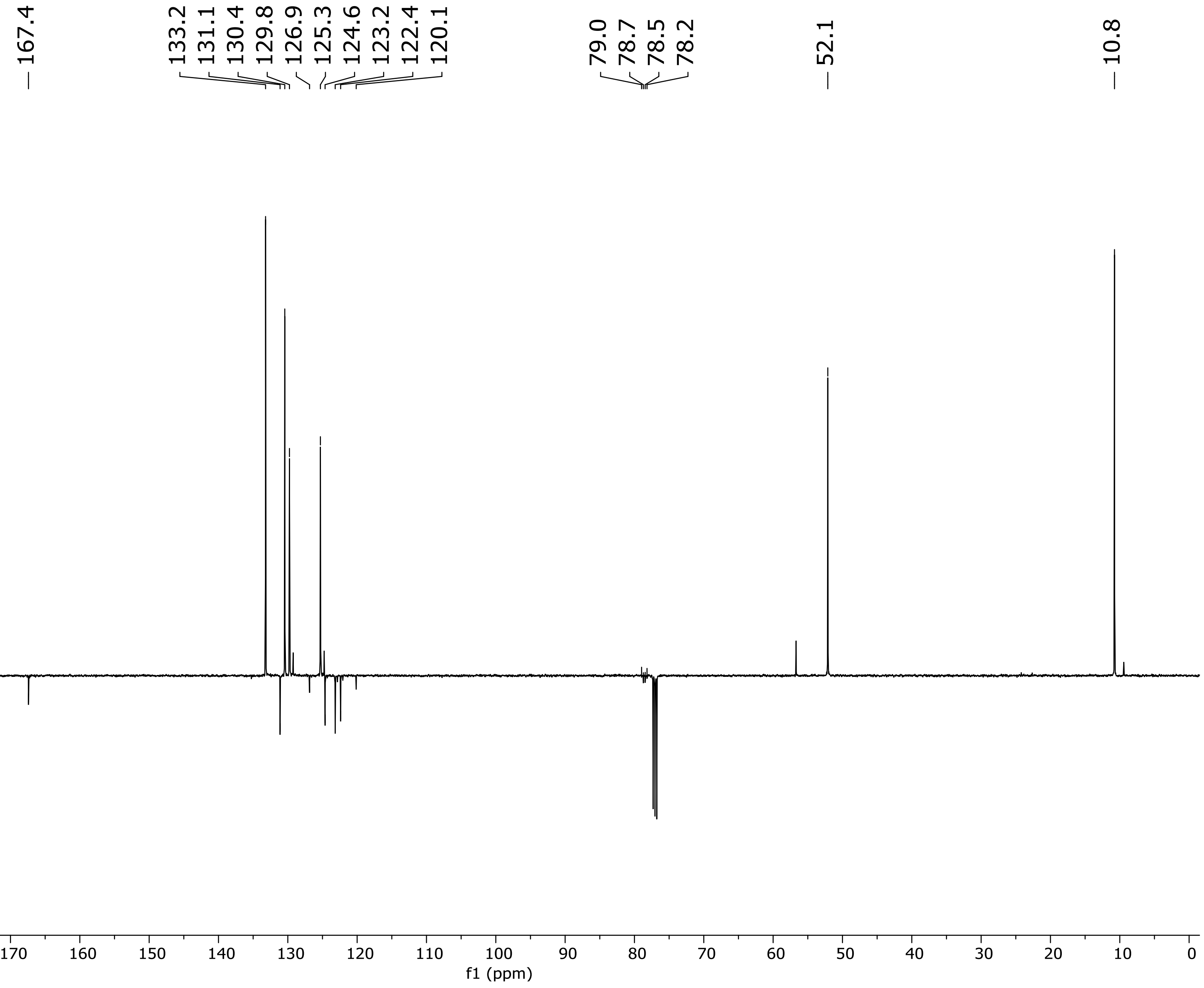

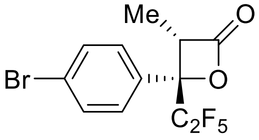

**29**

$^{1}H$, $CDCl_3$, 400 MHz

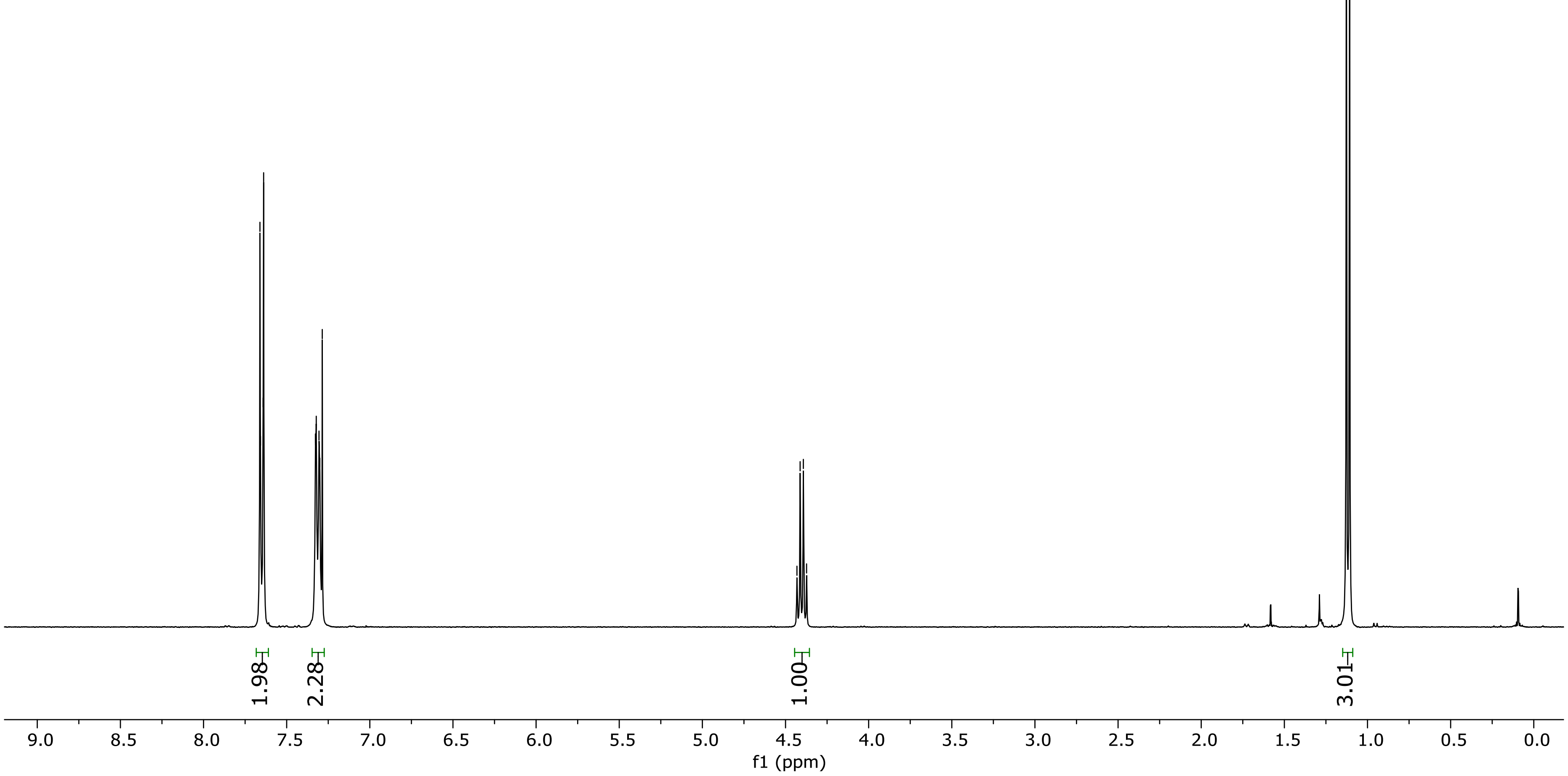

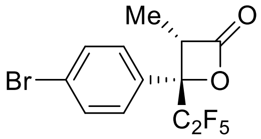

**29**

$^{19}$F, $CDCl_3$, 376 MHz

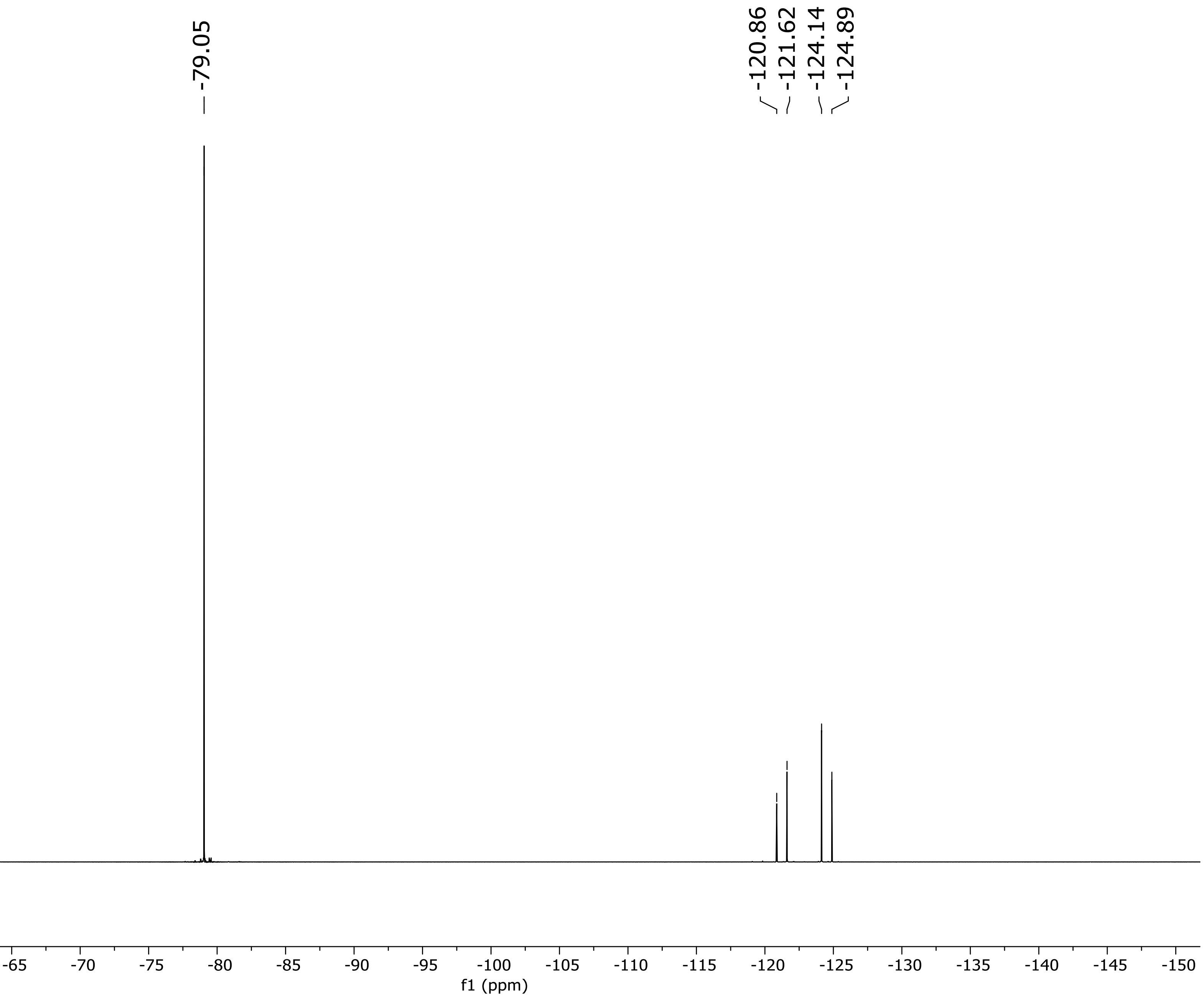

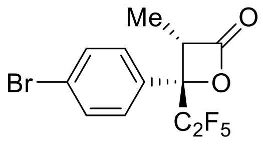

**29**

$^{13}C$, $CDCl_3$, 126 MHz

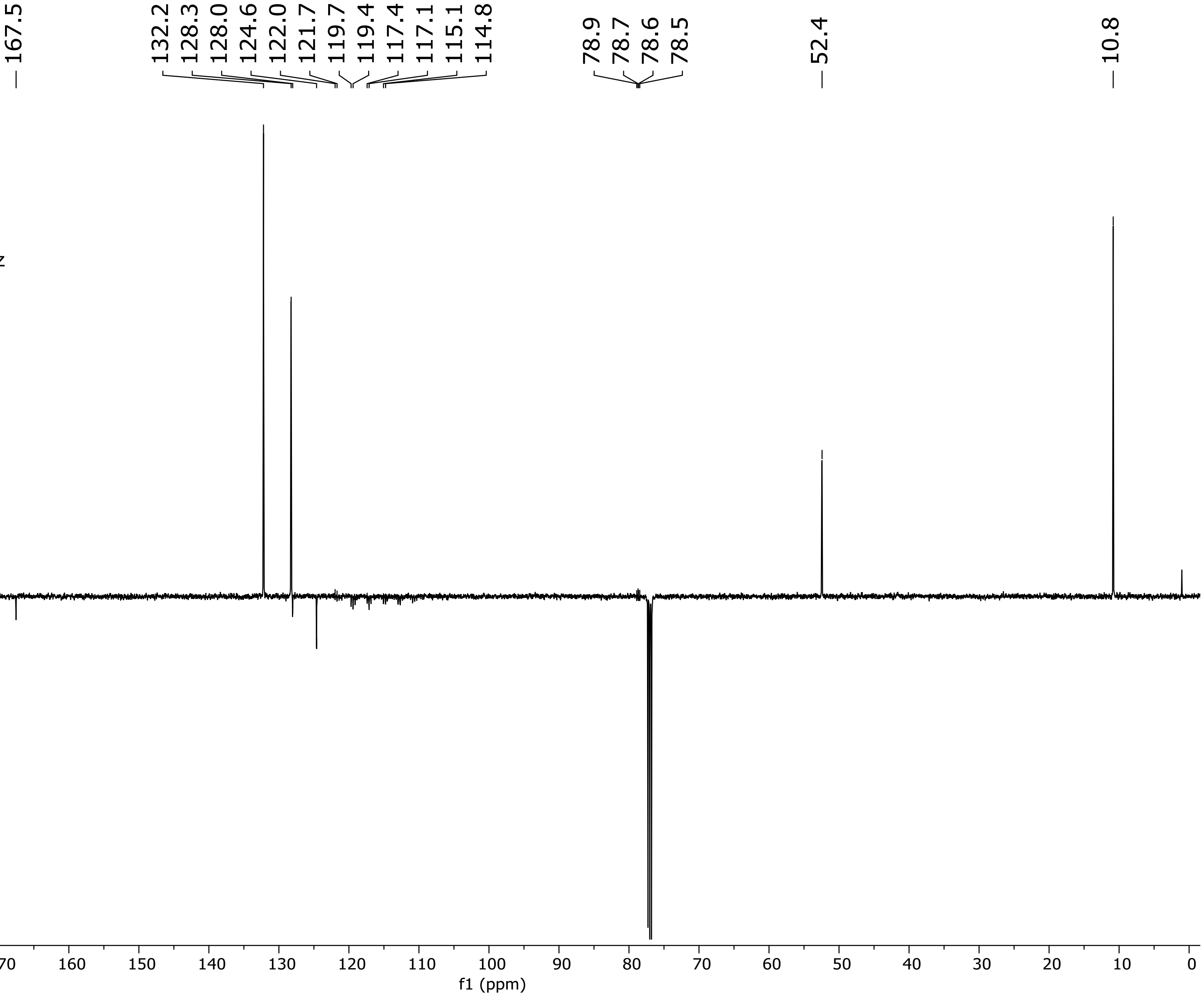

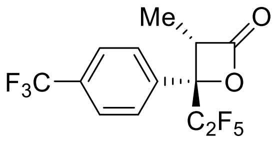

**30**

$^1$H, $CDCl_3$, 400 MHz

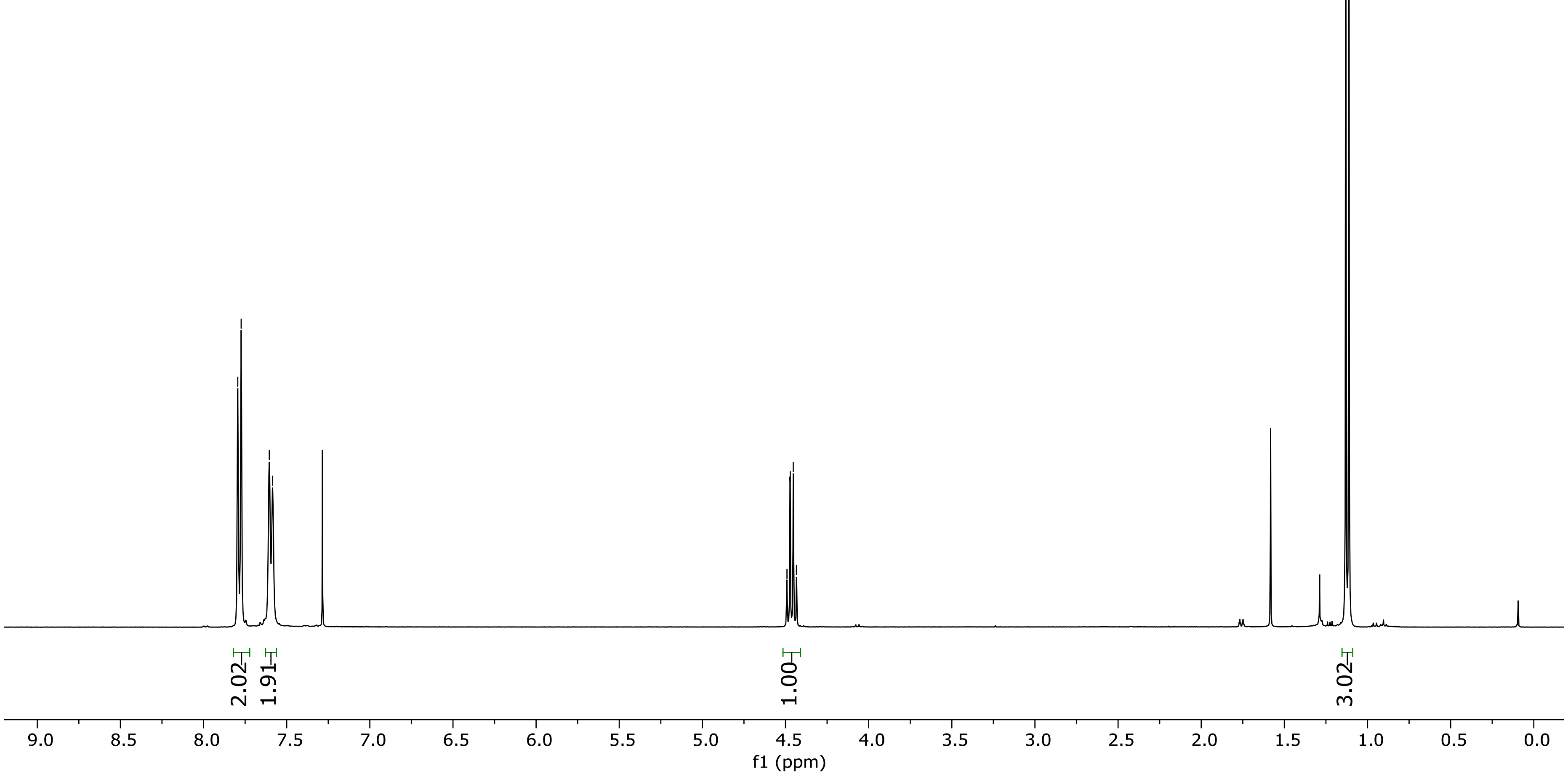

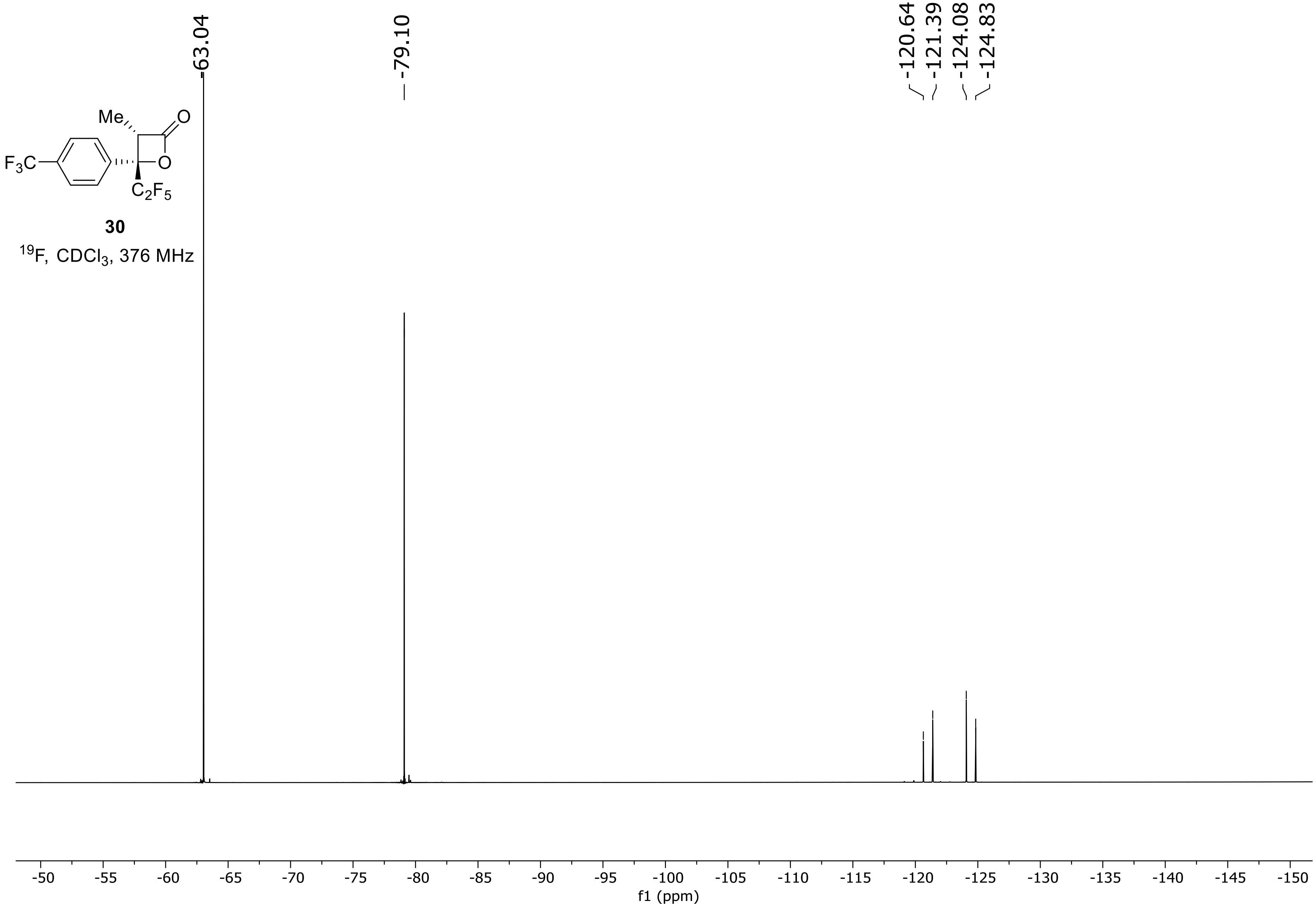


**30**

$^{19}$F, $CDCl_3$, 376 MHz

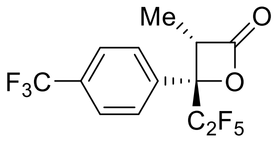


**30**

$^{13}C$, $CDCl_3$, 126 MHz

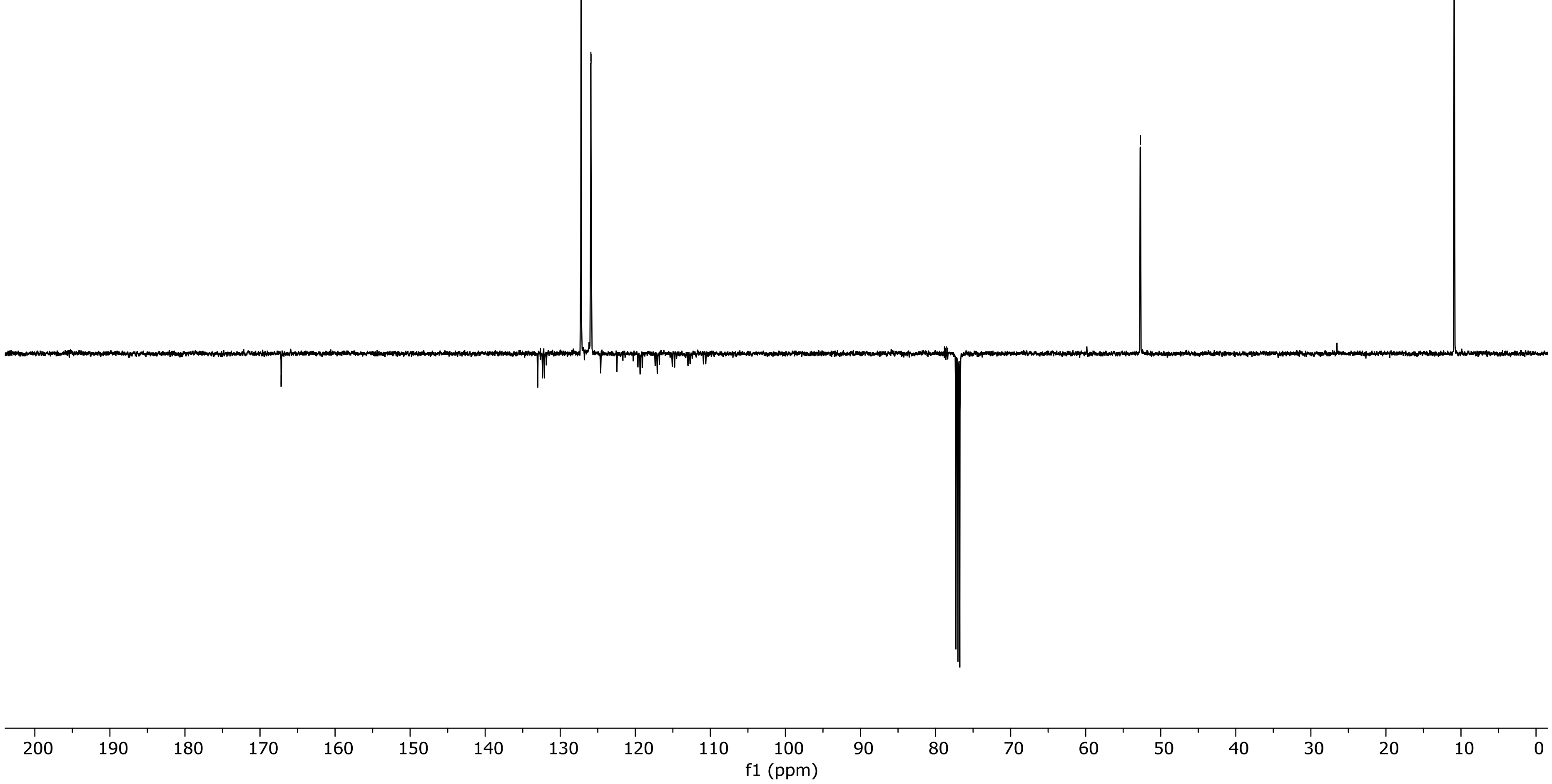

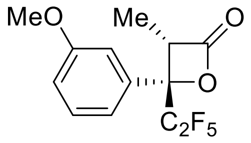

**31**

$^1H$, $CDCl_3$, 400 MHz

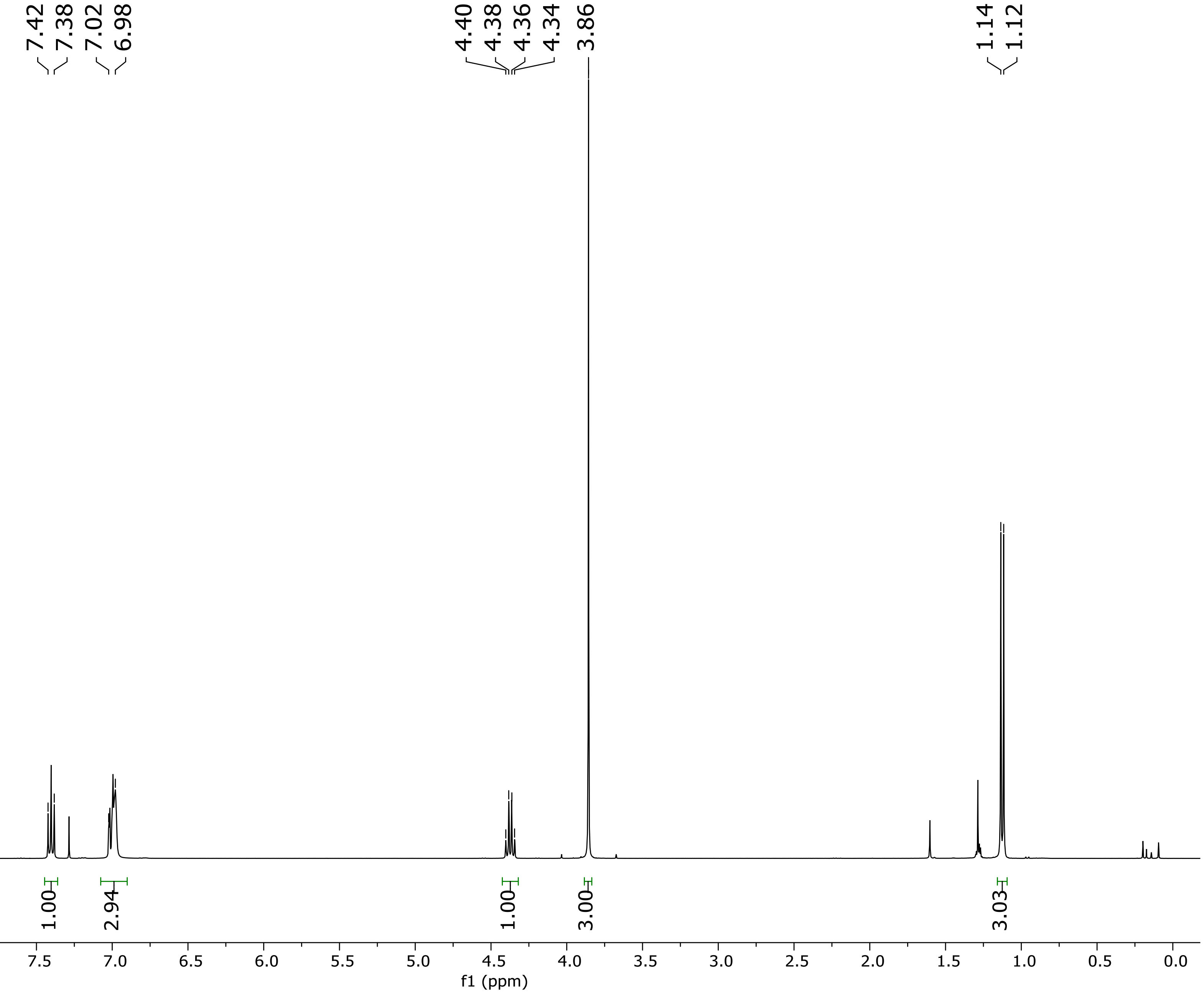

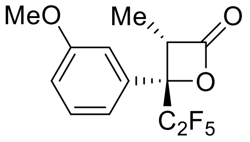


**31**

$^{19}F$, $CDCl_3$, 376 MHz

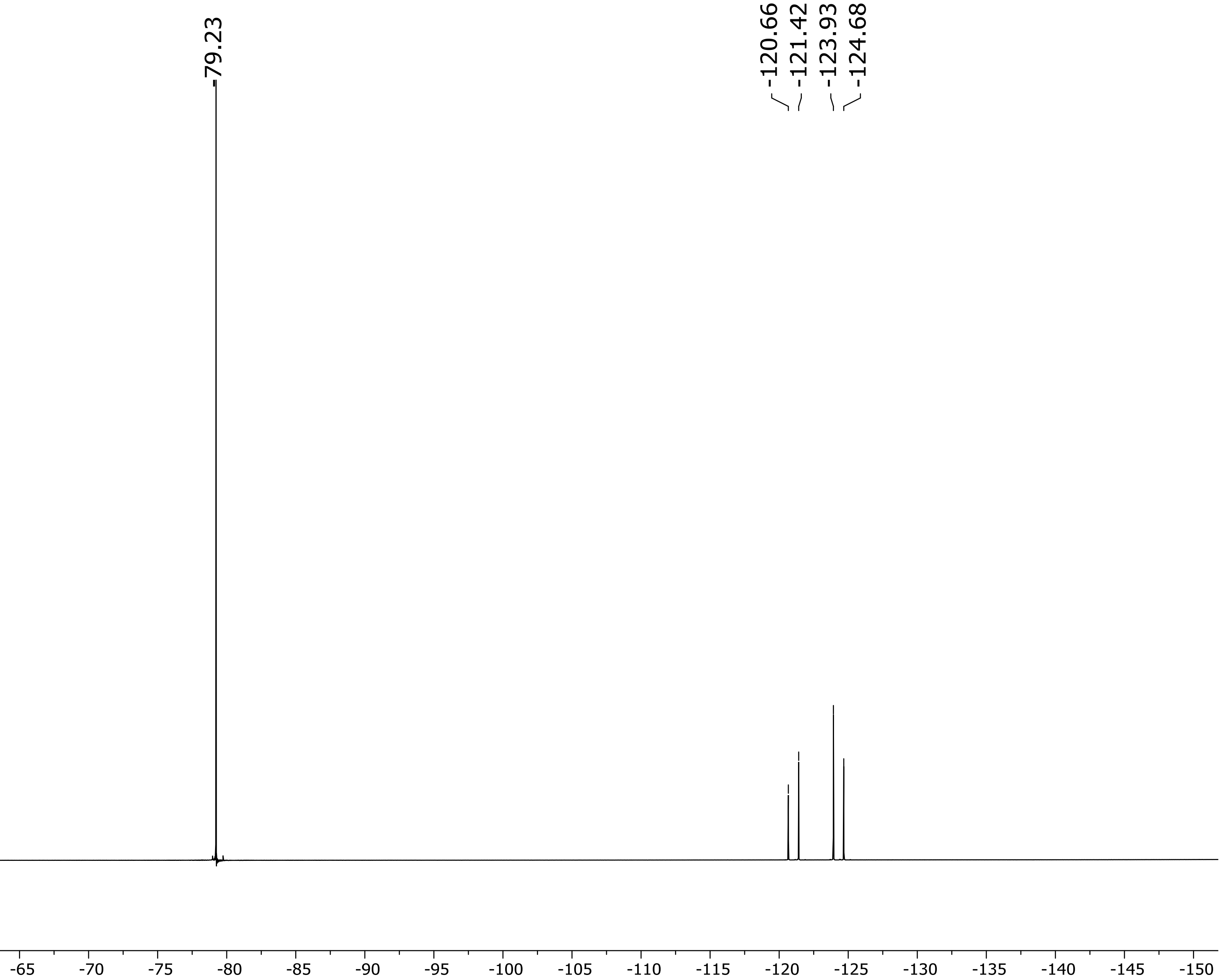

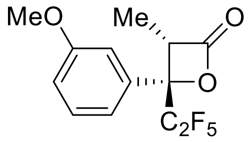


**31**

$^{13}C$, $CDCl_3$, 126 MHz

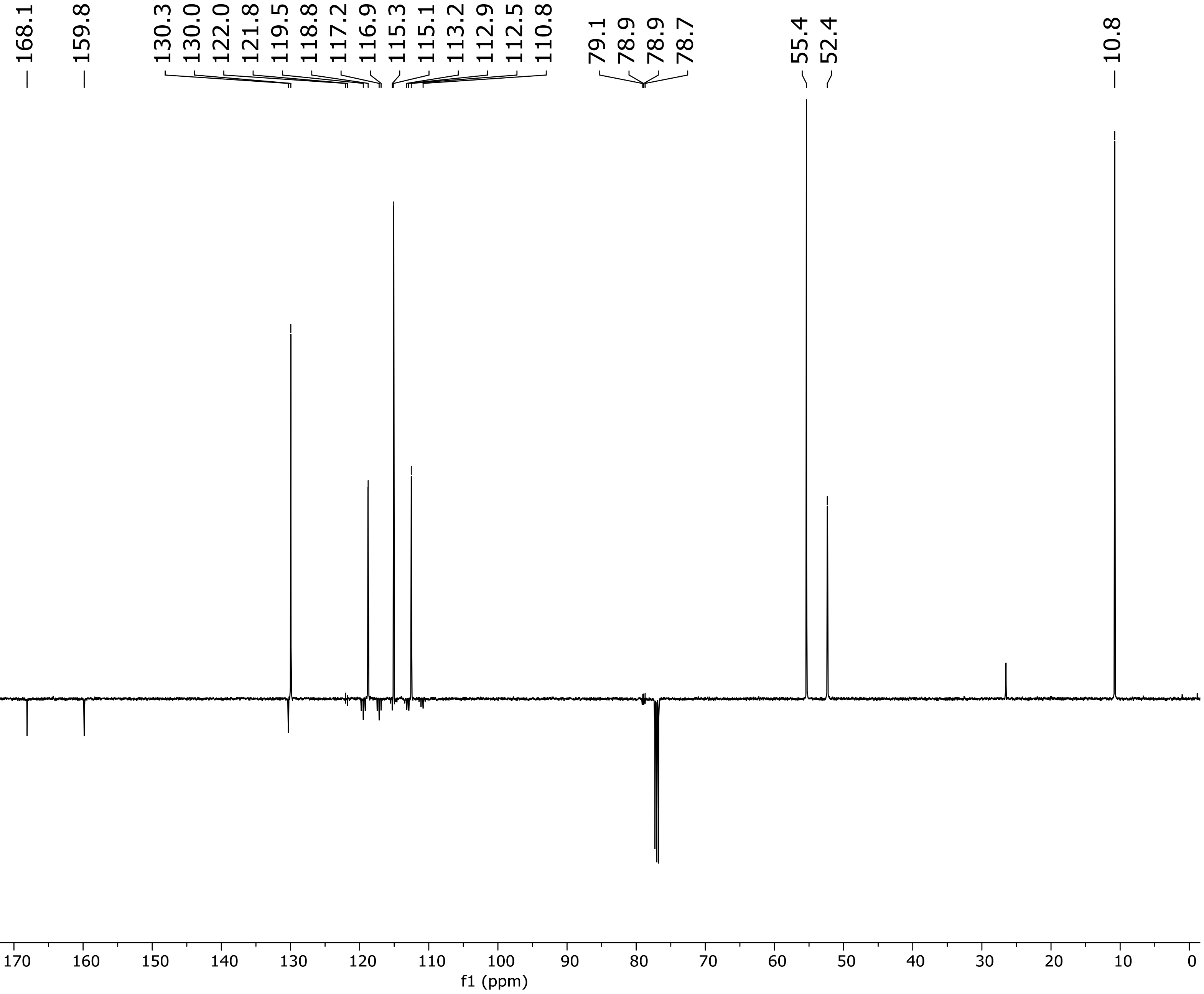

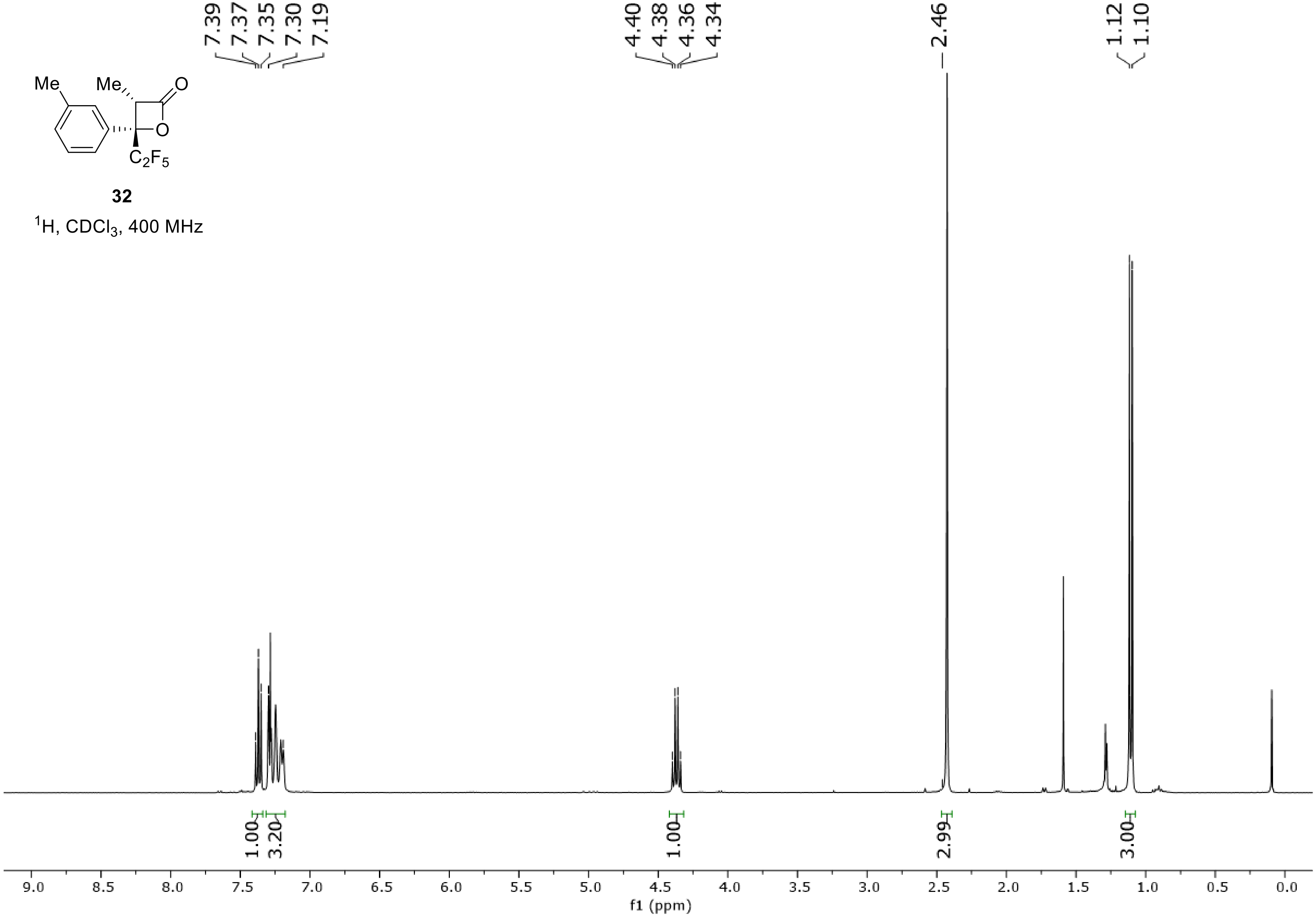


**32**

$^1H$, $CDCl_3$, 400 MHz

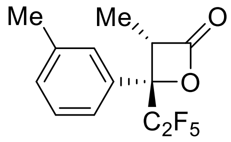

**32**

$^{19}F$, $CDCl_3$, 376 MHz

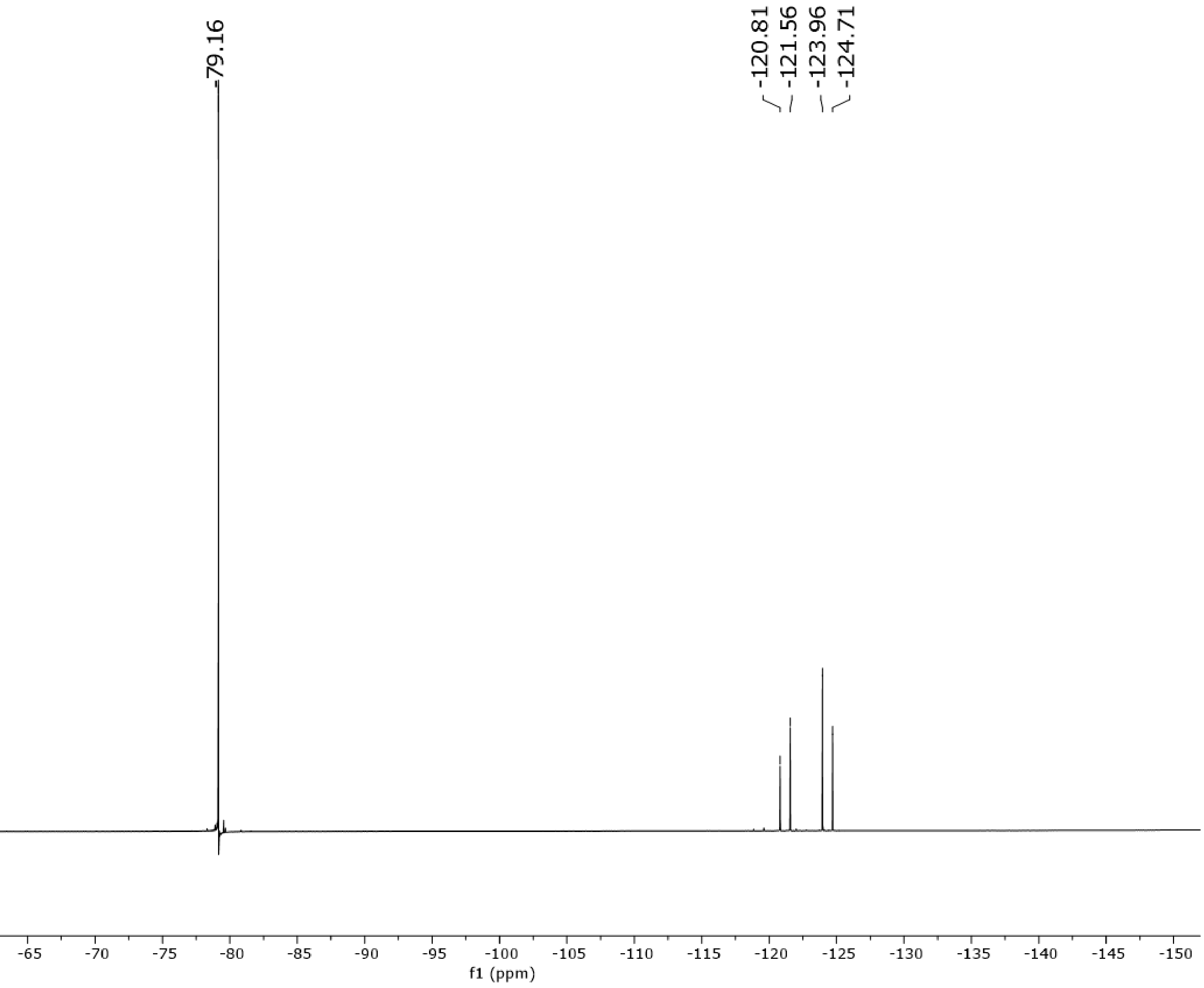

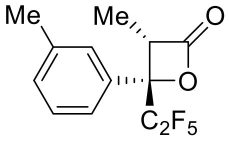

**32**

$^{13}C$, $CDCl_3$, 126 MHz

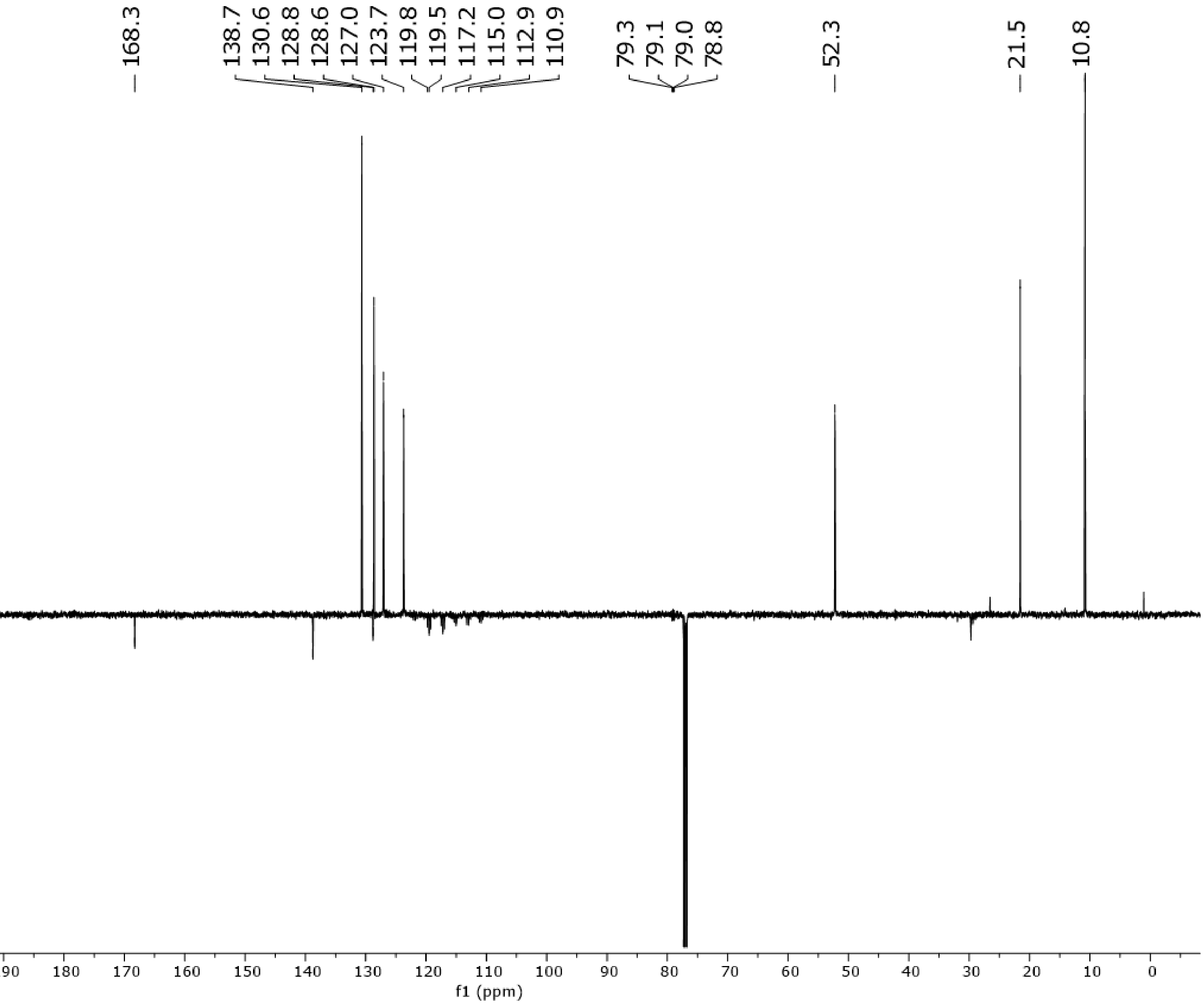

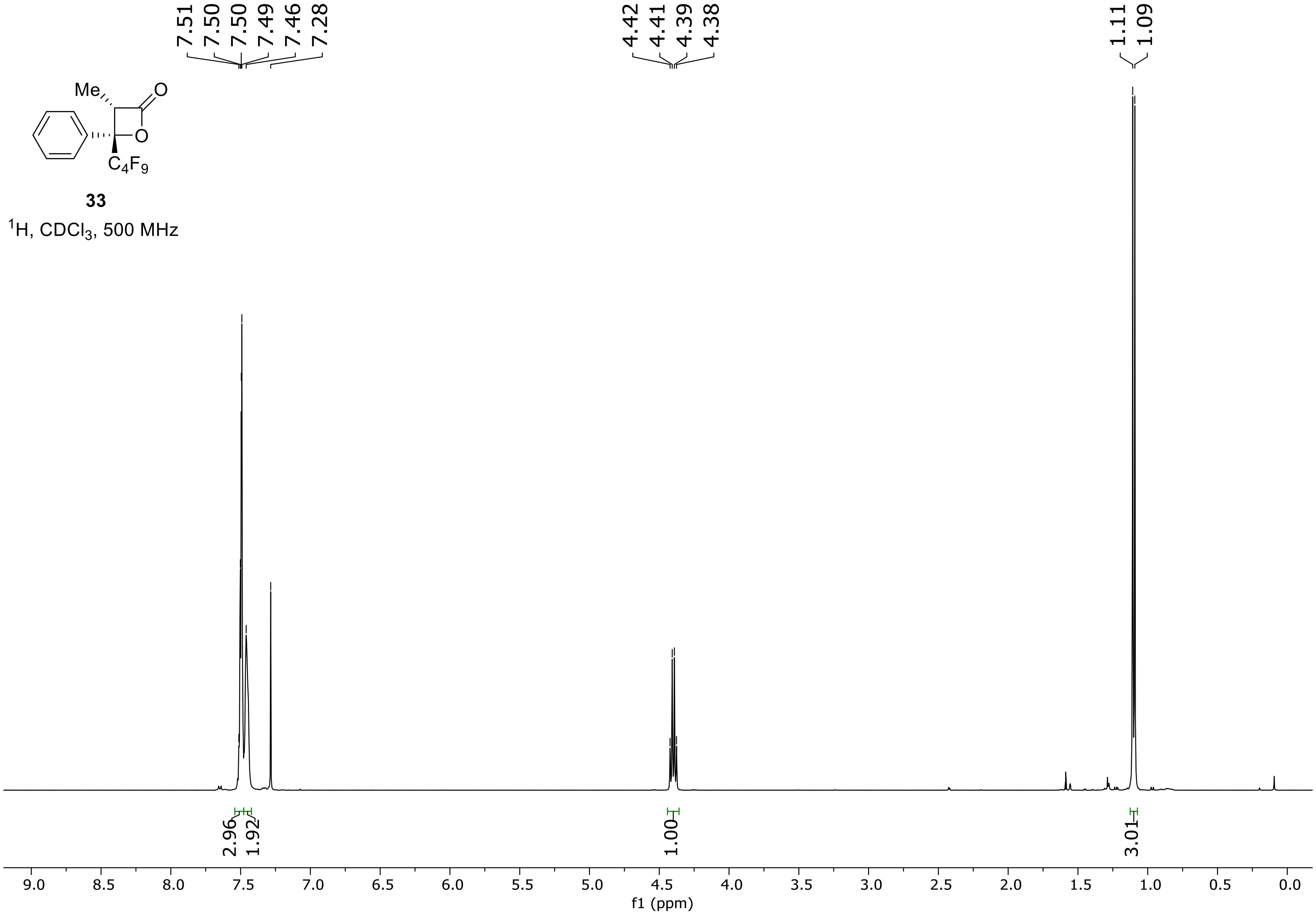
7.51
7.50
7.50
7.49
7.46
7.28
4.42
4.41
4.39
4.38
1.11
1.09
Me
O
O
$C_4F_9$
33
$^1$H, $CDCl_3$, 500 MHz
2.96
1.92
1.00
3.01
9.0
8.5
8.0
7.5
7.0
6.5
6.0
5.5
5.0
4.5
4.0
3.5
3.0
2.5
2.0
1.5
1.0
0.5
0.0
f1 (ppm)

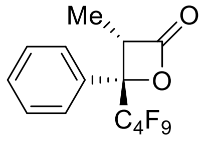

**33**

$^{19}F$, $CDCl_3$, 376 MHz

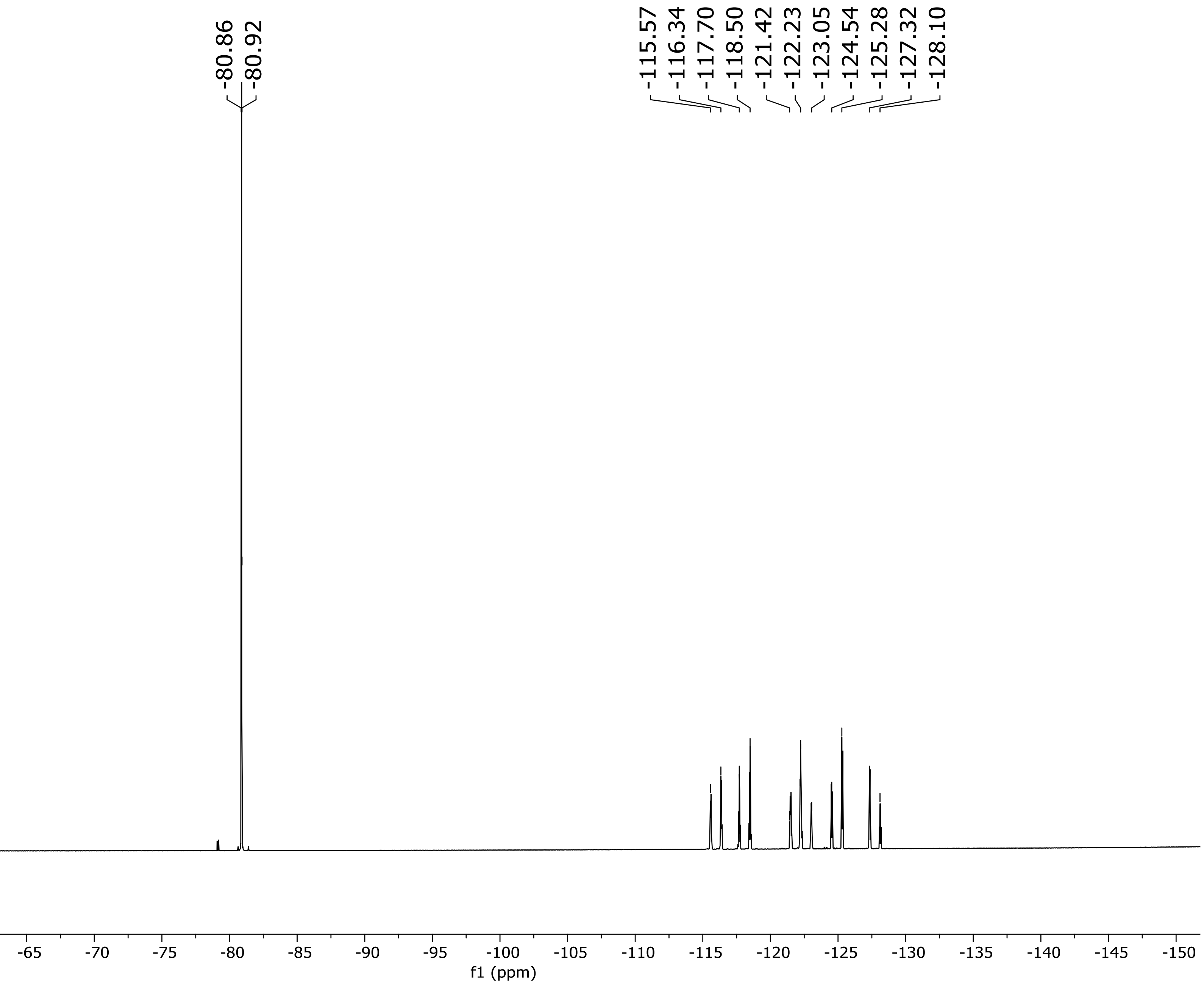

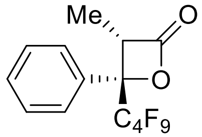

**33**

$^{13}C$, $CDCl_3$, 126 MHz

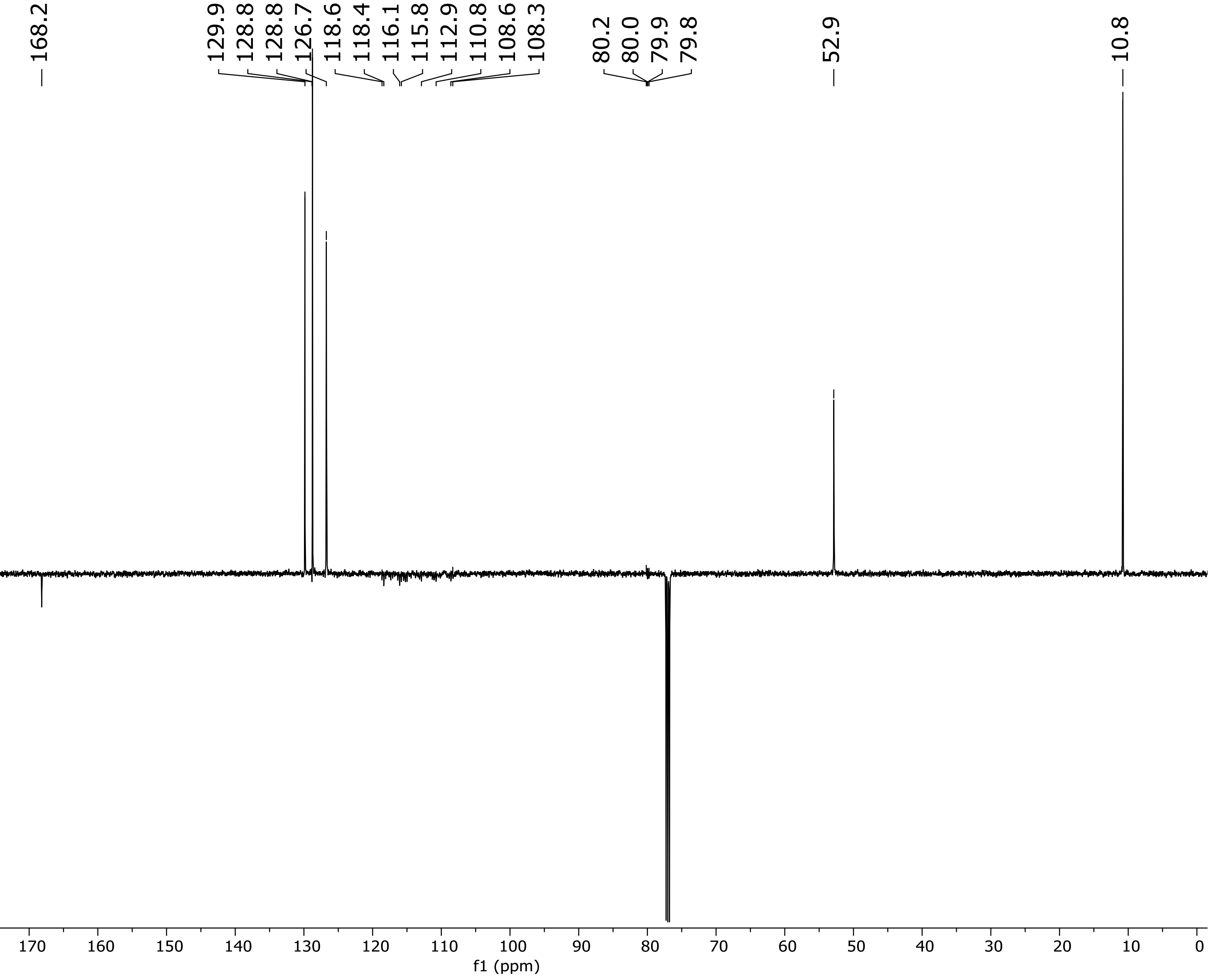

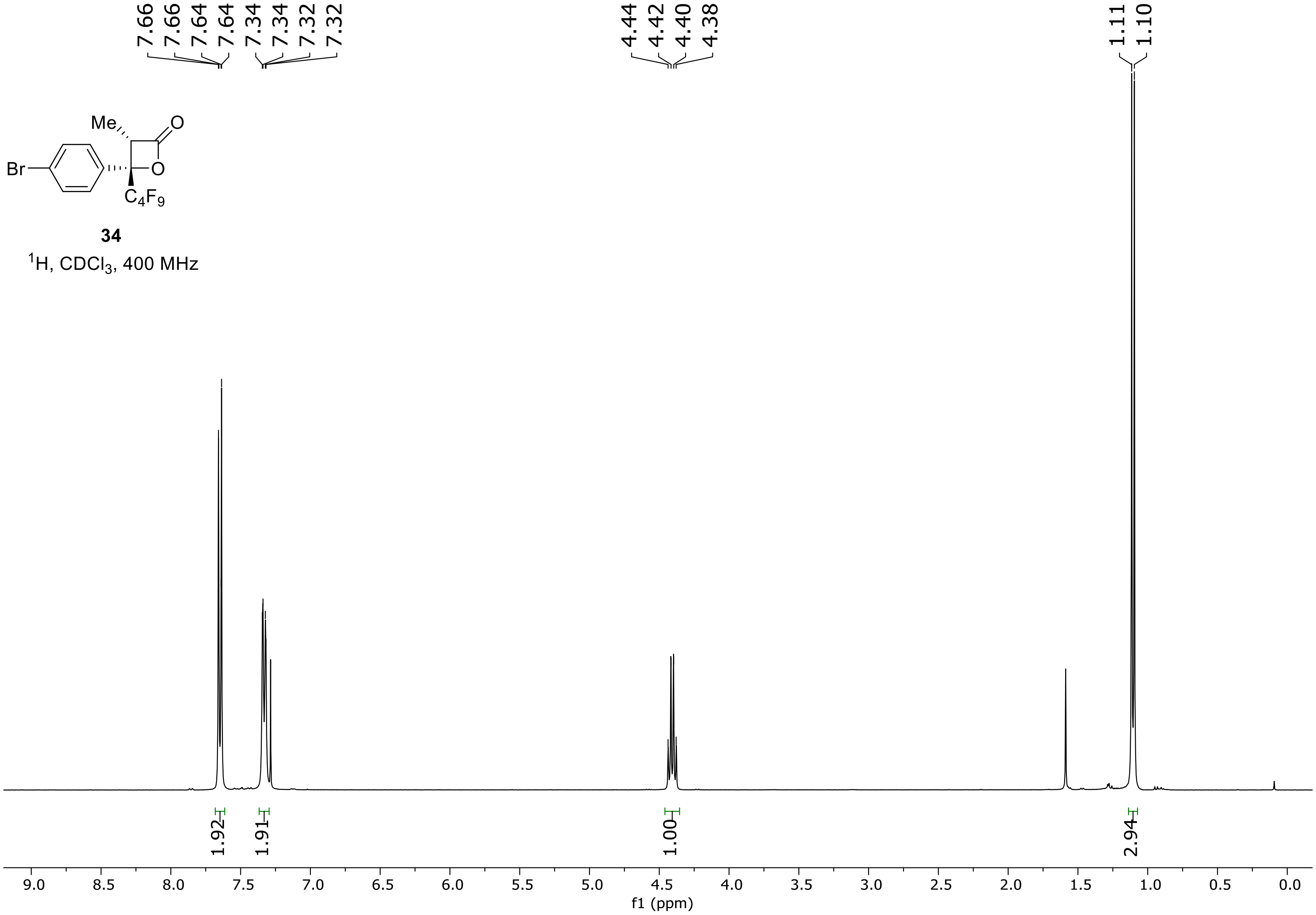


**34**

$^1H$, $CDCl_3$, 400 MHz

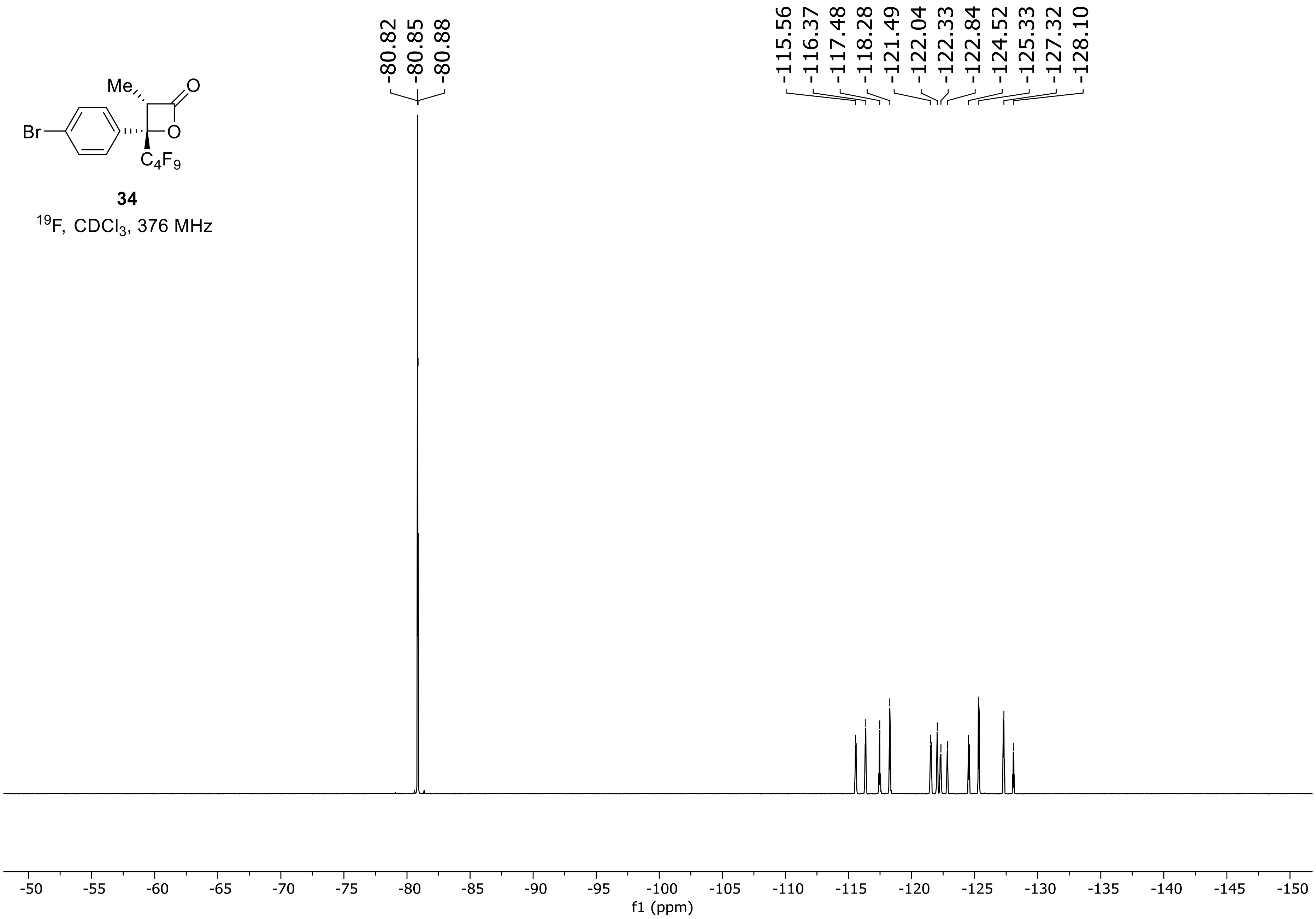
Me
O
Br
O
C4F9
34
19F, CDCl3, 376 MHz
-80.82
-80.85
-80.88
-115.56
-116.37
-117.48
-118.28
-121.49
-122.04
-122.33
-122.84
-124.52
-125.33
-127.32
-128.10
-50
-55
-60
-65
-70
-75
-80
-85
-90
-95
-100
-105
-110
-115
-120
-125
-130
-135
-140
-145
-150
f1 (ppm)

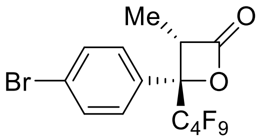


**34**

$^{13}C$, $CDCl_3$, 126 MHz

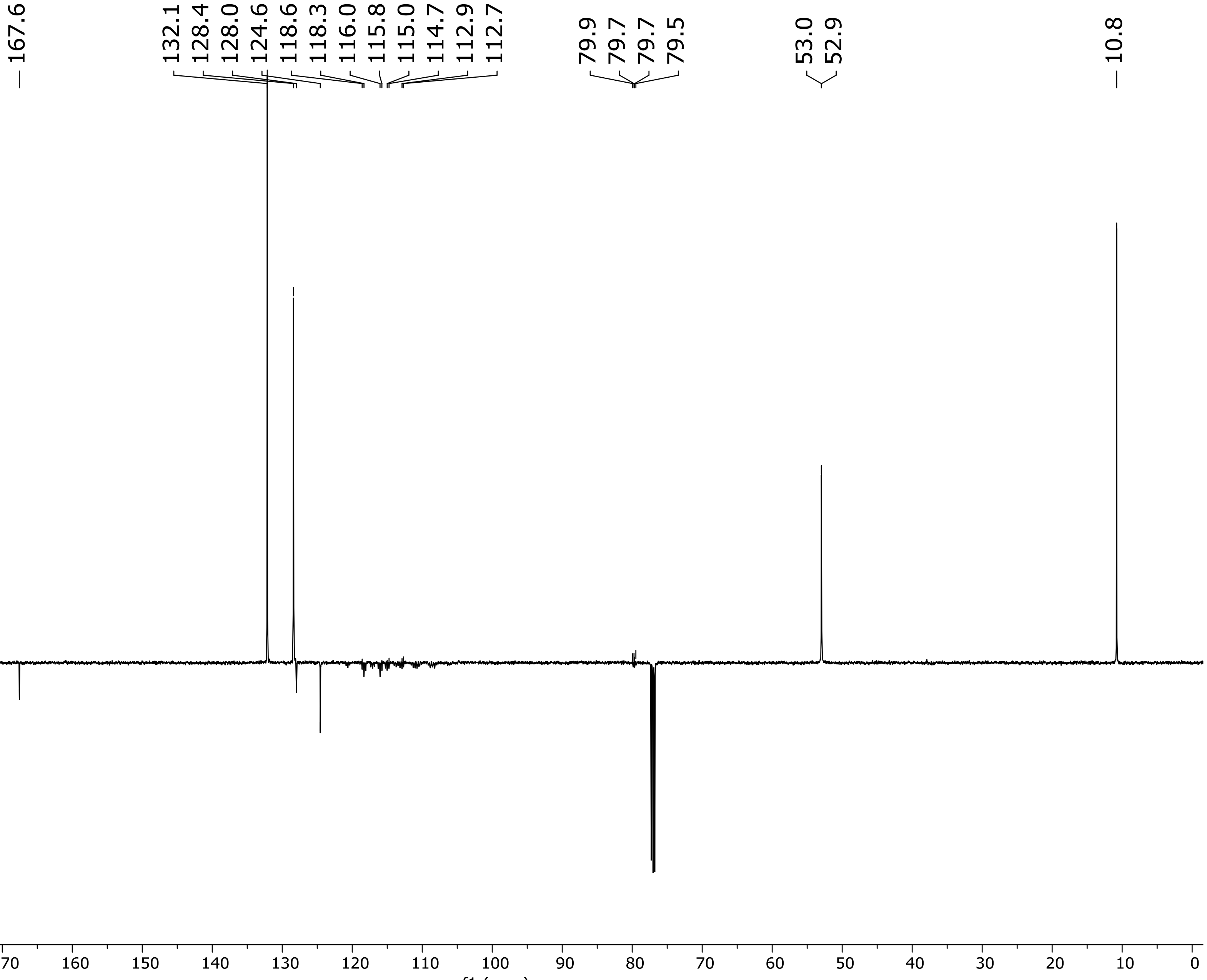

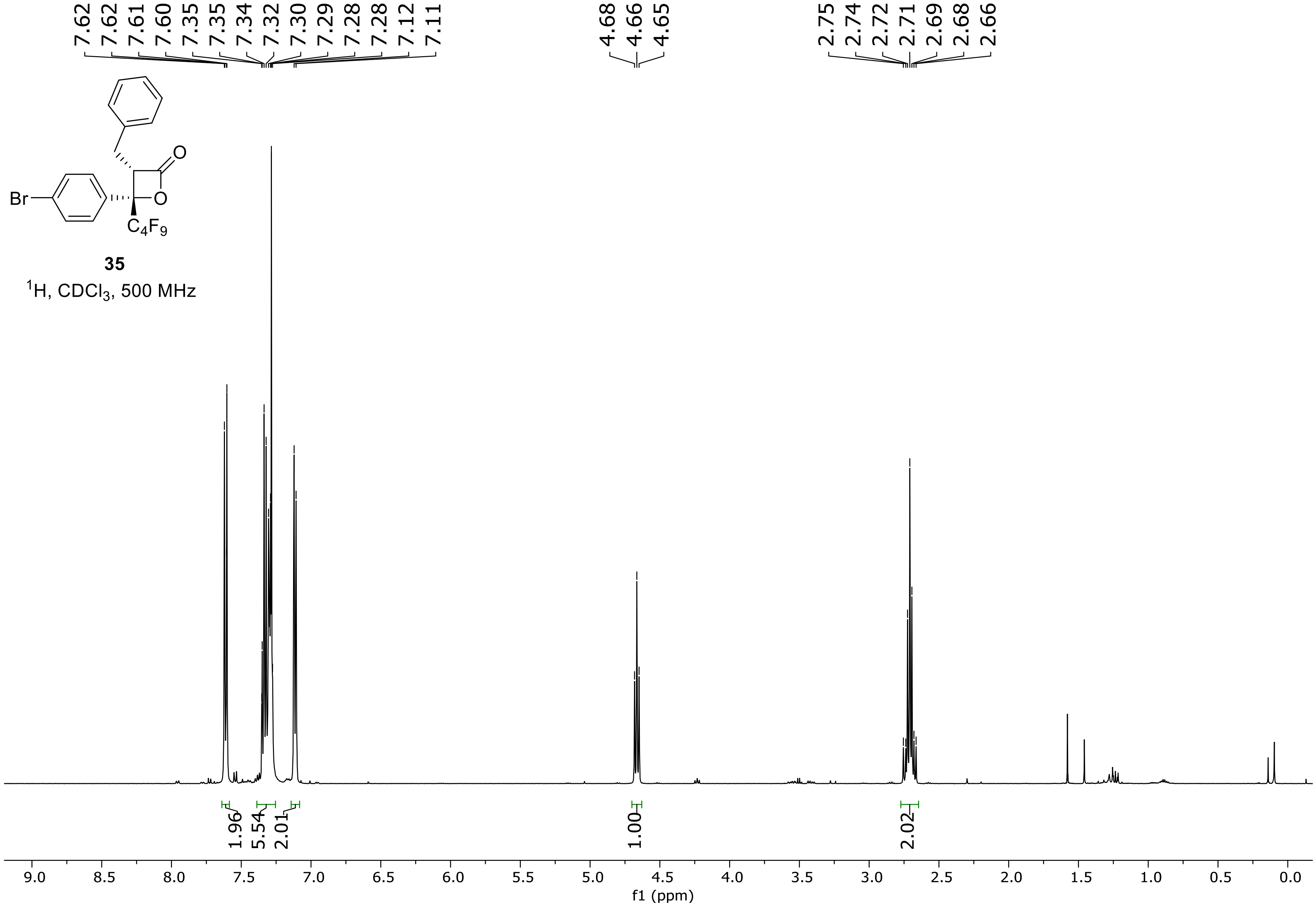

35
1H, CDCl3, 500 MHz
7.62
7.62
7.61
7.60
7.35
7.35
7.34
7.32
7.30
7.29
7.28
7.28
7.12
7.11
4.68
4.66
4.65
2.75
2.74
2.72
2.71
2.69
2.68
2.66
1.96
5.54
2.01
1.00
2.02
9.0 8.5 8.0 7.5 7.0 6.5 6.0 5.5 5.0 4.5 4.0 3.5 3.0 2.5 2.0 1.5 1.0 0.5 0.0
f1 (ppm)

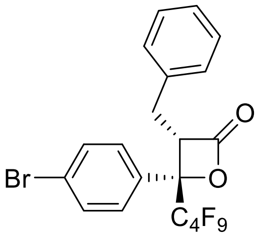

**35**

$^{19}F$, $CDCl_3$, 471 MHz

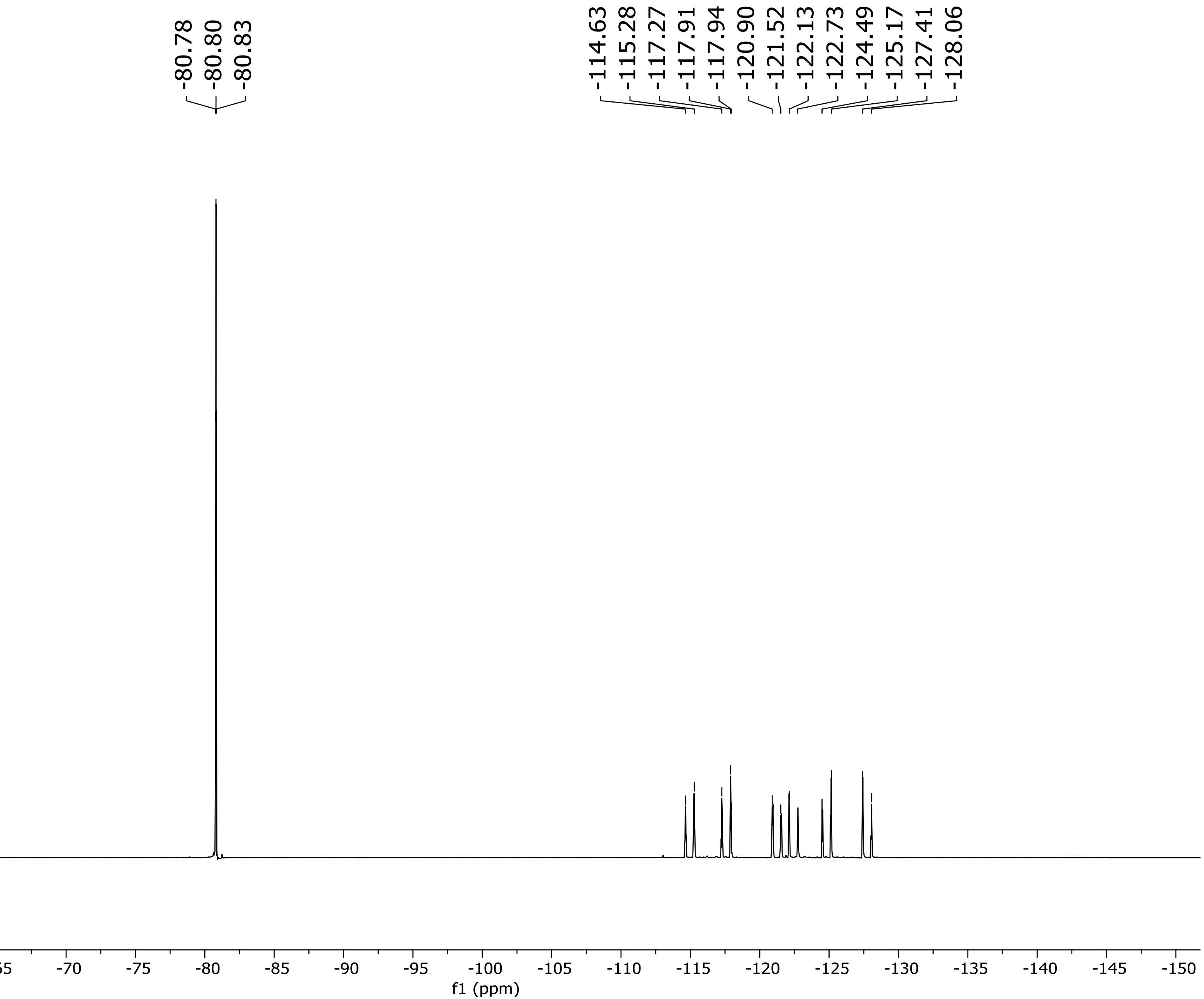

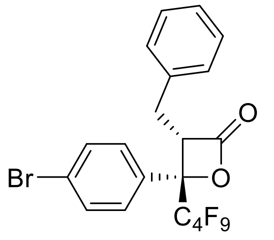

**35**

$^{13}C$, $CDCl_3$, 126 MHz

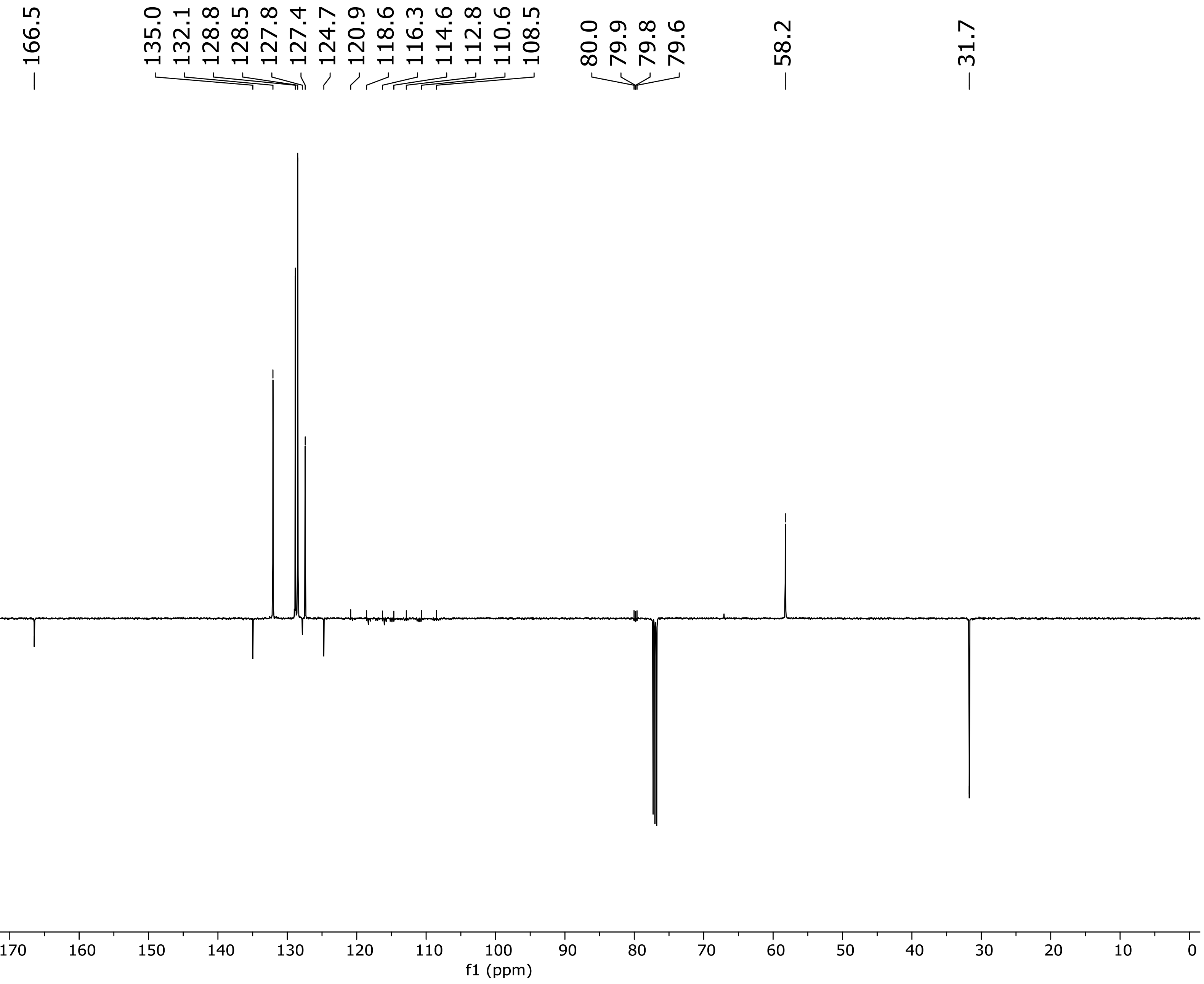

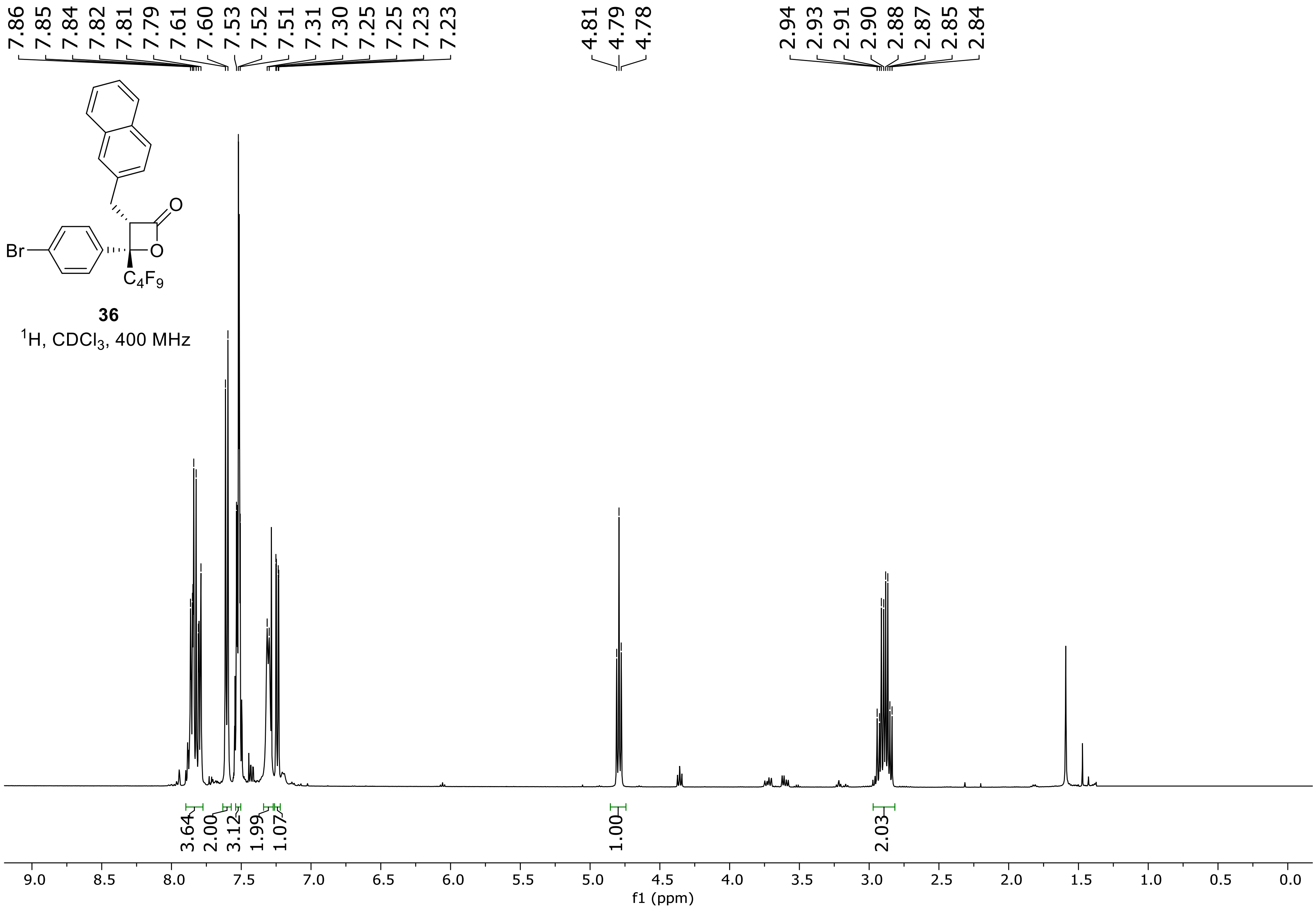
7.86
7.85
7.84
7.82
7.81
7.79
7.61
7.60
7.53
7.52
7.51
7.31
7.30
7.25
7.25
7.23
7.23
4.81
4.79
4.78
2.94
2.93
2.91
2.90
2.88
2.87
2.85
2.84
Br
O
O
$C_4F_9$
**36**
$^1$H, $CDCl_3$, 400 MHz
3.64
2.00
3.12
1.99
1.07
1.00
2.03
9.0
8.5
8.0
7.5
7.0
6.5
6.0
5.5
5.0
4.5
4.0
3.5
3.0
2.5
2.0
1.5
1.0
0.5
0.0
f1 (ppm)

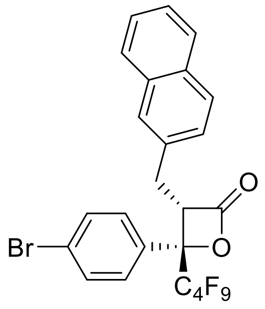


**36**

$^{19}F$, $CDCl_3$, 376 MHz

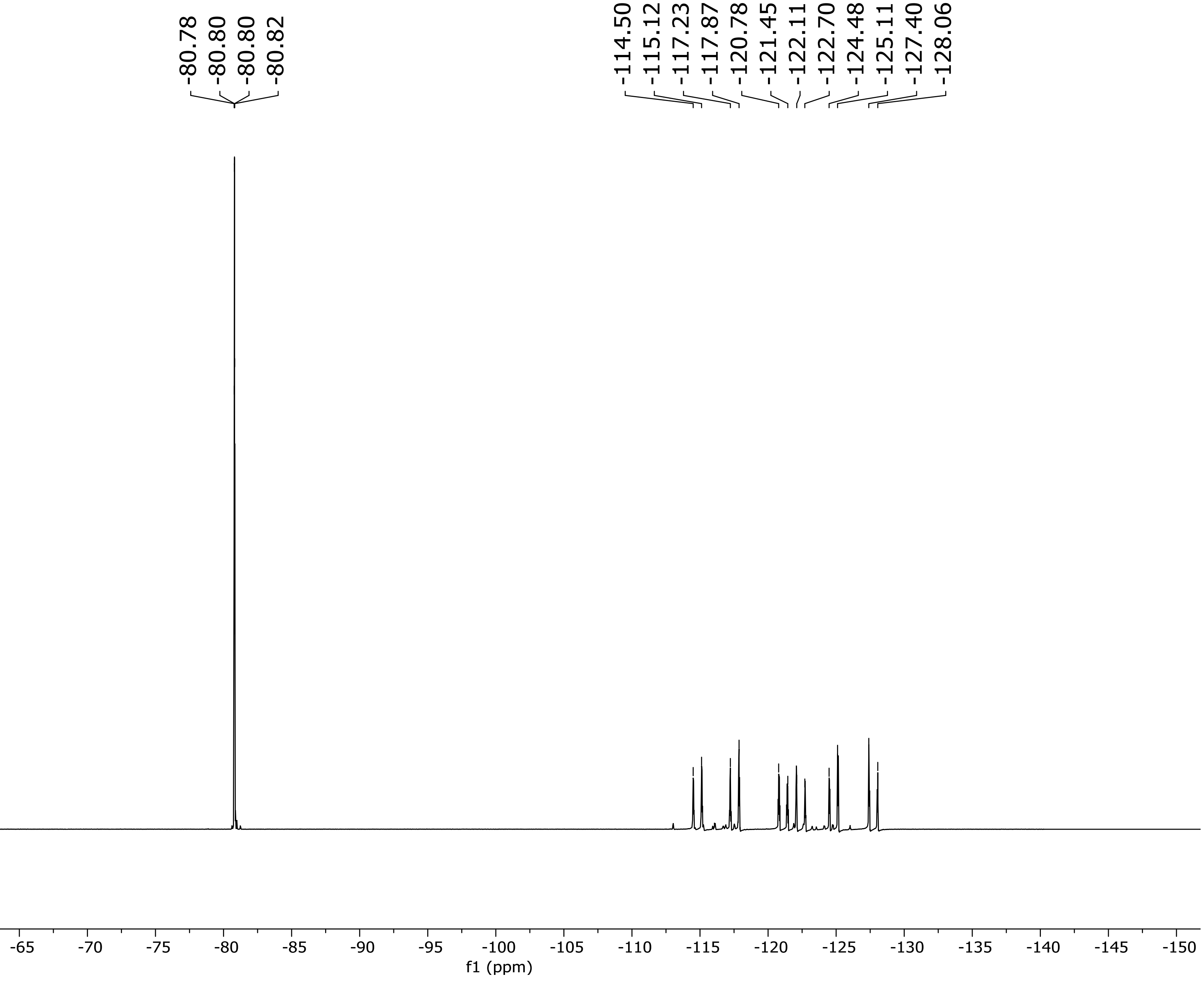

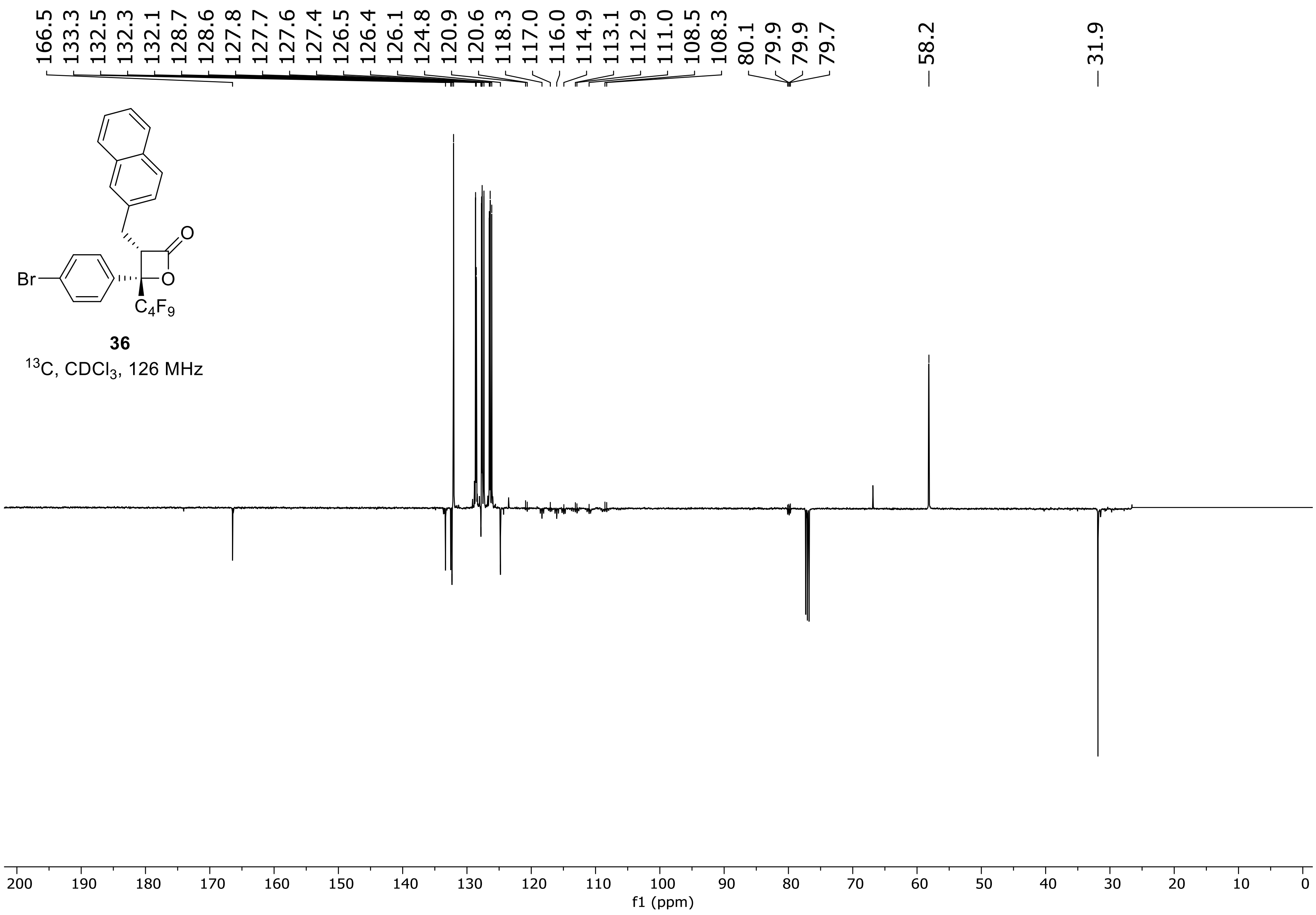
166.5 133.3 132.5 132.3 132.1 128.7 128.6 127.8 127.7 127.6 127.4 126.5 126.4 126.1 124.8 120.9 120.6 118.3 117.0 116.0 114.9 113.1 112.9 111.0 108.5 108.3 80.1 79.9 79.9 79.7 58.2 31.9
Br
O
O
$C_4F_9$
**36**
$^{13}$C, $CDCl_3$, 126 MHz
200 190 180 170 160 150 140 130 120 110 100 90 80 70 60 50 40 30 20 10 0
f1 (ppm)

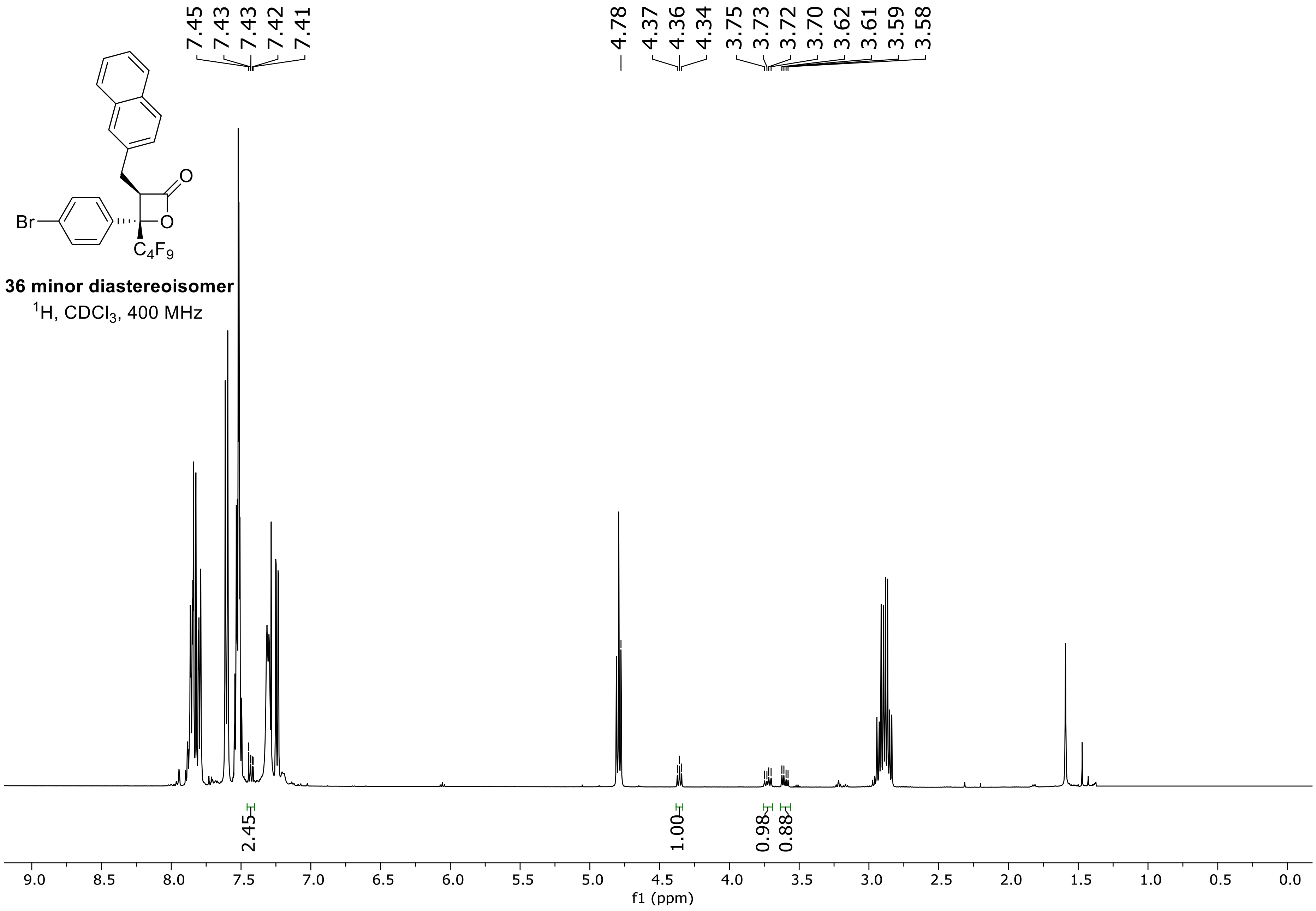

7.45
7.43
7.43
7.42
7.41
4.78
4.37
4.36
4.34
3.75
3.73
3.72
3.70
3.62
3.61
3.59
3.58
Br
O
O
C4F9
36 minor diastereoisomer
1H, CDCl3, 400 MHz
2.45
1.00
0.98
0.88
9.0
8.5
8.0
7.5
7.0
6.5
6.0
5.5
5.0
4.5
4.0
3.5
3.0
2.5
2.0
1.5
1.0
0.5
0.0
f1 (ppm)

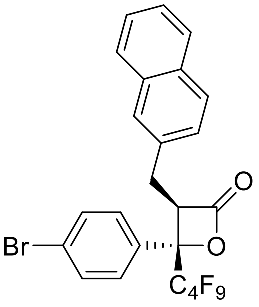


**36 minor diastereoisomer**

$^{13}C$, $CDCl_3$, 126 MHz

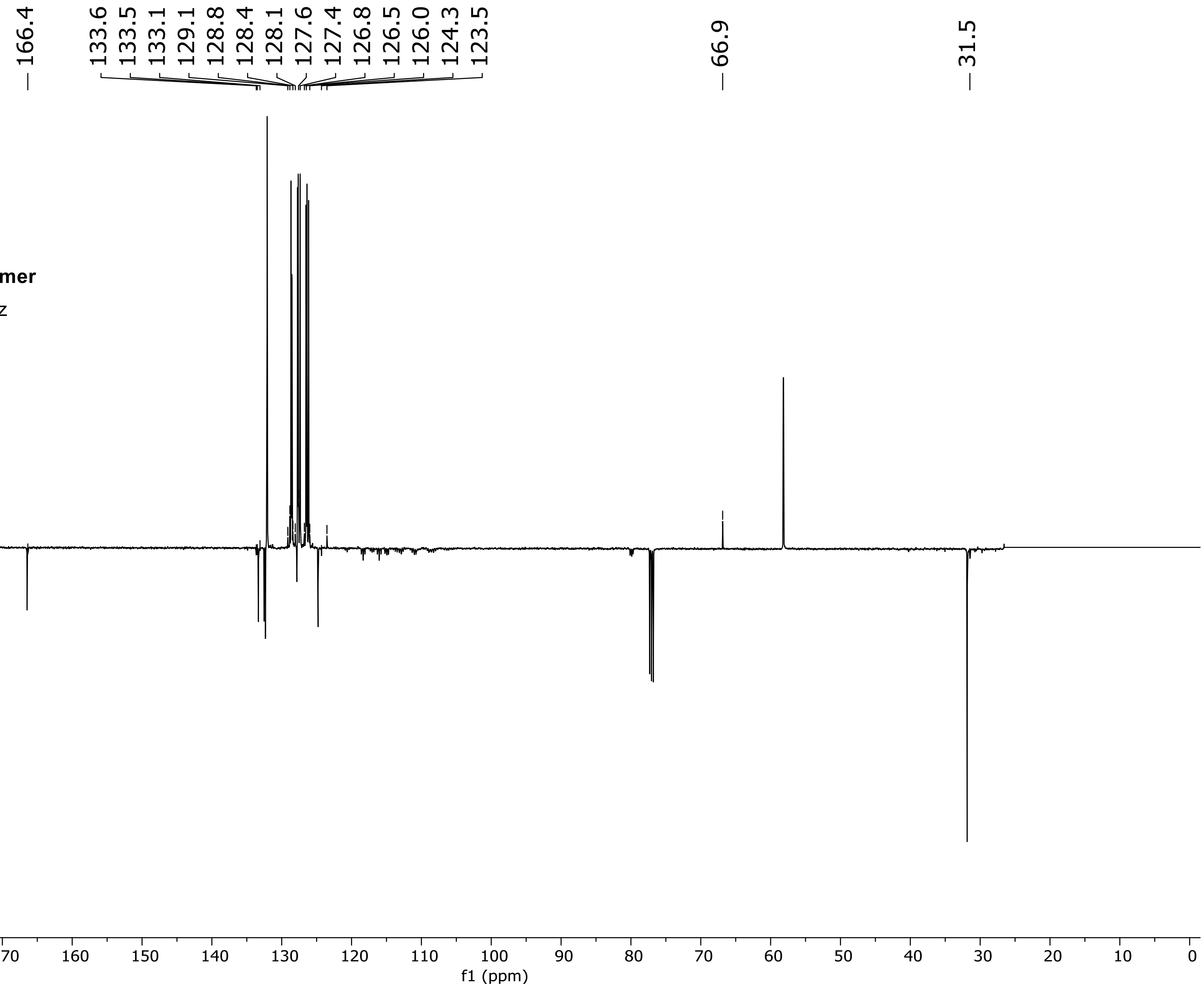

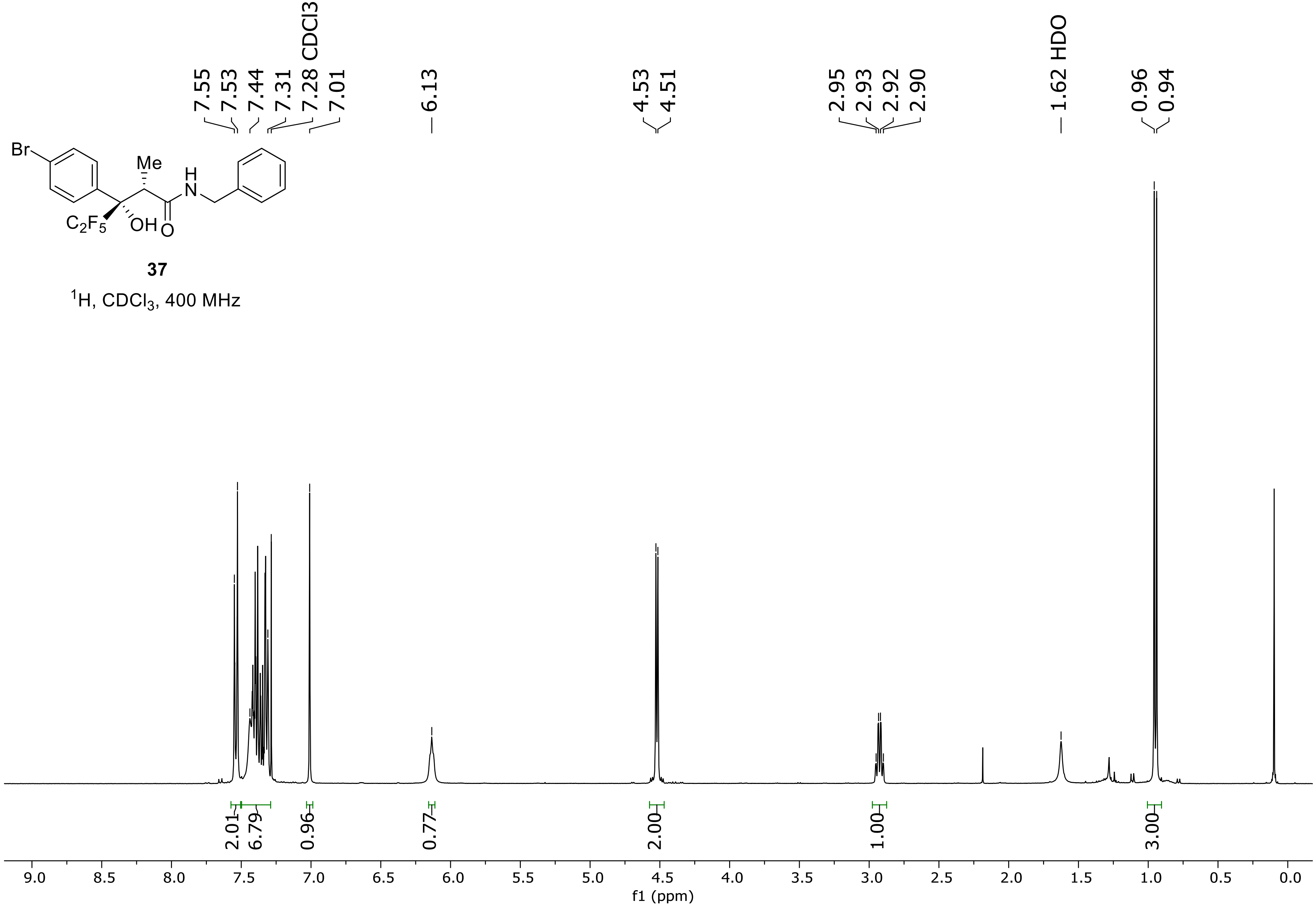


**37**

$^{1}H$, $CDCl_3$, 400 MHz

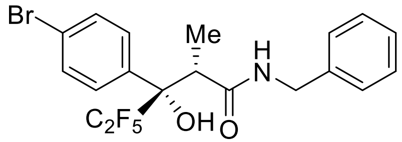

**37**

$^{19}F$, $CDCl_3$, 376 MHz

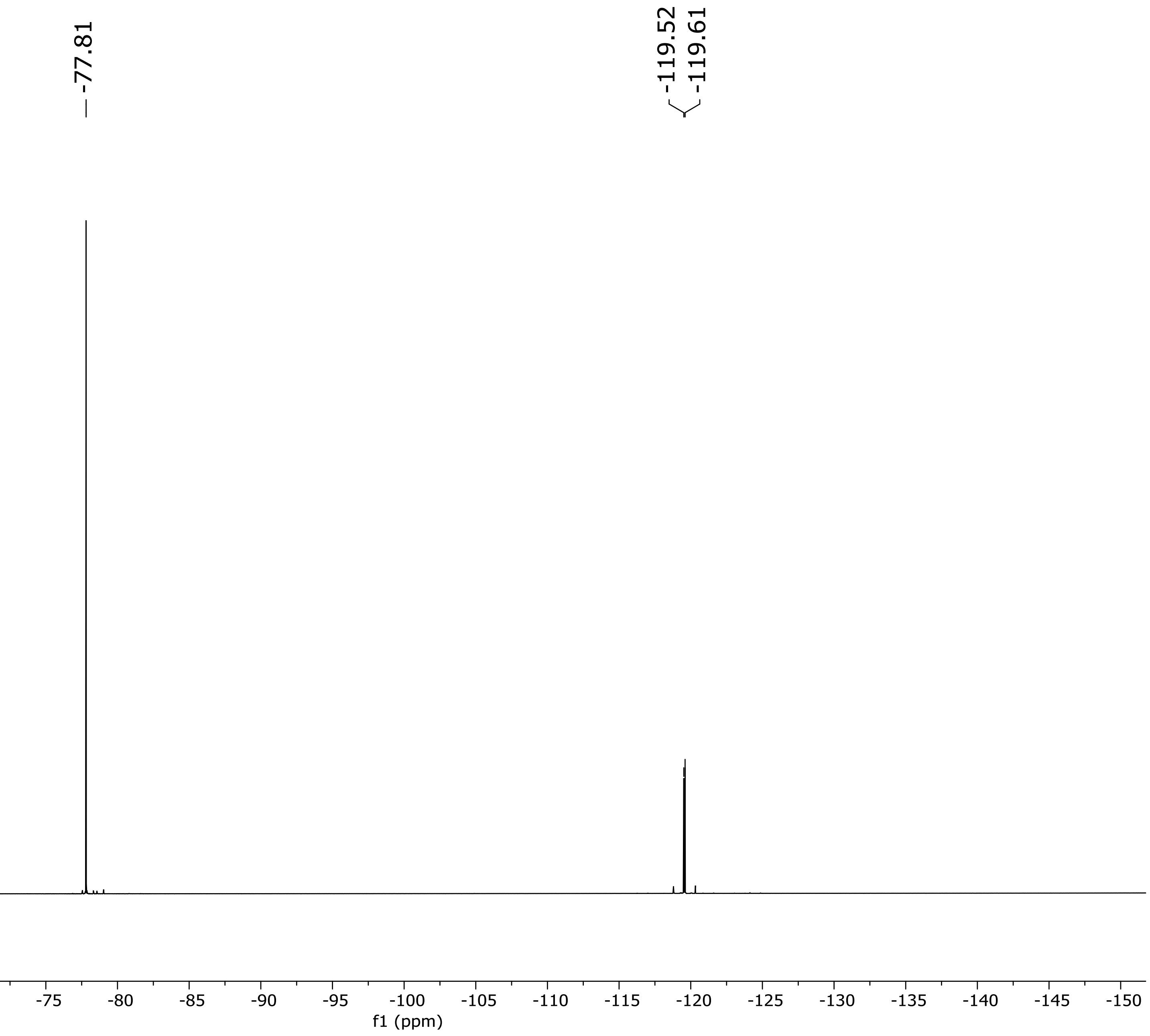

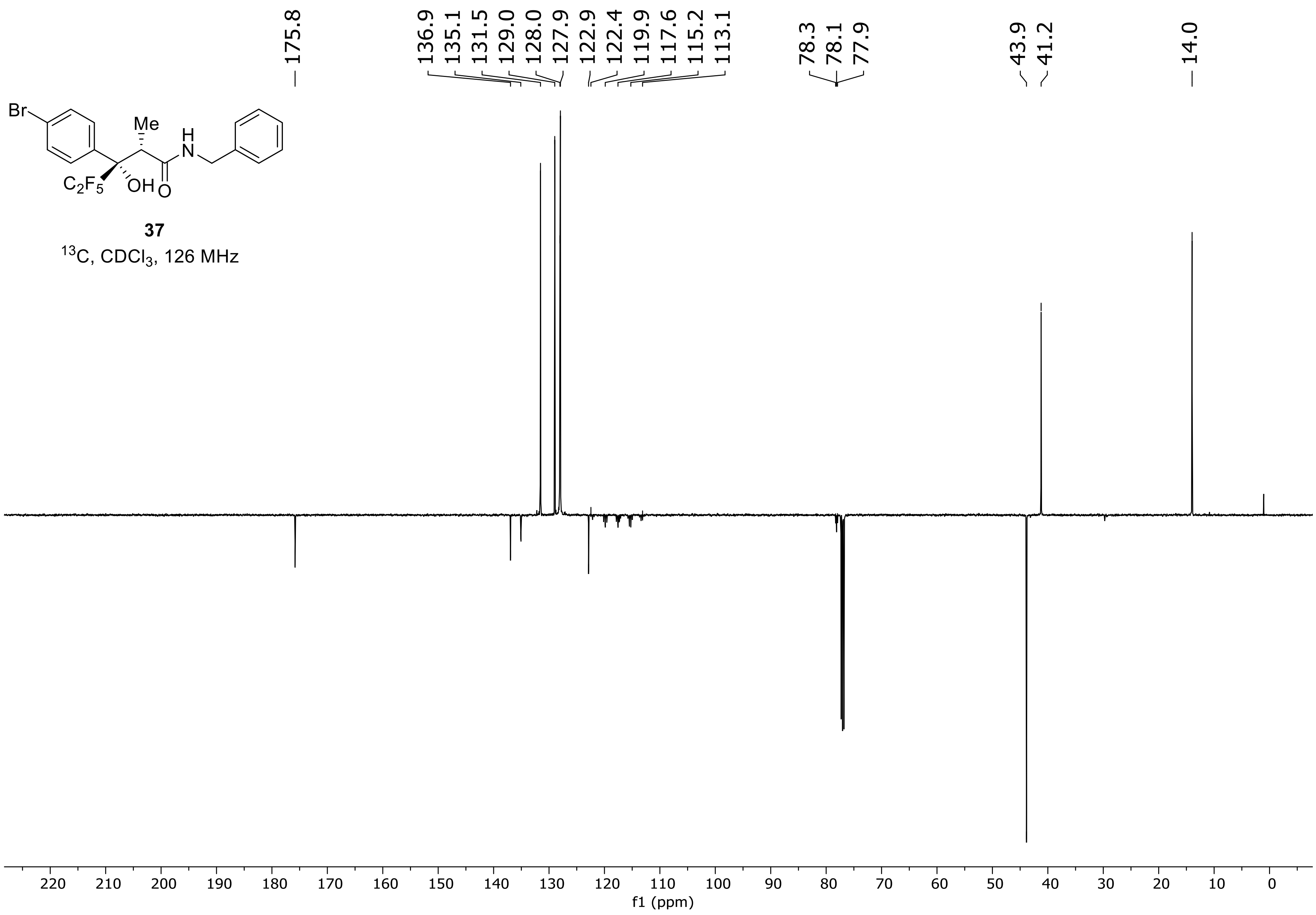


**37**

$^{13}C$, $CDCl_3$, 126 MHz

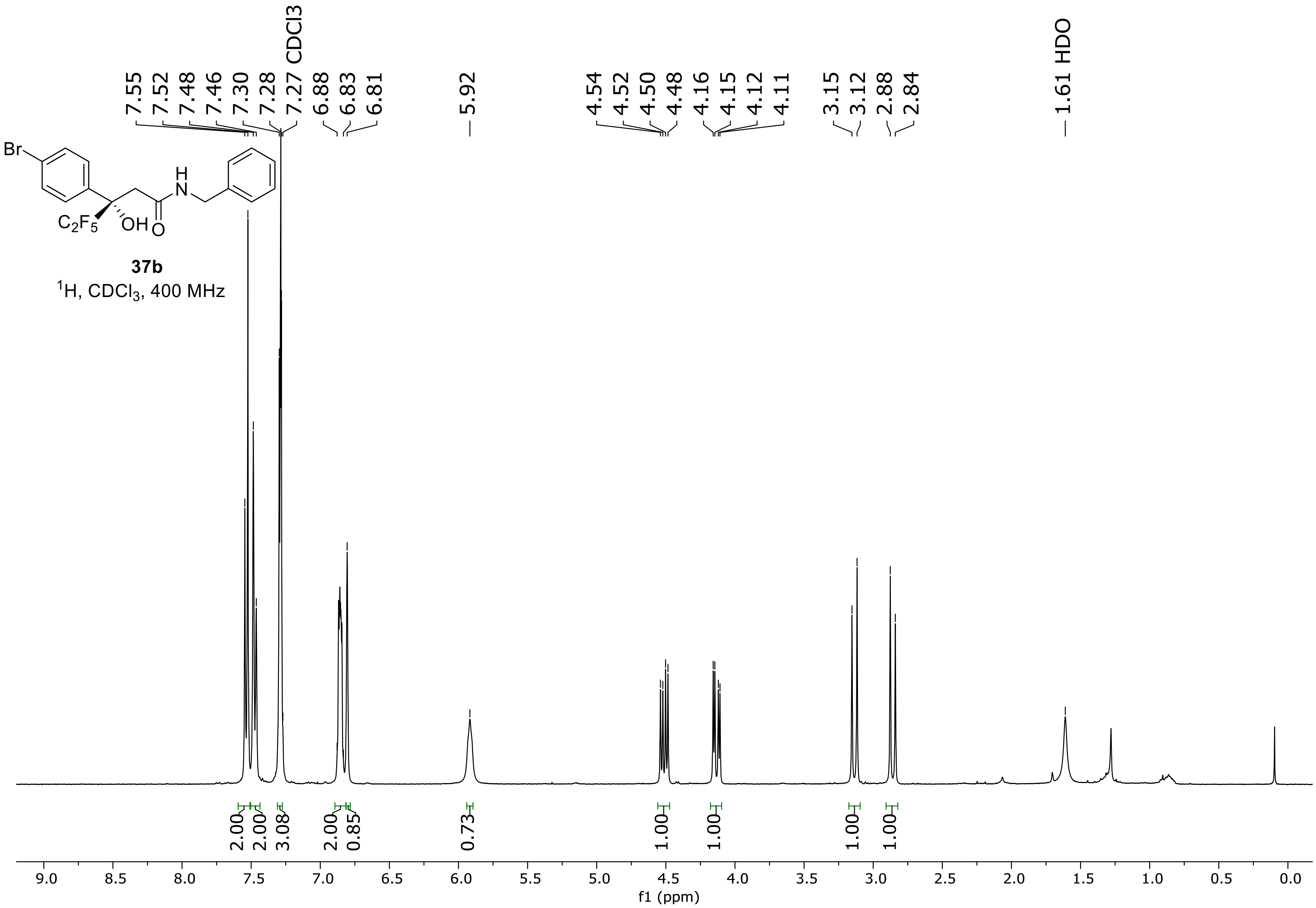
7.55
7.52
7.48
7.46
7.30
7.28
7.27 CDCl3
6.88
6.83
6.81
5.92
4.54
4.52
4.50
4.48
4.16
4.15
4.12
4.11
3.15
3.12
2.88
2.84
1.61 HDO
Br
H
N
$C_2F_5$
OH
O
37b
$^1$H, $CDCl_3$, 400 MHz
2.00
2.00
3.08
2.00
0.85
0.73
1.00
1.00
1.00
1.00
9.0
8.5
8.0
7.5
7.0
6.5
6.0
5.5
5.0
4.5
4.0
3.5
3.0
2.5
2.0
1.5
1.0
0.5
0.0
f1 (ppm)

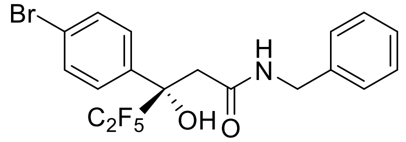


**37b**

$^{19}F$, $CDCl_3$, 376 MHz

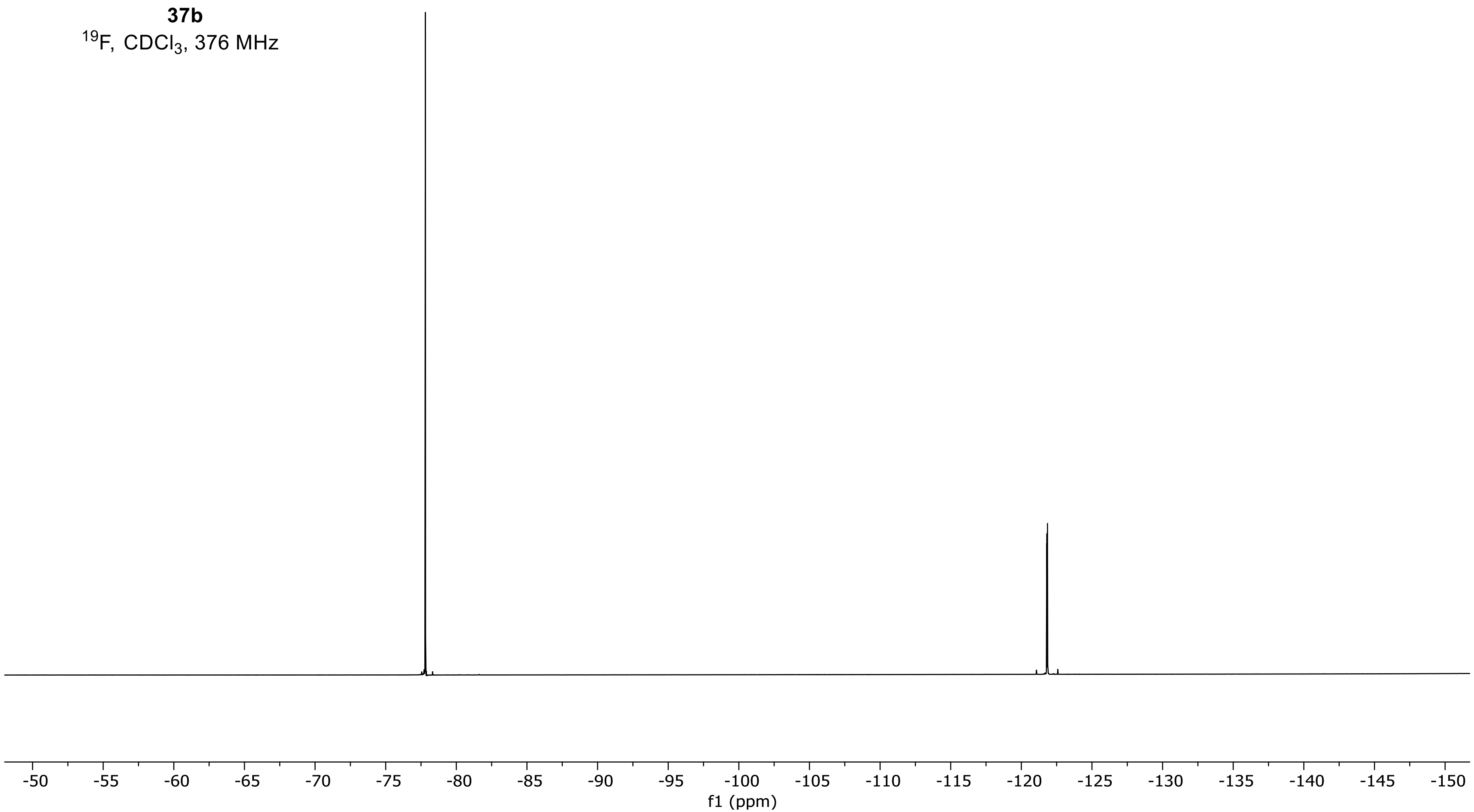

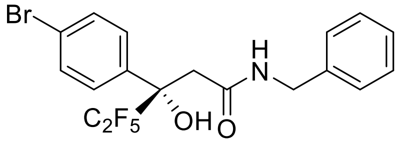

**37b**

$^{13}C$, $CDCl_3$, 126 MHz

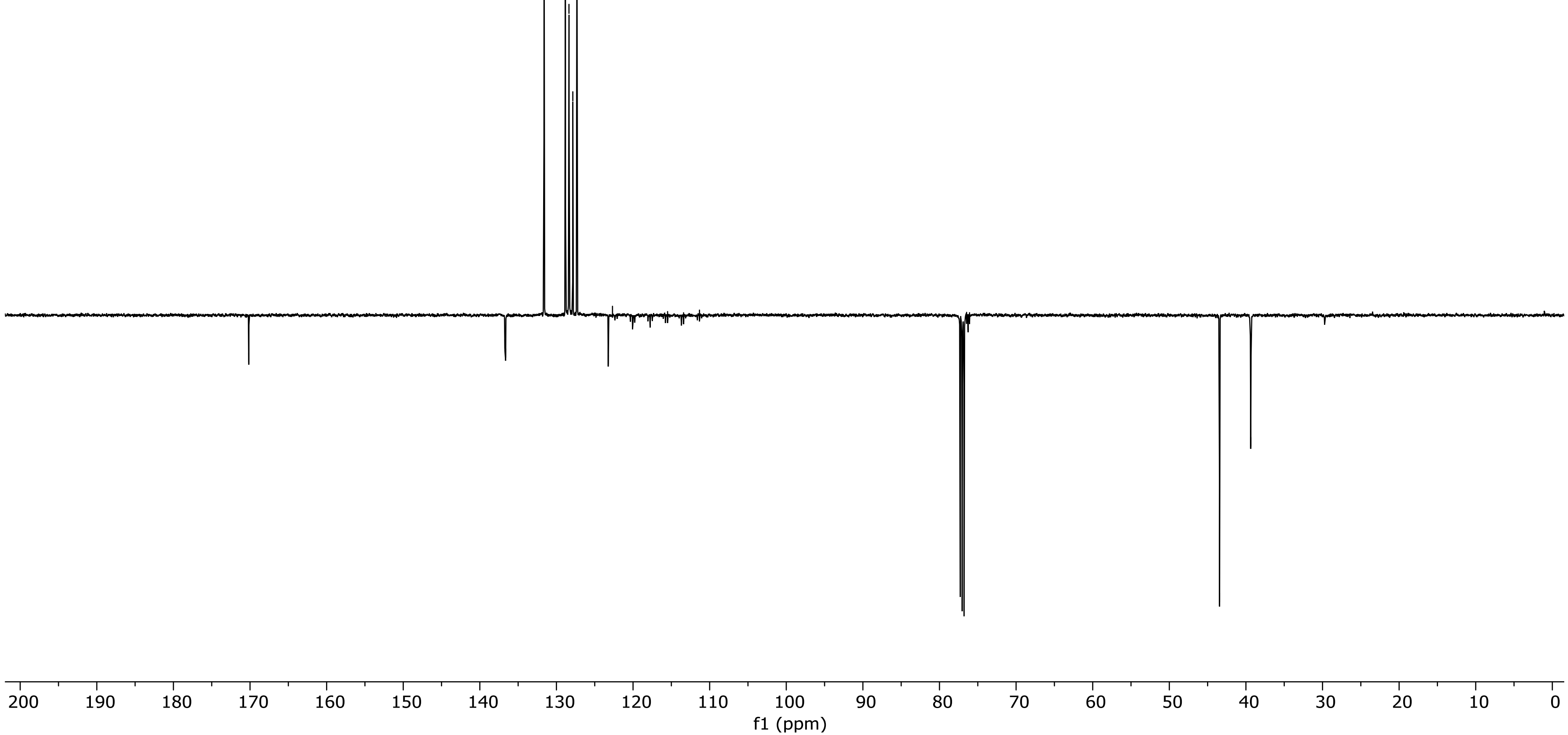

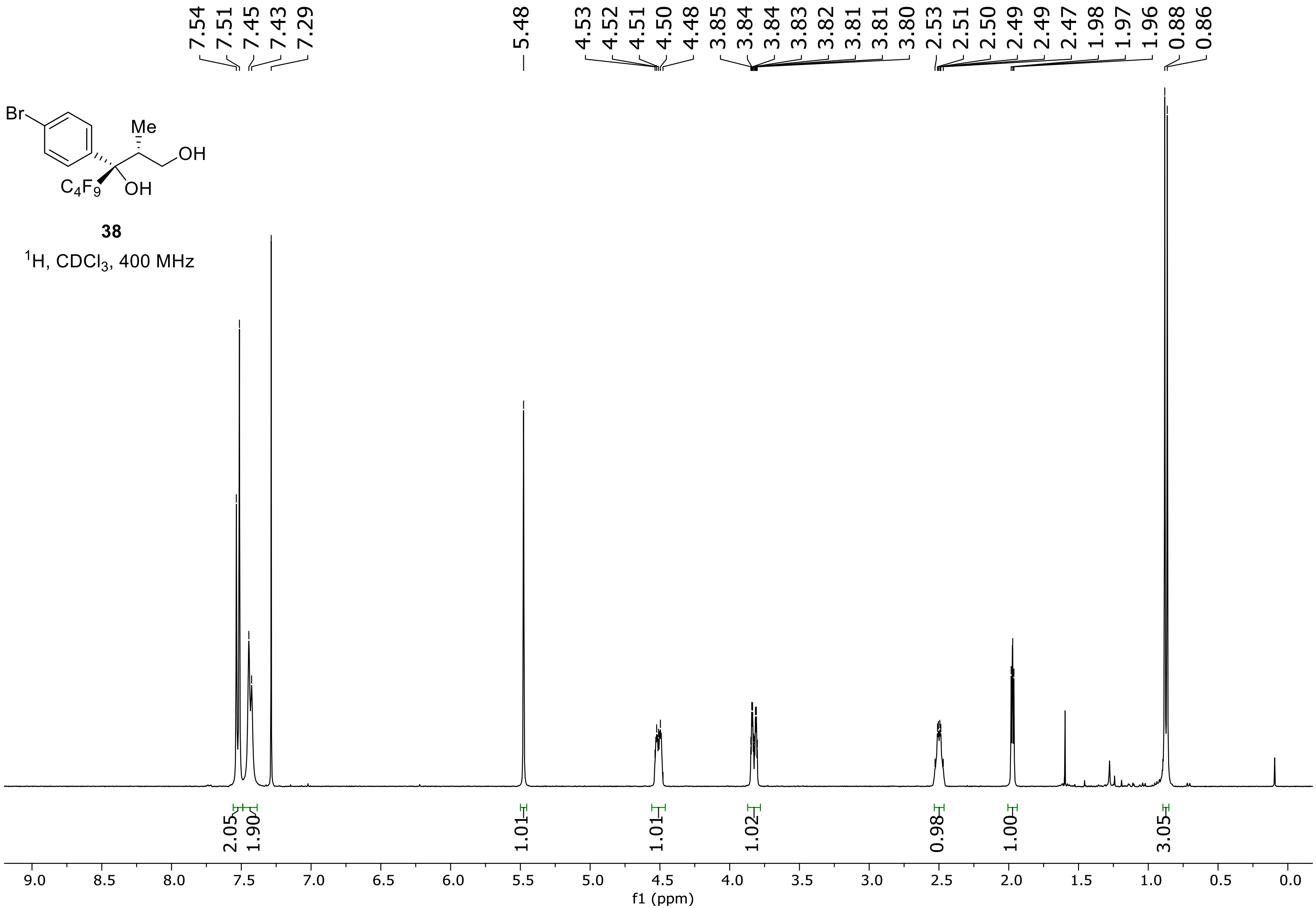
7.54
7.51
7.45
7.43
7.29
5.48
4.53
4.52
4.51
4.50
4.48
3.85
3.84
3.84
3.83
3.82
3.81
3.81
3.80
2.53
2.51
2.50
2.49
2.49
2.47
1.98
1.97
1.96
0.88
0.86
Br
Me
OH
$C_4F_9$
OH
38
$^1$H, $CDCl_3$, 400 MHz
2.05
1.90
1.01
1.01
1.02
0.98
1.00
3.05
9.0 8.5 8.0 7.5 7.0 6.5 6.0 5.5 5.0 4.5 4.0 3.5 3.0 2.5 2.0 1.5 1.0 0.5 0.0
f1 (ppm)

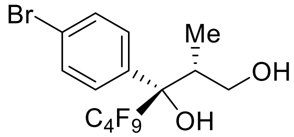

**38**

$^{19}F$, $CDCl_3$, 376 MHz

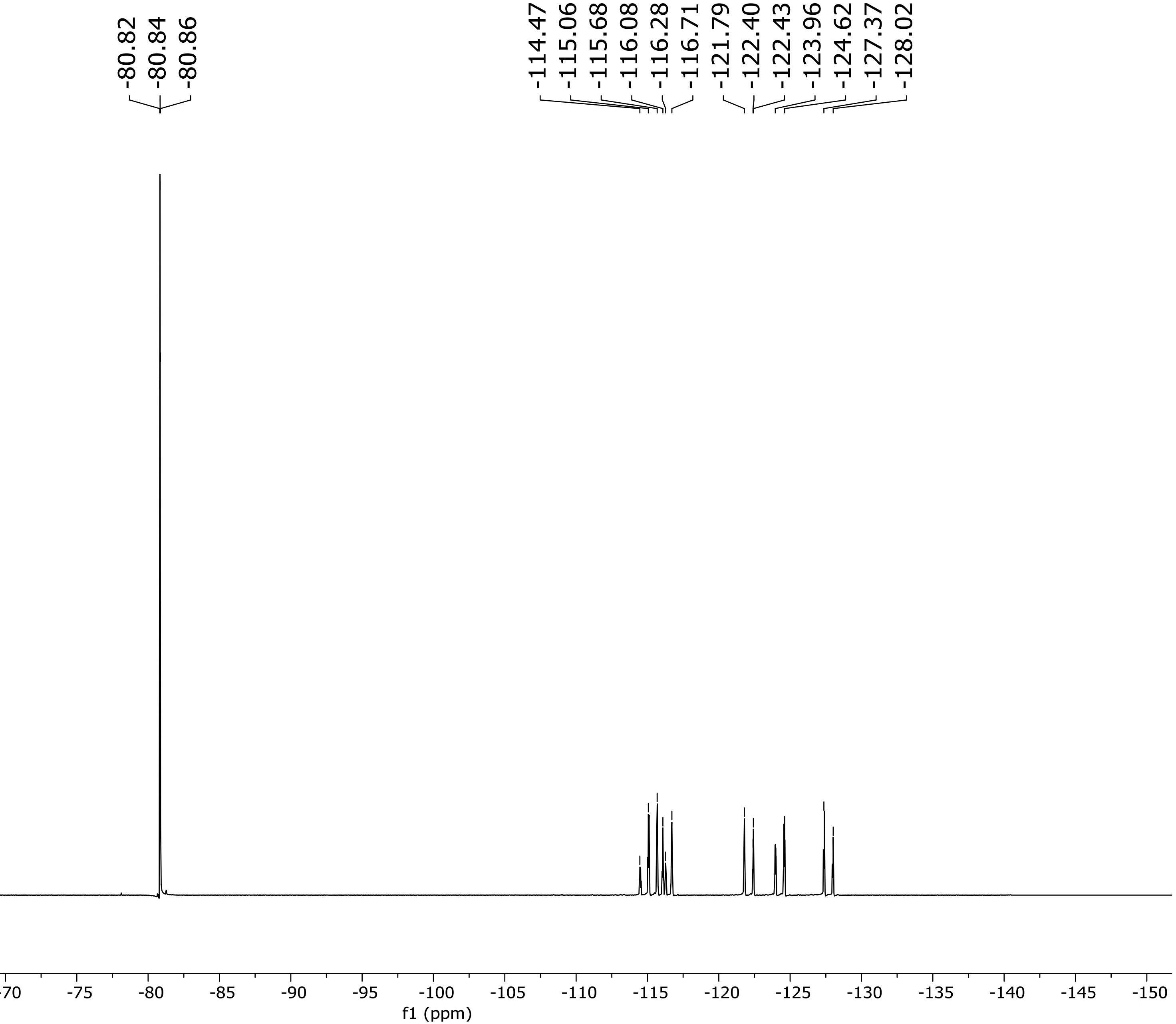

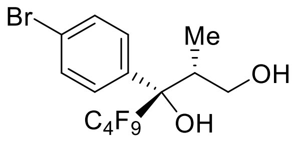

**38**

$^{13}C$, $CDCl_3$, 126 MHz

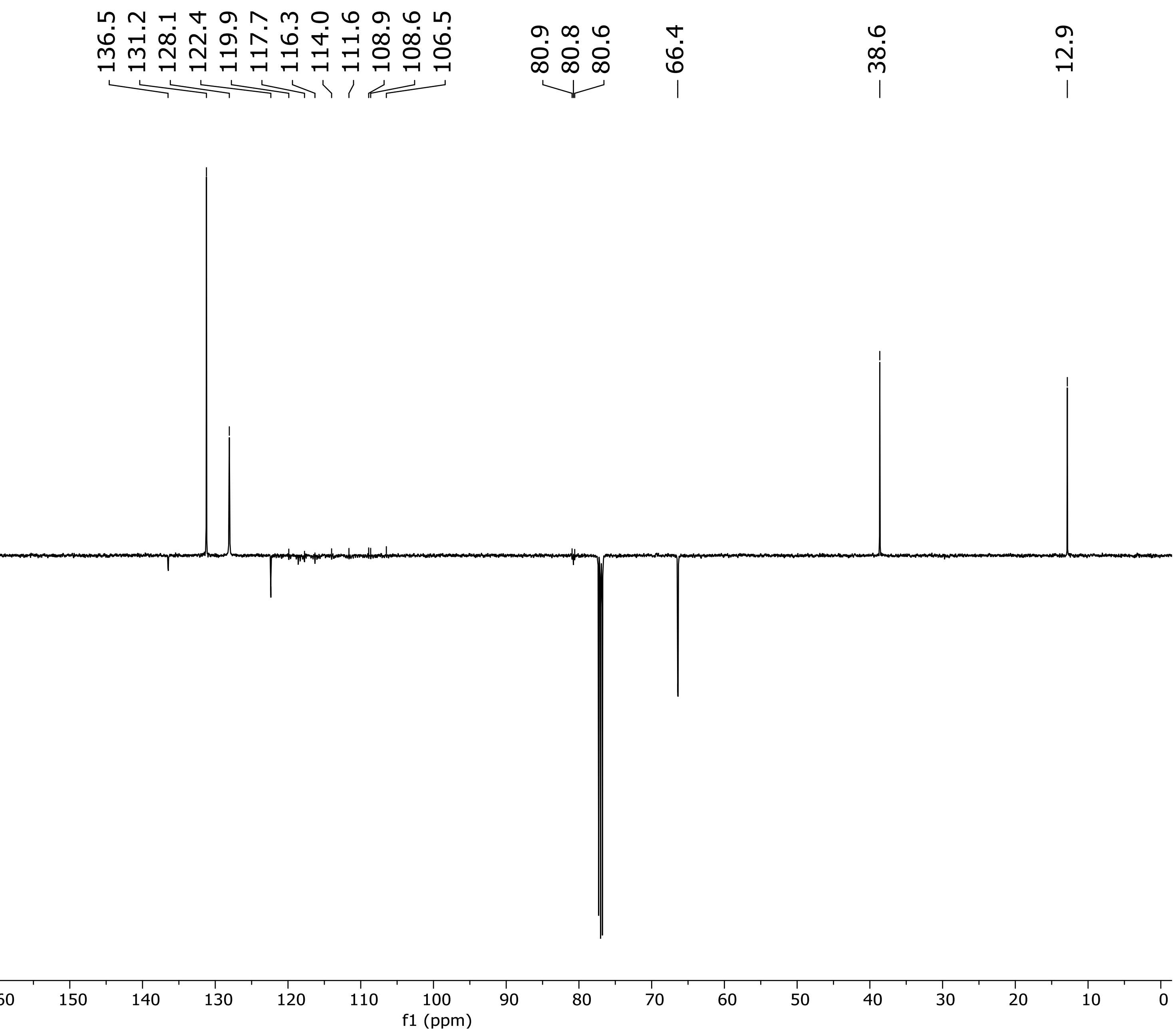

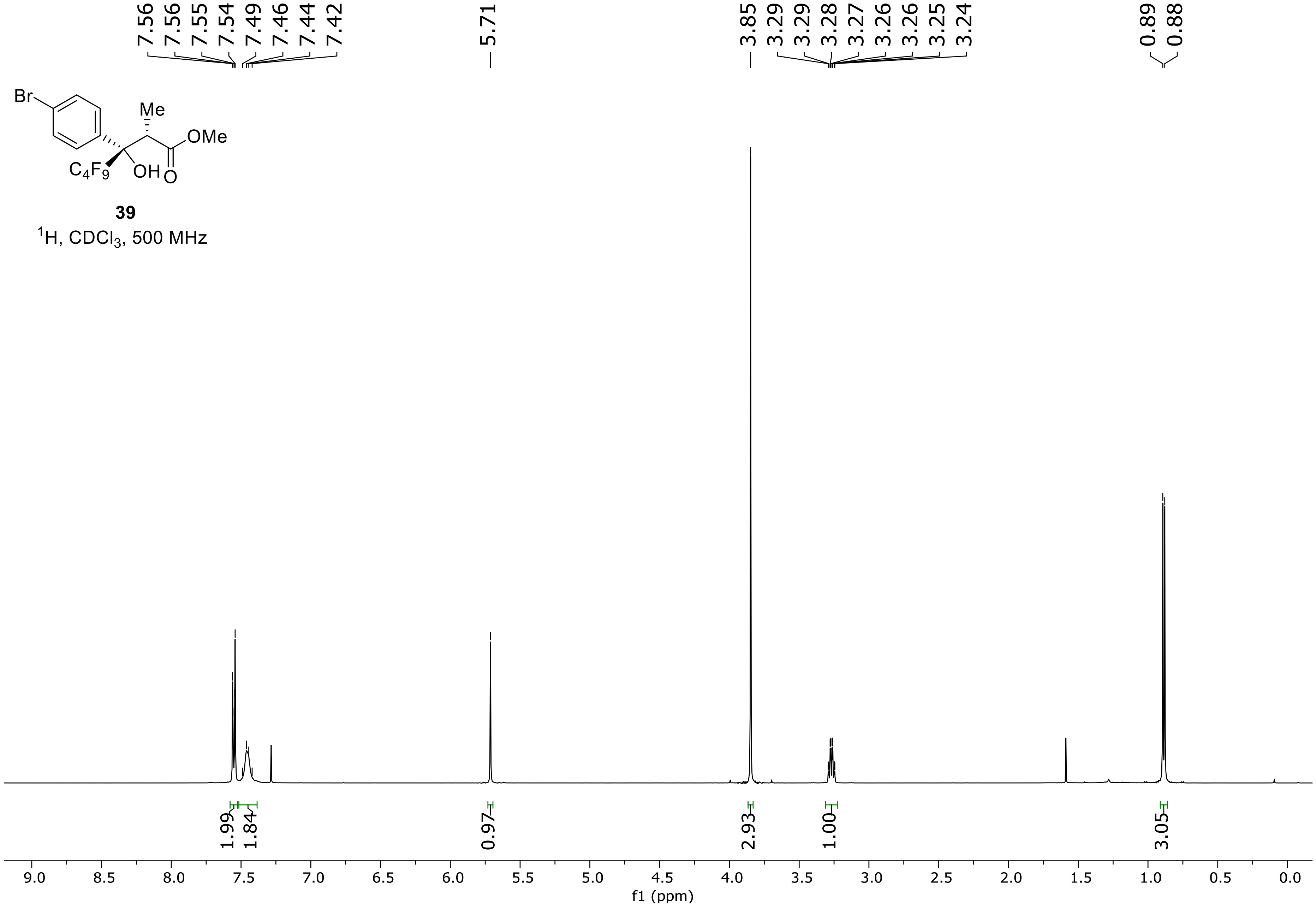


**39**

$^{1}H$, $CDCl_3$, 500 MHz

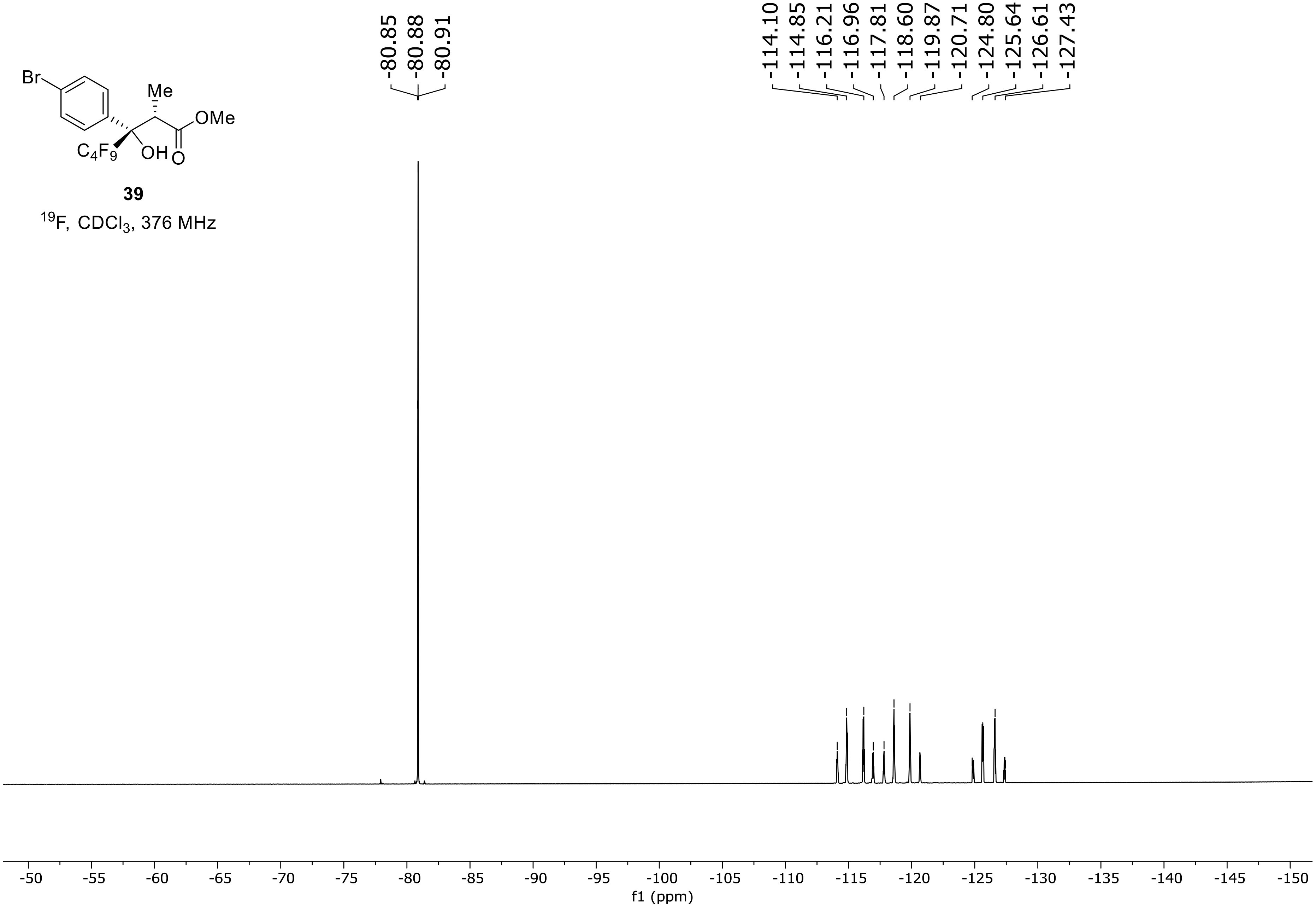
Br
Me
OMe
C4F9
OH
O
39
19F, CDCl3, 376 MHz
-80.85
-80.88
-80.91
-114.10
-114.85
-116.21
-116.96
-117.81
-118.60
-119.87
-120.71
-124.80
-125.64
-126.61
-127.43
-50
-55
-60
-65
-70
-75
-80
-85
-90
-95
-100
-105
-110
-115
-120
-125
-130
-135
-140
-145
-150
f1 (ppm)

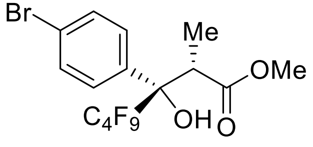

**39**

$^{13}C$, $CDCl_3$, 126 MHz

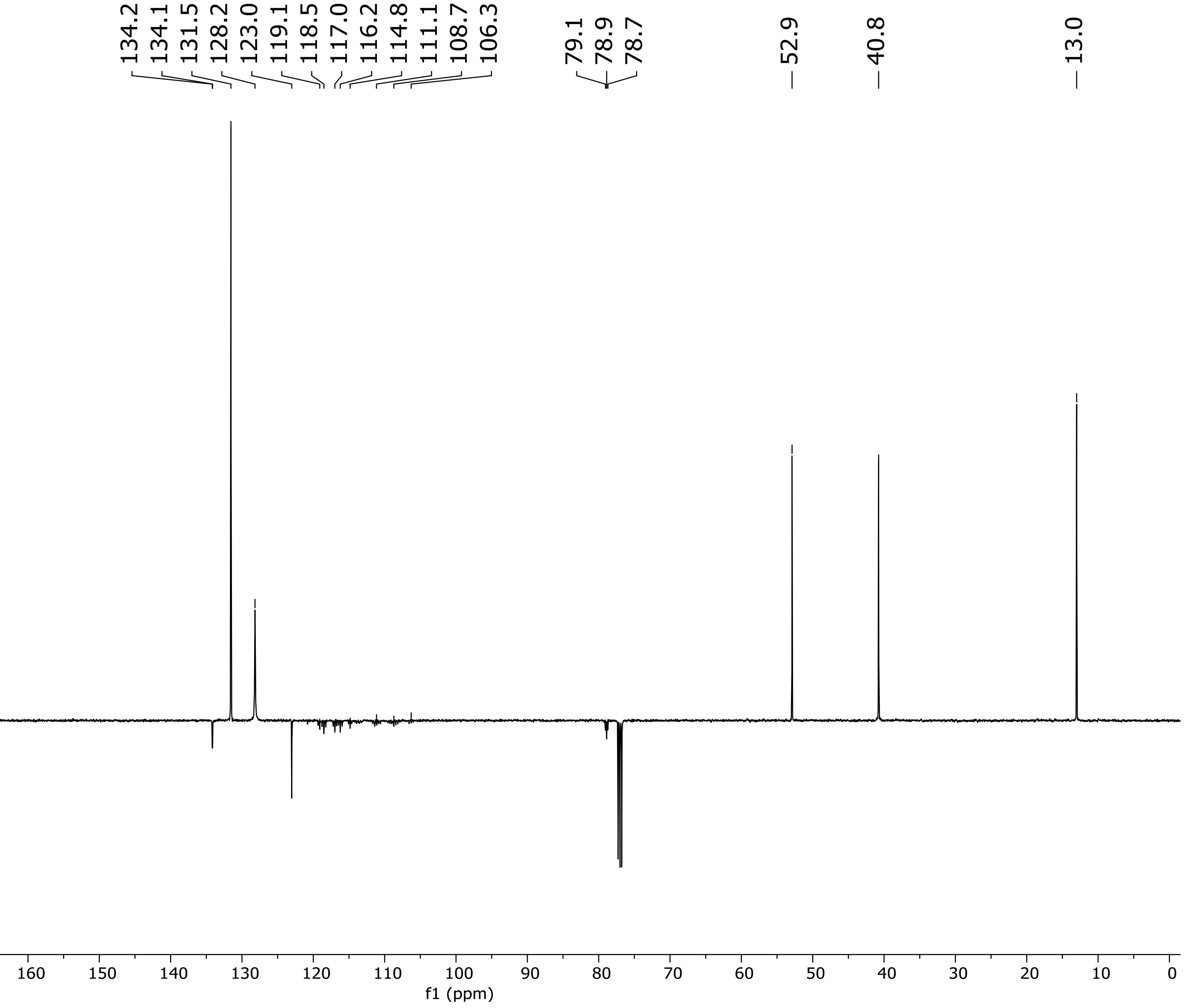

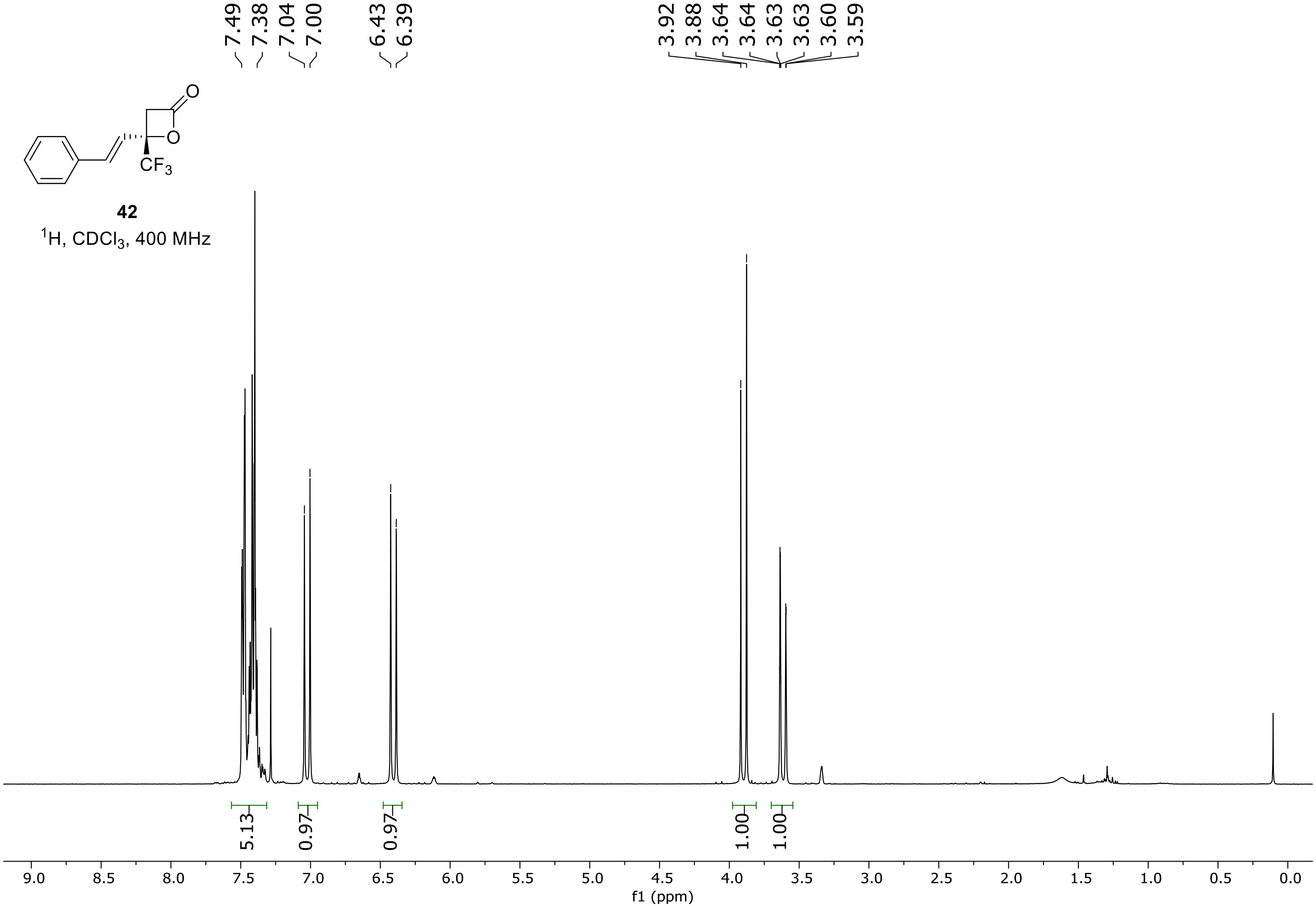

7.49
7.38
7.04
7.00
6.43
6.39
3.92
3.88
3.64
3.64
3.63
3.63
3.60
3.59
CF3
O
O
42
1H, CDCl3, 400 MHz
5.13
0.97
0.97
1.00
1.00
9.0 8.5 8.0 7.5 7.0 6.5 6.0 5.5 5.0 4.5 4.0 3.5 3.0 2.5 2.0 1.5 1.0 0.5 0.0
f1 (ppm)

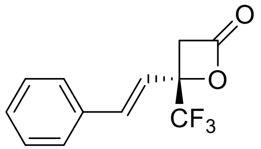

**42**

$^{19}F$, $CDCl_3$, 376 MHz

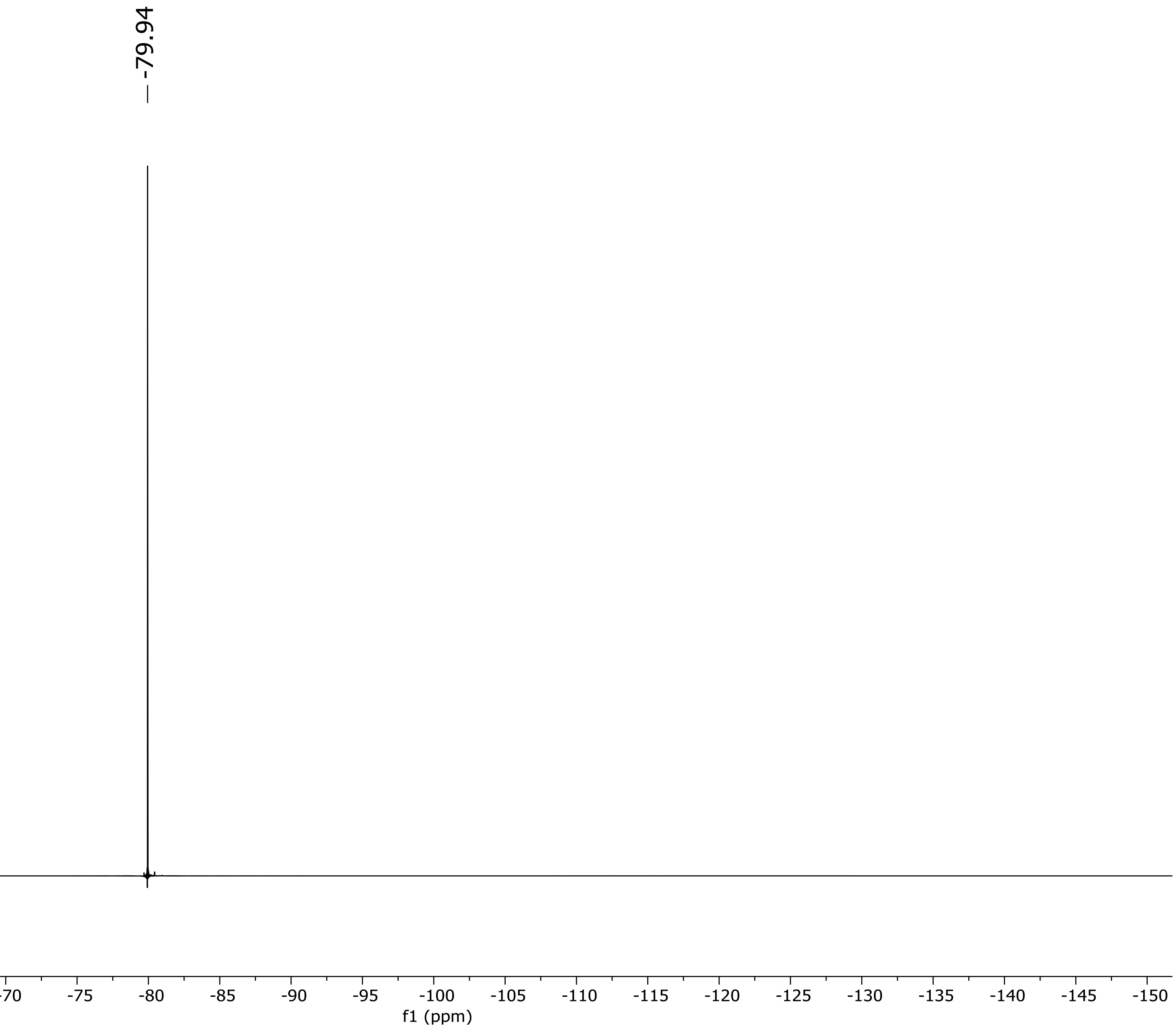

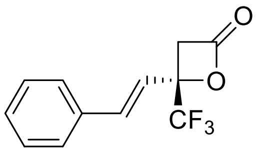

**42**

$^{13}C$, $CDCl_3$, 126 MHz

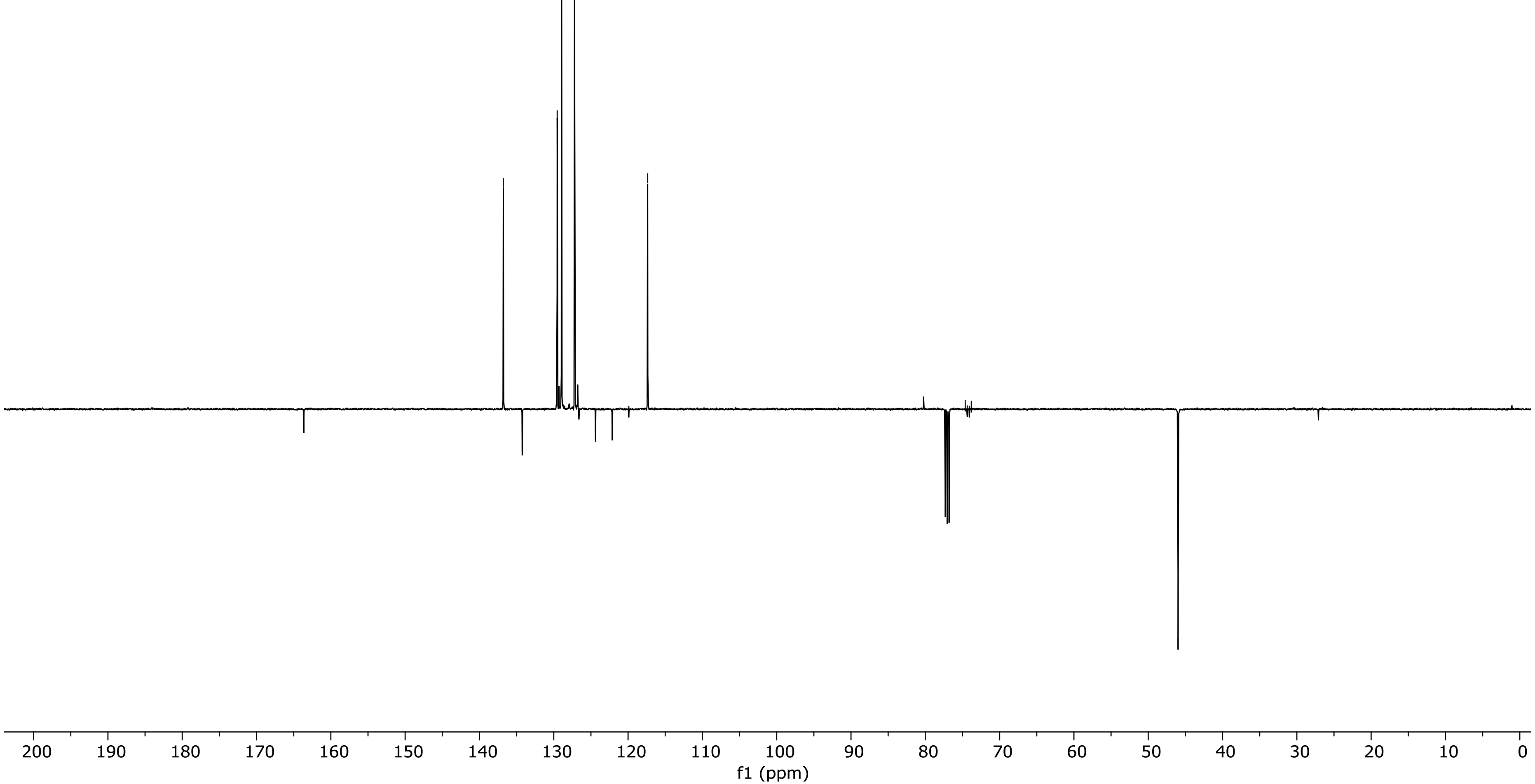

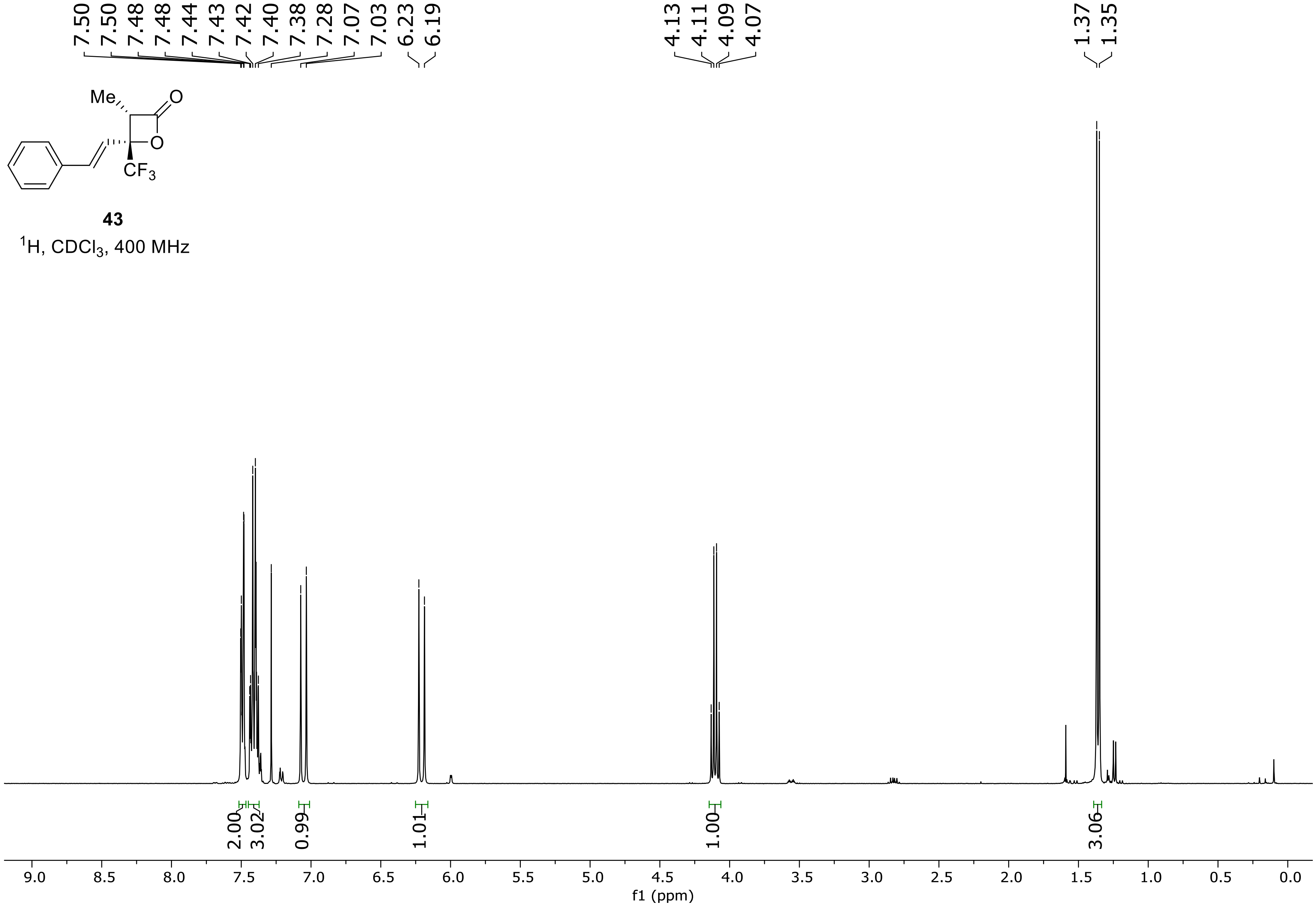
7.50
7.50
7.48
7.48
7.44
7.43
7.42
7.40
7.38
7.28
7.07
7.03
6.23
6.19
4.13
4.11
4.09
4.07
1.37
1.35
Me
O
O
$CF_3$
43
$^1$H, $CDCl_3$, 400 MHz
2.00
3.02
0.99
1.01
1.00
3.06
9.0
8.5
8.0
7.5
7.0
6.5
6.0
5.5
5.0
4.5
4.0
3.5
3.0
2.5
2.0
1.5
1.0
0.5
0.0
f1 (ppm)

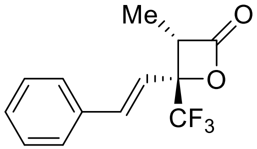


**43**

$^{19}F$, $CDCl_3$, 376 MHz

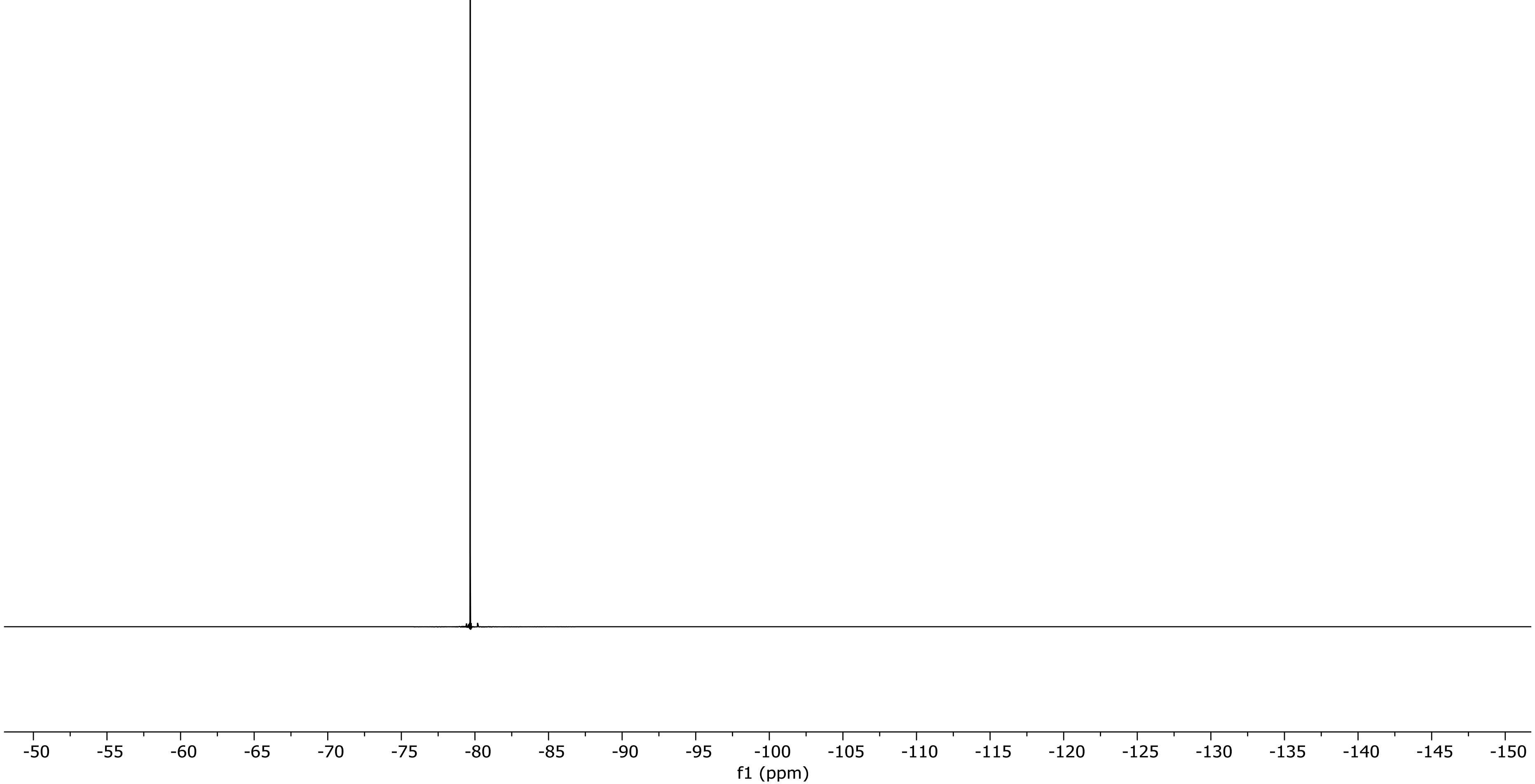

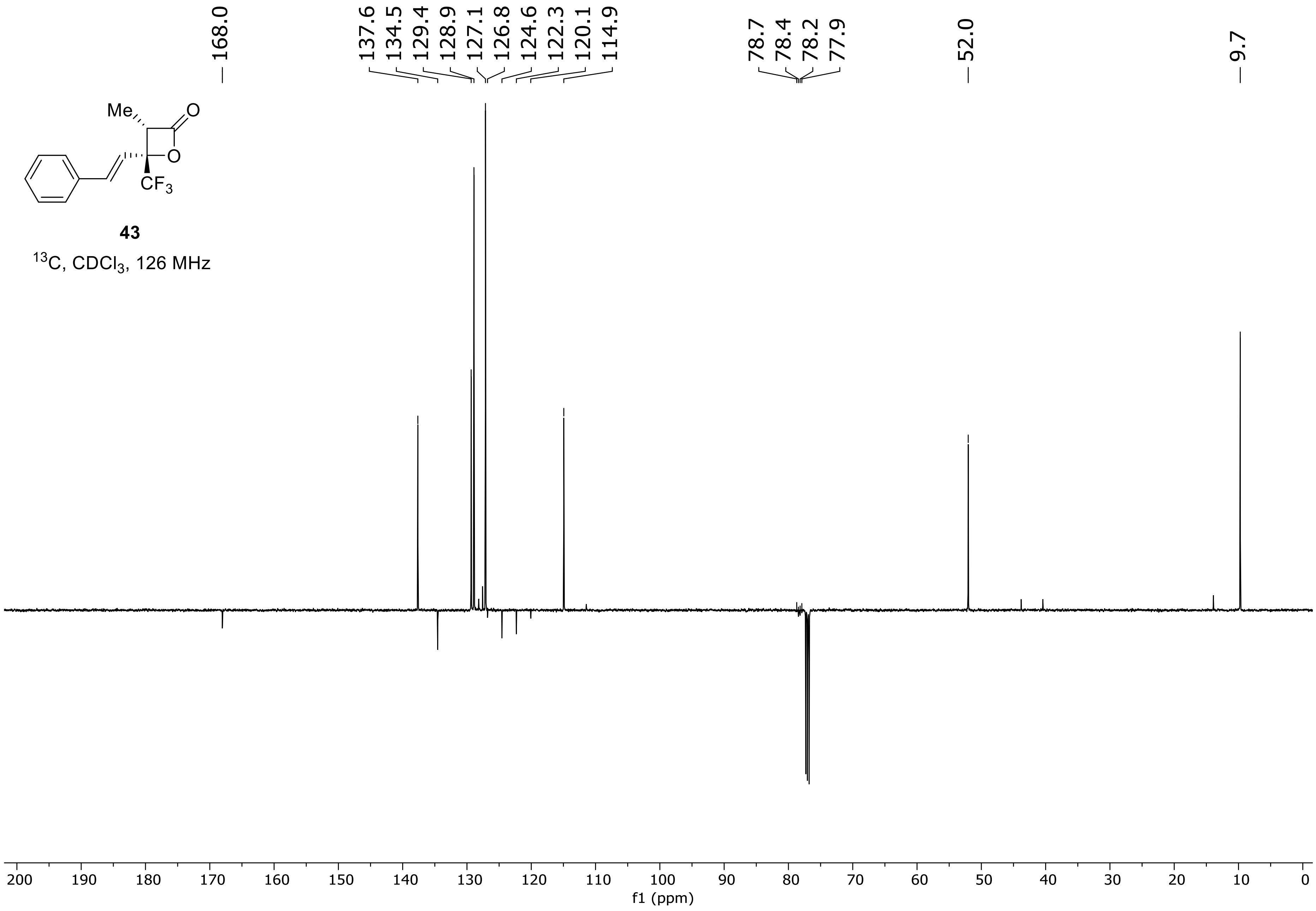
Me
O
O
CF3
43
13C, CDCl3, 126 MHz
168.0
137.6
134.5
129.4
128.9
127.1
126.8
124.6
122.3
120.1
114.9
78.7
78.4
78.2
77.9
52.0
9.7
200
190
180
170
160
150
140
130
120
110
100
90
80
70
60
50
40
30
20
10
0
f1 (ppm)

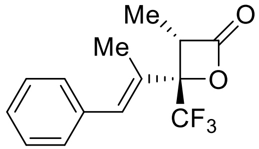

**44**

$^{1}H$, $CDCl_3$, 500 MHz

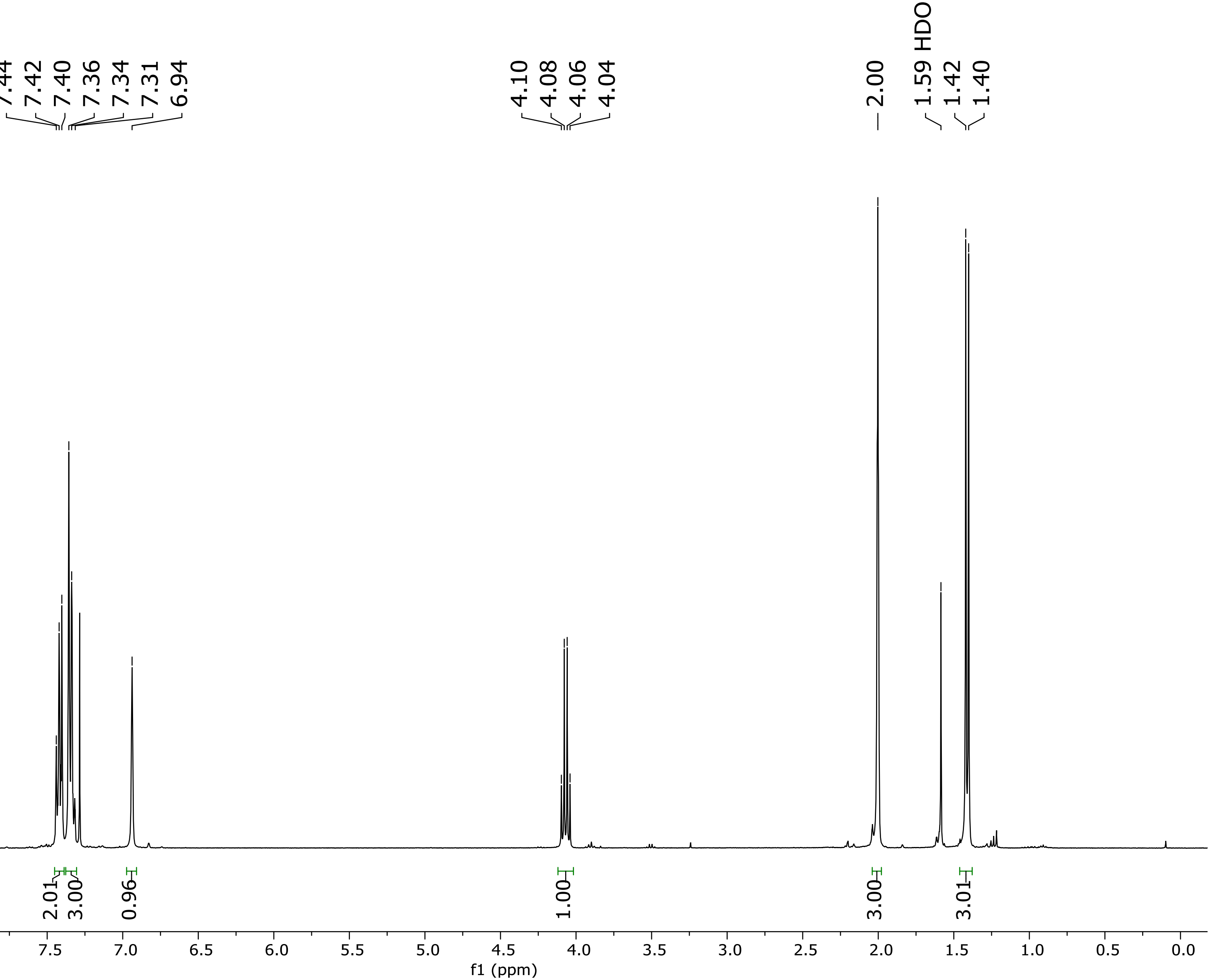

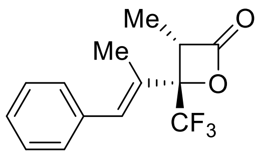

**44**

$^{19}F$, $CDCl_3$, 376 MHz

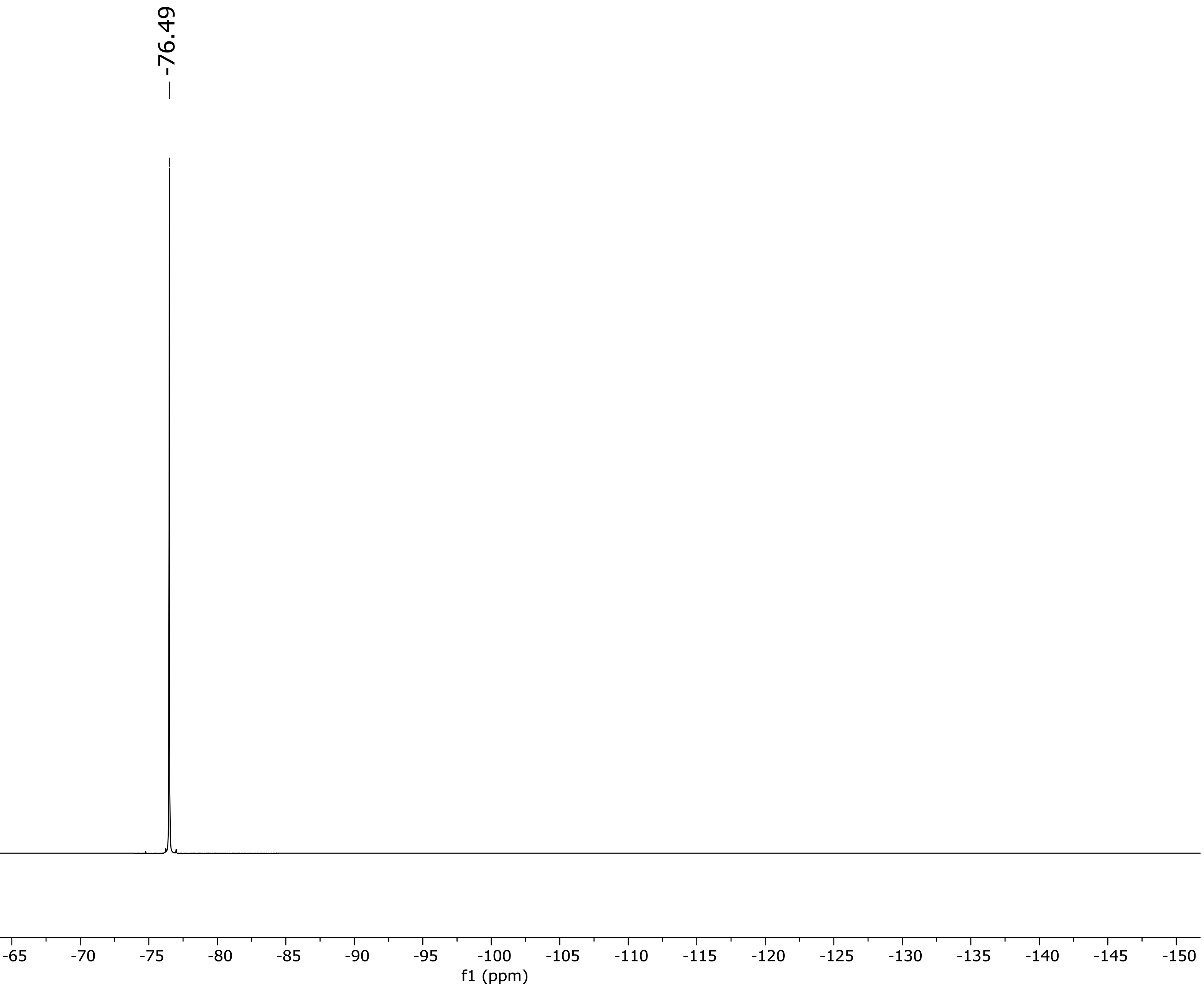

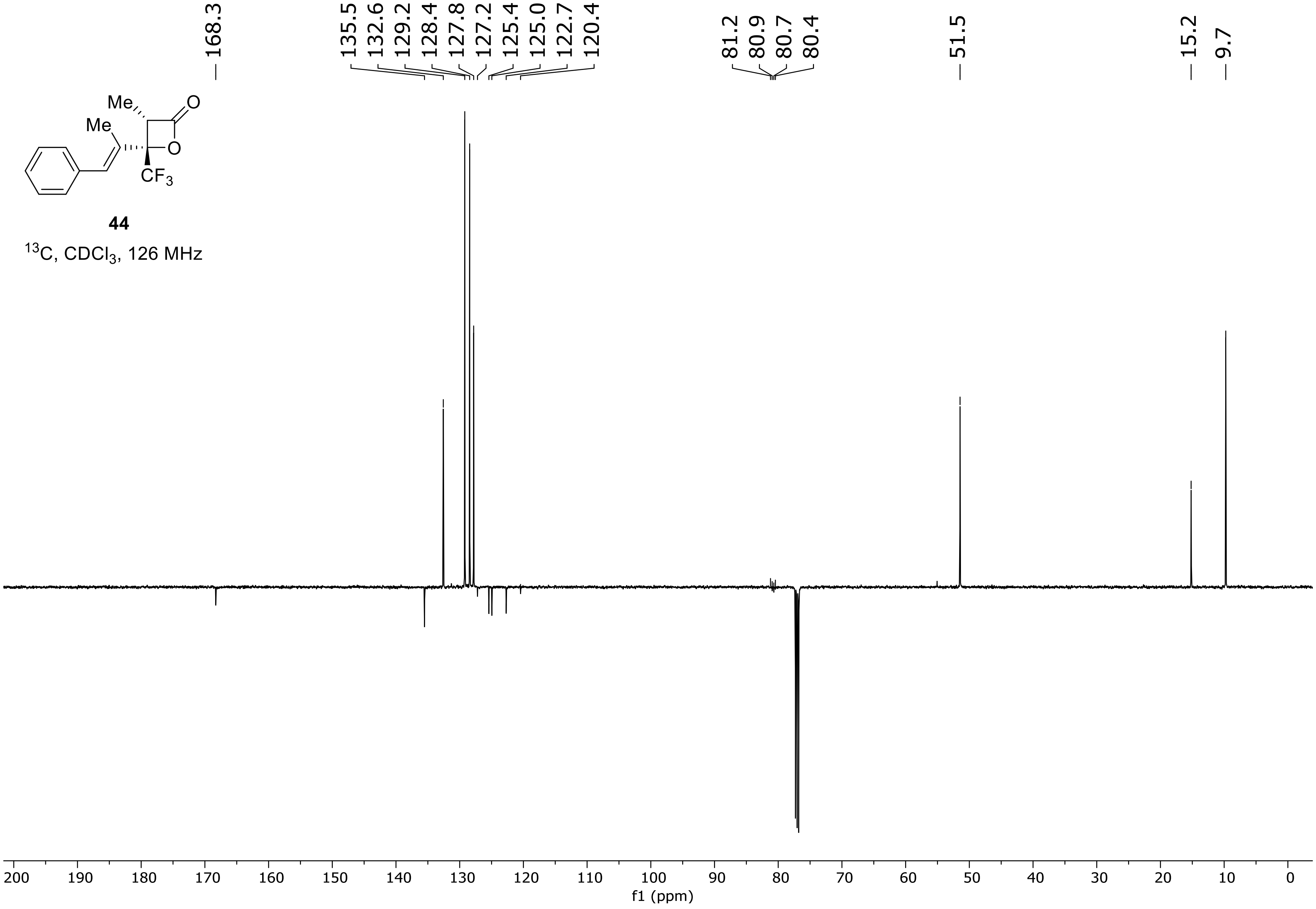
168.3
135.5
132.6
129.2
128.4
127.8
127.2
125.4
125.0
122.7
120.4
81.2
80.9
80.7
80.4
51.5
15.2
9.7
Me
Me
O
O
$CF_3$
44
$^{13}C$, $CDCl_3$, 126 MHz
200 190 180 170 160 150 140 130 120 110 100 90 80 70 60 50 40 30 20 10 0
f1 (ppm)

## Appendix II. HPLC Traces

HPLC Data for **S7**: Chiralpak IC (99.8:0.2 hexane:IPA, flow rate 1.0 mL min$^{-1}$, 211nm, 30 °C), $t_R$(major): 6.3 min, $t_R$(minor): 7.0 min, 90:10 er.

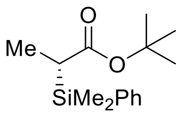


**S7**

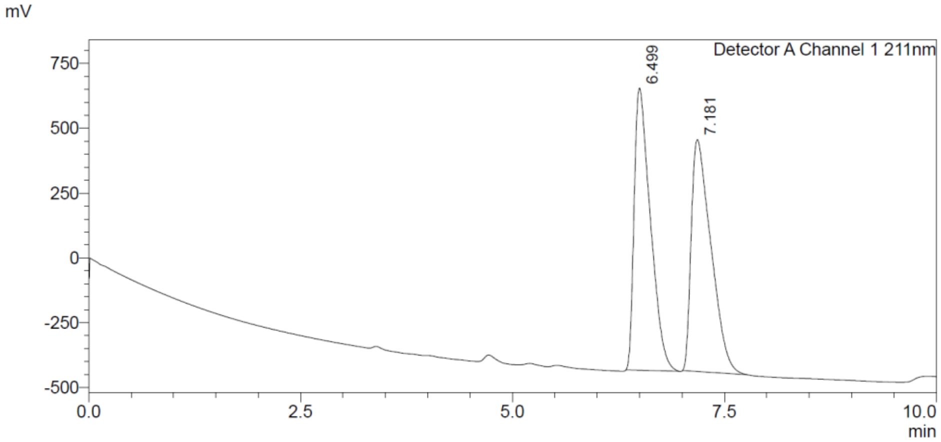


**<Peak Table>**

Detector A Channel 1 211nm

| Peak# | Ret. Time | Area% |
|---|---|---|
| 1 | 6.499 | 49.977 |
| 2 | 7.181 | 50.023 |
| Total | | 100.000 |

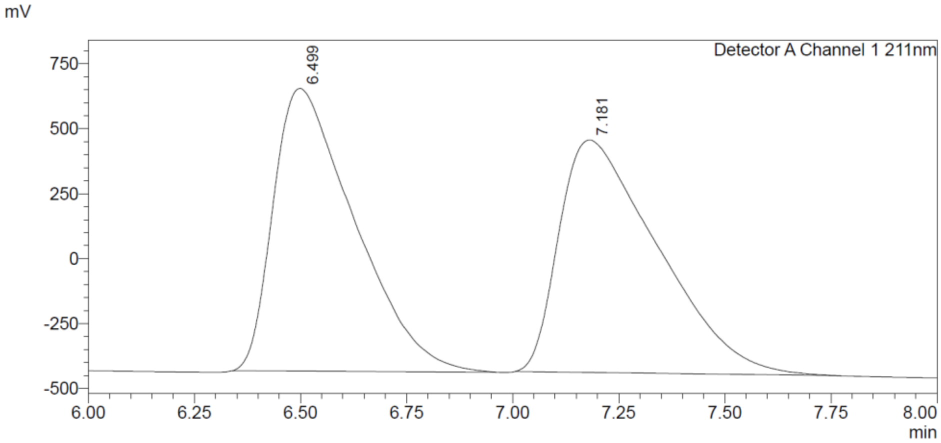


**<Peak Table>**

Detector A Channel 1 211nm

| Peak# | Ret. Time | Area% |
|---|---|---|
| 1 | 6.499 | 49.977 |
| 2 | 7.181 | 50.023 |
| Total | | 100.000 |

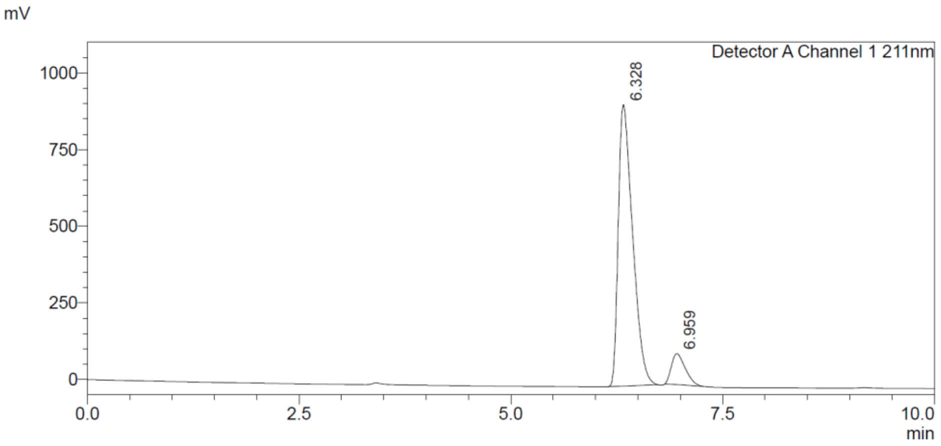


**<Peak Table>**

Detector A Channel 1 211nm

| Peak# | Ret. Time | Area% |
|---|---|---|
| 1 | 6.328 | 90.366 |
| 2 | 6.959 | 9.634 |
| Total | | 100.000 |

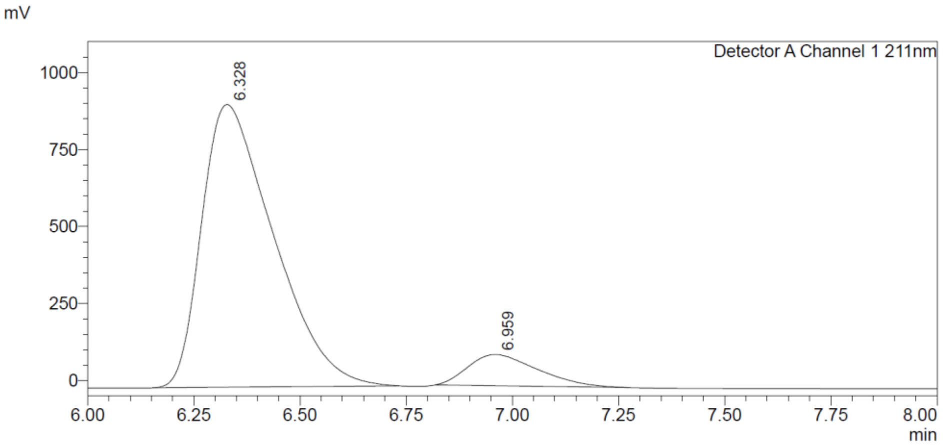


**<Peak Table>**

Detector A Channel 1 211nm

| Peak# | Ret. Time | Area% |
|---|---|---|
| 1 | 6.328 | 90.366 |
| 2 | 6.959 | 9.634 |
| Total | | 100.000 |

HPLC Data for **S8**: Chiralcel OJ-H (97:3 hexane:IPA, flow rate 0.6 mL min$^{-1}$, 211nm, 30 °C), $t_R$(minor): 18.6 min, $t_R$(major): 22.7 min, 89:11 er.

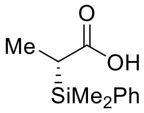


(***R***)**-S8**

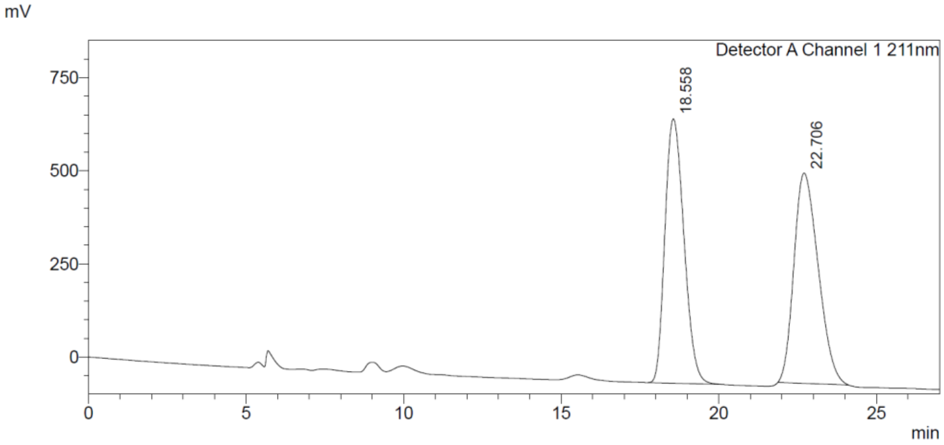


**<Peak Table>**

Detector A Channel 1 211nm

| Peak# | Ret. Time | Area% |
|---|---|---|
| 1 | 18.558 | 49.528 |
| 2 | 22.706 | 50.472 |
| Total | | 100.000 |

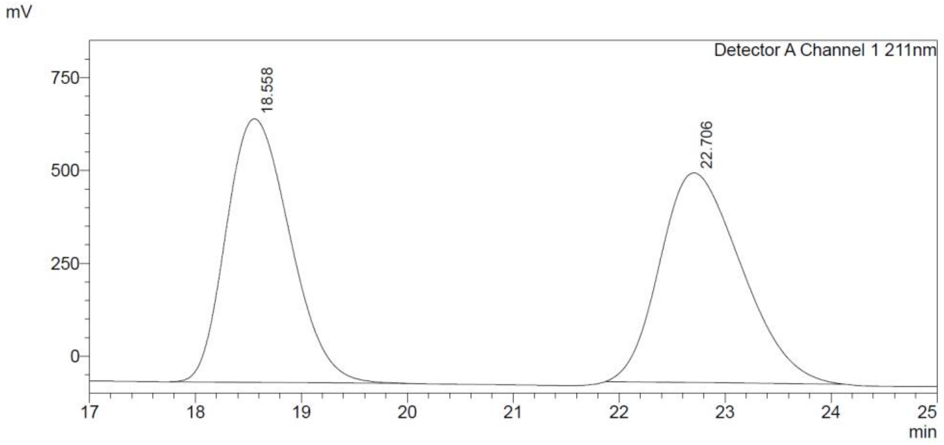


**<Peak Table>**

Detector A Channel 1 211nm

| Peak# | Ret. Time | Area% |
|---|---|---|
| 1 | 18.558 | 49.528 |
| 2 | 22.706 | 50.472 |
| Total | | 100.000 |

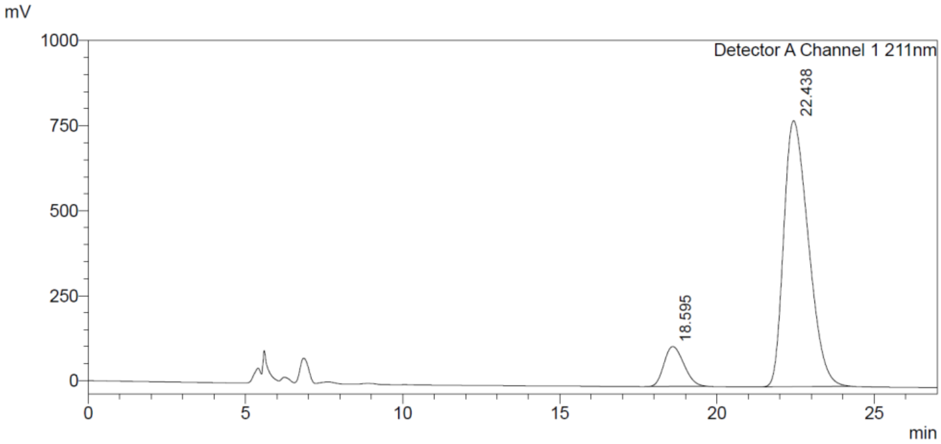


**<Peak Table>**

Detector A Channel 1 211nm

| Peak# | Ret. Time | Area% |
|---|---|---|
| 1 | 18.595 | 10.747 |
| 2 | 22.438 | 89.253 |
| Total | | 100.000 |

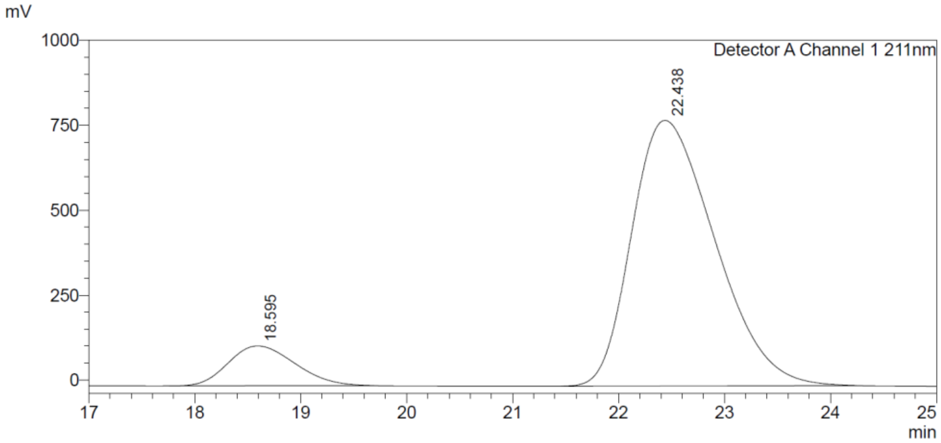


**<Peak Table>**

Detector A Channel 1 211nm

| Peak# | Ret. Time | Area% |
|---|---|---|
| 1 | 18.595 | 10.747 |
| 2 | 22.438 | 89.253 |
| Total | | 100.000 |

HPLC Data for **46**: Chiralcel OD-H (99.9:0.1 hexane:IPA, flow rate 1.0 mL min$^{-1}$, 211 nm, 30 °C), $t_R$(major): 8.4 min, $t_R$(minor): 11.4 min, 89:11 er.

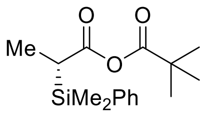


**46**

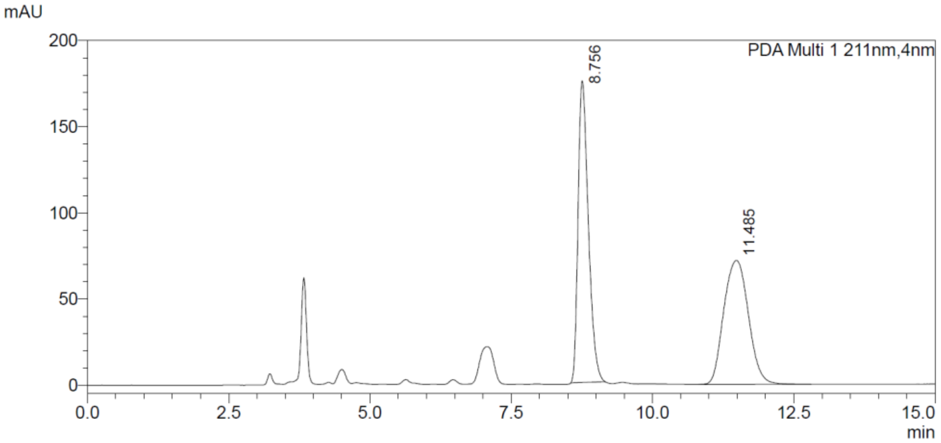


**<Peak Table>**

PDA Ch1 211nm

| Peak# | Ret. Time | Area% |
|---|---|---|
| 1 | 8.756 | 50.307 |
| 2 | 11.485 | 49.693 |
| Total | | 100.000 |

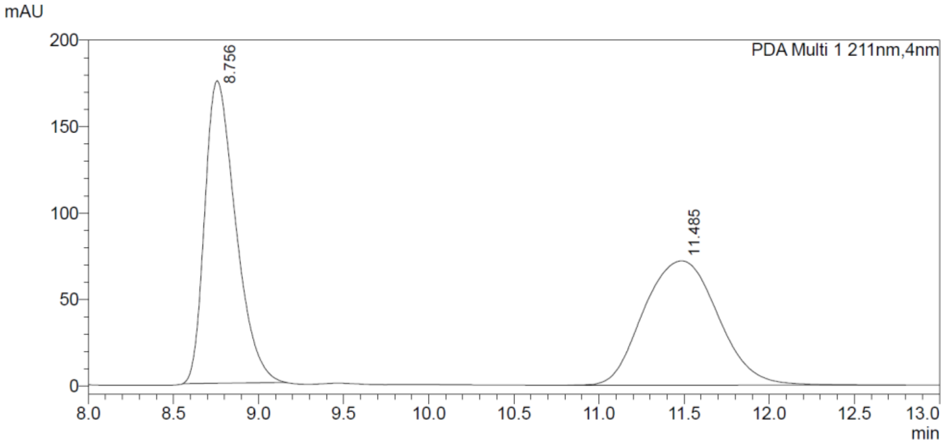


**<Peak Table>**

PDA Ch1 211nm

| Peak# | Ret. Time | Area% |
|---|---|---|
| 1 | 8.756 | 50.307 |
| 2 | 11.485 | 49.693 |
| Total | | 100.000 |

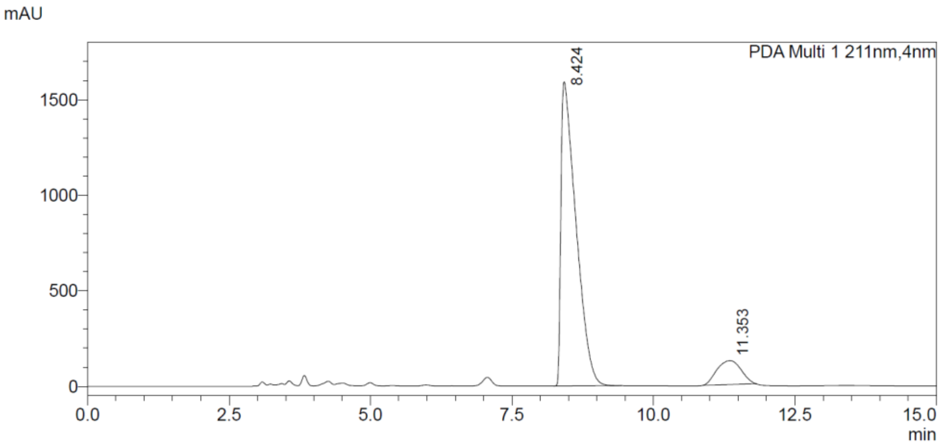


**<Peak Table>**

PDA Ch1 211nm

| Peak# | Ret. Time | Area% |
|---|---|---|
| 1 | 8.424 | 88.719 |
| 2 | 11.353 | 11.281 |
| Total | | 100.000 |

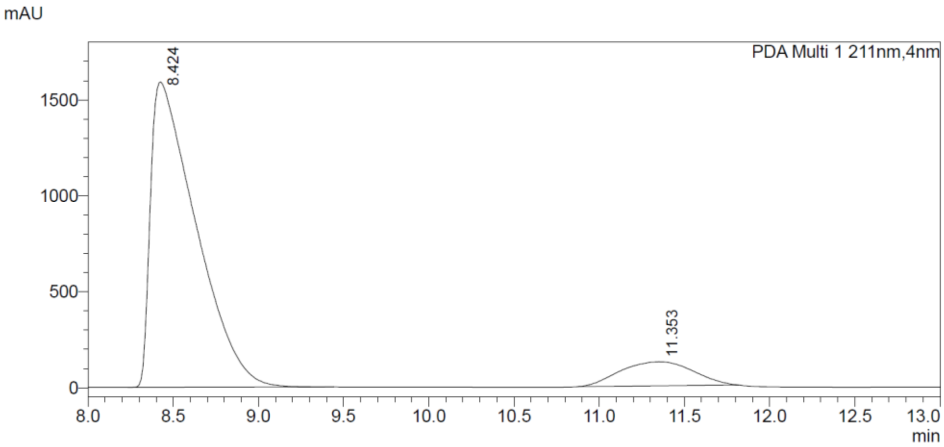


**<Peak Table>**

PDA Ch1 211nm

| Peak# | Ret. Time | Area% |
|---|---|---|
| 1 | 8.424 | 88.719 |
| 2 | 11.353 | 11.281 |
| Total | | 100.000 |

HPLC Data for **3**: Chiralpak IB (99.3:0.7 hexane:IPA, flow rate 1.0 mLmin$^{-1}$, 254 nm, 30 °C), $t_R$ (major): 9.3 min, $t_R$ (minor):10.3 min, 94:6 er.

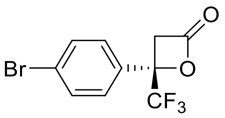


**3**

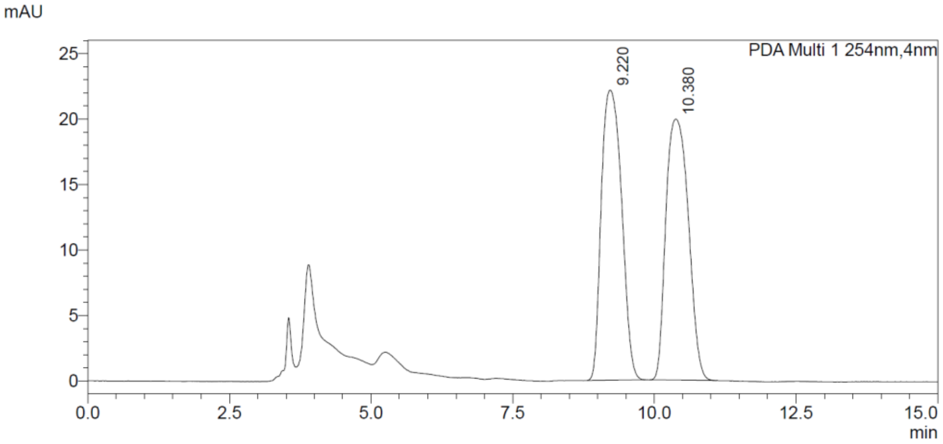


**<Peak Table>**

PDA Ch1 254nm

| Peak# | Ret. Time | Area% |
|---|---|---|
| 1 | 9.220 | 50.002 |
| 2 | 10.380 | 49.998 |
| Total | | 100.000 |

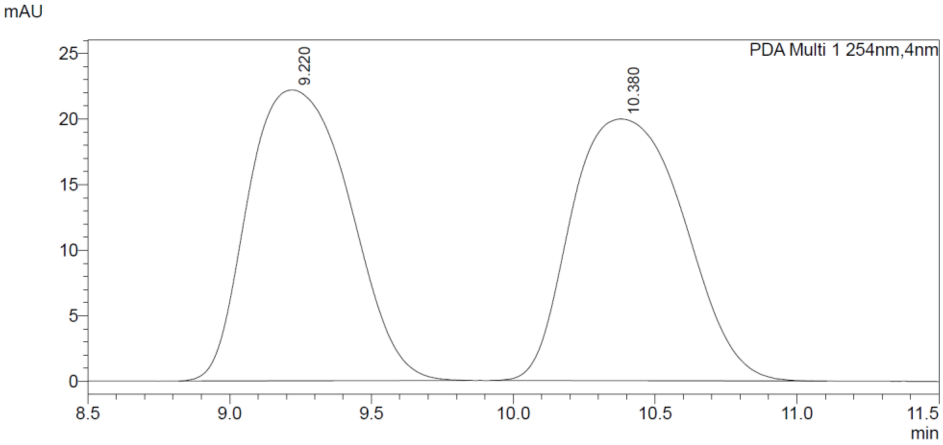


**<Peak Table>**

PDA Ch1 254nm

| Peak# | Ret. Time | Area% |
|---|---|---|
| 1 | 9.220 | 50.002 |
| 2 | 10.380 | 49.998 |
| Total | | 100.000 |

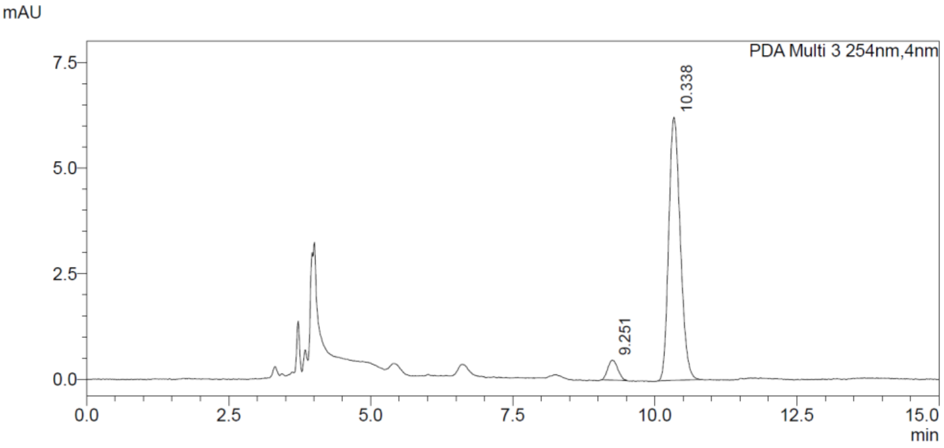


## <Peak Table>

PDA Ch3 254nm

| Peak# | Ret. Time | Area% |
|---|---|---|
| 1 | 9.251 | 6.136 |
| 2 | 10.338 | 93.864 |
| Total | | 100.000 |

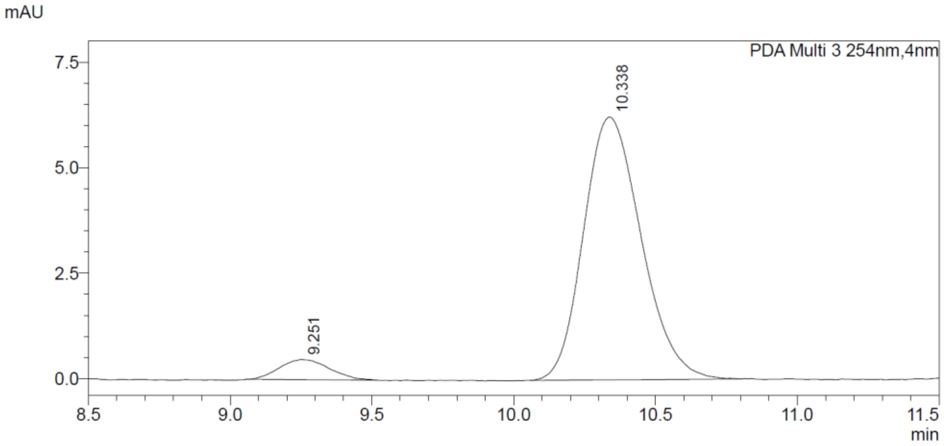


## <Peak Table>

PDA Ch3 254nm

| Peak# | Ret. Time | Area% |
|---|---|---|
| 1 | 9.251 | 6.136 |
| 2 | 10.338 | 93.864 |
| Total | | 100.000 |

HPLC Data for **7**: Chiralpak AS-H (99.5:0.5 hexane:IPA, flow rate 1.0 mLmin$^{-1}$, 211 nm, 30 °C), $t_R$ (minor): 4.6 min, $t_R$ (major):5.7 min, 97:3 er.

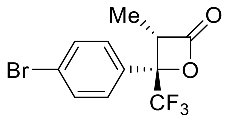


**7**

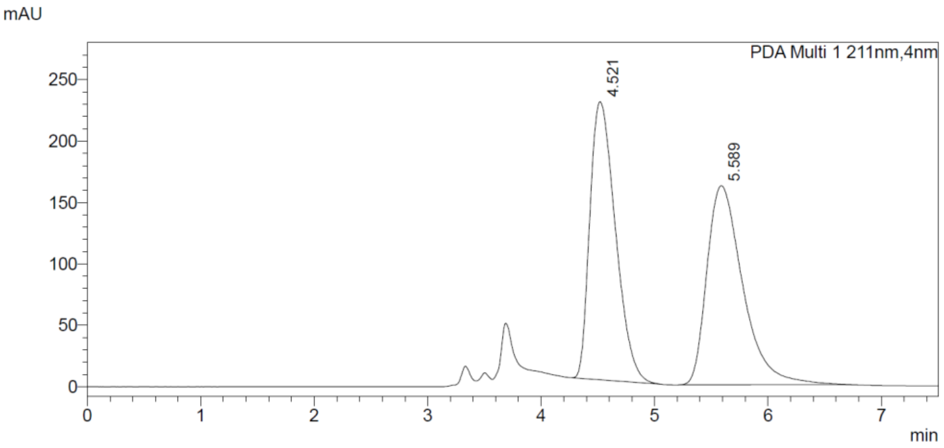


**<Peak Table>**

PDA Ch1 211nm

| Peak# | Ret. Time | Area% |
|---|---|---|
| 1 | 4.521 | 49.787 |
| 2 | 5.589 | 50.213 |
| Total | | 100.000 |

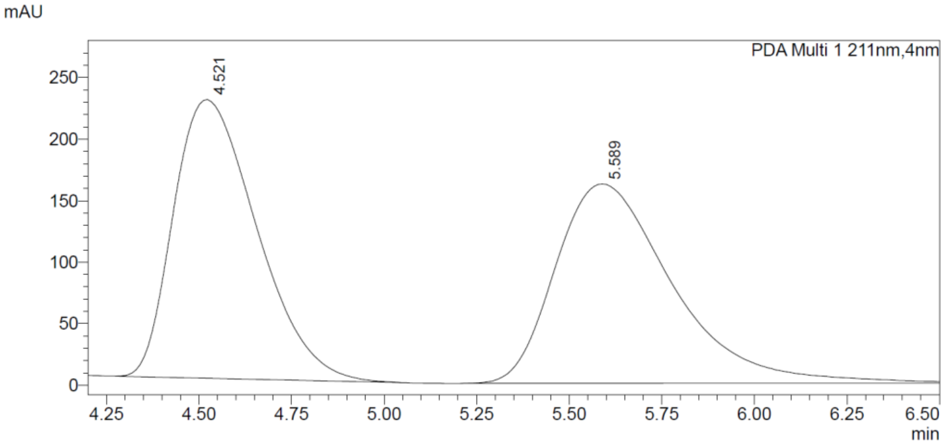


**<Peak Table>**

PDA Ch1 211nm

| Peak# | Ret. Time | Area% |
|---|---|---|
| 1 | 4.521 | 49.787 |
| 2 | 5.589 | 50.213 |
| Total | | 100.000 |

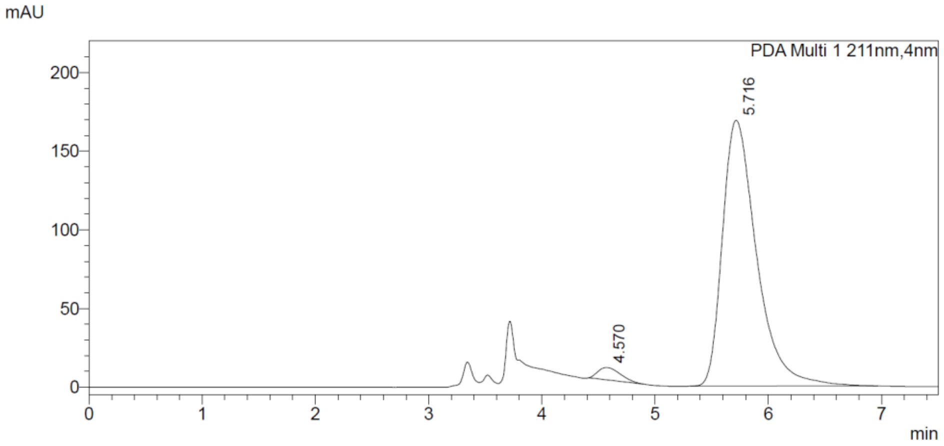


**<Peak Table>**

PDA Ch1 211nm

| Peak# | Ret. Time | Area% |
|---|---|---|
| 1 | 4.570 | 2.962 |
| 2 | 5.716 | 97.038 |
| Total | | 100.000 |

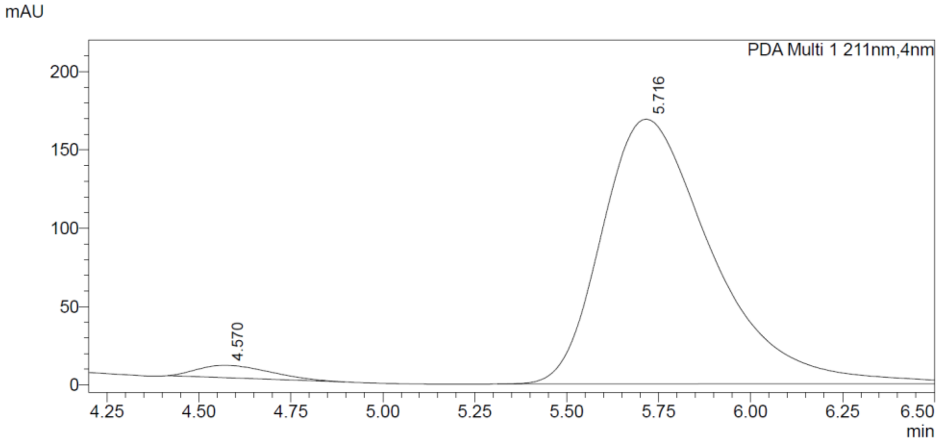


**<Peak Table>**

PDA Ch1 211nm

| Peak# | Ret. Time | Area% |
|---|---|---|
| 1 | 4.570 | 2.962 |
| 2 | 5.716 | 97.038 |
| Total | | 100.000 |

HPLC Data for **8**: Chiralpak IB (99.9:0.1 hexane:IPA, flow rate 1.0 mLmin$^{-1}$, 211 nm, 30 °C), $t_R$ (minor): 5.4 min, $t_R$ (major): 5.9 min, 96:4 er.

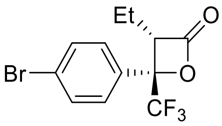


**8**

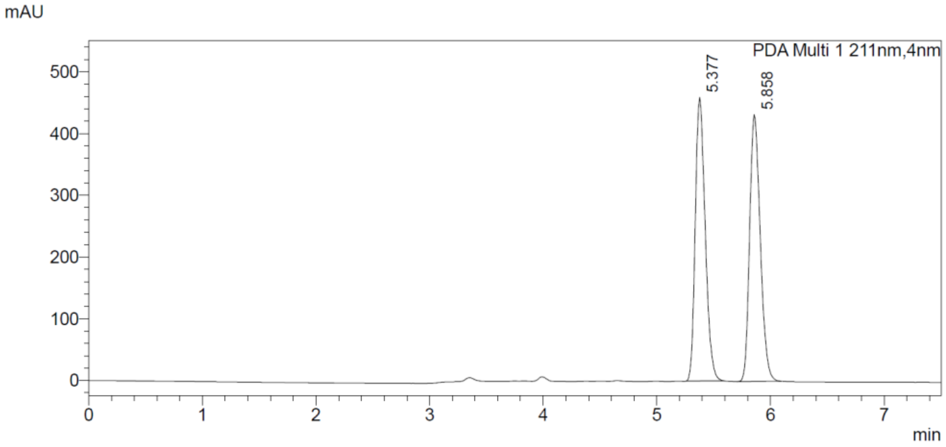


**<Peak Table>**

PDA Ch1 211nm

| Peak# | Ret. Time | Area% |
|---|---|---|
| 1 | 5.377 | 49.792 |
| 2 | 5.858 | 50.208 |
| Total | | 100.000 |

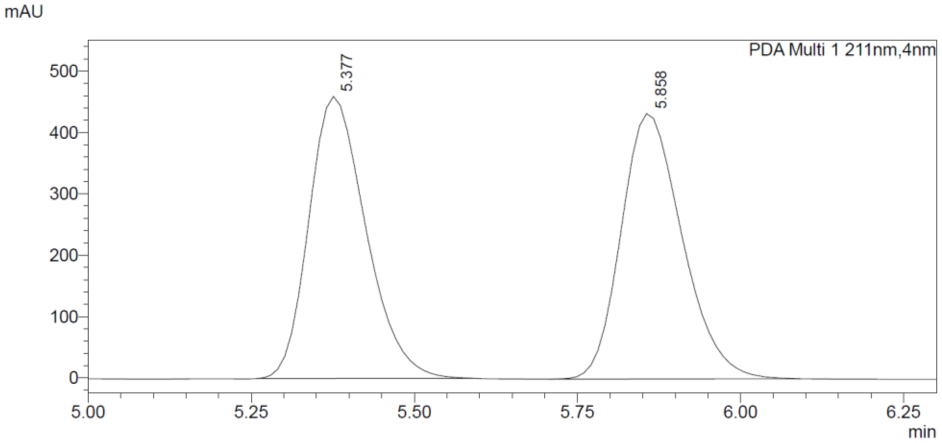


**<Peak Table>**

PDA Ch1 211nm

| Peak# | Ret. Time | Area% |
|---|---|---|
| 1 | 5.377 | 49.792 |
| 2 | 5.858 | 50.208 |
| Total | | 100.000 |

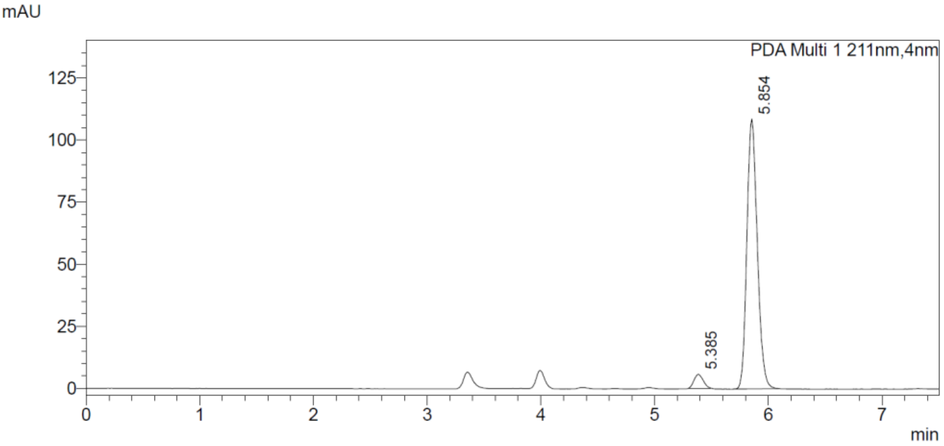


**<Peak Table>**

PDA Ch1 211nm

| Peak# | Ret. Time | Area% |
|---|---|---|
| 1 | 5.385 | 4.171 |
| 2 | 5.854 | 95.829 |
| Total | | 100.000 |

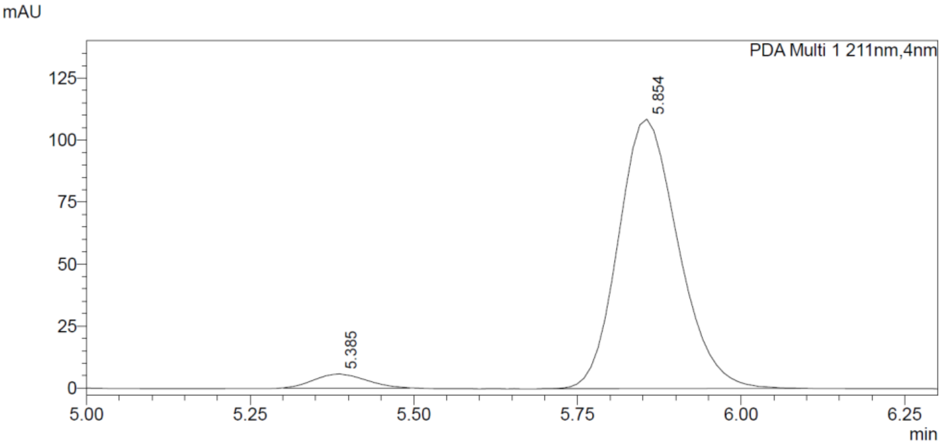


**<Peak Table>**

PDA Ch1 211nm

| Peak# | Ret. Time | Area% |
|---|---|---|
| 1 | 5.385 | 4.171 |
| 2 | 5.854 | 95.829 |
| Total | | 100.000 |

HPLC Data for **9**: **major diastereoisomer**: Chiralcel OJ-H (99.8:0.2 hexane:IPA, flow rate 1.0 mLmin$^{-1}$, 211 nm, 30 °C), $t_R$ (major): 5.9 min, $t_R$ (minor): 6.5 min, >99:1 er.

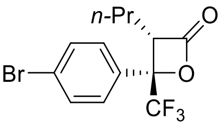


**9**

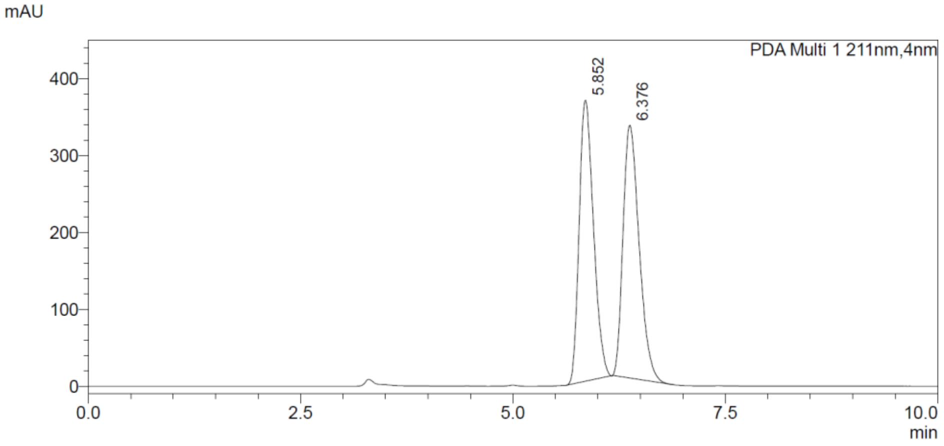


**<Peak Table>**

PDA Ch1 211nm

| Peak# | Ret. Time | Area% |
|---|---|---|
| 1 | 5.852 | 49.716 |
| 2 | 6.376 | 50.284 |
| Total | | 100.000 |

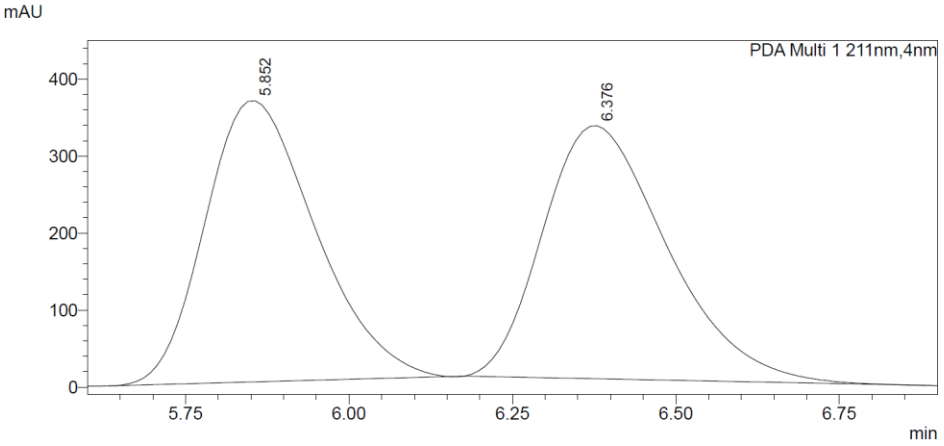


**<Peak Table>**

PDA Ch1 211nm

| Peak# | Ret. Time | Area% |
|---|---|---|
| 1 | 5.852 | 49.716 |
| 2 | 6.376 | 50.284 |
| Total | | 100.000 |

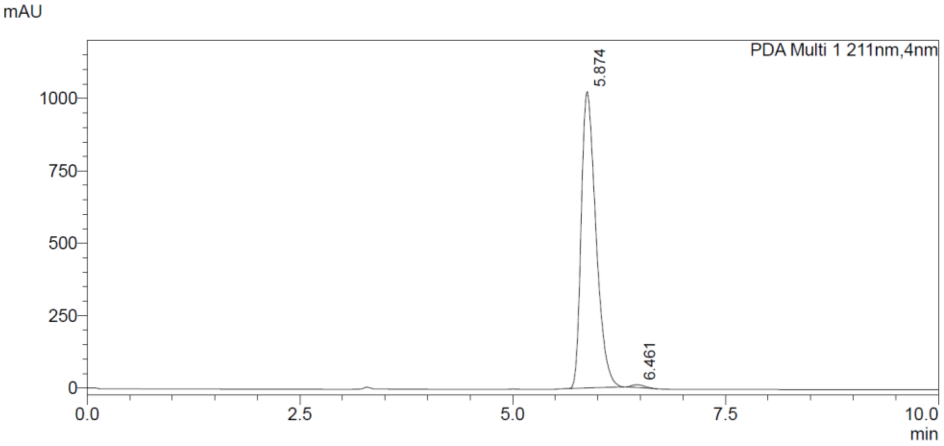


**<Peak Table>**

PDA Ch1 211nm

| Peak# | Ret. Time | Area% |
|---|---|---|
| 1 | 5.874 | 99.146 |
| 2 | 6.461 | 0.854 |
| Total | | 100.000 |

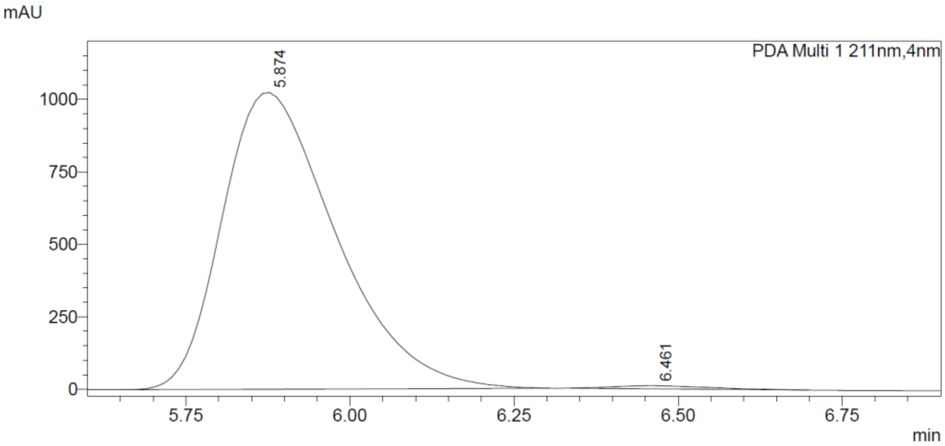



**<Peak Table>**

PDA Ch1 211nm

| Peak# | Ret. Time | Area% |
|---|---|---|
| 1 | 5.874 | 99.146 |
| 2 | 6.461 | 0.854 |
| Total | | 100.000 |

**minor diastereoisomer**: Chiralcel OD-H (99.9:0.1 hexane:IPA, flow rate 1.0 mLmin$^{-1}$, 211 nm, 30 °C), $t_R$ (major): 8.5 min, $t_R$ (minor): 9.3 min, 90:10 er.

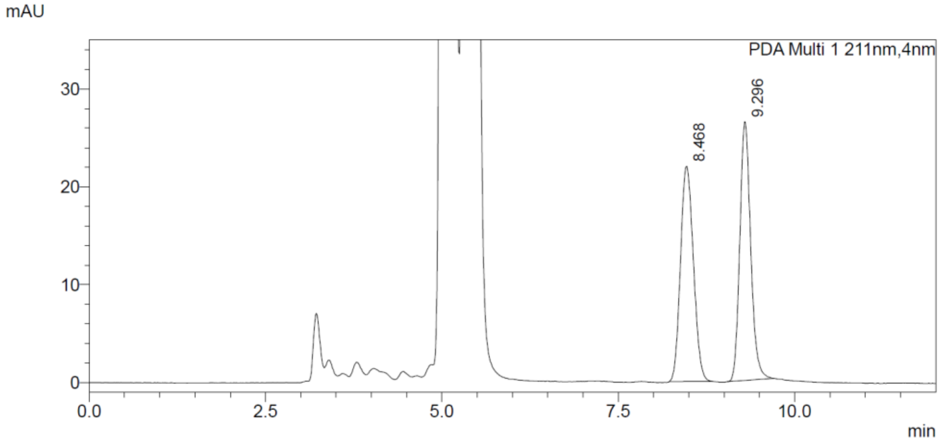


**<Peak Table>**

PDA Ch1 211nm

| Peak# | Ret. Time | Area% |
|---|---|---|
| 1 | 8.468 | 49.710 |
| 2 | 9.296 | 50.290 |
| Total | | 100.000 |

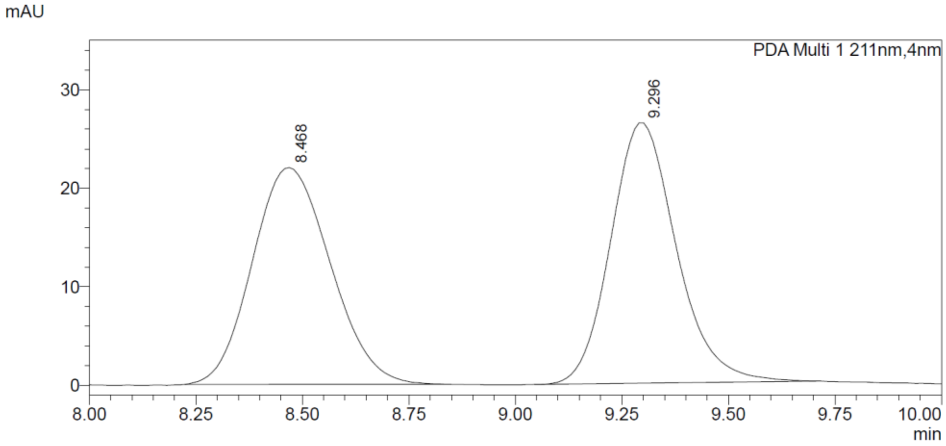


**<Peak Table>**

PDA Ch1 211nm

| Peak# | Ret. Time | Area% |
|---|---|---|
| 1 | 8.468 | 49.710 |
| 2 | 9.296 | 50.290 |
| Total | | 100.000 |

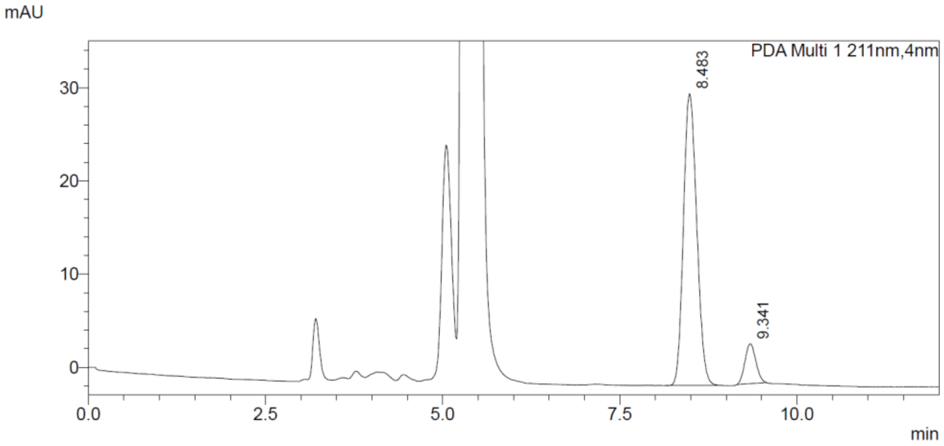


## <Peak Table>

PDA Ch1 211nm

| Peak# | Ret. Time | Area% |
|---|---|---|
| 1 | 8.483 | 89.908 |
| 2 | 9.341 | 10.092 |
| Total | | 100.000 |

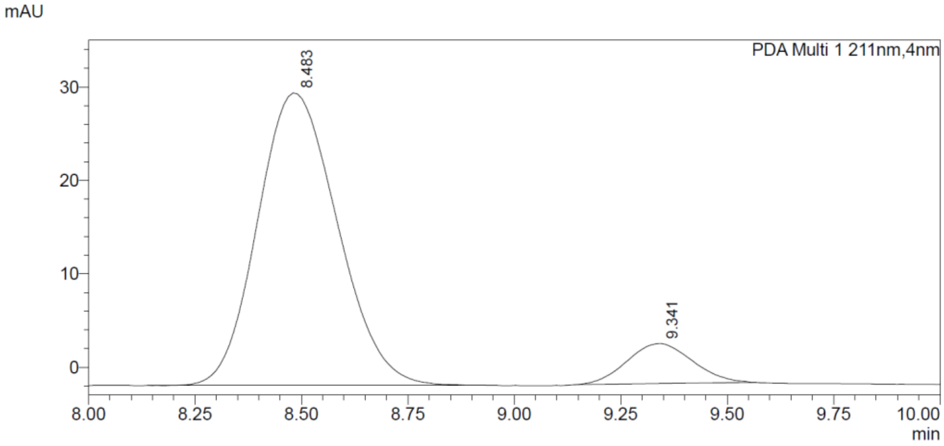


## <Peak Table>

PDA Ch1 211nm

| Peak# | Ret. Time | Area% |
|---|---|---|
| 1 | 8.483 | 89.908 |
| 2 | 9.341 | 10.092 |
| Total | | 100.000 |

HPLC Data for **10**: Chiralcel OD-H (99.5:0.5 hexane:IPA, flow rate 1.0 mL min$^{-1}$, 211 nm, 30 °C), **major diastereoisomer**: $t_R$ (minor): 9.4 min, $t_R$ (major): 10.3 min, 96:4 er.

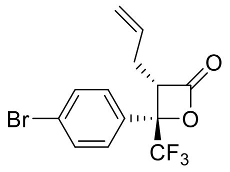

**10**

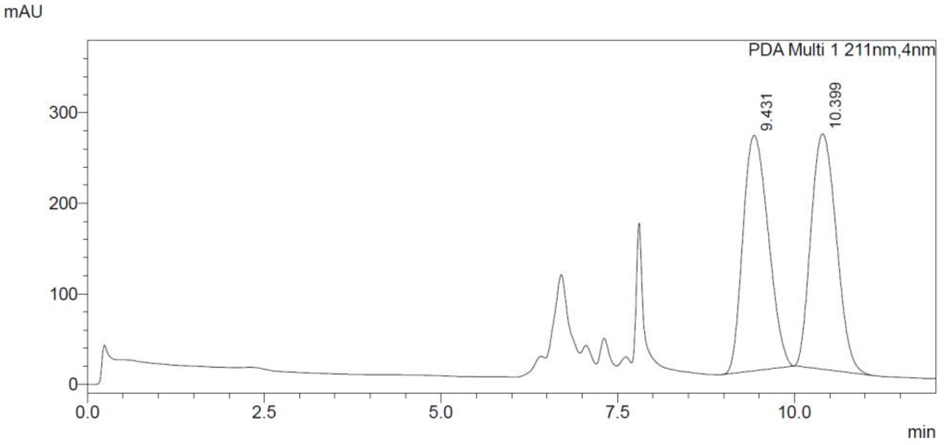


**<Peak Table>**

PDA Ch1 211nm

| Peak# | Ret. Time | Area% |
|---|---|---|
| 1 | 9.431 | 50.040 |
| 2 | 10.399 | 49.960 |
| Total | | 100.000 |

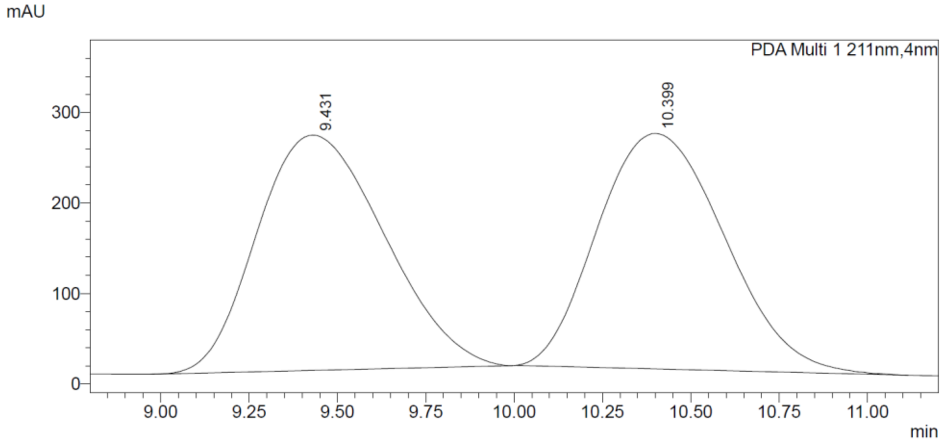


**<Peak Table>**

PDA Ch1 211nm

| Peak# | Ret. Time | Area% |
|---|---|---|
| 1 | 9.431 | 50.040 |
| 2 | 10.399 | 49.960 |
| Total | | 100.000 |

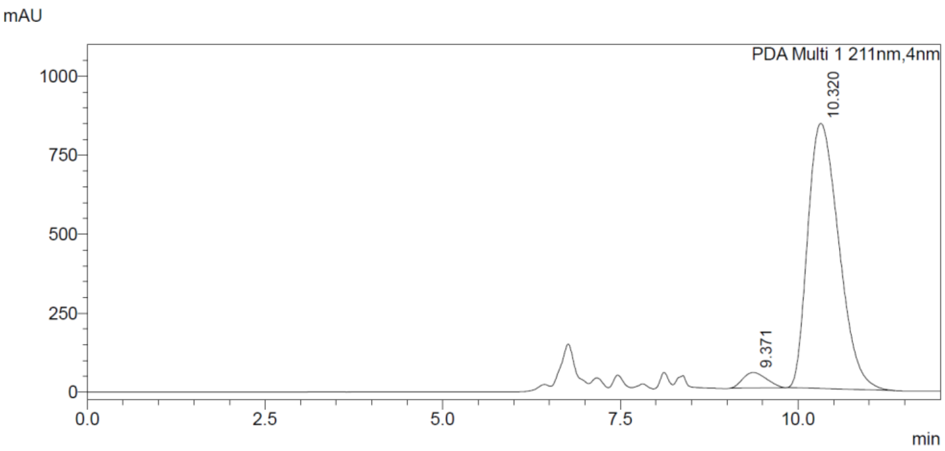


**<Peak Table>**

PDA Ch1 211nm

| Peak# | Ret. Time | Area% |
|---|---|---|
| 1 | 9.371 | 4.227 |
| 2 | 10.320 | 95.773 |
| Total | | 100.000 |

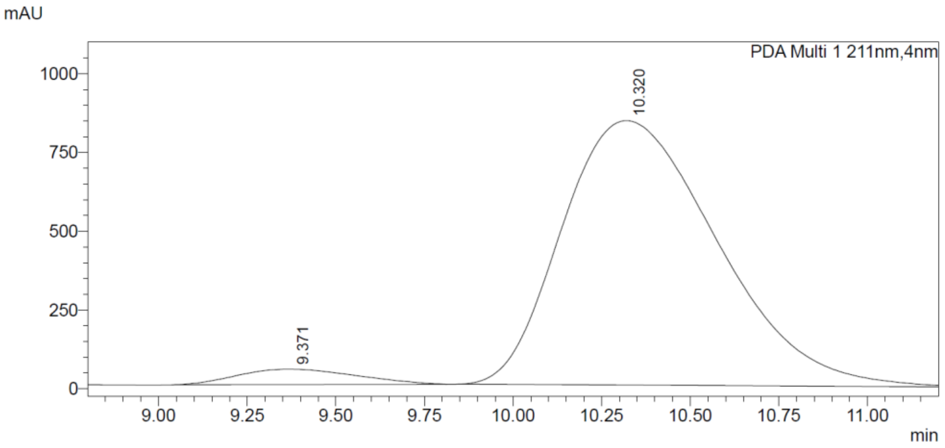


**<Peak Table>**

PDA Ch1 211nm

| Peak# | Ret. Time | Area% |
|---|---|---|
| 1 | 9.371 | 4.227 |
| 2 | 10.320 | 95.773 |
| Total | | 100.000 |

**Minor diastereoisomer**: $t_R$ (major): 13.6 min, $t_R$ (minor): 14.8 min, 72:28 er.

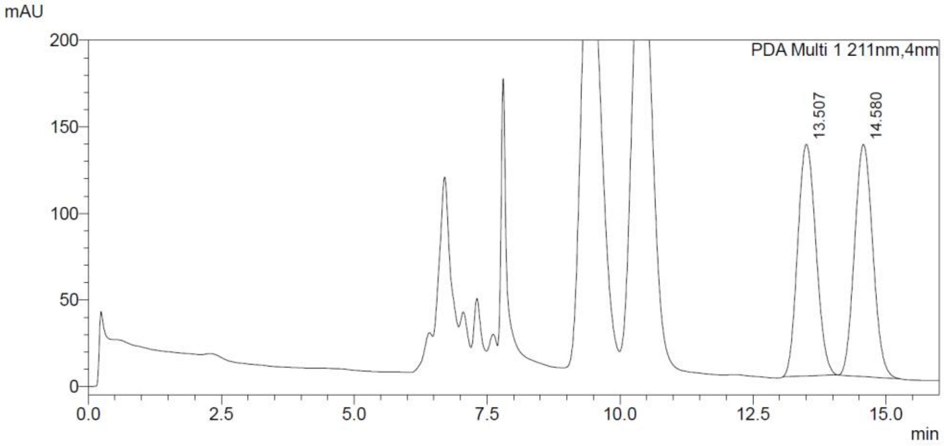


**<Peak Table>**

PDA Ch1 211nm

| Peak# | Ret. Time | Area% |
|---|---|---|
| 1 | 13.507 | 49.874 |
| 2 | 14.580 | 50.126 |
| Total | | 100.000 |

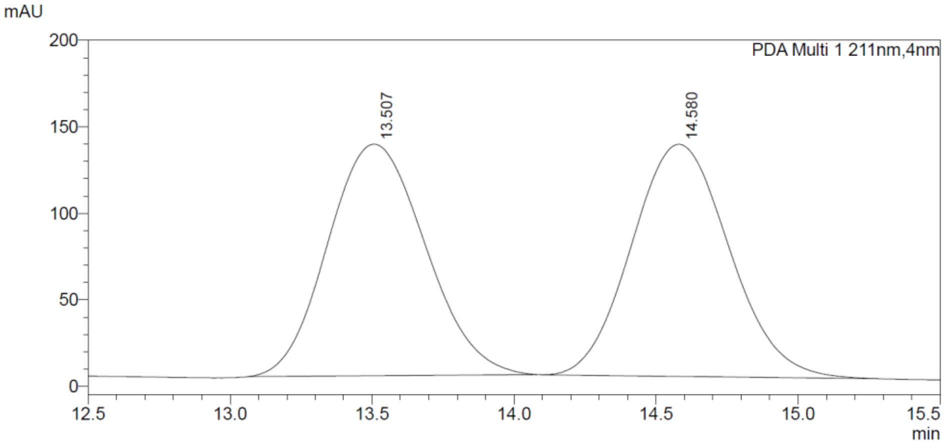



**<Peak Table>**

PDA Ch1 211nm

| Peak# | Ret. Time | Area% |
|---|---|---|
| 1 | 13.507 | 49.874 |
| 2 | 14.580 | 50.126 |
| Total | | 100.000 |

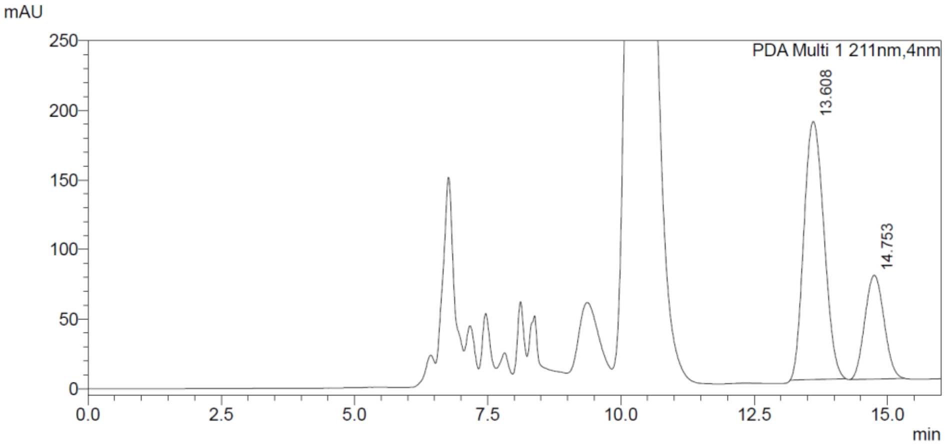


<Peak Table>

PDA Ch1 211nm

| Peak# | Ret. Time | Area% |
|---|---|---|
| 1 | 13.608 | 72.264 |
| 2 | 14.753 | 27.736 |
| Total | | 100.000 |

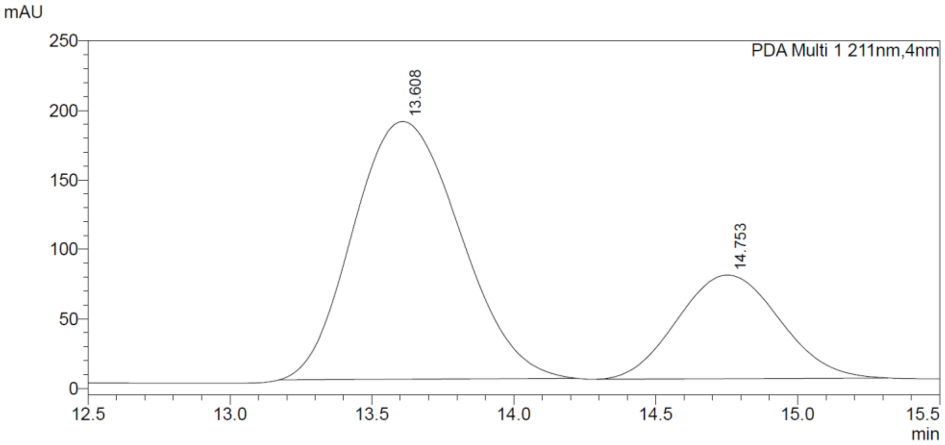


<Peak Table>

PDA Ch1 211nm

| Peak# | Ret. Time | Area% |
|---|---|---|
| 1 | 13.608 | 72.264 |
| 2 | 14.753 | 27.736 |
| Total | | 100.000 |

HPLC Data for **11**: Chiralcel OJ-H (99.5:0.5 hexane:IPA, flow rate 1.0 mLmin$^{-1}$, 211 nm, 30 °C), $t_R$ (major): 13.7 min, $t_R$ (minor): 17.4 min, 98:2 er.

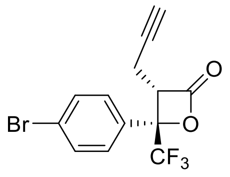


**11**

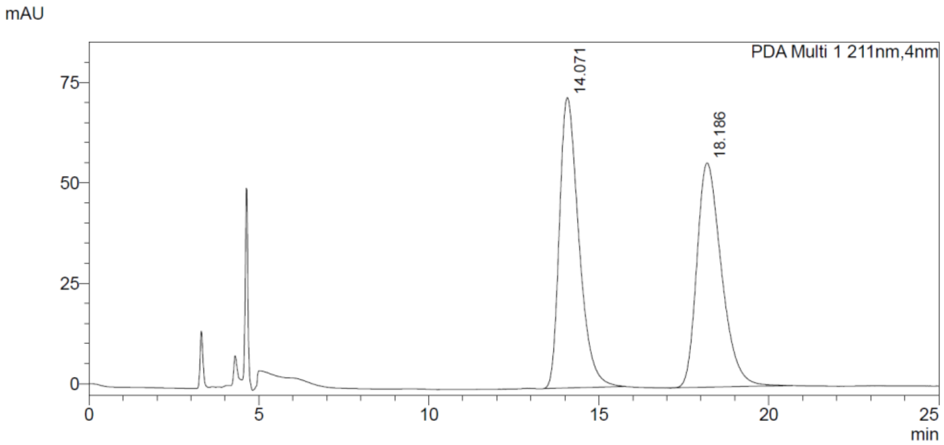


**<Peak Table>**

PDA Ch1 211nm

| Peak# | Ret. Time | Area% |
|---|---|---|
| 1 | 14.071 | 50.448 |
| 2 | 18.186 | 49.552 |
| Total | | 100.000 |

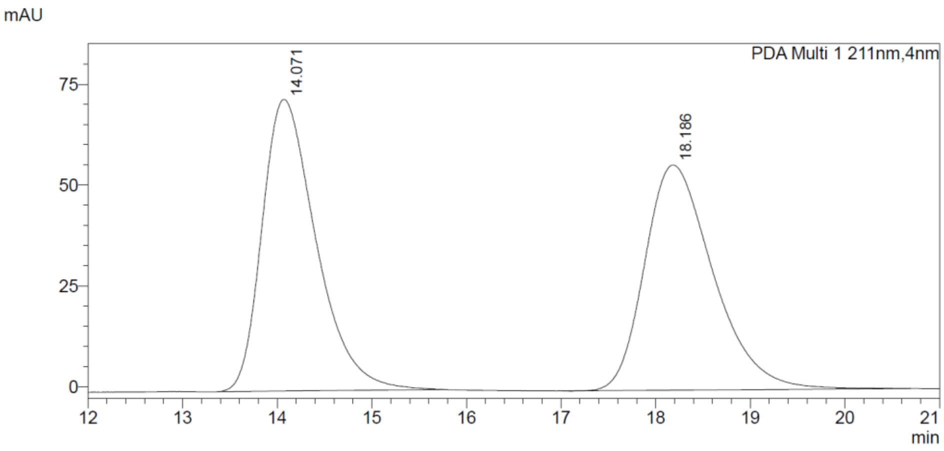


**<Peak Table>**

PDA Ch1 211nm

| Peak# | Ret. Time | Area% |
|---|---|---|
| 1 | 14.071 | 50.448 |
| 2 | 18.186 | 49.552 |
| Total | | 100.000 |

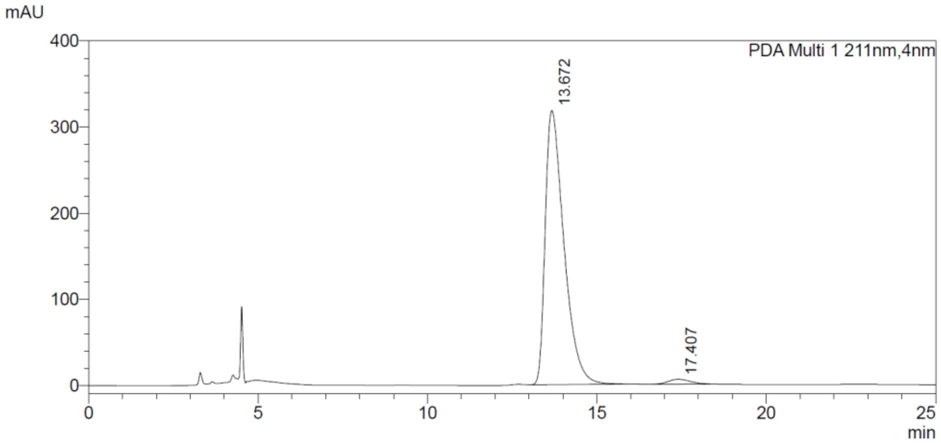


**<Peak Table>**

PDA Ch1 211nm

| Peak# | Ret. Time | Area% |
|---|---|---|
| 1 | 13.672 | 97.941 |
| 2 | 17.407 | 2.059 |
| Total | | 100.000 |

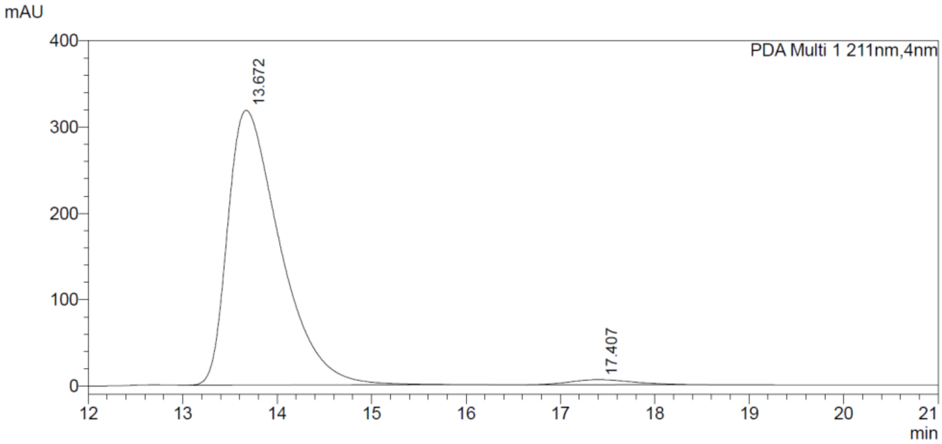


**<Peak Table>**

PDA Ch1 211nm

| Peak# | Ret. Time | Area% |
|---|---|---|
| 1 | 13.672 | 97.941 |
| 2 | 17.407 | 2.059 |
| Total | | 100.000 |

HPLC Data for **12**: **major diastereoisomer**: Chiralpak IB (99.9:0.1 hexane:IPA, flow rate 1.0 mLmin$^{-1}$, 211 nm, 30 °C), $t_R$ (minor): 20.9 min, $t_R$(major): 21.5 min, >99:1 er.

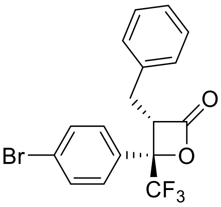


**12**

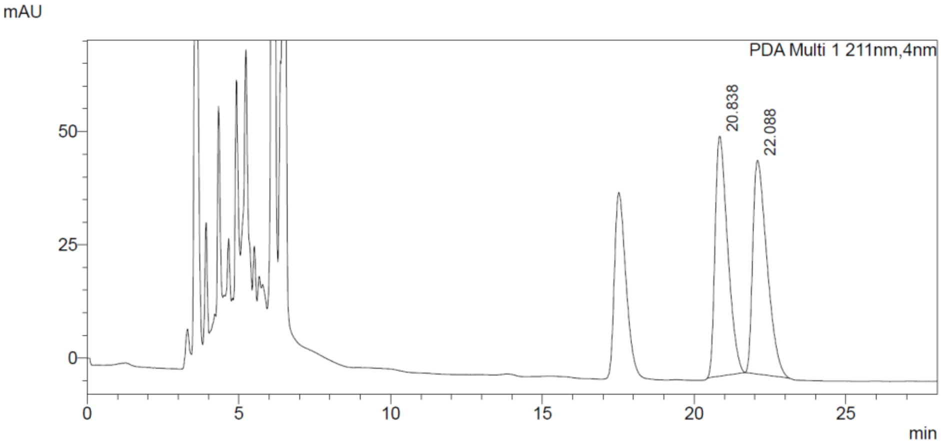


**<Peak Table>**

PDA Ch1 211nm

| Peak# | Ret. Time | Area% |
|---|---|---|
| 1 | 20.838 | 49.862 |
| 2 | 22.088 | 50.138 |
| Total | | 100.000 |

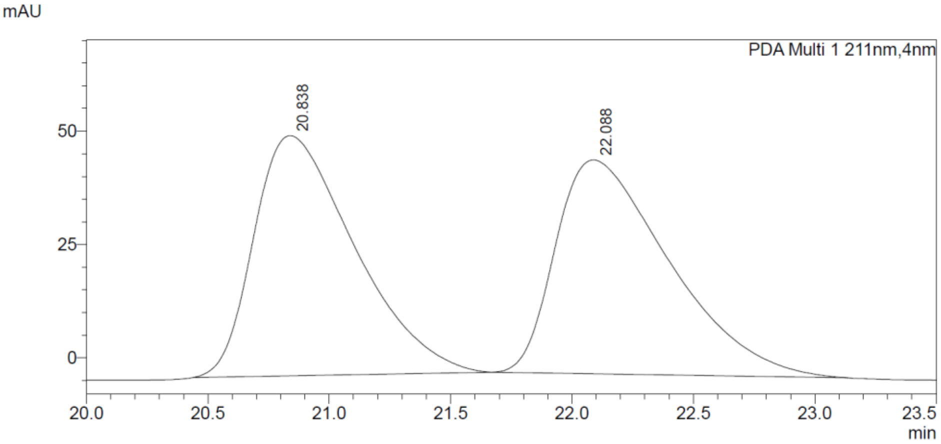


**<Peak Table>**

PDA Ch1 211nm

| Peak# | Ret. Time | Area% |
|---|---|---|
| 1 | 20.838 | 49.862 |
| 2 | 22.088 | 50.138 |
| Total | | 100.000 |

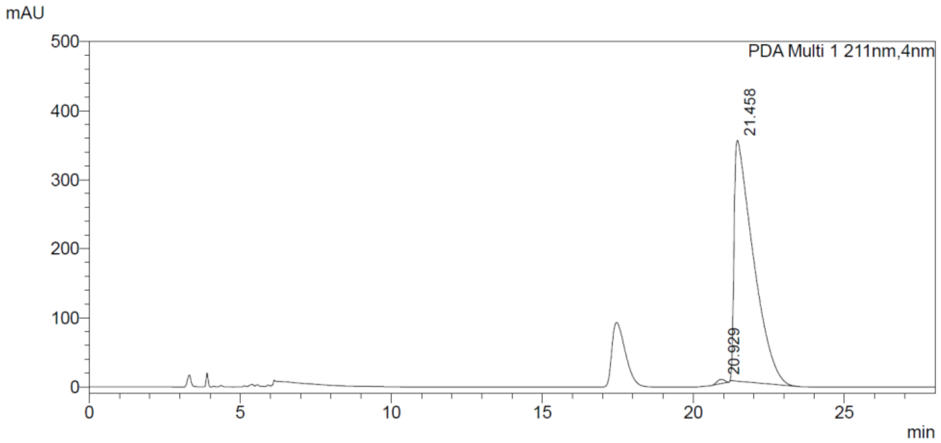


**<Peak Table>**

PDA Ch1 211nm

| Peak# | Ret. Time | Area% |
|---|---|---|
| 1 | 20.929 | 0.676 |
| 2 | 21.458 | 99.324 |
| Total | | 100.000 |

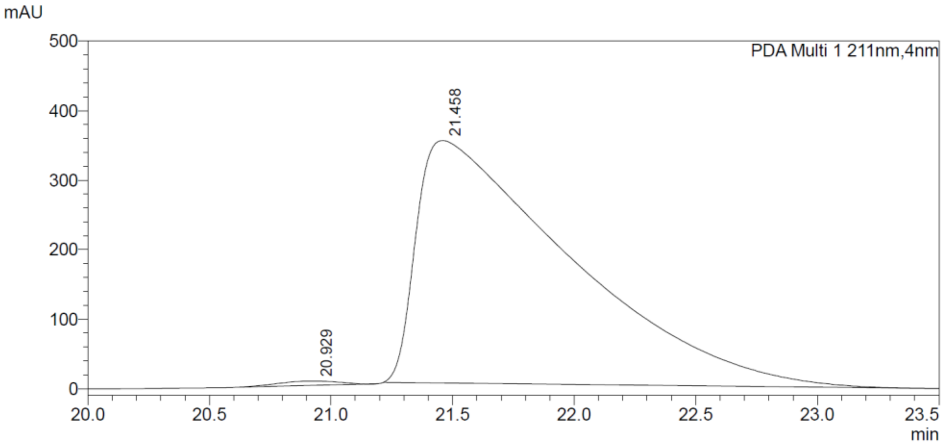


**<Peak Table>**

PDA Ch1 211nm

| Peak# | Ret. Time | Area% |
|---|---|---|
| 1 | 20.929 | 0.676 |
| 2 | 21.458 | 99.324 |
| Total | | 100.000 |

**Minor diastereoisomer**: Chiralpak IB (99.9:0.1 hexane:IPA, flow rate 1.0 mLmin$^{-1}$, 254 nm, 30 °C), $t_R$ (major): 19.0 min, $t_R$ (minor): 35.0 min, 82:18 er.

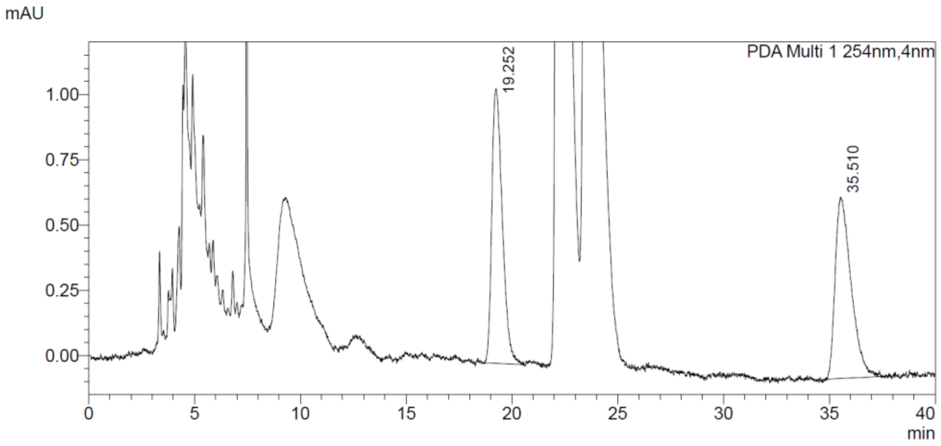


**<Peak Table>**

PDA Ch1 254nm

| Peak# | Ret. Time | Area% |
|---|---|---|
| 1 | 19.252 | 49.926 |
| 2 | 35.510 | 50.074 |
| Total | | 100.000 |

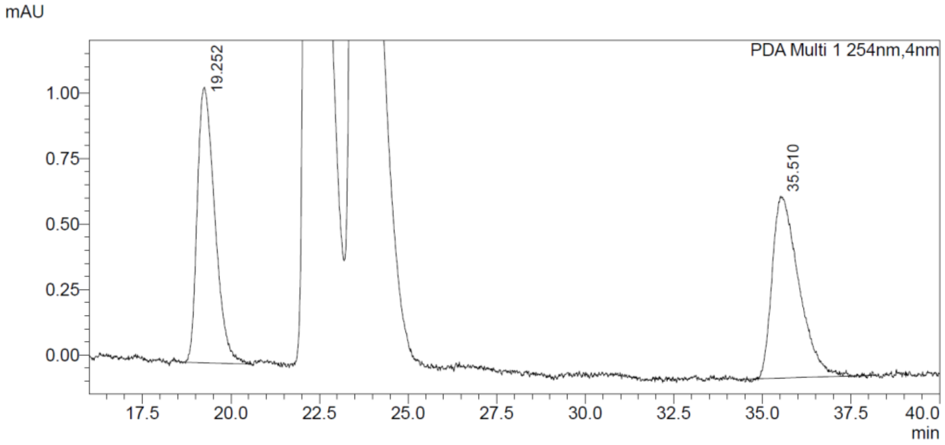


**<Peak Table>**

PDA Ch1 254nm

| Peak# | Ret. Time | Area% |
|---|---|---|
| 1 | 19.252 | 49.926 |
| 2 | 35.510 | 50.074 |
| Total | | 100.000 |

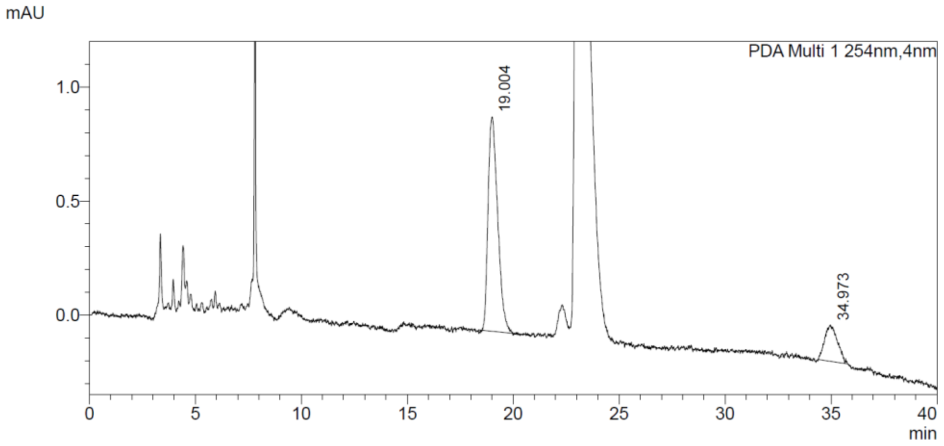


**<Peak Table>**

PDA Ch1 254nm

| Peak# | Ret. Time | Area% |
|---|---|---|
| 1 | 19.004 | 81.691 |
| 2 | 34.973 | 18.309 |
| Total | | 100.000 |

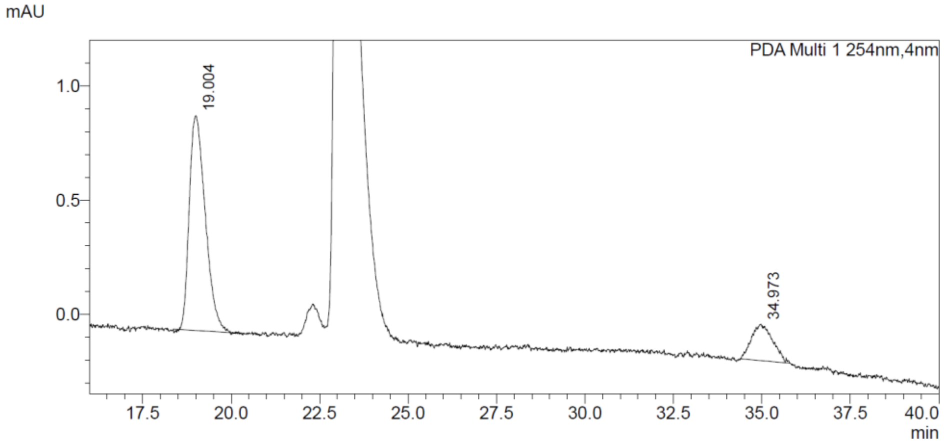


**<Peak Table>**

PDA Ch1 254nm

| Peak# | Ret. Time | Area% |
|---|---|---|
| 1 | 19.004 | 81.691 |
| 2 | 34.973 | 18.309 |
| Total | | 100.000 |

HPLC Data for **13**: Chiralpak IB (99.9:0.1 hexane:IPA, flow rate 1.0 mLmin$^{-1}$, 211 nm, 30 °C), **major diastereoisomer**: $t_R$ (major): 16.5 min, $t_R$ (minor): 21.3 min, 97:3 er.

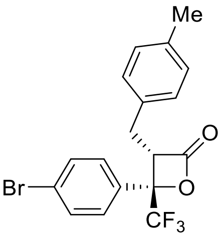

**13**

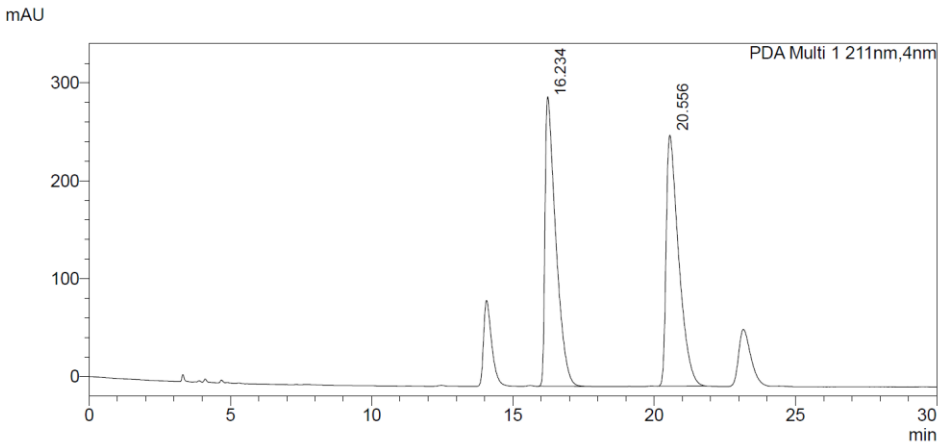


**<Peak Table>**

PDA Ch1 211nm

| Peak# | Ret. Time | Area% |
|---|---|---|
| 1 | 16.234 | 49.770 |
| 2 | 20.556 | 50.230 |
| Total | | 100.000 |

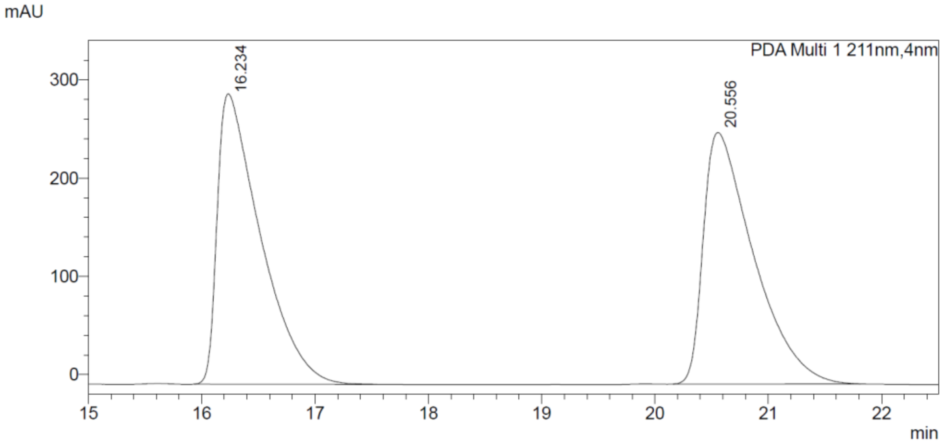


**<Peak Table>**

PDA Ch1 211nm

| Peak# | Ret. Time | Area% |
|---|---|---|
| 1 | 16.234 | 49.770 |
| 2 | 20.556 | 50.230 |
| Total | | 100.000 |

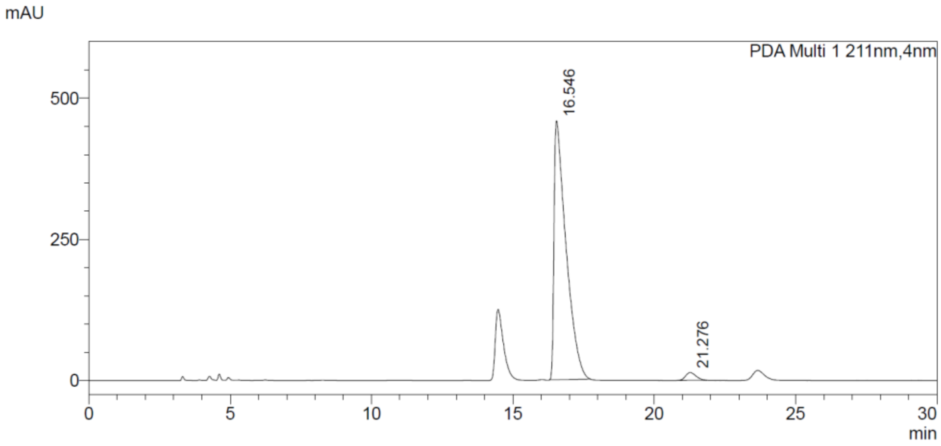


**<Peak Table>**

PDA Ch1 211nm

| Peak# | Ret. Time | Area% |
|---|---|---|
| 1 | 16.546 | 97.408 |
| 2 | 21.276 | 2.592 |
| Total | | 100.000 |

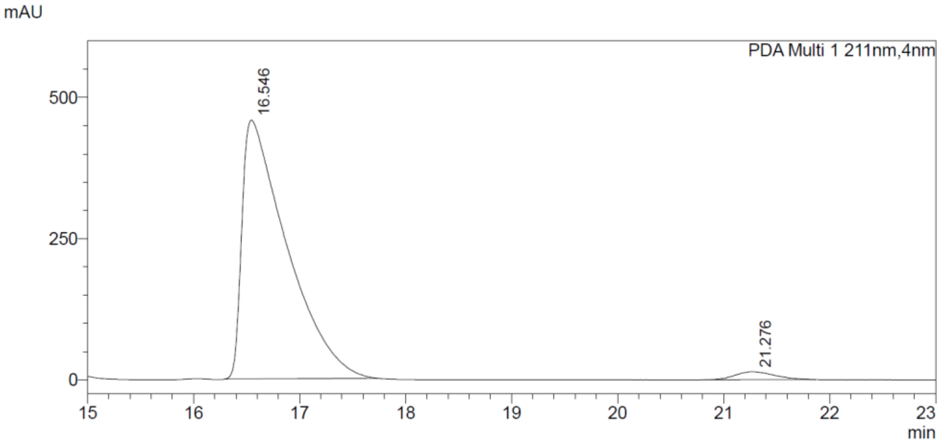


**<Peak Table>**

PDA Ch1 211nm

| Peak# | Ret. Time | Area% |
|---|---|---|
| 1 | 16.546 | 97.408 |
| 2 | 21.276 | 2.592 |
| Total | | 100.000 |

**Minor diastereoisomer**: $t_R$ (major): 14.5 min, $t_R$ (minor): 23.7 min, 83:17 er.

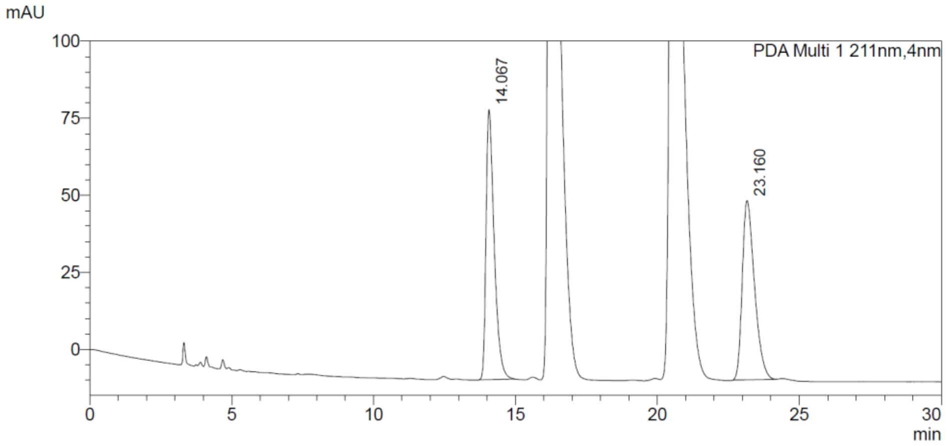


**<Peak Table>**

PDA Ch1 211nm

| Peak# | Ret. Time | Area% |
|---|---|---|
| 1 | 14.067 | 50.010 |
| 2 | 23.160 | 49.990 |
| Total | | 100.000 |

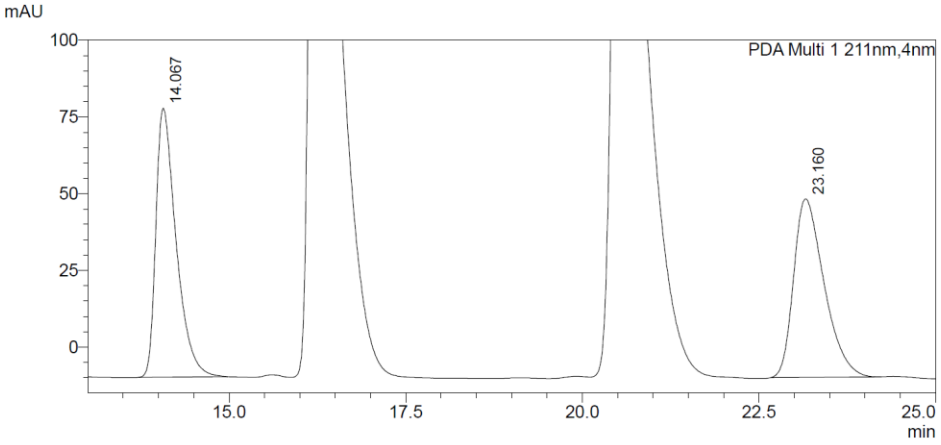


**<Peak Table>**

PDA Ch1 211nm

| Peak# | Ret. Time | Area% |
|---|---|---|
| 1 | 14.067 | 50.010 |
| 2 | 23.160 | 49.990 |
| Total | | 100.000 |

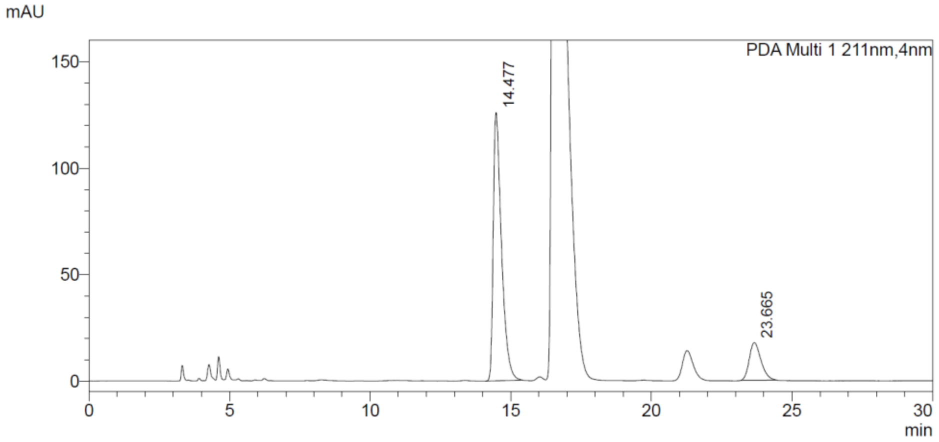


### <Peak Table>

PDA Ch1 211nm

| Peak# | Ret. Time | Area% |
|---|---|---|
| 1 | 14.477 | 83.436 |
| 2 | 23.665 | 16.564 |
| Total | | 100.000 |

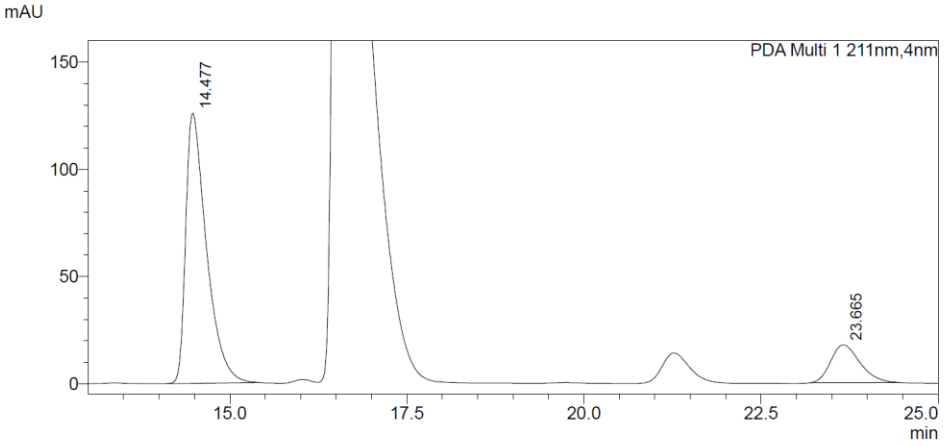



### <Peak Table>

PDA Ch1 211nm

| Peak# | Ret. Time | Area% |
|---|---|---|
| 1 | 14.477 | 83.436 |
| 2 | 23.665 | 16.564 |
| Total | | 100.000 |

HPLC Data for **14**: Chiralpak AD-H (99.5:0.5 hexane:IPA, flow rate 1.0 mLmin$^{-1}$, 211 nm, 30 °C), **major diastereoisomer**: $t_R$ (major): 8.6 min, $t_R$ (minor): 9.4 min, 98:2 er.

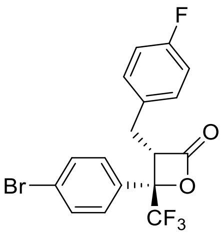


**14**

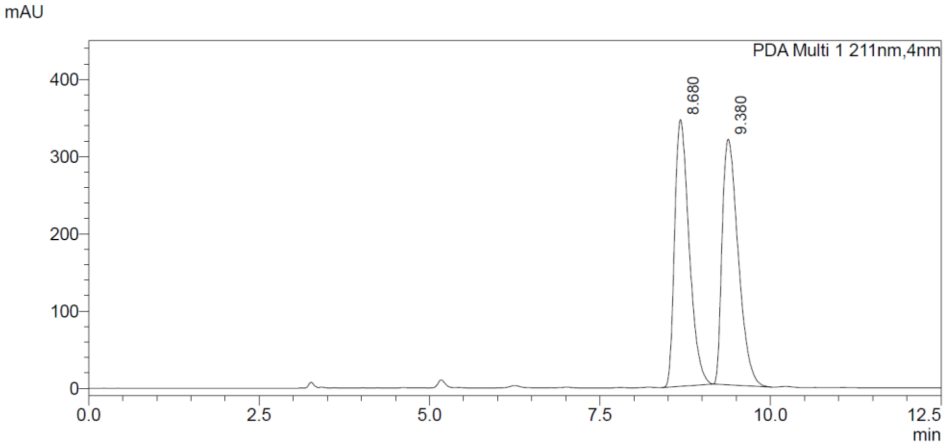


**<Peak Table>**

PDA Ch1 211nm

| Peak# | Ret. Time | Area% |
|---|---|---|
| 1 | 8.680 | 49.523 |
| 2 | 9.380 | 50.477 |
| Total | | 100.000 |

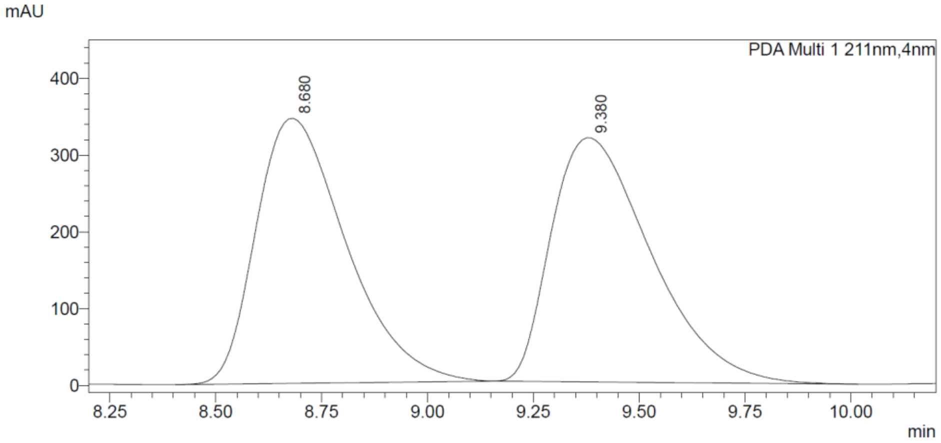


**<Peak Table>**

PDA Ch1 211nm

| Peak# | Ret. Time | Area% |
|---|---|---|
| 1 | 8.680 | 49.523 |
| 2 | 9.380 | 50.477 |
| Total | | 100.000 |

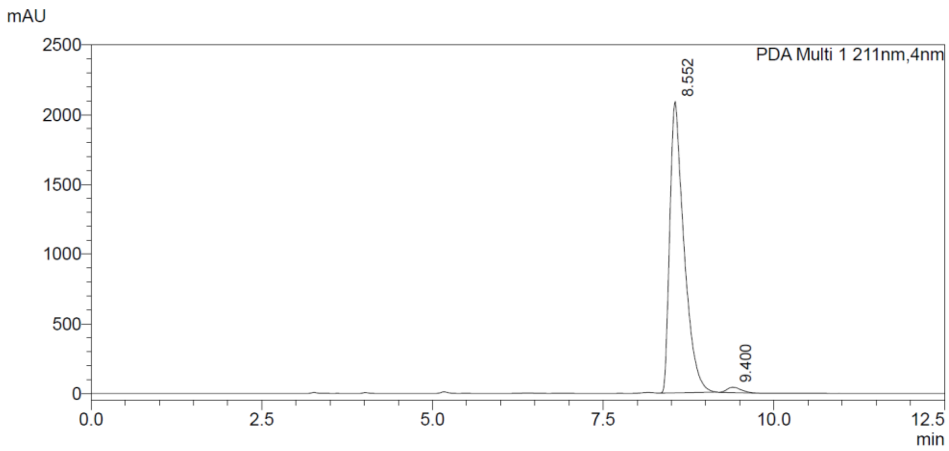


## <Peak Table>

PDA Ch1 211nm

| Peak# | Ret. Time | Area% |
|---|---|---|
| 1 | 8.552 | 98.288 |
| 2 | 9.400 | 1.712 |
| Total | | 100.000 |

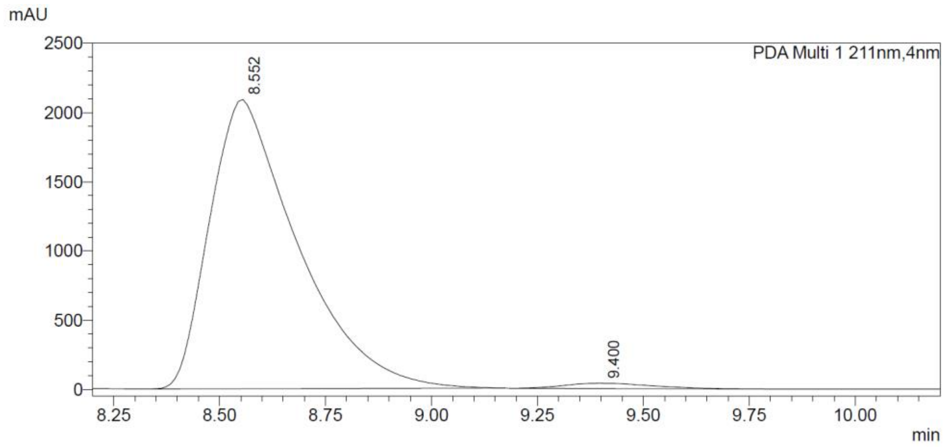


## <Peak Table>

PDA Ch1 211nm

| Peak# | Ret. Time | Area% |
|---|---|---|
| 1 | 8.552 | 98.288 |
| 2 | 9.400 | 1.712 |
| Total | | 100.000 |

**Minor diastereoisomer**: $t_R$ (minor): 13.0 min, $t_R$ (minor): 13.9 min, 85:15 er.

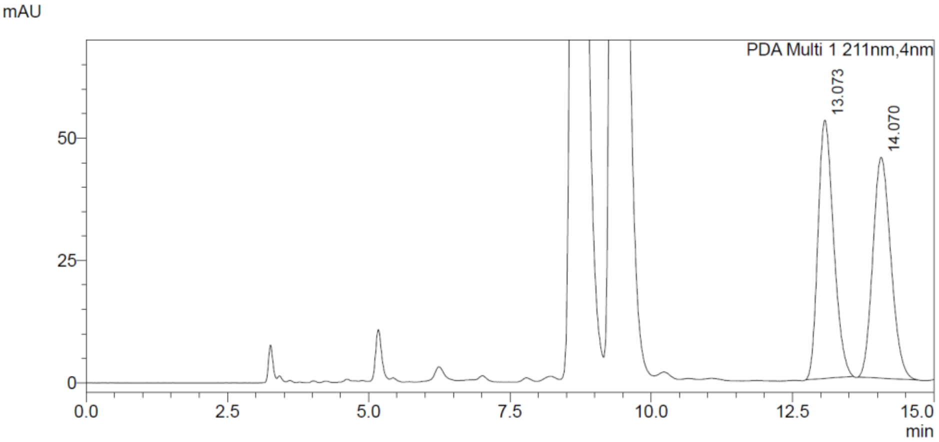


**<Peak Table>**

PDA Ch1 211nm

| Peak# | Ret. Time | Area% |
|---|---|---|
| 1 | 13.073 | 50.263 |
| 2 | 14.070 | 49.737 |
| Total | | 100.000 |

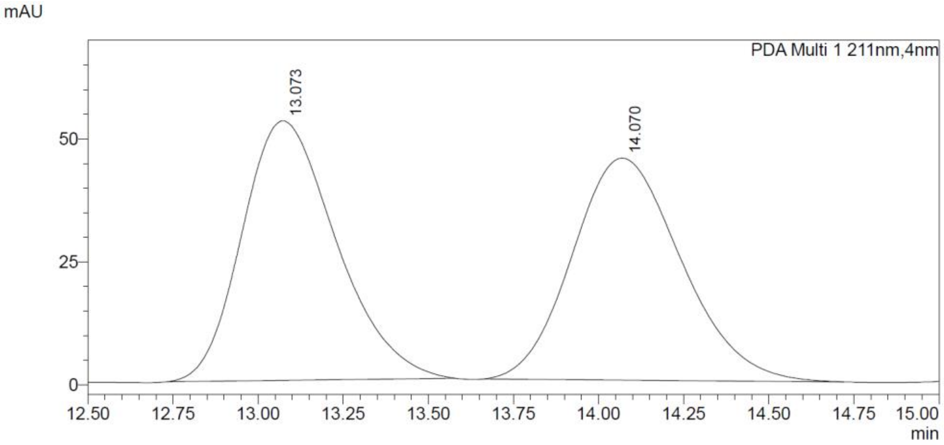


**<Peak Table>**

PDA Ch1 211nm

| Peak# | Ret. Time | Area% |
|---|---|---|
| 1 | 13.073 | 50.263 |
| 2 | 14.070 | 49.737 |
| Total | | 100.000 |

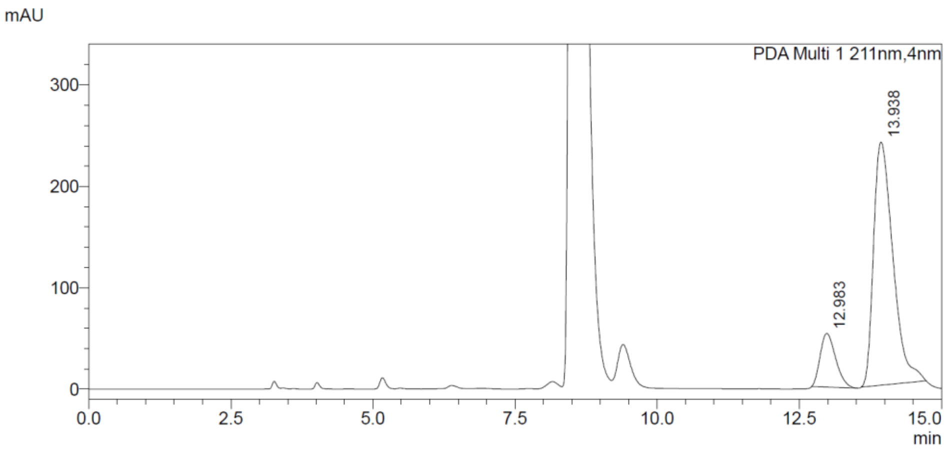


**<Peak Table>**

PDA Ch1 211nm

| Peak# | Ret. Time | Area% |
|---|---|---|
| 1 | 12.983 | 15.056 |
| 2 | 13.938 | 84.944 |
| Total | | 100.000 |

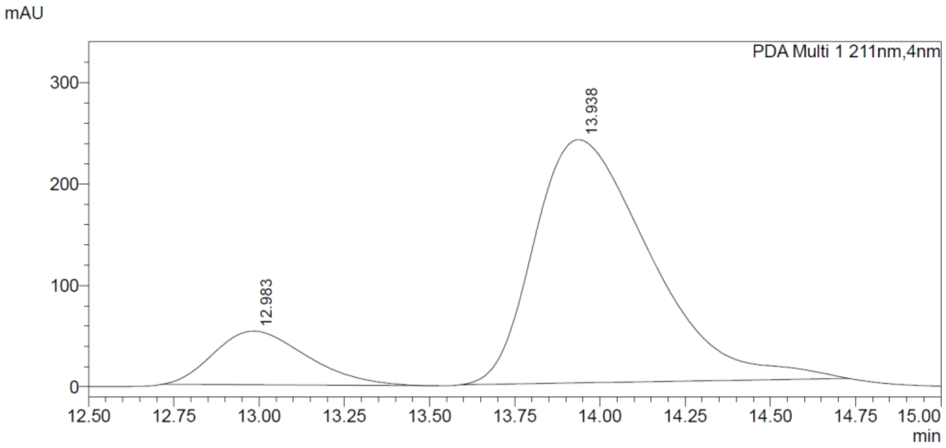


**<Peak Table>**

PDA Ch1 211nm

| Peak# | Ret. Time | Area% |
|---|---|---|
| 1 | 12.983 | 15.056 |
| 2 | 13.938 | 84.944 |
| Total | | 100.000 |

HPLC Data for **15**: Chiralcel OD-H (98:2 hexane:IPA, flow rate 1.0 mLmin$^{-1}$, 211 nm, 30 °C), **major diastereoisomer**: $t_R$ (minor): 26.2 min, $t_R$ (major): 32.0 min, 99:1 er.

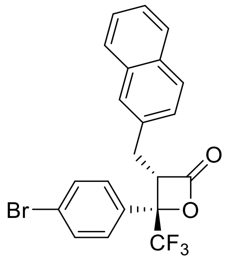


**15**

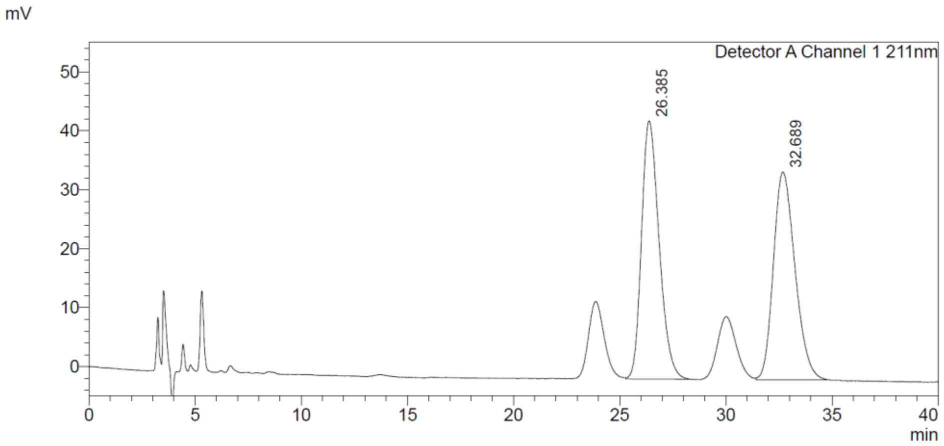


**<Peak Table>**

Detector A Channel 1 211nm

| Peak# | Ret. Time | Area% |
|---|---|---|
| 1 | 26.385 | 50.359 |
| 2 | 32.689 | 49.641 |
| Total | | 100.000 |

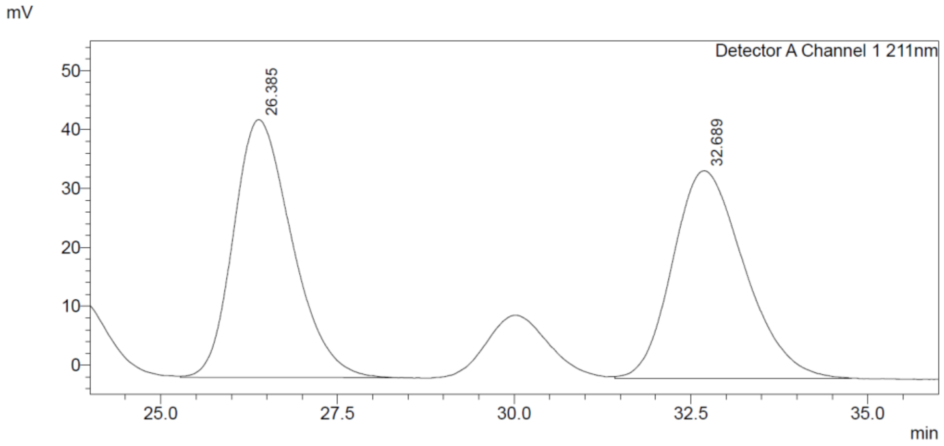


**<Peak Table>**

Detector A Channel 1 211nm

| Peak# | Ret. Time | Area% |
|---|---|---|
| 1 | 26.385 | 50.359 |
| 2 | 32.689 | 49.641 |
| Total | | 100.000 |

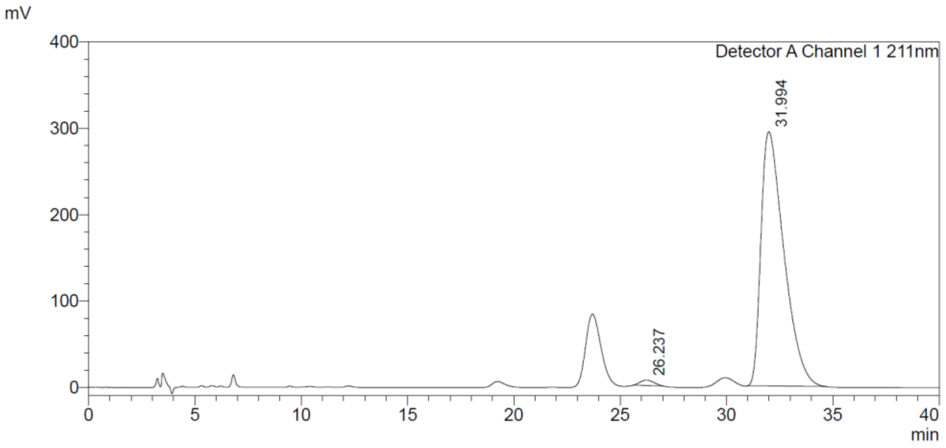


## <Peak Table>

Detector A Channel 1 211nm

| Peak# | Ret. Time | Area% |
|---|---|---|
| 1 | 26.237 | 1.262 |
| 2 | 31.994 | 98.738 |
| Total | | 100.000 |

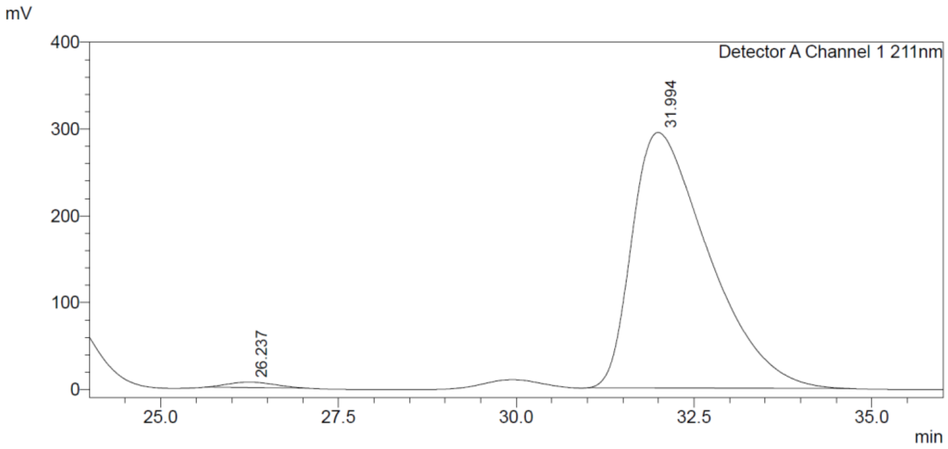


## <Peak Table>

Detector A Channel 1 211nm

| Peak# | Ret. Time | Area% |
|---|---|---|
| 1 | 26.237 | 1.262 |
| 2 | 31.994 | 98.738 |
| Total | | 100.000 |

**Minor diastereoisomer**: $t_R$ (major): 23.7 min, $t_R$ (minor): 29.9 min, 89:11 er.

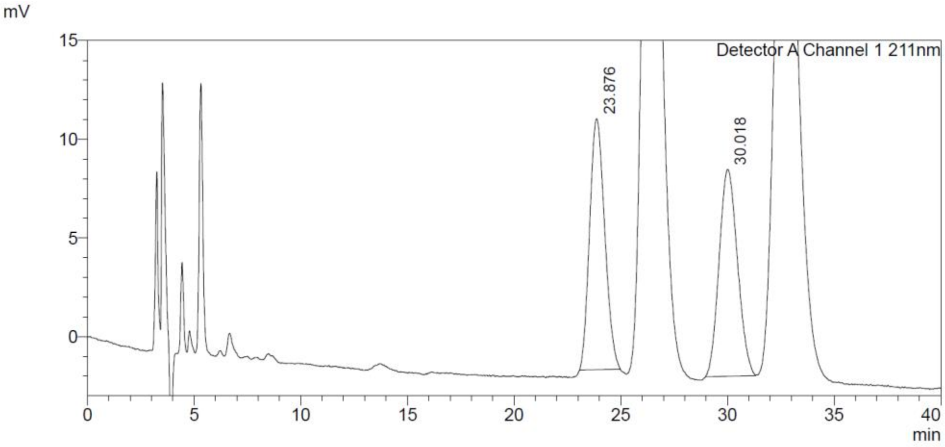


**<Peak Table>**

Detector A Channel 1 211nm

| Peak# | Ret. Time | Area% |
|---|---|---|
| 1 | 23.876 | 49.986 |
| 2 | 30.018 | 50.014 |
| Total | | 100.000 |

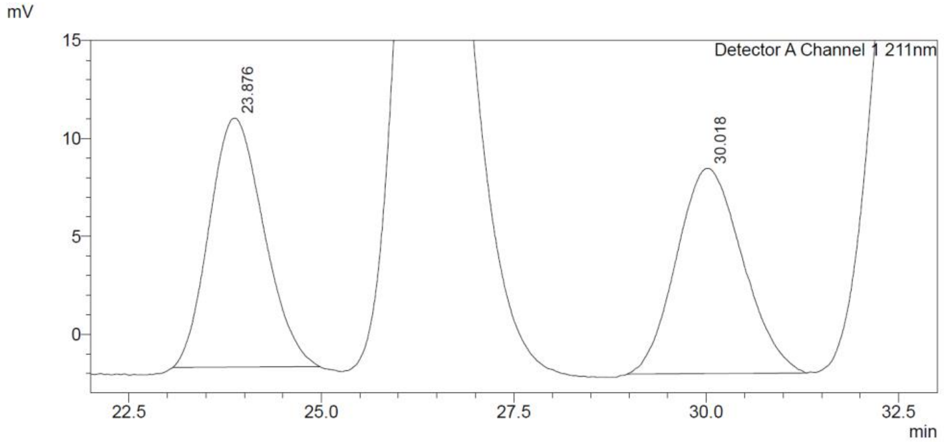


**<Peak Table>**

Detector A Channel 1 211nm

| Peak# | Ret. Time | Area% |
|---|---|---|
| 1 | 23.876 | 49.986 |
| 2 | 30.018 | 50.014 |
| Total | | 100.000 |

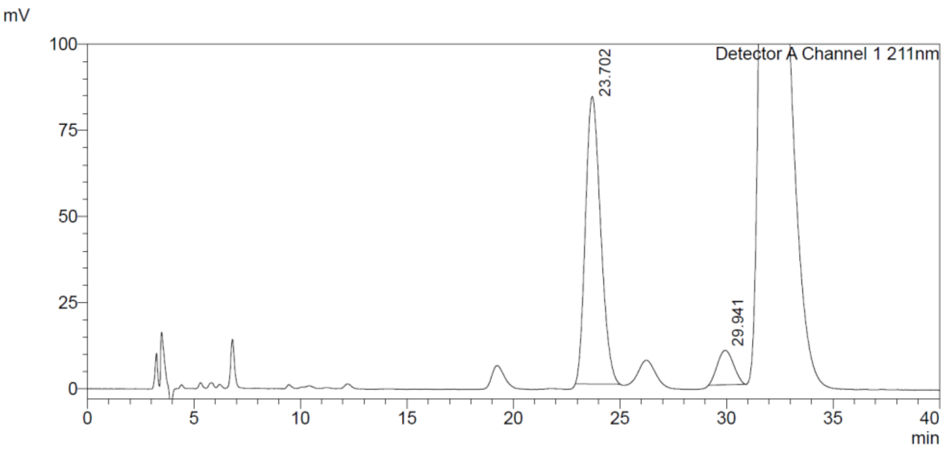


**<Peak Table>**

Detector A Channel 1 211nm

| Peak# | Ret. Time | Area% |
|---|---|---|
| 1 | 23.702 | 88.772 |
| 2 | 29.941 | 11.228 |
| Total | | 100.000 |

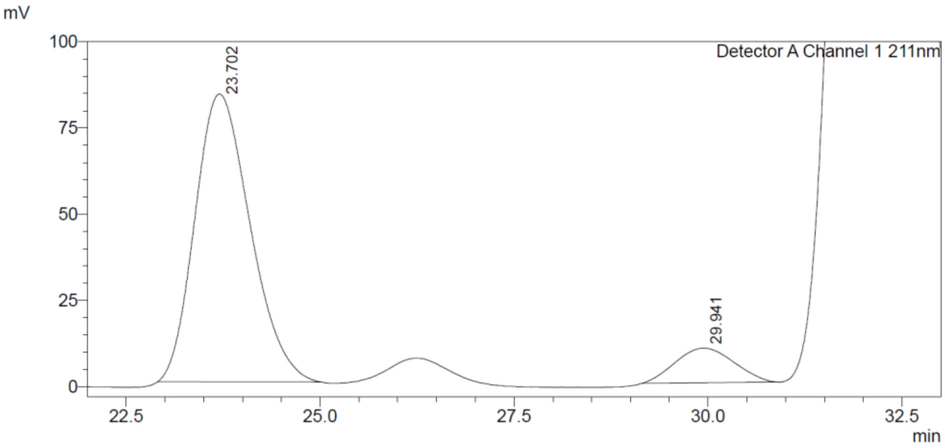


**<Peak Table>**

Detector A Channel 1 211nm

| Peak# | Ret. Time | Area% |
|---|---|---|
| 1 | 23.702 | 88.772 |
| 2 | 29.941 | 11.228 |
| Total | | 100.000 |

HPLC Data for **16**: Chiralcel OD-H (97:3 hexane:IPA, flow rate 1.0 mLmin$^{-1}$, 211 nm, 40 °C),

**major diastereoisomer**: $t_R$ (minor): 5.0 min, $t_R$ (major): 6.6 min, 96:4 er.

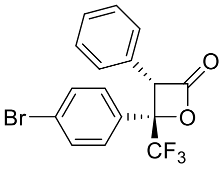

**16**

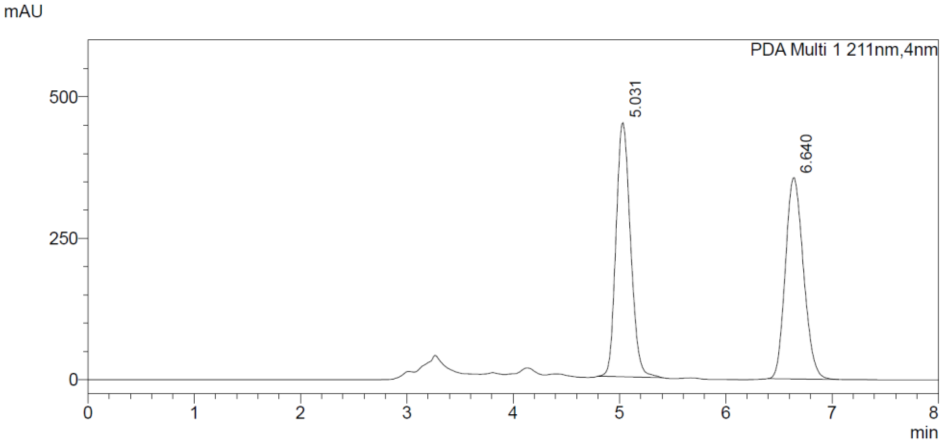


**<Peak Table>**

PDA Ch1 211nm

| Peak# | Ret. Time | Area% |
|---|---|---|
| 1 | 5.031 | 50.248 |
| 2 | 6.640 | 49.752 |
| Total | | 100.000 |

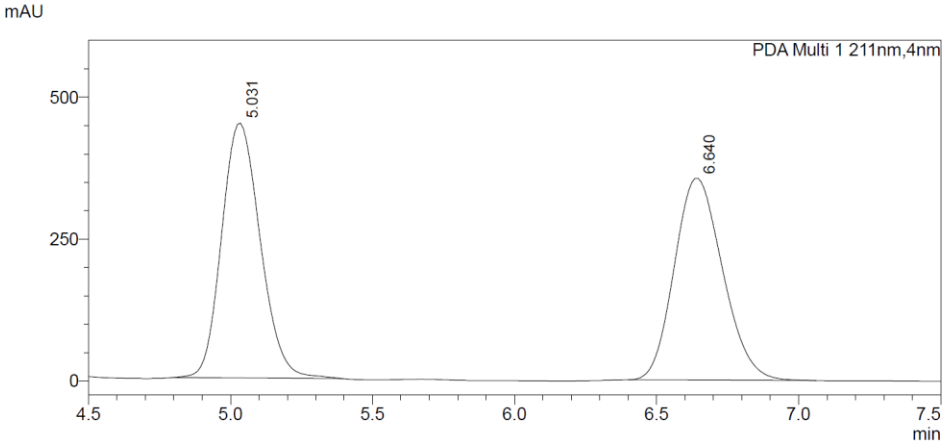


**<Peak Table>**

PDA Ch1 211nm

| Peak# | Ret. Time | Area% |
|---|---|---|
| 1 | 5.031 | 50.248 |
| 2 | 6.640 | 49.752 |
| Total | | 100.000 |

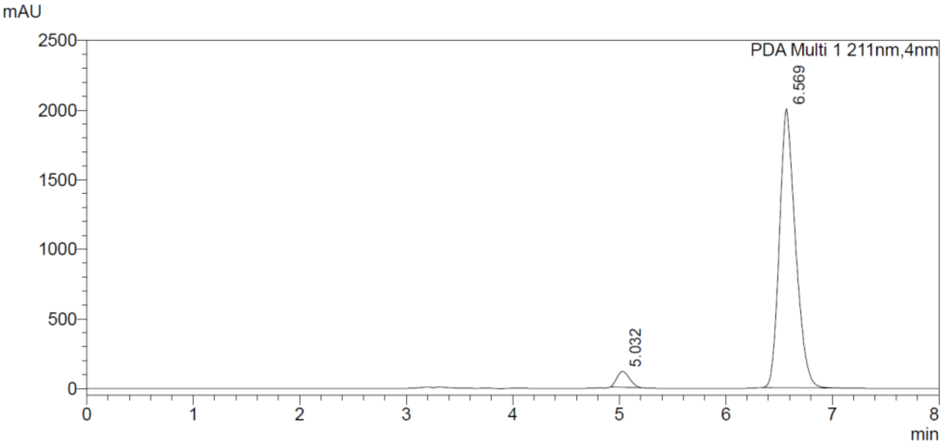


**<Peak Table>**

PDA Ch1 211nm

| Peak# | Ret. Time | Area% |
|---|---|---|
| 1 | 5.032 | 4.212 |
| 2 | 6.569 | 95.788 |
| Total | | 100.000 |

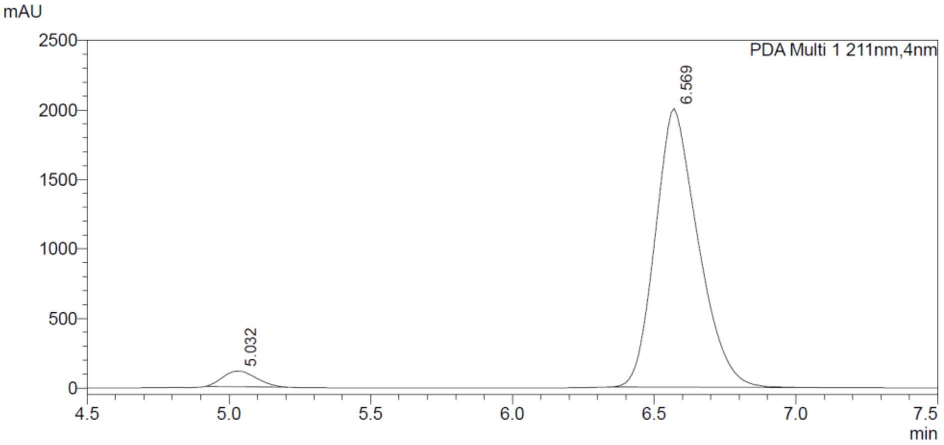


**<Peak Table>**

PDA Ch1 211nm

| Peak# | Ret. Time | Area% |
|---|---|---|
| 1 | 5.032 | 4.212 |
| 2 | 6.569 | 95.788 |
| Total | | 100.000 |

**Minor diastereoisomer**: $t_R$ (major): 8.8 min, $t_R$ (minor): 9.8 min, 95:5 er.

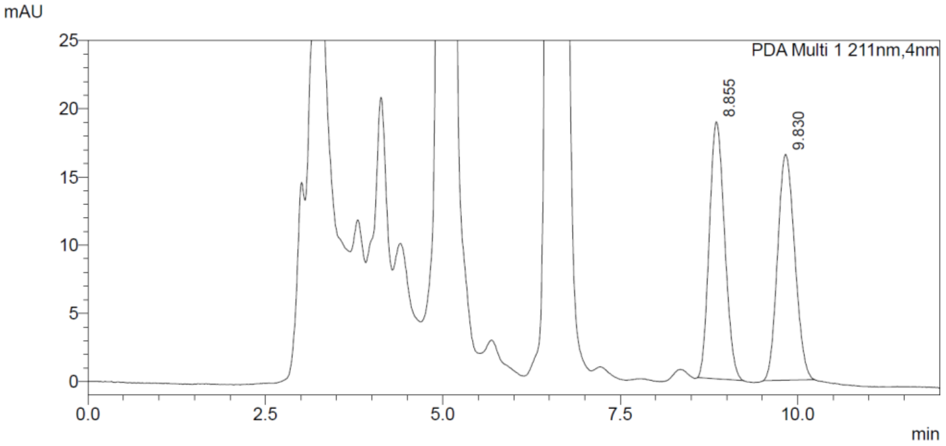


**<Peak Table>**

PDA Ch1 211nm

| Peak# | Ret. Time | Area% |
|---|---|---|
| 1 | 8.855 | 50.280 |
| 2 | 9.830 | 49.720 |
| Total | | 100.000 |

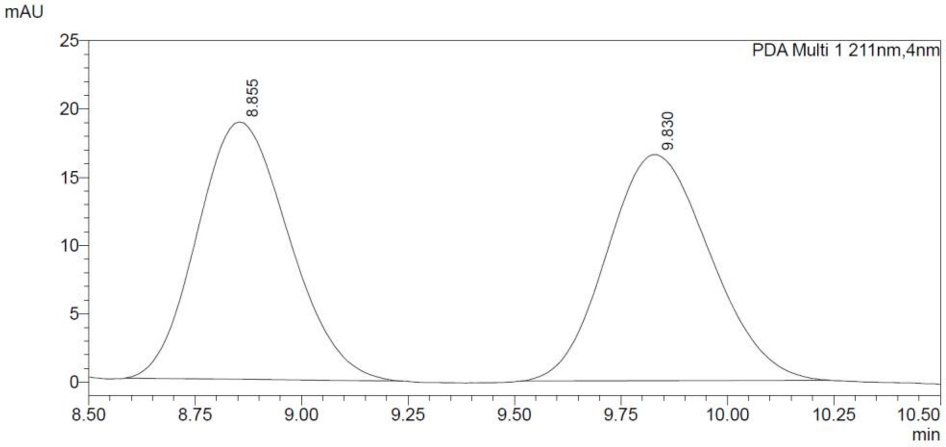



**<Peak Table>**

PDA Ch1 211nm

| Peak# | Ret. Time | Area% |
|---|---|---|
| 1 | 8.855 | 50.280 |
| 2 | 9.830 | 49.720 |
| Total | | 100.000 |

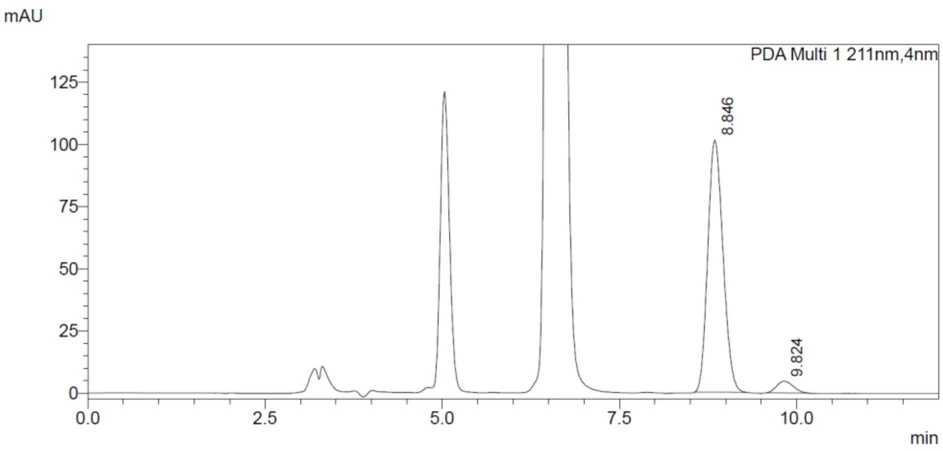


## <Peak Table>

PDA Ch1 211nm

| Peak# | Ret. Time | Area% |
|---|---|---|
| 1 | 8.846 | 95.222 |
| 2 | 9.824 | 4.778 |
| Total | | 100.000 |

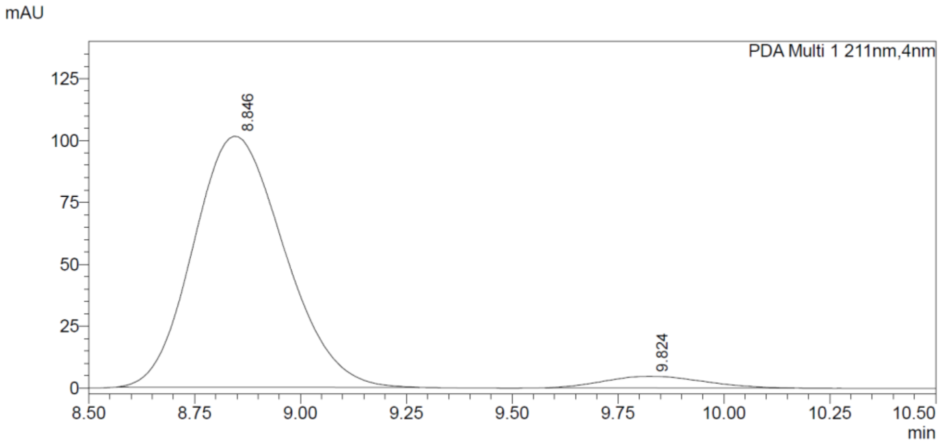


## <Peak Table>

PDA Ch1 211nm

| Peak# | Ret. Time | Area% |
|---|---|---|
| 1 | 8.846 | 95.222 |
| 2 | 9.824 | 4.778 |
| Total | | 100.000 |

HPLC Data for **17**: Chiralpak IB (99.3:0.7 hexane:IPA, flow rate 1.0 mLmin$^{-1}$, 254 nm, 30 °C), $t_R$ (minor): 8.1 min, $t_R$ (major): 9.1 min, 93:7 er.

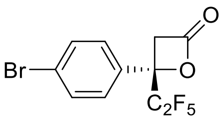


**17**

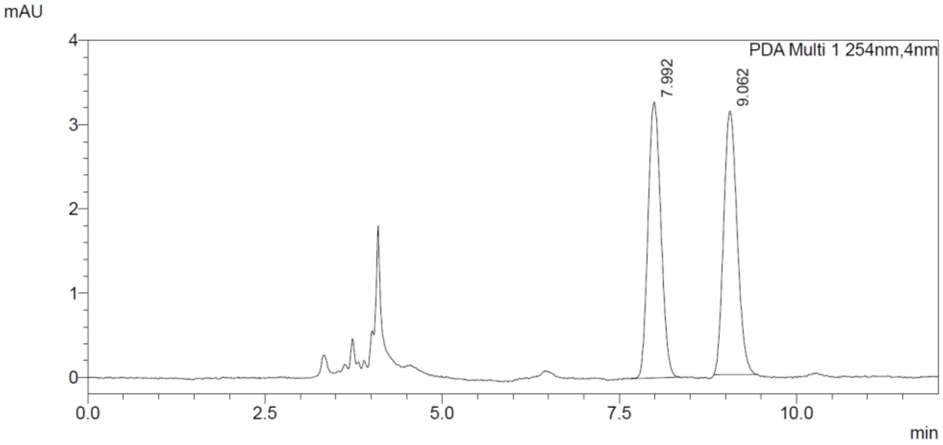


**<Peak Table>**

PDA Ch1 254nm

| Peak# | Ret. Time | Area% |
|---|---|---|
| 1 | 7.992 | 49.875 |
| 2 | 9.062 | 50.125 |
| Total | | 100.000 |

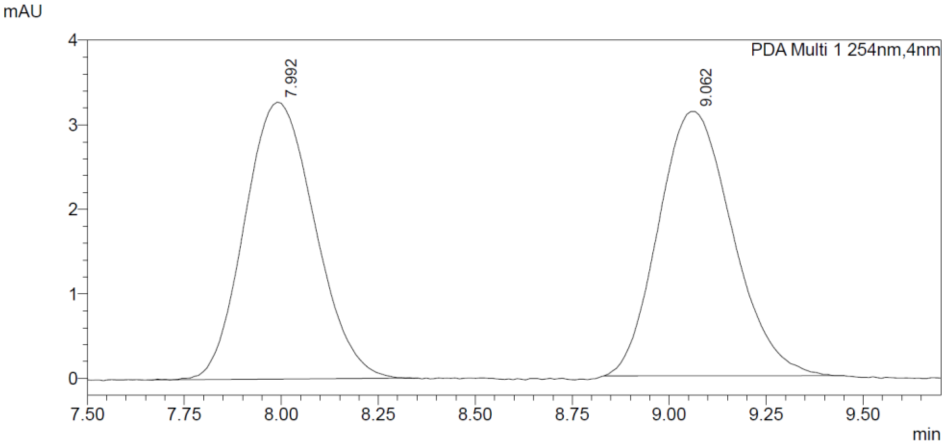


**<Peak Table>**

PDA Ch1 254nm

| Peak# | Ret. Time | Area% |
|---|---|---|
| 1 | 7.992 | 49.875 |
| 2 | 9.062 | 50.125 |
| Total | | 100.000 |

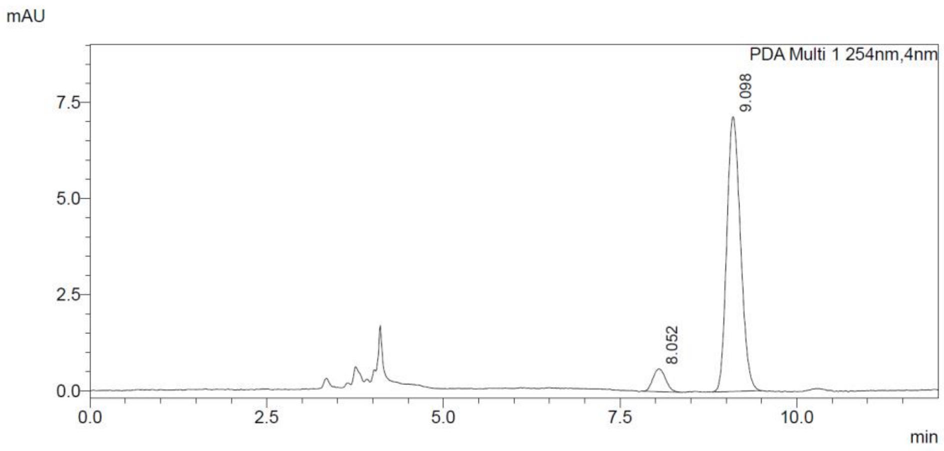


**<Peak Table>**

PDA Ch1 254nm

| Peak# | Ret. Time | Area% |
|---|---|---|
| 1 | 8.052 | 7.060 |
| 2 | 9.098 | 92.940 |
| Total | | 100.000 |

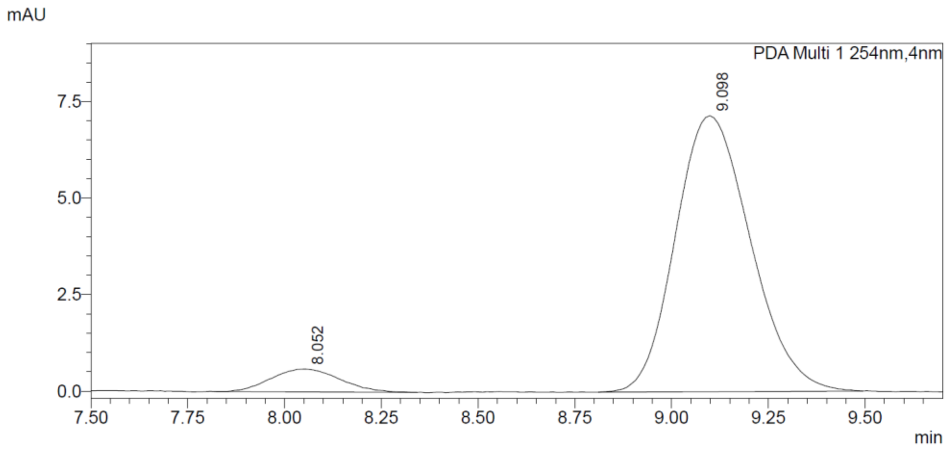


**<Peak Table>**

PDA Ch1 254nm

| Peak# | Ret. Time | Area% |
|---|---|---|
| 1 | 8.052 | 7.060 |
| 2 | 9.098 | 92.940 |
| Total | | 100.000 |

HPLC Data for **18**: Chiralpak AS-H (99.5:0.5 hexane:IPA, flow rate 1.0 mLmin$^{-1}$, 211 nm, 30 °C), $t_R$ (minor): 5.4 min, $t_R$ (major): 8.0 min, 87:13 er.

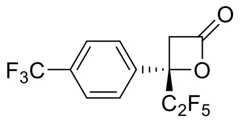


**18**

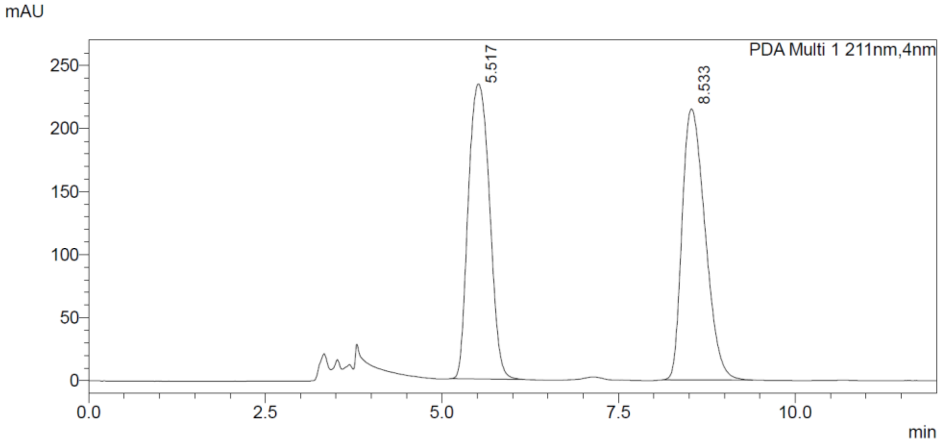


### <Peak Table>

PDA Ch1 211nm

| Peak# | Ret. Time | Area% |
|---|---|---|
| 1 | 5.517 | 50.069 |
| 2 | 8.533 | 49.931 |
| Total | | 100.000 |

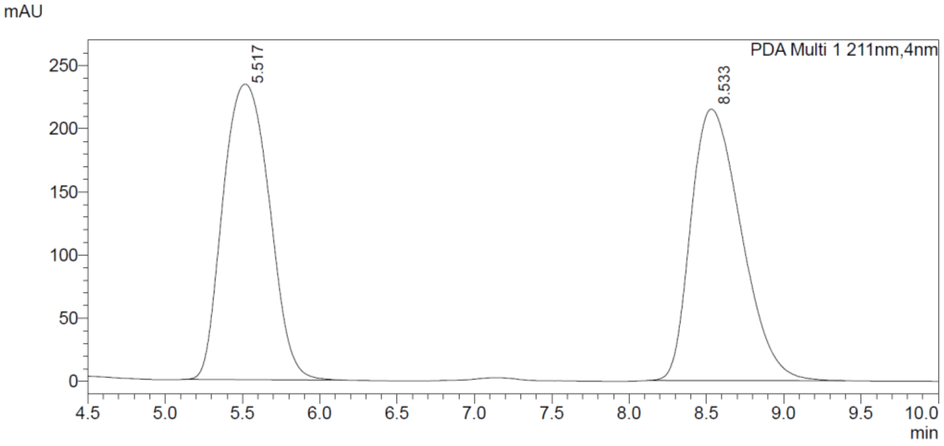


### <Peak Table>

PDA Ch1 211nm

| Peak# | Ret. Time | Area% |
|---|---|---|
| 1 | 5.517 | 50.069 |
| 2 | 8.533 | 49.931 |
| Total | | 100.000 |

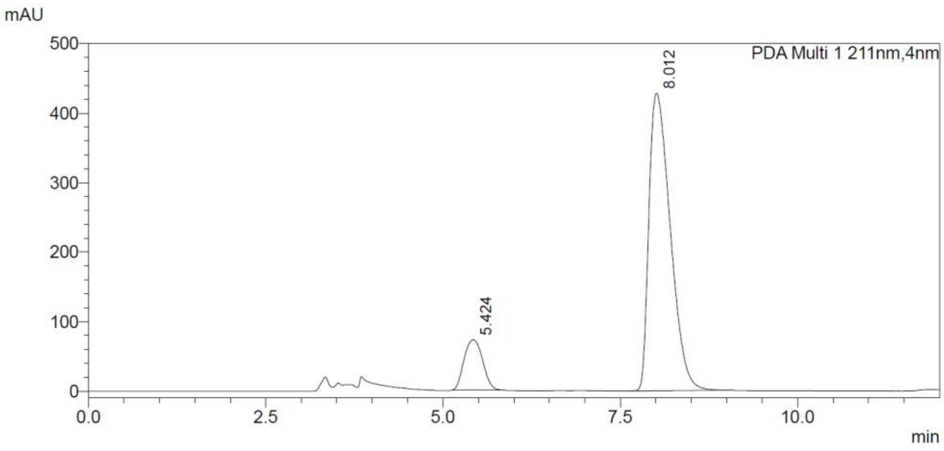


## <Peak Table>

PDA Ch1 211nm

| Peak# | Ret. Time | Area% |
|---|---|---|
| 1 | 5.424 | 12.927 |
| 2 | 8.012 | 87.073 |
| Total | | 100.000 |

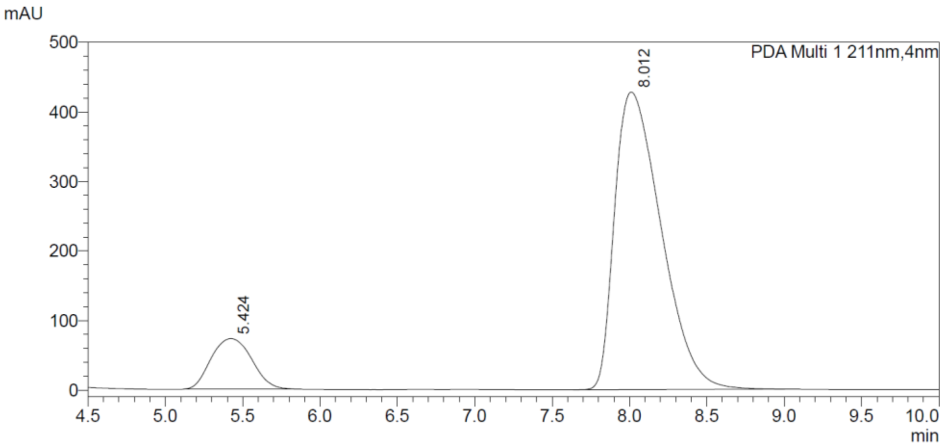


## <Peak Table>

PDA Ch1 211nm

| Peak# | Ret. Time | Area% |
|---|---|---|
| 1 | 5.424 | 12.927 |
| 2 | 8.012 | 87.073 |
| Total | | 100.000 |

HPLC Data for **19**: Chiralpak IB (99.3:0.7 hexane:IPA, flow rate 1.0 mLmin$^{-1}$, 254 nm, 30 °C), $t_R$ (minor): 6.6 min, $t_R$ (major): 7.2 min, 92:8 er.

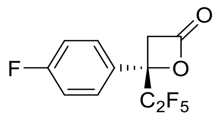


**19**

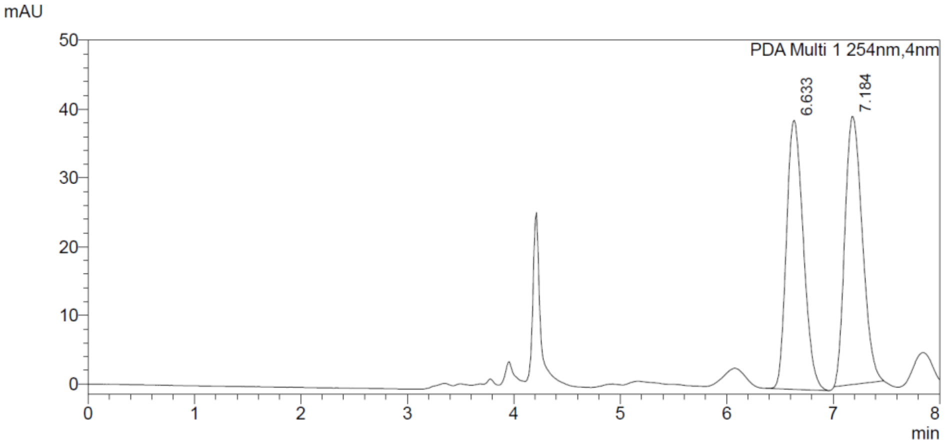


**<Peak Table>**

PDA Ch1 254nm

| Peak# | Ret. Time | Area% |
|---|---|---|
| 1 | 6.633 | 49.303 |
| 2 | 7.184 | 50.697 |
| Total | | 100.000 |

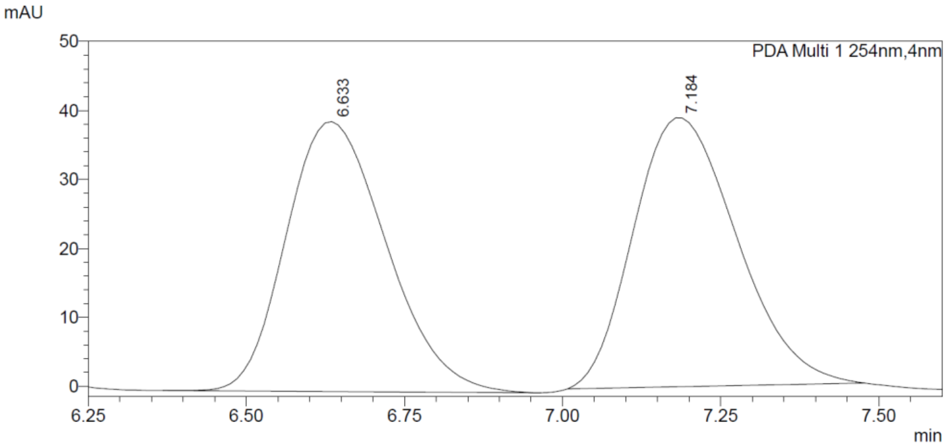


**<Peak Table>**

PDA Ch1 254nm

| Peak# | Ret. Time | Area% |
|---|---|---|
| 1 | 6.633 | 49.303 |
| 2 | 7.184 | 50.697 |
| Total | | 100.000 |

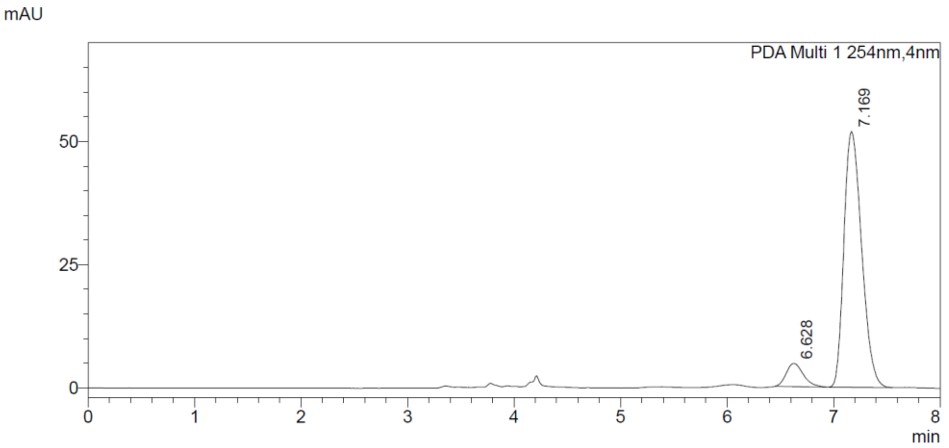


**<Peak Table>**

PDA Ch1 254nm

| Peak# | Ret. Time | Area% |
|---|---|---|
| 1 | 6.628 | 8.012 |
| 2 | 7.169 | 91.988 |
| Total | | 100.000 |

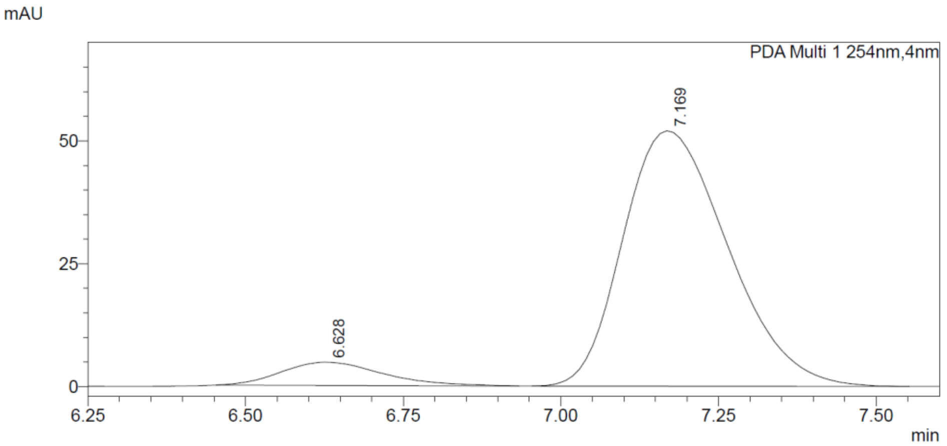


**<Peak Table>**

PDA Ch1 254nm

| Peak# | Ret. Time | Area% |
|---|---|---|
| 1 | 6.628 | 8.012 |
| 2 | 7.169 | 91.988 |
| Total | | 100.000 |

HPLC Data for **20**: Chiralpak IB (99.3:0.7 hexane:IPA, flow rate 1.0 mLmin$^{-1}$, 254 nm, 30 °C), $t_R$ (minor): 6.9 min, $t_R$ (major): 8.3 min, 88:12 er.

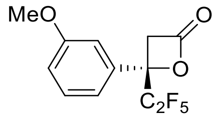


**20**

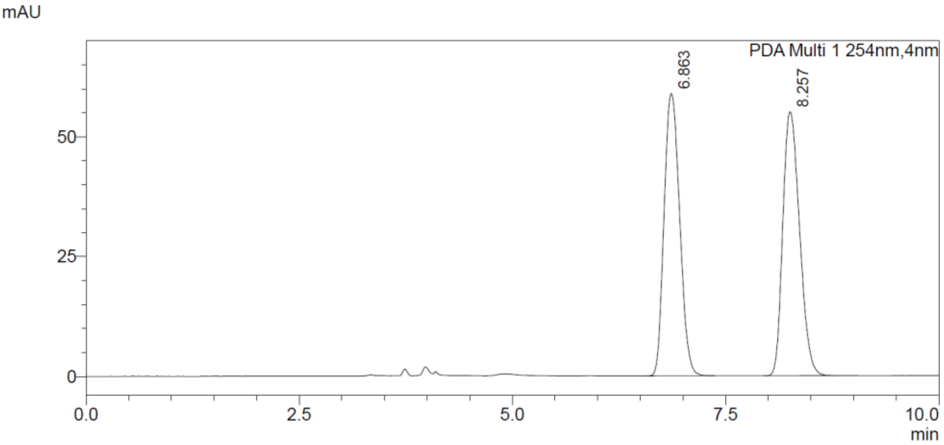


**<Peak Table>**

PDA Ch1 254nm

| Peak# | Ret. Time | Area% |
|---|---|---|
| 1 | 6.863 | 49.826 |
| 2 | 8.257 | 50.174 |
| Total | | 100.000 |

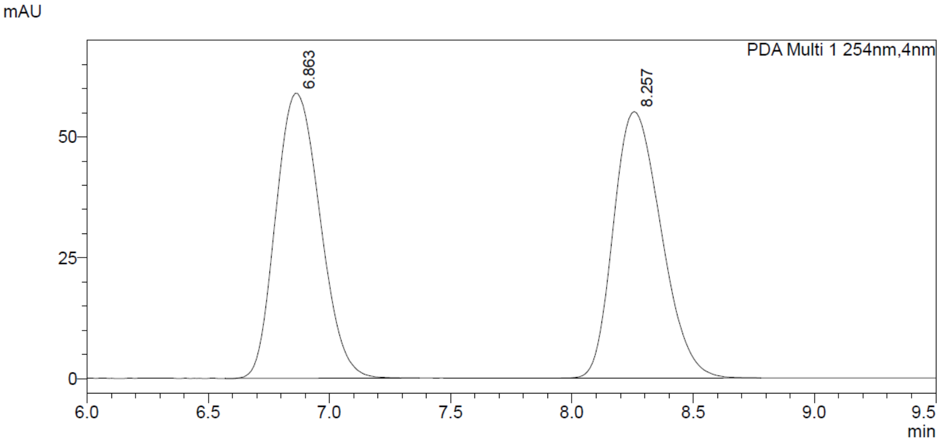


**<Peak Table>**

PDA Ch1 254nm

| Peak# | Ret. Time | Area% |
|---|---|---|
| 1 | 6.863 | 49.826 |
| 2 | 8.257 | 50.174 |
| Total | | 100.000 |

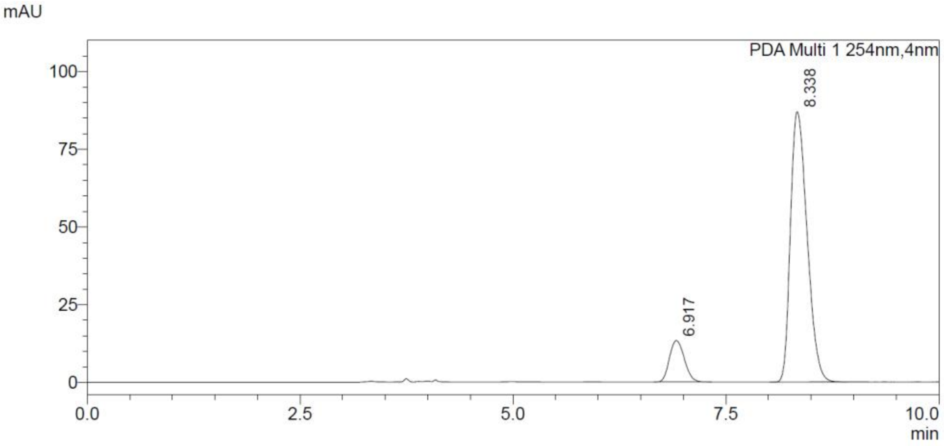


## <Peak Table>

PDA Ch1 254nm

| Peak# | Ret. Time | Area% |
|---|---|---|
| 1 | 6.917 | 12.190 |
| 2 | 8.338 | 87.810 |
| Total | | 100.000 |

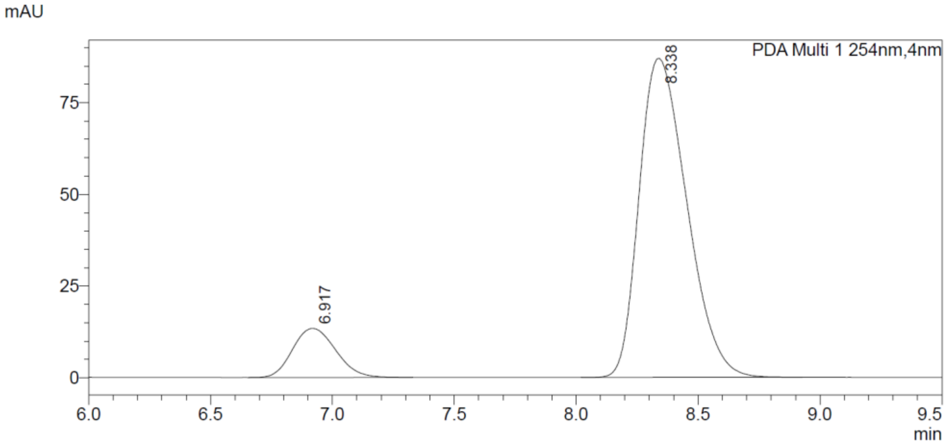


## <Peak Table>

PDA Ch1 254nm

| Peak# | Ret. Time | Area% |
|---|---|---|
| 1 | 6.917 | 12.190 |
| 2 | 8.338 | 87.810 |
| Total | | 100.000 |

HPLC Data for **21**: Chiralpak IB (99.3:0.7 hexane:IPA, flow rate 1.0 mLmin$^{-1}$, 254 nm, 30 °C), $t_R$ (minor): 4.9 min, $t_R$ (major): 5.6 min, 88:12 er**.**

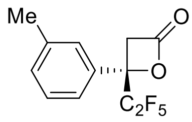


**21**

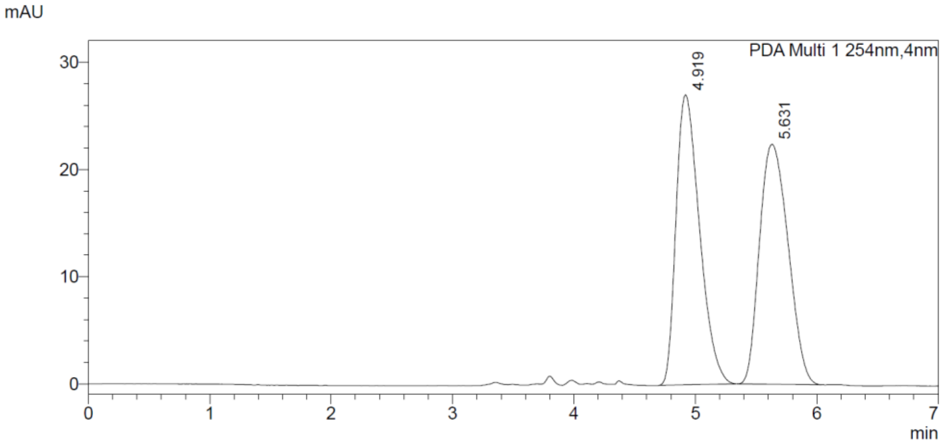


**<Peak Table>**

PDA Ch1 254nm

| Peak# | Ret. Time | Area% |
|---|---|---|
| 1 | 4.919 | 49.759 |
| 2 | 5.631 | 50.241 |
| Total | | 100.000 |

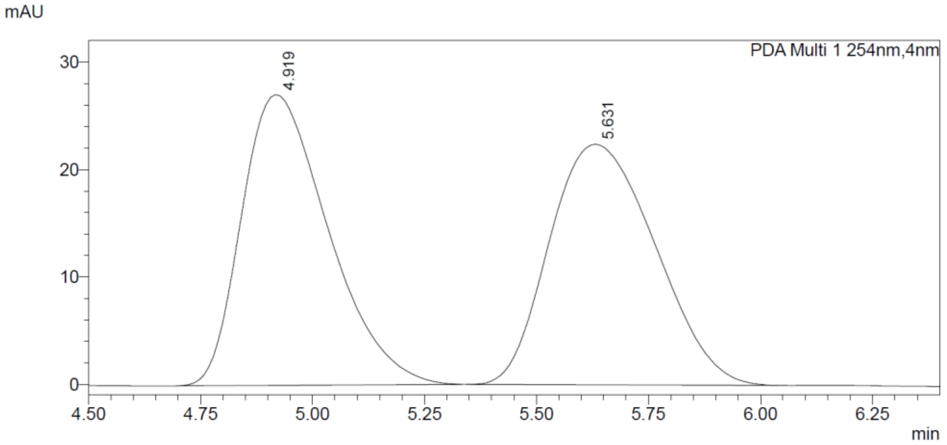


**<Peak Table>**

PDA Ch1 254nm

| Peak# | Ret. Time | Area% |
|---|---|---|
| 1 | 4.919 | 49.759 |
| 2 | 5.631 | 50.241 |
| Total | | 100.000 |

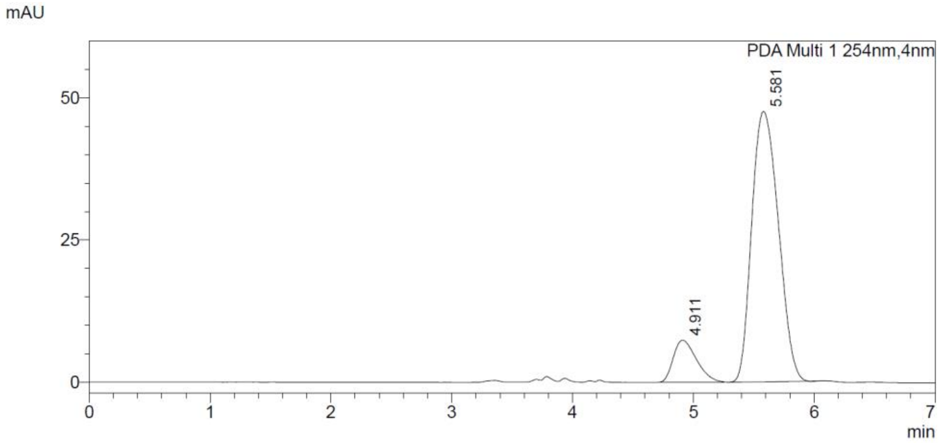


**<Peak Table>**

PDA Ch1 254nm

| Peak# | Ret. Time | Area% |
|---|---|---|
| 1 | 4.911 | 11.741 |
| 2 | 5.581 | 88.259 |
| Total | | 100.000 |

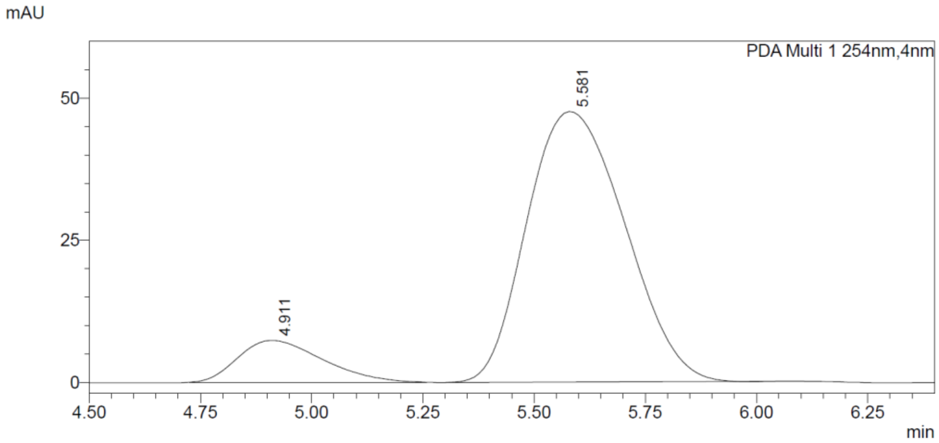


**<Peak Table>**

PDA Ch1 254nm

| Peak# | Ret. Time | Area% |
|---|---|---|
| 1 | 4.911 | 11.741 |
| 2 | 5.581 | 88.259 |
| Total | | 100.000 |

HPLC Data for **22**: Chiralpak IB (99.3:0.7 hexane:IPA, flow rate 1.0 mLmin$^{-1}$, 254 nm, 30 °C), $t_R$ (minor): 5.1 min, $t_R$ (major): 58 min, 88:12 er.

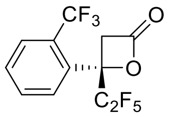


**22**

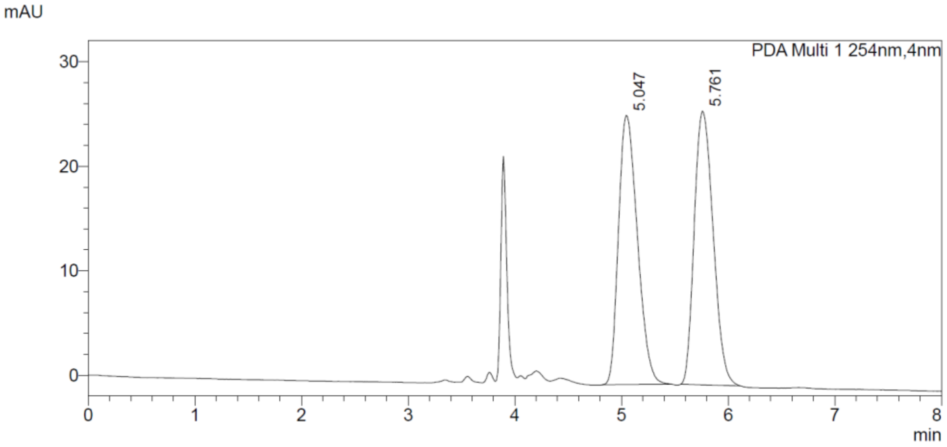


## <Peak Table>

PDA Ch1 254nm

| Peak# | Ret. Time | Area% |
|---|---|---|
| 1 | 5.047 | 49.629 |
| 2 | 5.761 | 50.371 |
| Total | | 100.000 |

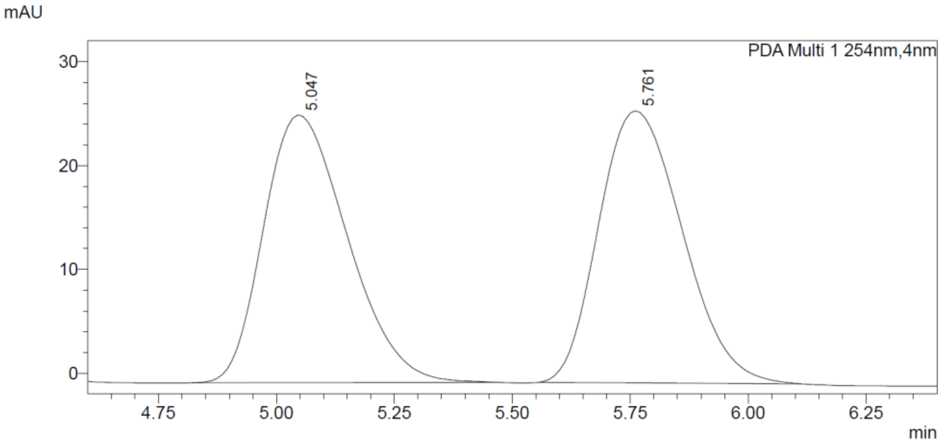


## <Peak Table>

PDA Ch1 254nm

| Peak# | Ret. Time | Area% |
|---|---|---|
| 1 | 5.047 | 49.629 |
| 2 | 5.761 | 50.371 |
| Total | | 100.000 |

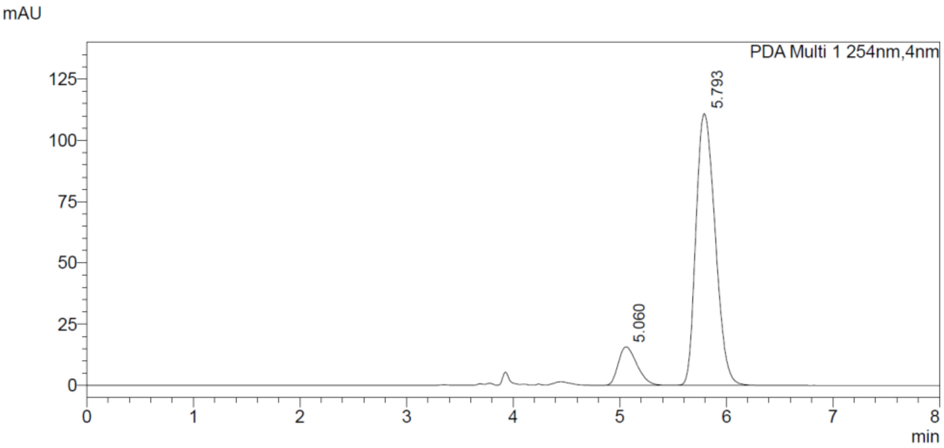


## <Peak Table>

PDA Ch1 254nm

| Peak# | Ret. Time | Area% |
|---|---|---|
| 1 | 5.060 | 11.864 |
| 2 | 5.793 | 88.136 |
| Total | | 100.000 |

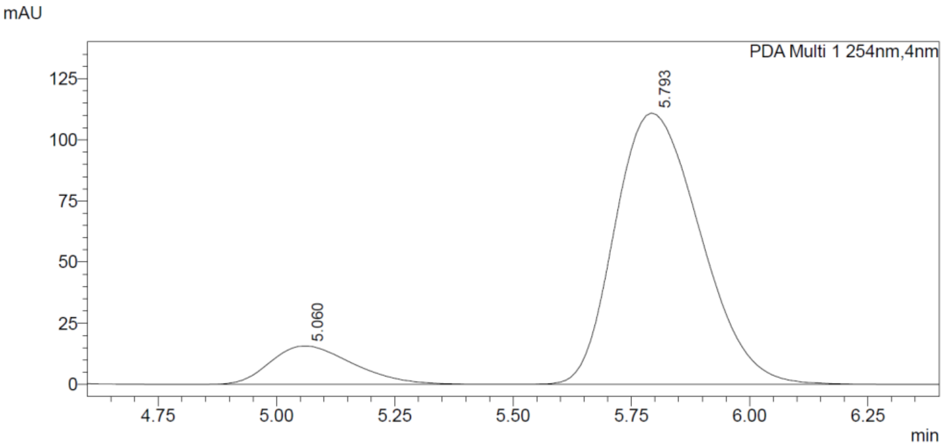



## <Peak Table>

PDA Ch1 254nm

| Peak# | Ret. Time | Area% |
|---|---|---|
| 1 | 5.060 | 11.864 |
| 2 | 5.793 | 88.136 |
| Total | | 100.000 |

HPLC Data for **23**: Chiralpak IB (99.3:0.7 hexane:IPA, flow rate 1.0 mLmin$^{-1}$, 254 nm, 30 °C), $t_R$ (minor): 5.1 min, $t_R$ (major): 6.0 min, 89:11 er**.**

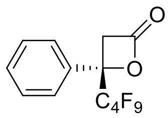

**23**

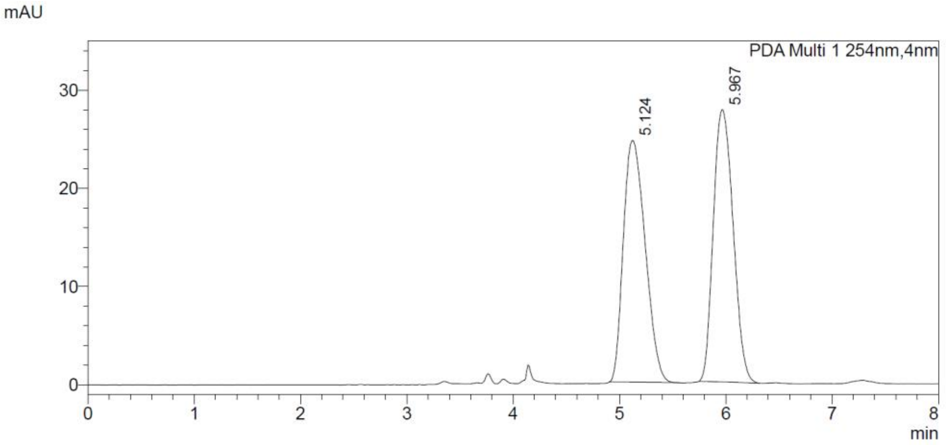


**<Peak Table>**

PDA Ch1 254nm

| Peak# | Ret. Time | Area% |
|---|---|---|
| 1 | 5.124 | 49.514 |
| 2 | 5.967 | 50.486 |
| Total | | 100.000 |

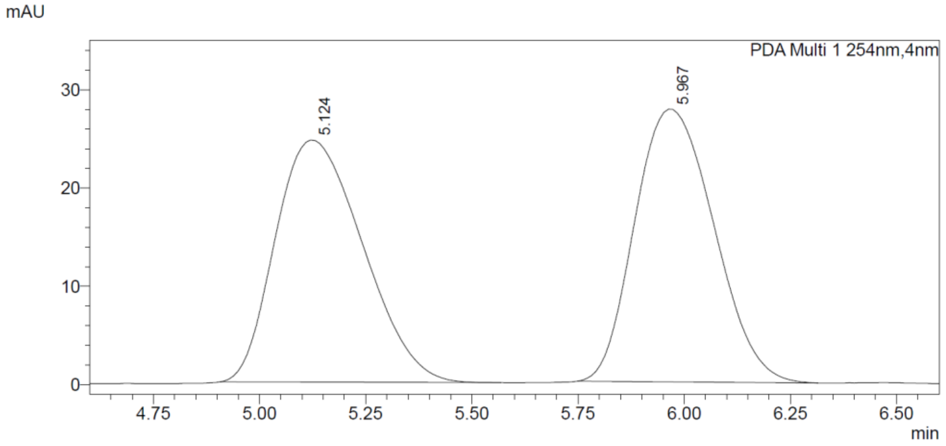


**<Peak Table>**

PDA Ch1 254nm

| Peak# | Ret. Time | Area% |
|---|---|---|
| 1 | 5.124 | 49.514 |
| 2 | 5.967 | 50.486 |
| Total | | 100.000 |

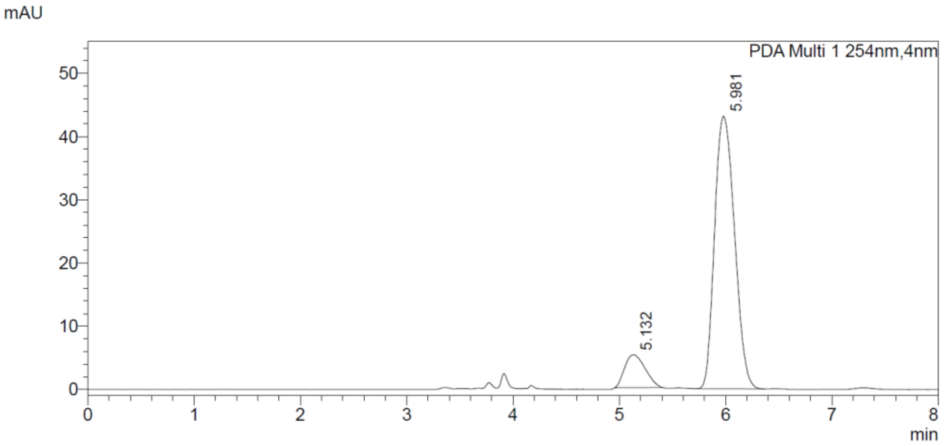


**<Peak Table>**

PDA Ch1 254nm

| Peak# | Ret. Time | Area% |
|---|---|---|
| 1 | 5.132 | 11.000 |
| 2 | 5.981 | 89.000 |
| Total | | 100.000 |

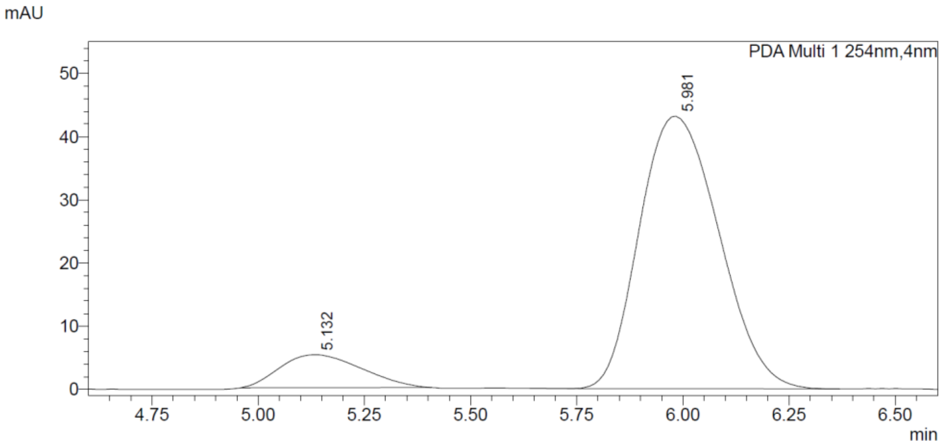


**<Peak Table>**

PDA Ch1 254nm

| Peak# | Ret. Time | Area% |
|---|---|---|
| 1 | 5.132 | 11.000 |
| 2 | 5.981 | 89.000 |
| Total | | 100.000 |

HPLC Data for **24**: Chiralpak IB (99.3:0.7 hexane:IPA, flow rate 1.0 mLmin$^{-1}$, 254 nm, 30 °C), $t_R$ (minor): 7.2 min, $t_R$ (major): 8.5 min, 86:14 er.

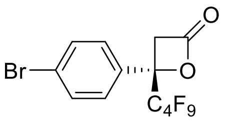


**24**

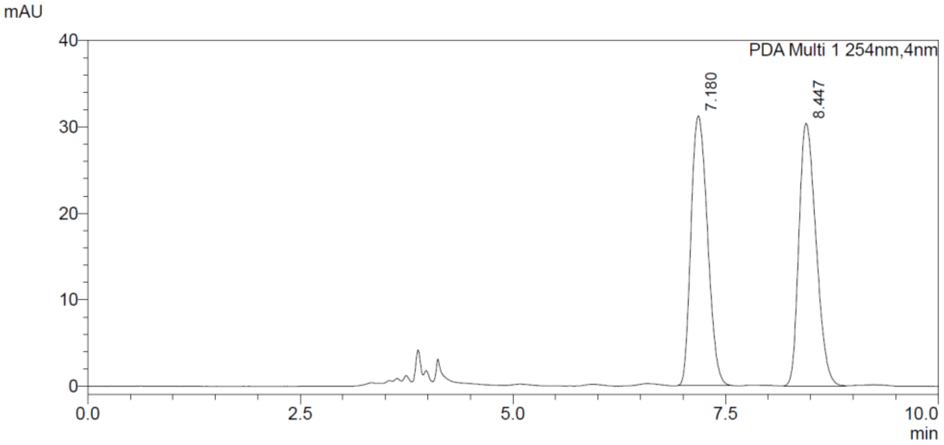


**<Peak Table>**

PDA Ch1 254nm

| Peak# | Ret. Time | Area% |
|---|---|---|
| 1 | 7.180 | 49.779 |
| 2 | 8.447 | 50.221 |
| Total | | 100.000 |

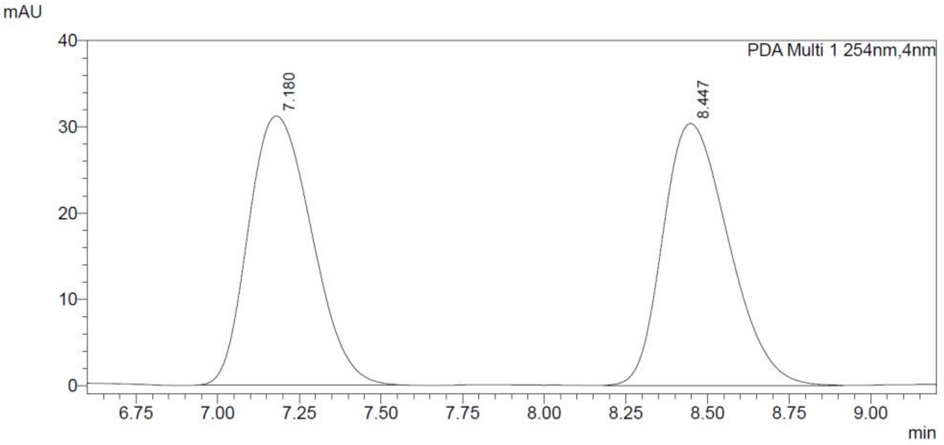


**<Peak Table>**

PDA Ch1 254nm

| Peak# | Ret. Time | Area% |
|---|---|---|
| 1 | 7.180 | 49.779 |
| 2 | 8.447 | 50.221 |
| Total | | 100.000 |

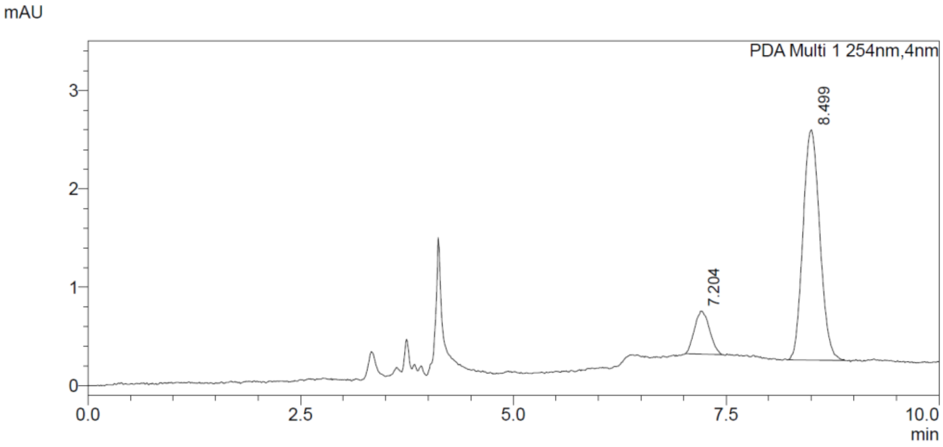


## <Peak Table>

PDA Ch1 254nm

| Peak# | Ret. Time | Area% |
|---|---|---|
| 1 | 7.204 | 14.276 |
| 2 | 8.499 | 85.724 |
| Total | | 100.000 |

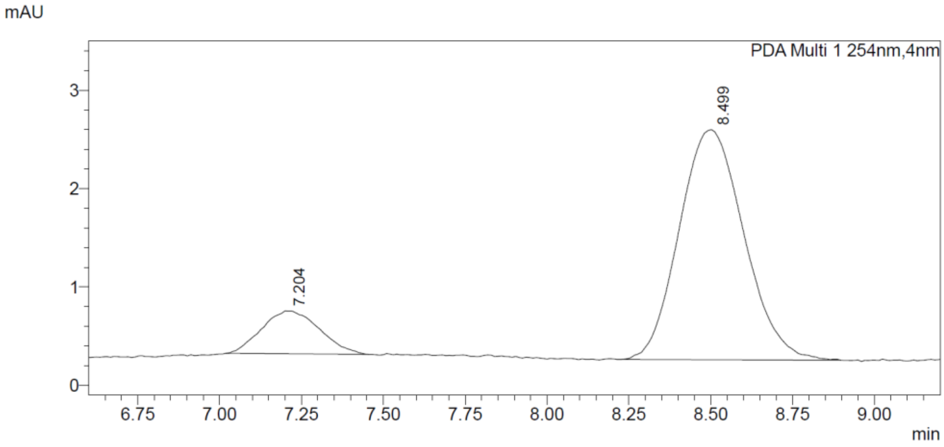


## <Peak Table>

PDA Ch1 254nm

| Peak# | Ret. Time | Area% |
|---|---|---|
| 1 | 7.204 | 14.276 |
| 2 | 8.499 | 85.724 |
| Total | | 100.000 |

HPLC Data for **25**: Chiralpak AS-H (99.5:0.5 hexane:IPA, flow rate 1.0 $mLmin^{-1}$, 211 nm, 30 °C), $t_R$ (minor): 5.1 min, $t_R$ (major): 6.1 min, >99:1 er.

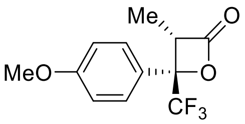


**25**

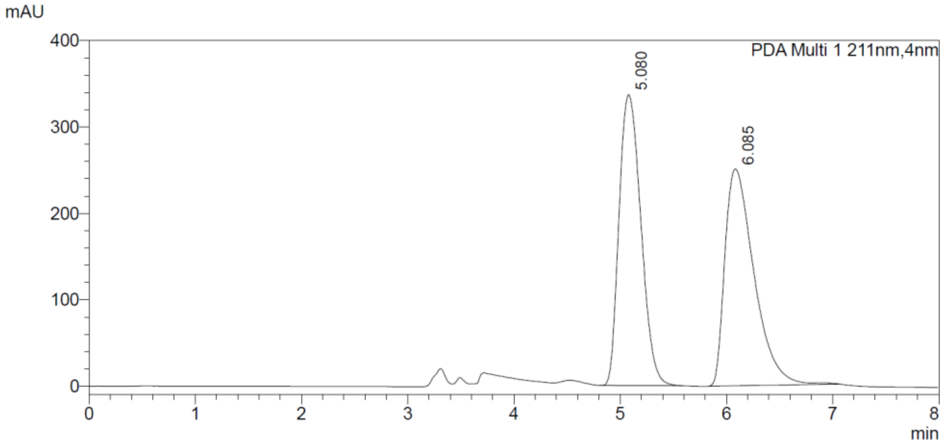


**<Peak Table>**

PDA Ch1 211nm

| Peak# | Ret. Time | Area% |
|---|---|---|
| 1 | 5.080 | 50.136 |
| 2 | 6.085 | 49.864 |
| Total | | 100.000 |

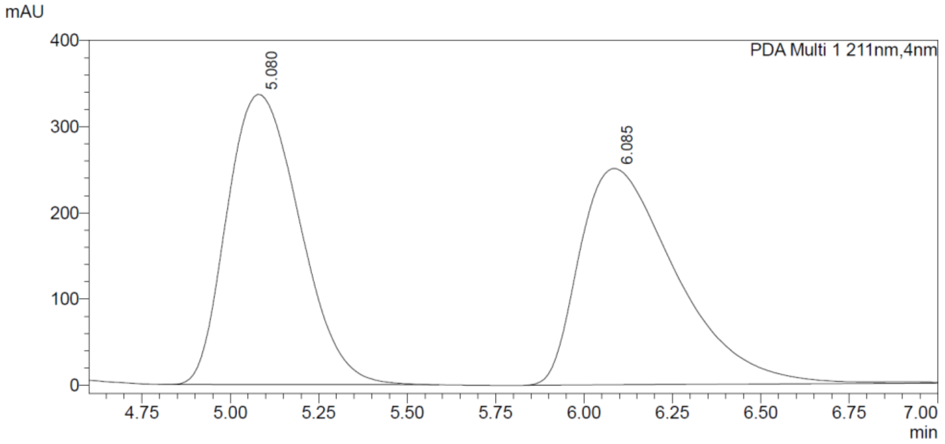


**<Peak Table>**

PDA Ch1 211nm

| Peak# | Ret. Time | Area% |
|---|---|---|
| 1 | 5.080 | 50.136 |
| 2 | 6.085 | 49.864 |
| Total | | 100.000 |

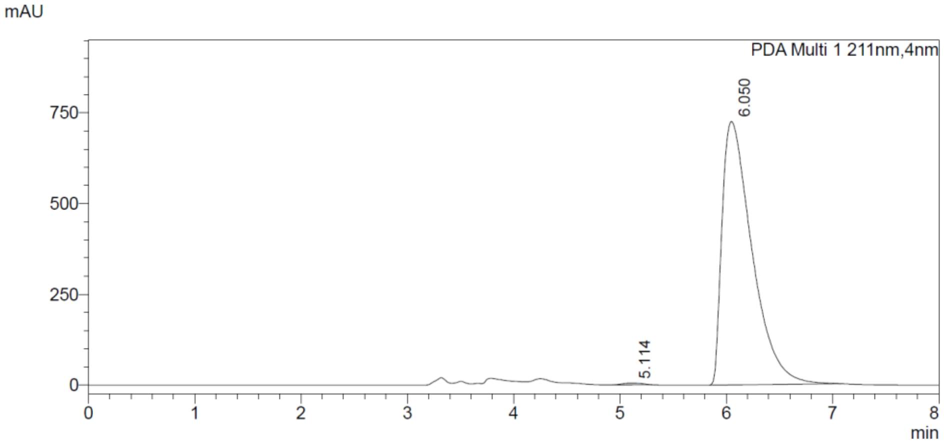


**<Peak Table>**

PDA Ch1 211nm

| Peak# | Ret. Time | Area% |
|---|---|---|
| 1 | 5.114 | 0.431 |
| 2 | 6.050 | 99.569 |
| Total | | 100.000 |

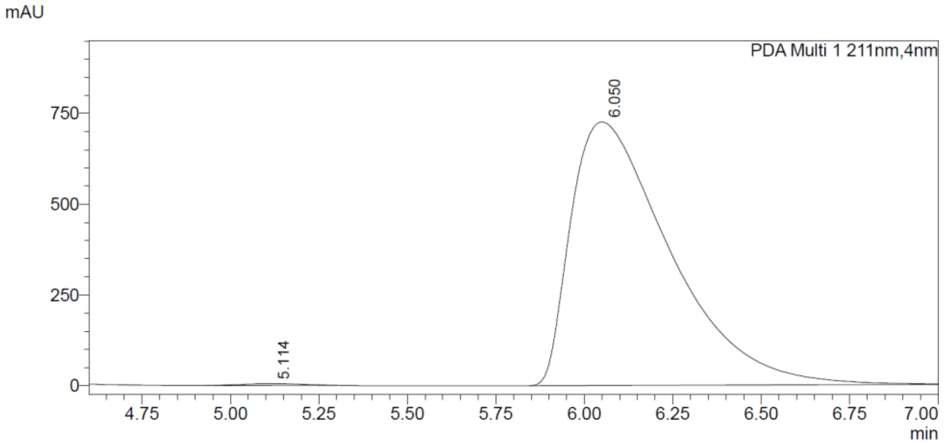


**<Peak Table>**

PDA Ch1 211nm

| Peak# | Ret. Time | Area% |
|---|---|---|
| 1 | 5.114 | 0.431 |
| 2 | 6.050 | 99.569 |
| Total | | 100.000 |

HPLC Data for **26**: Chiralpak AS-H (99.5:0.5 hexane:IPA, flow rate 1.0 mLmin$^{-1}$, 211 nm, 30 °C), $t_R$ (minor): 7.0 min, $t_R$ (major): 8.1 min, >99:1 er.

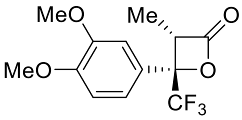


**26**

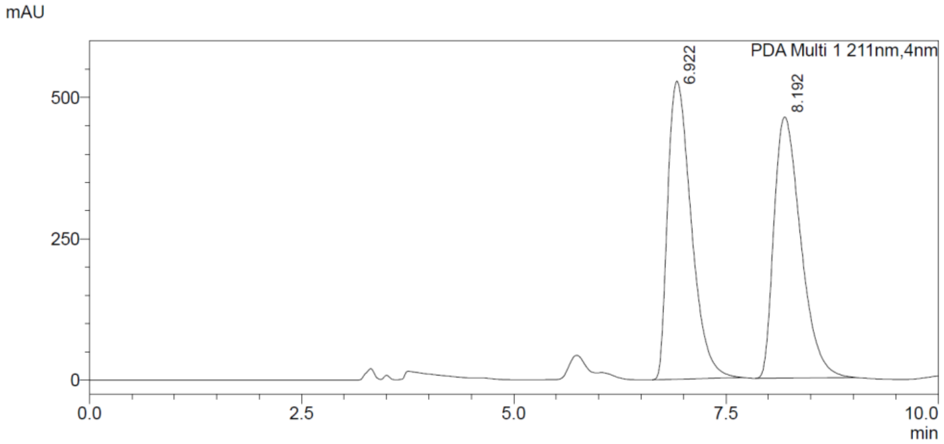


**<Peak Table>**

PDA Ch1 211nm

| Peak# | Ret. Time | Area% |
|---|---|---|
| 1 | 6.922 | 50.007 |
| 2 | 8.192 | 49.993 |
| Total | | 100.000 |

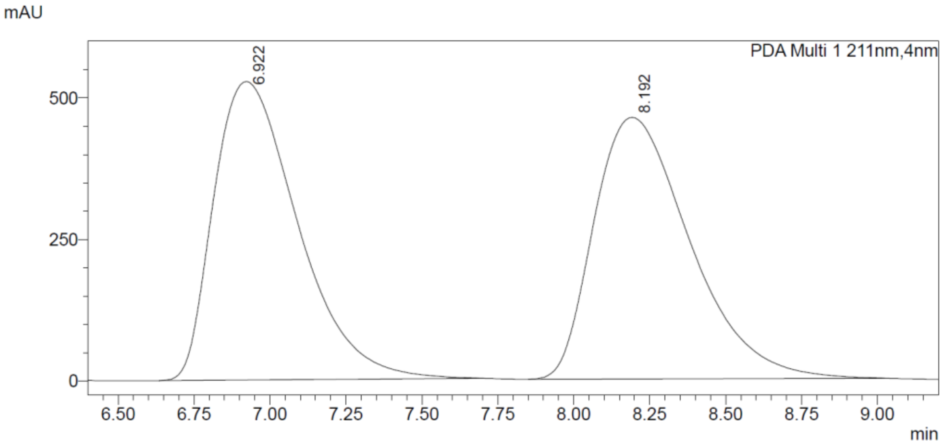


**<Peak Table>**


PDA Ch1 211nm

| Peak# | Ret. Time | Area% |
|---|---|---|
| 1 | 6.922 | 50.007 |
| 2 | 8.192 | 49.993 |
| Total | | 100.000 |

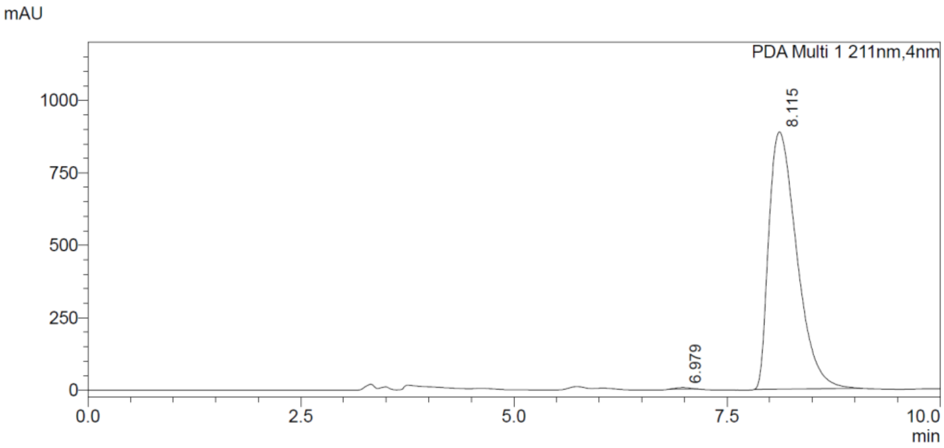


**<Peak Table>**

PDA Ch1 211nm

| Peak# | Ret. Time | Area% |
|---|---|---|
| 1 | 6.979 | 0.295 |
| 2 | 8.115 | 99.705 |
| Total | | 100.000 |

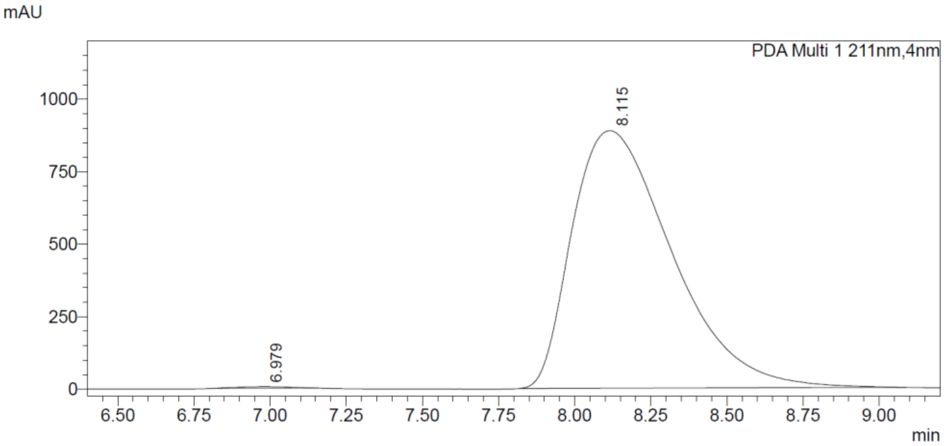


**<Peak Table>**

PDA Ch1 211nm

| Peak# | Ret. Time | Area% |
|---|---|---|
| 1 | 6.979 | 0.295 |
| 2 | 8.115 | 99.705 |
| Total | | 100.000 |

HPLC Data for **27**: Chiralcel OJ-H (99.8:0.2 hexane:IPA, flow rate 1.0 mLmin$^{-1}$, 211 nm, 30 °C), $t_R$ (minor): 6.2 min, $t_R$ (major): 7.7 min, >99:1 er.

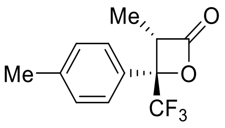


**27**

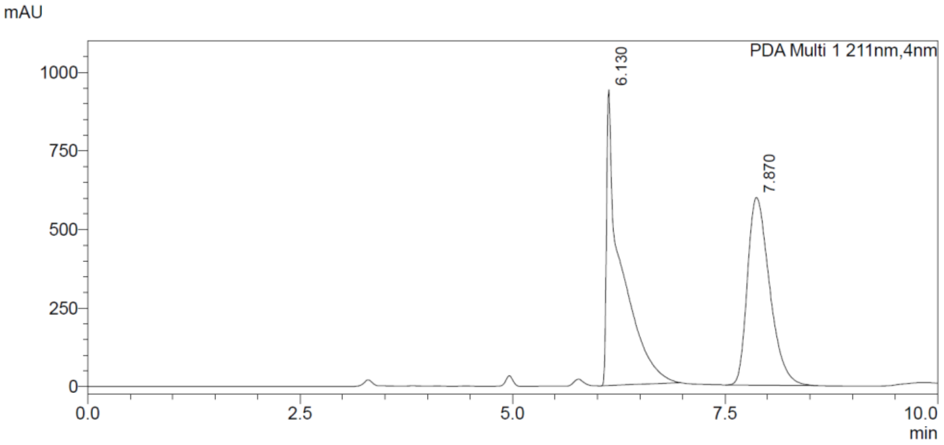


**<Peak Table>**

PDA Ch1 211nm

| Peak# | Ret. Time | Area% |
|---|---|---|
| 1 | 6.130 | 50.460 |
| 2 | 7.870 | 49.540 |
| Total | | 100.000 |

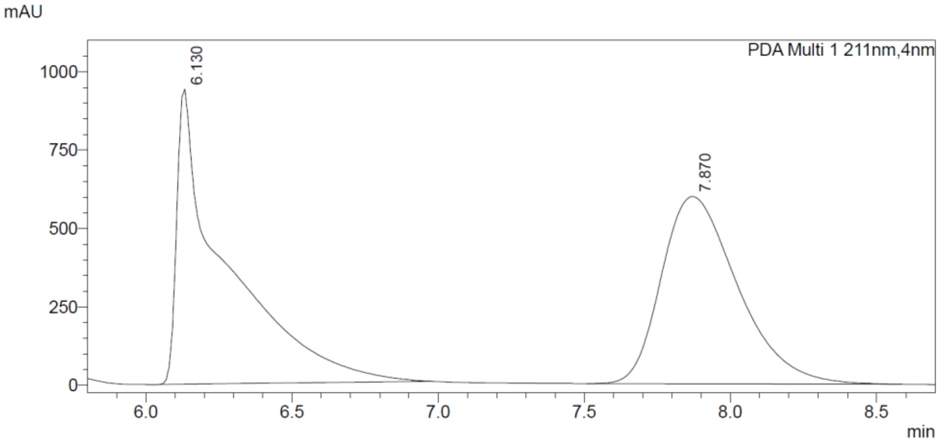


**<Peak Table>**

PDA Ch1 211nm

| Peak# | Ret. Time | Area% |
|---|---|---|
| 1 | 6.130 | 50.460 |
| 2 | 7.870 | 49.540 |
| Total | | 100.000 |

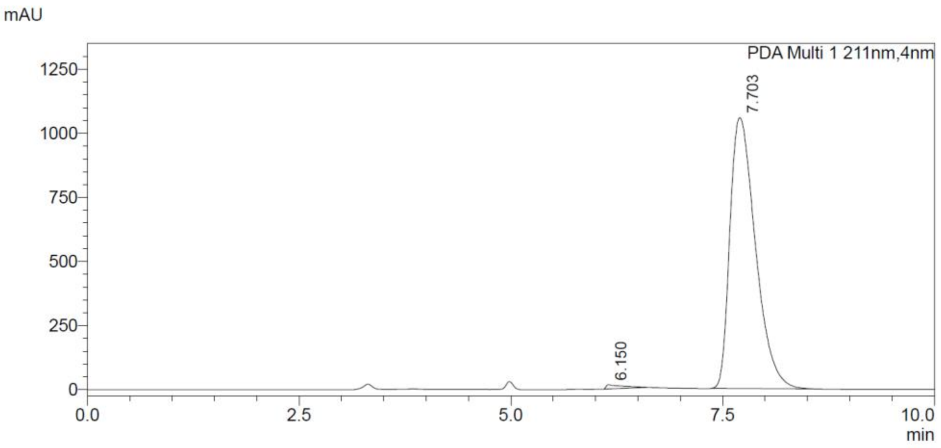


**<Peak Table>**

PDA Ch1 211nm

| Peak# | Ret. Time | Area% |
|---|---|---|
| 1 | 6.150 | 0.949 |
| 2 | 7.703 | 99.051 |
| Total | | 100.000 |

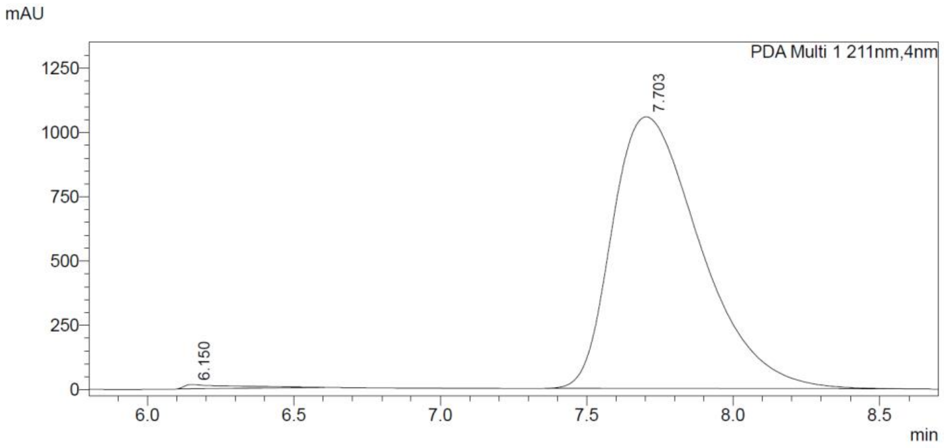


**<Peak Table>**

PDA Ch1 211nm

| Peak# | Ret. Time | Area% |
|---|---|---|
| 1 | 6.150 | 0.949 |
| 2 | 7.703 | 99.051 |
| Total | | 100.000 |

HPLC Data for **28**: Chiralcel OJ-H (99.8:0.2 hexane:IPA, flow rate 1.0 mLmin$^{-1}$, 211 nm, 30 °C), $t_R$ (minor): 8.8 min, $t_R$ (major): 11.0 min, >99:1 er.

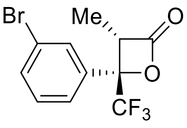


**28**

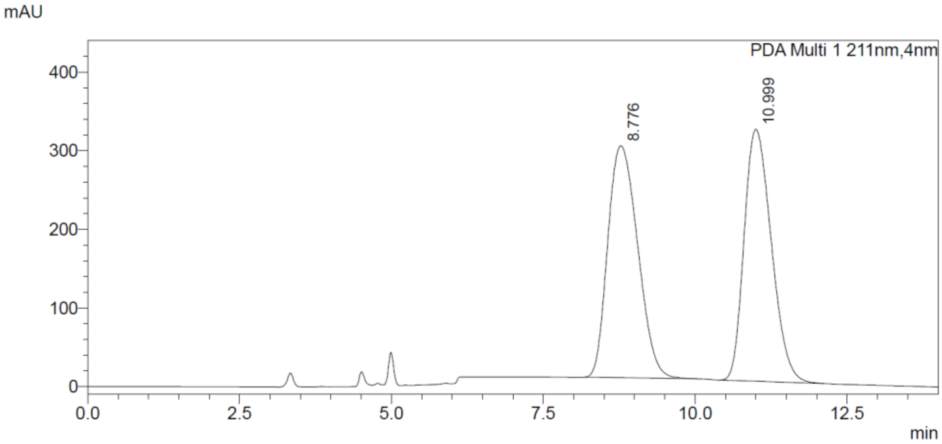


**<Peak Table>**

PDA Ch1 211nm

| Peak# | Ret. Time | Area% |
|---|---|---|
| 1 | 8.776 | 50.316 |
| 2 | 10.999 | 49.684 |
| Total | | 100.000 |

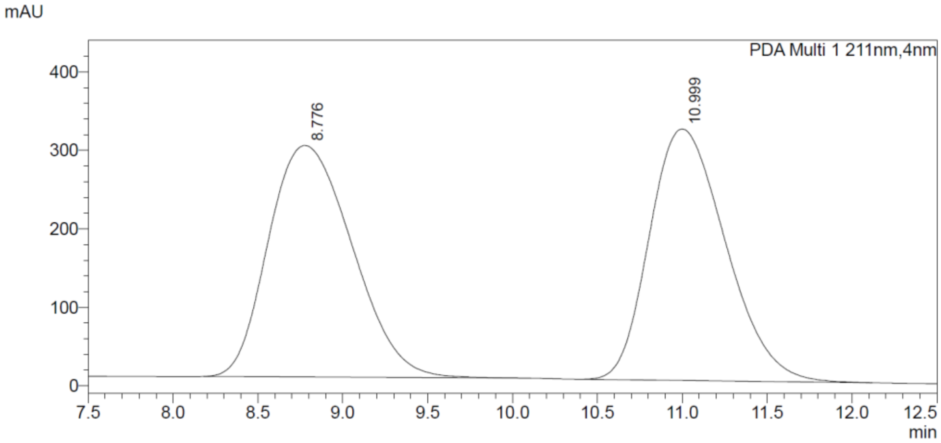


**<Peak Table>**

PDA Ch1 211nm

| Peak# | Ret. Time | Area% |
|---|---|---|
| 1 | 8.776 | 50.316 |
| 2 | 10.999 | 49.684 |
| Total | | 100.000 |

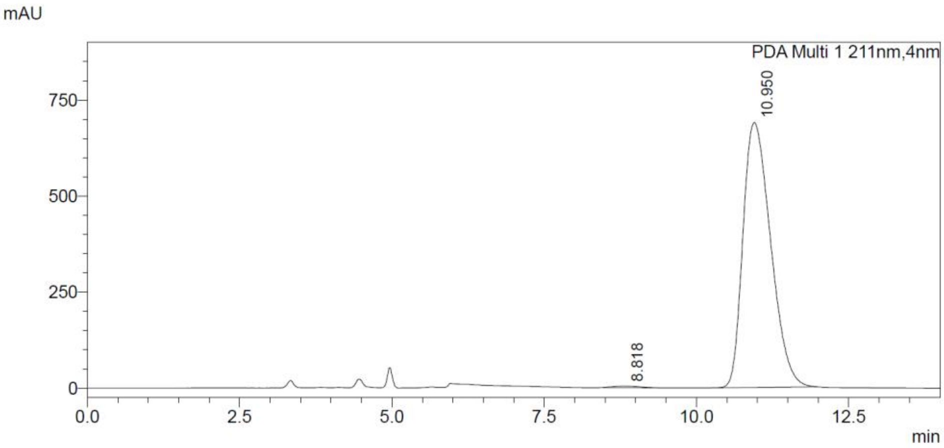


## <Peak Table>

PDA Ch1 211nm

| Peak# | Ret. Time | Area% |
|---|---|---|
| 1 | 8.818 | 0.464 |
| 2 | 10.950 | 99.536 |
| Total | | 100.000 |

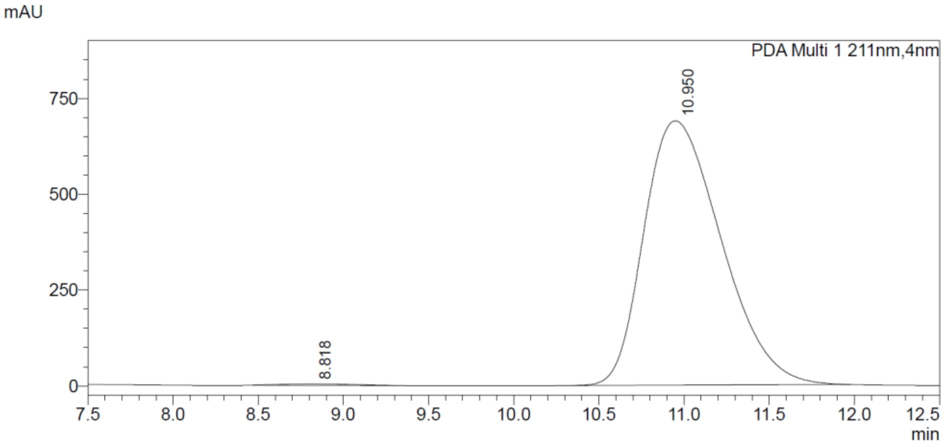



## <Peak Table>

PDA Ch1 211nm

| Peak# | Ret. Time | Area% |
|---|---|---|
| 1 | 8.818 | 0.464 |
| 2 | 10.950 | 99.536 |
| Total | | 100.000 |

HPLC Data for **29**: Chiralpak IB (99.9:0.1 hexane:IPA, flow rate 1.0 mLmin$^{-1}$, 254 nm, 30 °C), $t_R$ (major): 5.9 min, >99:1 er.

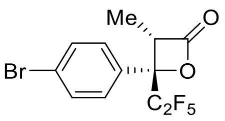


**29**

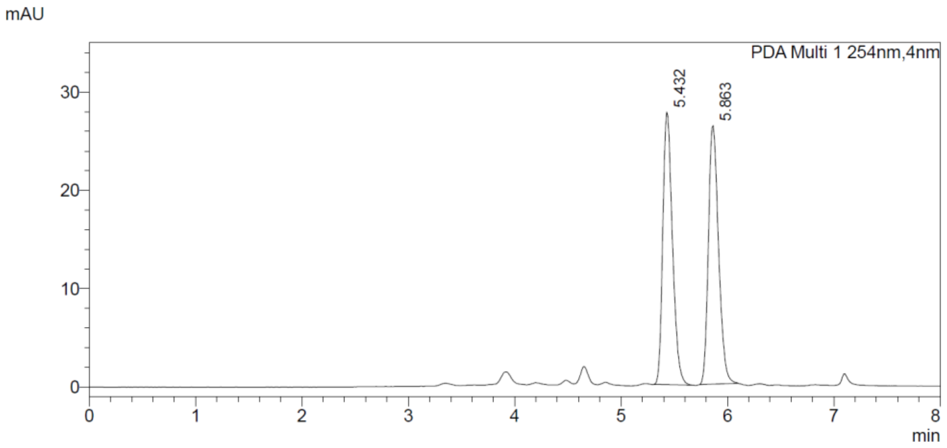


**<Peak Table>**

PDA Ch1 254nm

| Peak# | Ret. Time | Area% |
|---|---|---|
| 1 | 5.432 | 49.957 |
| 2 | 5.863 | 50.043 |
| Total | | 100.000 |

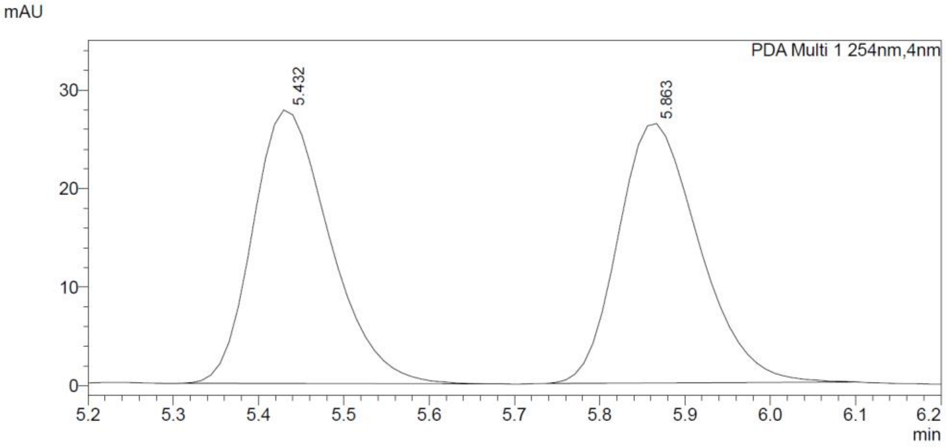


**<Peak Table>**

PDA Ch1 254nm

| Peak# | Ret. Time | Area% |
|---|---|---|
| 1 | 5.432 | 49.957 |
| 2 | 5.863 | 50.043 |
| Total | | 100.000 |

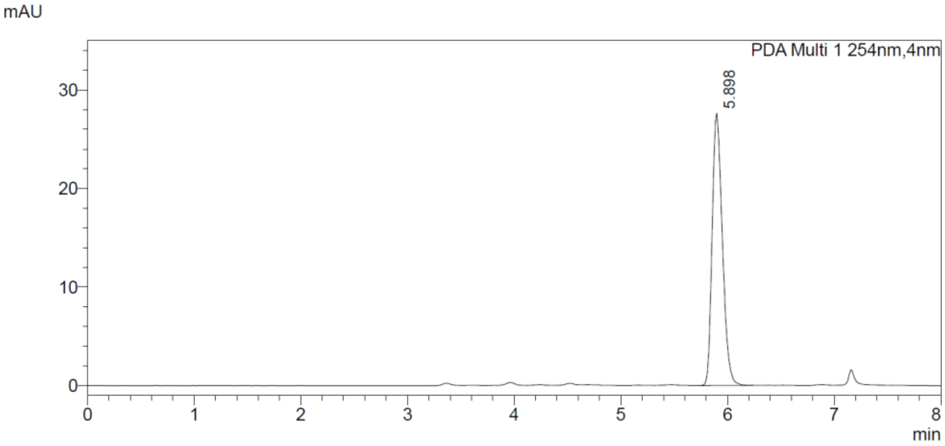


**<Peak Table>**

PDA Ch1 254nm

| Peak# | Ret. Time | Area% |
|---|---|---|
| 1 | 5.898 | 100.000 |
| Total | | 100.000 |

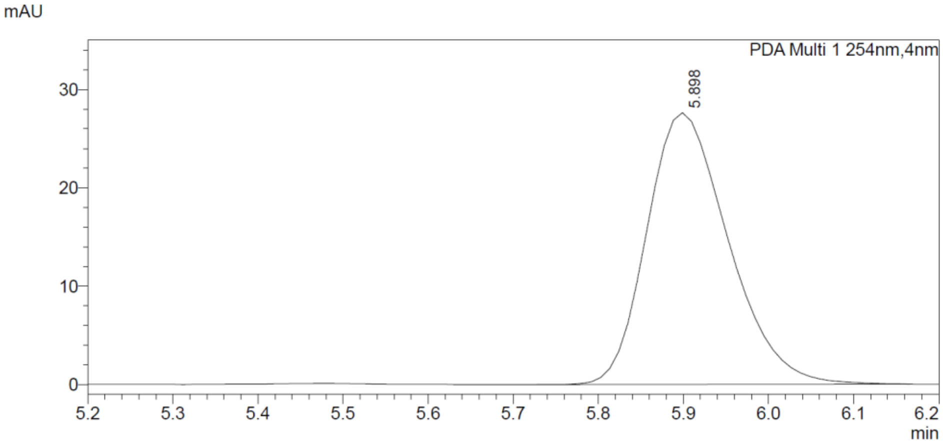


**<Peak Table>**

PDA Ch1 254nm

| Peak# | Ret. Time | Area% |
|---|---|---|
| 1 | 5.898 | 100.000 |
| Total | | 100.000 |

HPLC Data for **30**: Chiralpak AS-H (99.9:0.1 hexane:IPA, flow rate 1.0 mLmin$^{-1}$, 254 nm, 30 °C), $t_R$ (minor): 4.1 min, $t_R$ (major): 4.4 min, 98:2 er.

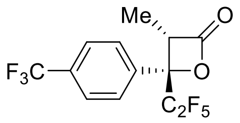


**30**

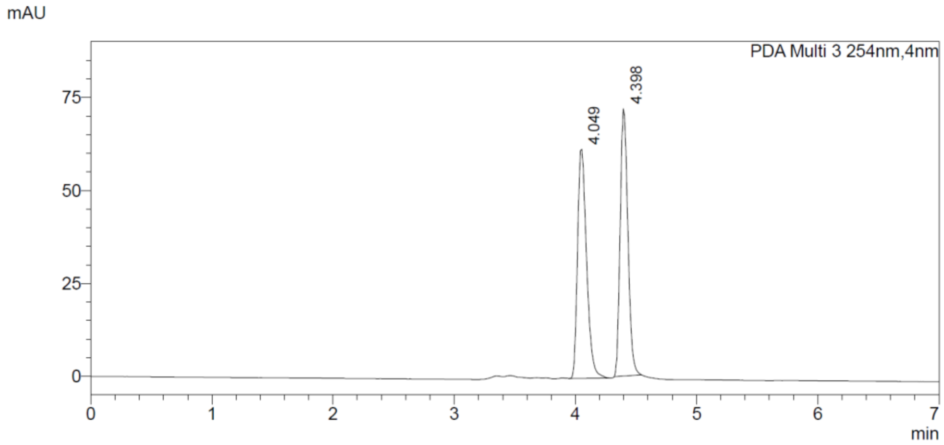


**<Peak Table>**

PDA Ch3 254nm

| Peak# | Ret. Time | Area% |
|---|---|---|
| 1 | 4.049 | 49.926 |
| 2 | 4.398 | 50.074 |
| Total | | 100.000 |

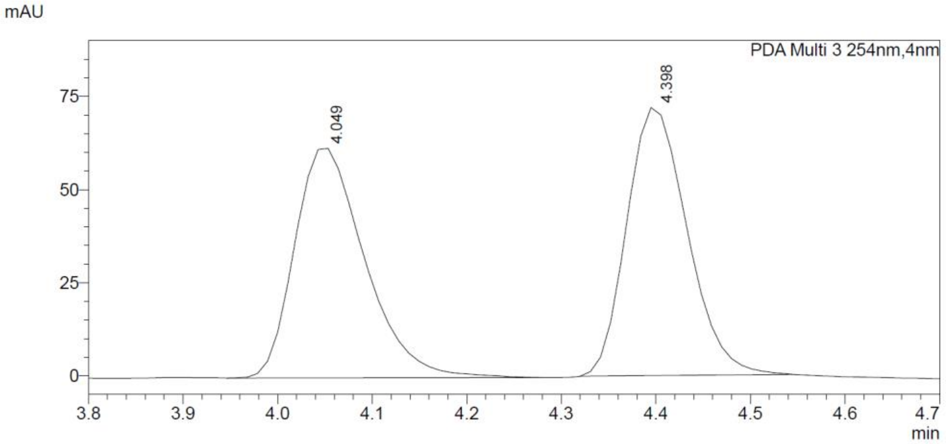



**<Peak Table>**

PDA Ch3 254nm

| Peak# | Ret. Time | Area% |
|---|---|---|
| 1 | 4.049 | 49.926 |
| 2 | 4.398 | 50.074 |
| Total | | 100.000 |

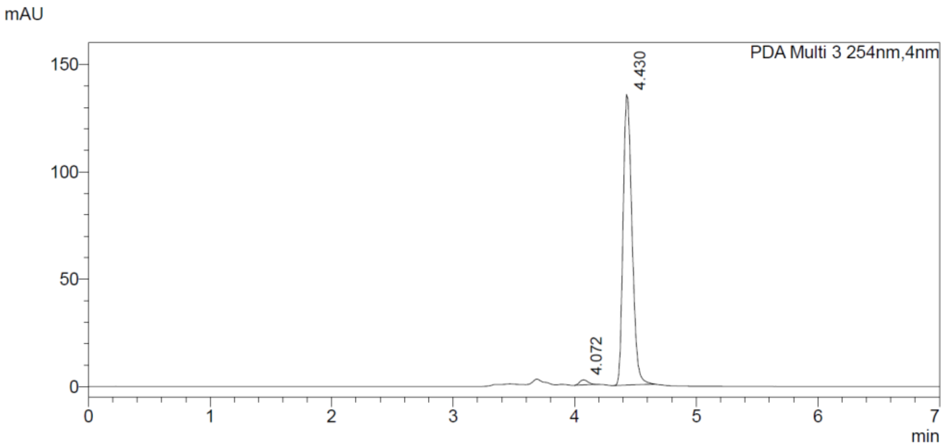


**<Peak Table>**

PDA Ch3 254nm

| Peak# | Ret. Time | Area% |
|---|---|---|
| 1 | 4.072 | 1.640 |
| 2 | 4.430 | 98.360 |
| Total | | 100.000 |

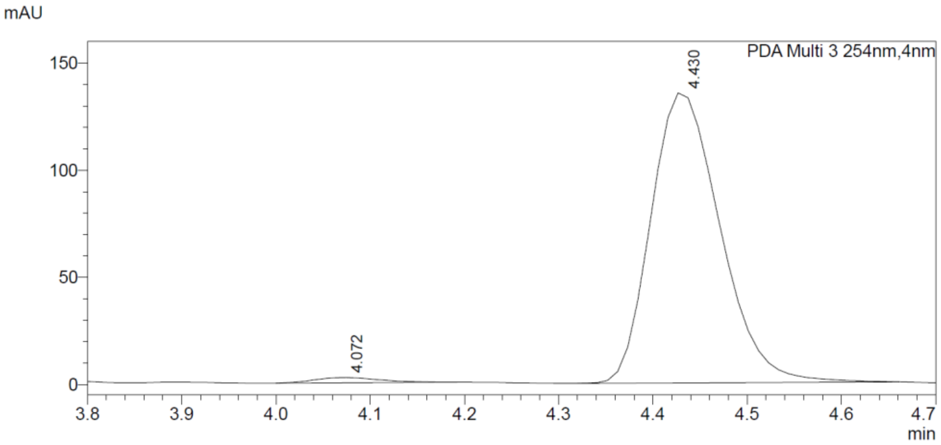


**<Peak Table>**

PDA Ch3 254nm

| Peak# | Ret. Time | Area% |
|---|---|---|
| 1 | 4.072 | 1.640 |
| 2 | 4.430 | 98.360 |
| Total | | 100.000 |

HPLC Data for **31**: Chiralpak AS-H (99.9:0.1 hexane:IPA, flow rate 1.0 mLmin$^{-1}$, 254 nm, 30 °C), $t_R$ (minor): 4.7 min, $t_R$ (major): 5.3 min, >99:1 er.

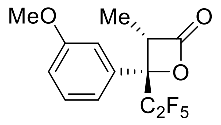

**31**

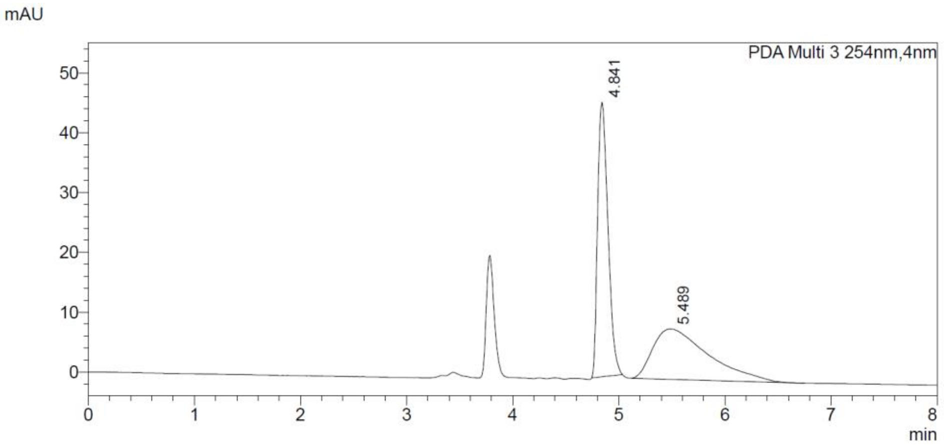


**<Peak Table>**

PDA Ch3 254nm

| Peak# | Ret. Time | Area% |
|---|---|---|
| 1 | 4.841 | 50.360 |
| 2 | 5.489 | 49.640 |
| Total | | 100.000 |

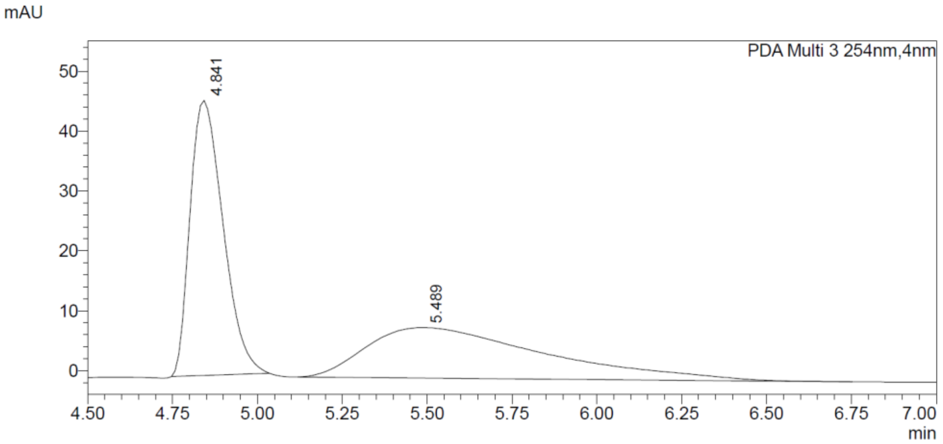


**<Peak Table>**

PDA Ch3 254nm

| Peak# | Ret. Time | Area% |
|---|---|---|
| 1 | 4.841 | 50.360 |
| 2 | 5.489 | 49.640 |
| Total | | 100.000 |

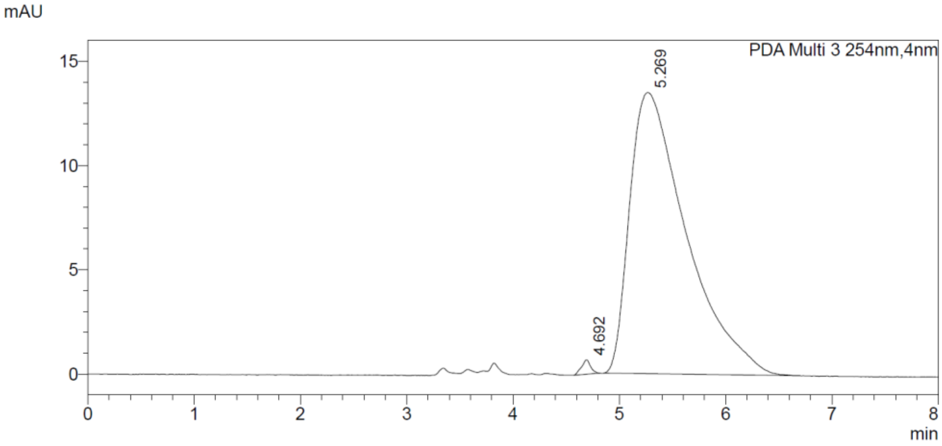


## <Peak Table>

PDA Ch3 254nm

| Peak# | Ret. Time | Area% |
|---|---|---|
| 1 | 4.692 | 0.824 |
| 2 | 5.269 | 99.176 |
| Total | | 100.000 |

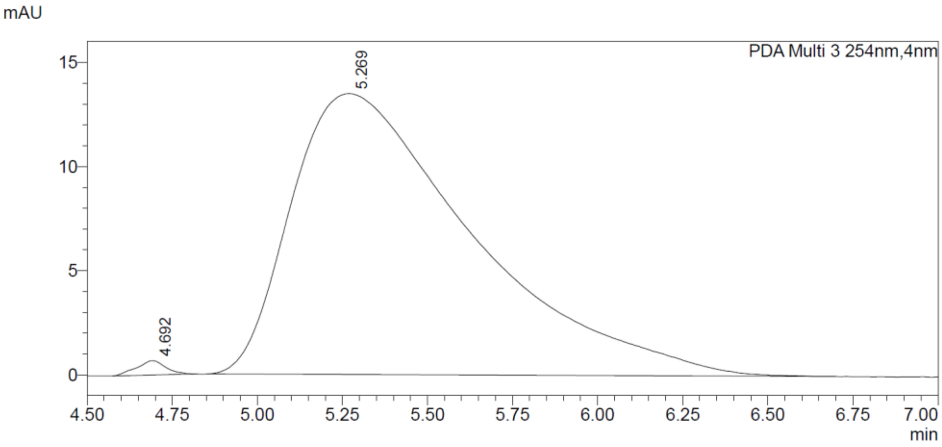


## <Peak Table>

PDA Ch3 254nm

| Peak# | Ret. Time | Area% |
|---|---|---|
| 1 | 4.692 | 0.824 |
| 2 | 5.269 | 99.176 |
| Total | | 100.000 |

HPLC Data for **32**: Chiralpak AS-H (99.9:0.1 hexane:IPA, flow rate 1.0 mLmin$^{-1}$, 211 nm, 30 °C), $t_R$ (minor): 4.0 min, $t_R$ (major): 4.2 min, >99:1 er.

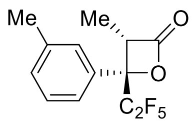


**32**

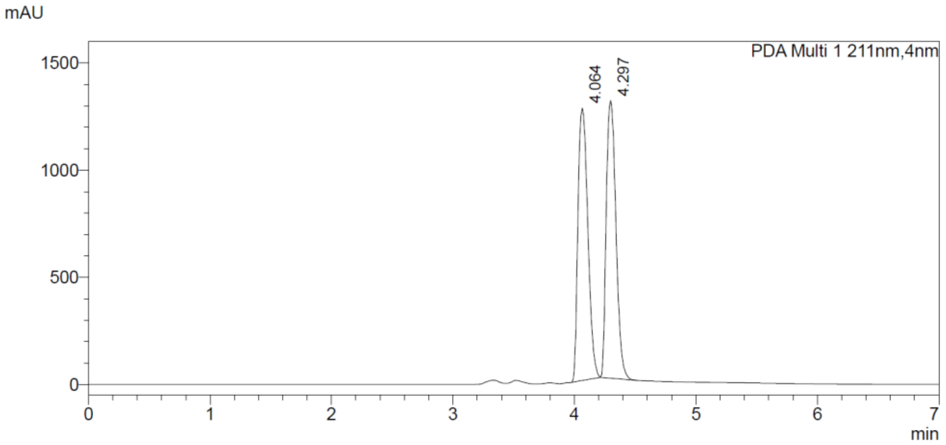


**<Peak Table>**

PDA Ch1 211nm

| Peak# | Ret. Time | Area% |
|---|---|---|
| 1 | 4.064 | 50.057 |
| 2 | 4.297 | 49.943 |
| Total | | 100.000 |

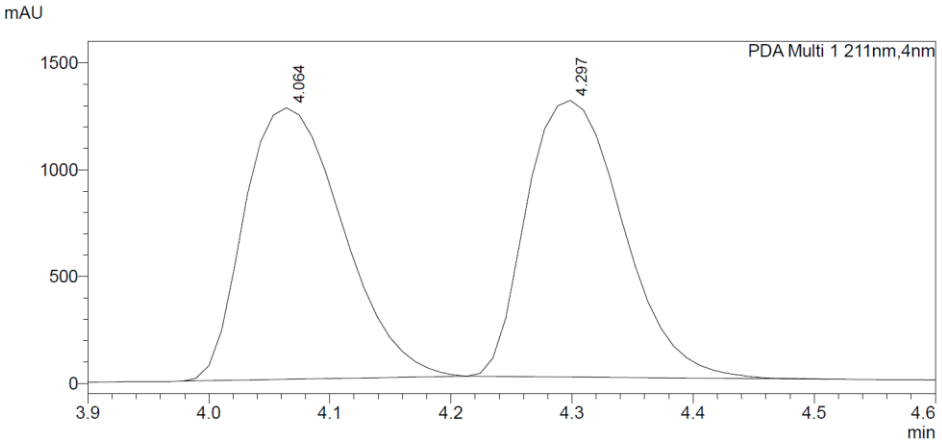


**<Peak Table>**

PDA Ch1 211nm

| Peak# | Ret. Time | Area% |
|---|---|---|
| 1 | 4.064 | 50.057 |
| 2 | 4.297 | 49.943 |
| Total | | 100.000 |

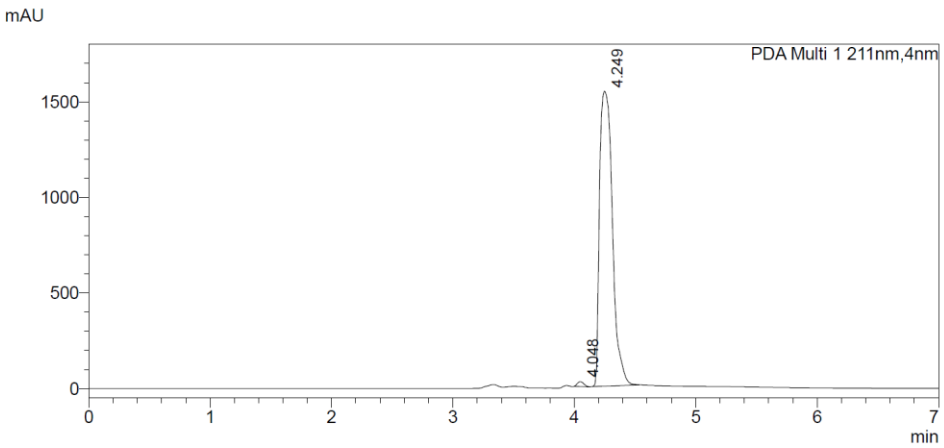


**<Peak Table>**

PDA Ch1 211nm

| Peak# | Ret. Time | Area% |
|---|---|---|
| 1 | 4.048 | 0.841 |
| 2 | 4.249 | 99.159 |
| Total | | 100.000 |

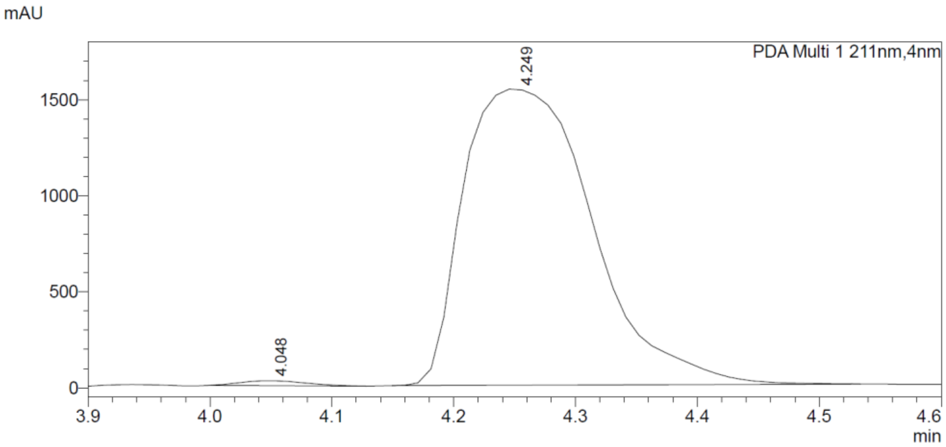


**<Peak Table>**

PDA Ch1 211nm

| Peak# | Ret. Time | Area% |
|---|---|---|
| 1 | 4.048 | 0.841 |
| 2 | 4.249 | 99.159 |
| Total | | 100.000 |

HPLC Data for **33**: Chiralcel OJ-H (99.8:0.2 hexane:IPA, flow rate 1.0 mLmin$^{-1}$, 211 nm, 30 °C), $t_R$ (major): 4.5 min, $t_R$ (minor): 5.0 min, 98:2 er.

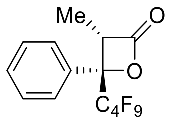

**33**

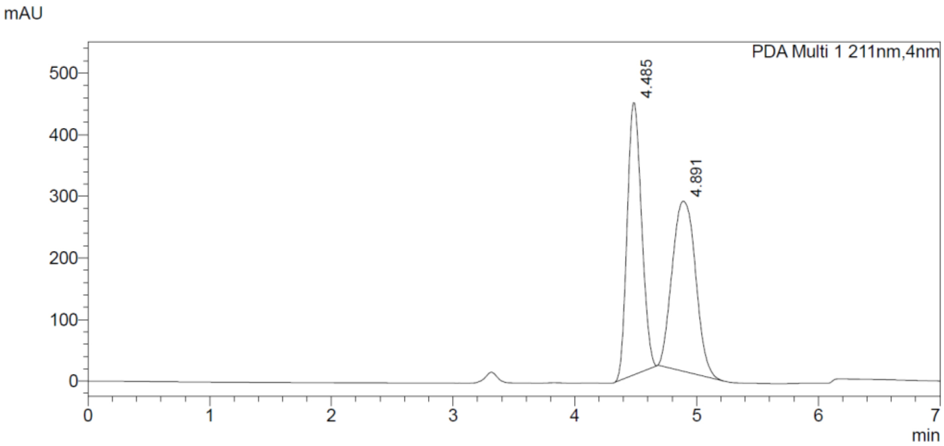


**<Peak Table>**

PDA Ch1 211nm

| Peak# | Ret. Time | Area% |
|---|---|---|
| 1 | 4.485 | 49.516 |
| 2 | 4.891 | 50.484 |
| Total | | 100.000 |

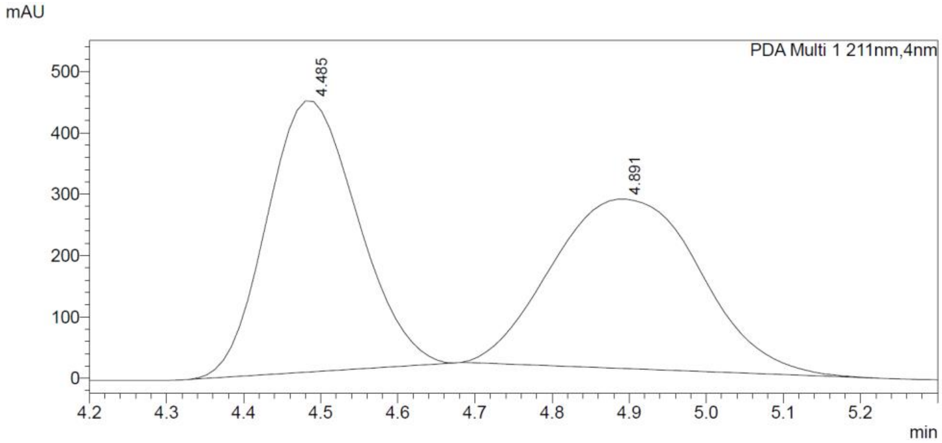


**<Peak Table>**

PDA Ch1 211nm

| Peak# | Ret. Time | Area% |
|---|---|---|
| 1 | 4.485 | 49.516 |
| 2 | 4.891 | 50.484 |
| Total | | 100.000 |

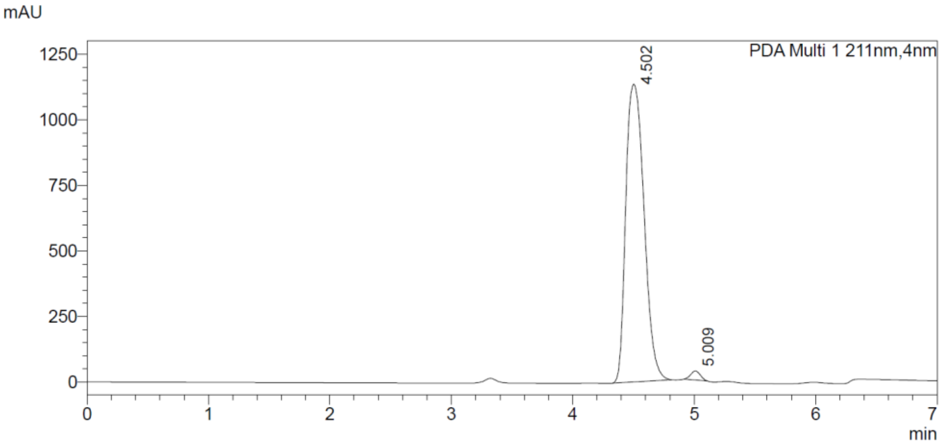


## <Peak Table>

PDA Ch1 211nm

| Peak# | Ret. Time | Area% |
|---|---|---|
| 1 | 4.502 | 98.440 |
| 2 | 5.009 | 1.560 |
| Total | | 100.000 |

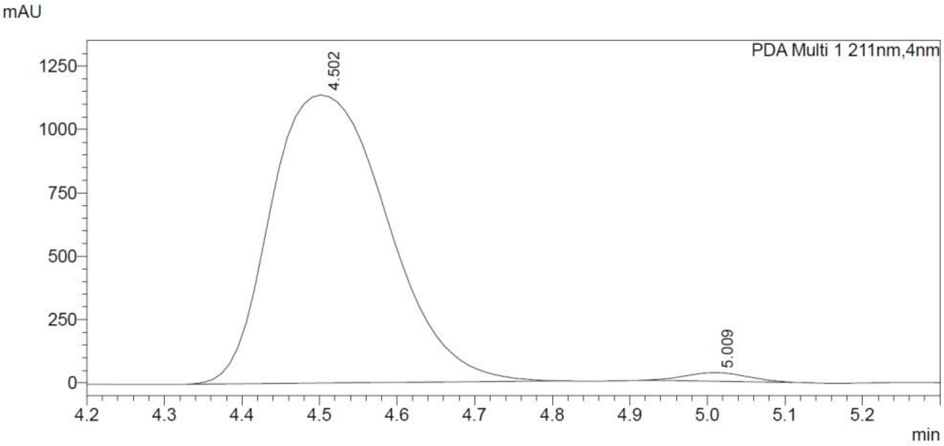




## <Peak Table>

PDA Ch1 211nm

| Peak# | Ret. Time | Area% |
|---|---|---|
| 1 | 4.502 | 98.440 |
| 2 | 5.009 | 1.560 |
| Total | | 100.000 |

HPLC Data for **34**: Chiralpak IB (99.9:0.1 hexane:IPA, flow rate 1.0 mLmin$^{-1}$, 254 nm, 30 °C), $t_R$ (major): 5.4 min, >99:1 er.

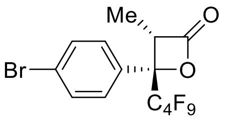


**34**

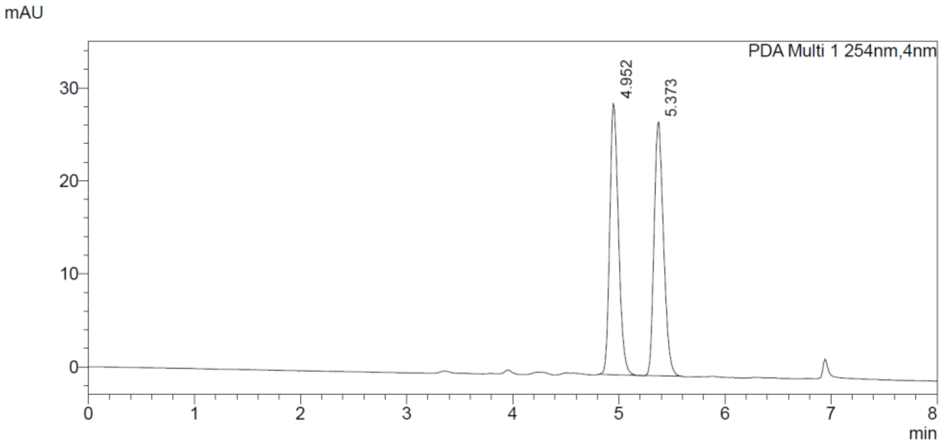


**<Peak Table>**

PDA Ch1 254nm

| Peak# | Ret. Time | Area% |
|---|---|---|
| 1 | 4.952 | 49.749 |
| 2 | 5.373 | 50.251 |
| Total | | 100.000 |

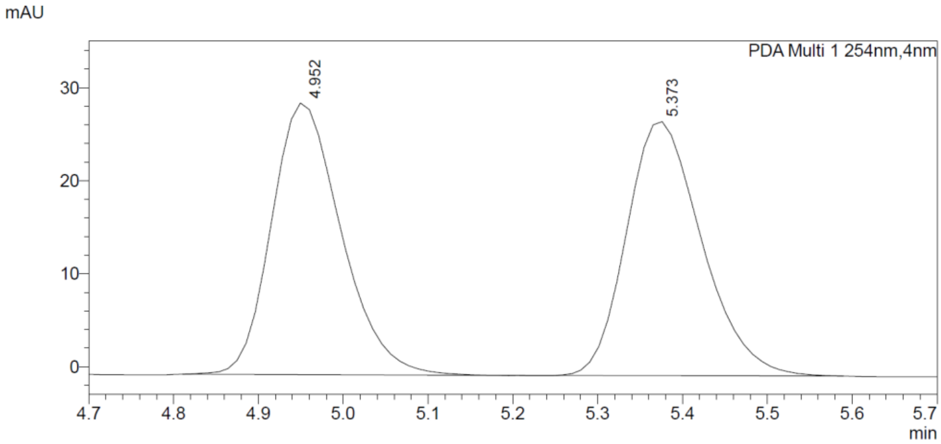



**<Peak Table>**

PDA Ch1 254nm

| Peak# | Ret. Time | Area% |
|---|---|---|
| 1 | 4.952 | 49.749 |
| 2 | 5.373 | 50.251 |
| Total | | 100.000 |

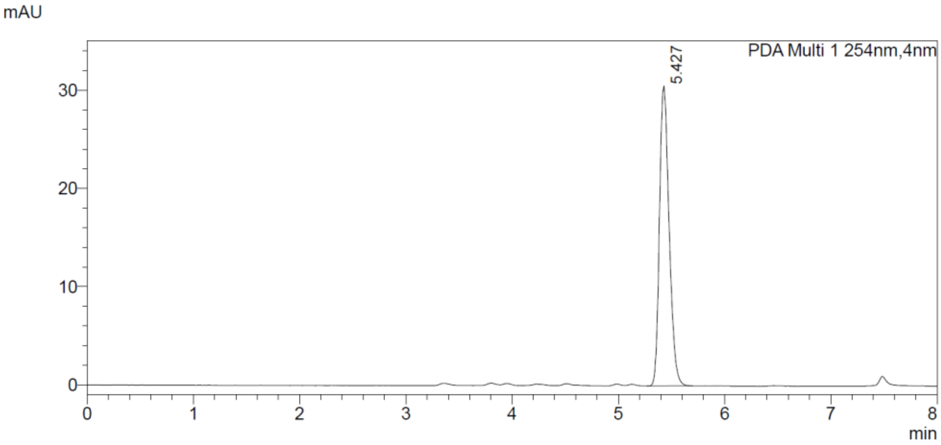


## <Peak Table>

PDA Ch1 254nm

| Peak# | Ret. Time | Area% |
|---|---|---|
| 1 | 5.427 | 100.000 |
| Total | | 100.000 |

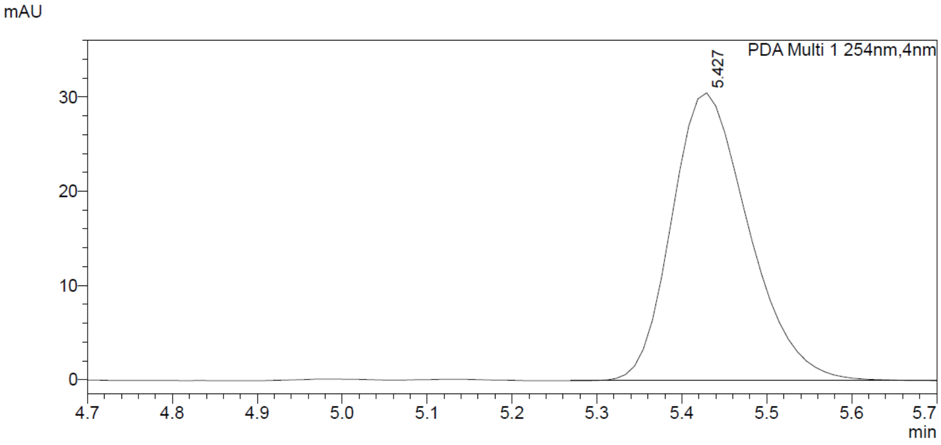


## <Peak Table>

PDA Ch1 254nm

| Peak# | Ret. Time | Area% |
|---|---|---|
| 1 | 5.427 | 100.000 |
| Total | | 100.000 |

HPLC Data for **35**: Chiralpak IB (99.9:0.1 hexane:IPA, flow rate 1.0 mLmin$^{-1}$, 211 nm, 30 °C), major: $t_R$ (minor): 15.1min, $t_R$ (major): 19.3min, >99:1 er.

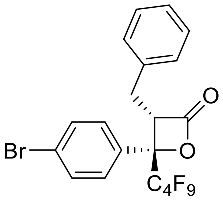


**35**

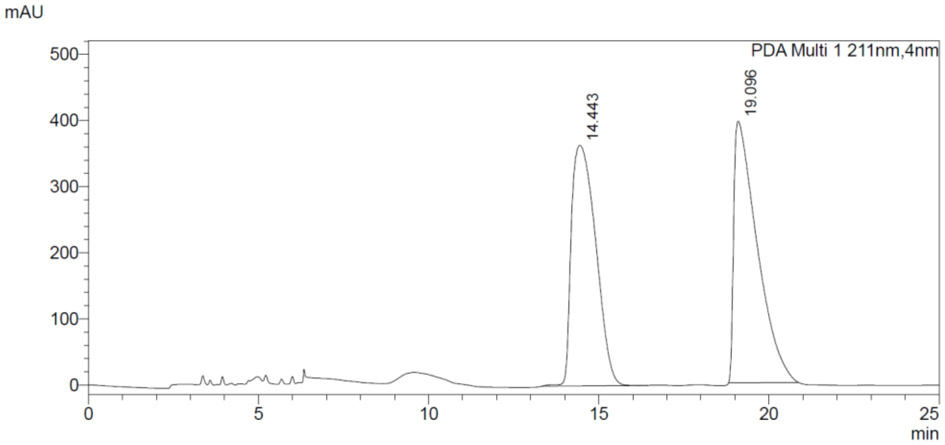


**<Peak Table>**

PDA Ch1 211nm

| Peak# | Ret. Time | Area% |
|---|---|---|
| 1 | 14.443 | 49.013 |
| 2 | 19.096 | 50.987 |
| Total | | 100.000 |

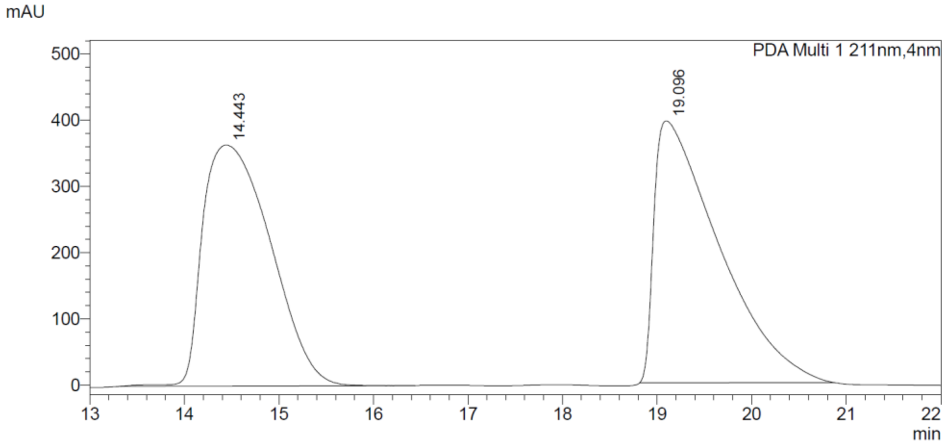


**<Peak Table>**

PDA Ch1 211nm

| Peak# | Ret. Time | Area% |
|---|---|---|
| 1 | 14.443 | 49.013 |
| 2 | 19.096 | 50.987 |
| Total | | 100.000 |

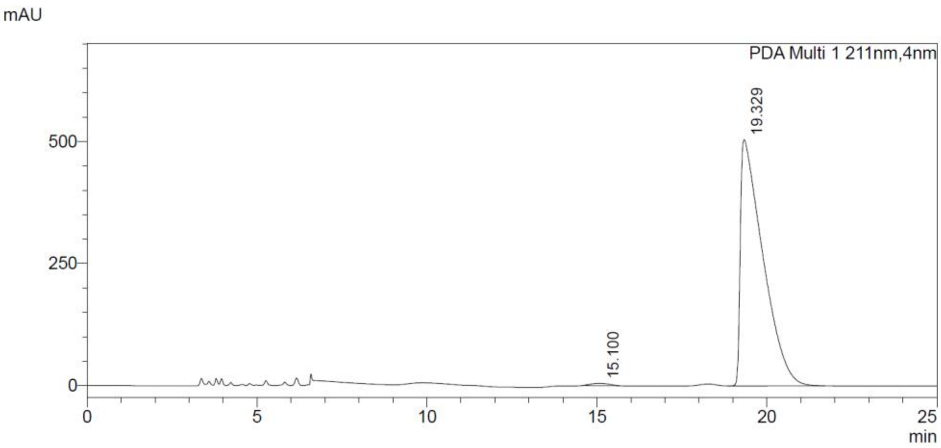


**<Peak Table>**

PDA Ch1 211nm

| Peak# | Ret. Time | Area% |
|---|---|---|
| 1 | 15.100 | 0.760 |
| 2 | 19.329 | 99.240 |
| Total | | 100.000 |

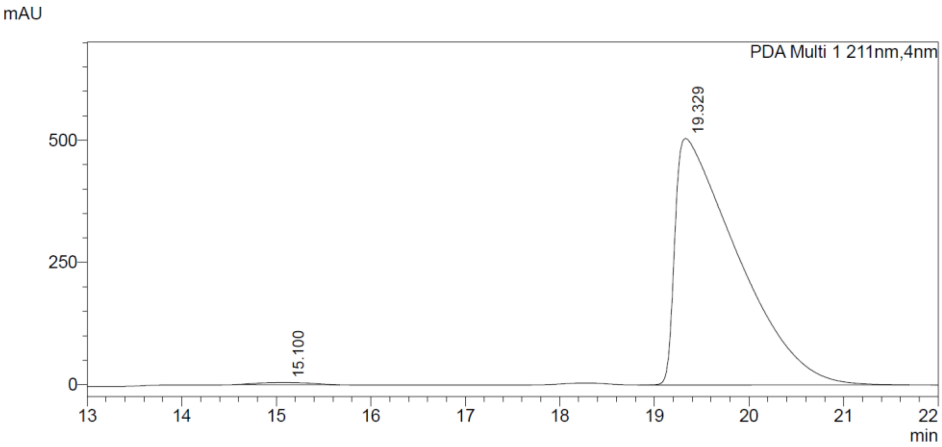


**<Peak Table>**

PDA Ch1 211nm

| Peak# | Ret. Time | Area% |
|---|---|---|
| 1 | 15.100 | 0.760 |
| 2 | 19.329 | 99.240 |
| Total | | 100.000 |

HPLC Data for **36**: Chiralpak IB (99:1 hexane:IPA, flow rate 1.0 mLmin$^{-1}$, 211 nm, 30 °C), **major diastereoisomer**: $t_R$ (minor): 18.6 min, $t_R$ (major): 27.9 min, 99:1 er.

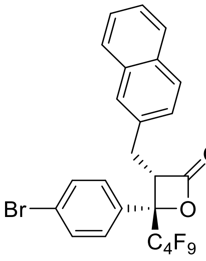


**36**

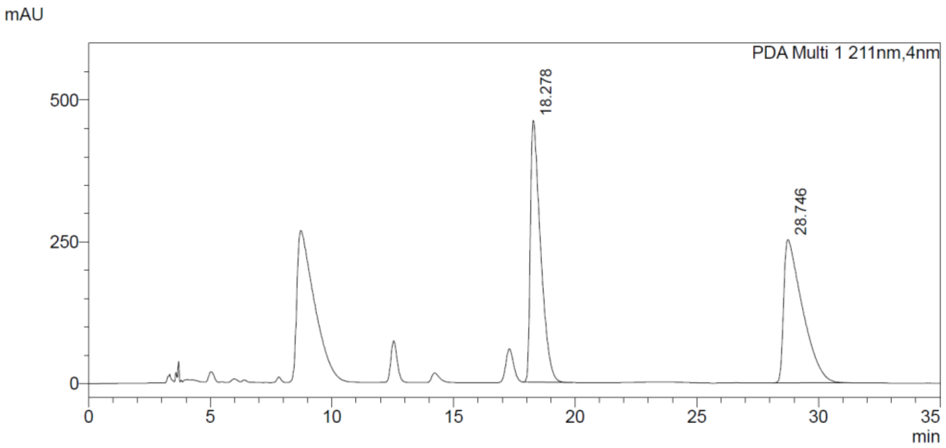


**<Peak Table>**

PDA Ch1 211nm

| Peak# | Ret. Time | Area% |
|---|---|---|
| 1 | 18.278 | 49.820 |
| 2 | 28.746 | 50.180 |
| Total | | 100.000 |

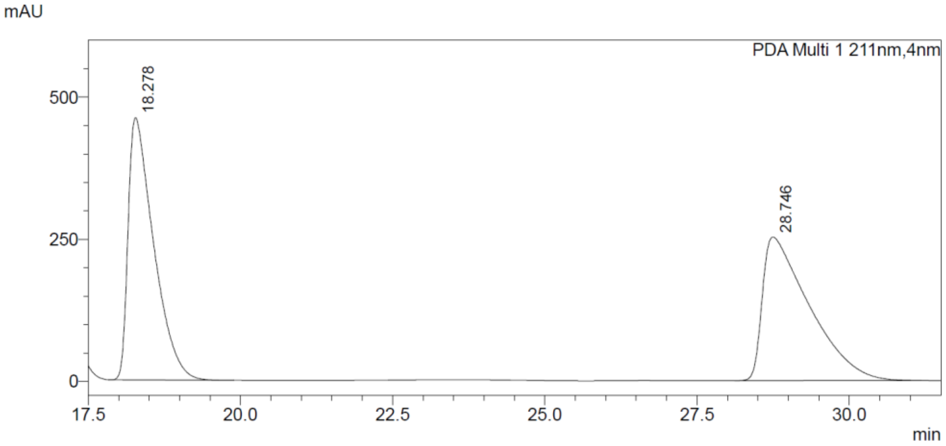


**<Peak Table>**

PDA Ch1 211nm

| Peak# | Ret. Time | Area% |
|---|---|---|
| 1 | 18.278 | 49.820 |
| 2 | 28.746 | 50.180 |
| Total | | 100.000 |

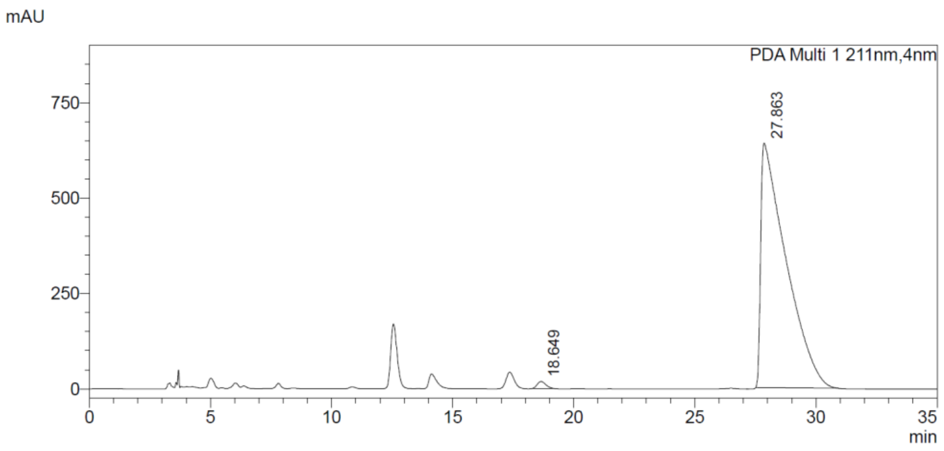


**<Peak Table>**

PDA Ch1 211nm

| Peak# | Ret. Time | Area% |
|---|---|---|
| 1 | 18.649 | 1.008 |
| 2 | 27.863 | 98.992 |
| Total | | 100.000 |

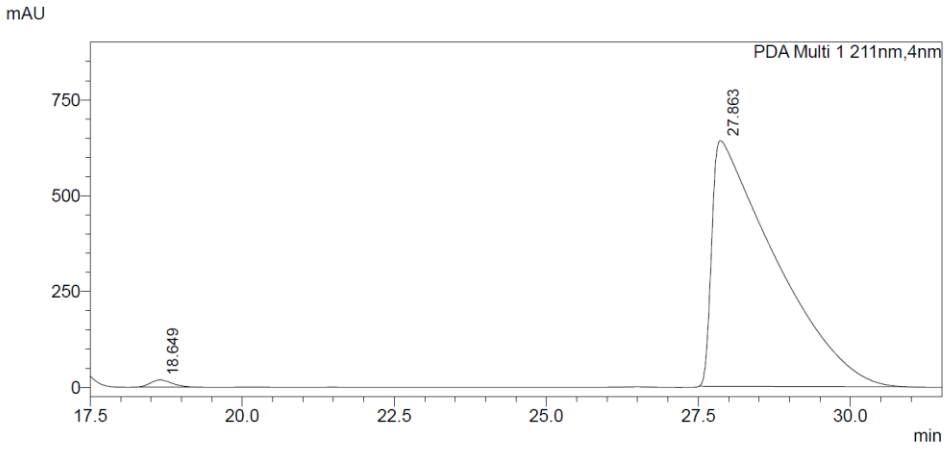


**<Peak Table>**

PDA Ch1 211nm

| Peak# | Ret. Time | Area% |
|---|---|---|
| 1 | 18.649 | 1.008 |
| 2 | 27.863 | 98.992 |
| Total | | 100.000 |

**Minor diastereoisomer**: $t_R$ (major): 12.6 min, $t_R$ (minor): 17.4 min, 76:24 er.

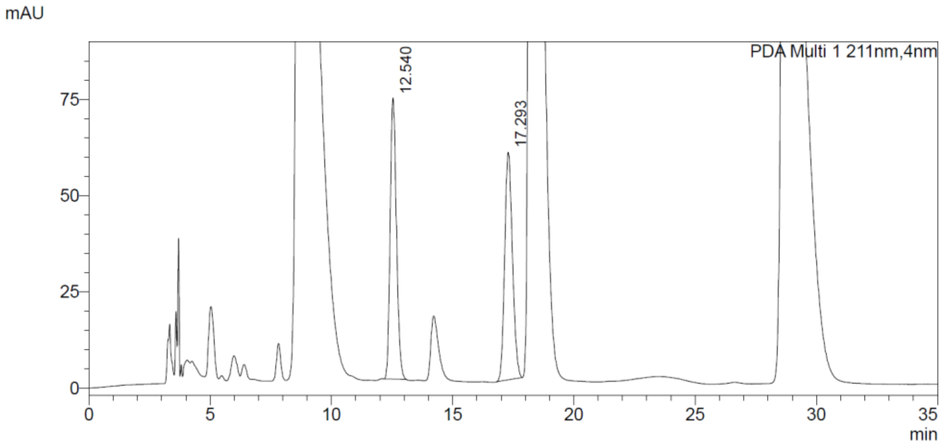


**<Peak Table>**

PDA Ch1 211nm

| Peak# | Ret. Time | Area% |
|---|---|---|
| 1 | 12.540 | 49.877 |
| 2 | 17.293 | 50.123 |
| Total | | 100.000 |

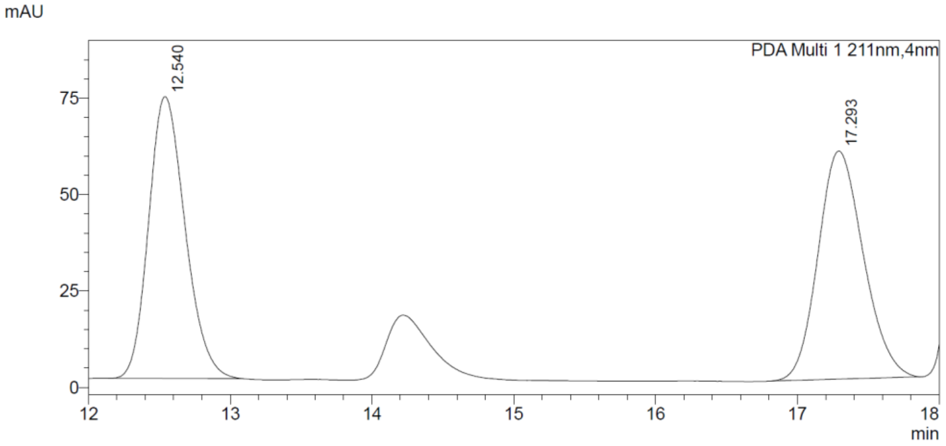


**<Peak Table>**

PDA Ch1 211nm

| Peak# | Ret. Time | Area% |
|---|---|---|
| 1 | 12.540 | 49.877 |
| 2 | 17.293 | 50.123 |
| Total | | 100.000 |

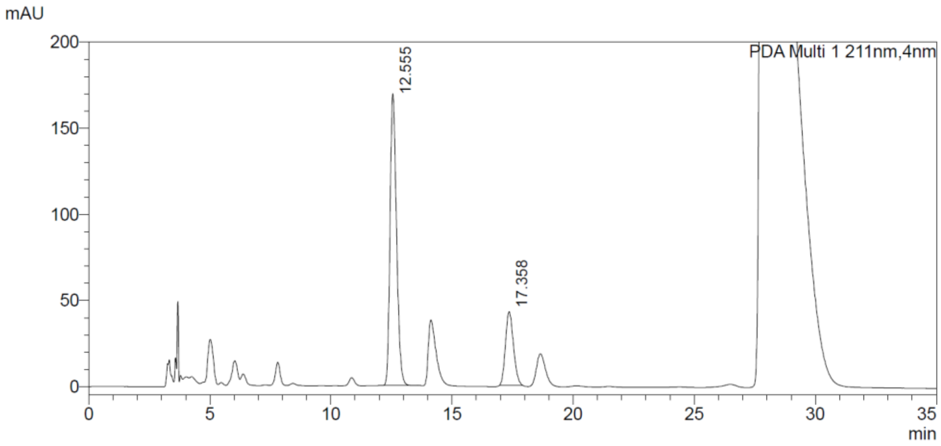


**<Peak Table>**

PDA Ch1 211nm

| Peak# | Ret. Time | Area% |
|---|---|---|
| 1 | 12.555 | 76.258 |
| 2 | 17.358 | 23.742 |
| Total | | 100.000 |

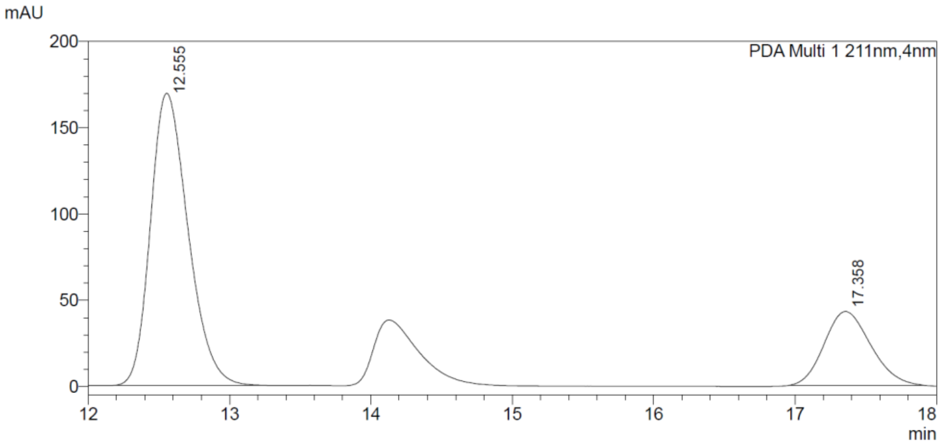


**<Peak Table>**

PDA Ch1 211nm

| Peak# | Ret. Time | Area% |
|---|---|---|
| 1 | 12.555 | 76.258 |
| 2 | 17.358 | 23.742 |
| Total | | 100.000 |

HPLC Data for **42**: Chiralpak IB (99.3:0.7 hexane:IPA, flow rate 1.0 mL min$^{-1}$, 254 nm, 30 °C), $t_R$ (major): 16.1 min, $t_R$ (minor): 24.9 min, 92:8 er.

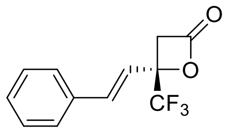

**42**

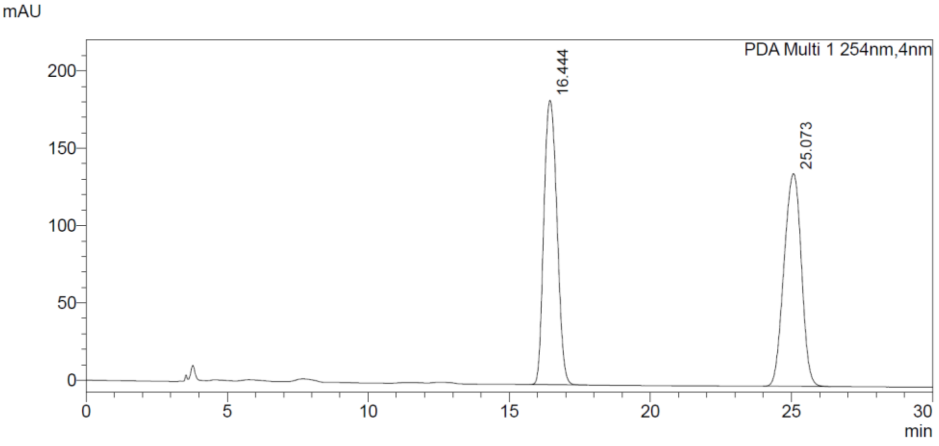


**<Peak Table>**

PDA Ch1 254nm

| Peak# | Ret. Time | Area% |
|---|---|---|
| 1 | 16.444 | 50.222 |
| 2 | 25.073 | 49.778 |
| Total | | 100.000 |

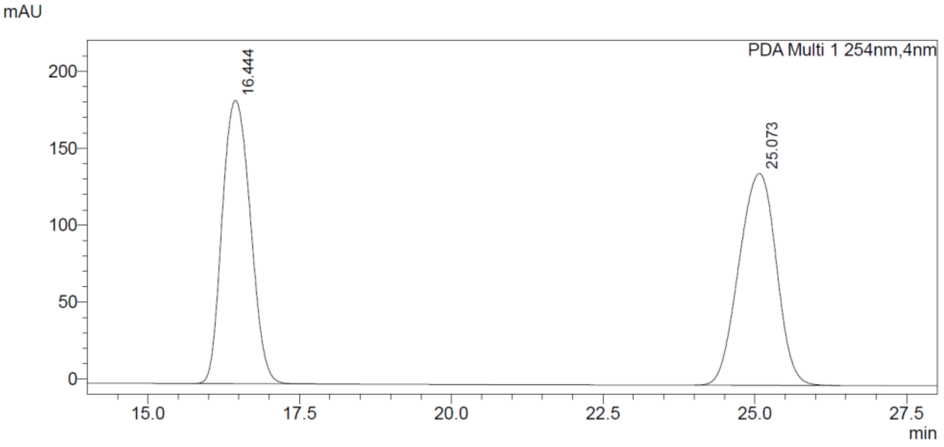



**<Peak Table>**

PDA Ch1 254nm

| Peak# | Ret. Time | Area% |
|---|---|---|
| 1 | 16.444 | 50.222 |
| 2 | 25.073 | 49.778 |
| Total | | 100.000 |

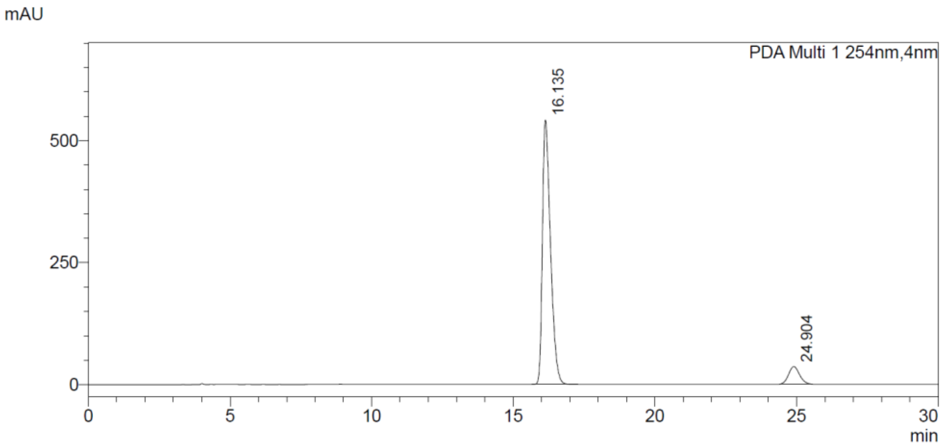


**<Peak Table>**

PDA Ch1 254nm

| Peak# | Ret. Time | Area% |
|---|---|---|
| 1 | 16.135 | 91.869 |
| 2 | 24.904 | 8.131 |
| Total | | 100.000 |

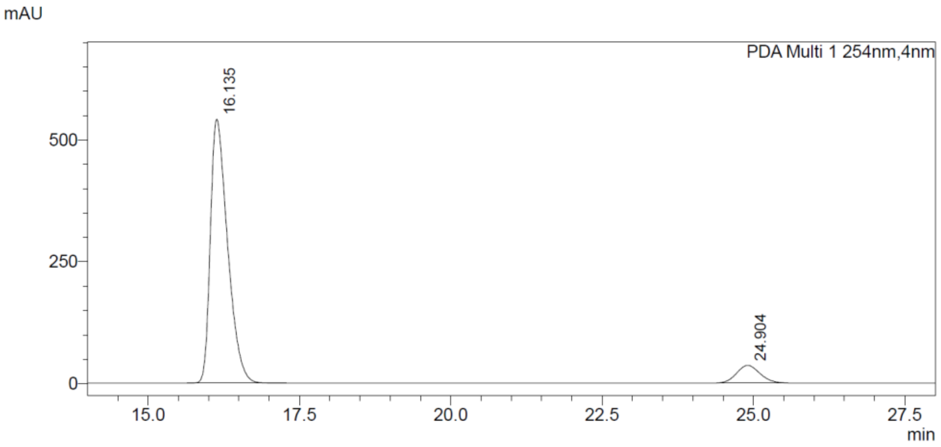


**<Peak Table>**

PDA Ch1 254nm

| Peak# | Ret. Time | Area% |
|---|---|---|
| 1 | 16.135 | 91.869 |
| 2 | 24.904 | 8.131 |
| Total | | 100.000 |

HPLC Data for **43**: **major diastereoisomer**: Chiralpak IB (99.5:0.5 hexane:IPA, flow rate 0.7 $mLmin^{-1}$, 254 nm, 30 °C), $t_R$ (minor): 7.3 min, $t_R$ (major): 9.2 min, 99:1 er.

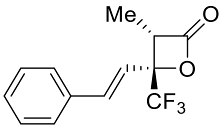


**43**

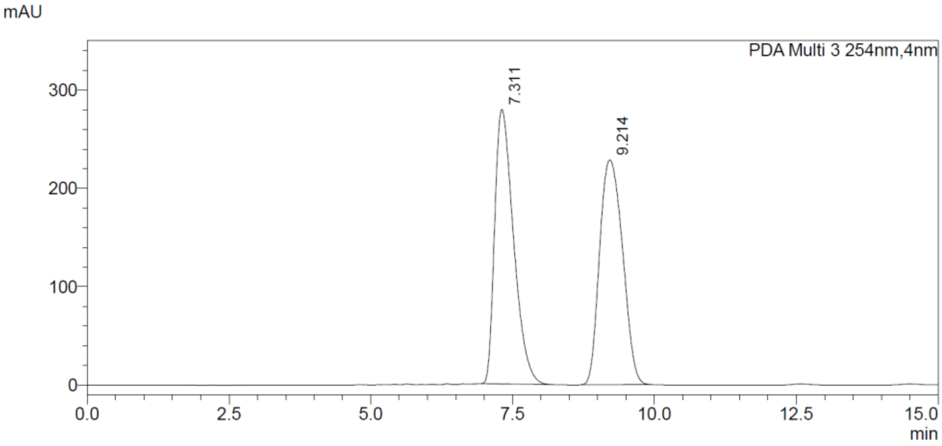


## <Peak Table>

PDA Ch3 254nm

| Peak# | Ret. Time | Area% |
|---|---|---|
| 1 | 7.311 | 49.988 |
| 2 | 9.214 | 50.012 |
| Total | | 100.000 |

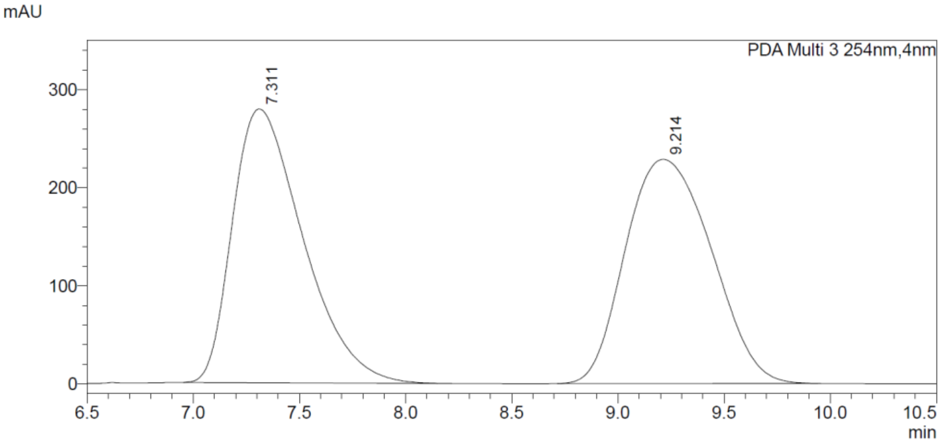


## <Peak Table>

PDA Ch3 254nm

| Peak# | Ret. Time | Area% |
|---|---|---|
| 1 | 7.311 | 49.988 |
| 2 | 9.214 | 50.012 |
| Total | | 100.000 |

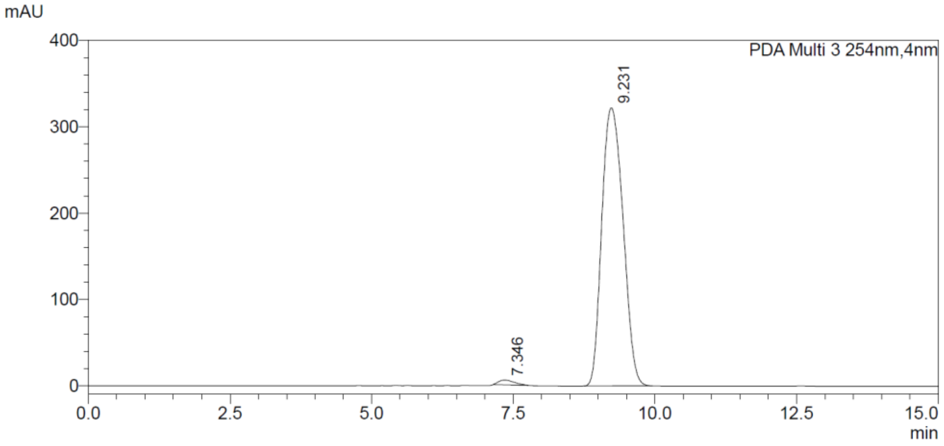


**<Peak Table>**

PDA Ch3 254nm

| Peak# | Ret. Time | Area% |
|---|---|---|
| 1 | 7.346 | 1.219 |
| 2 | 9.231 | 98.781 |
| Total | | 100.000 |

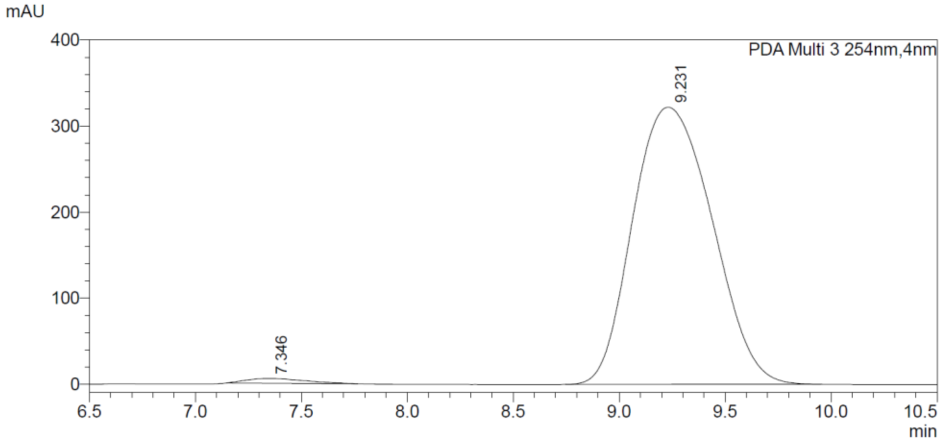


**<Peak Table>**

PDA Ch3 254nm

| Peak# | Ret. Time | Area% |
|---|---|---|
| 1 | 7.346 | 1.219 |
| 2 | 9.231 | 98.781 |
| Total | | 100.000 |

**Minor diastereoisomer**: Chiralpak IB (99.5:0.5 hexane:IPA, flow rate 0.7 mLmin$^{-1}$, 211 nm, 30 °C), $t_R$ (major): 12.1 min, $t_R$ (minor): 13.9 min, 77:23 er.

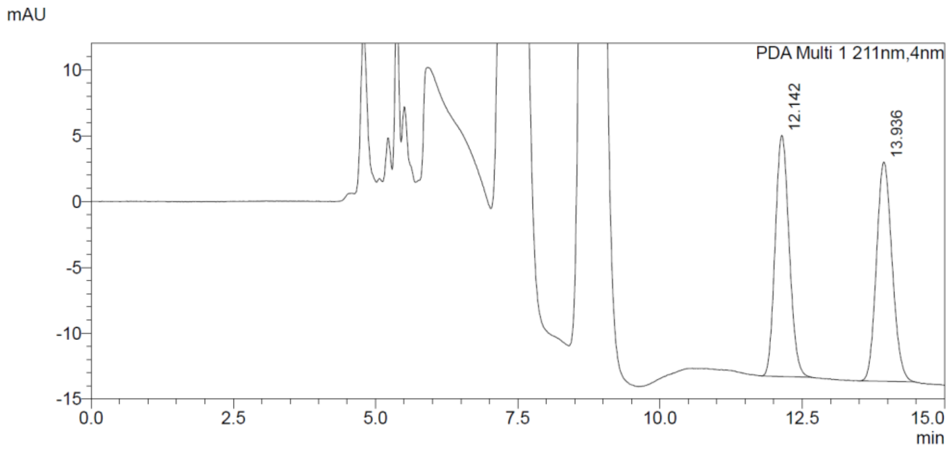


**<Peak Table>**

PDA Ch1 211nm

| Peak# | Ret. Time | Area% |
|---|---|---|
| 1 | 12.142 | 49.603 |
| 2 | 13.936 | 50.397 |
| Total | | 100.000 |

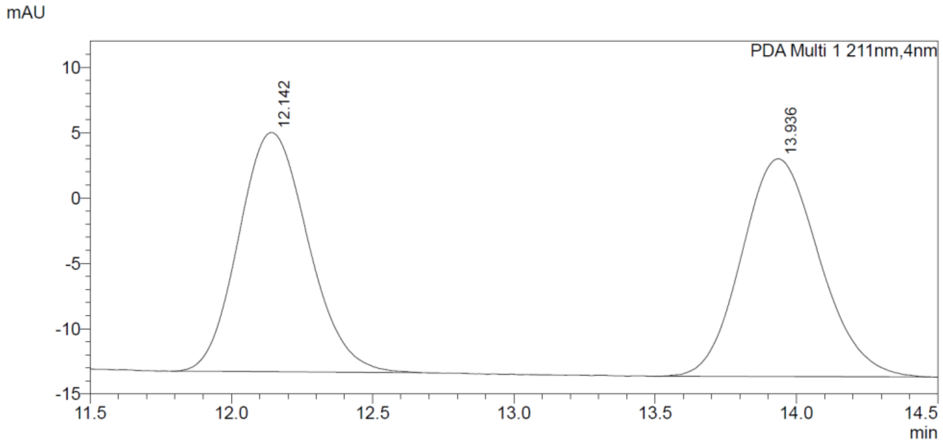


**<Peak Table>**

PDA Ch1 211nm

| Peak# | Ret. Time | Area% |
|---|---|---|
| 1 | 12.142 | 49.603 |
| 2 | 13.936 | 50.397 |
| Total | | 100.000 |

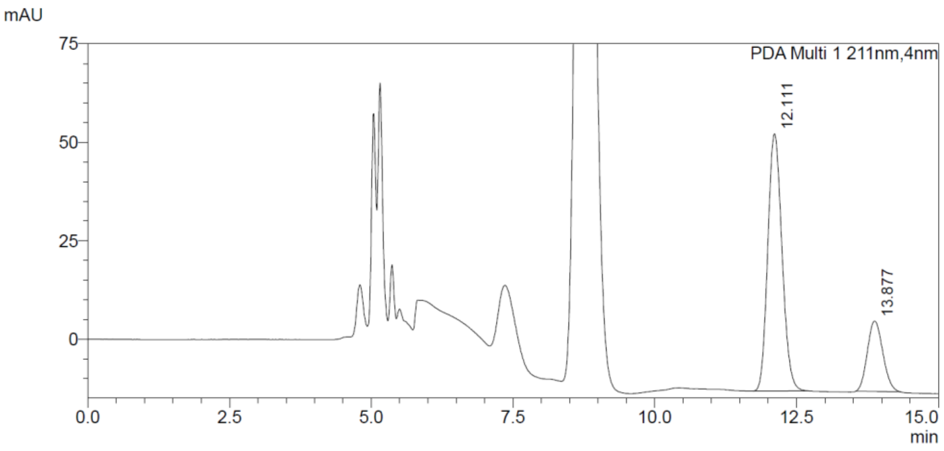


**<Peak Table>**

PDA Ch1 211nm

| Peak# | Ret. Time | Area% |
|---|---|---|
| 1 | 12.111 | 77.036 |
| 2 | 13.877 | 22.964 |
| Total | | 100.000 |

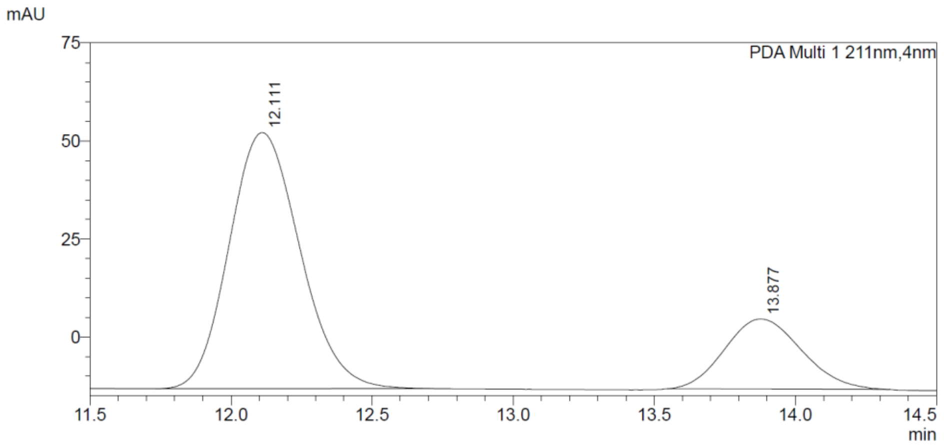


**<Peak Table>**

PDA Ch1 211nm

| Peak# | Ret. Time | Area% |
|---|---|---|
| 1 | 12.111 | 77.036 |
| 2 | 13.877 | 22.964 |
| Total | | 100.000 |

HPLC Data for **44**: Chiralcel OJ-H (99.8:0.2 hexane:IPA, flow rate 1.0 mLmin$^{-1}$, 254 nm, 30 °C), $t_R$ (minor): 13.1 min, $t_R$ (major): 17.4 min, >99:1 er.

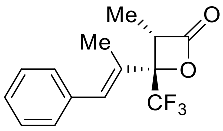


**44**

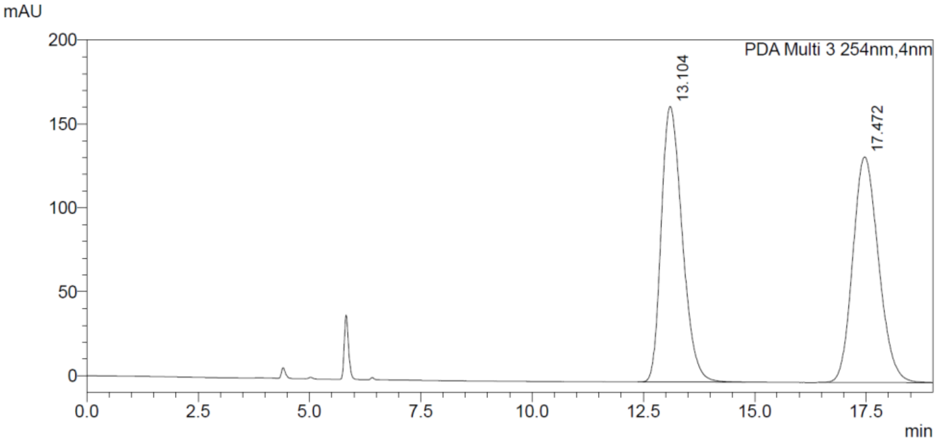


**<Peak Table>**

PDA Ch3 254nm

| Peak# | Ret. Time | Area% |
|---|---|---|
| 1 | 13.104 | 49.862 |
| 2 | 17.472 | 50.138 |
| Total | | 100.000 |

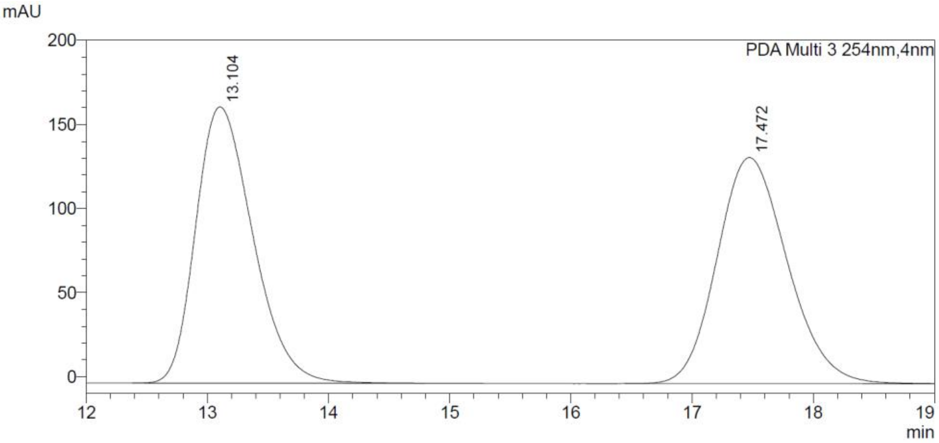


**<Peak Table>**

PDA Ch3 254nm

| Peak# | Ret. Time | Area% |
|---|---|---|
| 1 | 13.104 | 49.862 |
| 2 | 17.472 | 50.138 |
| Total | | 100.000 |

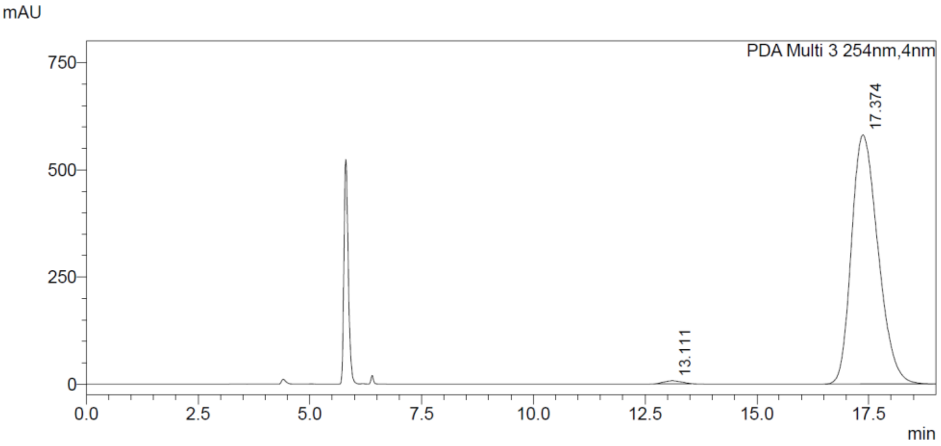


## <Peak Table>

PDA Ch3 254nm

| Peak# | Ret. Time | Area% |
|---|---|---|
| 1 | 13.111 | 0.688 |
| 2 | 17.374 | 99.312 |
| Total | | 100.000 |

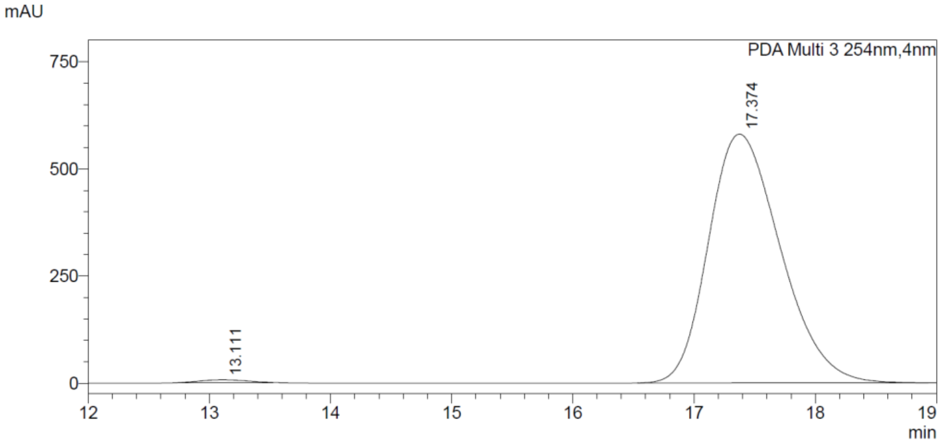


## <Peak Table>

PDA Ch3 254nm

| Peak# | Ret. Time | Area% |
|---|---|---|
| 1 | 13.111 | 0.688 |
| 2 | 17.374 | 99.312 |
| Total | | 100.000 |

HPLC Data for **45**: Chiralpak AD-H (95:5 hexane:IPA, flow rate 1.0 mLmin$^{-1}$, 211 nm, 30 °C), **major diastereoisomer**: $t_R$ (major): 8.8 min, $t_R$ (minor): 9.6 min, 95:5 er.

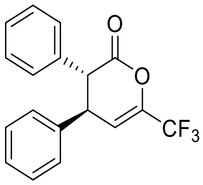


**45**

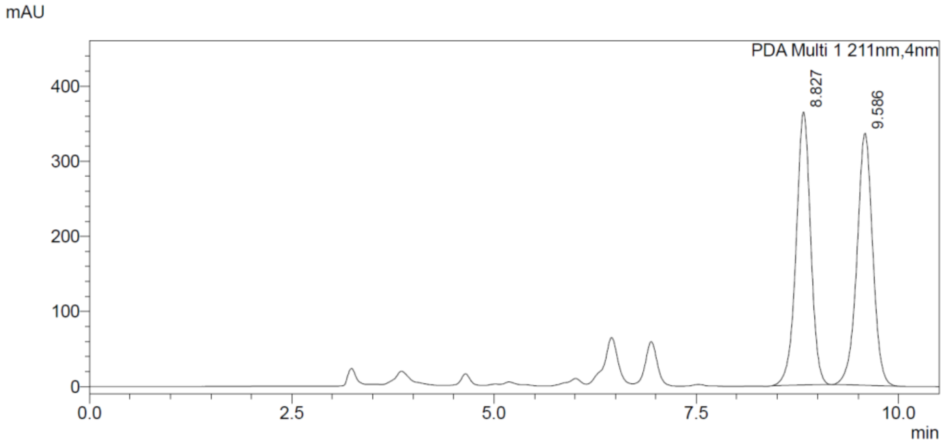


**<Peak Table>**

PDA Ch1 211nm

| Peak# | Ret. Time | Area% |
|---|---|---|
| 1 | 8.827 | 49.927 |
| 2 | 9.586 | 50.073 |
| Total | | 100.000 |

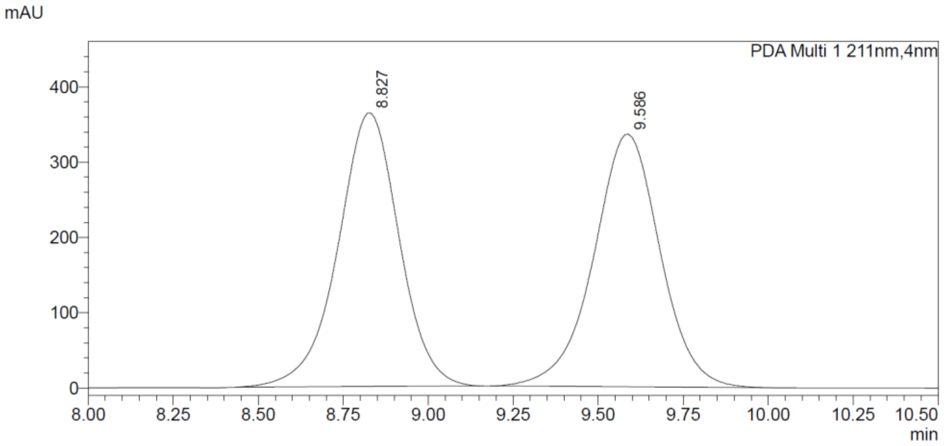


**<Peak Table>**

PDA Ch1 211nm

| Peak# | Ret. Time | Area% |
|---|---|---|
| 1 | 8.827 | 49.927 |
| 2 | 9.586 | 50.073 |
| Total | | 100.000 |

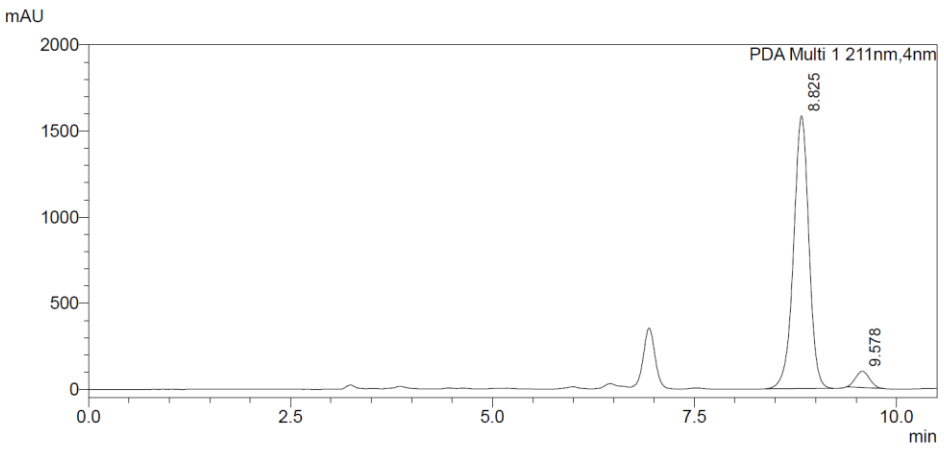


## <Peak Table>

PDA Ch1 211nm

| Peak# | Ret. Time | Area% |
|---|---|---|
| 1 | 8.825 | 94.884 |
| 2 | 9.578 | 5.116 |
| Total | | 100.000 |

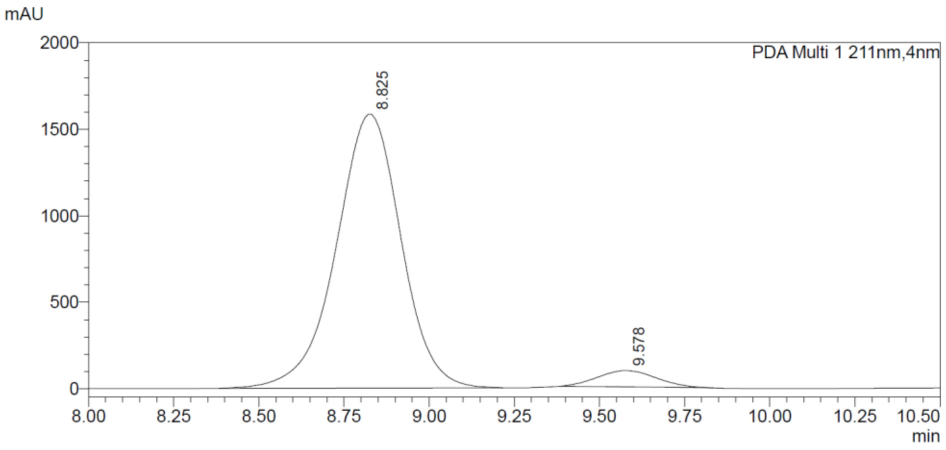


## <Peak Table>

PDA Ch1 211nm

| Peak# | Ret. Time | Area% |
|---|---|---|
| 1 | 8.825 | 94.884 |
| 2 | 9.578 | 5.116 |
| Total | | 100.000 |

**Minor diastereoisomer**: $t_R$ (minor): 6.5 min, $t_R$ (major): 6.9 min, 93:7 er.

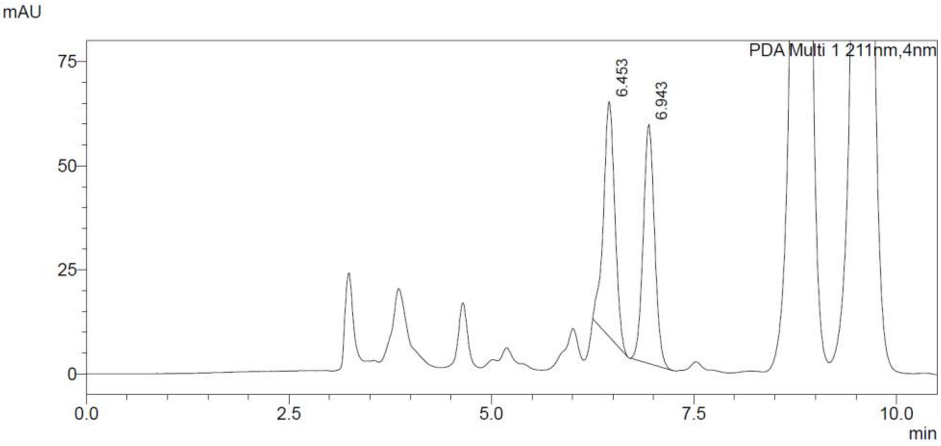


**<Peak Table>**

PDA Ch1 211nm

| Peak# | Ret. Time | Area% |
|---|---|---|
| 1 | 6.453 | 49.568 |
| 2 | 6.943 | 50.432 |
| Total | | 100.000 |

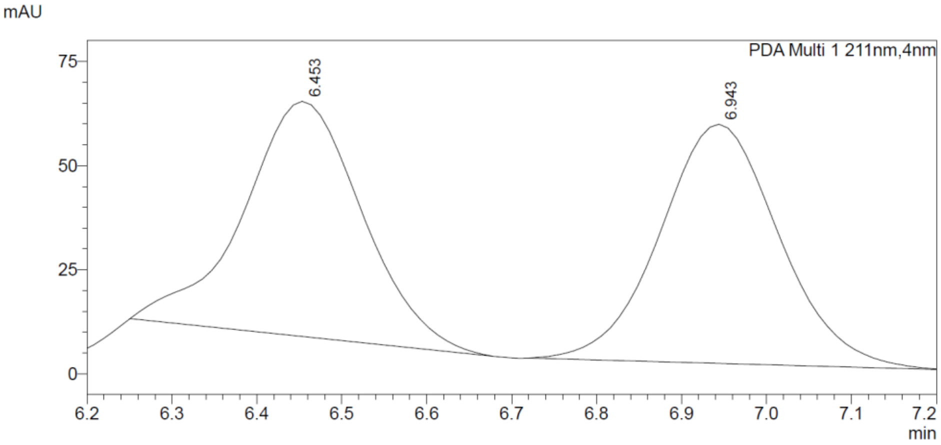


**<Peak Table>**

PDA Ch1 211nm

| Peak# | Ret. Time | Area% |
|---|---|---|
| 1 | 6.453 | 49.568 |
| 2 | 6.943 | 50.432 |
| Total | | 100.000 |

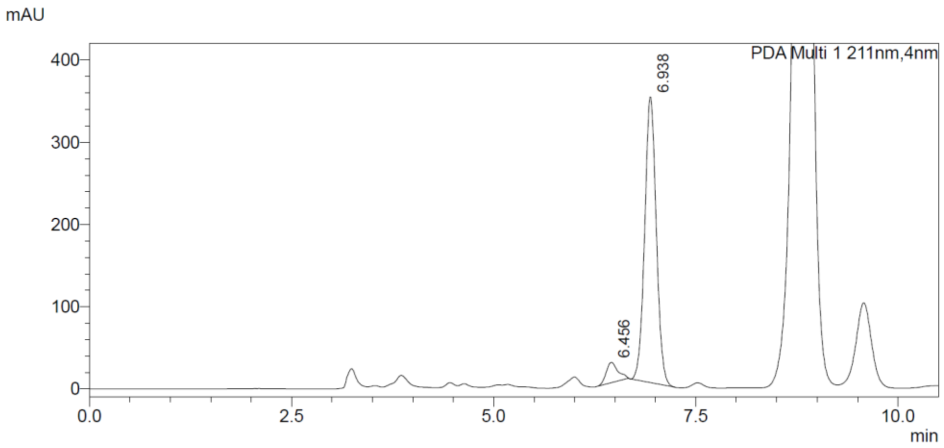


**<Peak Table>**

PDA Ch1 211nm

| Peak# | Ret. Time | Area% |
|---|---|---|
| 1 | 6.456 | 6.751 |
| 2 | 6.938 | 93.249 |
| Total | | 100.000 |

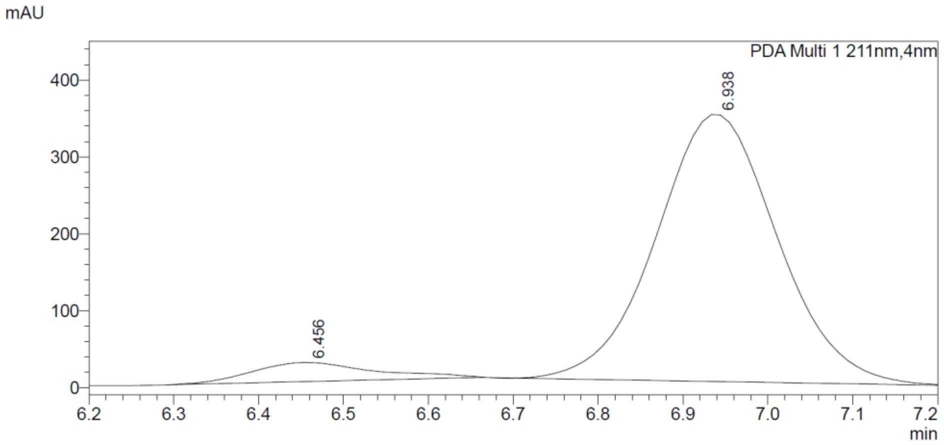


**<Peak Table>**

PDA Ch1 211nm

| Peak# | Ret. Time | Area% |
|---|---|---|
| 1 | 6.456 | 6.751 |
| 2 | 6.938 | 93.249 |
| Total | | 100.000 |

HPLC Data for **37b**: Chiralpak IA (95:5 hexane:IPA, flow rate 1.0 mLmin$^{-1}$, 211 nm, 30 °C), $t_R$ (minor): 12.6 min, $t_R$ (major): 15.1 min, 93:7 er.

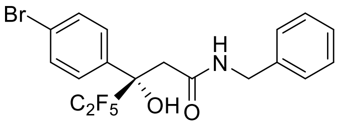


**37b**

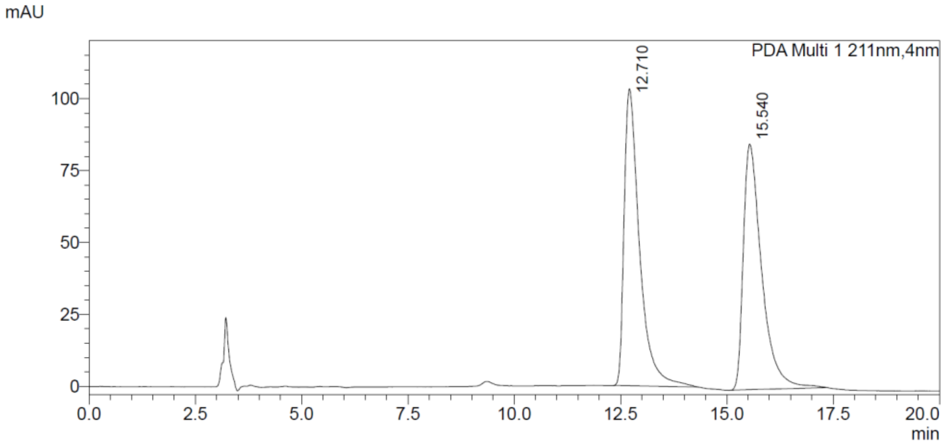


**<Peak Table>**

PDA Ch1 211nm

| Peak# | Ret. Time | Area% |
|---|---|---|
| 1 | 12.710 | 50.259 |
| 2 | 15.540 | 49.741 |
| Total | | 100.000 |

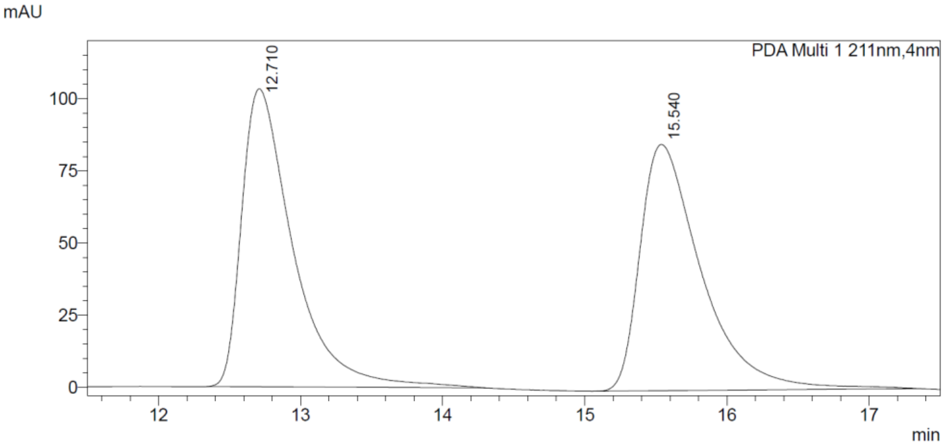


**<Peak Table>**

PDA Ch1 211nm

| Peak# | Ret. Time | Area% |
|---|---|---|
| 1 | 12.710 | 50.259 |
| 2 | 15.540 | 49.741 |
| Total | | 100.000 |

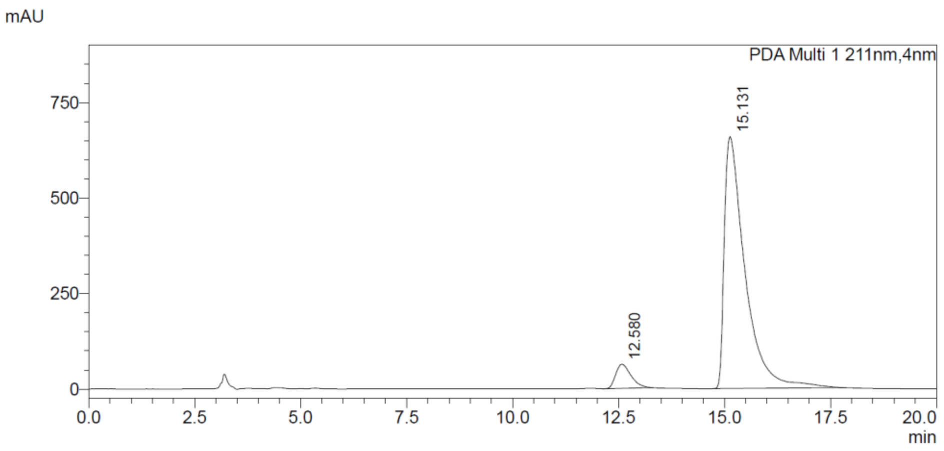


**<Peak Table>**

PDA Ch1 211nm

| Peak# | Ret. Time | Area% |
|---|---|---|
| 1 | 12.580 | 6.592 |
| 2 | 15.131 | 93.408 |
| Total | | 100.000 |

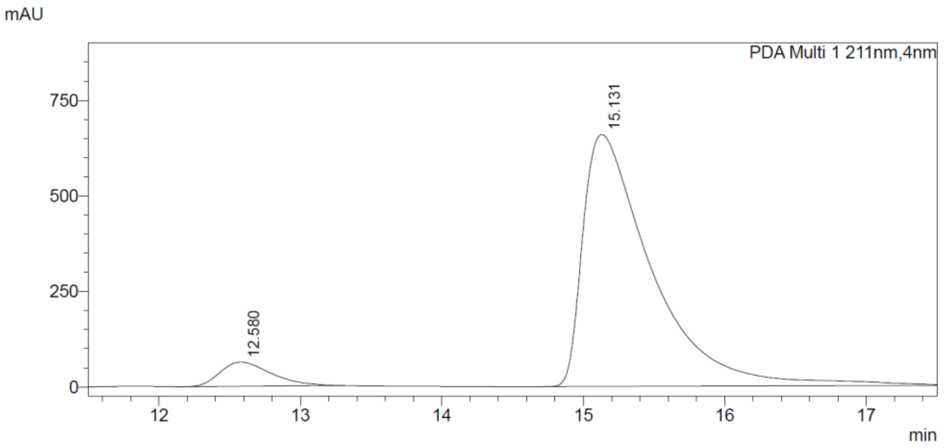


**<Peak Table>**

PDA Ch1 211nm

| Peak# | Ret. Time | Area% |
|---|---|---|
| 1 | 12.580 | 6.592 |
| 2 | 15.131 | 93.408 |
| Total | | 100.000 |

HPLC Data for **37**: Chiralcel OD-H (95:5 hexane:IPA, flow rate 1.0 mLmin$^{-1}$, 211 nm, 30 °C), $t_R$ (major): 26.5 min, >99:1 er.

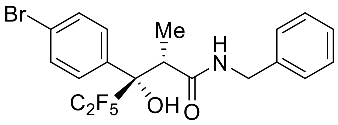


**37**

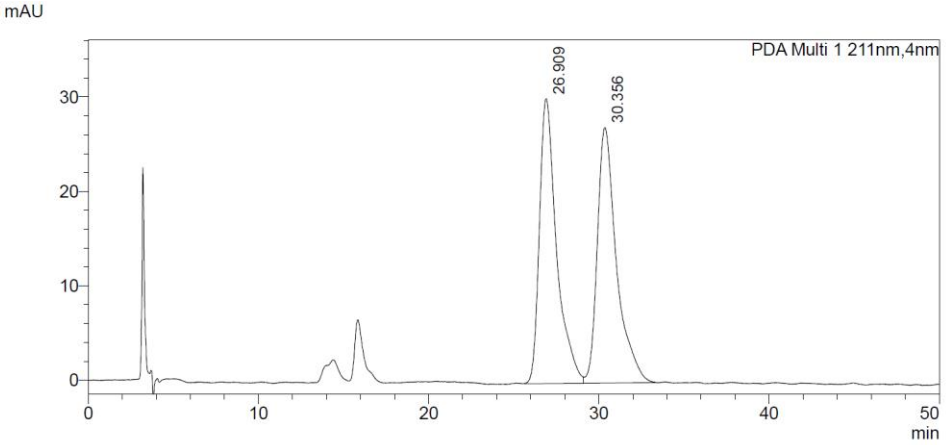


**<Peak Table>**

PDA Ch1 211nm

| Peak# | Ret. Time | Area% |
|---|---|---|
| 1 | 26.909 | 49.941 |
| 2 | 30.356 | 50.059 |
| Total | | 100.000 |

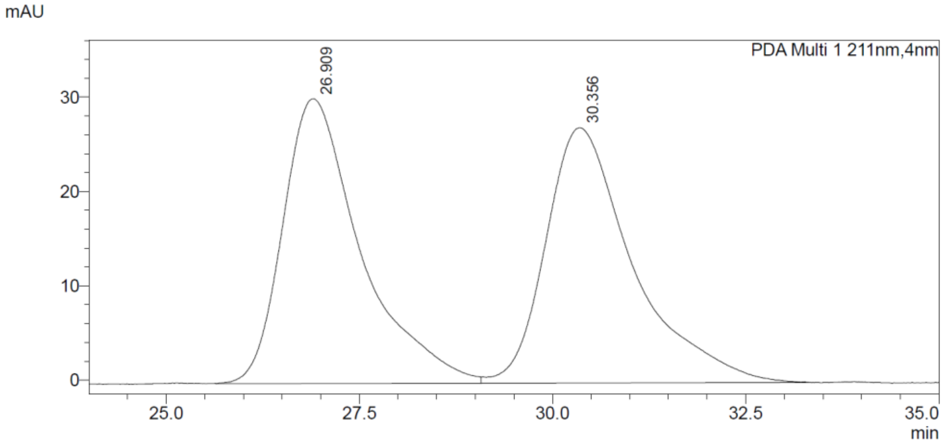


**<Peak Table>**

PDA Ch1 211nm

| Peak# | Ret. Time | Area% |
|---|---|---|
| 1 | 26.909 | 49.941 |
| 2 | 30.356 | 50.059 |
| Total | | 100.000 |

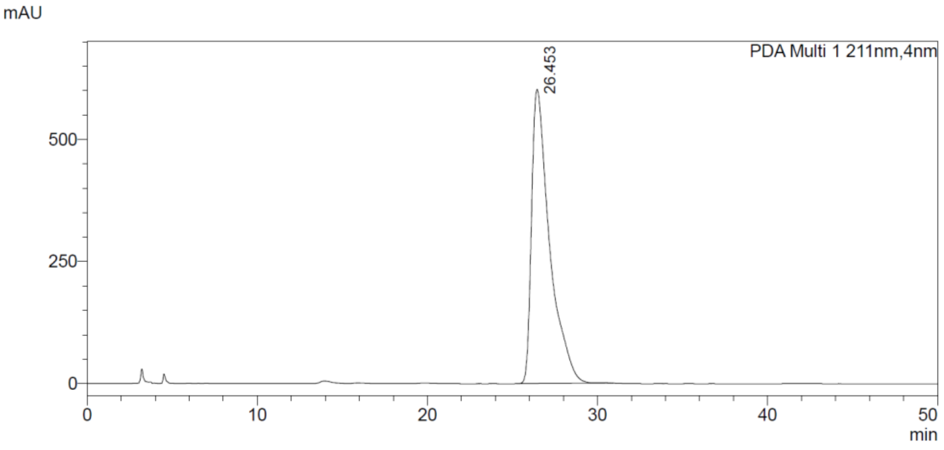


**<Peak Table>**

PDA Ch1 211nm

| Peak# | Ret. Time | Area% |
|---|---|---|
| 1 | 26.453 | 100.000 |
| Total | | 100.000 |

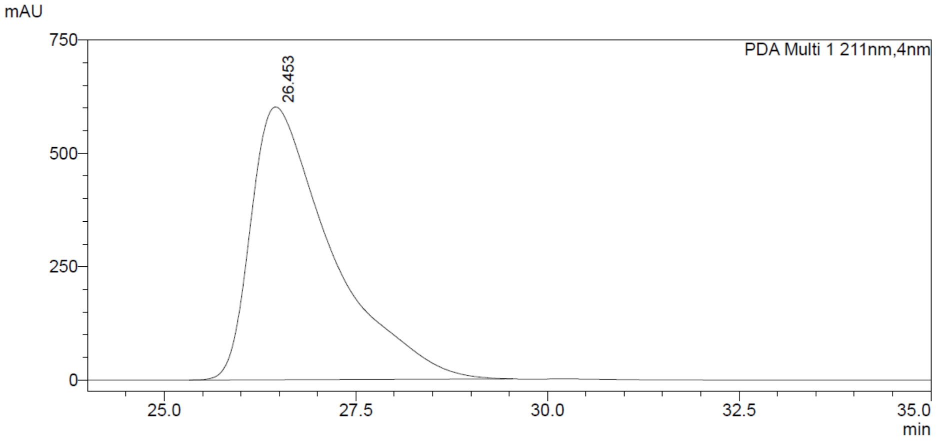


**<Peak Table>**

PDA Ch1 211nm

| Peak# | Ret. Time | Area% |
|---|---|---|
| 1 | 26.453 | 100.000 |
| Total | | 100.000 |

HPLC Data for **38**: Chiralpak AD-H (99.5:0.5 hexane:IPA, flow rate 1.0 mLmin$^{-1}$, 211 nm, 30 °C), $t_R$ (major): 23.8 min, $t_R$ (minor): 26.8 min, >99:1 er.

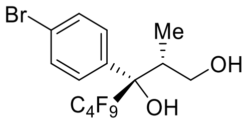


**38**

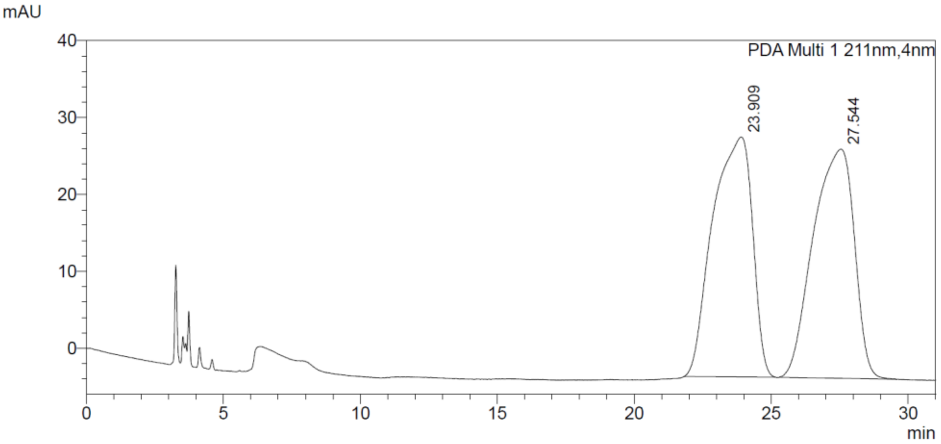


**<Peak Table>**

PDA Ch1 211nm

| Peak# | Ret. Time | Area% |
|---|---|---|
| 1 | 23.909 | 50.375 |
| 2 | 27.544 | 49.625 |
| Total | | 100.000 |

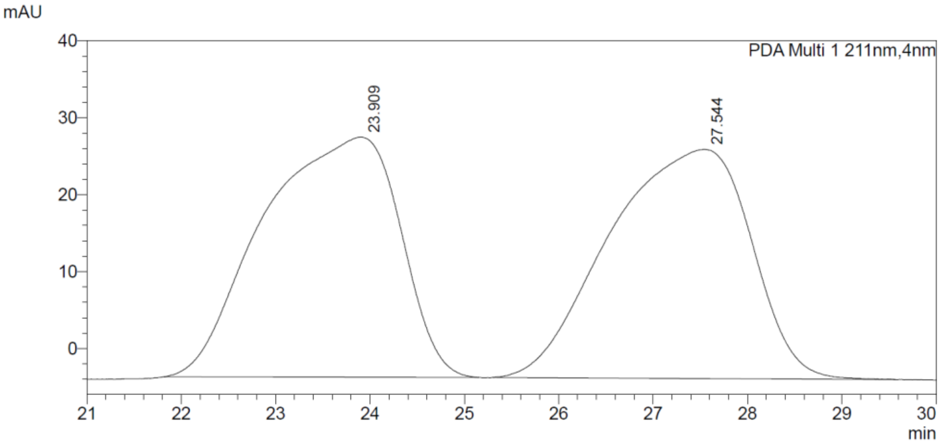


**<Peak Table>**

PDA Ch1 211nm

| Peak# | Ret. Time | Area% |
|---|---|---|
| 1 | 23.909 | 50.375 |
| 2 | 27.544 | 49.625 |
| Total | | 100.000 |

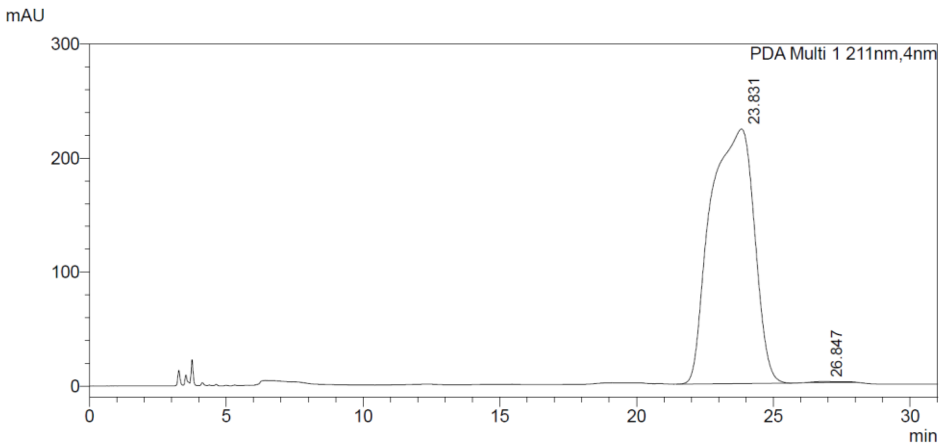


**<Peak Table>**

PDA Ch1 211nm

| Peak# | Ret. Time | Area% |
|---|---|---|
| 1 | 23.831 | 99.791 |
| 2 | 26.847 | 0.209 |
| Total | | 100.000 |

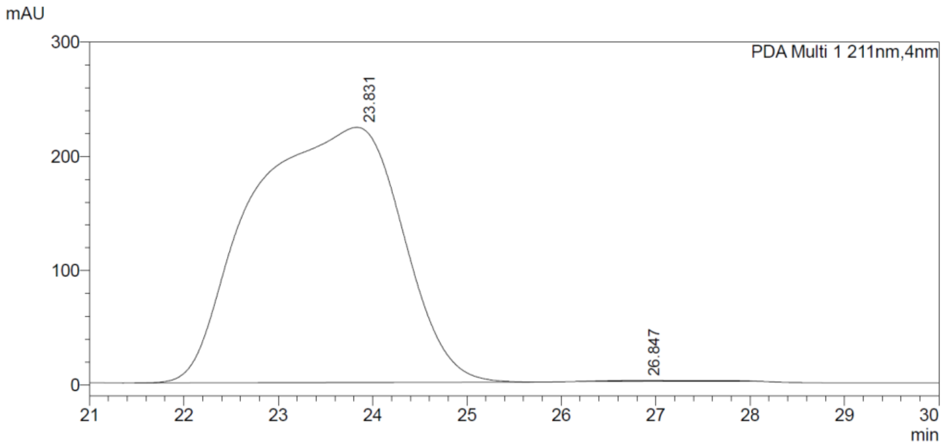


**<Peak Table>**

PDA Ch1 211nm

| Peak# | Ret. Time | Area% |
|---|---|---|
| 1 | 23.831 | 99.791 |
| 2 | 26.847 | 0.209 |
| Total | | 100.000 |

HPLC Data for **39**: Chiralpak AD-H (99.9:0.1 hexane:IPA, flow rate 1.0 mLmin$^{-1}$, 211 nm, 30 °C), $t_R$ (minor): 4.3 min, $t_R$ (major): 4.8 min, >99:1 er.

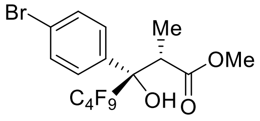


**39**

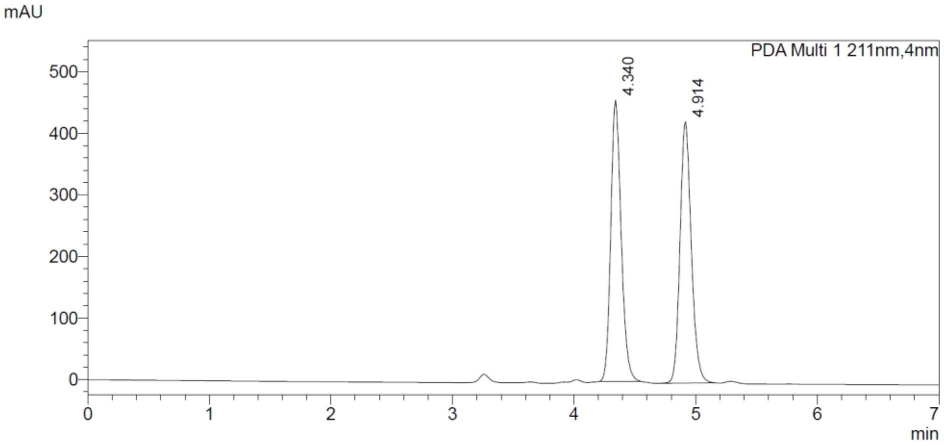


**<Peak Table>**

PDA Ch1 211nm

| Peak# | Ret. Time | Area% |
|---|---|---|
| 1 | 4.340 | 49.815 |
| 2 | 4.914 | 50.185 |
| Total | | 100.000 |

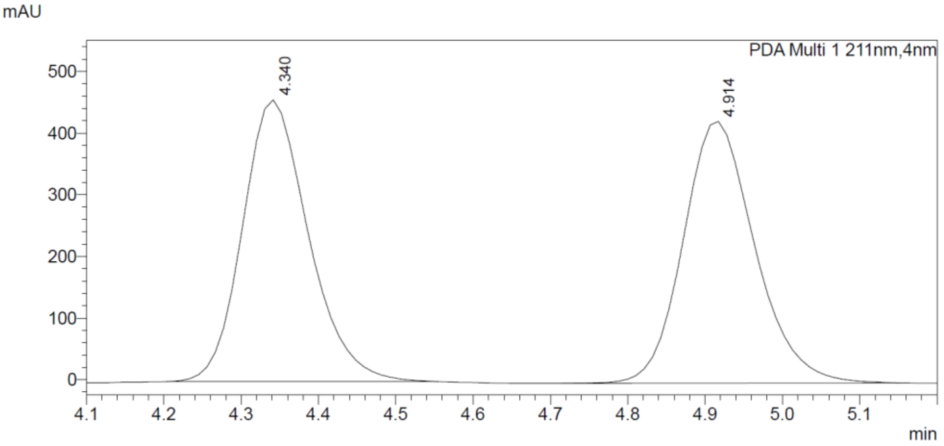


**<Peak Table>**

PDA Ch1 211nm

| Peak# | Ret. Time | Area% |
|---|---|---|
| 1 | 4.340 | 49.815 |
| 2 | 4.914 | 50.185 |
| Total | | 100.000 |

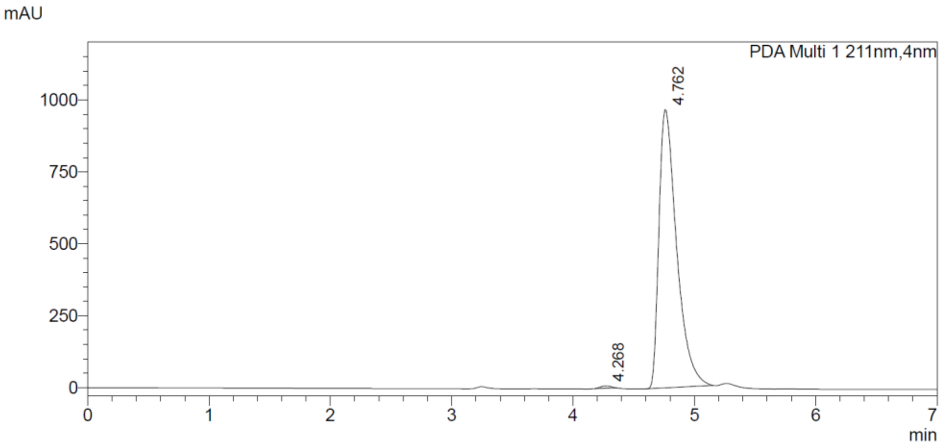


**<Peak Table>**

PDA Ch1 211nm

| Peak# | Ret. Time | Area% |
|---|---|---|
| 1 | 4.268 | 0.505 |
| 2 | 4.762 | 99.495 |
| Total | | 100.000 |

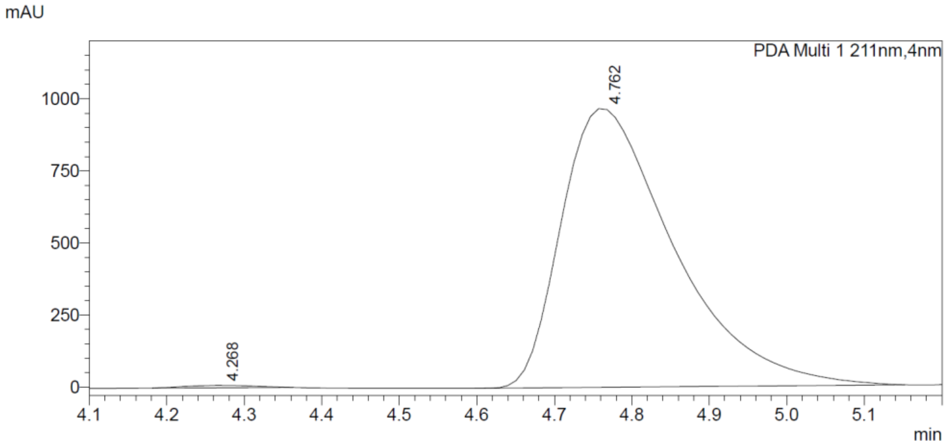


**<Peak Table>**

PDA Ch1 211nm

| Peak# | Ret. Time | Area% |
|---|---|---|
| 1 | 4.268 | 0.505 |
| 2 | 4.762 | 99.495 |
| Total | | 100.000 |
